# Transcriptome Analysis Reveals Distinct Gene Expression Profiles in Eosinophilic and Noneosinophilic Chronic Rhinosinusitis with Nasal Polyps

**DOI:** 10.1038/srep26604

**Published:** 2016-05-24

**Authors:** Weiqing Wang, Zhiqiang Gao, Huaishan Wang, Taisheng Li, Wei He, Wei Lv, Jianmin Zhang

**Affiliations:** 1 Department of Otolaryngology, Peking Union Medical College Hospital, Chinese Academy of Medical Science and Peking Union Medical College, Beijing, China; 2 Department of Immunology, Institute of Basic Medical Sciences, Chinese Academy of Medical Sciences and School of Basic Medicine, Peking Union Medical College, State Key Laboratory of Medical Molecular Biology, Beijing, China; 3 Department of Infectious Disease, Peking Union Medical College Hospital, Chinese Academy of Medical Science and Peking Union Medical College, Beijing, China

## Abstract

Chronic rhinosinusitis with nasal polyps (CRSwNP), one of the most prevalent chronic diseases, is characterized by persistent inflammation of sinonasal mucosa. However, the pathogenesis of CRSwNP remains unclear. Here, we performed next-generation RNA sequencing and a comprehensive bioinformatics analyses to characterize the transcriptome profiles, including mRNAs and long noncoding RNAs (lncRNAs), in patients with eosinophilic and noneosinophilic CRSwNP. A total of 1917 novel lncRNAs and 280 known lncRNAs were identified. We showed eosinophilic CRSwNP (ECRSwNP) and noneosinophilic CRSwNP (non-ECRSwNP) display distinct transcriptome profiles. We identified crucial pathways, including inflammatory, immune response and extracellular microenvironment, connected to the pathogenetic mechanism of CRSwNP. We also discovered key lncRNAs differentially expressed, including lncRNA XLOC_010280, which regulates CCL18 and eosinophilic inflammation. The qRT-PCR and *in situ* RNA hybridization results verified the key differentially expressed genes. The feature of distinct transcriptomes between ECRSwNP and non-ECRSwNP suggests the necessity to develop specific biomarkers and personalized therapeutic strategies. Our findings lay a solid foundation for subsequent functional studies of mRNAs and lncRNAs as diagnostic and therapeutic targets in CRSwNP by providing a candidate reservoir.

Chronic rhinosinusitis (CRS) is one of the most prevalent chronic diseases with a heavy socioeconomic burden^1^. CRS is a heterogeneous group of sinus diseases, characterized by persistent inflammation of sinonasal mucosa. Based on the absence or presence of nasal polyps, CRS is classified into either CRS with nasal polyps (CRSwNP) or CRS without nasal polyps (CRSsNP), which have distinct inflammatory and remodeling profiles^1^,^2^,^3^. CRSwNP is the most commonly studied type of CRS. Eosinophil infiltration is a typical feature of CRSwNP, found in approximately 80% of Caucasian patients and 50% East Asian patients^1^,^2^,^4^. In China, CRSwNP is divided into two subtypes with distinct immunopathologic profiles: eosinophilic chronic rhinosinusitis with nasal polyps (ECRSwNP), which is characterized by Th2-dominant inflammation and similar to the CRSwNP in Western countries; and noneosinophilic chronic rhinosinusitis with nasal polyps (non-ECRSwNP), which is characterized by Th1/Th17-dominant inflammation^2^,^5^,^6^,^7^. Compared with non-ECRSwNP, ECRSwNP demonstrates a closer correlation with high objective disease severity^8^, co-morbid asthma^9^ and the need for revision surgery^10^,^11^. Although several studies have shown specific chemokines and cytokines are crucial to eosinophil survival and differentiation in ECRSwNP^12^,^13^, the underlying pathogenetic mechanisms of ECRSwNP and non-ECRSwNP are still unclear.

Global gene expression has been studied using complementary DNA microarray techniques in CRS populations, primarily CRSwNP^14^,^15^,^16^,^17^,^18^. These studies have shed some light on the complex disease mechanism and pathways of CRSwNP, but few studies offer insights into the differential gene expression profiles between the eosinophil and noneosinophilic subtypes. Nor have they explored the underlying mechanism driving the distinct disease characteristics and prognoses. In addition, the genomic coverage of these studies was primarily limited to protein coding genes using conventional microarray techniques. Although the protein-encoding capacity of the human genome is approximately 3%, up to 74.7% of the human genome is transcribed^19^. The majority of these transcripts are categorized as long noncoding RNA (lncRNA), a heterogeneous class of noncoding transcripts longer than 200 nucleotides^20^. Emerging as a new interest in biomedical research, lncRNAs play crucial roles in physiological and pathological processes through transcriptional or posttranscriptional regulatory mechanisms, dispelling the traditional belief of “transcription noise”^21^,^22^. The roles of lncRNAs in the regulation of immunologic and inflammatory processes, such as asthma, rheumatoid arthritis and psoriasis, are just beginning to be studied^23^,^24^,^25^. However, to the best of our knowledge, lncRNAs have not been studied in research related to the human CRS transcriptome. It is crucial to conduct genome-wide analysis to discover new CRS lncRNAs to form a solid foundation for further functional analysis.

Our aim in this study was to delineate a more complete transcriptome picture and identify key pathways that are dysregulated in eosinophilic and noneosinophilic CRSwNP. Therefore, we analyzed the profiles of gene expression in nasal polyps from ECRSwNP (n = 3), non-ECRSwNP (n = 3), and normal nasal mucosa from control subjects (CTRLs; n = 3). We used next-generation high-throughput RNA sequencing (RNA-Seq), an unbiased technology that depicts the whole set of transcriptional deviations in a disease, including novel transcripts not annotated in the database or measured by conventional assays^26^ to gain new insights into the mechanisms of ECRSwNP and non-ECRSwNP. We believe these findings will be helpful in developing specific molecular biomarkers and personalized therapeutic strategies.

## Results

### RNA-Seq analysis of CRSwNP transcriptome identified mRNAs and lncRNAs

To comprehensively identify lncRNAs and mRNAs related to CRSwNP, we applied whole transcriptome strand specific RNA-Seq on rRNA-depleted RNAs from 3 ECRSwNP, 3 non-ECRSwNP and 3 CTRL sample tissues. We obtained a total of 1.017 billion raw reads, from which 969 million clean reads were isolated (Supplementary Table S1). More than 875 million read pairs (90.3%) were aligned to the human genome (hg19), where 35.4% mapped to exons, 37.3% to introns, and 27.3% to intergenic regions.

**Figure 1:**
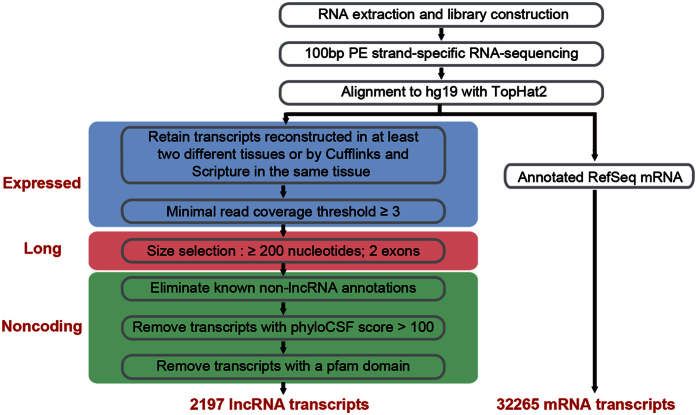
The computational pipeline for the systematic identification of CRSwNP lncRNAs and mRNAs.

**Figure 2:**
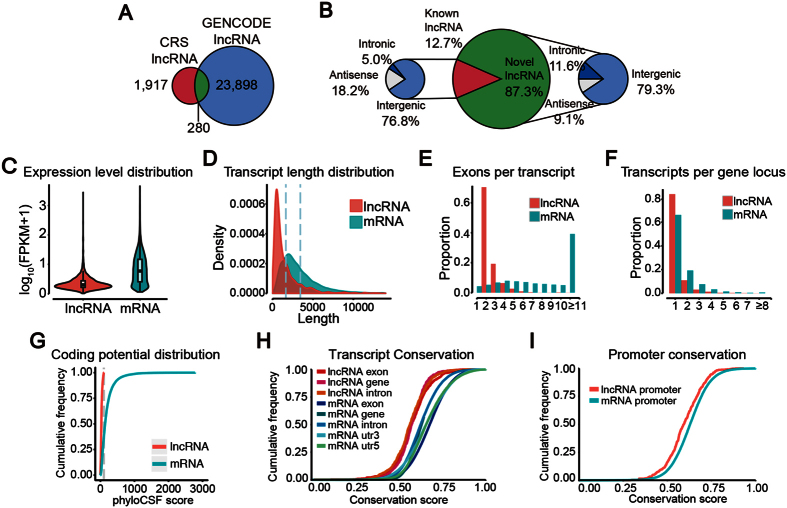
Properties of CRSwNP lncRNAs. (**A**) A venn diagram depicting the overlap between our catalog of CRSwNP lncRNAs and those in the Gencode v19. (**B**) A pie chart demonstrating the classification of identified lncRNAs. (**C**) A violin plot of expression level (showed in log_10_ (FPKM+1)) for lncRNA and mRNA transcripts. Boxes represent first and third quartiles. (**D**) Transcript size distributions for lncRNA and mRNA transcripts. (**E**) The number of exons per transcript for lncRNA and mRNA transcripts. (**F**) The number of isoforms per gene locus for lncRNAs and mRNAs. (**G**) Coding potential (phyloCSF score) of lncRNA and mRNA transcripts. (**H**) Conservation of the genomic transcript sequences for lncRNAs and mRNAs, and (**I**) of their promoters.

Then, we developed a CRSwNP transcripts computational identification pipeline. For mRNAs, we chose RefSeq database (Build 37.3) as the annotation reference. For lncRNAs, we chose GENCODE v19 database as the annotation reference. Through the transcripts identification pipeline (Fig. 1 and Supplementary Methods), we obtained 2197 reliably expressed lncRNA isoforms derived from 1747 lncRNA loci and 32265 mRNA isoforms derived from 19612 mRNA loci. The detailed information of all lncRNAs is summarized in Supplementary Table S2. Only 280 (12.7%) of all 2197 lncRNA transcripts were identified in the GENCODE v19 lncRNA annotation (Fig. 2A), indicating the presence of many novel lncRNAs in CRSwNP. The novel lncRNAs include 1521 (79.34%) long intergenic ncRNAs (lincRNAs), 222 (11.58%) intronic lncRNAs, 174 (9.08%) antisense lncRNA; the known lncRNAs include 215 (76.79%) lincRNAs, 14 (5.00%) intronic lncRNAs, 51 (18.21%) antisense lncRNA (Fig. 2B).

### The genomic characteristics of CRSwNP lncRNAs

To examine chromatin features of lncRNA loci, we analyzed the publically available epigenetic modification information across nine ENCODE cell lines^27^ at the genomic regions encoding the 2197 lncRNAs detected in our study. Most of these genomic regions contain DNase I hypersensitivity loci (n = 1974, 90%) and epigenetic markers signaling active transcription, including histone H3 lysine 4 trimethylation (H3K4me3, n = 1625, 74%), a modification associated with promoters^27^, and lysine 27 acetylation (H3K27Ac, n = 1728, 79%), associated with active regulatory regions^27^. These results, together with previous studies^27^,^28^, suggest that lncRNAs are independent transcripts encoded from genomic regions with active transcriptional activity.

We characterized the basic features of the lncRNAs and compared them with protein-coding genes where appropriate. Our data show that average lncRNA expression was lower than protein-coding gene expression (Fig. 2C). LncRNAs had smaller sizes and fewer exons than mRNAs. On average, lncRNAs were 1644 bp long and had 2.47 exons, whereas mRNAs were 3433 bp long and had 11.49 exons (Fig. 2D,E). Both lncRNAs and mRNAs were alternatively spliced. On average, there were 1.26 isoforms per lncRNA locus and 1.65 isoforms per mRNA locus (Fig. 2F). PhyloCSF coding potential analysis showed lncRNAs had very little protein-coding potential compared with mRNAs (Fig. 2G). Conservation analysis revealed that lncRNA sequence conservation was lower than that of mRNA (Fig. 2H), a similar case was found in the promoter regions (Fig. 2I). In summary, we identified lncRNA transcripts that displayed distinct genomic features compared with mRNA transcripts.

### Patients with ECRSwNP and those with non-ECRSwNP have distinct gene expression profiles and cluster separately

**Figure 3:**
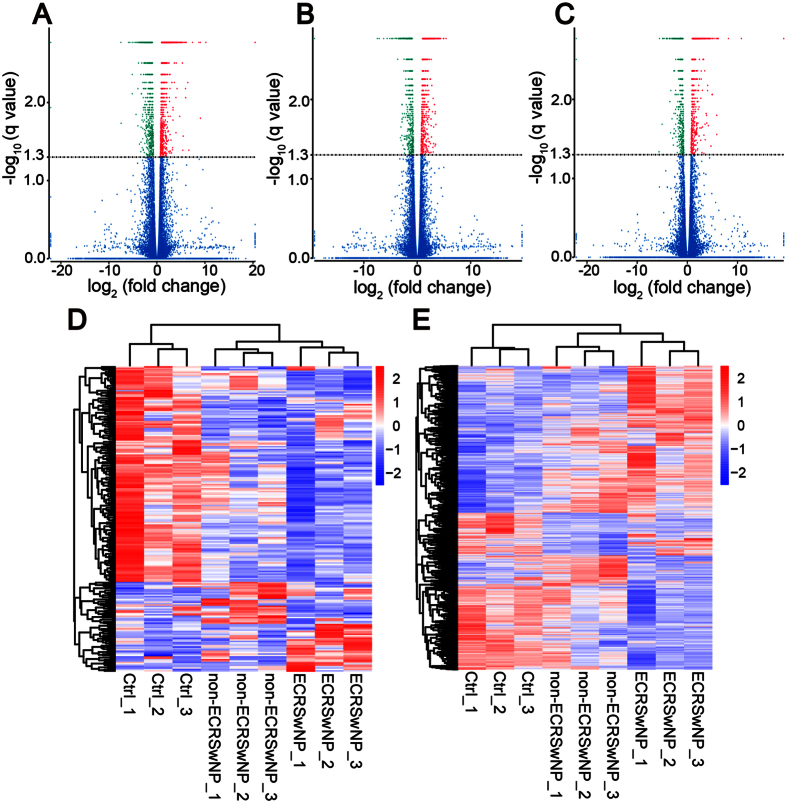
Transcriptome profile of RNA-Seq data distinguishing three groups. (**A**) A volcano plot of differentially expressed transcripts (lncRNAs and mRNAs) between ECRSwNP and control group, and (**B**) between non-ECRSwNP and control group, and (**C**) between ECRSwNP and non-ECRSwNP group, respectively. (**D–E**) Unsupervised hierarchical clustering of the expression profiles of differentially expressed lncRNAs (**D**) and mRNA (**E**) both distinguish ECRSwNP, non-ECRSwNP and control group.

In the ECRSwNP group, 151 lncRNA transcripts (26 up-regulated and 125 down-regulated) and 1921 mRNA transcripts (1058 up-regulated and 863 down-regulated) were differentially expressed compared to the control group (Fig. 3A and Supplementary Table S3). In non-ECRSwNP, 99 lncRNA transcripts (23 up-regulated and 76 down-regulated) and 1155 mRNA transcripts (647 up-regulated and 508 down-regulated) were differentially expressed compared to the control group (Fig. 3B and Supplementary Table S4). And in the ECRSwNP group, 62 lncRNA transcripts (22 up-regulated and 40 down-regulated) and 1173 mRNA transcripts (606 up-regulated and 567 down-regulated) were differentially expressed compared to the non-ECRSwNP group (Fig. 3C and Supplementary Table S5). As shown in Supplementary Fig. S1 and Supplementary Table S2, the differentially expressed lncRNAs and mRNAs are widely distributed throughout almost every chromosome. In unsupervised hierarchical clustering analysis, heat maps were generated using the differentially expressed lncRNAs and mRNAs respectively and they clearly self-segregated into ECRSwNP, non-ECRSwNP and control clusters (Fig. 3D,E). The mRNAs with more than 16-folds difference between ECRSwNP and non-ECRSwNP are listed in Table 1, of which 30 were up-regulated in ECRSwNP and 13 of those were up-regulated in non-ECRSwNP. These results reflect distinct lncRNA and mRNA expression profiles between these groups, implying distinct underlying pathophysiology in ECRSwNP and non-ECRSwNP.

### Experimental validation of novel lncRNA transcripts

**Figure 4:**
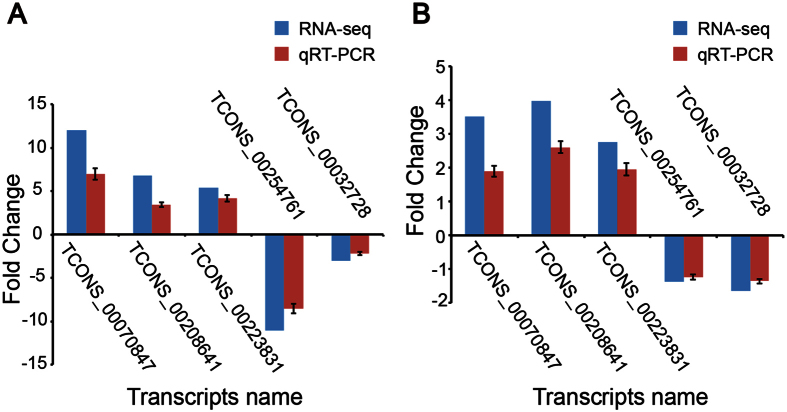
Confirmation of the expression patterns of lncRNAs using qRT-PCR. Five random differentially expressed lncRNAs were validated by qRT-PCR in an independent cohort of 32 ECRSwNP subjects, 28 non-ECRSwNP subjects and 31 control subjects. Each sample was detected in triplicate. *GAPDH* was used as the reference gene. The heights of columns represent the fold changes compared with control group. Bars represent S.E.M. The trends of qRT-PCR results are consistent with RNA-seq data.

To confirm the reliability of our RNA-Seq data, we randomly selected five lncRNAs transcripts from all dysregulated lncRNAs and analyzed expression levels by qRT-PCR in another independent cohort of 32 ECRSwNP subjects, 28 non-ECRSwNP subjects and 31 control subjects. The qRT-PCR fold change values for each lncRNA were calculated and compared with data obtained from RNA-Seq. The qRT-PCR results were consistent with the RNA-Seq data with the same trends (up- or down-regulated) for each lncRNA (Fig. 4), confirming the accuracy and reliability of RNA-Seq analyses.

### Dysfunction of inflammatory and immune response and extracellular microenvironment are two central characteristics in CRSwNP

To ascertain the functions of the differentially expressed mRNAs and connections among them, we performed GO term and KEGG pathway enrichment analyses. We drew directed acyclic graphs (DAGs) for the significantly over-represented GO terms to clarify the relationship (Supplementary Fig. S2), in which the downstream term is a subset of the upstream term. In GO analysis of the differentially expressed mRNAs between ECRSwNP and control group, the significantly over-represented terms are shown in Supplementary Fig. S2A–C, including the following biological process terms: immune system processes, microtubule-based movement, cell adhesion, and cellular complex assembly; cellular component terms: extracellular matrix, cytoskeleton part and dynein complex, and membrane; molecular function terms: metallopeptidase activity, microtubule motor activity, cytokine activity, chemokine activity (associated with G-protein coupled receptor binding), and calcium ion binding. Comparing non-ECRSwNP and control group, the significantly over-represented terms are shown in Supplementary Fig. S2D–F, which had less terms than the ECRSwNP and control group, including the following biological process terms: immune response; cellular component terms: extracellular matrix and membrane; molecular function terms: metallopeptidase activity, cytokine activity, and chemokine activity (associated with G-protein coupled receptor binding). Comparing ECRSwNP and non-ECRSwNP group, the significantly over-represented terms are shown in Supplementary Fig. S2G–I, including the following biological process terms: immune response, cell adhesion, cell recognition, microtubule-based movement; cellular component terms: cell periphery, dynein complex, extracellular matrix; molecular function terms: metalloendopeptidase activity, cytokine activity, and chemokine activity (associated with G-protein coupled receptor binding). For instance, by using GO analyses, we found IL-6 and oncostatin M (OSM), which are associated with barrier function of airway epithelium in eosinophilic mucosal inflammation, were specifically highly expressed in ECRSwNP in the GO term “cytokine activity”, and IFN-γ, which is a critical cytokine in type 1 immune response, was specifically highly expressed in non-ECRSwNP in the GO term “immune response”.

**Figure 5:**
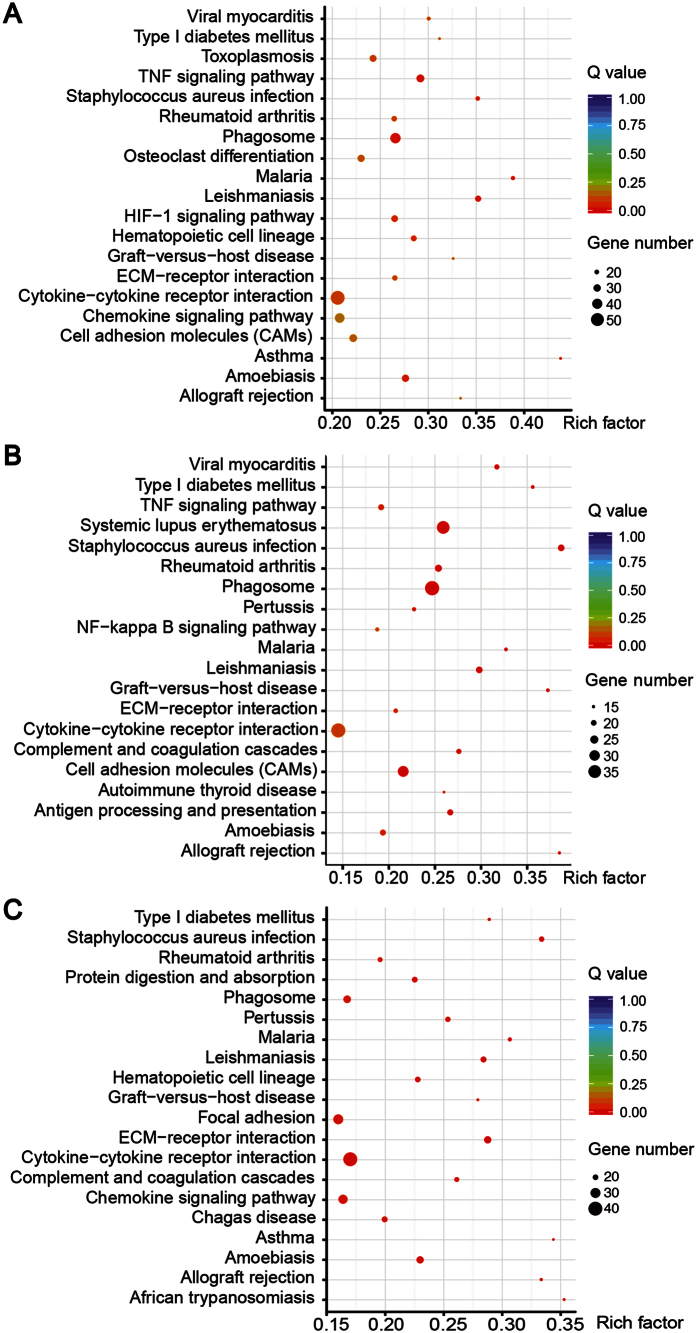
KEGG pathway analyses of dysregulated protein-coding genes. (**A–C**) Analysis of top 20 over-represented KEGG pathways between ECRSwNP and CTRL group (**A**), and between non-ECRSwNP and CTRL group (**B**), and between ECRSwNP and non-ECRSwNP group (**C**), respectively. The size of each circle stands for the number of significantly differentially expressed genes enriched in corresponding pathway. The rich factor was calculated using the number of enriched genes divided by the number of all background genes in corresponding pathway. *Q* value was calculated using the Benjamini–Hochberg correction. The pathway showing *q* value < 0.05 are to be considered as statistically significantly over-represented.

The KEGG pathway analyses mapped the dysregulated protein-coding genes to KEGG reference pathways to infer systemic biological behaviors. The KEGG pathway analysis of the differentially expressed mRNAs between ECRSwNP and control group revealed that phagosome, TNF signaling pathway, staphylococcus aureus infection, and asthma are significantly over-represented (Fig. 5A); comparing non-ECRSwNP and control group, staphylococcus aureus infection, phagosome, antigen processing and presentation, cell adhesion molecules, TNF signaling pathway ECM-receptor interaction are significantly over-represented (Fig. 5B); comparing ECRSwNP and non-ECRSwNP group, extracellular matrix (ECM) receptor interaction, staphylococcus aureus infection, asthma, cytokine-cytokine receptor interaction, chemokine signaling pathway, focal adhesion, and phagosome are significantly over-represented (Fig. 5C). Other unrelated inflammatory disease pathways may be enriched given common inflammatory molecules.

Although the same over-represented terms appeared in multiple comparison groups, the genes enriched in the terms were distinct among different comparison groups. For example, cytokine activity and chemokine activity were over-represented in all three comparison groups, but IL-13 and CCL26 were specifically highly expressed in ECRSwNP patients, while CXCL1 and CXCL5 were specifically highly expressed in non-ECRSwNP patients (Supplementary Table S3–S5). Therefore, when studying the pathophysiology of CRSwNP, specific genes in the over-represented terms should be taken into consideration.

**Figure 6:**
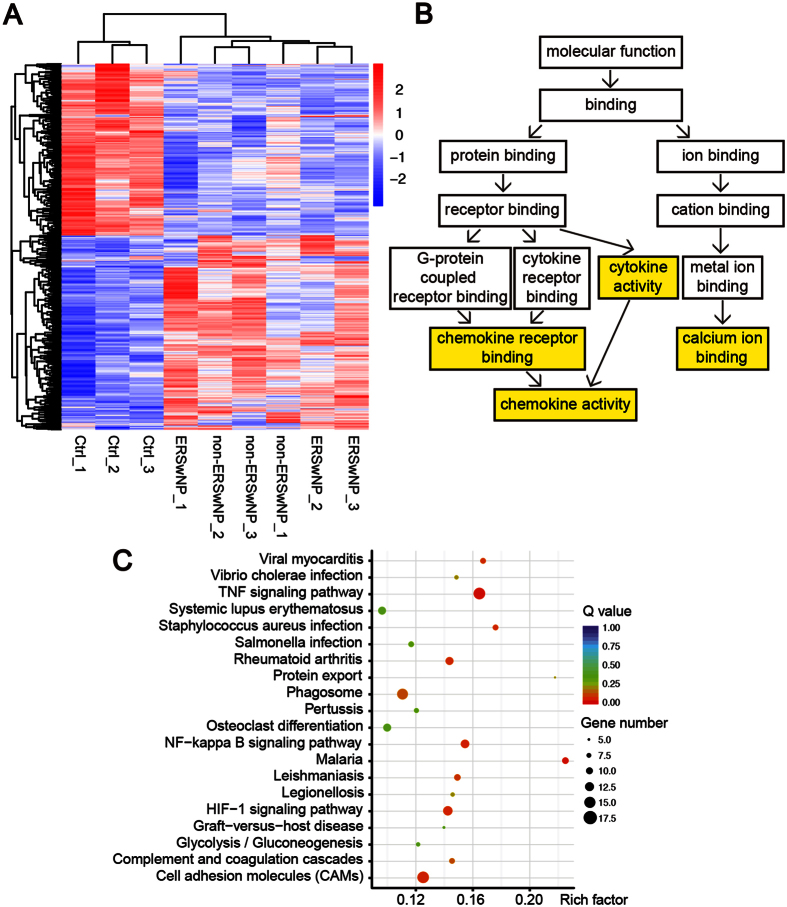
The common dysregulated protein-coding genes of ECRSwNP and non-ECRSwNP compared with Control group. (**A**) Unsupervised hierarchical clustering of the expression profiles of the common differentially expressed genes could divide subjects into CRTL and CRSwNP group, but could not distinguish between ECRSwNP with non-ECRSwNP. (**B**) The directed acyclic graph (DAG) of GO analysis of the common differentially expressed genes. The statistically over-represented GO terms are marked in yellow. (**C**) Top 20 over-represented KEGG pathways of the common differentially expressed genes.

To gain insights into the common pathophysiological mechanism of ECRSwNP and non-ECRSwNP, we analyzed the 623 common dysregulated mRNAs (Supplementary Table S6) of the 1921 ECRSwNP and 1155 non-ECRSwNP differentially expressed mRNAs compared with control group. Unsupervised hierarchical clustering analysis demonstrated that these common dysregulated mRNAs separated normal control from CRSwNP, but did not distinguish between ECRSwNP and non-ECRSwNP (Fig. 6A). This result suggests they may serve as biomarkers for CRSwNP and reflect common pathophysiological mechanisms. The significant over-represented GO term included cytokine activity, chemokine activity, and calcium ion binding (Fig. 6B). The top enriched KEGG pathways included TNF signaling pathway, NF-κB signaling pathway, HIF-1 signaling pathway, cell adhesion molecules, and staphylococcus aureus infection (Fig. 6C), indicating that these pathways may play a crucial role in the pathogenesis of CRSwNP.

### Prediction of lncRNA function

Most of the known lncRNAs are not functionally annotated in current databases. Previous studies demonstrated that lncRNAs act in *cis* and regulate the expression of nearby protein-coding genes^22^,^29^. Other studies revealed that lncRNAs act in *trans* and regulate the expression of remote genes^29^,^30^. To evaluate the potential *cis* regulatory function of lncRNAs, we calculated the Pearson correlation coefficient (PCC) between the expression levels of lncRNAs and neighboring protein-coding genes (lncRNA:*cis*-mRNA pairs) and compared with those of mRNA:*cis*-mRNA pairs and random protein-coding gene pairs (Supplementary Fig. S3). There was no obvious positive or negative correlation between random gene pairs (mean PCC = −0.004). Both lncRNA:*cis*-mRNA pairs (mean PCC = 0.107) and mRNA:*cis*-mRNA pairs (mean PCC = 0.127) had a significantly positive correlation (both P < 2.2e-16, Student’s t-test). However, we did not observe a stronger correlation of lncRNA:*cis*-mRNA pairs compared with mRNA:*cis*-mRNA pairs (P  = 0.004, Student’s t-test). Taken together, these results suggest that lncRNAs perform a *cis* regulatory role on neighboring protein-coding genes, but not stronger than that of mRNAs. We performed the GO enrichment analysis of *cis*-mRNAs to predict the function of dysregulated lncRNAs, but no significant enrichment was found since the number of *cis*-mRNAs was relatively small.

Numerous lncRNAs regulate their target protein-coding genes in *trans*. The function of differentially expressed lncRNAs can be predicted by their closely correlated *trans*-regulated target protein-coding genes^31^. Each lncRNA may have multiple *trans*-regulated potential target protein-coding genes. To clarify the functional categories with which the differentially expressed lncRNAs were associated, we performed GO enrichment analysis on their *trans*-regulated target mRNAs (Supplementary Fig. S4). Comparing ECRSwNP and control group, the significantly over-represented terms are shown in Supplementary Fig. S4A–C, including the following biological process terms: regulation of cellular process, cell communication, cellular response to stimulus, signal transduction (these four terms share the common down-stream term, G-protein coupled receptor signaling pathway, to perform their biological function), and microtubule-based movement; cellular component terms: cytoskeleton part and dynein complex, endomembrane system, and integral to membrane; molecular function terms: microtubule motor activity, ion channel activity, G-protein coupled receptor activity. Comparing non-ECRSwNP and control group, the significantly over-represented terms are shown in Supplementary Fig. S4D–F, including the following biological process terms: cell communication, cellular response to stimulus, signaling (these three terms share the common down-stream term, G-protein coupled receptor signaling pathway, to perform their biological function), oligosaccharide biosynthetic process, and microtubule-based movement; cellular component terms: dynein complex, endomembrane system, and integral to membrane; molecular function terms: microtubule motor activity, extracellular ligand-gated ion channel activity, G-protein coupled receptor activity. Comparing ECRSwNP and non-ECRSwNP group, the significantly over-represented terms are shown in Supplementary Fig. S4G–I, including the following biological process terms: regulation of cellular process, cell communication, cellular response to stimulus, signal transduction (these four terms share the common down-stream term, G-protein coupled receptor signaling pathway, to perform their biological function); cellular component terms: dynein complex, and integral to membrane; molecular function terms: microtubule motor activity, channel activity, G-protein coupled receptor activity, cytokine activity and carbohydrate binding. The predicted function categories of dysregulated lncRNAs are closely related to those of the dysregulated protein-coding genes, indicating the potential strong connection and interaction between lncRNAs and mRNAs.

### Significant Correlation between lncRNA XLOC_010280 and CCL18

**Figure 7:**
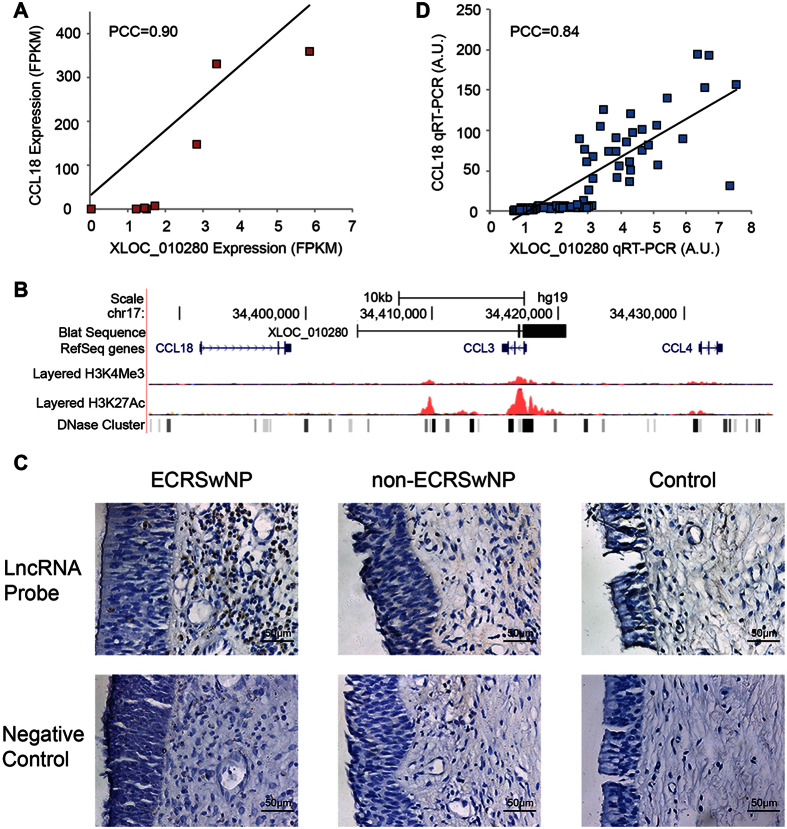
Significant correlation between lncRNA XLOC_010280 and CCL18. (**A**) A scatterplot of XLOC_010280 and CCL18 expression levels in individual nasal samples determined by RNA-Seq. (**B**) The genomic location of XLOC_010280 and CCL18 shown by UCSC genome browser. (**C**) The specific up-regulation of XLOC_010280 in ECRSwNP compared with non-ECRSwNP and control detected by *in situ* hybridization. Representative of 5 donors per group shown as original magnification 200×. (**D**) A scatterplot of XLOC_010280 and CCL18 expression levels in individual nasal samples determined by qRT-PCT.

In *cis* prediction of functions of lncRNAs, we found an interesting lncRNA, XLOC_010280, and its predicted *cis*-regulated target CCL18 were highly correlated (PCC = 0.90, Fig. 7A). They are both located on chromosome 17q11.2-q12 CC chemokine gene cluster (Fig. 7B). Many CC chemokine family genes located in this 17q11.2-q12 gene cluster likely contribute strongly to eosinophilic inflammation^32^. *In situ* hybridization demonstrated that XLOC_010280 was specifically expressed in ECRSwNP, whereas no significant signals were observed in non-ECRSwNP and control group (Fig. 7C). The results of qPCR performed in an independent cohort of patients confirmed their close correlation (PCC = 0.84, Fig. 7D). These data indicate a strong association between XLOC_010280 and CCL18.

## Discussion

ECRSwNP and non-ECRSwNP are two subtypes of CRSwNP, which display different disease severities, inflammation profiles and prognoses^2^,^4^,^5^,^6^,^8^,^9^,^10^. Therefore, understanding the underlying mechanism is crucial to identify new biomarkers, personalize treatment regiments and improve prognoses. To our knowledge, this is the first study to perform genome-wide comparison of gene expression patterns between ECRSwNP and non-ECRSwNP. And it is also the first to systematically identify and analyze the lncRNAs associated with ECRSwNP and non-ECRSwNP. In our study, we found ECRSwNP and non-ECRSwNP possess distinct transcriptome profiles and lncRNA XLOC_010280 plays a crucial role in eosinophilic inflammation by regulating CCL18 expression. Our findings on the transcriptome analysis of CRS subtypes provide us with an overall vision of molecular changes between CRSwNP subtypes and useful clues for subsequent CRS research.

Three sample groups have distinct gene expression profiles and self-segregated in unsupervised hierarchical clustering analysis. This suggests different disease pathogeneses of the two CRSwNP subtypes and offers promise for developing new biomarkers of the two subtypes. When analyzing the dysregulated mRNAs in our study, we found that almost all significantly enriched GO terms and KEGG pathways were related with two central characteristics of CRSwNP: one is inflammatory and immune response, such as cytokine and chemokine activities; the other is regional microenvironment change in nasal polyps, such as extracellular matrix (ECM) and metallopeptidase activity. In the common differentially expressed mRNAs analysis, ECRSwNP and non-ECRSwNP share many common dysregulated genes in inflammatory and immune response and extracellular matrix microenvironment, including staphylococcus aureus infection, HIF-1 signaling pathway, and cell adhesion molecules, but they also have their own distinctive characteristics. For instance, on the cytokine and chemokine side, we found IL-13 and eotaxins (CCL11, CCL24, CCL26) highly expressed in ECRSwNP, while IFN-γ and IL-8 highly expressed in non-ECRSwNP (Supplementary Tables S3–S5). This suggests ECRSwNP has Th2 skewed inflammation and more eosinophil infiltration, whereas non-ECRSwNP has Th1 skewed inflammation and more neutrophil infiltration, which is consistent with previous studies^1^,^2^. Moreover, we found that the IFN-γ-inducible chemokines CXCL9, CXCL10 and CXCL11 are all specifically up-regulated in non-ECRSwNP. These three chemokines are all CXCR3 ligands. CXCL9 and CXCL10 induce effector Th1/Th17 cells to promote inflammation, while CXCL11 drives the development of the T regulatory 1 cell subset and dampens inflammation in autoimmune encephalomyelitis mice^33^. These results imply CXCL9, CXCL10 and CXCL11 play a critical role in the Th1/Th17 inflammation in non-ECRSwNP. On the regional microenvironment side, ECRSwNP had higher expression levels of matrix metallopeptidase 1 (MMP1) than non-ECRSwNP (Supplementary Table S5). MMP1, which is also known as interstitial collagenase, degrades extracellular matrix collagens types I, II and III^34^. The up-regulated expression of MMP1 in ECRSwNP may be related to the phenomenon reported previously that eosinophilic nasal polyps display edema and minimal collagen deposition, but non-eosinophilic nasal polyps manifest dense collagen deposition^35^.

We compared the most differentially expressed mRNAs between ECRSwNP and non-ECRSwNP in our study (Table 1) with previous CRS studies. Consistent with previous studies, some of these differentially expressed genes were specifically highly expressed in ECRSwNP, such as CCL23^36^ and OSM^37^, which supports the accuracy and reliability of our study. Furthermore, we found the roles of many differentially expressed genes have not been characterized in CRS, such as amphiregulin (AREG) and neurotensin (NTS). Or although studied before, the roles of many of these genes have not been characterized between eosinophilic and non-eosinophilic subtypes, such as serum amyloid A^38^ (SAA, including SAA1 and SAA2) and IL-6^39^. Therefore, our study provides numerous candidate targets for future pathophysiological research of CRSwNP. For example, innate lymphoid cells (ILCs) represent an emerging family of cell types that seem to have crucial roles in innate immunity and tissue remodeling^40^,^41^,^42^. In contrast to lung, gut and healthy nasal tissues and non-eosinophilic nasal polyps, relatively high proportions of type 2 ILCs (ILC2s) were found in nasal polyps of Caucasian ECRSwNP, which is characterized by eosinophilia and type 2 immune response^40^,^41^,^42^. Type 2 ILCs (ILC2s) have been implicated in the initiation and coordination of type 2 immune responses in response to epithelial cell-derived IL-25 and IL-33 and shown to produce the canonical Th2 cytokines IL-5, IL-9 and IL-13^40^,^41^. A recent study^43^ revealed a previously unrecognized pathway of innate immune cell-mediated tissue protection in which IL-33 ameliorated disease and restored epithelial barrier function through induction of ILC2s and in an AREG-dependent manner^43^. Based on these previous findings and our data, the specific AREG up-regulation in ECRSwNP may play a critical part in eosinophilic inflammation and tissue remodeling. The manipulation of the IL-33–ILC2–AREG pathway may provide new therapeutic benefits in treatment of ECRSwNP. NTS up-regulates the expression level of HIF-1α and VEGFα and promotes angiogenesis in inflammatory bowel disease^44^. In our study, NTS and VEGFα is specifically highly expressed in ECRSwNP, HIF-1α was highly expressed both in ECRSwNP and non-ECRSwNP. Although the HIF-1α levels were higher in ECRSwNP than non-ECRSwNP (FPKM value 113 v.s. 72), no statistical significance was reached because of limited sample size (Supplementary Table S5). The obstruction of the middle meatus and sinus ostia in CRSwNP may result in tissue hypoxia and induce the expression of HIF-1α and subsequent numerous inflammatory genes including VEGFα, which is associated with angiogenesis, epithelial cell overgrowth and nasal polyposis^45^,^46^. Thus, the higher NTS level in ECRSwNP may be related to its more active epithelial cell proliferation and higher recurrence rate through up-regulating the levels of HIF-1α and VEGFα. The level of SAA was significantly up-regulated in Chinese CRSwNP patients compared with the controls^38^, but the expression levels in eosinophilic and non-eosinophilic subtypes were not clear. SAA stimulates the production of cytokines related to netrophilic inflammation, including IL-8 and IL-17A, and enhances chemotaxis of neutrophils^38^,^47^. Furthermore, given the fact that the Chinese CRSwNP patients demonstrate less eosinophilia and more neutrophilia^1^,^2^, our data indicates the higher level of SAA in non-ECRSwNP may contribute to the enhanced neutrophil infiltration compared with ECRSwNP. Although elevated IL-6 expression was shown in CRSwNP^39^, the expression levels in eosinophilic and non-eosinophilic subtypes have not been reported. OSM is a member of IL-6 family of cytokines and its levels were increased and could decrease the barrier function of airway epithelium in eosinophilic mucosal diseases including CRSwNP by a recent study^37^. Based on the fact IL-6 and OSM come from the same cytokine family and, and our data also showed IL-6 levels significantly increased in ECRSwNP, indicating that IL-6 plays an important role in the pathophysiology of eosinophilic inflammation in ECRSwNP. These hypotheses are worth being tested and may give us new insights into the pathophysiology of CRSwNP and provide new medical targets for us.

Among the numerous uncharacterized transcripts identified by our study, we obtained 1917 novel and 280 known GENCODE v19 lncRNAs transcripts through our pipeline. The high ratio of novel to known lncRNAs may have resulted from the high tissue specificity of lncRNA^20^,^28^ and the rarity of lncRNA studies of sinonasal diseases. The qRT-PCR results were consistent with the RNA-Seq results and showed the same trends of changes in expression levels, confirming the reliability of the RNA-Seq data. Recent studies have demonstrated that lncRNAs regulate gene expression on multiple levels, either in *cis* or in *trans*^29^. In the present study, we explored the potential functions of the dysregulated CRSwNP lncRNAs by *cis*- and *trans*- regulated target protein-coding genes. The neighbor gene correlation suggests that lncRNAs perform a *cis* regulatory role on neighboring protein-coding genes, but not stronger than that of mRNAs. The enrichment of *cis*-regulated target protein-coding genes was not significant because of limited number of target genes. The *trans*-regulated target protein-coding genes of the three comparison group commonly enriched in terms about cellular process regulation, cell communication and signal transduction, cell movement and migration, and cytokine activity. The identified enriched function categories of dysregulated lncRNAs are closely related to those of the dysregulated protein-coding genes, suggesting that lncRNAs play an important part in regulating the expression of protein-coding genes in CRSwNP.

When predicting functions of lncRNAs, we identified an interesting lncRNA XLOC_010280 and its predicted *cis*-regulated target CCL18 were highly correlated. CCL18 plays a vital role in Th2 inflammation and is mainly produced by M2 macrophage in nasal polyps in Caucasian CRSwNP patients^48^. Because Caucasian CRSwNP is characterized by eosinophilia and Th2 inflammation^1^,^49^, the scholars posed an interesting question asking whether elevated CCL18 expression exists in patients with non-ECRSwNP in China to testify the role of CCL18 in eosinophilia and Th2 inflammation^48^. Our data answered this question appropriately by showing that CCL18 levels in ECRSwNP were more than 71-folds higher than those in non-ECRSwNP (Adjusted *P* value < 0.0017) (Table 1). Furthermore, we found an lncRNA XLOC_010280 was closely related to its predicted *cis*-regulated target CCL18 (PCC = 0.89). The results of qRT-PCR and *in situ* hybridization showed that XLOC_010280 was also highly expressed in ECRSwNP. Both XLOC_010280 and CCL18 are located in chromosome 17q11.2-q12 CC chemokine gene cluster (Fig. 7B). This gene cluster also includes CCL3, CCL4, CCL5, CCL23, and has a strong correlation with eosinophilic inflammation^32^. These findings suggest that XLOC_010280 play a role in eosinophilic inflammation by regulating the expression level of CCL18.

There are several advantages of using RNA-Seq over microarrays: (1) the ability to identify novel transcripts, not confined to annotated transcripts in databases; (2) sensitive detection and reliable quantification of coding and noncoding transcripts, which is particularly important because of the low expression level of lncRNAs. However, our study has several limitations. First, the sample size was relatively small and the gender of the RNA-Seq subjects was heterogeneous. Nevertheless, the consistency between our results and the previous research^1^,^12^,^14^,^16^,^35^,^36^,^37^,^38^,^39^,^45^,^46^,^48^ and the confirmative qRT-PCR results suggest that many of the differentially expressed mRNAs and lncRNAs detected in our study are disease-specific. Second, we provided indirect experimental evidence to imply the functional link between lncRNA and its predicted target gene. Therefore, we will confirm our RNA-Seq data in larger cohorts of well-controlled subjects and illustrate the functional role of lncRNAs with direct evidence in subsequent research.

In conclusion, the dysfunction of inflammatory and immune response and extracellular microenvironment are the key pathogenic mechanisms of CRSwNP. Differences in the pathophysiology of two subtypes of CRSwNP demonstrate the significance and necessity of developing specific biomarkers and personalized therapeutic strategies. The high consistency between predicted functions of dysregulated lncRNAs and functions of dysregulated mRNAs indicates lncRNAs play a critical role in regulating the expression of protein-coding genes in CRSwNP. This study lays a solid foundation for subsequent functional studies of mRNAs and lncRNAs as diagnostic and therapeutic targets in CRSwNP by providing a candidate reservoir.

## Methods

An expanded Method section is available in the Supplementary Information.

### Patients and biopsy specimens

The study was approved by the Ethics Committee of Peking Union Medical College Hospital. All study participants provided written informed consent and were recruited at Peking Union Medical College Hospital. All experiments were performed in accordance with approved guidelines. A total of 35 patients with ECRSwNP, 31 patients with non-ECRSwNP and 34 control subjects were recruited for the study. The diagnosis of CRSwNP was based on standard criteria issued in the European Position Paper on Rhinosinusitis and Nasal Polyps 2012 guidelines^1^. Details of subjects’ characteristics are included in Supplementary Table S7 and Supplementary Methods online. None of the subjects used systemic or topical corticosteroids or other immune-modulating drugs for at least 4 weeks before surgery. Nasal polyp tissues from the apex region of polyps were collected during functional endoscopic sinus surgery. Subjects who had an immune deficient disease, antrochoanal polyps, fungal sinusitis, cystic fibrosis, primary ciliary dyskinesia, or gastroesophageal reflux disease were excluded from the study.

The CRSwNP patients were classified based on the tissue eosinophil number, counted in the lamina propria of the polyps in five random microscopic high power fields (HPFs, ×400 magnification). We defined ECRSwNP as subjects whose tissue eosinophil number per HPF was more than twenty^4^, and non-ECRSwNP as not fulfilling the criteria.

### Whole transcriptome library preparation and sequencing

Total RNAs from 3 ECRSwNP, 3 non-ECRSwNP and 3 control subjects were isolated and quality controlled. The preparation of whole transcriptome libraries and deep sequencing were performed by Novogene Bioinformatics Technology Cooperation (Beijing, China). Ribosomal RNA (rRNA) was removed and strand-specific sequencing libraries were generated following manufacture’s recommendations. RNA-Seq was performed on an Illumina Hiseq 2000 platform and 100 bp paired-end reads were generated according to Illumina’s protocol. Detailed procedures are in the Supplementary Information. The NCBI Gene Expression Omnibus accession number for the RNA-Seq data reported in this paper is GSE72713.

### RNA-Seq data analysis

The adapter sequences were removed from the raw sequencing data and the individual libraries were converted to the FASTQ format. Sequence reads were aligned to the human genome (hg19) with TopHat2 and the resulting alignment files were reconstructed with Cufflinks^50^ and Scripture^51^. For mRNA analyses, the RefSeq database (Build 37.3) was chosen as the annotation references. For lncRNA analyses, the GENCODE v19 database was chosen as the annotation references. The low-confidence transcripts were filtered by the computational pipeline. The read counts of each transcript were normalized to the length of the individual transcript and to the total mapped fragment counts in each sample and expressed as fragments per kilo-base of exon per million fragments mapped (FPKM) of both lncRNAs and mRNAs in each sample. The mRNA and lncRNA differential expression analyses for all pairwise comparisons: ECRSwNP versus CTRL, non-ECRSwNP versus CRTL, and ECRSwNP versus non-ECRSwNP using Cuffdiff. An adjusted *P* value < 0.05 (Student’s *t*-test with Benjamini-Hochberg false discovery rate (FDR) adjustment) was used as the cut-off for significantly differentially expressed genes. Differentially expressed genes were analyzed by enrichment analyses to detect over-represented functional terms present in the genomic background. See the Supplementary Information for details on the bioinformatics analyses.

### Prediction of the function of lncRNAs

Most of the lncRNAs in current databases have not yet been functionally annotated. Thus, the prediction of their functions is based on the functional annotations of their related *cis* and *trans* target mRNAs. We defined potentially *cis*-regulated target genes as protein-coding genes within 100kb in genomic distance from the lncRNA, and potentially *trans*-regulated target genes as protein-coding genes coexpressed with the lncRNA with Pearson correlation coefficient (PCC) >0.95 or <−0.95 and beyond 100 kb in genomic distance from the lncRNA or in different chromosomes. We analyzed the expression correlation of lncRNA:*cis*-mRNA pairs and lncRNA:*trans*-mRNA pairs by calculating PCC.

### Quantitative Real-time PCR

All primers qRT-PCR for qRT-PCR experiments are listed in Supplementary Table S8. Detailed procedures are in the Supplementary Information.

### *In situ* hybridization

*In situ* hybridization analyses for lncRNA XLOC_010280 were performed on paraffin-embedded sections by using digoxigenin (DIG)-labeled XLOC_010280 antisense probe or sense control probe. More information is provided in the Method section in Supplementary Materials.

## Additional Information

**Accession codes:** Data for the RNA-Seq experiments are deposited in the NCBI Gene Expression Omnibus as GSE72713.

**How to cite this article**: Wang, W. *et al*. Transcriptome Analysis Reveals Distinct Gene Expression Profiles in Eosinophilic and Noneosinophilic Chronic Rhinosinusitis with Nasal Polyps. *Sci. Rep*. **6**, 26604; doi: 10.1038/srep26604 (2016).

## Supplementary Material

Supplementary Information

## Figures and Tables

**Table 1:** The mRNAs with over 16-folds difference between ECRSwNP and non-ECRSwNP.

Transcript ID	Gene name	Fold Change	Adjusted P value
NM_001898.2	CST1	1732.2	1.70E-03
NM_182519.2	C20orf186	289.7	1.70E-03
NM_006183.4	NTS	72.3	1.70E-03
NM_002988.2	CCL18	71.3	1.70E-03
NM_005408.2	CCL13	63.3	1.70E-03
NM_006072.4	CCL26	62.3	1.70E-03
NM_012387.2	PADI4	57.7	2.78E-02
NM_001657.2	AREG	57.2	1.59E-02
NM_153264.5	COL6A5	56.6	1.70E-03
NM_001828.4	CLC	44.9	1.70E-03
3497	IGHE	34.5	1.70E-03
NM_001102469.1	LIPN	31.2	8.70E-03
NM_001432.2	EREG	30.6	1.70E-03
NM_014375.2	FETUB	30.2	8.70E-03
NM_020530.4	OSM	29.2	1.70E-03
NM_005064.3	CCL23	25.8	3.91E-02
NM_173157.2	NR4A1	24.0	1.70E-03
NM_032571.3	EMR3	23.0	1.70E-03
NM_002188.2	IL13	22.0	1.70E-03
NM_005304.3	FFAR3	20.6	1.70E-03
NM_002991.2	CCL24	20.5	1.70E-03
NM_000600.3	IL6	19.9	1.70E-03
NM_178329.2	CCR3	19.8	1.70E-03
XM_003403441.1	RAB44	19.2	1.70E-03
NM_006981.3	NR4A3	18.8	1.70E-03
NM_138983.2	OLIG1	18.0	2.15E-02
NM_001805.2	CEBPE	17.6	1.70E-03
NM_152891.2	PRSS33	16.7	1.70E-03
NM_007015.2	LECT1	16.3	6.66E-03
NM_004778.2	GPR44	16.0	7.72E-03
NM_199161.3	SAA1	−16.0	1.70E-03
NM_001127380.2	SAA2	−16.8	1.70E-03
NM_002994.3	CXCL5	−17.4	1.70E-03
NM_033049.3	MUC13	−17.6	1.70E-03
NM_014479.3	ADAMDEC1	−18.2	1.70E-03
NM_005409.4	CXCL11	−18.6	1.70E-03
NM_030754.4	SAA2	−25.0	1.70E-03
NM_002416.1	CXCL9	−25.3	1.70E-03
NM_001565.3	CXCL10	−28.8	1.70E-03
NM_001063.3	TF	−30.9	1.70E-03
NM_002127.5	HLA-G	−33.1	2.07E-02
NM_001927.3	DES	−47.5	1.70E-03
NM_178565.4	RSPO2	−50.4	4.61E-02
