# Supplementary material for: Transcriptome Analysis Reveals Distinct Gene Expression Profiles in Eosinophilic and Noneosinophilic Chronic Rhinosinusitis with Nasal Polyps
Source: Sci Rep. 2016 May 24;6:26604. doi: 10.1038/srep26604 (PMC4877582; doi:10.1038/srep26604)
Supplement: Supplementary Information [file srep26604-s1.pdf]

Transcriptome Analysis Reveals Distinct Gene Expression Profiles in Eosinophilic and Noneosinophilic Chronic Rhinosinusitis with Nasal Polyps

Weiqing Wang[1], Zhiqiang Gao[1], Huaishan Wang[2], Taisheng Li[3], Wei He[2], Wei Lv[1]*, and Jianmin Zhang[2]*

[1]Department of Otolaryngology, Peking Union Medical College Hospital, Chinese Academy of Medical Science and Peking Union Medical College, Beijing, China

[2]Department of Immunology, Institute of Basic Medical Sciences, Chinese Academy of Medical Sciences and School of Basic Medicine, Peking Union Medical College, State Key Laboratory of Medical Molecular Biology, Beijing, China

[3]Department of Infectious Disease, Peking Union Medical College Hospital, Chinese Academy of Medical Science and Peking Union Medical College, Beijing, China

* Address correspondence to Dr. Jianmin Zhang,

Department of Immunology, Institute of Basic Medical Sciences, Chinese Academy of Medical Sciences and School of Basic Medicine, Peking Union Medical College, State Key Laboratory of Medical Molecular Biology,

5 Dongdansantiao Street, Beijing 100730, China.

Tel.: +86 10 69156474, E-mail: jzhang42@gmail.com

or to Dr. Wei Lv,

Department of Otolaryngology, Peking Union Medical College Hospital, Chinese Academy of Medical Science and Peking Union Medical College,

No.1 Shuaifuyuan, Beijing 100730, China.

Tel.: +86 10 69156311, E-mail: lili20020615@sina.com

Supplementary Methods

Patients and biopsy specimens

The study was approved by the Ethics Committee of Peking Union Medical College Hospital. All study participants provided written informed consent and were recruited at Peking Union Medical College Hospital. All experiments were performed in accordance with approved guidelines. A total of 35 patients with ECRSwNP, 31 patients with non-ECRSwNP and 34 control subjects were recruited for the study. The diagnosis of CRSwNP was based on standard criteria issued in the European Position Paper on Rhinosinusitis and Nasal Polyps 2012 guidelines(1). None of the subjects used systemic or topical corticosteroids or other immune-modulating drugs for at least 4 weeks before surgery.

All CRSwNP patients were Chinese with bilateral CRSwNP. The presence of CRS and bilateral NPs was confirmed by means of office endoscopy and computed tomographic imaging. They failed to respond to maximal medical therapy and subsequently underwent functional endoscopic sinus surgery. Nasal polyp tissues from the apex region of polyps were collected during functional endoscopic sinus surgery. Control subjects were patients undergoing endoscopic trans-sphenoid removal of nonfunctioning pituitary adenomas not having inflammatory sinonasal diseases. The sphenoid mucosal tissues of control subjects were collected during the surgery.

The atopic status was evaluated by the Phadiatop test (Phadia, Uppsala, Sweden) to detect IgE antibodies against various common inhalant allergens. Specific IgE values of 0.35 kIU/L or greater were considered positive. The diagnosis of asthma was based on history and pneumologist diagnosis. Subjects who had an immune deficient

disease, antrochoanal polyps, fungal sinusitis, cystic fibrosis, primary ciliary dyskinesia, or gastroesophageal reflux disease were excluded from the study.

The CRSwNP patients were classified based on the tissue eosinophil number, counted in the lamina propria of the polyps in five random microscopic high power fields (HPFs, ×400 magnification). We defined ECRSwNP as subjects whose tissue eosinophil number per HPF was more than twenty(2), and non-ECRSwNP as not fulfilling the criteria.

Sampling of tissue specimens and histological analysis

The tissues were divided into three parts immediately after harvest from the surgery. One part was fixed in 10% formalin, and later embedded in paraffin for hematoxylin and eosin (HE) staining. The other two parts were immediately snap-frozen in liquid nitrogen and stored at -80°C for whole transcriptome sequencing and quantitative Real-time PCR (qRT-PCR) experiments. Paraffin sections (4μm) were stained with HE dye. In order to determine the degree of eosinophil infiltration, two of the authors independently detected the number of eosinophils per HPF by manually counting five random HPF of lamina propria per specimen in the HE sections. We used a Leica DM3000 light microscope with an N Plan 40×/0.65 lens and an FN 22 eyepiece; this ‘HPF’ includes an area of $0.24mm^2$ on the slide. In cases of disagreement (when 2 average numbers differed by >10%), a consensus was reached by our research team reviewing the specimen together.

Whole transcriptome library preparation and sequencing

Total RNA was isolated from 3 ECRSwNP, 3 non-ECRSwNP and 3 control subjects using Trizol (Invitrogen) according to the manufacturer’s instructions. RNA purity was checked using the NanoPhotometer spectrophotometer (IMPLEN, CA, USA). RNA

concentration was measured using Qubit RNA Assay Kit in Qubit 2.0 Flurometer (Life Technologies, CA, USA). RNA integrity was verified on an Agilent Bioanalyzer 2100 (Agilent Technologies, Palo Alto, CA). Only samples with RNA integrity number (RIN) over 7.0 were used for library construction. A total amount of 3 μg RNA per sample was used as input material for library construction. Ribosomal RNA (rRNA) was removed by Epicentre Ribo-zero rRNA Removal Kit (Epicentre, USA) according to the manufacturer's instructions. Subsequently, strand-specific sequencing libraries were generated with the dUTP method using the resulting RNA by NEBNext Ultra Directional RNA Library Prep Kit for Illumina (NEB, USA) following manufacturer's recommendations. The library quality and quantity was assessed using the Agilent Bioanalyzer 2100 system and qRT-PCR. RNA-seq was performed on an Illumina Hiseq 2000 platform and 100 bp paired-end reads were generated according to Illumina's protocol. The NCBI Gene Expression Omnibus accession number for the RNA-Seq data reported in this paper is GSE72713.

RNA-seq data analysis

(1) Quality control: The raw sequencing data (raw reads) were stored in the FASTQ format. Clean data (clean reads) were obtained by removing reads containing adapter, reads containing N (N means not knowing the base information) for more than 10% of all the bases and low quality reads marked by sequencing platform from raw data. All the down stream analyses were based on the clean data with high quality.

(2) Reads mapping and transcriptome assembly: Clean reads were aligned to the human genome (hg19) using TopHat2 (v2.0.9). The transcriptome of each sample was assembled from the mapped reads separately by both Cufflinks (v2.1.1) (3) and Scripture (beta2) (4).

(3) Transcripts identification pipeline: For mRNA analyses, the RefSeq database (Build 37.3) was chosen as the annotation references. For lncRNA analyses, the GENCODE v19 database was chosen as the annotation references. To identify lncRNA transcripts from the assembled transcripts set, we ran the set through the following filters:

(a) Expression level threshold. The transcripts were reconstructed in at least two different tissues or by both Scripture and Cufflinks in the same tissue with minimal read coverage $\geqslant$ 3. This step aimed to distinguish the lowly expressed lncRNAs from the various lowly expressed, unreliable fragments assembled from the RNA-seq data.

(b) Size selection. The transcripts were multiexonic and not smaller than 200 bases. This step aimed to remove transcripts which were not consistent with the definition of lncRNA, or single-exonic, which might result from potential DNA contamination.

(c) Filter of known non-lncRNA annotations. We eliminated all transcripts that overlapped a transcript which included all known protein-coding genes, microRNAs, tRNAs, miscRNA, rRNAs and pseudogenes and retained all transcripts previously annotated as lncRNAs (GENCODE v19) or that have not been annotated thus far.

(d) Positive coding potential threshold. We scored the coding potential of novel transcripts using phylogenetic codon substitution frequency (PhyloCSF) (5). All transcripts with PhyloCSF scores >100 were discarded.

(e) Known protein domain filter. To eliminate protein-coding transcripts thoroughly, we translated every transcript in all three possible reading frames and employed Pfam Scan (v1.3) to detect any of the known protein domains cataloged in the Pfam database (release 27; used both Pfam A and Pfam B) (6). We excluded all transcripts

with a Pfam hit. Finally, the remaining transcripts were considered reliably expressed lncRNAs.

(4) Differential Expression Analysis

The read counts of each transcript were normalized to the length of the individual transcript and to the total mapped fragment counts in each sample and expressed as fragments per kilo-base of exon per million fragments mapped (FPKM) of both lncRNAs and mRNAs in each sample. The mRNA and lncRNA differential expression analyses for all pairwise comparisons: ECRSwNP versus CTRL, non-ECRSwNP versus CRTL, and ECRSwNP versus non-ECRSwNP using Cuffdiff (v2.1.1) (3). An adjusted *P* value <0.05 (Student's t-test with Benjamini-Hochberg false discovery rate (FDR) adjustment) was used as the cut-off for significantly differentially expressed genes.

(5) GO and KEGG enrichment analysis

Differentially expressed genes were analyzed by enrichment analyses to detect over-represented functional terms present in the genomic background. Gene ontology (GO) analysis was performed using the GOseq R package(7), in which gene length bias was corrected. Kyoto Encyclopedia of Genes and Genomes (KEGG) pathway analysis were performed using KOBAS software(8).

Prediction of the function of lncRNAs

Most of the lncRNAs in current databases have not yet been functionally annotated. Thus the prediction of their functions is based on the functional annotations of their related *cis* and *trans* target mRNAs. We defined potentially cis-regulated target genes as protein-coding genes within 100kb in genomic distance from the lncRNA, and potentially trans-regulated target genes as protein-coding genes coexpressed with the

lncRNA with Pearson correlation coefficient (PCC) >0.95 or <-0.95 and beyond 100kb in genomic distance from the lncRNA or in different chromosomes. We analyzed the expression correlation of lncRNA:*cis*-mRNA pairs and lncRNA:*trans*-mRNA pairs by calculating PCC.

Quantitative Real-time PCR

Total RNA was isolated using Trizol (Invitrogen) following the manufacturer's instruction. The cDNA was synthesized using Superscript III (Invitrogen). All qRT-PCR primers (Table S8) were verified to produce specific PCR product and to react efficiently. All qRT-PCR reactions were performed on a Roche Lightcycler480 real-time PCR system using TOYOBO Thunderbird SYBR qRT-PCR Mix (TOYOBO, OSAKA JAPAN) with technical triplicates. Relative quantification of target genes was performed using the $2^{-\Delta\Delta Ct}$ method with *GAPDH* as a reference gene.

***In situ* hybridization**

In situ hybridization analyses for lncRNA XLOC_010280 were performed on paraffin-embedded sections. Briefly, paraffin embedded sections (6μm) were deparaffinized and rehydrated. Then, tissue sections were hybridized with digoxigenin (DIG)-labeled XLOC_010280 antisense probe (5'–GTTTAACACAGCGCCCTAGTCACTACATGACTAATAAATAGACAAATGACTGAAACATGACCTCATGCTTTCTATTCCTCCAGCTTTCATTCAGTTCTTTGCCTCTGGGAGGAGGAAGGGTTGTGCAGCCCTCCACAGCATCAGCCCATCAACCCTATCCCTGTGGTTATAGCAGCTGAGGAAGCAGAATTGCAGCTCT-3', Biosense Biotechnology, Guangzhou, China) or sense control probe at 42°C overnight. Slides were then washed and incubated with Biotinylated Mouse anti-DIG antibody (Boster Biotechnology, Wuhan, China) for 30 min at 37°C. Color development was achieved

with 3,3'-diaminobenzidine (DAB) (Boster Biotechnology).

References

1. Fokkens WJ, Lund VJ, Mullol J, Bachert C, Alobid I, Baroody F, et al. European Position Paper on Rhinosinusitis and Nasal Polyps 2012. *Rhinol Suppl* 2012(23):3 p preceding table of contents, 1-298.

2. Wang ET, Zheng Y, Liu PF, Guo LJ. Eosinophilic chronic rhinosinusitis in East Asians. *World J Clin Cases* 2014;**2**(12):873-882.

3. Trapnell C, Williams BA, Pertea G, Mortazavi A, Kwan G, van Baren MJ, et al. Transcript assembly and quantification by RNA-Seq reveals unannotated transcripts and isoform switching during cell differentiation. *Nat Biotechnol* 2010;**28**(5):511-515.

4. Guttman M, Garber M, Levin JZ, Donaghey J, Robinson J, Adiconis X, et al. Ab initio reconstruction of cell type-specific transcriptomes in mouse reveals the conserved multi-exonic structure of lincRNAs. *Nat Biotechnol* 2010;**28**(5):503-510.

5. Lin MF, Jungreis I, Kellis M. PhyloCSF: a comparative genomics method to distinguish protein coding and non-coding regions. *Bioinformatics* 2011;**27**(13):i275-282.

6. Punta M, Coggill PC, Eberhardt RY, Mistry J, Tate J, Boursnell C, et al. The Pfam protein families database. *Nucleic Acids Res* 2012;**40**(Database issue):D290-301.

7. Young MD, Wakefield MJ, Smyth GK, Oshlack A. Gene ontology analysis for RNA-seq: accounting for selection bias. *Genome Biol* 2010;**11**(2):R14.

8. Mao X, Cai T, Olyarchuk JG, Wei L. Automated genome annotation and pathway identification using the KEGG Orthology (KO) as a controlled vocabulary. *Bioinformatics* 2005;**21**(19):3787-3793.

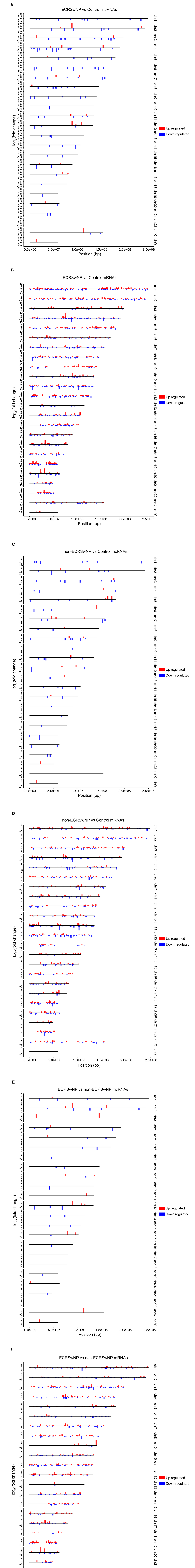

Figure S1. Chromosomal locations of differentially expressed lncRNAs and mRNAs. (A-B) Differentially expressed lncRNAs (A) and mRNAs (B) between ECRSwNP and Control group. (C-D) Differentially expressed lncRNAs (C) and mRNAs (D) between non-ECRSwNP and Control group. (E-F) Differentially expressed lncRNAs (E) and mRNAs (F) between ECRSwNP and non-ECRSwNP group. The y-axis represents their log2 fold change and x-axis represents their chromosomal locations.

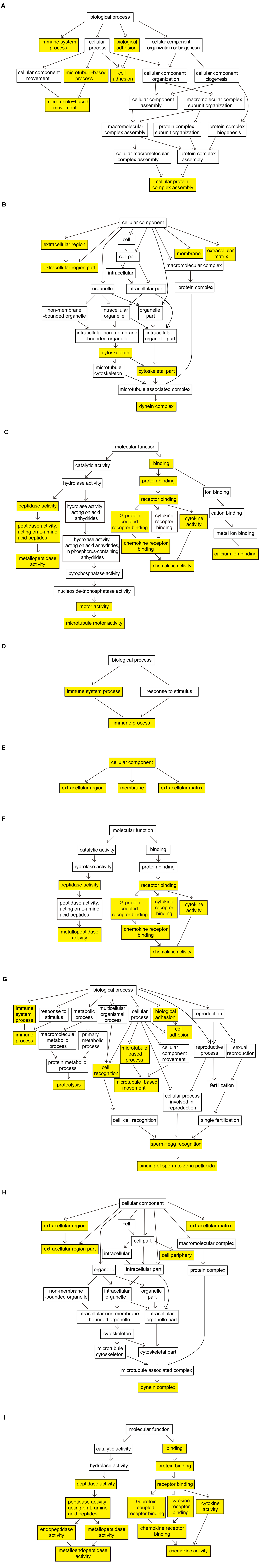

Figure S2. The GO analysis of differentially expressed protein-coding genes. In directed acyclic graph (DAG) the downstream term is a subset of the upstream term. The statistically over-represented GO terms are marked in yellow. (A-C) The DAG of over-represented biological process (A), cellular component (B), and molecular function (C) terms in GO analysis between ECRSwNP and Control group. (D-F) The DAG of over-represented biological process (D), cellular component (E), and molecular function (F) terms in GO analysis between non-ECRSwNP and Control group. (G-I) The DAG of over-represented biological process (G), cellular component (H), and molecular function (I) terms in GO analysis between ECRSwNP and non-ECRSwNP group.

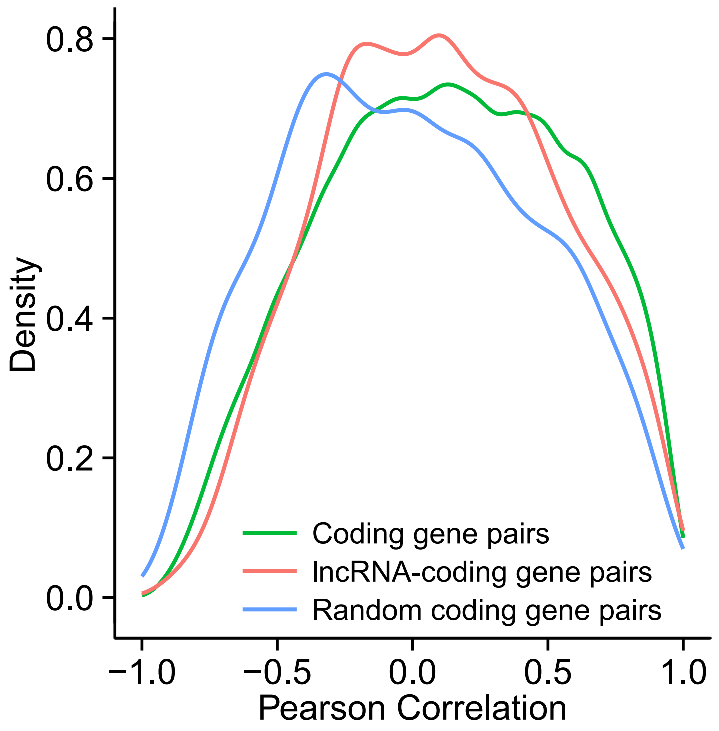

Figure S3. Correlation of expression patterns between pairs of neighboring genes. Density distributions of Pearson correlation coefficients in expression levels are analyzed among coding gene neighbors (green), lncRNAs and their neighboring coding gene (red), and random pairs of protein-coding genes (blue).

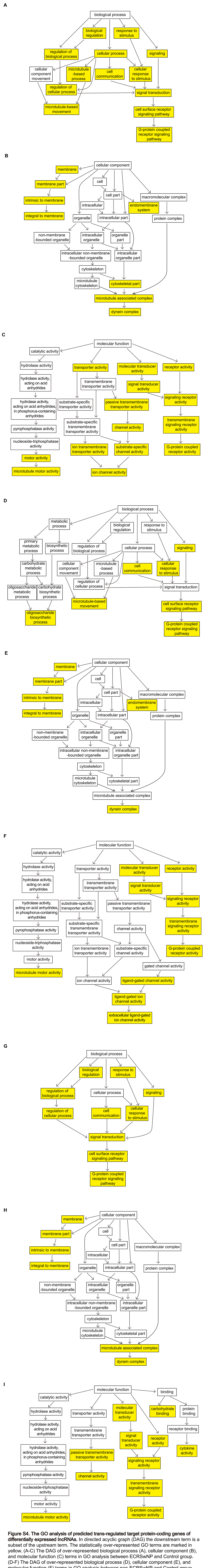

Figure S4. The GO analysis of predicted trans-regulated target protein-coding genes of differentially expressed lncRNAs. In directed acyclic graph (DAG) the downstream term is a subset of the upstream term. The statistically over-represented GO terms are marked in yellow. (A-C) The DAG of over-represented biological process (A), cellular component (B), and molecular function (C) terms in GO analysis between ECRSwNP and Control group. (D-F) The DAG of over-represented biological process (D), cellular component (E), and molecular function (F) terms in GO analysis between non-ECRSwNP and Control group. (G-I) The DAG of over-represented biological process (G), cellular component (H), and molecular function (I) terms in GO analysis between ECRSwNP and non-ECRSwNP group.

Table S1. The data yields and mapping conditions of each sample in RNA-seq.

Sample name	Ctrl_1	Ctrl_2	Ctrl_3	ECRSwNP_1	ECRSwNP_2	ECRSwNP_3	non-ECRSwNP_1	non-ECRSwNP_2	non-ECRSwNP_3
Raw reads	114468362	110800134	108048076	114186598	115703956	106189180	109985166	109714356	128170192
Clean reads	106130072	106252622	103355394	109364286	110898200	101838586	104328114	104064708	122760360
Total mapped*	97557166 (91.92%)	97899889 (92.14%)	97623477 (94.45%)	102669396 (93.88%)	103785494 (93.59%)	95567031 (93.84%)	95245291 (91.29%)	95307506 (91.58%)	113887310 (92.77%)
Multiple mapped*	2845670 (2.68%)	3144533 (2.96%)	2276128 (2.2%)	2848626 (2.6%)	2214910 (2%)	2460852 (2.42%)	2402037 (2.3%)	2818124 (2.71%)	3440474 (2.8%)
Uniquely mapped*	94711496 (89.24%)	94755356 (89.18%)	95347349 (92.25%)	99820770 (91.27%)	101570584 (91.59%)	93106179 (91.43%)	92843254 (88.99%)	92489382 (88.88%)	110446836 (89.97%)
Reads map to '+' strand*	47351657 (44.62%)	47356733 (44.57%)	47659081 (46.11%)	49929217 (45.65%)	50699722 (45.72%)	46544789 (45.7%)	46393912 (44.47%)	46268483 (44.46%)	55258137 (45.01%)
Reads map to '-' strand*	47359839 (44.62%)	47398623 (44.61%)	47688268 (46.14%)	49891553 (45.62%)	50870862 (45.87%)	46561390 (45.72%)	46449342 (44.52%)	46220899 (44.42%)	55188699 (44.96%)
Non-splice reads*	88808686 (83.68%)	85058090 (80.05%)	85555747 (82.78%)	87935155 (80.41%)	89924832 (81.09%)	82985611 (81.49%)	79929736 (76.61%)	83723684 (80.45%)	98363786 (80.13%)
Splice reads*	5902810 (5.56%)	9697266 (9.13%)	9791602 (9.47%)	11885615 (10.87%)	11645752 (10.5%)	10120568 (9.94%)	12913518 (12.38%)	8765698 (8.42%)	12083050 (9.84%)

*Numbers in the brackets stand for the percentages of clean reads.

Table S2. Characteristics of all of the lncRNAs identified in this study.

Transcript ID	Gene ID	Gene Name	Transcript Type	Transcript Length	Chromosome	Transcript Start	Transcript End	Strand	Exon Number	Ctrl_1	Ctrl_2	Ctrl_3	ECRSwNP _1	ECRSwNP _2	ECRSwNP _3	non-ECRSwNP _1	non-ECRSwNP _2	non-ECRSwNP _3
ENST00000428504.1	ENSG00000228327.2	RP11-206L10.2	lincRNA	1317	chr1	700237	714006	-	7	10.6818	11.6694	8.8324	12.9021	8.60499	15.3168	6.87741	10.694	10.8262
TCONS_00005701	XLOC_000081	-	lincRNA	731	chr1	1210155	1214753	+	3	0.95824	0.93894	1.32932	0.966796	0.466762	1.32935	0.335345	1.07395	0.766975
TCONS_00013785	XLOC_000122	-	intronic_lncRNA	307	chr1	3466292	3467059	+	2	0	0	0	1.8079	0	0.666249	1.19688	0.805181	1.12074
TCONS_00018121	XLOC_001455	-	lincRNA	456	chr1	4378028	4379740	-	2	0.39578	0.84389	0.28073	0.503226	1.71391	0.277175	1.66388	0.973062	0.22984
TCONS_00018124	XLOC_001465	-	lincRNA	368	chr1	6260705	6266075	-	2	0	0	0	0	0.754141	0.424225	0	0.50315	1.41554
ENST00000412639.2	ENSG00000234546.2	RP3-510D11.2	lincRNA	985	chr1	9242263	9252148	+	3	1.55766	0.99473	2.4147	0.408264	1.57318	1.07735	1.95127	1.75411	1.4034
TCONS_00006803	XLOC_001488	-	intronic_lncRNA	1354	chr1	9740074	9747626	-	2	0.53026	0.79791	0.90926	0.999019	1.44134	0.915099	1.11756	1.18866	0.701834
TCONS_00012664	XLOC_001501	-	lincRNA	1925	chr1	11371578	11376966	-	3	1.53812	1.55787	1.86068	0.630859	1.88967	0.937987	0.440246	0.373204	0.368318
TCONS_00000463	XLOC_000192	-	lincRNA	4312	chr1	14153723	14172101	+	2	3.84944	1.2875	1.75953	0.653422	1.43313	1.79479	0.990412	1.01445	1.05152
TCONS_00005779	XLOC_000192	-	lincRNA	16777	chr1	14219595	14523049	+	2	10.4124	5.04458	4.42439	1.49517	3.70003	3.81532	1.89756	2.56912	1.59587
TCONS_00023764	XLOC_000192	-	lincRNA	17064	chr1	14219863	14559392	+	3	16.9833	6.93797	7.25314	1.86359	5.19906	5.49792	2.95395	3.74289	2.41673
TCONS_00008846	XLOC_001516	-	intronic_lncRNA	887	chr1	15414805	15416980	-	2	1.63091	3.30986	2.5589	0.746659	0.809754	0.615807	0.343783	0.826729	0.422427
TCONS_00001970	XLOC_001528	-	lincRNA	757	chr1	16542415	16543688	-	2	0.36547	1.02249	0.63314	0.460757	1.22306	0.886907	1.27738	0.730688	1.14842
TCONS_00003383	XLOC_000243	-	lincRNA	4161	chr1	20523818	20530457	+	3	4.38283	3.06538	3.81159	0.597588	0.852411	1.36639	3.96624	3.36217	3.63886
TCONS_00012726	XLOC_001573	-	intronic_lncRNA	3939	chr1	22463123	22467367	-	2	2.45801	1.756	2.28906	0.239546	2.16744	0.50759	0.295731	0.49357	0.431786
TCONS_00025889	XLOC_001596	-	lincRNA	496	chr1	24822766	24828953	-	3	3.08406	0.48516	0	0	0.844525	1.19599	2.04254	1.11659	1.31029
TCONS_00006901	XLOC_001596	-	lincRNA	372	chr1	24822865	24826292	-	2	0.0004	6.68E-05	1.69806	0.751138	0.368066	0.0017709	6.28E-05	0	1.86164
TCONS_00005879	XLOC_000333	-	lincRNA	421	chr1	31199514	31201046	+	2	0.45781	1.30756	0.65387	0	0.569171	0.321549	0.277393	0	0.801385
TCONS_00010014	XLOC_000388	-	lincRNA	289	chr1	38568097	38569434	+	2	1.10352	0	2.51415	0	0.71713	0.796597	0.723977	0	0.67234
TCONS_00010015	XLOC_000391	-	lincRNA	771	chr1	39508189	39513847	+	3	2.49581	0.99718	1.48169	1.34827	2.16868	1.60654	0.518912	0.427485	0.407268
TCONS_00020825	XLOC_000412	-	lincRNA	771	chr1	41898873	41930804	+	3	0.01387	0.12462	0.12345	0.906128	0.650569	1.93062	2.20235	0.56998	0.522392
TCONS_00013963	XLOC_000412	-	lincRNA	888	chr1	41898883	41931007	+	2	0.87677	2.65E-05	2.45E-05	2.97624	2.99E-05	1.67845	2.89888	0	0.495145
TCONS_00009052	XLOC_001763	-	lincRNA	1148	chr1	45253604	45258474	-	3	1.29044	1.8698	2.66085	0.946195	0.130105	0.668698	0.371873	1.53478	0.916327
TCONS_00010064	XLOC_000455	-	lincRNA	812	chr1	46598952	46600903	+	2	1.83007	0.69734	0.57541	0.314416	0.707713	0.576329	1.35366	0.796808	0.379735
TCONS_00019022	XLOC_000467	-	lincRNA	2384	chr1	47375299	47377874	+	2	2.89773	1.6503	1.91485	0.527464	4.84032	3.7997	0.747833	1.58258	1.55928
TCONS_00010075	XLOC_000477	-	lincRNA	2318	chr1	50889646	50892499	+	3	0.6265	0.43381	0.95447	0.060435	1.21887	0.365261	0.826361	0.455473	0.354305
TCONS_00000816	XLOC_000492	-	lincRNA	362	chr1	53132934	53134283	+	2	2.48732	0.89861	0.90387	0.797778	0.391423	0	1.1565	0.522685	1.10203
ENST00000449958.1	ENSG00000230138.1	RP11-117D22.2	lincRNA	850	chr1	53832340	53833917	-	2	0.15668	0.32811	0.43305	0.295982	0.190279	0.325501	0.545583	0.499699	1.07203
TCONS_00011012	XLOC_001829	-	lincRNA	643	chr1	54878636	54879380	-	2	0.91813	0.48333	0.63949	1.15967	0	0.318997	0.269215	0.738207	0.263217
TCONS_00002339	XLOC_001830	-	lincRNA	563	chr1	54982451	54994454	-	3	2.23858	0.39431	3.13559	0.177071	2.74483	0.779611	0.826428	1.81032	1.44955
TCONS_00007092	XLOC_001849	-	lincRNA	869	chr1	58797260	58801922	-	2	1.37017	1.27487	1.36691	0.191701	1.38621	1.05407	2.03092	0.970613	1.04133
TCONS_00000840	XLOC_000521	-	lincRNA	609	chr1	59180299	59181451	+	2	1.9121	0.52405	1.11128	0.203459	1.10086	1.2099	1.1764	1.32636	0.894721
TCONS_00000841	XLOC_000521	-	lincRNA	849	chr1	59180517	59184024	+	3	0.83259	0.87632	0.28118	0.464877	0.739135	0	0.997246	1.17318	1.04954
TCONS_00000842	XLOC_000524	-	lincRNA	4447	chr1	59276786	59281987	+	2	3.27026	1.51244	2.30953	0.72302	1.66213	1.48946	1.15061	1.62429	1.6153
TCONS_00010095	XLOC_000525	-	lincRNA	2053	chr1	59486093	59509603	+	3	0.88114	0.30498	1.20412	0.119203	0.366568	0.25946	0.313906	1.21901	2.08796
TCONS_00014008	XLOC_000525	-	lincRNA	432	chr1	59486116	59487411	+	2	0.00069	0.00219	0.0035	0.430417	0.799699	0.356692	1.06429	1.76739	0
TCONS_00002363	XLOC_001856	-	lincRNA	3621	chr1	60427121	60434026	-	2	2.47741	0.49535	0.67137	0.187018	0.376926	0.102742	0.170167	0.422277	0.724839
TCONS_00010101	XLOC_000534	-	lincRNA	711	chr1	62102650	62114107	+	2	0.99558	1.95218	1.79693	0.125597	0.242618	0	0.581312	0.957222	0.455613
TCONS_00023934	XLOC_000540	-	lincRNA	396	chr1	63705311	63708496	+	2	0.51547	0	0	0.329282	0	0	1.25958	0	0
ENST00000448344.1	ENSG00000231485.1	RP4-535B20.1	lincRNA	310	chr1	65532310	65533420	-	2	3.62	4.66	6.08446	1.17307	3.48642	0	1.16268	3.13037	3.27019
TCONS_00014028	XLOC_000555	-	lincRNA	247	chr1	66169966	66185518	+	2	0	1.43774	0	1.25249	0	0	0	0	0
TCONS_00014038	XLOC_000563	-	lincRNA	1150	chr1	68027734	68041795	+	2	0.21462	0.4478	0.07376	0.202328	0.064915	0	0.865854	0.255253	0.548632
TCONS_00026900	XLOC_000573	-	lincRNA	667	chr1	71225848	71255026	+	3	0	0	0.00126	0	0.003338	0	0	0.359423	2.97E-05
TCONS_00010142	XLOC_000573	-	lincRNA	1126	chr1	71226041	71251883	+	2	0.33026	0.53606	0.45348	1.2455	0.79762	0.15214	1.26942	2.00334	0.312728
ENST00000455406.1	ENSG00000229956.5	ZRANB2-AS2	lincRNA	783	chr1	71547007	71703406	+	4	2.79385	0.61029	2.29699	1.87061	1.48652	3.38871	2.43843	3.34835	1.49552
TCONS_00000917	XLOC_000579	-	lincRNA	2532	chr1	72765667	72813714	+	2	2.93287	0.84841	1.79287	0.548846	0.948637	0.904649	1.07519	0.792558	0.618681
TCONS_00016660	XLOC_001901	-	anti-sense_lncRNA	4447	chr1	78408146	78413468	-	2	13.4898	9.39045	16.265	12.0202	11.1724	15.4407	10.1363	15.2041	14.2391
ENST00000443565.1	ENSG00000234184.1	RP5-887A10.1	lincRNA	473	chr1	81106969	81112473	+	3	0.17707	0.79032	1.53027	4.15631	0.917144	0.836789	2.86167	3.53	6.25104
TCONS_00010162	XLOC_000608	-	lincRNA	716	chr1	81106974	81112850	+	2	0.89195	0	0.43448	0.544356	0	0.653802	0.589746	0.689971	1.57427
TCONS_00002427	XLOC_001907	-	lincRNA	299	chr1	82529650	82531100	-	2	1.9994	0	0.7529	0	0	0	0	0	0
TCONS_00011063	XLOC_001910	-	lincRNA	12819	chr1	84203468	84326689	-	3	0.29829	0.00098	0.07202	0.283981	0.0001847	0.0001974	0.0004665	1.88235	0.0001444
TCONS_00002428	XLOC_001910	-	lincRNA	13743	chr1	84203468	84316817	-	2	4.72604	1.8206	1.90473	0.804754	1.90286	2.89124	1.55709	2.3411	1.1986
TCONS_00018393	XLOC_001910	-	lincRNA	12526	chr1	84203513	84326708	-	2	0.00974	1.18197	1.48367	0.44624	1.08343	1.17781	0.464955	0.952724	1.44112
TCONS_00020088	XLOC_001910	-	lincRNA	3772	chr1	84312358	84326925	-	2	5.78768	3.21464	4.79904	1.58585	3.30026	4.23102	3.06344	5.61568	3.46659
TCONS_00021564	XLOC_001918	-	lincRNA	414	chr1	85184523	85215276	-	2	0.94523	0.67567	1.01415	0.301387	1.47064	0.33218	0	1.56292	0
TCONS_00007152	XLOC_001928	-	lincRNA	3191	chr1	86730987	86781126	-	2	1.77509	1.25145	0.83801	0.213922	0.49282	0.775672	1.0126	1.10056	1.21493

TCONS_00021576	XLOC_001928	-	lincRNA	3961	chr1	86781788	86786008	-	2	2.93237	1.82079	1.09157	0.374227	0.81619	1.27089	1.2843	1.10935	1.22645
TCONS_00002457	XLOC_001928	-	lincRNA	2829	chr1	86792150	86795285	-	2	4.15513	2.28398	2.2255	0.608498	0.981444	1.6047	2.41617	2.04701	1.93121
TCONS_00014816	XLOC_001937	-	lincRNA	615	chr1	88430507	88452652	-	2	0.97976	0.34425	1.53802	1.70261	2.84543	0.510983	0.7198	0.986443	0.421825
TCONS_00024663	XLOC_001937	-	lincRNA	560	chr1	88430899	88442512	-	2	1.12856	0	0	0.178554	0.865022	0	0.500131	1.59758	0
TCONS_00006107	XLOC_000633	-	lincRNA	466	chr1	89533281	89564239	+	3	3.04822	1.35255	1.8888	0.24209	1.41268	1.06663	1.37012	0.623473	0.442043
TCONS_00000987	XLOC_000634	-	anti-sense_lncRNA	2318	chr1	89613469	89616437	+	2	1.9277	0.53392	1.94185	0.060435	0.377269	0.630905	0.41318	0.531386	0.381559
TCONS_00021596	XLOC_001963	-	lincRNA	6498	chr1	94243937	94311809	-	2	2.12454	1.71407	1.61155	1.68591	2.70834	2.25388	2.05793	1.88363	1.79332
TCONS_00021607	XLOC_001978	-	lincRNA	1560	chr1	95081545	95086663	-	2	0.75011	0.88474	0.25688	0.659157	1.49368	1.3972	0.989807	0.355542	0.467572
TCONS_00013175	XLOC_001979	-	lincRNA	10587	chr1	95129225	95171795	-	3	1.52084	1.02677	1.25492	0.68321	1.65949	2.89395	1.13215	0.518582	0.835525
TCONS_00016751	XLOC_001979	-	lincRNA	10164	chr1	95129306	95285790	-	4	0.71248	1.12628	0.91095	0.401733	2.73389	1.63292	1.36635	1.19422	1.10366
TCONS_00009310	XLOC_001979	-	lincRNA	730	chr1	95138471	95149978	-	3	0.53495	2.27438	1.27049	0.24478	3.12699	0	2.00753	0.449222	0
TCONS_00023250	XLOC_001979	-	lincRNA	1755	chr1	95143936	95285858	-	3	2.07084	1.3629	1.33723	1.16796	2.81101	2.53118	1.62341	0.876383	1.44621
TCONS_00009313	XLOC_001979	-	lincRNA	1761	chr1	95208136	95285858	-	2	2.24363	2.67735	2.16945	1.77646	4.2845	5.50263	2.29815	2.20551	2.9321
TCONS_00005095	XLOC_001979	-	lincRNA	9990	chr1	95226177	95285808	-	2	3.32018	2.12348	2.75323	1.45516	5.04572	4.98492	1.97341	2.0591	2.71438
TCONS_00005102	XLOC_001983	-	anti-sense_lncRNA	1727	chr1	95613197	95699580	-	7	6.60171	4.60958	7.48625	0.51907	1.67466	2.15863	4.32616	3.60739	4.43042
TCONS_00014847	XLOC_001983	-	anti-sense_lncRNA	1497	chr1	95629526	95639889	-	2	4.89403	1.36754	2.78523	0.264011	1.61009	1.18475	1.66507	1.33147	1.49458
TCONS_00009314	XLOC_001983	-	anti-sense_lncRNA	1463	chr1	95681927	95699636	-	3	1.28997	1.48292	1.13968	0.115221	0.56178	0.519515	0.887411	0.330348	0.933757
TCONS_00022281	XLOC_000661	-	lincRNA	592	chr1	95714201	95718438	+	2	1.5554	2.1884	2.17376	0.327817	1.11073	1.98427	3.20614	1.67316	0.595906
TCONS_00009322	XLOC_001986	-	lincRNA	605	chr1	99834544	99999693	-	6	0	0.84	0	0	0	0	0	0	0.213314
TCONS_00007204	XLOC_001986	-	lincRNA	663	chr1	99834544	99999693	-	5	0	3.91201	0.6529	0	0.738524	1.05323	6.60801	1.28774	1.08831
TCONS_00023267	XLOC_001986	-	lincRNA	3440	chr1	99888878	99999706	-	6	3.21952	0.78947	1.45358	0.199118	0.956568	1.04085	1.74961	1.03098	1.93865
TCONS_00005110	XLOC_001986	-	lincRNA	3958	chr1	99934635	99999574	-	6	7.37016	1.24382	0.91498	0	2.2004	0.599632	6.05414	1.57717	2.81765
TCONS_00002518	XLOC_001986	-	lincRNA	3887	chr1	99934682	99999827	-	5	0	0.2996	2.23043	5.28E-05	0.0004372	0.114304	0	0.0003366	0.532487
TCONS_00014850	XLOC_001986	-	lincRNA	4106	chr1	99934682	99958585	-	4	0.01694	0.00031	0.1652	0.719402	1.24893	2.56586	0.0020939	1.09105	0.300901
ENST00000438829.2	ENSG00000224445.2	RP11-413P11.1	lincRNA	442	chr1	99937971	99999571	-	4	0	0.69988	1.25929	0.120862	0	0	0.705835	0	1.91828
TCONS_00014852	XLOC_001987	-	lincRNA	273	chr1	100236272	100280051	-	2	7.90958	8.8986	2.0336	1.7302	4.33275	5.75181	7.05624	3.53427	4.06011
TCONS_00010218	XLOC_000680	-	lincRNA	832	chr1	101615967	101629344	+	2	1.12786	1.57518	0.66833	1.72514	1.17448	1.56244	0.935707	0.642679	1.56235
TCONS_00010241	XLOC_000716	-	lincRNA	643	chr1	110613413	110617055	+	3	1.14767	1.45	1.59872	0.289916	0.700856	0.318997	1.07686	0.184552	0.263217
TCONS_00003861	XLOC_000716	-	lincRNA	2713	chr1	110624635	110628588	+	2	5.35045	3.74837	3.89667	0.631707	1.36492	1.8729	1.60085	1.21024	0.85355
TCONS_00010242	XLOC_000716	-	lincRNA	526	chr1	110625802	110651653	+	2	4.1392	3.44574	5.34687	0.827098	0.798443	1.53448	4.06142	1.80492	1.2263
TCONS_00011136	XLOC_002037	-	lincRNA	1222	chr1	112151642	112155824	-	2	1.09743	0.69348	0.82217	1.56707	0.361897	1.30922	0.631814	1.10649	0.453129
TCONS_00010253	XLOC_000732	-	lincRNA	5283	chr1	112170375	112179794	+	2	10.81	6.59481	12.4497	8.34359	9.79162	11.061	7.69879	10.9479	11.7359
TCONS_00002574	XLOC_002042	-	lincRNA	259	chr1	112713844	112715760	-	2	3.15634	0	0	0	0	0	0	1.43384	1.96654
TCONS_00013233	XLOC_002043	-	lincRNA	1741	chr1	112776126	112903328	-	2	0.57713	1.92817	2.37337	0.379215	1.66137	1.59809	5.84705	0.940581	0.882764
TCONS_00024693	XLOC_002043	-	lincRNA	734	chr1	112780421	112903317	-	2	0.24463	0	0.34573	0.345166	0.509347	0	1.2102	0	0.267685
ENST00000422022.1	ENSG00000224167.1	RP3-522D1.1	lincRNA	490	chr1	113392698	113420489	+	2	0.34974	0	0.98729	0.66579	0.431169	0.244399	0.208662	0.855329	0.404767
TCONS_00010262	XLOC_000740	-	lincRNA	945	chr1	113541157	113551121	+	2	1.23079	2.00175	3.11165	1.37677	0.74623	1.32463	0.712303	1.41431	0.856447
ENST00000441071.1	ENSG00000235527.2	RP5-1073O3.7	anti-sense_lncRNA	571	chr1	114466622	114471940	-	3	0	0	0.53972	0	0	0	0.206705	0	0.19036
ENST00000427953.1	ENSG00000235527.2	RP5-1073O3.7	anti-sense_lncRNA	1321	chr1	114466623	114471854	-	2	1.36459	2.08538	1.75911	1.14275	1.15421	0.628067	1.71163	1.15194	1.22798
ENST00000448680.1	ENSG00000233730.1	RP4-666F24.3	lincRNA	491	chr1	115642293	115645279	+	2	19.1699	16.2918	24.1029	1.7693	3.43727	8.28075	20.9994	5.96607	12.5038
TCONS_00024037	XLOC_000749	-	lincRNA	634	chr1	115822890	115824546	+	3	0.93708	1.15146	2.28558	0.591909	0	0.488478	1.51226	0.942313	1.07499
TCONS_00018480	XLOC_002063	-	lincRNA	242	chr1	116465459	116466735	-	2	2.04171	0	1.63776	1.36234	2.76464	0	0	5.69924	0
TCONS_00007268	XLOC_002067	-	lincRNA	1936	chr1	117045560	117047964	-	2	1.1169	1.5887	1.56748	1.32746	1.8068	1.90459	0.942171	1.20518	1.56372
TCONS_00010283	XLOC_000760	-	lincRNA	375	chr1	118302409	118321324	+	2	0.5764	1.24544	3.33532	3.6909	0.723171	4.88505	1.77559	6.74938	6.44786
TCONS_00019272	XLOC_000763	-	anti-sense_lncRNA	1313	chr1	119683178	119722041	+	4	2.3823	2.16409	3.89782	2.53216	2.49117	1.64474	1.79146	2.24813	1.87153
TCONS_00011178	XLOC_002080	-	lincRNA	1099	chr1	120639068	120643525	-	2	0.22677	0.15779	0.62385	0.285107	0.617588	0.705251	0.261612	0.359846	0.257755
TCONS_00006208	XLOC_000770	-	lincRNA	3554	chr1	121116765	121124608	+	2	7.24572	5.36841	6.66143	3.62437	3.4236	4.33857	2.74254	2.77585	2.99176
TCONS_00012088	XLOC_000773	-	lincRNA	793	chr1	121484304	121485436	+	2	664.752	454.007	201.17	90.6499	84.2286	59.6066	154.471	449.63	202.084
TCONS_00012089	XLOC_000775	-	lincRNA	4929	chr1	142794931	142807339	+	2	2.78592	1.77599	0.98537	1.23096	0.778704	0.460717	0.491921	0.898786	0.329098
TCONS_00024052	XLOC_000779	-	lincRNA	980	chr1	143119061	143162803	+	2	2.35143	0.6371	0.18003	0.246532	1.6625	0.632494	4.30565	0.726966	0
TCONS_00015721	XLOC_000790	-	anti-sense_lncRNA	4729	chr1	145002892	145019052	+	3	8.90706	6.10899	4.92984	2.61305	2.69689	3.50718	2.55567	4.55112	4.45471
TCONS_00009427	XLOC_002094	-	lincRNA	2518	chr1	145375156	145377769	-	2	1.67345	0.945	1.98411	0.579772	0.954381	1.36518	1.28297	1.59473	1.17016
TCONS_00007292	XLOC_002096	-	lincRNA	443	chr1	145515371	145516073	-	2	2.91729	2.37335	1.77772	1.06068	1.80769	1.46076	0.753138	3.42364	1.45447
TCONS_00021670	XLOC_002117	-	intronic_lncRNA	447	chr1	147234173	147237255	-	2	1.23021	0.29176	1.16528	0.78254	1.26983	1.72425	0.246782	1.00984	0.238399
TCONS_00005237	XLOC_002128	-	lincRNA	893	chr1	148343961	148346692	-	2	2.05778	1.3329	0.40583	1.38783	1.15944	1.11917	1.53339	2.45842	1.33997
TCONS_00014906	XLOC_002129	-	lincRNA	986	chr1	148350887	148352179	-	2	2.07425	2.07723	2.14364	2.28336	1.88539	2.42094	0.524664	1.54575	1.69668
TCONS_00003974	XLOC_000814	-	lincRNA	1024	chr1	148555737	148557179	+	2	1.48413	2.58365	1.02186	2.2556	1.87275	2.73652	1.78611	1.76838	2.18065
ENST00000442049.1	ENSG00000223779.4	RP11-403I13.4	lincRNA	454	chr1	149239894	149250552	+	3	2.79209	3.11912	3.11318	0.253608	0.246815	1.11751	1.91726	1.635	0.926754
TCONS_00024078	XLOC_000855	-	anti-sense_lncRNA	957	chr1	151727519	151732128	+	3	2.82645	1.12559	1.67004	0.0846805	0.407951	0.931111	0.93444	0.963423	0.996001
TCONS_00011224	XLOC_002164	-	lincRNA	907	chr1	152019760	152021030	-	2	2.01716	0.50242	2.08791	0.816166	0.349628	0.398881	0.667779	0.344156	1.067
TCONS_00001245	XLOC_000859	-	anti-sense_lncRNA	3140	chr1	152221827	152308474	+	4	1.67497	1.39473	1.26688	1.04356	0.812718	1.39159	0.639698	0.932064	0.827536

TCONS_00017787	XLOC_000859	-	anti-sense_lncRNA	556	chr1	152287067	152307837	+	3	0.88098	0.97124	0.92194	2.05656	2.91279	2.23329	0.882531	1.21034	1.46248
TCONS_00006281	XLOC_000859	-	anti-sense_lncRNA	8336	chr1	152297674	152308599	+	2	1.49	1.36292	1.12329	1.01132	1.04389	1.7401	0.517536	0.734558	0.690179
TCONS_00008354	XLOC_000859	-	lincRNA	1409	chr1	152304096	152310643	+	3	0.00037	0.52252	0.71661	0.901291	0	1.46063	0	0.136388	0.500046
TCONS_00014935	XLOC_002179	-	lincRNA	404	chr1	153530979	153533083	-	2	0	0.3548	0.35531	1.58137	3.70705	2.78914	0.301845	0.821432	0.289959
TCONS_00022472	XLOC_000895	-	anti-sense_lncRNA	462	chr1	155165065	155167004	+	2	1.16039	0.54945	0.54817	0.491623	0.717354	1.6246	0.928006	0.949992	1.12233
TCONS_00007393	XLOC_002237	-	lincRNA	411	chr1	156647384	156647882	-	2	0.47926	0.34275	0.34305	0.305705	0.596831	0.336954	1.1651	2.37911	0.840312
TCONS_00014254	XLOC_000922	-	lincRNA	871	chr1	157152522	157155259	+	2	0.45537	0.21184	0.10483	1.14677	0.184273	1.57637	0.616229	0.84672	1.21124
TCONS_00011284	XLOC_002252	-	lincRNA	212	chr1	157622833	157624011	-	2	0	0	6.3004	0	0	0	0	0	0
TCONS_00011287	XLOC_002257	-	lincRNA	301	chr1	158101935	158116600	-	2	0.98112	0.72469	2.21325	0.637464	0.632974	0	0	0.854187	0
TCONS_00013414	XLOC_002274	-	anti-sense_lncRNA	995	chr1	159779495	159796387	-	2	0.25635	1.33928	2.1191	0.725609	0.621298	1.1524	1.40772	1.62991	2.18783
ENST00000528818.1	ENSG00000232995.3	RP11-267N12.3	lincRNA	574	chr1	163288628	163291531	-	2	5.97607	3.82544	6.84264	1.89021	4.82644	3.40435	3.36554	5.70612	3.90634
TCONS_00017868	XLOC_001000	-	lincRNA	1620	chr1	168808648	168871350	+	3	3.52045	2.79081	5.2635	2.3898	2.16715	0.446019	5.56215	5.21977	4.19246
TCONS_00004123	XLOC_001005	-	anti-sense_lncRNA	2503	chr1	169831754	169842893	+	2	2.26057	2.02507	1.21035	0.805856	1.33418	1.31301	1.19009	0.977052	1.3031
TCONS_00006415	XLOC_001006	-	lincRNA	3576	chr1	170285698	170442886	+	3	0.84689	1.96603	0.4948	1.42135	1.07312	1.06195	0.707019	1.11735	0.136654
TCONS_00005401	XLOC_002357	-	anti-sense_lncRNA	1620	chr1	170832603	171220971	-	3	4.00261	1.53931	2.0787	3.8554	1.80589	0.914817	1.13154	1.50585	1.14761
TCONS_00017055	XLOC_002357	-	anti-sense_lncRNA	5073	chr1	171086240	171108163	-	2	9.43127	3.01242	5.59449	2.76983	2.87153	1.39897	2.11227	2.79754	1.98714
TCONS_00021763	XLOC_002357	-	anti-sense_lncRNA	594	chr1	171216591	171220979	-	2	4.71718	8.00323	2.29633	4.15779	1.7893	1.71217	1.4483	0.927711	4.41749
TCONS_00002919	XLOC_002360	-	intronic_lncRNA	4552	chr1	172105344	172113585	-	3	9.21576	14.1585	8.97151	6.34713	7.60506	4.63303	12.5135	10.1762	5.07496
TCONS_00005406	XLOC_002360	-	intronic_lncRNA	5328	chr1	172105344	172114481	-	4	0.99092	2.65282	2.40209	2.12136	1.11368	1.53972	2.50189	2.14062	1.08963
ENST00000417354.1	ENSG00000230630.1	DNM3OS	intronic_lncRNA	568	chr1	172109461	172113934	-	4	18.0884	23.3852	15.9566	8.75855	5.03034	8.41559	19.4189	11.5939	9.67695
TCONS_00001435	XLOC_001022	-	lincRNA	537	chr1	172820381	172828965	+	2	0.90382	0.42522	0.42299	1.14485	1.11007	0.42011	0.357009	0.732719	0.173694
TCONS_00006448	XLOC_001033	-	lincRNA	491	chr1	176197333	176198662	+	2	0.82765	1.39834	4.79E-05	0	2.53051	0.602	0.807311	1.65276	1.14918
TCONS_00014306	XLOC_001033	-	lincRNA	436	chr1	176198113	176234236	+	2	1.55529	0.71376	0.61074	1.91166	1.12307	1.36258	1.32468	1.47603	0.32515
TCONS_00009608	XLOC_002388	-	intronic_lncRNA	3599	chr1	178304125	178310743	-	2	2.1029	1.22563	4.32058	0.639981	2.83624	1.17885	2.41494	1.81813	2.85029
TCONS_00017908	XLOC_001067	-	lincRNA	404	chr1	181150172	181151227	+	3	0.72008	0.68707	0.69621	0.632549	2.0327	0.697251	2.11291	0.821432	0.878216
TCONS_00001489	XLOC_001067	-	lincRNA	495	chr1	181150215	181151433	+	2	3.97038	0.74576	0.73737	0.218145	0.512703	2.43E-05	0	0.280124	0.789836
TCONS_00027684	XLOC_002399	-	lincRNA	1077	chr1	182047856	182287049	-	4	0	0	0.32494	0	0.872116	0.386827	0.333623	1.86865	2.4952
TCONS_00011353	XLOC_002399	-	lincRNA	1869	chr1	182281555	182287049	-	2	0.85597	0.46614	0.91723	0.306847	1.05418	0.977293	0.841123	1.14495	1.84237
TCONS_00010507	XLOC_001094	-	lincRNA	633	chr1	186399883	186404492	+	2	5.16579	1.64879	3.10917	0.296641	1.86487	1.46884	2.61829	1.32235	2.155
TCONS_00004267	XLOC_001094	-	lincRNA	1389	chr1	186403897	186406353	+	2	1.97292	0.89344	1.52931	0.592512	0.984275	1.77621	0.64074	0.94985	2.04323
TCONS_00014349	XLOC_001106	-	lincRNA	999	chr1	192578503	192603573	+	2	0.51015	0.44418	0.08785	2.80765	0.386353	1.23481	0.368569	0.304061	0.58048
TCONS_00017927	XLOC_001113	-	lincRNA	400	chr1	193318317	193325066	+	3	1.01058	1.08619	1.08813	0.967944	0.945781	0.355692	1.8494	0.419291	3.25498
TCONS_00030091	XLOC_002430	-	lincRNA	612	chr1	194136318	194258809	-	2	0.98686	2.60094	1.54946	2.18281	0.60346	1.02945	0.870239	2.18634	3.25786
TCONS_00001553	XLOC_001141	-	anti-sense_lncRNA	3301	chr1	201368674	201372976	+	2	1.31705	0.66042	0.9429	0.433291	1.64377	1.11087	0.957758	0.750752	0.520781
TCONS_00001593	XLOC_001177	-	lincRNA	1002	chr1	204547761	204554678	+	2	0.88943	0.531	1.40027	0.479507	0.846775	0.790827	0.881207	1.41358	1.87945
TCONS_00026513	XLOC_002482	-	lincRNA	1099	chr1	205409197	205419100	-	2	0.34015	0.78893	0.46789	0.142554	0.892072	1.64559	0.850238	0.809654	1.1599
ENST00000417522.1	ENSG00000240754.1	RP11-38J22.6	lincRNA	292	chr1	206288269	206289577	-	2	0.36757	0	3.68208	2.11141	5.20717	9.16161	0	2.44684	6.15428
TCONS_00007578	XLOC_002498	-	lincRNA	540	chr1	206289144	206306125	-	2	0.49471	1.47494	0.72478	1.69682	1.19167	3.35763	1.76872	2.51557	2.15949
ENST00000487977.1	ENSG00000203709.5	C1orf132	lincRNA	3197	chr1	207986905	207996048	-	3	5.41683	5.67914	5.92399	7.21608	5.30768	6.99052	3.16772	4.33984	6.08171
TCONS_00011431	XLOC_002514	-	lincRNA	5004	chr1	208990367	208995768	-	2	0.61652	0.47011	0.15925	0.239684	0.22996	0.307223	0.472116	0.634333	1.30782
TCONS_00011435	XLOC_002520	-	lincRNA	692	chr1	210462941	210468569	-	4	0	0	1.29828	0.130457	0.14873	0.369977	0	0.165817	0
TCONS_00018708	XLOC_002520	-	lincRNA	841	chr1	210463368	210468413	-	3	1.42982	0.12449	0.65474	6.59E-06	0.658047	0.266501	1.22851	0.380114	0.181203
TCONS_00007589	XLOC_002520	-	lincRNA	293	chr1	210463833	210466546	-	2	0	3.04719	0	0	0	0.0001529	1.85512	0	0
TCONS_00012489	XLOC_001226	-	intronic_lncRNA	347	chr1	210914076	210926824	+	2	0.68391	0	2.00095	0	0.432478	0.970761	2.13759	1.15736	0.811667
TCONS_00027175	XLOC_001231	-	lincRNA	1229	chr1	211857330	211860075	+	2	4.35993	2.41056	2.17742	0.373533	0.599036	1.23185	1.25493	1.41286	1.23758
TCONS_00007609	XLOC_002554	-	intronic_lncRNA	2294	chr1	217242963	217252255	-	2	3.94829	2.59901	2.3637	0.397339	1.20352	1.94802	1.97827	1.95805	2.01242
TCONS_00021243	XLOC_001253	-	lincRNA	2192	chr1	219564186	219607322	+	2	1.33218	1.49043	1.08512	0.642613	0.4012	0.918024	0.292983	0.847733	0.347795
TCONS_00024299	XLOC_001263	-	lincRNA	318	chr1	221232546	221240403	+	2	0.84626	0	0	1.09473	0	0	0.539895	5.09457	2.53878
TCONS_00006626	XLOC_001264	-	lincRNA	445	chr1	221250954	221251527	+	2	0.82677	0	0.29378	0.262991	0.256086	0	0	0.339465	0.961554
TCONS_00024905	XLOC_002570	-	lincRNA	1296	chr1	222086436	222089552	-	3	0.09304	0.19393	0.19155	0.292216	0.168668	0.0642427	0.74923	1.3994	0.791961
TCONS_00018025	XLOC_001281	-	intronic_lncRNA	382	chr1	225463616	225465325	+	2	0.55456	0	0	0.3548	1.38924	0	1.02189	0	0.32597
TCONS_00008723	XLOC_001294	-	anti-sense_lncRNA	738	chr1	226320758	226342534	+	3	1.70247	1.58846	1.44291	0.238543	0.806093	1.04958	2.64706	1.05973	1.83817
TCONS_00010661	XLOC_001304	-	lincRNA	394	chr1	227976233	227976831	+	2	3.64502	1.12061	1.87202	0.665404	0.975811	0.733628	0.318332	1.29852	0.915891
TCONS_00009742	XLOC_002607	-	lincRNA	328	chr1	228392031	228393248	-	2	1.56489	2.2856	0.57792	1.00989	1.49482	1.67312	1.48597	0	1.86997
TCONS_00001739	XLOC_001314	-	anti-sense_lncRNA	270	chr1	228696460	228697785	+	2	1.36721	0	0	0.898394	0	0.995779	0.918523	0	0.844108
TCONS_00010675	XLOC_001316	-	lincRNA	1164	chr1	228945698	229048261	+	3	1.05756	0.58835	1.67151	0	0.0639666	0.146126	0.42656	0.419182	0.420481
ENST00000436334.1	ENSG00000177788.5	RP5-1061H20.4	lincRNA	315	chr1	229405113	229406775	-	2	0.86741	3.18347	1.93735	0.561452	2.22226	6.82516	2.21908	1.49495	0
ENST00000416221.1	ENSG00000233461.1	RP11-295G20.2	lincRNA	465	chr1	231658134	231664302	-	3	2.29472	2.17241	0.81264	1.70107	2.12719	2.9444	1.37553	1.87772	2.21873
TCONS_00017288	XLOC_002636	-	lincRNA	922	chr1	233546446	233621345	-	5	0	9.83E-06	0.09733	1.02344	0.216422	0.994211	0	9.13E-06	0.0803516
TCONS_00015160	XLOC_002636	-	lincRNA	1267	chr1	233546450	233621314	-	4	0.2867	0.13281	3.15E-06	4.69495	1.63236	3.04223	0.0549876	0.151338	4.53E-06
TCONS_00017289	XLOC_002636	-	lincRNA	853	chr1	233566951	233621345	-	2	0.93575	0.10886	0.21552	2.28526	0.493321	2.12958	0.0905049	0.124342	0

TCONS_00004483	XLOC_001336	-	lincRNA	588	chr1	234492782	234501727	+	2	1.83324	0.92137	1.09834	0.662426	0.481005	0.729041	2.00586	1.69083	1.80641
TCONS_00001771	XLOC_001339	-	lincRNA	1501	chr1	234887340	234911775	+	3	2.11698	1.1971	1.07443	0.0984418	0.425923	0.324598	0.54006	0.123927	0.266655
TCONS_00015167	XLOC_002647	-	lincRNA	1999	chr1	235095404	235098902	-	2	0.68074	0.55027	0.50411	2.06372	0.820389	0.469225	0.454477	0.447215	0.321019
TCONS_00003231	XLOC_002660	-	lincRNA	370	chr1	240078016	240080394	-	3	3.55842	0.42766	0.85947	0	0.745029	0.419184	0.732461	0.496953	0
TCONS_00014481	XLOC_001355	-	lincRNA	528	chr1	241587980	241596964	+	2	0.61897	0.87417	0	1.96074	0	0.215864	0.367173	0	0.17854
TCONS_00003245	XLOC_002667	-	lincRNA	776	chr1	243255812	243260949	-	3	2.82768	8.40178	3.05965	10.024	3.86941	6.86015	1.74866	2.68352	5.55086
TCONS_00018078	XLOC_001363	-	intronic_lncRNA	2376	chr1	243637243	243641137	+	2	2.25165	1.62391	1.95395	1.41175	2.37259	2.84411	0.777405	1.21903	1.48545
TCONS_00005663	XLOC_002671	-	lincRNA	1054	chr1	244394746	244488466	-	3	4.36072	0.27749	0.2969	0	0.91499	1.2035	0.785268	0.7668	0.571313
TCONS_00020658	XLOC_002671	-	lincRNA	808	chr1	244394861	244488487	-	2	0	1.12656	1.48E-05	0.94947	0.91219	0.0007167	0	0	1.7533
TCONS_00005665	XLOC_002671	-	lincRNA	1101	chr1	244421727	244488953	-	3	0.84406	0.8683	0.18557	0.0711209	0.0941251	0.891833	0.561366	1.42785	0.722271
TCONS_00006704	XLOC_001366	-	lincRNA	1686	chr1	244499875	244504230	+	2	0.58731	1.12859	1.03698	0.162366	0.277597	0.50563	0.255867	0.0769802	0.0388889
TCONS_00008758	XLOC_001366	-	lincRNA	329	chr1	244499986	244506361	+	2	0	1.69569	3.43953	0.501052	0	3.4686	0.982497	0	0
TCONS_00008759	XLOC_001366	-	lincRNA	621	chr1	244500020	244506479	+	2	0.59065	2.25726	6.725	2.0187	1.07693	2.86312	3.90064	0.112706	0
TCONS_00027805	XLOC_002680	-	intronic_lncRNA	1030	chr1	246438977	246440089	-	2	8.96335	5.3007	6.50902	4.47851	9.44433	6.4521	4.53889	6.33913	5.02788
TCONS_00021936	XLOC_002681	-	intronic_lncRNA	847	chr1	246600293	246603844	-	3	2.361	0.32965	1.63145	0.892075	1.83958	2.11201	0.681522	0.878587	0.732351
TCONS_00018789	XLOC_002681	-	intronic_lncRNA	2334	chr1	246600478	246603001	-	2	1.48278	0.62926	1.73128	0.779759	1.28904	1.60249	0.397497	1.09244	1.07766
ENST00000419361.1	ENSG00000227953.2	RP11-439E19.3	lincRNA	552	chr1	246953926	246955687	+	2	1.73108	1.42388	0.80902	0.730536	1.77	0.201038	0.341264	0	0.166175
TCONS_00036347	XLOC_003284	-	lincRNA	412	chr10	309039	309605	-	2	0.95404	0.68219	0.68274	0	1.48486	0.335348	0	0	0.27875
TCONS_00035060	XLOC_002717	-	lincRNA	787	chr10	4915235	4975806	+	4	1.54546	1.69656	1.03944	0.711013	1.11194	0.360591	0.605365	0.583071	0.139268
TCONS_00036711	XLOC_002717	-	lincRNA	647	chr10	4931657	4975472	+	4	0.2477	0.47962	0.68786	1.36385	0.202142	0.158063	0	0.510482	0.599063
TCONS_00035061	XLOC_002717	-	lincRNA	332	chr10	4991816	4995082	+	2	0	0.55382	0.5597	0	0	0	0.479396	3.23813	0.453055
TCONS_00034268	XLOC_002733	-	lincRNA	1694	chr10	6629028	6657792	+	5	1.29755	1.23132	1.21526	1.07136	0.906134	0.659382	0.508869	0.700813	1.43116
TCONS_00031932	XLOC_002733	-	lincRNA	437	chr10	6660693	6663270	+	2	0.42721	0.30434	1.21625	0.543867	0.264931	0	0.257724	0	1.49213
TCONS_00038086	XLOC_003324	-	lincRNA	1557	chr10	6976337	7198205	-	4	0.2276	0.31295	0.31566	1.41561	0.589727	0.518623	0.262041	0.177719	0.643506
TCONS_00044834	XLOC_003324	-	lincRNA	1103	chr10	6976379	7077205	-	3	0.10978	0	0.53329	0	0	7.90E-06	0.450852	1.88141	1.08381
TCONS_00040842	XLOC_002751	-	lincRNA	347	chr10	13425135	13442192	+	2	0	0.49626	1.50072	0	0.864956	2.4269	1.28255	1.73605	1.62333
TCONS_00038408	XLOC_002754	-	anti-sense_lncRNA	9178	chr10	13682621	13692193	+	2	5.38174	4.07769	4.73679	2.21549	3.7714	3.42003	2.81443	3.65888	3.59334
TCONS_00033207	XLOC_002755	-	anti-sense_lncRNA	2030	chr10	13709066	13712528	+	4	1.31057	0.90303	1.03371	0.205251	0.998337	1.55469	0.701956	0.424714	0.973898
TCONS_00036044	XLOC_002755	-	intronic_lncRNA	1092	chr10	13709595	13711890	+	2	0.97127	0.84509	0.85524	0.656018	1.19578	0.0437667	0	1.02813	1.5677
TCONS_00035544	XLOC_003349	-	anti-sense_lncRNA	435	chr10	14909735	14920720	-	2	0.43081	1.53495	0.92023	0.548529	1.87067	0.3022	1.04012	0.354473	0.250853
ENST00000449529.1	ENSG00000152487.6	ARL5B-AS1	lincRNA	689	chr10	18943480	18948185	-	2	7.69732	3.64463	5.34864	2.49412	2.9171	2.74387	2.55413	3.33708	1.54812
TCONS_00034706	XLOC_003366	-	intronic_lncRNA	297	chr10	20011331	20017609	-	2	2.03793	6.03297	0	3.3143	3.29456	0.733256	0.662772	4.45011	1.23576
TCONS_00038124	XLOC_003367	-	intronic_lncRNA	2205	chr10	21234047	21249586	-	3	3.19956	1.67615	2.37184	1.83212	4.1911	3.2818	3.69546	4.08092	4.54629
TCONS_00032642	XLOC_003367	-	intronic_lncRNA	1009	chr10	21234256	21249556	-	4	6.94706	3.19837	6.22867	1.87022	4.29705	3.00053	7.20918	7.73153	5.23945
TCONS_00039635	XLOC_003373	-	lincRNA	2897	chr10	23492863	23542555	-	6	2.27128	1.15242	2.34998	0.28471	1.54886	0.782099	0.950633	0.92316	0.898428
TCONS_00034305	XLOC_002790	-	lincRNA	514	chr10	23788457	23819067	+	2	0.64638	2.513	0.9097	0	0.994049	1.12784	1.34465	0.525335	0
TCONS_00034313	XLOC_002815	-	lincRNA	710	chr10	30397692	30400146	+	2	1.39654	2.3752	0.55401	0.251688	0.243099	0.553749	0.465984	0.159856	0.228258
ENST00000433770.1	ENSG00000237797.1	RP11-472N13.3	lincRNA	347	chr10	31982012	31996316	-	2	0	0.49626	1.50072	0.439754	0	0	0.427518	1.73605	0.405833
TCONS_00037820	XLOC_002828	-	intronic_lncRNA	439	chr10	32923016	32924146	+	2	0.84733	1.20697	0.90428	2.69641	0.525327	1.18837	2.81005	3.13483	2.4656
TCONS_00036846	XLOC_002829	-	intronic_lncRNA	1728	chr10	34921922	34924167	+	3	1.53582	0.60191	2.5589	0.167599	0.442966	0.322342	1.79851	0.527018	0.756321
TCONS_00036088	XLOC_002844	-	lincRNA	557	chr10	38692108	38697019	+	2	1.70698	0.60156	0.7974	0.180063	1.22137	0.396404	0	0.230194	0.491433
TCONS_00037388	XLOC_003418	-	lincRNA	788	chr10	42383107	42385731	-	2	130.646	165.489	25.9945	16.1464	10.5116	3.95972	55.0828	159.254	52.2645
TCONS_00035646	XLOC_003418	-	lincRNA	912	chr10	42383461	42389980	-	2	12.3636	5.83116	3.16974	1.08056	0.533352	0.0990175	2.99217	9.74571	2.7912
TCONS_00037389	XLOC_003418	-	lincRNA	566	chr10	42387085	42394591	-	2	187.064	161.691	38.0885	17.3856	10.8879	5.21893	51.7877	176.817	58.6177
TCONS_00032728	XLOC_003418	-	lincRNA	2076	chr10	42390288	42597886	-	2	51.8997	79.6704	11.1907	4.36728	6.91565	1.68727	25.582	36.2738	12.5584
TCONS_00044425	XLOC_003418	-	lincRNA	537	chr10	42392974	42396759	-	2	14.7624	9.99277	2.11495	0.572425	1.4801	0.210055	4.99813	9.2811	1.91063
ENST00000442526.2	ENSG00000230555.2	RP11-517P14.2	lincRNA	760	chr10	43916186	43917548	+	2	0.9088	0.38136	2.39283	1.71855	0.995273	1.00815	1.27039	1.30807	0.934534
TCONS_00042624	XLOC_003437	-	intronic_lncRNA	1275	chr10	46081346	46082698	-	2	5.97603	1.97733	3.71119	1.19174	3.26762	1.70302	1.85551	2.32823	3.01471
TCONS_00033335	XLOC_002888	-	lincRNA	652	chr10	48185738	48187765	+	2	0.89994	0.63146	0.93975	0.568239	1.37345	0.312608	0.791123	0.903998	1.28956
TCONS_00042629	XLOC_003459	-	lincRNA	397	chr10	49294700	49313272	-	3	4.10311	4.41254	2.57925	0.655196	0.320188	1.0835	1.25281	0	1.20212
TCONS_00033357	XLOC_002907	-	lincRNA	675	chr10	52391425	52402660	+	2	1.85738	0.60053	0.5956	0.135154	0.391842	0.546137	1.12161	0.65411	0.735826
TCONS_00037845	XLOC_002907	-	lincRNA	4771	chr10	52391449	52396871	+	2	4.11101	1.5904	3.42368	2.687	5.15618	3.7517	2.78661	4.22849	2.86262
TCONS_00036890	XLOC_002907	-	lincRNA	825	chr10	52402103	52407702	+	2	0.86778	0.11378	1.46451	0.307857	0.197959	0.262634	0.477819	0.155238	0.185883
TCONS_00035229	XLOC_002916	-	intronic_lncRNA	1390	chr10	60347210	60350136	+	3	3.34258	2.97561	2.70341	1.23785	1.0352	1.83387	1.28039	0.67789	1.11797
TCONS_00044675	XLOC_002917	-	intronic_lncRNA	2426	chr10	60361288	60363772	+	2	3.11825	3.20643	3.22489	2.18501	1.32523	1.89556	1.10047	1.73313	1.60757
TCONS_00032101	XLOC_002928	-	lincRNA	786	chr10	65390659	65394378	+	2	3.30078	1.3357	1.32292	2.62729	0.633789	0.602014	0.60642	1.2491	0.892674
TCONS_00034366	XLOC_002929	-	lincRNA	1410	chr10	67330047	67345904	+	4	3.9594	2.35171	1.6068	0.381234	1.95755	0.250661	1.21076	0.967958	0.595287
TCONS_00042347	XLOC_002929	-	lincRNA	1655	chr10	67330102	67376037	+	5	3.44695	3.24624	1.78819	1.75453	1.775	1.87285	2.11979	1.57076	1.39852
TCONS_00032102	XLOC_002929	-	lincRNA	1247	chr10	67330285	67333551	+	3	0.86269	0.4872	0.83667	0.139862	0.552363	0.984034	0.426264	0.553999	0.0999198
TCONS_00032105	XLOC_002929	-	lincRNA	2363	chr10	67344653	67376848	+	2	3.68147	1.40066	1.85072	0.226041	1.62515	1.73416	0.368476	0.963434	0.574541
TCONS_00039414	XLOC_002930	-	lincRNA	310	chr10	67616845	67637175	+	2	0	0	0	0	2.32428	0.64835	1.16268	0	0.545031

TCONS_00040929	XLOC_002979	-	intronic_lncRNA	2541	chr10	74548364	74551455	+	2	3.31406	1.69029	2.14314	1.94091	3.72743	3.99517	1.34502	2.12821	2.21867
TCONS_00036544	XLOC_003548	-	lincRNA	701	chr10	75381375	75385597	-	2	3.45226	0.85353	1.97447	0.38434	0.866306	1.1275	1.06776	0.325563	0.581011
TCONS_00032185	XLOC_002998	-	lincRNA	470	chr10	77043766	77045628	+	2	0	1.33212	0.2657	0	0.927546	0.525341	1.57373	0.613918	0
ENST00000449852.1	ENSG00000233871.1	DLG5-AS1	lincRNA	487	chr10	79687130	79689582	+	2	4.24036	1.5021	1.2474	1.12132	1.96094	1.72883	2.95311	2.01731	2.04539
TCONS_00037891	XLOC_003006	-	lincRNA	323	chr10	80113911	80115763	+	2	0	0	0	2.10133	1.03783	0.580342	1.03341	1.39418	0.486829
ENST00000601369.1	ENSG00000226381.3	RP11-119F19.2	lincRNA	616	chr10	81451256	81526085	-	2	3.66532	4.97949	2.3866	2.62496	3.43607	4.75763	5.31334	1.37762	3.08585
TCONS_00032873	XLOC_003573	-	lincRNA	4185	chr10	81555029	81575597	-	5	2.30635	1.36402	2.4617	2.00671	2.64964	3.10435	1.50391	1.50988	2.63311
TCONS_00036975	XLOC_003013	-	lincRNA	2407	chr10	81586793	81590076	+	2	7.90892	4.00261	6.53711	2.52282	4.59481	3.56887	4.96855	3.89683	3.89671
TCONS_00039776	XLOC_003589	-	lincRNA	487	chr10	89260190	89264192	-	2	0.35336	0.75105	0.99792	0.224265	0.653647	0.246976	0.210936	0.576374	1.63632
TCONS_00037911	XLOC_003048	-	lincRNA	503	chr10	89802095	89819924	+	3	0.33484	0.47379	0.47185	3.39761	0	0	0.598045	0.544999	0.967708
TCONS_00032236	XLOC_003060	-	lincRNA	7128	chr10	90939114	90948660	+	2	2.18205	1.65991	1.40462	0.987853	1.84196	0.963566	0.780059	0.798214	0.399211
TCONS_00037920	XLOC_003067	-	lincRNA	4559	chr10	91647808	91678714	+	2	0.48757	0.6892	0.6506	0.869085	0.880021	0.280387	0.541643	1.03851	1.22033
TCONS_00034454	XLOC_003067	-	lincRNA	1395	chr10	91648715	91675595	+	2	0.27218	0.91485	0.77427	1.00653	0.541452	0.625062	1.04998	1.72548	0.615875
TCONS_00034455	XLOC_003068	-	lincRNA	546	chr10	91825510	91826869	+	2	0.58697	0.41399	0.61756	0.371637	0.720481	0	0.347392	1.9017	1.01462
TCONS_00036573	XLOC_003605	-	lincRNA	1500	chr10	92911775	92914017	-	2	3.76641	2.23267	2.6344	1.77332	1.4682	1.67838	1.17104	1.86035	1.60116
TCONS_00032926	XLOC_003620	-	anti-sense_lncRNA	1809	chr10	96984619	96988928	-	3	2.1562	0.65943	0.65073	1.79045	0.841141	1.39926	1.05342	0.850566	0.825832
TCONS_00037990	XLOC_003163	-	lincRNA	361	chr10	109449333	109462662	+	4	0	0.90433	2.27434	2.0069	2.75744	1.32855	1.16415	1.57826	1.10905
TCONS_00039240	XLOC_003706	-	intronic_lncRNA	1125	chr10	111672593	111674179	-	2	1.32245	0.45998	0.90935	0.900501	0.600122	1.67535	0.635403	1.48592	1.62803
TCONS_00033056	XLOC_003720	-	lincRNA	4085	chr10	115241616	115252075	-	2	1.86141	1.02923	1.64597	2.68073	1.40254	2.38313	2.05228	0.937566	0.783471
TCONS_00042758	XLOC_003720	-	lincRNA	3914	chr10	115246723	115252132	-	2	1.21485	0.5771	0.88235	0.675374	0.632178	2.16293	3.05532	1.07158	0.205045
TCONS_00037677	XLOC_003728	-	lincRNA	1582	chr10	115717190	115720554	-	2	1.25491	0.56333	1.16272	0.370657	0.534475	0.305537	0.42344	0.466458	0.209136
TCONS_00040748	XLOC_003736	-	lincRNA	1644	chr10	118591885	118595264	-	3	7.34797	3.3323	6.7233	1.33021	3.49481	4.58085	2.87625	2.84516	4.08248
TCONS_00035004	XLOC_003755	-	lincRNA	1050	chr10	122952485	123003281	-	2	0.95923	0.75124	1.89803	0	0.290413	0.0828924	0.0692275	0	0.0681778
TCONS_00038320	XLOC_003763	-	lincRNA	623	chr10	125471687	125472853	-	2	0.48066	0.33766	0	2.27764	0.293777	0.668345	0.423474	0.580421	0.275828
TCONS_00038327	XLOC_003777	-	lincRNA	349	chr10	127473742	127474478	-	2	1.34987	0.48946	0	1.30151	0.426524	0.957641	0.42141	0	1.20076
TCONS_00032490	XLOC_003247	-	lincRNA	239	chr10	128112956	128113529	+	2	4.29612	0	1.73088	1.43625	1.45938	0	0	0	0
TCONS_00034643	XLOC_003270	-	intronic_lncRNA	462	chr10	134222676	134223674	+	2	0.77359	1.09889	1.37043	1.22906	1.43471	0.541534	1.85601	0.633328	0.6734
TCONS_00036698	XLOC_003803	-	intronic_lncRNA	564	chr10	134665303	134666070	-	2	1.30044	0.78642	1.44801	0.214555	0.369015	1.03908	1.77844	2.06634	0.802573
TCONS_00035041	XLOC_003803	-	intronic_lncRNA	508	chr10	134665359	134665968	-	2	10.6541	2.56281	2.68878	0.790611	1.9957	1.30039	2.78483	1.56553	2.47504
TCONS_00038336	XLOC_003802	-	lincRNA	586	chr10	134679484	134681272	-	2	3.42214	1.85241	2.0243	1.49822	0.967069	0.183211	2.17171	0.212476	1.36193
ENST00000534742.1	ENSG00000255026.1	RP11-326C3.2	lincRNA	812	chr11	288038	288987	+	2	1.33096	3.25423	1.84131	0.838444	0.505509	0.691595	1.25697	2.52323	0.75947
ENST00000602429.1	ENSG00000251661.3	RP11-326C3.11	anti-sense_lncRNA	1151	chr11	318653	325631	+	2	1.92957	2.60941	2.79973	0.538974	2.13993	0.962842	3.08904	1.4449	1.03521
TCONS_00057978	XLOC_003859	-	lincRNA	479	chr11	2356875	2363383	+	3	0	1.03066	0.25686	0	0.224256	0.979746	1.08625	0	0.791376
TCONS_00045857	XLOC_003859	-	lincRNA	738	chr11	2356926	2358067	+	2	1.70247	0.52949	0.39352	0.238543	0.690937	0.150165	0.330882	0.605558	0.242298
TCONS_00045862	XLOC_003865	-	lincRNA	589	chr11	3302131	3372370	+	2	1.04489	0.18379	0.18258	0.165179	0.799584	0.363577	0	0	0.300284
TCONS_00047494	XLOC_003867	-	lincRNA	512	chr11	3533478	3542144	+	3	2.60198	1.60961	1.14462	0	2.40129	0.681075	1.54698	0	1.12716
ENST00000415809.1	ENSG00000228661.1	AC090587.5	lincRNA	494	chr11	3875548	3876739	-	2	0.69003	0.48856	0.97345	0.218892	0	0.48209	0.41141	0.281107	0.199578
TCONS_00058641	XLOC_003881	-	lincRNA	713	chr11	6344799	6351716	+	2	2.38012	1.25003	2.47819	0.750633	0.845815	0.963365	1.50538	2.22465	0.226909
TCONS_00048097	XLOC_004599	-	lincRNA	1488	chr11	6754007	6767693	-	2	2.29671	2.63832	5.75236	4.17635	2.29462	3.44275	3.45523	5.57083	8.79902
TCONS_00053249	XLOC_004602	-	intronic_lncRNA	4335	chr11	7458677	7471664	-	2	2.42031	1.60468	1.74972	1.43881	2.12288	2.56671	0.900468	1.95938	1.97978
TCONS_00056785	XLOC_004621	-	lincRNA	505	chr11	9142160	9143107	-	2	0.33266	0.23532	0	0.632849	0.409571	0.464558	0.593966	0.541315	1.92249
TCONS_00050750	XLOC_003916	-	intronic_lncRNA	1942	chr11	12217262	12220489	+	2	1.34738	1.29904	2.96391	2.57228	1.55342	1.17112	0.771242	2.21725	2.18827
TCONS_00047584	XLOC_003947	-	anti-sense_lncRNA	3909	chr11	19217909	19316459	+	5	18.6961	12.6969	11.4321	1.99666	3.04041	4.43941	7.67599	2.72033	5.69152
TCONS_00047580	XLOC_003947	-	anti-sense_lncRNA	921	chr11	19218163	19274537	+	3	7.47878	7.6802	3.47479	0.555237	0.884631	2.02926	4.40276	1.71552	2.64671
TCONS_00053726	XLOC_003947	-	lincRNA	4699	chr11	19330376	19335385	+	2	9.46582	7.27932	5.41113	1.15173	1.75991	2.46828	2.89597	1.05179	3.25443
TCONS_00048685	XLOC_003947	-	lincRNA	3649	chr11	19349530	19353695	+	2	6.2469	3.45973	1.99773	0.296799	0.854539	1.1006	1.56966	0.930754	1.65524
TCONS_00050782	XLOC_003959	-	lincRNA	502	chr11	22856486	22860151	+	2	1.00782	0.71308	1.89382	1.06529	0.413716	0.234606	0.600106	1.36715	1.16517
TCONS_00051288	XLOC_004677	-	lincRNA	4229	chr11	27168785	27255720	-	3	0.83588	0.45809	0.36933	0.439821	1.42873	3.39548	0.694146	0.981198	1.16753
TCONS_00056024	XLOC_004677	-	lincRNA	1910	chr11	27171141	27241420	-	3	0.33266	0.49027	0.56513	0.546987	1.51891	1.86335	0.548534	1.4579	1.45224
TCONS_00056827	XLOC_004677	-	lincRNA	2836	chr11	27237200	27241213	-	2	0.94685	0.50918	0.57504	0.720707	1.37503	2.21593	0.97001	1.88138	1.43174
TCONS_00050788	XLOC_003969	-	lincRNA	1029	chr11	27532396	27535272	+	2	3.07333	1.28398	2.62371	0.309236	1.78692	1.86997	1.56215	1.95288	0.908915
TCONS_00050791	XLOC_003970	-	intronic_lncRNA	5774	chr11	28246675	28255093	+	3	6.81638	3.0334	6.01246	3.13775	5.13015	4.66488	3.82768	4.86149	3.93923
TCONS_00047606	XLOC_003970	-	intronic_lncRNA	831	chr11	28251844	28255171	+	2	6.77787	1.95949	5.02056	4.32723	4.01923	2.79452	2.9991	2.7036	3.31379
TCONS_00045980	XLOC_003970	-	lincRNA	1331	chr11	28433497	28447147	+	2	2.25424	1.12737	0.80418	0.736211	0.980531	1.05825	0.466582	1.49845	0.818488
TCONS_00054728	XLOC_003971	-	lincRNA	1810	chr11	28547519	28549423	+	2	1.58446	0.61509	0.56361	0.437394	0.611357	0.655496	0.508231	0.800032	0.825318
TCONS_00049749	XLOC_003979	-	lincRNA	1775	chr11	30795298	30815442	+	3	2.20269	1.572	1.01944	2.19498	0.859364	0.625414	0.445382	0.715677	0.0366836
TCONS_00048192	XLOC_004688	-	lincRNA	4458	chr11	30836099	30853975	-	2	2.42238	0.67967	0.81686	0.165264	0.504557	0.478697	0.737761	0.62166	0.988336
TCONS_00059822	XLOC_004688	-	lincRNA	1848	chr11	30850369	30852897	-	2	1.17646	0.98712	0.63524	0	0.671863	0.213453	0.921934	1.17222	0.946436
TCONS_00046840	XLOC_004691	-	intronic_lncRNA	3614	chr11	31523722	31527779	-	2	4.69579	2.00615	2.99681	1.21811	3.25541	2.51206	1.79043	2.6329	3.17557
TCONS_00046009	XLOC_004001	-	lincRNA	817	chr11	34632537	34634403	+	2	2.64043	2.42072	2.05445	0.519768	1.70467	0.800295	0.671236	0.395126	0.188315
TCONS_00048719	XLOC_004003	-	lincRNA	4428	chr11	34733082	34776587	+	3	0.94201	0.90153	0.80622	0.24209	2.01805	0.398975	0.371505	1.17627	0.245431

TCONS_00056843	XLOC_004708	-	lincRNA	1543	chr11	35038957	35088718	-	4	0.08818	0.29167	0.83174	0.393398	0.175821	0.907417	0.221071	0.436593	0
TCONS_00049251	XLOC_004708	-	lincRNA	1724	chr11	35085628	35088718	-	2	0.45788	1.08936	0.59626	0.955684	0.975613	0.954997	0.419267	1.20081	0.872061
TCONS_00046870	XLOC_004717	-	lincRNA	1543	chr11	40668370	40955268	-	2	2.27876	0.68516	0.83249	0.0476795	0.5959	0.419236	0.523017	1.68034	0.172187
TCONS_00046872	XLOC_004718	-	lincRNA	470	chr11	41244411	41482011	-	2	2.56854	0.99676	1.81941	0	0.630136	0.791349	0.770267	2.66151	0.0591431
TCONS_00046871	XLOC_004718	-	lincRNA	1179	chr11	41244411	41266721	-	3	5.00651	0.86178	0.68332	0	0.230682	0	1.38499	1.76647	0
TCONS_00055596	XLOC_004019	-	lincRNA	2033	chr11	43879217	43881307	+	2	3.619	3.2402	2.58772	2.30476	2.44892	2.64793	1.81582	2.1505	2.42586
TCONS_00052895	XLOC_004024	-	intronic_lncRNA	2717	chr11	44322406	44328139	+	2	0.60839	0.36489	0.33213	1.95763	0.317328	0.698407	0.277894	0.542587	0.48141
TCONS_00048739	XLOC_004025	-	lincRNA	469	chr11	44360830	44525249	+	2	0.37681	1.87204	1.06689	0.239372	0	0.263656	0.677075	0	0
TCONS_00049259	XLOC_004723	-	intronic_lncRNA	1647	chr11	45161264	45164016	-	2	2.39725	1.46707	1.25497	0.0884963	0.510372	0.486313	0.68724	0.723724	0.878703
TCONS_00058425	XLOC_004765	-	anti-sense_lncRNA	1850	chr11	57298292	57310973	-	2	0.24738	0.21433	0.16919	0.19399	0.260962	0.255832	0.318741	0.682961	1.08533
TCONS_00046940	XLOC_004774	-	lincRNA	6724	chr11	58896278	58904397	-	3	3.44334	3.20593	3.94185	1.09399	2.90484	2.17577	2.22303	2.91346	3.08002
ENST00000532845.1	ENSG00000245571.2	AP001258.4	lincRNA	516	chr11	58903398	58907588	-	2	1.30588	0.80724	2.27111	1.10241	1.8015	1.79302	1.61234	4.13813	1.53547
TCONS_00046942	XLOC_004776	-	lincRNA	1432	chr11	59038403	59052044	-	3	2.64929	1.32191	2.55384	1.09162	2.74931	1.94262	0.665706	2.29111	1.68994
TCONS_00058074	XLOC_004078	-	lincRNA	743	chr11	60674261	60677586	+	2	1.12457	1.04912	1.29946	0.354509	0.456338	0.909884	1.20181	1.34974	1.17834
TCONS_00046154	XLOC_004106	-	intronic_lncRNA	474	chr11	62225986	62238717	+	2	1.84997	0	0	0	0	0	0	0.302326	0
TCONS_00052951	XLOC_004124	-	intronic_lncRNA	375	chr11	63590086	63590746	+	2	0	0	0.41692	1.84545	0	0.814175	0.355118	0	0.339361
TCONS_00049343	XLOC_004834	-	lincRNA	746	chr11	64015388	64016361	-	2	0.1864	1.43454	0.25844	0.470072	0.340354	0.258532	0.760491	0.447398	0.426107
ENST00000499732.1	ENSG00000245532.4	NEAT1	lincRNA	1745	chr11	65190245	65192232	+	2	38.3858	100.419	50.03	172.393	76.567	110.98	32.7914	80.8779	69.0475
TCONS_00055658	XLOC_004156	-	lincRNA	2043	chr11	65190277	65192644	+	3	48.0523	49.4876	56.5769	75.6351	78.0167	97.864	49.5976	54.6561	54.0414
TCONS_00049886	XLOC_004156	-	lincRNA	10347	chr11	65193896	65211439	+	3	7.57803	39.413	12.9863	41.4173	28.2979	37.1251	12.7819	22.9071	28.8235
TCONS_00057282	XLOC_004156	-	lincRNA	11432	chr11	65193898	65211621	+	2	47.6177	141.625	32.158	75.9986	67.8739	74.0206	41.6656	52.1371	61.9227
TCONS_00049882	XLOC_004156	-	lincRNA	2221	chr11	65201356	65204968	+	2	0.23747	0.69981	0.22359	0.875336	0.290363	0.839298	0	0	0.207199
TCONS_00053854	XLOC_004158	-	lincRNA	380	chr11	65265170	65266599	+	3	0	0	0.4598	0.958278	0.881235	0	0.371591	0	0.33966
TCONS_00047764	XLOC_004158	-	lincRNA	850	chr11	65265194	65266650	+	3	10.0273	7.10908	9.83715	14.7329	10.0364	10.416	8.54036	10.1189	8.66289
TCONS_00047768	XLOC_004158	-	lincRNA	5023	chr11	65268350	65273491	+	2	5267.65	2856.41	5078.53	0	0	0	3564.51	4764.74	4086.63
TCONS_00048848	XLOC_004163	-	lincRNA	525	chr11	65433405	65439134	+	2	0.93697	1.32356	1.53662	1.58315	0.191963	1.52511	0.556035	0.760559	0.901084
TCONS_00049382	XLOC_004880	-	lincRNA	2359	chr11	66012233	66015654	-	2	1.13468	1.11298	0.35514	0.266788	0.512426	0.586335	0.513367	0.595715	0.320824
TCONS_00048861	XLOC_004179	-	lincRNA	655	chr11	66115284	66116924	+	2	1.34105	1.72492	1.40015	1.69343	1.5007	1.55268	1.30961	2.15499	1.02476
ENST00000534517.1	ENSG00000255031.1	RP11-802E16.3	anti-sense_lncRNA	664	chr11	67820290	67821229	+	2	0.21919	0.15373	0	0	0.133749	0.152239	0.128353	0	0
ENST00000512200.1	ENSG00000250508.1	RP11-757G1.6	lincRNA	3252	chr11	68638132	68642010	+	2	1.17091	0.71732	0.77559	0.880504	0.845165	1.01354	0.267152	0.605012	0.963822
TCONS_00056948	XLOC_004920	-	lincRNA	238	chr11	68804881	68808163	-	2	0	0	0	0	0	0	1.54696	0	1.39062
TCONS_00047127	XLOC_004929	-	lincRNA	286	chr11	70055303	70058619	-	2	2.27737	2.54278	2.60128	0.743465	0	0	1.49923	0	0
TCONS_00053020	XLOC_004233	-	intronic_lncRNA	1100	chr11	71186269	71187946	+	2	1.69887	1.02447	1.09051	5.05502	1.57652	1.25238	0.783951	2.06679	2.5103
TCONS_00053440	XLOC_004933	-	lincRNA	686	chr11	71512288	71513975	-	2	1.4652	0.73348	0	1.05666	0.255246	0.290621	0.24479	0.167904	0.11983
TCONS_00051997	XLOC_004250	-	lincRNA	2106	chr11	72911033	72918216	+	3	0.14384	0.40599	0.14843	0.0715712	0.183373	0.57422	1.05993	0.409166	0.630023
TCONS_00046325	XLOC_004250	-	lincRNA	2242	chr11	72911418	72918117	+	3	1.33682	1.07496	1.65315	0.434765	1.07567	0.566248	2.89778	1.74447	1.31154
TCONS_00046326	XLOC_004250	-	lincRNA	1719	chr11	72911527	72918090	+	3	1.24727	0	0.85155	0.126345	0.428708	0	0.538545	0	0.373267
TCONS_00049429	XLOC_004946	-	lincRNA	260	chr11	72926864	72929110	-	2	3.11332	1.17742	0	1.02775	0	0	3.18345	1.41251	0
TCONS_00057329	XLOC_004262	-	anti-sense_lncRNA	3464	chr11	74559160	74570328	+	3	0.15621	0.16492	0	0.353999	0.0569053	0.175391	0.141153	0.073492	0.0989448
TCONS_00055723	XLOC_004262	-	anti-sense_lncRNA	9475	chr11	74559160	74574066	+	2	3.18301	2.42128	2.28499	2.13723	2.69808	2.91548	1.46597	2.10779	3.03349
TCONS_00053939	XLOC_004263	-	intronic_lncRNA	1827	chr11	74585309	74588698	+	3	1.31706	1.52159	1.71597	1.69193	1.58799	2.20536	0.646562	0.989531	2.05942
ENST00000527803.1	ENSG00000255326.1	CTD-2530H12.4	anti-sense_lncRNA	651	chr11	75294241	75305710	+	2	0.45096	0.47466	0.62792	0.142376	0.137653	0.156653	0.132154	0.543627	0.646225
ENST00000528075.1	ENSG00000255100.1	RP11-21L23.3	lincRNA	336	chr11	76493625	76494106	-	2	2.21331	2.68623	4.88295	2.85203	2.81009	1.57455	0.464419	3.76645	0.878908
TCONS_00056977	XLOC_004979	-	intronic_lncRNA	2498	chr11	77519414	77522230	-	2	4.84191	2.61374	3.42671	1.92156	4.46589	3.8252	1.77635	3.53207	3.56622
ENST00000532831.1	ENSG00000246174.3	KCTD21-AS1	anti-sense_lncRNA	856	chr11	77850817	77853105	+	2	2.17347	2.60065	2.03791	0.684281	1.60693	0.956308	3.12637	1.98025	1.80522
ENST00000527321.1	ENSG00000246174.3	KCTD21-AS1	lincRNA	586	chr11	77850861	77852968	+	2	0	5.46E-05	0	1.45E-05	0.314697	0.568766	0.82133	0	1.15081
TCONS_00049484	XLOC_005004	-	lincRNA	675	chr11	85564771	85565950	-	2	1.92726	1.95171	1.63789	0.270307	0.783684	0	0.626516	0.85935	0.245275
TCONS_00052037	XLOC_004301	-	lincRNA	11166	chr11	86411219	86422606	+	2	4.6485	1.60151	3.75067	1.05561	3.97663	4.0044	2.15536	1.99424	1.24492
TCONS_00049984	XLOC_004304	-	lincRNA	3774	chr11	87069743	87266594	+	3	0.91428	1.14577	0.91516	3.04314	1.88972	3.67795	1.54715	3.18831	5.56622
TCONS_00053981	XLOC_004304	-	lincRNA	6951	chr11	87266925	87275516	+	2	0.48416	0.742	0.54571	1.90435	1.47225	2.91447	0.7489	2.01825	3.39698
TCONS_00053066	XLOC_004305	-	lincRNA	1098	chr11	88071017	88144456	+	4	2.04318	1.34269	2.26399	1.07035	1.85484	1.33363	3.40479	2.16151	0.967665
TCONS_00056634	XLOC_004306	-	lincRNA	417	chr11	88157600	88160432	+	2	0	0.33306	0	1.18876	1.15985	1.31016	0.282785	3.46594	0
TCONS_00053071	XLOC_004314	-	lincRNA	237	chr11	90094676	90102414	+	2	0	0	0	4.46793	3.02948	0	1.57731	0	1.41681
TCONS_00051038	XLOC_004317	-	lincRNA	1175	chr11	92980982	92985352	+	2	0.72114	0.96308	0.41791	0	0.25116	0	0.843017	0.599585	0.492759
TCONS_00046409	XLOC_004317	-	lincRNA	8317	chr11	92981247	92992465	+	3	2.69947	0.97658	1.31356	0.181231	0.547067	0.508452	1.11902	0.925638	0.859848
TCONS_00051037	XLOC_004317	-	lincRNA	977	chr11	92981337	92983925	+	2	0.73371	2.0453	0.611	0	0.368844	0.568189	1.03572	0	0.414905
TCONS_00056239	XLOC_005018	-	anti-sense_lncRNA	1213	chr11	93097266	93098858	-	2	2.01302	1.0495	0.62216	0.442685	1.15625	0.62568	1.33294	0.877179	0.857207
TCONS_00046420	XLOC_004324	-	intronic_lncRNA	882	chr11	93907206	93909416	+	2	2.38956	0.52099	0.30935	0.940186	0.725103	0.310169	0.519524	1.42778	0.8511
TCONS_00056646	XLOC_004333	-	lincRNA	781	chr11	94884658	94890810	+	2	1.75215	0.61241	2.42633	0.441663	0.639297	1.9431	0.509791	1.54003	0.700334
TCONS_00049506	XLOC_005032	-	lincRNA	276	chr11	95663339	95683980	-	2	0	1.90519	0.97835	0.833956	0	1.8478	0	1.13346	0
TCONS_00056259	XLOC_005033	-	intronic_lncRNA	2615	chr11	95721820	95724514	-	2	4.69208	1.90169	2.91389	1.40442	2.54433	2.30028	1.64157	1.96294	1.79211

TCONS_00050561	XLOC_005038	-	lincRNA	878	chr11	98365043	98366112	-	2	1.05158	1.67708	0.6224	0	0	0.104002	0.0871073	0	0.256849
TCONS_00058192	XLOC_004343	-	lincRNA	929	chr11	101000702	101003858	+	2	0.41924	0.29229	0.48194	0.0879413	0.169497	0.0967002	0.80911	0.556059	0.397904
TCONS_00053082	XLOC_004344	-	lincRNA	368	chr11	101212719	101253579	+	2	2.40004	0.43288	0.43508	1.1537	0.377071	0	1.11261	1.0063	0.353884
TCONS_00048996	XLOC_004351	-	anti-sense_lncRNA	4676	chr11	102477970	102558005	+	7	0.36506	3.21686	0.03108	1.65786	0.75421	0.0314047	0.376856	1.54101	0.862868
TCONS_00058222	XLOC_004372	-	lincRNA	479	chr11	110351038	110360344	+	2	0.36342	0.25767	0.51372	0.461474	0.224256	0.508243	0.868998	1.78036	0.21052
TCONS_00057873	XLOC_005084	-	lincRNA	5007	chr11	111254569	111262296	-	3	10.9629	6.56279	8.33354	2.31549	3.21742	5.05871	3.88345	1.93517	2.80585
TCONS_00050601	XLOC_005084	-	lincRNA	1111	chr11	111268305	111326680	-	5	2.39406	2.88464	1.38894	0.717119	0.334737	4.13359	1.6992	0.885858	0.86979
TCONS_00050600	XLOC_005084	-	lincRNA	1171	chr11	111268305	111326680	-	4	3.04333	8.6014	2.90448	2.04899	1.47129	0.0009726	3.6079	0.297895	2.44257
TCONS_00046517	XLOC_004403	-	anti-sense_lncRNA	1043	chr11	114387746	114411483	+	5	1.59978	0.99213	0.99353	0.324024	0.732832	0.805947	0.0002473	1.152	0.252731
TCONS_00058242	XLOC_004403	-	anti-sense_lncRNA	1069	chr11	114387942	114458372	+	6	0.01016	0.00089	7.96E-05	0.0008405	0.0006681	0	0.93988	5.55E-05	0
TCONS_00058243	XLOC_004403	-	anti-sense_lncRNA	1152	chr11	114387942	114458372	+	7	0.50124	0.98321	0.81399	0.721894	0.581745	0.766306	1.30293	0	0.628
TCONS_00052745	XLOC_005135	-	lincRNA	3741	chr11	118786227	118796325	-	2	2.65304	2.99086	2.06362	1.89711	2.96507	1.98516	1.49587	1.63171	2.47525
ENST00000526453.1	ENSG00000255121.2	RP11-110I1.12	lincRNA	1359	chr11	118866905	118868714	-	2	1.31993	1.40557	1.50885	0.663135	0.637817	0.4252	1.06211	0.835402	1.34767
TCONS_00056715	XLOC_004436	-	lincRNA	293	chr11	119607387	119610291	+	2	0	0	0	0	0.686888	0	1.38431	0	0
TCONS_00051124	XLOC_004438	-	lincRNA	2822	chr11	119776065	119786458	+	2	0.54511	0.1886	0.37191	0.219644	2.22587	0.643594	0.311166	0.245074	0.15403
TCONS_00055019	XLOC_004439	-	lincRNA	510	chr11	119808785	119810626	+	2	0	0.46291	0.23044	0.207527	1.8128	0.914007	0.194669	0	0
TCONS_00053150	XLOC_004440	-	lincRNA	13299	chr11	119846308	119894308	+	2	0.81109	0.38765	0.46669	2.07009	2.95426	2.03814	1.36081	1.08228	0.22865
TCONS_00053151	XLOC_004441	-	lincRNA	3507	chr11	119929725	119940673	+	3	0.46315	0.8968	0.42096	1.50906	0.612765	0.191317	0.316899	0.533958	0.191827
ENST00000528986.1	ENSG00000255248.2	RP11-166D19.1	lincRNA	3287	chr11	121959818	121971737	-	2	43.9905	49.3907	30.2198	25.9082	19.3374	20.9973	27.128	26.3438	22.6635
TCONS_00047422	XLOC_005165	-	lincRNA	759	chr11	124821166	124823855	-	2	0.72834	0.12735	1.00934	0.803431	0.7755	0.631228	1.59089	0.728029	0.624148
TCONS_00051153	XLOC_004473	-	lincRNA	220	chr11	124984689	124995191	+	2	3.10309	0	0	2.10687	0	0	0	0	0
TCONS_00056736	XLOC_004496	-	lincRNA	242	chr11	128132772	128174926	+	2	0	1.56472	0	0	0	0	2.86883	0	0
TCONS_00047468	XLOC_005205	-	anti-sense_lncRNA	5222	chr11	134129624	134146297	-	2	1.32189	0.85721	1.17704	0.318438	1.49088	1.41342	0.7642	0.814317	0.837814
ENST00000540226.1	ENSG00000256948.1	RP11-598F7.3	intronic_lncRNA	586	chr12	215690	221016	-	2	0.52648	0	0.18403	0.998812	0.161178	0.183211	0.155122	0.849903	2.26989
TCONS_00067221	XLOC_005215	-	lincRNA	513	chr12	799000	812423	+	2	0.97265	0.2292	2.73807	1.64421	0.797826	0.678881	0.57819	1.05413	0
TCONS_00064303	XLOC_005931	-	intronic_lncRNA	473	chr12	2905130	2906130	-	2	1.48535	1.3172	1.05075	0.943368	1.14643	0	0.222237	1.82083	0.21524
TCONS_00067231	XLOC_005230	-	lincRNA	276	chr12	3426008	3427416	+	2	1.2724	0	0.97835	0.833956	0.834464	1.8478	0	0	0
TCONS_00067232	XLOC_005232	-	lincRNA	436	chr12	3528853	3529617	+	2	0	0	0.6108	0	0	0	1.03549	0	0
TCONS_00067234	XLOC_005235	-	lincRNA	233	chr12	3865725	3866645	+	2	0	0	9.72401	0	0	0	0	0	0
TCONS_00065541	XLOC_005937	-	lincRNA	424	chr12	3983968	4000879	-	2	2.2587	0.64482	1.28955	0	0.280682	2.22038	0	0	0
TCONS_00062815	XLOC_005944	-	lincRNA	683	chr12	5539811	5540652	-	2	1.68487	0.44285	0.58555	0.132906	0.128425	0	0	0.168967	0.120583
TCONS_00065015	XLOC_005249	-	lincRNA	2335	chr12	5621174	5632911	+	3	0.86586	1.16197	0.77591	0.052758	0.210535	0.342243	0.327725	0.908724	0.241416
TCONS_00062023	XLOC_005249	-	lincRNA	3508	chr12	5629453	5633177	+	2	1.69428	0.57418	0.65724	0.217394	1.08941	0.416739	0.580916	0.239372	0.245303
TCONS_00072331	XLOC_005253	-	lincRNA	664	chr12	6502628	6503552	+	2	1.75354	0.9224	1.37247	0.415112	1.60498	1.97911	0	0.704112	0.376741
ENST00000537269.1	ENSG00000257084.1	U47924.27	anti-sense_lncRNA	350	chr12	7072409	7073610	+	2	19.4455	16.042	22.5303	4.30936	18.2151	16.1706	25.5235	13.0311	8.74538
TCONS_00063656	XLOC_005269	-	anti-sense_lncRNA	1306	chr12	7091837	7096977	+	3	2.85832	1.79353	2.21442	1.10036	2.00562	1.97348	1.32568	0.802794	1.25562
TCONS_00067267	XLOC_005280	-	lincRNA	1055	chr12	8380220	8388617	+	2	0.94493	0.34955	0.87515	1.02475	0.113895	0.279385	4.51E-06	1.23032	0.248186
ENST00000304751.9	ENSG00000215241.3	RP11-266K4.9	lincRNA	795	chr12	8388011	8391542	+	2	1.21025	0.93117	0.63142	0.574419	0.87604	0.428011	0.497542	3.43E-05	0.130702
TCONS_00070358	XLOC_005283	-	lincRNA	3246	chr12	8929837	8935716	+	3	3.0273	2.06488	3.64811	1.00697	1.31725	0	1.51873	2.11585	0.502758
TCONS_00068238	XLOC_005283	-	lincRNA	1014	chr12	8929846	8935806	+	3	4.40997	0.00077	0.82295	0.0009201	0	0	3.30525	1.79254	0
TCONS_00068237	XLOC_005283	-	lincRNA	1557	chr12	8929846	8935806	+	3	0	1.53193	2.51561	0.404754	0.43997	1.00709	1.86043	1.06244	0
TCONS_00063672	XLOC_005283	-	lincRNA	1102	chr12	8929875	8935920	+	4	1.22458	0	0.46606	0.458661	0.774394	0	0.170516	0	0.279904
TCONS_00063671	XLOC_005283	-	lincRNA	1180	chr12	8929875	8935920	+	3	2.65441	3.65126	1.24711	1.32328	2.80896	1.34942	2.41268	2.65979	1.75208
TCONS_00062080	XLOC_005283	-	lincRNA	3874	chr12	8929875	8935920	+	2	13.9739	8.68415	13.3844	6.77829	6.34159	7.53989	9.92215	7.14596	8.30897
TCONS_00067269	XLOC_005292	-	lincRNA	3470	chr12	9393145	9397024	+	2	2.27948	1.92189	3.93803	2.23052	2.40373	1.44037	2.52837	2.30734	1.64019
TCONS_00062084	XLOC_005292	-	lincRNA	2210	chr12	9397373	9410728	+	4	0.68667	0.882	1.77883	0.847687	1.10326	1.20286	0.901271	1.24379	1.44127
TCONS_00063676	XLOC_005292	-	lincRNA	906	chr12	9398926	9408744	+	3	1.22318	0.39449	1.9635	1.75987	0.343757	0.561683	0.665266	1.02351	1.3004
ENST00000541404.1	ENSG00000256582.1	RP11-75L1.1	lincRNA	560	chr12	9856673	9861946	+	2	0.56428	0.19882	0.59295	0.535663	0.173004	0.196539	0.666841	1.14113	0.812123
ENST00000544591.1	ENSG00000256039.1	RP11-291B21.2	lincRNA	868	chr12	10705962	10710648	+	2	3.681	0.71803	20.2255	1.29007	0.462768	0.633391	11.4302	10.4721	5.22983
TCONS_00078227	XLOC_005318	-	lincRNA	1576	chr12	10709204	10727469	+	3	0.87909	0.06437	3.80484	0.0726397	0	0	1.86074	0.926342	0.916823
TCONS_00068266	XLOC_005318	-	lincRNA	1840	chr12	10725590	10729031	+	2	0.93331	0.21564	2.55344	0.195175	0.0375087	0	1.24716	0.883478	0.774944
ENST00000538329.1	ENSG00000256084.1	RP11-134N1.2	lincRNA	640	chr12	14369524	14374231	+	2	0.69327	0.32445	0.48295	0.145951	0.423418	0.160592	0.271102	0.743348	0.132517
TCONS_00065085	XLOC_005338	-	anti-sense_lncRNA	1123	chr12	14765816	14819420	+	5	1.93501	1.6971	0.97075	0.258758	0.746123	0.873977	0.774488	0.874316	0.353202
TCONS_00075155	XLOC_005338	-	anti-sense_lncRNA	619	chr12	14767456	14813966	+	4	3.49581	2.57832	2.56543	1.28029	1.22502	1.05185	2.93433	1.40082	1.29707
TCONS_00075154	XLOC_005338	-	anti-sense_lncRNA	4282	chr12	14767456	14776682	+	3	3.57927	2.29625	3.01759	1.47193	2.09898	1.79616	1.85649	1.92295	1.80859
TCONS_00067297	XLOC_005342	-	lincRNA	355	chr12	15154634	15158080	+	3	0	0.94018	0.47329	0	1.22876	0	0	0	0
TCONS_00068313	XLOC_005368	-	lincRNA	2178	chr12	19802412	19806074	+	4	0.44371	0.06632	0.3301	0.224564	1.11781	1.07109	0.630599	0.280733	0.149567
TCONS_00078233	XLOC_005368	-	lincRNA	1192	chr12	19802662	19804840	+	3	0.64342	0.36002	0.5938	0.387559	0.341813	0.622102	0.792802	0.341623	1.19397
TCONS_00069543	XLOC_005368	-	lincRNA	2073	chr12	19803106	19806074	+	2	0.77051	0.49472	1.05109	0.822196	1.29788	1.24367	0.599619	1.84239	0.937796
TCONS_00068314	XLOC_005368	-	lincRNA	465	chr12	19823824	19825652	+	2	0.38245	0.27155	1.08352	0.24301	0.236354	1.87371	0.229255	1.25182	0.887492
TCONS_00067312	XLOC_005368	-	lincRNA	824	chr12	20076081	20098026	+	2	2.04709	0.1521	1.37193	0.719487	0.793103	0.580441	0.131345	0.8955	0

TCONS_00073449	XLOC_005368	-	lincRNA	436	chr12	20088546	20097526	+	2	0.62427	1.73169	0.5624	0	4.05E-05	2.06577	0.935669	0.04326	0.499532
TCONS_00062924	XLOC_006049	-	lincRNA	1598	chr12	22506138	22508106	-	2	0.72975	0.25312	0.89947	0.229006	0.440278	0.251695	0.585972	1.21034	0.826931
TCONS_00075174	XLOC_005387	-	lincRNA	8788	chr12	22852535	23217671	+	11	2.7551	0	2.81754	0.22294	2.69524	2.4271	2.08456	1.02703	1.37042
TCONS_00070450	XLOC_005387	-	lincRNA	11746	chr12	22852725	23221060	+	9	0.00055	0.00703	0.0005	0.092229	1.05652	0.546946	0	0.767844	0.0008512
TCONS_00068332	XLOC_005387	-	lincRNA	5113	chr12	22852740	23203329	+	6	6.72358	1.64818	4.02352	0.840948	4.19512	4.24112	1.90511	1.45187	1.7503
TCONS_00069557	XLOC_005387	-	lincRNA	8337	chr12	22852895	23217665	+	10	5.53478	1.37835	2.63126	1.03918	3.70175	3.54932	0.0301308	1.8024	1.31625
ENST00000537293.1	ENSG00000256923.1	RP11-449P1.1	lincRNA	424	chr12	22895516	22896025	+	2	7.22783	3.86894	4.8358	1.72701	6.175	7.61274	3.00829	2.60811	3.68852
TCONS_00075907	XLOC_005387	-	lincRNA	12066	chr12	22940187	23221060	+	10	8.13548	1.51042	2.93547	0.860293	4.67196	5.30497	1.34403	0.697042	2.07007
TCONS_00073457	XLOC_005389	-	intronic_lncRNA	1134	chr12	24972483	24975448	+	2	0.9823	0.07596	0.22509	0.205834	0.396203	0.527916	0.50336	1.73134	0.372126
TCONS_00062936	XLOC_006061	-	intronic_lncRNA	3825	chr12	25731901	25737682	-	2	6.11281	1.57759	3.78181	0.405923	1.43966	1.41557	1.83038	1.35031	2.09968
TCONS_00071112	XLOC_006066	-	intronic_lncRNA	1815	chr12	26759515	26769960	-	2	1.70593	2.23371	1.90166	2.29911	2.1331	3.09306	1.01329	1.74462	2.32517
TCONS_00072039	XLOC_006087	-	lincRNA	420	chr12	31516331	31522637	-	2	0	0.32841	0.32848	0	0.571821	0	2.22977	0.379639	0
ENST00000535163.1	ENSG00000255760.1	RP11-428G5.5	lincRNA	852	chr12	32030013	32040137	-	2	0	0.21806	2.59021	1.377	0.37938	1.29799	1.08776	2.61525	3.56238
TCONS_00067360	XLOC_005437	-	lincRNA	628	chr12	43240695	43243569	+	2	0.71261	1.00098	1.32474	0.300122	0.435449	0.495367	3.34707	1.14698	0
TCONS_00065152	XLOC_005437	-	lincRNA	2048	chr12	43460122	43539392	+	2	1.15974	1.33909	0.30196	0.138548	0.232916	0.418702	2.62249	1.78472	0.187489
TCONS_00066251	XLOC_005437	-	lincRNA	736	chr12	43541591	43542502	+	2	0.18987	1.19584	0.52669	0	0.11559	0.526757	2.54645	1.21571	0.21707
TCONS_00070003	XLOC_006117	-	lincRNA	1020	chr12	45870542	45875923	-	2	0.24856	0.34618	1.0269	1.09423	0.828058	1.71869	0.430788	1.18474	0.565513
ENST00000551021.1	ENSG00000257261.1	RP11-96H19.1	lincRNA	281	chr12	46777890	46781755	+	2	44.4789	15.2549	20.234	16.5132	21.9935	40.0574	31.0487	44.7465	25.0326
ENST00000547626.1	ENSG00000247774.2	PCED1B-AS1	lincRNA	420	chr12	47599681	47603157	-	3	1.83946	2.62727	5.91257	5.27593	3.14502	2.58421	4.45955	8.73169	8.05115
TCONS_00067374	XLOC_005449	-	anti-sense_lncRNA	587	chr12	48099879	48113448	+	2	1.05026	1.6628	2.93666	0.996219	0.482267	1.09641	2.01121	1.27148	1.35836
TCONS_00069601	XLOC_005450	-	lincRNA	760	chr12	48153646	48166173	+	2	2.36288	1.14408	1.76314	0.572851	0.442344	2.39436	0.952792	0.872049	0.311511
TCONS_00067885	XLOC_006153	-	anti-sense_lncRNA	262	chr12	49691363	49697017	-	2	4.54546	1.14448	9.46622	1.99869	2.01048	0	4.1189	1.37142	0
TCONS_00065732	XLOC_006155	-	intronic_lncRNA	305	chr12	49945694	49946613	-	2	2.83751	0	0	0	0	1.35742	0	0	0
TCONS_00067891	XLOC_006165	-	anti-sense_lncRNA	708	chr12	50344952	50345908	-	2	0.20029	0.84164	0.83434	0.505366	1.88316	0.416955	0.81876	0.48149	0.229167
TCONS_00065740	XLOC_006165	-	anti-sense_lncRNA	266	chr12	50345713	50348023	-	2	0	3.24952	0	0	2.44155	1.04946	1.94282	2.59027	0
ENST00000550214.1	ENSG00000257588.1	RP11-469H8.6	anti-sense_lncRNA	1031	chr12	50352655	50356707	-	2	3.80178	3.58645	6.24782	1.31108	7.42747	4.57885	2.97501	3.70144	2.30166
ENST00000552576.1	ENSG00000258056.1	RP11-644F5.11	anti-sense_lncRNA	1274	chr12	56122888	56124467	+	2	3.70289	6.46533	5.86528	4.71175	4.13139	4.9827	5.29864	4.28496	4.31076
TCONS_00067431	XLOC_005550	-	lincRNA	2854	chr12	56403521	56414145	+	3	1.15402	0.87835	0.99726	0.50632	0.972179	0.529856	0.28544	0.635534	0.347815
TCONS_00062360	XLOC_005561	-	lincRNA	352	chr12	56914086	56914554	+	2	3.30951	0	0.48306	0	0	0.4693	0	1.11752	0
TCONS_00064686	XLOC_006282	-	lincRNA	1010	chr12	65277024	65298064	-	3	3.14523	0.35048	0.17329	0.158253	0.152428	0.695992	0.290791	0.499806	0.286271
TCONS_00065852	XLOC_006285	-	intronic_lncRNA	924	chr12	66270737	66275765	-	2	0.70355	1.17729	0.38824	0.177101	0.597369	0	0	0.783928	0.320538
TCONS_00066379	XLOC_005605	-	lincRNA	3836	chr12	68072646	68078740	+	2	2.27484	1.98057	2.4689	1.12611	1.72236	1.25662	1.66474	2.89105	2.12503
ENST00000538665.1	ENSG00000255733.1	IFNG-AS1	lincRNA	1791	chr12	68383225	68415107	+	4	3.59152	4.84627	10.9688	9.09391	3.32543	12.515	7.45803	19.8362	12.3466
TCONS_00075645	XLOC_006291	-	lincRNA	286	chr12	68489657	68512329	-	2	0	0	0.86709	0	0	0	6.74653	3.01289	0
TCONS_00069686	XLOC_005616	-	lincRNA	419	chr12	69853108	69854530	+	2	0.46194	0	0.66007	0.588911	0.287248	1.29807	0	0.381443	0
TCONS_00067483	XLOC_005620	-	lincRNA	1365	chr12	70218339	70249419	+	2	2.10103	1.09432	2.04138	0.219905	0.423011	2.23588	1.4591	1.03883	1.98622
ENST00000552998.1	ENSG00000257613.1	RP11-320P7.1	lincRNA	629	chr12	70612912	70615642	-	2	3.08083	1.8308	1.48678	0.598838	2.60652	1.64735	2.36515	1.90708	2.17536
TCONS_00066991	XLOC_006309	-	anti-sense_lncRNA	1525	chr12	75727270	75730109	-	2	3.91493	2.03024	2.61098	0.968209	1.56733	1.54995	1.6086	2.15897	1.81631
TCONS_00071303	XLOC_006309	-	anti-sense_lncRNA	2535	chr12	75727270	75784700	-	4	0.72757	2.29242	1.23986	0.564132	1.18224	0.559554	1.48384	1.53701	0.589528
TCONS_00064721	XLOC_006309	-	anti-sense_lncRNA	1649	chr12	75753315	75784721	-	3	3.53875	3.27048	0.82487	0.411867	0.0005165	0.971941	2.01133	0.0007054	1.72721
TCONS_00064720	XLOC_006309	-	anti-sense_lncRNA	1606	chr12	75753351	75784774	-	2	3.36396	2.48506	5.20984	2.63892	3.808	2.30171	2.54754	5.5006	1.53229
TCONS_00064722	XLOC_006309	-	lincRNA	4357	chr12	75779890	75784702	-	2	7.82144	6.26003	7.07938	2.64281	4.11989	4.04103	3.76414	4.45758	4.22181
TCONS_00065315	XLOC_005634	-	lincRNA	625	chr12	76953753	76956438	+	2	0.47841	0.67209	0.33357	0.604499	0.73094	1.33036	0.98332	1.15526	0.960794
ENST00000550042.1	ENSG00000231121.2	RP1-34H18.1	lincRNA	991	chr12	77718421	77966055	+	2	4.25116	6.19203	6.30106	1.7827	2.88814	2.85106	4.6172	4.09563	1.90578
ENST00000550268.1	ENSG00000257894.1	RP1-78O14.1	anti-sense_lncRNA	1155	chr12	79786697	79849240	-	2	1.17425	1.70753	1.17385	0.603801	2.32463	1.84384	3.38353	0.930978	0.606381
TCONS_00072171	XLOC_006326	-	intronic_lncRNA	1090	chr12	79802644	79804182	-	2	0.91622	0.63757	0.63022	0.647992	1.03979	0.633248	0.925028	0.636166	0.130189
TCONS_00065323	XLOC_005640	-	anti-sense_lncRNA	1125	chr12	80329029	80334856	+	2	1.87347	2.29988	2.27304	0.554154	1.60114	1.4469	3.74921	2.01036	1.1271
TCONS_00063280	XLOC_006329	-	anti-sense_lncRNA	1324	chr12	80848469	80852600	-	3	1.54241	0.63024	3.61038	1.1397	3.45334	5.07373	0.208675	1.07704	0.463281
TCONS_00062490	XLOC_005655	-	anti-sense_lncRNA	1340	chr12	81653343	81657188	+	2	3.48891	2.54754	4.41846	0.168551	1.24296	0.679333	0.977151	1.27416	0.558257
TCONS_00070151	XLOC_006346	-	lincRNA	298	chr12	89489327	89588356	-	4	0	0	0	4.59435	2.60898	2.17798	0	0	0
ENST00000605233.1	ENSG00000270344.1	RP11-734K2.4	anti-sense_lncRNA	338	chr12	89919431	89934079	+	2	8.00103	4.23452	6.41221	4.68457	3.69123	1.03443	3.20083	3.09117	4.7624
TCONS_00070755	XLOC_005672	-	anti-sense_lncRNA	2252	chr12	89954071	89988364	+	2	2.18915	0.93586	1.94879	1.0366	1.34225	1.80404	1.40358	2.76716	1.42438
ENST00000552778.1	ENSG00000258302.1	RP11-981P6.1	anti-sense_lncRNA	854	chr12	89954906	89987798	+	2	2.1784	0.63404	2.54394	0.0750427	1.53143	0.902329	2.85951	0.171289	0.831915
TCONS_00076970	XLOC_005673	-	lincRNA	415	chr12	90460844	90494747	+	3	1.41132	0.33623	0	0	0	0	0.856681	1.94418	0
ENST00000499685.2	ENSG00000245904.2	RP11-796E2.4	lincRNA	5699	chr12	92539957	92583436	+	3	0.53863	0.24382	0.73337	0.418761	0.613757	0.370615	0.1903	0.568605	0.524018
TCONS_00068025	XLOC_006359	-	lincRNA	768	chr12	92751076	92757759	-	2	0.71684	0.37594	1.24137	0.338853	0.763089	0.62118	0.521712	1.71912	0.511801
TCONS_00073620	XLOC_005677	-	lincRNA	858	chr12	92950804	92977315	+	2	0.33532	0	0.47972	0.180392	5.24E-06	0.154478	0.252527	0.698698	1.11971
TCONS_00067519	XLOC_005677	-	lincRNA	1112	chr12	92950804	93023381	+	2	0	0.43212	0.29494	0.139605	0.0939652	5.20E-06	0.546219	0.384944	1.88577
TCONS_00065893	XLOC_006360	-	lincRNA	296	chr12	93368826	93370166	-	2	2.05773	2.28548	0	0.669507	0	0	1.33981	0	0
TCONS_00065904	XLOC_006375	-	lincRNA	1374	chr12	96251024	96252559	-	2	1.73746	1.08589	1.01281	0.218216	0.419752	0.23986	0.748859	0.962083	0.788367
TCONS_00062536	XLOC_005694	-	lincRNA	16273	chr12	97856282	97872833	+	2	3.65475	0.68006	2.06133	13.9736	4.75765	5.6123	11.4422	5.65017	1.92061

TCONS_00067562	XLOC_005719	-	lincRNA	271	chr12	102698417	102709241	+	2	0	0	1.04393	0	1.77836	0	0	2.41919	0.833167
TCONS_00067580	XLOC_005739	-	lincRNA	315	chr12	105789372	105791090	+	2	0	0	1.93735	0.561452	0.555566	0.620469	2.21908	0.747476	0
TCONS_00071845	XLOC_005742	-	lincRNA	829	chr12	106155981	106157314	+	2	0.32378	0.22611	0	0.305893	1.67186	2.57917	1.03428	0.904107	0.738761
TCONS_00063384	XLOC_006412	-	lincRNA	2109	chr12	106404369	106453538	-	2	1.22965	1.66652	2.1189	1.47528	2.12568	1.62122	0.672765	2.14873	1.54258
TCONS_00070847	XLOC_005757	-	lincRNA	1130	chr12	109028967	109030444	+	2	0.21923	0.53374	0.67821	11.7123	1.19375	4.31729	0.568748	1.91251	2.5534
TCONS_00064168	XLOC_005770	-	anti-sense_lncRNA	405	chr12	110418757	110421591	+	2	2.46602	1.41209	3.53503	0.314988	0.922125	0.693943	5.4052	2.86038	14.137
TCONS_00063456	XLOC_006471	-	lincRNA	838	chr12	115765288	115787820	-	4	2.07497	0.78012	0.77229	0.603131	0.581669	0.773845	0.0926738	0.509237	0.364124
TCONS_00078133	XLOC_006473	-	lincRNA	712	chr12	115946028	116201272	-	2	2.30221	1.00689	1.20389	2.54738	1.12877	0.72816	0.193686	1.39032	0.562027
TCONS_00075737	XLOC_006473	-	lincRNA	4288	chr12	116171718	116201257	-	2	11.4766	1.60182	4.25916	4.20455	6.01666	3.24478	1.84064	2.55629	0.734126
TCONS_00065988	XLOC_006494	-	lincRNA	458	chr12	118555546	118573787	-	3	4.71288	3.34884	2.22784	1.99719	3.15777	0.825016	0.707303	0.32175	0.456041
TCONS_00065458	XLOC_005833	-	lincRNA	276	chr12	122115091	122127014	+	2	0	2.85779	0.97835	0.833956	0.834464	1.8478	0	0	0
TCONS_00062744	XLOC_005859	-	anti-sense_lncRNA	1122	chr12	124427894	124430006	+	2	3.2063	0.61531	0.76017	0.208485	0.334493	0.84041	0.828748	0.613861	1.06797
TCONS_00066046	XLOC_006542	-	intronic_lncRNA	321	chr12	124558259	124560407	-	2	0	1.81465	0.61268	0	0	0	0	0	0
TCONS_00069851	XLOC_005861	-	intronic_lncRNA	307	chr12	124665443	124683619	+	2	0	0	0.69552	0.602634	0	0	0	2.41554	0
TCONS_00065499	XLOC_005869	-	intronic_lncRNA	483	chr12	125999765	126000504	+	2	3.22487	0.50791	0	0.227455	0.66307	0	0.428093	1.16958	1.03743
TCONS_00067684	XLOC_005882	-	intronic_lncRNA	4283	chr12	128867163	128871500	+	2	1.05048	1.00275	3.22036	0.0470046	0.0451039	0.0344294	0.0427486	0.275064	0.0705996
TCONS_00085982	XLOC_006859	-	lincRNA	1025	chr13	19778616	19783565	-	3	4.69406	1.80635	2.38142	1.70907	1.57119	2.98943	1.42713	2.74744	3.09134
TCONS_00081814	XLOC_006593	-	lincRNA	2037	chr13	20437904	20527251	+	3	2.05557	1.77052	2.27829	1.81191	2.54399	1.56997	1.84363	1.92684	1.57179
TCONS_00079749	XLOC_006603	-	lincRNA	861	chr13	22386822	22388382	+	2	2.31116	0.32261	0.42575	0.0970141	0.561254	0	0.0893892	0.491264	0
ENST00000439928.2	ENSG00000228741.2	RP11-309I15.1	lincRNA	1721	chr13	24553949	24609166	+	5	5.46936	3.79306	3.05647	0.667059	1.12285	1.08901	1.07902	0.759146	2.34544
TCONS_00084423	XLOC_006611	-	lincRNA	1055	chr13	24553965	24608365	+	4	3.03581	1.53136	2.8236	0.910875	1.02806	1.93321	1.99102	1.29299	2.72888
TCONS_00082000	XLOC_006892	-	lincRNA	883	chr13	27819764	27825415	-	2	9.09691	5.09814	8.9578	3.94298	5.15873	5.57484	8.04061	9.02921	7.73357
TCONS_00080937	XLOC_006644	-	lincRNA	746	chr13	30221828	30229918	+	2	1.86403	1.43454	1.42142	0.822626	0	1.03413	0.651849	1.64046	2.02401
TCONS_00082944	XLOC_006914	-	lincRNA	370	chr13	34238014	34250923	-	2	1.18614	4.27661	4.29733	1.52006	2.6076	5.03021	5.85968	1.49086	2.79689
TCONS_00085334	XLOC_006918	-	lincRNA	685	chr13	36735787	36738413	-	2	0.20974	0.441	0	0	0.127889	0	0.367962	0.673028	1.08072
TCONS_00081870	XLOC_006669	-	lincRNA	425	chr13	37848157	37938461	+	3	0.00214	0	2.56719	1.71926	2.23492	0.0018079	0.0019822	1.85424	0.0035647
TCONS_00083624	XLOC_006669	-	lincRNA	1244	chr13	37880730	37925065	+	2	0.39067	0.13576	0.20118	0.613596	1.00365	0.809407	0	0.154709	0.0554412
TCONS_00084725	XLOC_006669	-	lincRNA	416	chr13	37881456	37938450	+	2	0.46606	0	0	0	0.0003266	4.60512	1.1346	8.46E-05	0.269746
TCONS_00079822	XLOC_006671	-	anti-sense_lncRNA	5405	chr13	38205904	38216518	+	2	1.56977	0.81348	0.82831	0.884846	1.28513	1.37721	0.569709	0.60078	0.442919
TCONS_00084988	XLOC_006927	-	lincRNA	1667	chr13	38627950	38629703	-	2	1.25185	0.96467	1.71382	0.916526	1.55211	2.68616	1.23598	1.64722	1.69396
TCONS_00083628	XLOC_006677	-	lincRNA	2822	chr13	40746442	40766339	+	5	0.40066	0	0.34554	0.0976195	1.32597	2.78E-06	0.259319	0.932226	3.88304
TCONS_00080971	XLOC_006677	-	lincRNA	2669	chr13	40746513	40765821	+	5	0.49485	2.44602	0.28197	0	1.10369	0.142397	0.397222	0.916391	0.896028
TCONS_00080972	XLOC_006677	-	lincRNA	1517	chr13	40754864	40763406	+	4	0.83201	2.17798	0.63678	0.0486226	0.937614	0.160323	0.77831	1.67745	3.34492
TCONS_00080974	XLOC_006680	-	lincRNA	1970	chr13	41707083	41751147	+	4	2.73293	1.71997	2.00923	0.753416	1.74084	1.38356	1.25576	1.61159	1.06554
TCONS_00080437	XLOC_006680	-	lincRNA	1245	chr13	41707088	41737458	+	3	1.52213	0.94585	0.87259	0.807157	1.05555	1.09084	0.390002	0.119614	0.683721
TCONS_00085224	XLOC_006685	-	lincRNA	827	chr13	42062513	42066930	+	3	0.32481	0.45367	0.44916	0.204581	0	0	0.188663	0.907035	0.926423
TCONS_00081883	XLOC_006685	-	lincRNA	415	chr13	42062517	42080479	+	2	0	0.33623	0.67283	0.899925	0	0	0.856681	0.777672	2.47289
TCONS_00081181	XLOC_006945	-	lincRNA	3392	chr13	43313376	43321686	-	3	18.5217	6.79732	9.72448	1.34133	2.60372	3.69538	3.91811	1.9499	2.90558
TCONS_00082561	XLOC_006945	-	lincRNA	4801	chr13	43313376	43321686	-	5	0	0.83664	1.06467	2.83E-05	0.607148	0	0.346063	0	1.06007
TCONS_00082559	XLOC_006945	-	lincRNA	3219	chr13	43313377	43321591	-	4	2.43701	0.28265	1.23414	0.470722	1.32757	0.0003526	0.950746	1.25341	0.0003766
TCONS_00082560	XLOC_006945	-	lincRNA	3226	chr13	43313377	43321591	-	4	2.71655	2.12354	3.1379	0.196933	0	1.14005	0.708199	9.01E-05	0.750788
TCONS_00081893	XLOC_006697	-	lincRNA	5183	chr13	44495494	44521419	+	2	5.73886	3.68231	3.90769	0.616188	1.52719	1.4668	3.956	2.17245	3.14618
TCONS_00080459	XLOC_006697	-	lincRNA	1752	chr13	44612467	44620660	+	4	5.86058	2.35018	2.91006	0.672179	0.906363	0.772477	2.50444	1.67047	2.03883
TCONS_00081896	XLOC_006697	-	lincRNA	1492	chr13	44612483	44617434	+	2	5.24932	2.63336	1.99904	0.547892	0.903542	1.27461	1.54665	1.48895	1.82333
TCONS_00081895	XLOC_006697	-	lincRNA	2735	chr13	44612483	44617434	+	3	0.04023	0.10683	0.51736	2.43E-05	0.279078	0.0004768	0.645734	0.113364	1.7959
TCONS_00079878	XLOC_006712	-	intronic_lncRNA	3633	chr13	46825416	46831907	+	3	3.71801	1.29574	2.16925	0.354092	0.482922	0.286673	1.28873	0.748083	0.722301
TCONS_00082976	XLOC_006966	-	lincRNA	537	chr13	46887219	46891482	-	2	0.60255	0.42522	0.95701	0.724779	0	0.285093	0.644442	1.43194	0
TCONS_00082059	XLOC_006966	-	lincRNA	994	chr13	46890480	46895857	-	2	0.64169	0.62579	0.83767	0.177719	0.155521	0.50084	0.399842	0.217988	1.16832
TCONS_00082846	XLOC_006718	-	lincRNA	225	chr13	49155332	49173591	+	2	0	0	0	1.88777	0	0	0	0	1.80487
TCONS_00081904	XLOC_006719	-	lincRNA	506	chr13	49227869	49267727	+	3	0.6171	0.00044	1.30066	1.37289	1.9465	0.940731	0.34937	0.0004486	1.95777
TCONS_00083653	XLOC_006719	-	lincRNA	529	chr13	49227869	49267727	+	3	0	0.46859	0	7.14379	1.78244	1.3031	0.807209	1.0785	0.0001177
TCONS_00082069	XLOC_006987	-	lincRNA	233	chr13	50948754	50961192	-	2	2.39078	0	3.88961	0	1.63625	0	0	2.25692	1.52997
TCONS_00081918	XLOC_006727	-	lincRNA	287	chr13	50964225	50989308	+	2	1.12673	1.67647	3.42885	0.7354	0	0.814037	2.22267	0.992828	0.687349
TCONS_00081919	XLOC_006731	-	intronic_lncRNA	1259	chr13	51289401	51338990	+	3	1.2347	0.86986	1.3701	0.544399	0.477567	0.411371	0.567036	1.74016	1.28722
TCONS_00079905	XLOC_006731	-	intronic_lncRNA	646	chr13	51331826	51382958	+	3	1.81646	0.71439	0.5196	0	0.841132	0.287262	0.721317	0.438605	0.414456
TCONS_00079906	XLOC_006731	-	intronic_lncRNA	1033	chr13	51382032	51383617	+	2	0.75975	0.81228	0.92648	0.692433	0.500299	0.338357	1.01541	1.53381	1.27162
ENST00000601286.1	ENSG00000233672.2	RNASEH2B-AS1	lincRNA	657	chr13	51456688	51479292	-	3	0.22254	0.15612	0.61951	0.561996	0.271646	1.08209	0.391117	0.893902	0.637637
TCONS_00082084	XLOC_007001	-	lincRNA	284	chr13	53171335	53174540	-	2	1.1633	0	2.66244	0	1.51703	0	0.767624	0	0.711041
TCONS_00084037	XLOC_006761	-	lincRNA	5365	chr13	74184978	74210526	+	3	0.63277	0.49187	0.44429	0.173386	0.855477	0.353773	2.0376	2.09576	1.19399
TCONS_00081039	XLOC_006763	-	lincRNA	1604	chr13	74861940	74981226	+	5	0	1.53793	1.03829	0	0.263317	0.0936955	0.192523	0	0.33272
TCONS_00081038	XLOC_006763	-	lincRNA	1643	chr13	74861940	74981226	+	6	0.37713	1.39307	0.00017	0	0.242974	0	0	0	4.76E-05

TCONS_00085261	XLOC_006763	-	lincRNA	1347	chr13	74862009	74981149	+	5	2.66613	0.75864	1.45624	0.273716	1.03427	0	1.42937	0.654738	0
TCONS_00080533	XLOC_006763	-	lincRNA	417	chr13	74862189	74906337	+	2	0	0	1.20771	0.0070968	0	0.327539	0	1.45653	1.08869
TCONS_00081468	XLOC_006763	-	lincRNA	2852	chr13	74862197	74982904	+	4	0.01486	0.00744	1.14735	0	0.0016218	0.70319	0.230949	0.658807	1.36683
TCONS_00079934	XLOC_006763	-	lincRNA	3002	chr13	74930154	74983084	+	3	0.5895	0.9052	0	0	0	5.17E-05	0	4.96E-05	0
TCONS_00081929	XLOC_006763	-	lincRNA	2687	chr13	74973382	74982904	+	3	2.623	2.17627	3.90777	0.0004992	1.11099	0.0671606	1.70877	1.07108	0.0815243
TCONS_00079935	XLOC_006763	-	lincRNA	3083	chr13	74973856	74983084	+	2	1.67211	0	0.27628	0.422908	0	0.176329	0	0	0.431676
TCONS_00082662	XLOC_007016	-	anti-sense_lncRNA	1556	chr13	76191609	76209950	-	2	2.18171	2.24449	2.47337	1.32221	1.45271	1.24563	1.20857	1.01036	0.895282
TCONS_00083824	XLOC_007022	-	lincRNA	297	chr13	77548016	77550877	-	2	0	0	0	0	1.31782	0	0	0.890022	0
TCONS_00080835	XLOC_007024	-	intronic_lncRNA	1968	chr13	77863245	77866665	-	2	1.78936	1.31988	3.03848	1.37582	1.77399	2.70563	1.28839	2.82161	3.23399
TCONS_00081246	XLOC_007034	-	lincRNA	883	chr13	80766730	80792753	-	6	3.22006	1.7583	3.13198	0.38852	0.426913	0.31042	1.50086	0.435048	1.16244
TCONS_00081245	XLOC_007034	-	lincRNA	980	chr13	80766730	80792753	-	5	0.02079	0.69839	0	0.0001925	0.851322	0.0002279	1.7347	0.0002105	0
TCONS_00086618	XLOC_007034	-	lincRNA	1506	chr13	80773784	80786906	-	4	3.20377	1.52845	2.07096	0.148514	0.888362	0.281216	0.809867	0.456118	1.83122
TCONS_00081762	XLOC_007034	-	lincRNA	839	chr13	80774098	80792861	-	4	2.58069	0.30828	1.48776	0.626393	0.421712	0.810615	0.0001917	1.07019	0
TCONS_00081763	XLOC_007034	-	lincRNA	1092	chr13	80807155	80810304	-	2	1.25699	0.63613	1.88637	0.215512	0.553303	0.39489	1.31846	1.0881	0.389686
TCONS_00081934	XLOC_006775	-	lincRNA	277	chr13	81388670	81398019	+	2	1.25776	1.88213	2.89832	1.64804	0.824236	0.912821	1.67429	2.23847	3.8624
ENST00000438290.2	ENSG00000227640.2	SOX21-AS1	anti-sense_lncRNA	3287	chr13	95364970	95368500	+	2	5.9536	1.25822	6.06652	1.90684	6.58505	3.66655	4.20691	4.525	3.75545
TCONS_00080250	XLOC_007046	-	lincRNA	545	chr13	95468725	95598264	-	3	3.53198	0.62281	0	0	0.180651	0	0.174218	0.953683	0.678403
TCONS_00084625	XLOC_007048	-	intronic_lncRNA	1705	chr13	96147992	96186172	-	3	0.81353	0.32924	0	0	0.408965	4.48837	0.272052	0.695659	0.460753
TCONS_00080565	XLOC_006800	-	lincRNA	1748	chr13	99229507	99231788	+	2	3.29567	1.78232	2.07464	1.19937	1.94755	0.863606	1.73731	1.5605	1.41838
TCONS_00081266	XLOC_007060	-	lincRNA	1632	chr13	100146731	100148679	-	2	2.49357	0.74125	4.24359	0.849533	1.24637	1.86738	2.20606	2.86916	3.14816
TCONS_00081060	XLOC_006805	-	lincRNA	539	chr13	100152765	100153537	+	2	0	0.63401	0	0.379353	1.10341	0.208806	0	0.728275	0.517956
TCONS_00086074	XLOC_007073	-	lincRNA	330	chr13	103931352	103931996	-	2	0.77079	0	0	0	0	0	0	0	0.92033
TCONS_00081526	XLOC_006821	-	lincRNA	2703	chr13	108895675	108905451	+	2	3.26289	1.89113	2.2266	1.89189	1.69372	3.03401	2.04934	3.14541	1.75201
TCONS_00083850	XLOC_007080	-	lincRNA	387	chr13	110212491	110223460	-	2	0	0	0.38903	0	1.01333	0.380696	1.65488	0.449785	0
TCONS_00081968	XLOC_006851	-	lincRNA	474	chr13	114518962	114519663	+	3	0.73999	1.04984	1.30849	1.40985	0.913728	1.55281	0.22139	0.906978	1.0722
TCONS_00080015	XLOC_006854	-	lincRNA	1778	chr13	114973938	114988307	+	3	3.16844	2.01729	3.36214	0.567986	1.01367	1.96185	2.37082	1.98986	2.48973
TCONS_00095028	XLOC_007110	-	lincRNA	562	chr14	20905875	20907382	+	2	0	0.39542	0	0.177563	0	0	0.994547	0.907727	0.323027
TCONS_00093117	XLOC_007585	-	lincRNA	467	chr14	21465930	21467355	-	2	0.37961	0.80843	0.80636	0.482355	1.17273	0.265649	0	0.310528	1.10088
TCONS_00088992	XLOC_007137	-	lincRNA	464	chr14	22265410	22266000	+	2	0.38389	2.99861	0.27194	1.21968	0.474537	0	0.230163	0.628362	1.78185
TCONS_00089639	XLOC_007141	-	lincRNA	1598	chr14	22319778	22323206	+	5	0.43785	1.36683	0.8495	0.82442	0.616389	0.302034	0.418552	2.30542	1.48848
TCONS_00090361	XLOC_007145	-	lincRNA	453	chr14	22371012	22371572	+	2	0	0.8541	0.28417	0.763852	0.991246	0.280493	0.24065	0.328341	1.39574
TCONS_00087175	XLOC_007140	-	lincRNA	1251	chr14	22445778	22447129	+	2	0.67914	0.33712	0.26643	0.18285	0.117286	0.134	0.167503	0.230504	0.93619
TCONS_00096046	XLOC_007163	-	lincRNA	427	chr14	22670471	22671481	+	2	0	0.31806	0.31797	0	0.276884	0	0	0	1.29951
TCONS_00090639	XLOC_007595	-	lincRNA	237	chr14	23025206	23025711	-	2	0	1.71126	3.59564	0	0	0	0	2.08594	0
TCONS_00092887	XLOC_007178	-	lincRNA	644	chr14	23451909	23452977	+	2	1.60313	0.80372	0.47851	0.433891	1.25866	0.477411	0.805774	0.368254	0.656542
TCONS_00089021	XLOC_007187	-	lincRNA	526	chr14	24058510	24088733	+	3	1.86824	2.85877	1.53178	0.986417	0.191364	0.868796	0.554265	0.505439	0
TCONS_00093357	XLOC_007210	-	anti-sense_lncRNA	543	chr14	24914735	24977661	+	3	0.88811	0.6265	3.32318	0.374905	1.45377	0.206353	0.70108	0.479699	2.72969
TCONS_00096660	XLOC_007657	-	lincRNA	2486	chr14	28971027	29082977	-	5	0.8551	0.56585	1.63573	0.766574	1.32202	2.55775	2.60973	2.63217	2.16774
TCONS_00095793	XLOC_007657	-	lincRNA	4651	chr14	29035565	29082867	-	3	1.60403	0.831	1.86775	0.499992	1.59786	2.30151	2.49606	3.04618	2.01775
ENST00000549742.1	ENSG00000258175.1	RP11-412H8.2	lincRNA	665	chr14	29061429	29082829	-	3	8.29868	2.03876	8.49448	4.94695	6.53297	11.5608	16.226	14.241	9.18983
TCONS_00087753	XLOC_007659	-	lincRNA	5536	chr14	29191417	29200769	-	3	2.75624	0.67447	2.05894	0.539447	1.95508	0.513645	0.708233	1.11191	0.540042
TCONS_00088173	XLOC_007212	-	lincRNA	459	chr14	29233521	29235255	+	2	3.12989	1.11188	1.94158	0.497357	0.96779	1.09573	0.939259	0.320463	0.454243
TCONS_00091663	XLOC_007218	-	lincRNA	502	chr14	32019852	32027250	+	2	0	0.71308	0.23673	0.852232	0.206858	0.469212	1.00018	0.820289	1.74775
TCONS_00092910	XLOC_007220	-	lincRNA	630	chr14	32476091	32496930	+	2	0.94575	0.49813	0.16481	0	1.01126	0.657399	1.24915	0.380509	0.271279
TCONS_00096063	XLOC_007224	-	intronic_lncRNA	1272	chr14	33585590	33587337	+	2	2.75855	1.98289	2.48111	0.597539	0.459904	0.788214	0.437835	1.95822	0.97174
TCONS_00095073	XLOC_007227	-	lincRNA	593	chr14	34420438	34422155	+	2	0.25858	0.1819	0	0.163489	0.158265	0	0	1.8774	0.594368
TCONS_00089398	XLOC_007674	-	lincRNA	508	chr14	35020932	35024358	-	2	4.61009	4.23072	2.11974	1.62711	2.46652	1.56551	3.73587	1.64264	1.73183
TCONS_00089399	XLOC_007674	-	lincRNA	519	chr14	35020932	35024358	-	2	0.00202	3.11165	0.64053	2.86536	0.750255	0.708937	1.12205	2.55054	2.00224
TCONS_00090407	XLOC_007241	-	intronic_lncRNA	2432	chr14	37405397	37407961	+	2	1.18907	0.91818	1.12411	0.573461	0.770972	0.819199	0.470357	1.08028	0.491317
TCONS_00090690	XLOC_007690	-	anti-sense_lncRNA	461	chr14	37967395	37970027	-	2	4.65917	3.03377	1.9262	0.24676	1.20027	1.63089	2.09639	1.27168	1.80276
TCONS_00087803	XLOC_007695	-	lincRNA	1761	chr14	39229871	39232380	-	2	2.15705	0.58914	0.80502	2.13267	1.4583	2.25369	0.636661	1.44427	0.740275
TCONS_00091683	XLOC_007251	-	lincRNA	1700	chr14	39901833	39941174	+	2	1.56445	1.22638	1.16382	0.981703	1.88704	1.50112	0.662761	2.04029	1.30984
TCONS_00089077	XLOC_007253	-	lincRNA	988	chr14	40124062	40125612	+	2	1.42243	0.72067	0.8018	1.13878	0.940285	1.52041	1.04662	1.02785	0.809381
TCONS_00095311	XLOC_007699	-	lincRNA	5854	chr14	42052940	42075160	-	3	2.08648	1.65272	1.91035	0.48647	0.379974	0.793866	1.97808	1.47207	0.740202
ENST00000557067.1	ENSG00000258636.1	CTD-2298J14.2	lincRNA	530	chr14	42057064	42074059	-	2	5.07522	2.94825	4.03064	0	0.753941	0.885953	3.94288	0.793985	0.244801
TCONS_00090431	XLOC_007275	-	lincRNA	898	chr14	51411790	51413971	+	2	1.16748	0.50895	0.40289	0.459288	0.708346	0.404044	0.507406	0.581089	0.914577
TCONS_00094073	XLOC_007293	-	lincRNA	297	chr14	53629470	53635105	+	2	0	1.21206	1.08571	0	0	0.733256	2.65109	0	3.34E-05
TCONS_00091702	XLOC_007293	-	lincRNA	350	chr14	53632507	53635105	+	2	0.67053	0.67705	1.26732	1.72374	0	0	1.67367	0.566567	0.795013
TCONS_00090723	XLOC_007738	-	lincRNA	409	chr14	54259966	54316699	-	2	0.62665	1.15205	3.07449	0.007013	0	0.680427	0	2.40285	1.31445
TCONS_00091970	XLOC_007738	-	lincRNA	373	chr14	54316513	54317609	-	2	1.57671	1.12216	0.89713	2.23182	2.92704	0.411842	0	0	1.83811
TCONS_00090728	XLOC_007743	-	anti-sense_lncRNA	350	chr14	55242685	55248124	-	3	0	0.48612	1.46939	0	0	0	0	0	0

TCONS_00089120	XLOC_007315	-	lincRNA	724	chr14	59357142	59361487	+	2	0.97098	0.13595	0.13474	0.367409	0.473066	1.07776	0	0.155507	0.444189
TCONS_00092641	XLOC_007763	-	lincRNA	1842	chr14	59921860	59928206	-	4	5.71733	5.16908	5.44066	2.41721	2.39761	3.81336	1.85066	2.4511	2.70899
ENST00000557618.1	ENSG00000258670.1	RP11-1042B17.3	lincRNA	868	chr14	60981837	61021634	+	2	2.74442	1.91519	0.94776	0.0959946	0.74043	0.738956	0.265306	0.243022	0.955995
TCONS_00091732	XLOC_007329	-	lincRNA	4862	chr14	61580080	61585241	+	2	1.55536	0.66612	0.64151	0.933062	1.51399	1.79399	0.973067	1.03215	0.729488
TCONS_00094299	XLOC_007775	-	lincRNA	788	chr14	62060563	62065991	-	3	0.17314	0.24202	0	1.74555	0.105269	2.15985	0	0.138309	0.0988454
TCONS_00090166	XLOC_007807	-	intronic_lncRNA	1367	chr14	68549240	68555361	-	2	2.18483	1.82072	1.07886	0.109764	0.158356	0.120651	1.00453	0.414815	0.247849
ENST00000556301.1	ENSG00000259038.1	CTD-2325P2.4	lincRNA	279	chr14	69093883	69095162	-	2	11.0643	13.7811	6.59686	2.41435	4.0222	4.45685	16.3236	4.3669	6.78578
TCONS_00087932	XLOC_007810	-	lincRNA	4484	chr14	69157944	69190851	-	3	2.83939	1.05079	2.0965	0.714003	0.742505	0.501049	0.325668	0.673843	0.414589
TCONS_00087931	XLOC_007810	-	lincRNA	3501	chr14	69158874	69190793	-	3	2.65682	1.18133	1.00978	0.371854	0.691623	0.499614	0.000299	0.0002685	0.178036
ENST00000500016.1	ENSG00000245466.1	CTD-2540L5.6	lincRNA	2941	chr14	71075515	71108015	-	3	2.1976	0.48963	2.97274	0.280136	1.18778	1.05169	1.57301	0.93759	1.28387
TCONS_00092991	XLOC_007374	-	lincRNA	2346	chr14	71165101	71180393	+	3	0.57077	0.0988	0.3573	0.328052	1.77574	0.589891	0.462127	0.486963	0.780033
TCONS_00094118	XLOC_007376	-	lincRNA	378	chr14	71288652	71290726	+	2	0	0.81596	0.40958	0.725657	0.710655	1.60062	1.04629	0.473599	1.00047
TCONS_00092334	XLOC_007378	-	lincRNA	3590	chr14	71787141	71952187	+	5	5.5724	2.8327	4.10623	3.01967	3.85812	4.79014	2.74766	2.62792	3.23211
TCONS_00087978	XLOC_007860	-	lincRNA	1263	chr14	77539917	77552808	-	3	1.97522	0.63231	0.73204	0.293859	0.548094	0.865352	0.442157	0.342278	1.03981
TCONS_00089517	XLOC_007860	-	lincRNA	1401	chr14	77539936	77542842	-	2	0.20458	0.97376	0.98307	0.539845	0.130589	0.46519	0.145856	0.637644	0.139743
TCONS_00087998	XLOC_007876	-	anti-sense_lncRNA	4580	chr14	81557725	81567855	-	3	1.8653	0.84407	1.53092	0.569608	0.896886	1.23916	0.460469	1.04306	0.701718
TCONS_00089527	XLOC_007876	-	anti-sense_lncRNA	1048	chr14	81560558	81566679	-	2	0	0.38672	0.3807	0	0	0.812465	0.022746	0.194754	0.0467313
TCONS_00088846	XLOC_007878	-	lincRNA	1066	chr14	81892621	81893774	-	2	2.82465	0.90111	1.29572	1.10998	1.85258	1.54577	0.679304	0.747415	0.936741
TCONS_00093032	XLOC_007433	-	intronic_lncRNA	3472	chr14	86016939	86028756	+	2	17.4757	12.0854	8.57326	8.21267	4.16641	5.82243	4.05714	6.01014	2.57334
TCONS_00088447	XLOC_007434	-	lincRNA	1060	chr14	86400912	86446079	+	2	1.42216	0.82494	3.01748	1.26678	0.932795	1.63851	1.36822	3.01075	2.82996
TCONS_00091128	XLOC_007434	-	lincRNA	1938	chr14	86499427	86508620	+	5	0.76332	0.69171	1.92698	0.478809	0.601571	0.526195	0.974689	2.17608	1.82777
TCONS_00091521	XLOC_007896	-	intronic_lncRNA	2529	chr14	91844764	91867545	-	2	1.31491	1.57766	2.63294	1.29145	1.7678	2.29473	1.35199	2.44976	3.22129
TCONS_00090835	XLOC_007904	-	lincRNA	580	chr14	92975780	92977848	-	2	0.80212	0.37642	0.374	1.01466	0.163762	0	1.10356	1.29549	0.461251
TCONS_00089588	XLOC_007927	-	lincRNA	583	chr14	95792719	95794299	-	2	0	0.74685	0	0.167779	0	0.184656	0	0.214181	1.0677
TCONS_00087575	XLOC_007492	-	lincRNA	3315	chr14	97196752	97204417	+	2	2.75339	0.7708	0.31288	0.0410802	0.0197153	0.18055	0.243033	0.257713	0.185156
TCONS_00089269	XLOC_007515	-	lincRNA	3324	chr14	101437149	101455234	+	10	2.65212	3.20185	1.72029	2.53418	1.709	2.10632	3.236	2.83143	1.01828
TCONS_00089270	XLOC_007515	-	lincRNA	2664	chr14	101440913	101451371	+	4	1.98272	2.96365	2.19984	2.11057	1.94663	1.40979	4.24437	1.9503	0.745757
TCONS_00087599	XLOC_007516	-	lincRNA	1794	chr14	101505067	101507077	+	2	0.64014	0.9763	0.78826	1.12456	0.964869	0.48553	1.17342	0.707096	0.543672
TCONS_00095210	XLOC_007519	-	lincRNA	1723	chr14	101520725	101522540	+	2	1.00491	0.74326	0.55015	1.30316	0.646411	0.462003	2.0732	1.42765	0.607051
ENST00000557121.1	ENSG00000259004.1	RP11-8L8.2	lincRNA	943	chr14	101587193	101589879	+	2	0.82273	0.76464	0	0.776521	0.249418	0.284615	0.555529	0.10909	0.85877
ENST00000553561.1	ENSG00000258460.1	RP11-168L7.1	lincRNA	447	chr14	101798853	101800668	-	2	0.41007	0	0.29132	0.521693	0.507933	0	0.493564	0.336614	0.238399
TCONS_00093308	XLOC_007947	-	lincRNA	563	chr14	101821341	101822769	-	3	0.27982	1.97155	0.39195	1.94778	2.05862	0.584708	2.47928	0.678871	1.28849
TCONS_00090281	XLOC_007950	-	lincRNA	1557	chr14	102023240	102026574	-	3	0.29466	1.10763	0.88559	0.939763	0.73507	0.502373	0.809427	0.43196	0.309265
ENST00000555882.1	ENSG00000258498.2	DIO3OS	lincRNA	789	chr14	102024890	102026643	-	2	0.3596	1.66278	0.81368	2.07848	0.188743	0.396905	1.73272	1.06681	0.665118
TCONS_00087603	XLOC_007522	-	intronic_lncRNA	5281	chr14	102287820	102293232	+	2	3.93976	3.764	5.88517	5.90457	3.84093	6.39025	2.78091	5.09737	6.26161
ENST00000559675.1	ENSG00000259508.1	RP11-661D19.3	lincRNA	567	chr14	103236377	103241128	-	2	1.93753	2.53467	5.8136	0.875656	0.848265	1.34934	1.96108	1.56632	0.955677
TCONS_00094187	XLOC_007541	-	lincRNA	707	chr14	104343607	104346121	+	2	0.60204	0.56221	1.39335	0.126592	0.489107	1.11408	1.17202	1.28655	0.918494
TCONS_00089632	XLOC_007983	-	lincRNA	344	chr14	106963048	106963476	-	2	0.69782	5.06826	3.57791	38.6081	0	0.991114	0	0.591309	2.48697
TCONS_00097909	XLOC_008011	-	lincRNA	385	chr15	24698879	24700428	+	2	3.27417	0.78433	0	0	0.683053	1.53943	0.334772	0.909741	0
TCONS_00107062	XLOC_008016	-	lincRNA	3449	chr15	25299167	25305309	+	3	2.13707	2.12994	1.19978	1.16186	1.24745	1.62279	1.5412	0.889392	1.66856
TCONS_00102872	XLOC_008020	-	lincRNA	252	chr15	25512032	25513567	+	2	1.74461	0	0	3.47054	0	0	1.20454	0	0
TCONS_00104430	XLOC_008454	-	lincRNA	241	chr15	29126972	29129701	-	2	0	0	0	0	0	0	0	0	1.31663
TCONS_00099390	XLOC_008458	-	anti-sense_lncRNA	1020	chr15	30906651	30918180	-	2	4.34977	3.98106	4.62106	2.11031	3.7639	1.46089	4.23608	3.06057	2.33274
TCONS_00102054	XLOC_008042	-	lincRNA	1546	chr15	30916661	30918316	+	2	0.45474	0.73622	1.14212	0.428161	0.640302	0.522875	0.608815	0.479021	0.300654
TCONS_00105914	XLOC_008051	-	lincRNA	481	chr15	32828970	32878382	+	3	2.52594	2.81377	2.80472	0.916337	2.00365	3.53215	1.94072	2.94545	1.67194
TCONS_00102455	XLOC_008470	-	anti-sense_lncRNA	965	chr15	32896154	32907597	-	2	1.59827	1.67061	1.83597	0.754134	1.21096	1.19774	1.84902	1.90645	1.44036
TCONS_00105041	XLOC_008474	-	anti-sense_lncRNA	360	chr15	34145246	34147113	-	2	6.29456	2.73035	3.20489	1.2117	3.56804	4.01078	3.12505	5.82484	1.86028
TCONS_00097964	XLOC_008064	-	intronic_lncRNA	2688	chr15	36952219	36954958	+	2	3.56977	1.24944	2.46408	1.85186	3.03749	2.82622	1.07774	1.96971	1.29876
TCONS_00104707	XLOC_008067	-	lincRNA	555	chr15	38285601	38317349	+	2	3.59973	3.85876	2.9806	0.464322	1.49839	1.725	0.935414	1.61681	1.68308
TCONS_00104709	XLOC_008067	-	lincRNA	861	chr15	38312216	38317180	+	3	4.94619	3.16688	5.2977	0.75706	2.60653	1.10114	2.68736	3.14449	2.62887
TCONS_00106634	XLOC_008067	-	lincRNA	5143	chr15	38312243	38358395	+	5	6.43393	3.97412	5.5548	0.487296	1.69915	1.9994	3.8707	0.911064	1.76042
TCONS_00104212	XLOC_008067	-	lincRNA	4944	chr15	38351498	38358621	+	2	1.01013	0.28366	0.99018	0.539066	0.540043	0.109639	0.678966	0.511421	0.678986
ENST00000565315.1	ENSG00000261183.1	RP11-532F12.5	anti-sense_lncRNA	629	chr15	41128006	41136530	-	2	2.26069	2.91885	2.42589	0	1.88762	1.33145	3.41279	1.61802	0.96814
ENST00000564302.1	ENSG00000261183.1	RP11-532F12.5	anti-sense_lncRNA	643	chr15	41128191	41136585	-	3	6.53268	7.969	5.64591	2.60925	8.82581	6.5263	12.0433	8.95403	7.4858
TCONS_00098521	XLOC_008556	-	lincRNA	734	chr15	43941370	43956641	-	2	3.43034	1.73386	1.05738	0.961346	2.0885	1.18966	0.666836	1.52544	0.871566
TCONS_00103444	XLOC_008124	-	lincRNA	1958	chr15	45021189	45025159	+	2	18.2275	16.1702	37.3846	21.3739	14.8327	17.7268	33.3579	37.8519	51.1191
TCONS_00100289	XLOC_008568	-	lincRNA	523	chr15	45139700	45174832	-	4	0.33183	3.11398	0.82819	0.199126	0	0.178306	0	0.305117	1.01176
TCONS_00100290	XLOC_008568	-	lincRNA	3396	chr15	45170890	45174997	-	2	0.28575	0.59595	1.50835	0.440423	0.230576	0.268062	0.382604	0.422055	0.747463
TCONS_00101818	XLOC_008575	-	lincRNA	455	chr15	45689184	45694404	-	3	0.39732	0.28242	1.97308	0.505213	0.983302	0	0.954693	0.977016	2.30761
ENST00000560359.1	ENSG00000244879.3	GABPB1-AS1	anti-sense_lncRNA	709	chr15	50647525	50648567	+	2	3.98982	6.18934	5.4261	5.05806	4.11436	4.17041	3.52964	5.94588	3.4836
ENST00000499624.2	ENSG00000244879.3	GABPB1-AS1	anti-sense_lncRNA	2310	chr15	50647664	50650527	+	2	20.4647	8.73557	14.038	4.81936	10.9888	10.5306	13.5976	7.23452	7.5381

TCONS_00102179	XLOC_008177	-	anti-sense_lncRNA	2024	chr15	60771364	60822442	+	3	1.62259	0.46522	1.68271	0.701941	1.21366	1.2727	1.63275	1.85251	1.89963
TCONS_00099997	XLOC_008185	-	lincRNA	692	chr15	63724294	63729556	+	2	1.86095	1.0142	1.00562	0.652329	2.14282	3.30117	3.86768	4.64287	2.9588
TCONS_00102981	XLOC_008215	-	lincRNA	326	chr15	68805107	68828063	+	2	0.79445	0	0.58746	1.53874	0	0	1.00734	1.35964	1.42514
TCONS_00102986	XLOC_008227	-	lincRNA	458	chr15	69760308	69760845	+	2	3.85912	2.82684	0.33494	1.25223	1.4208	0.458268	0.735429	0	0.549837
TCONS_00099136	XLOC_008227	-	lincRNA	1282	chr15	69760335	69819241	+	3	2.18402	0.64635	1.79804	0.295059	0.578129	0.607393	0.589916	0.149228	0.78026
ENST00000558411.1	ENSG00000259641.1	RP11-279F6.3	lincRNA	2121	chr15	69884468	69905229	+	2	1.54075	0.51522	1.52475	0.0999581	0.28805	0.109846	0.121552	0.293073	0.33063
TCONS_00106985	XLOC_008695	-	intronic_lncRNA	899	chr15	71773541	71816749	-	3	2.47735	2.13451	1.60922	0.917258	0.176831	0.201732	1.09778	0.696295	0.664188
TCONS_00106990	XLOC_008697	-	intronic_lncRNA	1037	chr15	72464848	72466087	-	2	1.82642	4.32355	2.26309	0.918822	0.737386	0.926022	1.75796	0.676902	0.484707
TCONS_00104597	XLOC_008714	-	intronic_lncRNA	660	chr15	74603796	74612016	-	2	1.10545	0.77543	0.46155	0	1.34926	1.84284	2.97848	0.710361	1.7736
ENST00000560590.1	ENSG00000259420.1	RP11-307C19.2	lincRNA	702	chr15	77861312	77865206	+	3	0.58195	0.61906	0.91935	0	0.741764	0.790703	0.0016567	1.67229	0.414936
TCONS_00106752	XLOC_008274	-	lincRNA	1312	chr15	77864559	77865989	+	2	0.74546	0.48669	0.72163	0.0576009	0.276728	0.657122	1.10676	0.63191	0.230093
TCONS_00098802	XLOC_008733	-	intronic_lncRNA	251	chr15	77910847	77911471	-	2	5.31266	0	0	0	2.37475	0	0	0	2.22308
TCONS_00103332	XLOC_008749	-	lincRNA	1106	chr15	79657532	79665223	-	2	0.67506	0.23484	0.30949	2.61682	2.04262	0.466524	0.194672	0.267778	0.0639378
TCONS_00100472	XLOC_008755	-	lincRNA	255	chr15	80672279	80690323	-	2	0	5.06931	0	0	0	1.22683	1.14718	3.04986	3.13216
TCONS_00100473	XLOC_008756	-	intronic_lncRNA	265	chr15	80772754	80773325	-	2	1.45646	2.19591	0	0	0	0	0	0	0
TCONS_00104623	XLOC_008762	-	lincRNA	399	chr15	82245682	82246961	-	2	2.0312	0.36393	0.36461	0	0.316886	0.357499	0	0	1.18973
TCONS_00098842	XLOC_008796	-	lincRNA	1332	chr15	85857352	85873776	-	2	1.08108	1.18895	2.6576	1.13163	0.97966	0.808535	1.13952	2.70906	2.0444
TCONS_00103064	XLOC_008347	-	lincRNA	3208	chr15	89177992	89181665	+	2	0.95042	1.05654	1.78225	1.02102	1.1434	1.28547	1.02621	1.62814	1.45733
ENST00000558221.1	ENSG00000259704.1	CTD-3094K11.1	lincRNA	402	chr15	91382954	91384608	+	2	0	0.35839	0.71794	0.958289	0	1.40853	1.52503	0.414959	0.878709
TCONS_00104659	XLOC_008827	-	lincRNA	582	chr15	92011920	92012975	-	2	0.53197	0	0.186	6.22416	2.44337	0.74057	0.313598	0.214756	0.305867
TCONS_00103086	XLOC_008381	-	lincRNA	1173	chr15	92127669	92148997	+	2	1.36214	1.23861	0.93595	0.921837	1.01394	0.579085	0.543298	0.913589	1.7853
TCONS_00103092	XLOC_008390	-	lincRNA	259	chr15	94039526	94041973	+	2	1.57817	0	0	1.04249	1.04992	1.15665	0	0	0
TCONS_00105654	XLOC_008400	-	lincRNA	3521	chr15	96533669	96651700	+	2	0.73792	0.25515	0.08384	0.866939	1.20187	1.92626	0.210372	0.604205	0.191017
TCONS_00099824	XLOC_008845	-	lincRNA	1110	chr15	96644942	96647885	-	2	2.57638	1.16901	1.23246	0.774548	0.813435	0.464468	0.581416	1.24408	0.509241
ENST00000558382.1	ENSG00000259275.1	RP11-522B15.3	lincRNA	501	chr15	96897466	96946964	+	2	0	0.71549	0.23754	0.21377	0.622674	0.941567	0.401454	0	0.779405
TCONS_00107865	XLOC_008401	-	lincRNA	1201	chr15	96897681	96938675	+	2	2.85194	1.98269	1.46925	1.024	1.35495	1.61818	1.05583	1.8563	0.983213
TCONS_00098356	XLOC_008412	-	lincRNA	243	chr15	99993576	99994296	+	2	2.00823	0	0	0	0	1.48812	0	0	1.27082
TCONS_00099322	XLOC_008416	-	lincRNA	1832	chr15	100417086	100422445	+	2	3.25131	1.17019	1.83898	1.37293	1.0554	0	1.03847	1.38107	1.06194
TCONS_00100530	XLOC_008871	-	intronic_lncRNA	326	chr15	101051848	101052549	-	2	4.76671	1.1612	0	0	0	0.566553	0.503671	0	0
TCONS_00114568	XLOC_008916	-	anti-sense_lncRNA	241	chr16	824683	825035	+	2	0	0	0	1.38626	0	1.54083	0	0	0
TCONS_00114903	XLOC_009466	-	anti-sense_lncRNA	298	chr16	1259482	1260062	-	2	0	0.74656	0	0	0.652244	0	0	1.76161	0.611644
ENST00000574387.1	ENSG00000262370.1	RP11-473M20.9	lincRNA	677	chr16	3128321	3137088	-	3	1.27948	2.84041	3.55831	4.84505	3.38155	4.29395	3.61816	2.05362	3.41929
ENST00000573447.1	ENSG00000263072.1	RP11-473M20.14	lincRNA	1117	chr16	3178834	3182043	-	2	4.5577	2.3974	3.74533	1.9565	2.28703	2.53502	2.62818	2.64533	1.89499
TCONS_00118472	XLOC_008976	-	lincRNA	1010	chr16	3661794	3691511	+	2	1.50971	2.97909	1.55957	2.69031	1.52428	2.95797	0.94507	1.99922	2.86271
ENST00000573220.1	ENSG00000263105.1	RP11-95P2.3	lincRNA	318	chr16	4230118	4233516	-	2	0	0	0.62882	0	0.541259	0	1.07979	0	0
TCONS_00111719	XLOC_009004	-	lincRNA	1390	chr16	9583911	9611977	+	3	1.11419	1.30927	0.41139	0.161458	0.10352	0.059157	0.443213	0.203367	0.0972147
TCONS_00111274	XLOC_009559	-	lincRNA	1004	chr16	10608258	10619987	-	2	2.66166	1.05934	0.61108	0.797186	1.38216	1.22711	0.146497	1.9136	0.865268
TCONS_00116191	XLOC_009562	-	lincRNA	371	chr16	10827125	10836088	-	2	0.58966	0.4251	0.42711	0.377779	1.11083	0	0	0	1.04254
TCONS_00111728	XLOC_009010	-	lincRNA	1129	chr16	11406760	11411091	+	2	0.32919	1.06863	1.20702	0.896616	0.663914	0.0758236	1.01221	1.21838	1.43394
TCONS_00117233	XLOC_009569	-	lincRNA	323	chr16	11487324	11490597	-	2	0	0.59494	1.20469	0	0.518915	1.74103	0	0.697091	0
TCONS_00114959	XLOC_009573	-	lincRNA	225	chr16	11524908	11529564	-	2	2.79276	4.33915	0	0	1.93169	2.10158	0	0	0
TCONS_00112232	XLOC_009588	-	lincRNA	3307	chr16	14100973	14110078	-	4	14.7009	4.70291	8.09774	1.28882	5.44584	4.23658	5.22355	6.75803	5.39791
ENST00000575424.1	ENSG00000262097.1	CTD-2135D7.5	lincRNA	760	chr16	14103137	14109873	-	3	3.75483	3.31644	4.35737	1.76609	2.37605	1.48241	2.99694	3.55278	3.24142
TCONS_00110341	XLOC_009596	-	lincRNA	291	chr16	15224555	15225140	-	2	8.64941	6.42098	4.09825	3.52347	2.10513	0	3.53872	1.89846	3.94751
TCONS_00110343	XLOC_009597	-	lincRNA	211	chr16	15226806	15227218	-	2	7.63867	6.00337	6.46865	0	0	0	2.8642	3.7552	2.51662
ENST00000576433.1	ENSG00000261465.2	RP11-626G11.5	anti-sense_lncRNA	455	chr16	19074076	19078717	-	3	0	2.37318	1.61691	0.252607	1.47495	0	4.50691	0	1.2974
TCONS_00110367	XLOC_009615	-	anti-sense_lncRNA	575	chr16	19074161	19075644	-	2	2.16735	0.68615	0.23952	0	0	0	0.178489	1.53206	0.838016
TCONS_00110368	XLOC_009617	-	lincRNA	1164	chr16	19313379	19316732	-	2	3.70145	1.61796	2.18023	0.199384	1.1514	1.24208	2.07186	1.42522	1.32151
TCONS_00111326	XLOC_009618	-	lincRNA	2490	chr16	19377493	19405235	-	3	3.14811	2.4992	3.95618	0.0001971	0.864768	1.29713	1.13495	0.758443	2.2231
TCONS_00118275	XLOC_009618	-	lincRNA	2278	chr16	19377569	19405344	-	2	0.00845	3.81E-05	0	0	0.644359	1.19337	0.883211	1.968	0.907329
TCONS_00110369	XLOC_009618	-	lincRNA	2403	chr16	19377608	19405401	-	3	3.20436	0	0.2446	0.221711	8.90E-05	4.01E-05	0	0.0002096	0
TCONS_00118767	XLOC_009618	-	lincRNA	931	chr16	19392586	19405355	-	2	3.35152	0.97232	3.1974	0.119363	1.01325	0.241048	3.79038	1.94263	2.70848
TCONS_00114649	XLOC_009051	-	anti-sense_lncRNA	3848	chr16	20685842	20694024	+	2	1.34362	0.56125	1.18269	2.75339	2.3394	2.83253	0.59031	1.58373	0.979984
TCONS_00111336	XLOC_009628	-	anti-sense_lncRNA	846	chr16	20754577	20775447	-	2	3.46815	0.99048	3.05037	0.595689	0.670135	0.764232	1.09805	0.879972	1.07873
TCONS_00109734	XLOC_009055	-	lincRNA	1108	chr16	21327479	21358496	+	3	2.69431	1.09349	1.158	0.917396	1.63048	1.62923	1.74814	2.76088	1.6587
TCONS_00114674	XLOC_009087	-	lincRNA	372	chr16	24838913	24841196	+	2	0	2.11283	0.42451	2.25341	0	0.828522	0.361704	0.490905	0.690877
TCONS_00115614	XLOC_009653	-	lincRNA	1420	chr16	26604869	26606723	-	2	0.75243	0.29021	0.22926	0.787409	0	0.288496	0.384182	0.925529	1.56442
ENST00000570025.1	ENSG00000260083.1	MIR4519	anti-sense_lncRNA	1429	chr16	30886543	30906400	+	2	2.24079	1.90131	2.84458	0.885851	2.0044	1.43189	2.33571	2.49361	2.30584
ENST00000570266.1	ENSG00000260911.1	RP11-196G11.2	lincRNA	461	chr16	31054471	31061201	+	2	0.77653	1.37899	2.47654	1.48056	0.480108	0.543629	0.465863	0	1.35207
TCONS_00114410	XLOC_009730	-	lincRNA	4688	chr16	46397969	46408768	-	2	54.795	30.2267	12.2282	2.66544	6.11326	1.44075	9.24061	27.9677	10.879
TCONS_00111412	XLOC_009731	-	lincRNA	2716	chr16	46419359	46434134	-	2	15.0535	9.49095	3.0457	0.610413	1.56285	0.251526	3.49823	6.92873	3.07308

TCONS_00111413	XLOC_009732	-	lincRNA	311	chr16	46422518	46434873	-	2	22.4308	5.2777	2.67915	0	2.30318	0	0	0.775324	1.08018
TCONS_00113777	XLOC_009736	-	lincRNA	240	chr16	46660866	46663519	-	2	0	1.62078	1.69891	0	1.43293	0	1.48899	0	4.02169
TCONS_00110493	XLOC_009751	-	lincRNA	435	chr16	49035789	49036358	-	2	1.29241	0.30699	0.61349	0.274265	0	0	1.04012	0	0.501706
ENST00000567728.1	ENSG00000261644.1	RP11-327F22.2	lincRNA	1150	chr16	50769624	50774627	-	2	0.42924	0.52244	0.22127	0.269771	0.194745	0.815572	0.18554	1.10609	0.609591
ENST00000563315.1	ENSG00000260929.1	RP11-327F22.1	anti-sense_lncRNA	640	chr16	50774821	50776726	-	2	0.23109	0.32445	0.16098	0.291901	0.282279	0.963554	0.406653	0.557511	0.795105
TCONS_00111042	XLOC_009189	-	lincRNA	1033	chr16	51789166	51812234	+	4	0.24537	0	0.59766	0.470329	0.625386	0	0	0.0879386	0.190248
TCONS_00119868	XLOC_009189	-	lincRNA	1104	chr16	51789166	51821520	+	5	2.62697	0.63823	2.23987	0	0.902867	1.40542	0.620788	0.297237	1.17514
TCONS_00111043	XLOC_009189	-	lincRNA	1295	chr16	51789166	51812234	+	4	0.77083	1.95142	0.54998	0.133735	0.0707669	0	0.60149	0.874854	0.0074786
TCONS_00111918	XLOC_009189	-	lincRNA	1176	chr16	51789212	51847935	+	5	3.85976	2.70652	4.86363	6.21E-06	1.73597	1.45359	1.81512	0.354668	1.06264
TCONS_00116263	XLOC_009763	-	lincRNA	261	chr16	52197030	52215510	-	2	0	0	0	1.01337	2.03953	0	1.04524	1.3917	0
TCONS_00111919	XLOC_009191	-	intronic_lncRNA	1543	chr16	53132482	53156055	+	2	0.9345	1.89257	0.9732	0.473874	1.40153	1.29157	0.192684	1.22718	1.41827
TCONS_00111920	XLOC_009191	-	intronic_lncRNA	1603	chr16	53148828	53156456	+	2	2.66845	0.50902	1.20971	1.50884	1.46634	2.42539	1.44211	1.06537	1.81492
ENST00000560029.1	ENSG00000245694.4	CRNDE	lincRNA	659	chr16	54952782	54954528	-	2	6.20395	8.54854	8.48047	6.85433	5.13851	7.07971	8.69585	6.94143	9.01451
TCONS_00112347	XLOC_009775	-	lincRNA	1695	chr16	55477239	55479333	-	2	0.40948	0.99386	0.09342	0.0856513	0.123481	0.188269	0.352057	0.646469	0.502501
TCONS_00116791	XLOC_009782	-	lincRNA	1578	chr16	56184737	56196432	-	2	1.33258	1.38674	0.96331	0.511135	0.848719	0.510709	0.721956	1.40346	0.46144
TCONS_00109918	XLOC_009212	-	lincRNA	509	chr16	56697292	56698890	+	2	2.29868	0	0.92484	0	0.202086	0.229247	0.781296	0	0.758865
TCONS_00118341	XLOC_009807	-	lincRNA	724	chr16	63101398	63124427	-	2	1.55357	0.27189	0.80843	0.12247	1.18266	0.40416	1.35972	1.08855	0.444189
TCONS_00111462	XLOC_009808	-	lincRNA	4988	chr16	63334957	63652068	-	4	2.15372	0.53524	1.37894	0.607984	3.26931	1.24463	0.961669	1.56642	0.646748
TCONS_00113068	XLOC_009808	-	lincRNA	2249	chr16	63366783	63651976	-	3	0.44981	0.36872	0.71685	0.327764	0.928507	0.281741	0.251318	1.19588	0.31771
TCONS_00113819	XLOC_009809	-	lincRNA	1583	chr16	63440959	63442871	-	2	1.62283	0.51174	1.11135	0.370389	1.15719	0.712403	0.380818	1.1653	0.376171
TCONS_00112371	XLOC_009811	-	lincRNA	208	chr16	64787221	64788542	-	2	0	3.24332	0	0	0	0	0	0	2.72483
TCONS_00110552	XLOC_009815	-	lincRNA	562	chr16	65844703	65846680	-	2	1.68355	0.79083	0.58959	0.532688	1.20423	1.36811	0.663031	1.58852	0.161513
TCONS_00119262	XLOC_009816	-	lincRNA	447	chr16	65952917	66167853	-	2	0	0	0	0.310262	0	0.287374	0.318258	3.27205	0
TCONS_00117328	XLOC_009816	-	lincRNA	4752	chr16	66161466	66167951	-	2	0.44871	1.02335	0.32088	1.24807	1.01128	2.64025	1.63182	0.921112	0.15196
TCONS_00110553	XLOC_009819	-	lincRNA	262	chr16	66280377	66300959	-	2	0	0	2.36655	0	0	0	0	0	0
TCONS_00112378	XLOC_009819	-	lincRNA	484	chr16	66280377	66281993	-	2	0	0.75913	1.26099	0	0	0.998431	0	0.291332	0
ENST00000499966.1	ENSG00000246898.1	LINC00920	lincRNA	2147	chr16	66442427	66446038	+	2	1.41535	0.7988	1.79083	0.98622	1.45254	0.794767	1.85874	3.13923	1.5717
TCONS_00113466	XLOC_009250	-	intronic_lncRNA	328	chr16	66471640	66475941	+	2	1.56489	0.5714	2.31166	0	0.996546	0.557708	0.495324	0	0
ENST00000602596.1	ENSG00000270049.1	RP11-297D21.4	anti-sense_lncRNA	696	chr16	67515287	67518572	+	2	0.61534	1.72446	2.13722	1.16469	1.50024	1.13892	1.67812	1.31563	1.52603
TCONS_00113881	XLOC_009889	-	lincRNA	1524	chr16	72276850	72383132	-	8	0.79465	0.28246	1.64014	0.665671	1.90692	1.36786	1.73161	1.88339	1.43772
TCONS_00112443	XLOC_009889	-	lincRNA	1166	chr16	72277158	72342225	-	7	1.92416	1.95518	0.64732	1.06347	0.318543	0.87931	5.22E-05	0.0008617	0.861529
TCONS_00111558	XLOC_009889	-	lincRNA	577	chr16	72283026	72324551	-	4	2.00883	0.20416	0.00011	0.0344027	0	0.226649	2.04033	1.31767	0.728944
TCONS_00112445	XLOC_009889	-	lincRNA	1441	chr16	72555466	72698929	-	6	0.73972	1.14112	1.46477	0.980481	1.14134	1.6449	1.32166	1.42961	1.21162
TCONS_00115104	XLOC_009891	-	lincRNA	476	chr16	73352171	73925643	-	4	0.53544	0.22975	0.71584	4.7088	1.09407	0.38889	0.0012532	1.40433	2.32914
TCONS_00116334	XLOC_009891	-	lincRNA	591	chr16	73489756	73925677	-	3	0.92064	1.23217	0.22594	0	0.566288	0.630501	2.2928	0	0.369182
TCONS_00115105	XLOC_009891	-	lincRNA	287	chr16	73643863	73925643	-	3	0	0	0	3.60608	0	0.814037	0.000758	0	1.00692
TCONS_00115106	XLOC_009891	-	lincRNA	1288	chr16	73712946	73925643	-	2	0.65609	0.87124	0.2573	2.05526	0.714503	0.388297	0.647788	0.617325	0.219183
TCONS_00118900	XLOC_009906	-	lincRNA	1702	chr16	75539152	75549338	-	2	3.39646	1.55447	2.09206	0.596762	1.76161	2.71715	1.67415	1.39411	1.4235
TCONS_00110107	XLOC_009349	-	intronic_lncRNA	748	chr16	79027732	79030280	+	2	2.2287	1.03946	0.77245	0.819614	0.904262	1.41671	0.54118	2.08011	1.37974
ENST00000569938.1	ENSG00000261472.1	RP11-467I17.1	lincRNA	419	chr16	79539500	79550190	+	2	0.92388	0.32994	0	0.294455	0	0	0.560127	0	0
TCONS_00113538	XLOC_009351	-	lincRNA	1629	chr16	79635820	79640634	+	2	3.42692	1.78275	2.1996	1.07535	1.59355	1.18188	1.31006	2.70609	1.29428
TCONS_00113163	XLOC_009933	-	intronic_lncRNA	507	chr16	82077979	82086169	-	2	0.66101	1.16878	1.39656	0.209567	2.23769	1.61527	0.393295	0.53768	0.954872
TCONS_00119134	XLOC_009394	-	lincRNA	1390	chr16	86750494	86753100	+	2	1.45702	1.01171	0.64647	1.00404	1.1758	0.650727	1.08341	1.96588	0.72911
TCONS_00112858	XLOC_009394	-	lincRNA	1369	chr16	86751612	86771901	+	3	1.64006	1.41551	0.88752	0.526782	2.10833	0.846782	0.93295	1.24033	0.91244
TCONS_00119135	XLOC_009394	-	lincRNA	1723	chr16	86770159	86772526	+	2	1.01841	1.00509	0.23688	0.493067	0.778655	0.551586	0.782938	1.69354	0.666282
TCONS_00113558	XLOC_009401	-	lincRNA	240	chr16	87530607	87531224	+	2	0	1.62078	1.69891	1.41089	0	0	0	0	0
TCONS_00119147	XLOC_009416	-	intronic_lncRNA	2157	chr16	89181335	89184058	+	2	1.13266	1.4278	1.60888	1.06047	1.03542	2.05859	1.00409	1.56156	1.31293
TCONS_00114263	XLOC_009416	-	intronic_lncRNA	674	chr16	89183749	89184530	+	2	0.48967	1.42535	1.55948	1.21274	2.23049	1.34907	0.909672	0	0.817854
TCONS_00117160	XLOC_009416	-	intronic_lncRNA	638	chr16	89184172	89184971	+	2	1.39285	0.6519	1.03466	1.99719	0.567169	2.68925	0.965636	0.746818	1.31043
TCONS_00110760	XLOC_009996	-	intronic_lncRNA	511	chr16	89493196	89497892	-	2	1.63143	0.46139	0.91873	1.65484	1.40534	0.911044	0.388037	2.12222	1.13085
TCONS_00125915	XLOC_010687	-	intronic_lncRNA	679	chr17	548986	550089	-	2	3.61011	4.31705	2.65738	0.938144	3.36726	4.8657	1.73922	1.02244	3.64805
TCONS_00121438	XLOC_010688	-	lincRNA	1638	chr17	623986	635441	-	2	1.56085	0.68894	1.70002	0.623356	1.11275	0.831915	0.650897	0.728283	0.281344
TCONS_00133475	XLOC_010708	-	lincRNA	933	chr17	1951888	1953169	-	2	4.58657	1.74408	1.82124	5.16015	1.01139	2.11575	2.81617	1.76955	2.05772
ENST00000576086.1	ENSG00000262823.1	RP13-580F15.2	anti-sense_lncRNA	854	chr17	4385261	4389648	-	2	0.93433	0.65218	0.75314	0.784443	1.04008	0.862672	0.542184	0.248299	1.1542
TCONS_00125962	XLOC_010742	-	lincRNA	344	chr17	4704530	4710344	-	2	0.69782	0	0.51113	0	0.441719	0	2.18504	0	0.414496
TCONS_00130474	XLOC_010052	-	anti-sense_lncRNA	2696	chr17	4890981	4893960	+	3	3.39454	1.72661	2.73526	1.30753	2.92927	2.50724	1.27475	1.66413	2.01123
ENST00000574260.1	ENSG00000262227.1	RP5-1050D4.5	anti-sense_lncRNA	551	chr17	4891001	4891741	+	2	0	0	0	0	0	0.0002083	0.755618	1.47502	0
TCONS_00125521	XLOC_010052	-	anti-sense_lncRNA	2448	chr17	4891019	4893560	+	2	0.00021	0	0	0	0	0	0	0	0
TCONS_00123970	XLOC_010748	-	lincRNA	309	chr17	4934278	4935102	-	2	0	1.34364	0.68243	1.18362	0	0	0	0.78999	0
ENST00000573187.1	ENSG00000233223.2	AC113189.5	anti-sense_lncRNA	679	chr17	7485790	7486836	-	2	1.27416	2.53068	2.50975	1.60825	1.2951	1.03212	0.993839	0.511219	0.851211
TCONS_00124607	XLOC_010100	-	lincRNA	491	chr17	7621047	7622369	+	2	3.83397	0.98738	2.70542	0.663488	0.429659	0.487103	0.83166	1.70459	1.41172

TCONS_00127300	XLOC_010117	-	lincRNA	313	chr17	8771299	8772137	+	2	1.76416	0	0	0	1.13102	0	0	0	0.530463
TCONS_00125592	XLOC_010133	-	lincRNA	328	chr17	12950511	12951249	+	2	0	0	0	1.00989	0	1.11542	1.48597	0	0
TCONS_00124058	XLOC_010831	-	anti-sense_lncRNA	803	chr17	13795965	13972787	-	3	0	2.0699	0.59995	1.1117	0.635127	0.641486	0	0	0.361758
TCONS_00126879	XLOC_010831	-	anti-sense_lncRNA	3498	chr17	13934267	13972965	-	3	3.41626	2.24567	4.24118	3.17398	2.67644	3.89362	2.03059	3.27373	3.90051
ENST00000602539.1	ENSG00000236088.5	COX10-AS1	anti-sense_lncRNA	774	chr17	13937168	13972796	-	2	1.71288	6.51804	3.91946	4.12865	2.59587	2.93196	3.30086	1.48606	5.79186
TCONS_00129400	XLOC_010851	-	lincRNA	302	chr17	16836061	16836489	-	2	0	1.43533	0.73039	1.2628	1.25357	2.09473	0.629103	0	0
ENST00000579168.2	ENSG00000233098.4	RP11-344E13.3	lincRNA	638	chr17	20859130	20861639	+	3	3.48212	3.42245	5.98416	0.43986	1.8433	2.25862	4.35799	3.73408	3.72765
TCONS_00122493	XLOC_010197	-	lincRNA	2475	chr17	20863582	20886156	+	2	5.83308	1.45989	4.4111	0.450087	2.07999	2.9984	3.10175	3.56792	5.22597
ENST00000456235.1	ENSG00000236819.1	AC087393.1	lincRNA	750	chr17	20978869	20993928	+	2	0.3701	0.12946	0.25653	0.11666	0.112617	0.256642	0.86267	0.592118	0.317233
TCONS_00125626	XLOC_010208	-	lincRNA	401	chr17	26176824	26193002	+	2	0	1.44087	1.44332	0.321031	0	0.353903	0	0	1.47199
TCONS_00130587	XLOC_010254	-	lincRNA	232	chr17	31204772	31205341	+	2	0	0	0	0	0	0	3.48841	2.30316	0
ENST00000590478.1	ENSG00000266947.1	RP11-799D4.4	lincRNA	2658	chr17	33558469	33569982	-	2	1.78588	1.58091	2.91942	1.11948	1.22486	1.25873	1.96846	1.6343	2.37105
TCONS_00123562	XLOC_010269	-	lincRNA	1904	chr17	33640429	33653083	+	3	0.71864	1.49412	0.85996	0.601075	0.64971	0.412854	0.959991	0.897342	1.3559
ENST00000592381.1	ENSG00000267321.1	RP11-1094M14.11	anti-sense_lncRNA	1298	chr17	33895139	33901811	+	2	3.80785	4.19403	4.27016	3.90855	3.59166	3.20627	3.31191	4.41105	3.63635
TCONS_00129091	XLOC_010280	-	anti-sense_lncRNA	3567	chr17	34404118	34420635	+	3	0.03862	0.04774	1.51529	3.38032	2.83914	5.84555	1.44312	1.2365	1.72882
TCONS_00130593	XLOC_010280	-	anti-sense_lncRNA	4703	chr17	34416126	34421050	+	2	0.53811	0.30915	0.55141	0.612384	0.208841	1.12407	0.39445	0.999685	0.308022
TCONS_00126956	XLOC_010957	-	lincRNA	498	chr17	34444140	34476429	-	3	0.68083	0.72283	3.35999	0	0.209687	0.713345	0.811323	0.277215	0.393699
ENST00000561550.2	ENSG00000260833.2	AC124789.1	intronic_lncRNA	702	chr17	36606638	36608643	-	4	0.54285	3.15853	2.43743	0.621297	1.6722	2.20098	1.86282	1.14633	2.05278
ENST00000581922.1	ENSG00000260833.2	AC124789.1	intronic_lncRNA	401	chr17	36607300	36607932	-	2	0	0.39928	0	0	0.940954	0.353903	0	0	0.382356
TCONS_00129463	XLOC_010965	-	intronic_lncRNA	365	chr17	36607779	36608633	-	2	2.02855	2.0475	3.40595	0.446694	0.56113	0.152018	1.99097	2.53473	0
TCONS_00121817	XLOC_010967	-	intronic_lncRNA	1208	chr17	36701138	36703346	-	2	1.82069	0.91411	0.69474	0.444884	0.672745	0.48906	0.524205	0.400714	1.03378
TCONS_00123586	XLOC_010305	-	intronic_lncRNA	287	chr17	37241750	37242737	+	2	1.12673	1.67647	0.85721	0	0	0	0	0	0
TCONS_00133880	XLOC_010308	-	lincRNA	1684	chr17	37707254	37724349	+	4	2.88758	2.43161	3.4352	2.28661	2.90266	2.89241	2.0099	2.71371	2.80379
TCONS_00121847	XLOC_010991	-	intronic_lncRNA	1987	chr17	38496838	38499119	-	2	0.34266	1.10797	0.78078	2.04177	0.997982	0.944786	0.653643	0.720381	0.775658
TCONS_00125696	XLOC_010326	-	lincRNA	1507	chr17	38679624	38683918	+	2	0.31219	0.70411	0.85557	0.391957	0.471066	0.753911	0.761539	0.740125	1.59255
TCONS_00126147	XLOC_011002	-	lincRNA	350	chr17	38873626	38875362	-	3	0	0	0.48979	0.430936	0.423606	5.23166	0.418419	0	0.397517
TCONS_00133029	XLOC_011025	-	lincRNA	436	chr17	39784242	39785060	-	3	8.15094	3.9736	6.71874	0	2.12864	0.902719	4.40082	0.705826	1.99813
TCONS_00129126	XLOC_010361	-	lincRNA	356	chr17	41480016	41485136	+	2	1.29055	1.86802	0.94024	0.828607	2.44135	0.914341	0	0.543763	0
TCONS_00123635	XLOC_010363	-	anti-sense_lncRNA	558	chr17	41613502	41621722	+	2	0.85112	1.79956	0.39756	0.538671	0.347973	0.197645	0.167674	0.688603	0.163346
TCONS_00121078	XLOC_010364	-	lincRNA	276	chr17	41756587	41757692	+	2	1.2724	0	0	0.833956	1.66893	0	0	0	0
ENST00000591137.1	ENSG00000267405.1	CTC-296K1.4	lincRNA	587	chr17	42872115	42875151	-	2	2.10052	0.18476	0.36708	1.16225	0.482267	0.365469	3.40359	0.63574	0.150929
ENST00000585471.1	ENSG00000267121.1	CTD-2020K17.1	anti-sense_lncRNA	1077	chr17	43295623	43297792	-	2	1.04599	1.75743	0.5597	1.68058	0.562828	1.12466	0.804798	1.10691	1.32131
ENST00000591365.1	ENSG00000267121.1	CTD-2020K17.1	lincRNA	2238	chr17	43296717	43299589	-	2	1.00165	2.40259	2.59999	2.638	1.71929	1.86357	1.31712	2.40663	2.91768
ENST00000589518.1	ENSG00000233483.2	CTD-2020K17.4	lincRNA	393	chr17	43324634	43325210	-	2	0.52338	1.50208	0.75285	1.67219	0.981001	1.10621	0.960239	0.435183	1.22768
TCONS_00130975	XLOC_011095	-	lincRNA	249	chr17	43441023	43442271	-	2	0	0	0	0	0	0	0	0	1.1481
TCONS_00127848	XLOC_011111	-	lincRNA	286	chr17	45713704	45726783	-	2	7.97078	1.69518	0	1.48693	2.22436	0	0.749615	1.0043	0
TCONS_00134137	XLOC_011121	-	anti-sense_lncRNA	688	chr17	46187383	46195749	-	3	0.62538	1.46087	1.30374	0.263079	0.127094	0.578847	1.09693	3.00965	1.79
TCONS_00133912	XLOC_010418	-	anti-sense_lncRNA	1327	chr17	46622664	46626800	+	2	1.53831	4.08559	1.48994	0.738828	0.656019	0.187415	1.97708	1.4322	1.43746
ENST00000511322.1	ENSG00000250948.1	RP11-1079K10.2	lincRNA	245	chr17	47452742	47457456	+	2	0	0	0	1.29457	0	0	0	1.80063	0
TCONS_00132068	XLOC_010457	-	lincRNA	637	chr17	50466936	50609979	+	3	0	0.3267	0.9731	0.146957	7.08E-06	0	1.38429	0	1.2015
TCONS_00130684	XLOC_010457	-	lincRNA	488	chr17	50467223	50468176	+	2	1.40858	0	0.99369	0	0.434211	1.47666	1.86198	2.29723	1.62968
TCONS_00127471	XLOC_010458	-	lincRNA	493	chr17	50816824	50819679	+	2	0.69236	0	0.97687	0.65893	0	0	0	1.41049	0
TCONS_00129174	XLOC_010464	-	lincRNA	493	chr17	53900053	53920344	+	2	0.69236	0.24513	0.48844	0.219643	0.426669	0.967498	0.206437	0	1.00136
TCONS_00121186	XLOC_010466	-	intronic_lncRNA	1114	chr17	54369380	54372750	+	2	2.45361	0.93109	1.45708	0.420628	0.404926	0.462429	0.514522	0.707759	1.45762
TCONS_00122022	XLOC_011164	-	lincRNA	2056	chr17	54900939	54903221	-	2	1.86958	2.43804	1.20259	1.06911	0.761987	1.02329	0.943775	1.17021	1.74233
ENST00000576313.1	ENSG00000263004.1	RP11-166P13.3	lincRNA	449	chr17	55156508	55162385	-	2	4.88167	3.18307	5.48906	1.03495	2.77069	1.71026	7.58582	2.33667	3.78308
TCONS_00123179	XLOC_011173	-	lincRNA	1536	chr17	56124338	56138101	-	6	4.39235	2.33985	2.3684	0.142557	0.649354	0.880775	1.40427	1.37427	0.840254
TCONS_00122042	XLOC_011173	-	lincRNA	2395	chr17	56127929	56138107	-	6	4.71897	1.92653	1.51526	0.292315	0.837424	0.713852	2.2043	0.484114	0.935671
TCONS_00129181	XLOC_010478	-	lincRNA	406	chr17	56297081	56310086	+	2	0.9815	0.35127	3.51717	0.313181	2.14091	0.690444	1.19499	0.813115	1.1483
ENST00000593015.1	ENSG00000267302.1	RP11-178C3.2	lincRNA	692	chr17	58042193	58050465	+	3	1.03386	1.59375	1.4366	0.391398	1.26048	0.717645	1.81297	1.65817	0.355056
TCONS_00126252	XLOC_011212	-	lincRNA	264	chr17	61532351	61536847	-	2	1.47557	0	2.29926	0.972287	0.977243	0	1.99969	0	0
ENST00000577544.1	ENSG00000264754.1	CTD-2653B5.1	intronic_lncRNA	612	chr17	65520597	65521538	+	2	0.98686	0.86698	1.03297	0.623661	0.60346	1.88742	0.14504	0.993792	0.566585
TCONS_00133407	XLOC_010538	-	lincRNA	2599	chr17	66610928	66654909	+	2	7.02119	2.44454	2.84626	1.68054	3.27851	1.81734	1.02071	2.8134	1.51543
TCONS_00123787	XLOC_010539	-	lincRNA	613	chr17	66677443	66678259	+	2	1.96897	2.07564	0.51521	0.311072	0.451484	0.68463	0.434034	1.38786	0.282597
TCONS_00132845	XLOC_010538	-	anti-sense_lncRNA	1633	chr17	66817852	66871541	+	3	2.42063	1.53084	1.26731	1.34043	0.68717	1.22768	0.857308	1.68656	0.967987
TCONS_00122784	XLOC_010540	-	anti-sense_lncRNA	241	chr17	67008117	67008505	+	2	8.30464	4.77703	3.33576	0	0	0	0	0	0
TCONS_00133712	XLOC_011247	-	lincRNA	633	chr17	68193759	68204704	-	2	0.93923	0.32976	0.32728	0.148321	0.143451	0	0.413414	1.13344	1.34688
TCONS_00123284	XLOC_011249	-	lincRNA	1107	chr17	70017598	70030391	-	4	3.59636	1.09517	0	2.18417	2.87173	7.82363	0.453707	4.50802	0.872202
ENST00000543512.1	ENSG00000228639.2	AC005152.3	lincRNA	323	chr17	70017992	70030233	-	2	0	0.59139	0	1.61971	4.03514	6.53954	0.0001665	1.70012	0.654028
TCONS_00130407	XLOC_011249	-	lincRNA	5924	chr17	70048341	70054419	-	2	2.0877	0.60405	0.14576	1.26276	3.41972	6.70337	0.40623	4.0196	1.21823
TCONS_00123793	XLOC_010547	-	lincRNA	226	chr17	70419740	70420167	+	2	0	2.12428	0	0	0	0	3.96798	0	0

TCONS_00121284	XLOC_010548	-	intronic_lncRNA	1754	chr17	70770204	70772575	+	2	3.28296	2.77693	3.50421	0.411978	0.752244	0.588603	2.06914	1.29537	1.41288
TCONS_00126301	XLOC_011271	-	intronic_lncRNA	532	chr17	72725994	72727011	-	2	1.83469	1.2952	2.57718	0.581094	0.563539	0.63973	1.08776	0.496045	1.58718
TCONS_00129642	XLOC_011302	-	lincRNA	490	chr17	74116187	74117085	-	2	0	0	1.23411	0	1.07792	1.22199	0.625987	0.57022	0.404767
TCONS_00127545	XLOC_010607	-	lincRNA	296	chr17	75662995	75673404	+	2	0	0.09886	0.22362	2.35181	1.00676	0	0	0	0.624239
TCONS_00126778	XLOC_010607	-	lincRNA	804	chr17	75663011	75664085	+	2	0.5057	0.45582	1.4826	0.582735	0.254872	0.116799	0.29399	0.269176	0
TCONS_00122252	XLOC_011317	-	lincRNA	1135	chr17	75883944	75889962	-	4	2.39479	0.64426	1.84465	0.0835981	0.10332	0.301343	0.628519	0.193851	0.185829
TCONS_00126342	XLOC_011317	-	lincRNA	1233	chr17	75886945	75889943	-	2	0.49701	0.65207	0.97521	0.296431	0.443569	0.409024	0.284094	0.606563	0.224139
TCONS_00128395	XLOC_010618	-	lincRNA	1638	chr17	76356527	76361808	+	3	0.56758	0.29526	0.24286	1.87007	0.213991	0.978723	0.406811	0.112044	0.522496
TCONS_00128019	XLOC_011384	-	lincRNA	285	chr17	80345285	80346277	-	2	1.15087	0.85714	0	2.25508	0	0	0	0	0
ENST00000578344.1	ENSG00000265458.1	RP13-20L14.6	anti-sense_lncRNA	210	chr17	80415242	80415575	-	2	0	0	0	0	2.76983	2.99218	0	3.85668	0
TCONS_00121430	XLOC_010676	-	intronic_lncRNA	472	chr17	80896363	80896962	+	2	3.72692	0.26443	2.10948	1.18357	2.07132	1.04289	1.56164	0.913875	2.16048
TCONS_00132177	XLOC_010677	-	lincRNA	3357	chr17	81054348	81058337	+	2	1.52011	1.76712	0.90408	4.94507	1.90634	2.73899	1.29116	1.37308	2.30187
TCONS_00125909	XLOC_010678	-	lincRNA	377	chr17	81091942	81092925	+	2	1.70995	0	0.412	0.364892	1.07217	0.80488	2.10514	0	0.335425
TCONS_00137109	XLOC_011623	-	lincRNA	261	chr18	2441764	2443245	-	2	0	0	0	0	0	0	0	2.78341	0
TCONS_00135425	XLOC_011421	-	intronic_lncRNA	367	chr18	6256802	6258948	+	2	1.81062	2.17767	0	0	0	0	0	0.506306	0
ENST00000583691.1	ENSG00000266604.1	RP11-856M7.6	lincRNA	631	chr18	10612399	10617010	-	2	0	0.16565	0.16442	0	0	0	0	0	0
ENST00000589930.1	ENSG00000267654.1	RP11-973H7.4	lincRNA	569	chr18	12739489	12749420	-	2	0	0.1939	0.38542	0.174177	0	0	0	0.222516	0.316806
TCONS_00137145	XLOC_011659	-	intronic_lncRNA	575	chr18	13600744	13608677	-	2	0.54184	0	1.51643	0	0.331957	0	0.319645	0.437732	0.155831
TCONS_00135733	XLOC_011679	-	lincRNA	1687	chr18	21543202	21563071	-	2	2.17478	1.59186	0.95525	0.0933918	0.58462	1.076	0.729375	0.905198	1.0116
TCONS_00137159	XLOC_011679	-	lincRNA	302	chr18	21547639	21562571	-	2	3.21159	1.10883	5.59405	1.78745	1.17384	0.880861	0.915219	3.62599	1.74575
TCONS_00135734	XLOC_011680	-	intronic_lncRNA	382	chr18	21574347	21574842	-	2	2.77279	0.79758	1.60075	0.709601	0.347309	1.56508	1.70315	0.92543	3.58567
TCONS_00135494	XLOC_011478	-	anti-sense_lncRNA	3317	chr18	24763055	24771567	+	2	6.0349	2.12401	5.43334	0	2.20181	1.82003	2.47809	1.78459	1.28293
TCONS_00135986	XLOC_011478	-	anti-sense_lncRNA	3552	chr18	24763055	24771567	+	3	2.46044	1.41664	3.08827	0.668039	1.14031	0.908171	2.81991	0.519779	1.14695
TCONS_00137738	XLOC_011479	-	lincRNA	1463	chr18	24815205	24846792	+	4	6.05713	4.26038	6.36517	1.52112	2.389	1.83911	2.41135	1.65989	1.19042
TCONS_00140529	XLOC_011692	-	lincRNA	722	chr18	26646773	26695729	-	3	0.19494	0.13647	0.27053	0	0.118725	0	0.910036	0.468342	3.79022
TCONS_00135504	XLOC_011492	-	intronic_lncRNA	3640	chr18	30530594	30541147	+	3	1.39513	0.1642	2.44808	0.037197	0.749681	0.408698	1.31995	1.0965	1.72664
TCONS_00137004	XLOC_011496	-	intronic_lncRNA	5549	chr18	32657969	32663834	+	2	1.43207	1.01585	1.91093	3.09727	1.26197	1.65543	1.36954	1.58885	1.79937
ENST00000593122.1	ENSG00000267583.1	RP11-322E11.5	lincRNA	364	chr18	33023833	33046716	-	3	0.61438	0	0.44618	2.36399	0	0	0.380499	0	0
ENST00000568654.1	ENSG00000260552.1	RP11-49I11.1	lincRNA	609	chr18	33759959	33767411	-	2	0.74555	1.92153	3.2956	2.04183	0.911923	1.55558	2.92272	0.60075	2.28319
TCONS_00139083	XLOC_011720	-	lincRNA	3032	chr18	36169402	36308390	-	4	0.57732	0.20914	0.59026	0.0903762	0.45086	0.869067	0.908227	1.31275	0.980416
TCONS_00137599	XLOC_011720	-	lincRNA	656	chr18	36174165	36308377	-	2	0.35839	0	1.18729	0	0.516391	1.19506	1.71115	0.570824	1.89085
TCONS_00137191	XLOC_011720	-	lincRNA	3517	chr18	36272082	36308351	-	2	0.56578	0.3324	2.14765	0.0192885	0.896365	1.51066	0.715314	1.89992	1.63558
TCONS_00139507	XLOC_011509	-	lincRNA	466	chr18	37445231	37446028	+	2	0.76206	0	0	0	0.235447	0.266657	1.14177	1.87042	0.221022
TCONS_00135525	XLOC_011510	-	lincRNA	432	chr18	37448857	37451152	+	2	2.1816	0	1.24343	0	0	0.306148	0.263566	0.359237	0
TCONS_00137020	XLOC_011513	-	lincRNA	438	chr18	39739056	39739765	+	2	0	1.21215	0.90821	1.89548	1.84655	2.38682	0	0.699667	0.742859
TCONS_00138810	XLOC_011732	-	anti-sense_lncRNA	542	chr18	42987960	43084017	-	3	1.09624	4.00167	3.32379	11.3128	2.64465	3.42142	1.93529	1.89424	1.36491
TCONS_00136843	XLOC_011732	-	intronic_lncRNA	1717	chr18	43071047	43084422	-	3	1.70215	3.53957	2.62566	6.44986	1.19614	1.59877	1.15605	1.38694	0.762714
TCONS_00139632	XLOC_011735	-	anti-sense_lncRNA	364	chr18	43327120	43348122	-	2	1.84317	0	1.78497	0.393999	0.386535	0	1.14172	1.03203	0.362746
TCONS_00137602	XLOC_011735	-	lincRNA	239	chr18	43359383	43362755	-	3	6.44418	0	1.73088	0	0	1.59672	4.5526	0	0
TCONS_00137196	XLOC_011736	-	anti-sense_lncRNA	1067	chr18	43379867	43410222	-	2	1.88094	0.49094	2.02221	0.147827	0.355852	0.568835	0.74636	0.279954	0.601492
TCONS_00138249	XLOC_011752	-	intronic_lncRNA	1824	chr18	44616851	44624604	-	2	2.38765	0.87109	1.37532	0.946078	1.43941	1.03974	0.791713	1.48705	1.67193
TCONS_00137913	XLOC_011773	-	lincRNA	1620	chr18	52270794	52274388	-	2	0.57477	0.29902	0.29515	0.270544	0.520115	0.545134	0.947626	0.851049	1.05829
TCONS_00137053	XLOC_011552	-	lincRNA	396	chr18	56327117	56327930	+	2	0.51547	0.73927	1.11127	1.64641	1.60932	0.363031	0.629788	0	1.51051
TCONS_00136915	XLOC_011795	-	lincRNA	1034	chr18	59567595	59685462	-	3	0.34941	0.95336	0.28855	1.22565	1.39574	2.03189	0.46981	0.857733	0.291732
TCONS_00135854	XLOC_011795	-	lincRNA	1334	chr18	59567782	59578190	-	2	1.90121	0.23705	0.7138	0.624135	0.550903	1.42488	0.379667	0.652018	0.193981
ENST00000589905.1	ENSG00000267390.1	RP11-635N19.1	anti-sense_lncRNA	785	chr18	61034561	61048862	+	2	0.87011	2.55437	2.04807	1.75453	0.740696	1.68853	2.02493	1.66836	2.0865
TCONS_00137932	XLOC_011814	-	lincRNA	1263	chr18	71314549	71358512	-	3	0.57556	0.19998	0.06585	2.53095	0.927637	1.39108	1.32472	1.06343	0.980011
TCONS_00137933	XLOC_011814	-	lincRNA	1046	chr18	71350501	71358527	-	2	0.24094	0.16774	0.08292	1.66655	0.948336	0.832855	0.278238	0.765281	0.411014
TCONS_00135624	XLOC_011593	-	intronic_lncRNA	1492	chr18	72722987	72724598	+	2	1.65799	1.53423	1.94754	1.28867	1.14374	1.9067	0.95175	1.93435	2.10341
TCONS_00137087	XLOC_011595	-	lincRNA	460	chr18	73168370	73170270	+	2	0	0.27688	1.65759	0.495431	0	0	0.233869	0.319187	0.22623
TCONS_00140069	XLOC_011604	-	lincRNA	264	chr18	76201919	76203401	+	2	0	0	1.14963	0	0.977243	0	0	3.99702	0
TCONS_00138428	XLOC_011609	-	lincRNA	335	chr18	77341580	77343751	+	2	0	0	0	0	1.41569	0	0	0	0
ENST00000589457.1	ENSG00000267751.1	AC009005.2	anti-sense_lncRNA	719	chr19	567212	571745	-	2	0.39212	0.41181	0.13606	0	0.477674	0.408074	0.915422	0.157033	0.11213
ENST00000606242.1	ENSG00000267530.2	AC006273.5	lincRNA	710	chr19	782755	784948	+	2	9.38275	11.9096	7.37607	4.37648	3.51648	4.86718	3.98328	2.34876	1.66346
ENST00000586061.1	ENSG00000267530.2	AC006273.5	lincRNA	829	chr19	782796	785080	+	2	3.55669	4.94715	1.31444	1.55221	2.76049	1.21579	1.58034	0.556281	1.23971
TCONS_00145657	XLOC_012657	-	lincRNA	339	chr19	2028705	2034959	-	2	0.72227	0.52543	2.12136	0.465089	0.458006	0.513477	0.907522	0.613584	0
TCONS_00150513	XLOC_012739	-	lincRNA	244	chr19	7448182	7459936	-	3	0	7.55888	3.16026	0	0	1.46288	6.91501	0	2.49777
TCONS_00149507	XLOC_012744	-	lincRNA	281	chr19	7771168	7771931	-	2	0	0	0	0	0.785484	1.74163	0	0	0
TCONS_00145718	XLOC_012771	-	lincRNA	632	chr19	9146316	9176438	-	2	1.64745	0.82633	1.31222	0.297331	1.1503	2.94452	1.2432	1.13613	0.270007
TCONS_00144633	XLOC_011973	-	lincRNA	4373	chr19	9820337	9834259	+	2	1.27256	1.55621	1.86712	0.337263	0.456009	1.26324	1.26866	0.672796	0.746009
ENST00000426044.1	ENSG00000234773.2	CTD-2666L21.1	lincRNA	917	chr19	12305832	12348582	+	3	1.98911	3.26939	1.4703	0.804753	0.775604	1.96649	1.56348	1.13097	1.13292

TCONS_00145766	XLOC_012821	-	lincRNA	670	chr19	12754689	12755756	-	2	0.86563	0.75874	0.30103	0.273195	0.528082	0.450854	0.380014	1.21615	1.11562
TCONS_00146759	XLOC_012023	-	lincRNA	572	chr19	13842589	13843254	+	2	2.45782	3.46168	2.48456	1.38212	1.84071	1.33108	0.805771	1.54473	2.19949
TCONS_00147176	XLOC_012857	-	lincRNA	287	chr19	14477515	14480783	-	2	1.12673	0	0	1.4708	0	0	0	0	0.687349
TCONS_00150233	XLOC_012042	-	lincRNA	405	chr19	15674435	15675252	+	2	0	0	0	0	0.307375	0.693848	1.80173	0	0
TCONS_00152386	XLOC_012886	-	lincRNA	271	chr19	16403733	16435339	-	2	0	2.02839	2.08786	0.887044	0	0	0.906072	1.20959	3.33267
TCONS_00143168	XLOC_012949	-	lincRNA	546	chr19	20411838	20432181	-	2	6.75018	0.62099	0.20585	0.185818	0	2.04551	0	0	0
TCONS_00141230	XLOC_012142	-	intronic_lncRNA	587	chr19	23281169	23283391	+	2	2.10052	0.55427	0.55062	0	0.160756	1.27914	0.309417	0.423827	0.150929
TCONS_00152121	XLOC_012145	-	lincRNA	1368	chr19	23956799	23963169	+	3	4.19129	1.75852	2.21575	0.712852	2.58426	3.4356	0.602197	0.897989	2.32778
TCONS_00152122	XLOC_012146	-	lincRNA	917	chr19	23969220	23970232	+	2	2.41534	0.99072	1.27426	0.357668	1.12032	1.47487	0.740595	0.90478	1.13292
TCONS_00144724	XLOC_012145	-	lincRNA	976	chr19	24216162	24241206	+	4	6.30271	8.96568	5.42895	5.86475	5.65002	7.62919	5.46719	6.26365	5.90336
TCONS_00143183	XLOC_012967	-	lincRNA	257	chr19	27731866	27740264	-	3	30.8333	35.67	0	0	1.08165	0	17.7826	0	0
TCONS_00143182	XLOC_012967	-	lincRNA	403	chr19	27731895	27732965	-	2	53.277	52.4179	13.2138	3.49629	6.20963	2.45265	14.564	40.0442	7.57702
TCONS_00148541	XLOC_012971	-	lincRNA	459	chr19	31647090	31719012	-	3	0.39124	0	0	0	0.96779	0.547867	1.40889	0.320463	0.681365
TCONS_00149283	XLOC_012170	-	lincRNA	265	chr19	33301000	33302196	+	2	1.45646	0	0	0	0.963747	2.12736	0	0	0
ENST00000592220.1	ENSG00000267024.1	CTD-2588C8.8	lincRNA	442	chr19	34917017	34919178	-	2	1.25539	0.89376	0.29756	0	0	0	0.252135	0	0
TCONS_00150925	XLOC_012231	-	intronic_lncRNA	1034	chr19	37064042	37066370	+	2	2.32177	1.4778	2.1574	1.93067	1.48016	2.66115	2.06909	1.35329	1.32541
ENST00000448373.2	ENSG00000233527.4	AC092295.7	intronic_lncRNA	1004	chr19	37064279	37068817	+	3	1.64769	2.5275	1.77686	1.50474	1.15185	2.67355	1.0756	1.51639	1.14849
ENST00000425254.2	ENSG00000225975.2	AC074138.3	lincRNA	815	chr19	37176367	37178312	-	3	2.31786	3.12256	2.29025	2.92019	2.11269	1.72052	2.40522	1.98215	2.17273
TCONS_00145421	XLOC_012248	-	lincRNA	534	chr19	38308002	38310936	+	3	0.60793	0.85821	1.06722	0	0.186702	1.27174	0.360334	0.246495	0.876399
TCONS_00143236	XLOC_013074	-	anti-sense_lncRNA	3066	chr19	41485436	41497381	-	3	7.7325	0.00026	1.89745	0.0001955	0.0004367	0.0062595	4.47937	1.71414	0.128819
TCONS_00146566	XLOC_013074	-	anti-sense_lncRNA	3068	chr19	41485492	41497381	-	3	3.70946	3.20922	13.965	4.70665	13.5689	6.06891	0.559245	0.0238409	0.128101
TCONS_00150690	XLOC_013074	-	lincRNA	738	chr19	41485722	41496278	-	2	2.97777	0.75132	7.17869	1.91165	5.11376	1.07289	5.85549	0.600673	0.34934
ENST00000594315.1	ENSG00000267107.2	AC011526.1	lincRNA	1062	chr19	41961601	42006559	-	3	3.54716	2.71594	1.87133	5.57572	2.50551	3.10593	2.5935	1.59571	1.74779
TCONS_00144364	XLOC_013082	-	lincRNA	1014	chr19	42005807	42006924	-	2	1.00154	1.56936	0.77591	2.28337	0.682531	1.38512	0.723351	0.397857	0.142428
TCONS_00143765	XLOC_012321	-	lincRNA	5318	chr19	42039173	42047238	+	2	0.90366	1.78207	1.61168	3.00823	1.41895	2.88068	1.45467	2.77152	2.76607
TCONS_00149929	XLOC_012321	-	lincRNA	3734	chr19	42039291	42043950	+	3	0.71417	0.33482	0.26355	0.331533	0.0636556	0.700478	0.131107	0.683447	0.838297
ENST00000601116.1	ENSG00000268027.1	AC006129.2	lincRNA	601	chr19	42055888	42057073	+	2	3.54825	8.01978	12.2123	10.574	3.25632	8.46302	7.30791	11.0343	15.2865
TCONS_00147655	XLOC_012322	-	lincRNA	2120	chr19	42055895	42073129	+	2	1.11629	0.76216	3.88252	2.44988	1.36293	2.83054	1.80887	3.38529	3.4163
TCONS_00143767	XLOC_012322	-	lincRNA	1276	chr19	42061702	42071557	+	2	2.65355	2.91719	3.12942	2.29261	2.14419	1.68414	1.82679	3.01523	4.53934
TCONS_00141422	XLOC_012325	-	lincRNA	1250	chr19	42254416	42256085	+	2	0.874	1.01233	1.6668	0.366049	0.293495	0.469449	0.726546	0.922901	0.82684
ENST00000593491.2	ENSG00000213904.4	LIPE-AS1	anti-sense_lncRNA	292	chr19	42901280	42912604	+	2	7.49224	5.55903	6.48578	5.57908	4.16536	4.63016	6.9987	4.69436	5.85827
TCONS_00150359	XLOC_012340	-	lincRNA	1042	chr19	42989256	42994046	+	2	1.57352	0.33708	0.58321	0.380564	1.17276	0.920506	0.279579	0.865077	1.03245
ENST00000595748.1	ENSG00000231412.2	CTC-490G23.2	lincRNA	301	chr19	43833447	43835582	-	2	0	0.72469	0.73775	1.27493	1.89892	1.40999	0	0	0.593612
TCONS_00141489	XLOC_012381	-	lincRNA	434	chr19	45690624	45691202	+	2	1.73051	0.925	0.92431	0.826336	0.805223	1.51752	0	1.78024	1.25975
ENST00000602172.1	ENSG00000268001.1	CTC-241F20.3	anti-sense_lncRNA	1969	chr19	48758932	48761456	+	2	2.88449	2.83814	4.73261	6.58575	4.6932	4.89126	7.42882	7.3684	6.92138
TCONS_00145568	XLOC_012481	-	lincRNA	298	chr19	50998586	50999843	+	2	0	0.74656	4.56414	1.31267	1.30449	1.45199	1.31156	1.76161	1.22329
TCONS_00148692	XLOC_013278	-	lincRNA	1314	chr19	52897979	52901003	-	2	2.60205	2.10283	2.32828	0.747465	2.10689	1.46729	1.60571	1.00777	0.596908
ENST00000598892.1	ENSG00000269834.1	CTD-3018O17.3	lincRNA	837	chr19	52899892	52900922	-	2	1.21107	0.32735	0.98702	0.20136	0.476493	0.972618	0.417483	0.649116	0.410994
TCONS_00145583	XLOC_012510	-	lincRNA	515	chr19	53400773	53401477	+	2	0	0	0.68005	0.61264	0.594502	2.24845	0.191459	0	0.186039
TCONS_00141613	XLOC_012515	-	lincRNA	1089	chr19	53929410	53930648	+	2	1.03191	1.11701	0.63093	0.648723	1.38796	0.396226	0.992231	0.454921	0.71685
TCONS_00145590	XLOC_012518	-	lincRNA	270	chr19	54016241	54016660	+	2	0	2.05473	3.17393	0	0	1.99156	0	0	1.68822
TCONS_00149416	XLOC_012521	-	lincRNA	222	chr19	54383860	54384231	+	2	0	0	0	0	0	2.24343	2.17363	0	3.85728
TCONS_00143958	XLOC_012567	-	lincRNA	1146	chr19	56826600	56828060	+	2	0.96987	0.1499	0.96291	0.203186	0.456338	0.372286	0.434781	0.34179	0.428529
ENST00000434052.1	ENSG00000142396.6	ERVK3-1	lincRNA	616	chr19	58816743	58818000	+	2	8.79678	8.75703	10.2283	19.9188	11.2046	10.8746	10.0523	12.3986	10.3797
ENST00000590505.1	ENSG00000142396.6	ERVK3-1	lincRNA	3889	chr19	58820396	58826569	+	2	9.57768	6.00932	9.90314	13.5618	10.3852	9.75597	9.26077	11.0061	9.94084
TCONS_00148026	XLOC_013363	-	anti-sense_lncRNA	824	chr19	58826344	58838866	-	3	0.16318	0.79777	0.45135	0.822271	0.594839	1.1304	1.13753	1.30209	1.02401
TCONS_00148360	XLOC_012608	-	intronic_lncRNA	294	chr19	58887235	58890421	+	2	0	1.55536	2.38018	2.04954	0	2.26763	2.05377	0	0
ENST00000450917.1	ENSG00000235078.1	AC142528.1	lincRNA	974	chr2	3523046	3526968	+	2	0.13165	0.82552	0.4536	0.165632	0.478709	0.455296	0.456795	0.314001	0.149841
TCONS_00175299	XLOC_013398	-	lincRNA	377	chr2	7208111	7209221	+	2	0	0.82068	0.82399	0.364892	0.714779	0.40244	0	2.382	1.00628
TCONS_00160638	XLOC_013401	-	lincRNA	246	chr2	7806502	7807793	+	2	0	0	0	2.54647	0	0	0	0	0
ENST00000417930.1	ENSG00000235576.1	AC092580.4	lincRNA	243	chr2	7865932	7870836	+	2	2.00823	6.15148	14.4767	8.03463	2.71622	4.46435	8.44998	18.6574	8.89571
TCONS_00170590	XLOC_014411	-	lincRNA	1680	chr2	8522936	8529824	-	2	0.79516	0.74541	0.72548	0.124856	0.236615	0.709238	0.403042	1.02813	1.10391
TCONS_00161386	XLOC_014411	-	lincRNA	1172	chr2	8528727	8529994	-	2	0.78303	0.61326	0.6216	0.468898	0.463678	0.513222	0.592257	1.58865	1.05612
TCONS_00164725	XLOC_014412	-	lincRNA	432	chr2	8532063	8533114	-	2	0	0.31105	0.62172	1.11135	0	0	0.790697	2.51466	1.01669
TCONS_00177772	XLOC_014413	-	lincRNA	2238	chr2	8597767	8601351	-	2	0.75124	1.00582	0.44473	0.471071	0.30163	0.586681	0.286331	1.18359	1.10475
TCONS_00158055	XLOC_014414	-	lincRNA	8797	chr2	8615250	8624353	-	2	0.89115	0.84514	1.01849	0.49845	0.72066	0.612983	0.797335	1.34208	1.6016
TCONS_00161387	XLOC_014415	-	lincRNA	1958	chr2	8739268	8747680	-	2	0.58049	0.76427	0.35718	0.072824	0.0349827	0.320123	1.22932	0.457701	0.591353
TCONS_00161401	XLOC_014440	-	lincRNA	1021	chr2	15126427	15138577	-	4	1.54227	0	0.42801	0.099918	1.24414	0.549538	0.295359	0.24655	0.0911174
TCONS_00154096	XLOC_014440	-	lincRNA	366	chr2	15131415	15138363	-	3	5.81321	0	5.2833	3.00484	2.46315	1.54465	4.46284	3.82108	3.47851
TCONS_00154104	XLOC_014446	-	lincRNA	448	chr2	17978966	17981543	-	3	3.67588	3.7773	4.64166	0.259787	2.02336	2.00343	2.70312	1.34083	3.56126
TCONS_00176190	XLOC_013455	-	lincRNA	1936	chr2	19247890	19252116	+	2	3.93853	0.77398	2.89382	0.774353	2.33821	1.41831	0.437437	1.06612	1.1312

TCONS_00161411	XLOC_014448	-	lincRNA	614	chr2	19284308	19325147	-	2	0.59148	0.26426	1.32115	0.555409	0.541963	1.65906	0.144318	1.18666	1.12762
TCONS_00154106	XLOC_014448	-	lincRNA	2021	chr2	19324943	19430218	-	2	2.61112	1.14422	0.47072	0.366287	0.992458	1.2857	0.513086	0.618499	0.507406
TCONS_00173215	XLOC_014449	-	lincRNA	1308	chr2	19545234	19547780	-	2	5.52227	2.94116	4.73655	0.924947	5.33861	3.81272	3.17583	3.71527	3.81226
TCONS_00154119	XLOC_014456	-	lincRNA	381	chr2	20259571	20260576	-	2	1.67273	0.40105	3.21997	0	0.34928	1.57381	0.685256	1.39617	0.327819
TCONS_00164127	XLOC_013459	-	lincRNA	563	chr2	20813859	20815459	+	2	0.55965	0.59147	0.58792	0.354142	0.343104	0	0.826428	0.22629	0
TCONS_00170135	XLOC_013461	-	lincRNA	877	chr2	21782778	21788485	+	2	0.60179	0.31492	0.31166	0.378859	0.273939	1.14572	0	0.239746	0.771694
TCONS_00152947	XLOC_013467	-	lincRNA	1788	chr2	24286630	24290022	+	2	0.83532	0.44546	0.30771	0.161259	0.774822	0.487368	0.257663	0.304193	0.400205
TCONS_00165304	XLOC_013471	-	lincRNA	2783	chr2	24714141	24789615	+	2	3.83382	2.87173	3.26307	2.99762	3.02067	4.24659	1.82763	3.14094	3.43994
TCONS_00176201	XLOC_013476	-	anti-sense_lncRNA	451	chr2	25643807	25650500	+	2	0	0.28702	0.28654	2.30994	0.249906	1.131	2.18394	1.98632	2.11066
TCONS_00158887	XLOC_013481	-	anti-sense_lncRNA	1197	chr2	26521813	26531431	+	2	0	0.1846	0	0	0.247565	0	0.424904	0	0
TCONS_00175323	XLOC_013481	-	lincRNA	3245	chr2	26521873	26528819	+	2	1.40832	0.8443	1.57762	0.336202	0.988229	0.80808	0.703572	1.37101	1.26911
TCONS_00164156	XLOC_013506	-	intronic_lncRNA	283	chr2	28005525	28008023	+	2	3.52796	0.87683	0.898	2.30599	1.53463	0.851104	3.88459	0	0
TCONS_00169271	XLOC_014517	-	anti-sense_lncRNA	439	chr2	28970695	28974649	-	3	0	0.30174	0.90428	0.539283	1.31332	0.594183	1.02184	0.348314	0.49312
TCONS_00157344	XLOC_013538	-	lincRNA	982	chr2	34938429	34947635	+	2	0.1303	0.63546	0	0.327867	0.315851	3.33461	0.753414	0.207167	0.148297
ENST00000414796.1	ENSG00000237320.1	AC019064.1	lincRNA	494	chr2	35223345	35225227	-	3	0.34501	0	0	1.53224	1.06298	1.68731	0.41141	0.562215	0
TCONS_00162172	XLOC_013545	-	lincRNA	4119	chr2	37827064	37835128	+	2	2.29306	2.21563	4.27861	1.22432	1.67613	1.47072	1.75203	4.27856	3.11887
TCONS_00158160	XLOC_014563	-	lincRNA	475	chr2	38696555	38742632	-	4	1.77182	1.16771	0.52014	0.702346	0.302449	0.709537	0.552807	7.09E-05	1.04999
TCONS_00158161	XLOC_014563	-	lincRNA	925	chr2	38722740	38742632	-	2	0.16776	1.13003	0.48511	0	0.142406	0.218612	0.528343	0.670973	0.0868494
TCONS_00157354	XLOC_013555	-	lincRNA	426	chr2	40767542	40772888	+	2	0.44778	0.31949	0.63885	0.570537	0	0.314361	0.812799	0	1.56648
TCONS_00164826	XLOC_014578	-	lincRNA	674	chr2	42370183	42375162	-	2	0.21459	0.3009	0.89531	1.21896	0.523571	0.447028	0.753447	0.34448	0.491598
TCONS_00160744	XLOC_013560	-	lincRNA	2386	chr2	43036908	43056061	+	2	0.51364	0.51727	0.82901	0.439151	0.534196	0.450399	0.373573	0.698637	0.501681
TCONS_00153084	XLOC_013569	-	intronic_lncRNA	2424	chr2	44625863	44628420	+	2	3.12105	1.71588	2.50703	1.928	3.20554	3.85777	1.80955	3.07185	2.85472
TCONS_00175357	XLOC_013569	-	intronic_lncRNA	1485	chr2	44718310	44722973	+	2	2.46082	2.47922	1.57746	1.1462	2.5875	3.12215	1.32155	2.82333	2.4299
ENST00000437916.2	ENSG00000259439.1	RP11-89K21.1	lincRNA	2880	chr2	45148216	45166338	-	2	3.07309	2.504	2.09247	1.83848	4.53857	2.54326	2.57694	4.48579	1.99215
TCONS_00163261	XLOC_014588	-	lincRNA	1156	chr2	45164494	45166375	-	2	1.52814	0.74163	2.01426	0.737206	3.09625	1.33062	2.5492	2.14054	1.72387
TCONS_00172826	XLOC_013577	-	intronic_lncRNA	1206	chr2	46072990	46128465	+	3	4.25669	2.39552	3.68952	1.7194	3.18661	2.73019	2.39285	2.65002	3.68301
TCONS_00172827	XLOC_013577	-	intronic_lncRNA	1574	chr2	46086304	46089319	+	2	5.56856	2.26648	3.61029	1.81749	2.5984	2.97075	2.17223	2.22866	2.73466
TCONS_00161502	XLOC_014597	-	lincRNA	356	chr2	46482455	46483944	-	2	0.64528	0.46701	2.35059	0.828607	0.406891	0.914341	1.60521	0	0
TCONS_00160757	XLOC_013580	-	lincRNA	210	chr2	46484923	46485349	+	2	0	0	6.64375	0	0	2.99218	0	0	0
TCONS_00155291	XLOC_013582	-	lincRNA	1719	chr2	46636474	46639918	+	2	4.09737	0.46576	2.52818	0.295037	0.445582	0.185289	0.192472	0.212064	0.152164
TCONS_00160762	XLOC_013589	-	lincRNA	378	chr2	47533502	47552982	+	2	2.26733	2.03989	2.86706	0	1.77664	0.800311	1.04629	0.947198	1.33396
TCONS_00164834	XLOC_014606	-	lincRNA	227	chr2	48223738	48230473	-	2	0	0	0	1.81013	0	0	0	0	0
TCONS_00173289	XLOC_014607	-	lincRNA	450	chr2	48540539	48541337	-	2	0.81039	0.28819	0.2877	4.12311	3.26107	0.851676	1.70574	0.332426	1.17738
TCONS_00161514	XLOC_014619	-	lincRNA	266	chr2	53832024	53833293	-	2	0	0	2.23519	0.946493	0.950572	1.04946	0.971409	0	0
TCONS_00157394	XLOC_013613	-	lincRNA	562	chr2	56812845	56816771	+	3	0	0.98854	0.98265	0.177563	0.172033	0.39089	1.32606	0.453864	0.323027
TCONS_00167344	XLOC_013614	-	lincRNA	519	chr2	57982897	57992339	+	2	0.95448	0.67444	0	0.201638	0	1.11002	0	0.775287	0.183665
TCONS_00163312	XLOC_014638	-	anti-sense_lncRNA	1730	chr2	58267079	58274263	-	2	1.20038	1.75712	1.96229	1.04612	0.924949	1.1957	0.879004	2.05267	1.81279
TCONS_00167906	XLOC_014651	-	lincRNA	243	chr2	62508896	62520656	-	2	0	0	0	0	1.35811	1.48812	5.63332	0	0
TCONS_00164234	XLOC_013631	-	anti-sense_lncRNA	1064	chr2	62668767	62728453	+	2	0.47186	0.24633	0.48703	1.2609	0.571353	0.896997	0.408539	1.77926	1.34132
TCONS_00159019	XLOC_013634	-	lincRNA	908	chr2	62677350	62691002	+	2	1.15104	0.40137	0.99282	0.362227	0.610977	0.697053	0.416762	1.2601	0.573719
TCONS_00177460	XLOC_013641	-	lincRNA	431	chr2	64500854	64550978	+	2	0.43819	2.4994	0.93676	0.837146	0.271976	0.922461	0.794298	0	2.55298
TCONS_00160811	XLOC_013647	-	lincRNA	2061	chr2	65257871	65260064	+	2	0.65808	1.06377	1.61162	4.60889	1.28858	4.42231	1.66283	6.22427	6.17481
TCONS_00161565	XLOC_014668	-	lincRNA	4047	chr2	65271564	65283312	-	2	1.24761	1.1928	1.15754	1.1142	1.00538	0.895304	0.862207	1.14706	1.03413
TCONS_00164868	XLOC_014673	-	lincRNA	357	chr2	65807329	65807995	-	2	0.64125	0.46396	0	0.823307	0	0	0	2.16054	0
TCONS_00156445	XLOC_014686	-	lincRNA	886	chr2	68813632	68817466	-	2	0.89088	0.41434	1.64011	0.0934688	0.36042	0.102785	1.11895	0.473115	0.253829
TCONS_00171289	XLOC_013667	-	lincRNA	1863	chr2	69871322	69881276	+	2	2.51581	1.19084	1.55273	1.53978	1.96036	2.49599	2.21362	2.46837	1.07681
TCONS_00164878	XLOC_014691	-	lincRNA	1255	chr2	70321030	70322401	-	2	0.67657	0.53734	0.59718	0.242874	0.525774	0.133491	0.556203	0.995029	0.987481
TCONS_00161595	XLOC_014714	-	lincRNA	3914	chr2	73530220	73534973	-	2	0.90733	0.74135	0.91806	0.155031	2.0498	0.511009	1.80204	0.799363	1.00912
TCONS_00177473	XLOC_013688	-	lincRNA	1368	chr2	73954375	73956205	+	2	2.79419	0.36383	2.21575	0.0548347	0.2637	0.602737	0.451648	0.552609	1.83251
ENST00000439192.1	ENSG00000237883.1	DGUOK-AS1	anti-sense_lncRNA	563	chr2	74185097	74208560	-	2	3.35787	0.59147	2.74365	2.12485	2.23018	2.72864	1.48757	0.678871	0.644247
TCONS_00156481	XLOC_014717	-	anti-sense_lncRNA	826	chr2	74185273	74209921	-	2	1.95195	0.4544	0.7873	0.102455	0.790561	0.901418	1.41728	0.908505	0.742333
ENST00000604271.2	ENSG00000270571.2	RP11-355F16.1	intronic_lncRNA	3438	chr2	75381492	75417075	+	3	10.9739	12.0817	5.56787	19.1271	2.93958	4.75465	1.83417	2.28063	3.52658
TCONS_00165548	XLOC_013709	-	lincRNA	2638	chr2	75502690	75506805	+	2	1.88427	2.20249	0.91447	7.74323	1.13412	1.55749	1.17168	1.38411	1.75149
TCONS_00160878	XLOC_013741	-	lincRNA	3088	chr2	87883252	87907659	+	2	0.34843	0.83618	1.18491	1.04577	0.910902	0.742721	0.335921	0.512934	0.797777
TCONS_00162367	XLOC_013741	-	lincRNA	1849	chr2	87883259	88003920	+	4	0.68962	1.53592	1.51755	5.31075	2.24453	2.3675	1.07538	1.44249	1.40333
TCONS_00165572	XLOC_013741	-	lincRNA	1161	chr2	88004008	88014481	+	5	0.84868	1.03284	1.02065	3.66681	1.02668	1.46584	1.10034	0.336403	0.843602
TCONS_00160879	XLOC_013745	-	lincRNA	818	chr2	88316288	88320932	+	2	1.15333	1.03576	1.70923	1.03785	2.40267	2.05456	1.14879	3.02432	0.940037
TCONS_00158314	XLOC_014776	-	lincRNA	1802	chr2	88986743	88990990	-	2	1.2102	1.10387	1.43789	0.439581	0.844829	1.09796	1.09455	0.703544	0.613074
TCONS_00165587	XLOC_013757	-	lincRNA	437	chr2	89326527	89384968	+	2	0	0	0	4.62287	0	4.19474	0	0	0
TCONS_00164313	XLOC_013760	-	lincRNA	2009	chr2	89620047	89622327	+	2	0.11283	0.03909	0.07712	0.459997	0.10198	0.0388856	0.064564	0.222365	0.319239
TCONS_00156537	XLOC_014786	-	lincRNA	2200	chr2	92317053	92325368	-	3	18.0656	10.0377	3.312	1.28008	1.78279	1.19568	4.55214	10.6948	4.33

TCONS_00160889	XLOC_013777	-	lincRNA	1696	chr2	95873229	95875737	+	2	1.03551	1.6212	1.59495	0.671605	1.46432	0.739448	1.06865	1.05994	0.827442
ENST00000425953.1	ENSG00000233757.2	AC092835.2	lincRNA	1096	chr2	95873283	95880597	+	5	4.64343	3.46095	4.2902	4.1699	4.02096	3.32416	2.14347	3.63877	3.01162
ENST00000421534.1	ENSG00000230747.1	AC021188.4	lincRNA	740	chr2	96973001	96987469	-	2	0.75386	1.45064	2.74428	1.30711	1.03253	1.82993	1.09876	1.50819	2.69307
TCONS_00173379	XLOC_014808	-	intronic_lncRNA	1474	chr2	97395393	97397177	-	2	3.04251	2.16732	2.46908	0.45239	1.40165	1.16026	1.74681	1.20246	1.13471
ENST00000608565.1	ENSG00000230606.6	AC159540.1	lincRNA	855	chr2	98087740	98088981	-	3	1.08839	0.43412	1.28909	0.881148	0.755254	1.83037	0.360894	0.991659	1.24108
ENST00000609004.1	ENSG00000228486.5	AC017099.3	lincRNA	1031	chr2	98285643	98288169	+	2	0.98111	1.36627	1.18202	1.07972	0.594198	0.932729	0.779169	0.779252	1.67393
TCONS_00154516	XLOC_014821	-	lincRNA	559	chr2	98670692	98701316	-	2	2.5463	1.59508	1.38749	0.179054	0	0.59127	0.501572	0.457756	0.488652
TCONS_00163498	XLOC_014824	-	lincRNA	501	chr2	99385370	99388442	-	2	1.14975	2.01226	2.67061	0.443632	1.76667	1.2277	3.29588	1.51373	2.06646
TCONS_00173395	XLOC_014824	-	lincRNA	921	chr2	99385370	99389619	-	4	0.50707	0.351	0.17126	0.348985	0.298935	0.174479	1.43849	0.391684	0.434114
TCONS_00160079	XLOC_014837	-	intronic_lncRNA	965	chr2	101876793	101878316	-	2	2.13103	2.4131	0.73439	1.59206	0.645844	1.01347	1.38676	2.01236	1.66779
TCONS_00160954	XLOC_013845	-	lincRNA	662	chr2	107318017	107394600	+	3	1.32082	0.46322	3.36973	0	0.403005	1.98772	0.515694	1.59125	0.12613
TCONS_00163542	XLOC_014852	-	anti-sense_lncRNA	877	chr2	109115549	109116613	-	2	2.40716	0.73481	1.45443	0.473574	0.913129	1.04156	1.30858	0.719238	1.02892
ENST00000440975.1	ENSG00000214184.3	AC012487.2	lincRNA	574	chr2	109128145	109150638	-	2	0.81492	1.3389	0.57022	0.171837	0	0.378261	0.641056	0.219466	0
TCONS_00153422	XLOC_013878	-	lincRNA	542	chr2	114082824	114086490	+	2	5.04725	0.41891	1.24993	0	0.364524	0.413922	0.175801	1.20285	0.513339
TCONS_00157608	XLOC_013888	-	lincRNA	2048	chr2	118596879	118599846	+	2	1.10451	1.53039	0.60391	0.45028	0.765296	1.10385	0.600329	1.1753	1.12493
TCONS_00167493	XLOC_013899	-	lincRNA	1894	chr2	120460703	120509900	+	4	2.53007	1.33597	1.44178	0.340103	1.37973	0.664472	1.41406	1.23523	1.32983
TCONS_00162494	XLOC_013915	-	lincRNA	854	chr2	126468474	126502713	+	2	0.46716	0.21739	0.64555	0.0980554	0.661866	0.107834	0.722912	1.73809	1.59813
TCONS_00153471	XLOC_013915	-	lincRNA	2214	chr2	126543818	126551502	+	2	0.96289	0.0351	0.6924	0.095338	0.763087	0.558759	1.65165	2.55521	2.00658
TCONS_00153474	XLOC_013919	-	lincRNA	385	chr2	128158192	128163635	+	2	1.09139	1.1765	0.78684	0.698014	0	0	2.00863	0.45487	0.641092
TCONS_00170807	XLOC_014906	-	lincRNA	3671	chr2	128226623	128230409	-	2	1.32384	0.97641	0.92234	0.387078	0.937563	1.3569	0.838537	0.994201	1.27924
TCONS_00164431	XLOC_013939	-	lincRNA	2411	chr2	131669782	131673798	+	2	1.9852	0.22374	1.48158	1.21571	1.83459	1.36771	0.369325	0.836115	0.443784
TCONS_00161047	XLOC_013963	-	lincRNA	647	chr2	140681627	140727323	+	2	0.45498	1.11756	2.5346	0	0.694503	0	0.533478	0.182865	0.130416
TCONS_00158492	XLOC_014953	-	intronic_lncRNA	4548	chr2	145077118	145082359	-	2	3.24131	1.99675	2.9117	2.33934	3.10577	2.87736	1.8195	2.23214	2.87704
TCONS_00153526	XLOC_013969	-	lincRNA	3500	chr2	145946300	145950988	+	2	2.97044	1.69041	2.06708	1.41532	0.967621	1.46987	1.19972	1.04587	0.751461
TCONS_00153536	XLOC_013980	-	intronic_lncRNA	1371	chr2	150225831	150235195	+	2	2.52576	0.96772	0.65704	0.328164	1.63073	0.721427	0.700745	1.17126	0.543397
TCONS_00153537	XLOC_013980	-	lincRNA	898	chr2	150338675	150342835	+	2	1.45935	0.40716	0.10072	0	0.354173	0.606066	0.33827	0.581089	0.24943
TCONS_00164455	XLOC_013981	-	lincRNA	378	chr2	150600233	150685364	+	3	0.56683	0.81596	0.81916	2.5398	0.355327	1.20047	1.04629	0	0.333491
TCONS_00155672	XLOC_013982	-	lincRNA	1352	chr2	151283054	151284611	+	2	5.52198	3.97074	8.71073	0.474977	1.95469	0.305564	1.75392	3.55823	2.98792
TCONS_00155673	XLOC_013982	-	lincRNA	9015	chr2	151283064	151294144	+	3	2.40601	2.12394	4.84458	0.395113	1.1165	0.127535	1.11718	2.31959	2.08154
TCONS_00159287	XLOC_013985	-	anti-sense_lncRNA	2389	chr2	152354074	152359955	+	2	2.65796	2.16308	2.86569	1.43259	2.24615	2.4738	2.79152	3.23134	2.68953
TCONS_00161074	XLOC_013985	-	anti-sense_lncRNA	2592	chr2	152357157	152362772	+	2	1.23764	1.06339	1.80599	1.23066	1.20736	1.11711	0.883338	1.47802	1.61638
TCONS_00177994	XLOC_014967	-	lincRNA	3651	chr2	152630516	152643606	-	2	4.76383	2.59841	3.67045	1.29774	3.18487	4.2369	1.82177	2.67436	2.60675
TCONS_00169711	XLOC_014968	-	intronic_lncRNA	643	chr2	152826505	152828265	-	2	0.45907	0.96667	0.47962	0.579833	2.52308	0.95699	0.807644	0.369103	0.263217
TCONS_00173509	XLOC_014973	-	lincRNA	469	chr2	156666443	156769661	-	5	0.00029	1.07045	0	0.2393	0.0003624	0	3.08117	2.8843	0.0430999
TCONS_00158513	XLOC_014973	-	lincRNA	525	chr2	156666453	156769650	-	5	1.0071	3.09385	1.85456	6.02E-05	0.909545	0	0	1.08361	0
TCONS_00158514	XLOC_014973	-	lincRNA	879	chr2	156696869	156769624	-	4	1.09765	0.75782	1.71563	0	0.118932	0	0.466451	1.05319	0.285304
TCONS_00161790	XLOC_014973	-	lincRNA	581	chr2	156762579	156769661	-	3	1.98869	0.70044	0.37076	0	0.972434	0	0.312079	0.334442	0.531393
TCONS_00154706	XLOC_014975	-	lincRNA	16334	chr2	156865822	156882927	-	2	9.45996	3.09929	5.93686	0.682786	3.5324	0.266666	3.28176	3.05152	1.41564
TCONS_00156737	XLOC_014975	-	lincRNA	3559	chr2	156875897	156881879	-	3	1.68848	0.81955	1.74357	0.129198	0.485978	0	0.357555	0.254978	0.118549
TCONS_00173513	XLOC_014975	-	lincRNA	2966	chr2	156875933	156880352	-	3	0	0	0	0	0	0	0.621599	0	0
ENST00000428651.1	ENSG00000226383.1	AC093375.1	lincRNA	1603	chr2	156877047	156881088	-	3	2.57976	1.61089	0.78784	0.45454	1.27418	0.0381089	1.28412	1.86973	0.804267
ENST00000442666.1	ENSG00000204380.2	AC005042.4	anti-sense_lncRNA	825	chr2	159535168	159591330	-	2	1.14045	0.56892	0.45062	2.25762	1.97959	0.67715	2.83918	1.94996	1.48706
TCONS_00156795	XLOC_014990	-	lincRNA	884	chr2	161411205	161504883	-	2	1.63804	0.41556	0.71968	1.59363	3.0726	3.19568	0.517977	0.474517	0.169718
ENST00000436506.1	ENSG00000227403.1	AC009299.3	lincRNA	765	chr2	162101266	162103965	+	3	2.70237	3.14961	5.11706	1.81685	4.49349	3.99676	3.7767	4.32101	4.01345
TCONS_00156810	XLOC_015004	-	lincRNA	2828	chr2	166688806	166695401	-	2	8.27457	3.27943	2.70343	1.3879	1.58961	1.79259	2.26187	1.8644	1.9539
TCONS_00173563	XLOC_015027	-	lincRNA	324	chr2	173139821	173144533	-	2	0	0.59009	0.5973	0	0	0	0	1.38249	0.96568
ENST00000450443.1	ENSG00000225205.1	AC093818.1	anti-sense_lncRNA	1397	chr2	173368167	173421324	-	3	0.68165	0.65079	1.63586	4.92238	1.33786	5.35173	1.56656	6.67156	7.00666
TCONS_00164508	XLOC_014058	-	lincRNA	233	chr2	173585551	173588988	+	2	0	0	0	3.21124	0	1.78608	0	0	0
ENST00000417038.1	ENSG00000226853.2	AC010894.3	lincRNA	393	chr2	175352131	175353028	+	2	1.57014	0	1.88213	0	0.327	0.737471	0.64016	0.870366	0.30692
TCONS_00165101	XLOC_015047	-	lincRNA	348	chr2	177626222	177628949	-	2	0	0	0.99342	1.31033	0	1.92831	0	0.574588	0
TCONS_00158588	XLOC_015049	-	lincRNA	390	chr2	177976434	178016097	-	2	0.53153	1.90798	1.14788	0.679515	0.332302	0.374621	2.6035	0.442365	0
ENST00000419129.1	ENSG00000237477.1	AC093911.1	lincRNA	552	chr2	180138558	180150434	+	2	7.21285	1.42388	1.41578	0	0.177	0	1.02379	0	0.332349
TCONS_00173056	XLOC_014088	-	lincRNA	261	chr2	180275910	180278620	+	2	1.53566	0	0	0	1.01976	2.24826	1.04524	4.17511	0
TCONS_00164524	XLOC_014091	-	lincRNA	1107	chr2	181988362	182091958	+	2	0.22477	1.0947	1.62301	1.27164	0.680119	0.621345	1.426	2.58563	1.91601
TCONS_00165120	XLOC_015067	-	lincRNA	538	chr2	183536587	183539207	-	3	1.50194	0.42395	1.26511	0.760963	2.39792	0.628287	1.2457	0	0.346342
TCONS_00169882	XLOC_015071	-	anti-sense_lncRNA	1163	chr2	186585307	186623219	-	3	3.10487	1.06035	1.15195	0.718642	2.20645	1.32347	1.6336	2.30287	1.32289
TCONS_00160359	XLOC_015071	-	anti-sense_lncRNA	3225	chr2	186620919	186650285	-	2	3.83675	1.50811	2.94982	1.06178	2.58948	2.29863	2.46866	2.88123	1.56353
ENST00000419719.1	ENSG00000237877.2	AC097500.2	lincRNA	587	chr2	186898258	186947950	-	5	10.5026	4.24939	4.58853	5.81127	6.10871	7.30937	4.48655	8.47653	5.58437
TCONS_00177641	XLOC_014105	-	lincRNA	1390	chr2	187868031	188057889	+	2	1.1999	0.83317	1.11663	1.77604	0.983444	0.709884	1.13265	1.89809	1.94429
TCONS_00168179	XLOC_015078	-	lincRNA	749	chr2	190210598	190305848	-	4	3.15159	0.77816	2.18455	0.701242	1.46672	1.54267	2.37676	2.52117	1.05939
TCONS_00173597	XLOC_015078	-	lincRNA	492	chr2	190258471	190259694	-	2	5.90509	3.68976	5.39176	1.5428	2.99711	1.69897	4.97216	3.96332	2.21068

TCONS_00154898	XLOC_015095	-	lincRNA	1032	chr2	192073829	192108934	-	2	1.95986	1.10875	1.43357	1.00139	0.445109	1.0163	0.636725	1.36203	0.417976
ENST00000594798.1	ENSG00000233766.3	AC098617.1	intronic_lncRNA	629	chr2	192899510	192908909	+	2	1.18493	0.66575	0.82599	1.79651	1.73768	1.15315	1.11301	0.953539	0.815759
ENST00000449346.1	ENSG00000231621.1	AC013264.2	intronic_lncRNA	455	chr2	198062715	198063997	+	2	14.3035	18.9222	15.2209	4.29431	5.16234	5.28717	13.843	8.1418	12.6918
TCONS_00164563	XLOC_014135	-	lincRNA	464	chr2	198562583	198563590	+	2	0	0.2726	0.27194	0.975747	1.42361	0	0.920652	1.25672	0.668193
TCONS_00157071	XLOC_015146	-	lincRNA	580	chr2	206959579	206960748	-	2	1.0695	0.37642	1.30901	0.676441	0.491285	0.930618	0.315302	0.647743	0.615001
TCONS_00155943	XLOC_014178	-	lincRNA	1042	chr2	207728120	207732313	+	2	3.99432	2.19099	2.66609	0.989466	1.17276	1.33892	2.44632	0.865077	0.894789
TCONS_00154986	XLOC_015165	-	lincRNA	2452	chr2	213755834	213768856	-	2	3.58938	2.7769	2.99591	0.953609	3.79501	2.59501	2.30488	2.58868	2.49691
TCONS_00157092	XLOC_015165	-	lincRNA	1457	chr2	213760746	213768539	-	2	2.98722	2.78849	2.01279	0.735843	2.7402	2.34626	2.0968	1.82594	2.64865
TCONS_00157093	XLOC_015165	-	lincRNA	4246	chr2	213796686	213801506	-	2	3.76076	1.81449	3.67975	1.15424	2.97378	2.77956	1.72563	2.31982	2.46512
TCONS_00175157	XLOC_015165	-	lincRNA	1881	chr2	213829611	213838409	-	2	5.94909	2.60853	5.81194	1.37094	5.45199	3.22254	3.40627	4.30905	3.67682
ENST00000415479.1	ENSG00000230838.1	AC093850.2	lincRNA	648	chr2	216582766	216584147	+	2	0.68095	2.54866	1.10636	1.72	1.94021	0.630831	1.59676	0.729793	0.780728
TCONS_00153839	XLOC_014219	-	lincRNA	843	chr2	217695236	217696205	+	2	3.00915	0.99514	0.7662	0.199476	0.384737	0.877489	0.643576	0.378914	0.451585
TCONS_00161255	XLOC_014221	-	lincRNA	705	chr2	218142332	218147478	+	2	1.20883	0.70558	1.11918	0.762569	0.7366	1.11852	0.823743	0.161468	0.230543
TCONS_00165206	XLOC_015199	-	lincRNA	213	chr2	220064032	220064650	-	2	0	0	3.06933	2.48787	0	0	0	0	0
ENST00000442416.1	ENSG00000227308.2	AC009502.4	lincRNA	361	chr2	220594832	220602464	+	2	0	2.26082	0	1.20414	1.18176	1.7714	2.32829	2.10435	0.369685
TCONS_00170517	XLOC_014265	-	lincRNA	351	chr2	220886475	220934017	+	3	2.66476	3.37981	2.9184	1.28422	0.420728	4.72432	4.15468	1.12528	2.76373
TCONS_00153899	XLOC_014266	-	lincRNA	1003	chr2	221494981	221499203	+	3	1.90355	1.59101	1.22369	0.0798181	0.691947	1.05312	0.953434	0.705899	0.433176
TCONS_00157941	XLOC_014266	-	lincRNA	456	chr2	221605020	221605559	+	2	2.37469	2.25036	1.12292	0.503226	0.734533	0.277175	2.85236	1.62177	0
TCONS_00156025	XLOC_014274	-	lincRNA	2898	chr2	224045812	224273133	+	2	3.47537	2.43447	1.67508	3.77094	2.06092	2.8758	1.39462	1.11019	2.8374
TCONS_00166142	XLOC_014274	-	lincRNA	7261	chr2	224046887	224073791	+	2	3.71246	4.66722	2.00342	6.78428	2.59105	7.04114	1.20327	2.5572	3.56763
TCONS_00171830	XLOC_014274	-	lincRNA	1900	chr2	224047053	224278638	+	3	1.80769	3.92814	1.32741	3.46281	1.82909	5.00766	1.06051	2.39158	3.26625
TCONS_00159579	XLOC_014274	-	lincRNA	4485	chr2	224047053	224187757	+	2	1.42113	2.09816	0.81364	3.16684	1.50466	4.58608	1.20608	1.16664	1.94763
TCONS_00167708	XLOC_014274	-	lincRNA	1201	chr2	224244354	224325893	+	4	0.36278	0.2233	0.72651	1.06006	0.96026	1.06516	0.500761	0.407134	0.879527
TCONS_00153907	XLOC_014274	-	lincRNA	4054	chr2	224319922	224325391	+	2	1.08843	1.35103	0.54809	1.24901	0.593158	2.63744	0.478085	0.855645	1.62757
TCONS_00171831	XLOC_014274	-	lincRNA	4555	chr2	224319924	224327550	+	3	0.80576	1.41152	0.48329	0.69323	0.5521	1.73091	0.277818	0.531419	0.979519
TCONS_00159580	XLOC_014278	-	anti-sense_lncRNA	5358	chr2	225837829	226167342	+	2	1.38606	1.81967	0.60668	0.607676	0.356933	0.354253	0.450911	1.22807	1.03918
TCONS_00162024	XLOC_015225	-	intronic_lncRNA	4380	chr2	225874364	225878807	-	2	2.32097	2.22909	3.69468	2.49463	2.01188	2.11869	1.61448	2.80181	4.88231
TCONS_00173159	XLOC_014288	-	lincRNA	1849	chr2	228794434	228798848	+	2	4.20794	1.58701	1.6083	0.310573	0.410333	0.725298	1.84274	1.07388	2.06688
TCONS_00155060	XLOC_015232	-	lincRNA	1058	chr2	229353366	229706954	-	3	4.51397	1.40568	2.53411	0.522832	0.503453	0.57482	2.2629	0.660153	0.405228
TCONS_00164657	XLOC_014290	-	lincRNA	3681	chr2	230989387	231007585	+	2	0.58668	0.6085	0.35988	1.36007	1.14653	0.767374	0.41806	0.53025	0.596365
ENST00000418025.1	ENSG00000228162.1	AC097713.3	lincRNA	562	chr2	235346972	235370771	+	3	0.84177	1.18625	1.17918	2.13075	9.2898	1.95445	1.49182	3.85784	0
TCONS_00157196	XLOC_015256	-	lincRNA	767	chr2	235742519	235743378	-	2	1.97477	0.12554	0.6218	0	0.764448	0.622281	0.10453	0.143517	0.102542
TCONS_00168321	XLOC_015269	-	lincRNA	576	chr2	240851548	240852489	-	2	0.54041	0.19024	0.37808	0.170918	1.32424	0.940587	0	0	0.310821
TCONS_00155106	XLOC_015272	-	intronic_lncRNA	864	chr2	241388823	241390375	-	2	1.99398	0.85636	0.74164	0.86917	3.25906	1.80546	0.889779	0.73352	1.22411
ENST00000433036.1	ENSG00000228989.1	AC133528.2	lincRNA	415	chr2	242629829	242633704	+	2	4.7044	11.0954	5.71909	4.19965	4.09816	2.64495	4.28341	5.05487	3.29719
TCONS_00170581	XLOC_014370	-	lincRNA	3002	chr2	242823535	242910908	+	3	1.00015	0.81429	0.74969	1.14515	1.2382	1.60416	0.768959	1.31512	1.78849
TCONS_00159685	XLOC_014370	-	lincRNA	1365	chr2	242824145	242909230	+	3	1.27388	0.71113	1.9114	0.651668	0.344648	0.60848	0.0008521	1.11588	0.80092
TCONS_00159686	XLOC_014371	-	lincRNA	836	chr2	242913041	242914067	+	2	0.80057	2.68317	1.43882	0.605033	0.972524	0.66539	0.836737	1.6603	1.3698
TCONS_00180143	XLOC_015676	-	lincRNA	309	chr20	998222	999651	-	2	1.82579	0	0.68243	0.591809	0	0	0	0	0
TCONS_00180982	XLOC_015308	-	lincRNA	425	chr20	1166736	1171411	+	2	0	1.92568	0	0.859637	0	0	0	0	0
TCONS_00183673	XLOC_015681	-	lincRNA	245	chr20	1394922	1399592	-	3	0	1.48641	0	0	1.31175	0	1.3584	1.80063	0
TCONS_00182128	XLOC_015688	-	lincRNA	575	chr20	1753090	1753774	-	2	0.27092	0.95377	0.18955	2.39927	0.995871	1.32036	0.319645	0.218866	0.467493
TCONS_00182914	XLOC_015690	-	lincRNA	3636	chr20	1844760	1848859	-	2	1.04013	0.63703	0.16205	1.76891	1.51898	1.24798	0.254133	0.443785	0.285311
TCONS_00180149	XLOC_015689	-	anti-sense_lncRNA	6626	chr20	1865314	1874889	-	2	2.14583	2.55828	1.04933	1.70208	1.69954	1.83716	0.615007	1.27229	0.923619
TCONS_00180182	XLOC_015712	-	lincRNA	1504	chr20	4448454	4450957	-	2	2.58148	1.52006	1.01835	0.294645	0.330508	0.4318	0.179602	0.3091	0.221699
TCONS_00185504	XLOC_015345	-	lincRNA	833	chr20	6150557	6151721	+	2	0	0.22467	0.22242	0.101318	0.293152	0.111426	0.28026	1.15497	0.367026
TCONS_00182738	XLOC_015354	-	lincRNA	330	chr20	10194340	10195939	+	2	0	0.56248	0	0.99443	0	0	0	0	0.92033
TCONS_00181963	XLOC_015358	-	lincRNA	224	chr20	10845382	10889495	+	3	0	0	14.128	0	3.94905	0	2.07547	0	0
TCONS_00185512	XLOC_015360	-	lincRNA	418	chr20	10919560	10920894	+	2	0	0.33149	0.33161	0	0	0.652042	0.562836	1.53306	0
TCONS_00179852	XLOC_015401	-	lincRNA	2227	chr20	24911288	24914086	+	2	12.4877	12.0308	15.7881	4.70473	13.071	9.26441	14.5962	13.0508	10.1393
ENST00000376445.3	ENSG00000226465.1	RP13-401N8.1	lincRNA	320	chr20	25731854	25733397	+	2	0.83271	2.43989	0	0.538341	0	1.18958	0	0	0.998365
TCONS_00183732	XLOC_015783	-	anti-sense_lncRNA	1376	chr20	30921433	30937936	-	2	9.10619	8.97338	10.1702	7.95132	9.84733	9.21887	8.82136	10.359	12.1496
TCONS_00183144	XLOC_015454	-	lincRNA	851	chr20	32774306	32779486	+	2	0.46931	0.4368	0.86475	2.36421	1.42488	3.35832	1.36182	0.87311	0.445988
ENST00000424358.1	ENSG00000126005.11	MMP24-AS1	anti-sense_lncRNA	867	chr20	33864953	33865932	-	2	5.49704	4.04928	4.64051	2.11506	6.76664	5.18048	6.46563	5.4763	4.17792
TCONS_00182201	XLOC_015807	-	anti-sense_lncRNA	3090	chr20	34061632	34066042	-	2	1.20117	1.4419	2.0239	0.686349	1.44522	1.55695	0.947351	1.77819	1.71655
TCONS_00182207	XLOC_015815	-	lincRNA	564	chr20	35199819	35201750	-	2	0.27906	1.17963	1.75881	0.529746	0.855366	0.388727	0.329632	1.12826	0.481837
TCONS_00182806	XLOC_015500	-	anti-sense_lncRNA	299	chr20	36888683	36917340	+	2	0	0.73914	0	3.89958	0.645701	1.43773	5.19134	4.35883	2.42209
TCONS_00180334	XLOC_015830	-	lincRNA	253	chr20	38414368	38416871	-	3	1.719	0	0	2.27843	0	0	0	1.5742	0
TCONS_00185138	XLOC_015833	-	lincRNA	425	chr20	39092885	39095574	-	2	0	0	1.6045	0	0.558809	0	1.361	0.370864	0.262267
TCONS_00181390	XLOC_015836	-	lincRNA	744	chr20	39225864	39234128	-	2	0.74834	0.91628	0.77822	0	1.02485	0.259487	0.32715	0.598763	0.641535
TCONS_00186215	XLOC_015837	-	lincRNA	272	chr20	39624338	39634101	-	2	1.33422	3.00397	3.09062	0	0.877736	0.970738	0	0	1.64495

TCONS_00183001	XLOC_015880	-	lincRNA	253	chr20	45114260	45117436	-	2	3.438	1.30662	0	2.27843	0	0	2.36981	3.1484	1.0769
TCONS_00180024	XLOC_015560	-	lincRNA	235	chr20	46648350	46649099	+	2	2.30501	0	0	1.54567	0	1.71905	0	0	0
TCONS_00181190	XLOC_015571	-	lincRNA	374	chr20	48789129	48789636	+	2	2.89828	0.41759	1.25826	0.371225	0.36372	0.409451	2.14372	0	0
TCONS_00182274	XLOC_015912	-	lincRNA	1924	chr20	51364247	51406264	-	4	0.71031	0.2051	1.45703	0.111393	0.392424	0.0816114	0.271079	0.140033	0.134012
TCONS_00183023	XLOC_015913	-	lincRNA	2053	chr20	51457000	51460553	-	2	0.88123	0.80128	0.63992	1.20902	0.862776	0.493493	0.756251	1.21553	0.124654
TCONS_00183024	XLOC_015913	-	lincRNA	905	chr20	51461061	51512001	-	2	1.58939	0.50386	0.79768	2.09169	0.876569	0.600026	0.75342	1.15048	0.576179
TCONS_00182087	XLOC_015586	-	lincRNA	733	chr20	52355374	52357651	+	2	0.95465	0.80176	0.5297	0.481578	0.464992	0.264868	1.00217	0.764175	0.327457
TCONS_00182863	XLOC_015598	-	lincRNA	1289	chr20	55549320	55552932	+	3	0.74912	0.45542	0.38559	0.764654	0.113172	0.38794	0.37705	1.63087	0.37197
TCONS_00182282	XLOC_015921	-	lincRNA	279	chr20	56064055	56070322	-	2	0	0	3.76963	4.02391	0.804441	0	1.63236	2.18345	0
TCONS_00184559	XLOC_015928	-	lincRNA	1197	chr20	56821088	56884504	-	10	2.55657	3.19932	3.30164	4.36951	2.34982	3.24937	2.5914	2.59313	3.13575
TCONS_00180457	XLOC_015929	-	intronic_lncRNA	2466	chr20	57457593	57461875	-	2	1.93701	1.12266	1.94409	0.593087	1.68147	0.508219	1.23536	1.59855	0.891442
TCONS_00180953	XLOC_015929	-	intronic_lncRNA	755	chr20	57457709	57463853	-	3	0	0	0.08665	0	0	0.137583	0	0	0
TCONS_00180458	XLOC_015929	-	intronic_lncRNA	1169	chr20	57459112	57463853	-	2	1.78857	0.5853	1.52586	0.528943	1.27269	0.766246	1.09114	1.4117	0.896355
TCONS_00180095	XLOC_015624	-	lincRNA	1504	chr20	58754901	58896571	+	4	3.5202	2.38866	1.39353	0.540183	0.283292	0.539749	0.583706	0.43274	0.620757
TCONS_00182697	XLOC_015938	-	anti-sense_lncRNA	254	chr20	60883424	60884086	-	2	8.47019	1.28672	5.34527	5.61018	4.52969	3.73658	2.33158	7.74619	1.06026
TCONS_00183046	XLOC_015944	-	intronic_lncRNA	5619	chr20	61277779	61283680	-	2	0.59754	0.26606	0.66418	0.903678	0.324018	0.283564	0.217709	0.510459	0.582936
TCONS_00181475	XLOC_015944	-	intronic_lncRNA	3660	chr20	61279599	61283559	-	2	0.3924	1.48089	1.33236	1.432	1.2491	1.12015	1.30737	2.19186	2.61976
TCONS_00182885	XLOC_015651	-	lincRNA	391	chr20	62259474	62261030	+	2	0.52879	0	0.38054	1.35186	0.661032	0.745277	0.323632	0	0
TCONS_00183666	XLOC_015661	-	intronic_lncRNA	241	chr20	62690023	62690988	+	2	2.07616	0	1.66788	1.38626	2.8145	0	0	0	1.31663
TCONS_00188453	XLOC_015974	-	lincRNA	378	chr21	9711969	9746428	+	2	1.13366	0.81596	0	0	1.06598	0.400155	0.348763	0	0
TCONS_00189590	XLOC_016136	-	lincRNA	776	chr21	15280021	15282494	-	3	0.70692	0.74133	0.12239	0.222755	0.967352	0.490011	0.308587	1.97733	0.706473
ENST00000428809.1	ENSG00000224905.2	AP001347.6	anti-sense_lncRNA	1332	chr21	15399742	15516789	+	3	1.62162	0.62576	0.92707	0.39607	1.9049	1.43048	1.55389	2.21002	0.919981
TCONS_00189438	XLOC_016143	-	lincRNA	1658	chr21	16133206	16135588	-	2	2.02931	3.36977	6.22601	3.03919	2.67036	3.84848	4.90324	7.91696	6.315
ENST00000435315.2	ENSG00000226751.2	AF127936.5	lincRNA	695	chr21	16134031	16135411	-	2	0	1.37752	0.00363	2.95549	1.21949	2.1771	0.333985	3.74117	1.13926
ENST00000413645.1	ENSG00000228798.1	AP000473.5	lincRNA	550	chr21	18003146	18012952	+	2	2.61137	2.04593	3.25503	0.183683	1.06817	1.01098	0.858206	0.939691	0.334279
TCONS_00188462	XLOC_015998	-	lincRNA	1224	chr21	25800779	25811350	+	2	0.697	0.48448	1.09407	0.3128	0.361186	0.275085	0.401269	1.02543	1.18713
TCONS_00187639	XLOC_016000	-	lincRNA	8047	chr21	26934153	26942309	+	2	1.02837	1.04284	5.30514	3.17065	0.938125	1.82672	1.28828	2.65509	3.25259
TCONS_00188840	XLOC_016003	-	lincRNA	323	chr21	28417830	28447247	+	2	4.06572	2.97471	7.22815	0	0	1.16068	0.516703	0.697091	0.973658
TCONS_00188348	XLOC_016166	-	lincRNA	908	chr21	29541986	29598351	-	4	0.50972	0.00014	0.09928	1.18176	0.503832	1.96371	0.454207	1.10166	1.10255
TCONS_00188916	XLOC_016166	-	lincRNA	3493	chr21	29581613	29598467	-	3	1.34752	0.10719	0.5073	1.30079	1.85037	1.62841	1.08825	1.30117	0.762653
TCONS_00187935	XLOC_016166	-	lincRNA	9118	chr21	29810755	29901568	-	3	5.20031	1.85878	2.36097	2.53161	2.99892	4.877	2.72265	4.49211	4.03096
TCONS_00187220	XLOC_016039	-	lincRNA	512	chr21	35665740	35697230	+	2	0.6505	0.68983	0.22892	0	0.200107	0.45405	2.32047	0.793206	0.563579
TCONS_00189165	XLOC_016194	-	intronic_lncRNA	1849	chr21	35855026	35859394	-	2	4.14606	1.07231	1.90457	1.16465	1.60404	1.91992	0.779623	0.927447	1.75166
TCONS_00187975	XLOC_016196	-	lincRNA	614	chr21	37315475	37354225	-	2	2.45529	7.76461	2.741	4.80991	2.40202	1.02445	0.865909	0.791104	0.704763
ENST00000457157.2	ENSG00000231106.2	AP000688.8	lincRNA	1048	chr21	37377636	37380136	+	2	14.1819	49.2808	19.3564	3.9298	2.54715	2.1603	8.88231	10.4021	4.78378
TCONS_00189898	XLOC_016198	-	lincRNA	584	chr21	37503222	37505273	-	2	0.79382	0.74488	1.11005	0.66936	0.972182	1.84172	0.467869	1.70888	2.43401
TCONS_00189252	XLOC_016063	-	lincRNA	801	chr21	40229451	40230347	+	2	1.35524	1.06532	0.23444	0.10673	0.10297	0	2.56089	0.405811	0.580125
TCONS_00188164	XLOC_016211	-	lincRNA	345	chr21	40395852	40400664	-	2	0	0	0.50745	1.78332	0	0	0.8676	1.17408	0
TCONS_00187356	XLOC_016229	-	lincRNA	607	chr21	43131841	43133218	-	2	1.99784	2.10666	2.26621	0.473519	0.458234	0.521091	0.734378	0.201257	0.573641
TCONS_00187673	XLOC_016076	-	lincRNA	900	chr21	43442115	43445077	+	2	6.54836	2.13144	6.12629	0.366378	0.353153	1.4101	1.51779	0.463525	0.580328
TCONS_00186871	XLOC_016235	-	anti-sense_lncRNA	491	chr21	43501668	43505139	-	2	2.78835	0.98738	1.22974	0	0.214829	0.243557	0.415832	0.5682	0.201675
TCONS_00188017	XLOC_016242	-	anti-sense_lncRNA	5674	chr21	43990408	44001592	-	4	1.09903	0.66965	0.75871	0.262629	0.564331	0.244952	1.58209	0.563489	0.937278
TCONS_00186883	XLOC_016242	-	anti-sense_lncRNA	8903	chr21	43991142	44000161	-	2	2.38381	1.48369	1.84661	0.371304	0.455737	0.394958	1.33517	0.548028	0.860379
ENST00000419628.1	ENSG00000225431.1	AP001626.1	lincRNA	1775	chr21	44019390	44035168	-	2	2.07312	1.70675	1.37403	0.569069	0.546868	0.670087	1.55884	0.511198	0.623621
TCONS_00187491	XLOC_016083	-	lincRNA	440	chr21	44299742	44304065	+	2	0	1.98799	0	0.537019	0	0.735737	1.38633	0.96338	1.51177
TCONS_00187682	XLOC_016083	-	lincRNA	533	chr21	44299754	44304136	+	2	0	0.18713	2.00537	0.289916	0.937113	0	0.282255	0.646372	0.441821
TCONS_00186702	XLOC_016083	-	lincRNA	643	chr21	44299754	44304136	+	2	2.43896	0.26298	1.16823	0.386226	0.433443	0.74687	1.34686	0.919734	0.612757
TCONS_00187031	XLOC_016085	-	lincRNA	3427	chr21	44337869	44341347	+	2	2.21491	1.20354	1.59626	0.634458	1.5985	1.76457	0.848047	0.870599	1.51911
TCONS_00187252	XLOC_016087	-	lincRNA	1402	chr21	44527844	44530491	+	2	0.76368	0.53024	1.86172	0.372969	0.922338	0.819911	0.682489	0.87239	0.625558
TCONS_00188584	XLOC_016248	-	lincRNA	392	chr21	44731449	44733926	-	2	0	0	0	0.336193	0.986246	0.370677	0.643692	1.7502	0.30856
ENST00000411694.1	ENSG00000225331.1	AP001055.6	lincRNA	863	chr21	45578623	45579955	-	2	0.30723	0.85767	0.74278	3.19178	1.11909	1.59547	1.15849	1.10196	1.75139
TCONS_00187700	XLOC_016118	-	lincRNA	338	chr21	47392853	47394107	+	2	0.72737	0	1.60305	0	1.84562	0	1.37178	0.618234	0
ENST00000444039.1	ENSG00000237476.1	XXbac-B135H6.1	lincRNA	684	chr22	21311380	21318967	+	2	0.21018	1.32578	1.16863	0.928415	1.92235	0.291828	1.59788	0.843057	1.08298
TCONS_00192180	XLOC_016641	-	lincRNA	1912	chr22	22442093	22452984	-	2	0.41725	0.57832	0.12228	1.72001	0.0359258	0.821844	0.204744	1.17517	3.6775
TCONS_00191467	XLOC_016642	-	lincRNA	718	chr22	22649093	22652390	-	3	1.76793	1.51291	0.68163	1.36283	1.31617	0.681442	0.573269	0.786708	1.12347
TCONS_00190558	XLOC_016650	-	lincRNA	481	chr22	23994588	23995352	-	2	1.80425	3.58116	1.27487	0.458168	0.445256	1.51378	1.07818	1.47273	1.67194
TCONS_00190225	XLOC_016374	-	lincRNA	219	chr22	24643924	24644876	+	2	3.17176	2.47621	0	0	2.21283	0	0	0	0
TCONS_00191054	XLOC_016685	-	intronic_lncRNA	566	chr22	29049511	29052415	-	2	2.22034	1.75964	1.16598	0	1.02074	0.966469	0.163885	1.57071	0.638885
TCONS_00191865	XLOC_016689	-	lincRNA	621	chr22	29582144	29601808	-	3	1.69027	2.71425	0.84202	0.915403	1.03317	0.503655	1.84403	1.55531	2.07867
TCONS_00190281	XLOC_016424	-	lincRNA	2183	chr22	30870870	30876341	+	2	3.3444	3.80685	1.95805	0.410325	1.14703	1.95079	3.11634	0.810735	1.63363
TCONS_00191286	XLOC_016424	-	lincRNA	2251	chr22	30870870	30876341	+	2	1.19547	1.66144	0.79256	0.289814	4.19E-05	0	0.458552	0.0003008	0.278381

TCONS_00191709	XLOC_016424	-	lincRNA	1742	chr22	30871270	30883227	+	4	3.0438	2.70686	3.30525	0.498225	0.877823	1.46017	2.23703	1.04441	0.936787
ENST00000442126.1	ENSG00000181123.4	RP4-539M6.14	anti-sense_lncRNA	502	chr22	30887789	30888791	+	2	2.35159	2.85235	2.13056	0.639174	0.413719	0.703824	0.800149	0.820289	0.776777
TCONS_00190643	XLOC_016725	-	anti-sense_lncRNA	563	chr22	32748643	32751037	-	2	2.51842	0.39431	0.7839	0.885355	1.37242	0.584711	0.495857	1.81032	0.483188
TCONS_00190673	XLOC_016757	-	lincRNA	3875	chr22	37584975	37594714	-	2	2.63972	1.26792	2.36689	1.06192	1.55369	1.81709	1.26694	2.4236	1.99262
TCONS_00190362	XLOC_016476	-	intronic_lncRNA	539	chr22	38483272	38484377	+	2	1.79705	1.47936	0.42043	0.758706	0.183902	0.208806	0.177413	0.242758	0.345304
ENST00000412067.1	ENSG00000228274.3	RP3-508I15.9	lincRNA	738	chr22	39071819	39077792	-	2	2.45912	1.45609	0.91822	0.477087	1.03641	0.524789	0.772058	1.05973	0.432509
TCONS_00193041	XLOC_016482	-	lincRNA	1733	chr22	39348725	39350567	+	2	0.4659	0.55379	0.09109	1.08583	0.361226	1.05566	0.305121	0.577818	1.65846
TCONS_00192255	XLOC_016794	-	lincRNA	391	chr22	41591340	41593482	-	2	2.64393	2.27729	1.52215	0.337966	0.330516	1.11792	1.61816	1.75978	0.310218
TCONS_00193994	XLOC_016848	-	lincRNA	363	chr22	45852959	45853568	-	2	1.85425	0	0	0	0	1.74946	0	1.03865	0
TCONS_00193890	XLOC_016563	-	lincRNA	3366	chr22	47620388	47624926	+	2	1.03203	0.28996	0.48373	0.242509	0.659501	0.466303	0.312673	0.659228	1.25696
TCONS_00192279	XLOC_016861	-	lincRNA	9027	chr22	47855847	47866361	-	2	1.7243	0.86306	1.68469	0.760795	2.20985	1.31341	0.42775	0.898616	0.789709
TCONS_00194858	XLOC_016564	-	lincRNA	306	chr22	47985213	47987715	+	2	0.93739	0	0	0	0.603117	0	0	1.62596	1.697
TCONS_00191411	XLOC_016584	-	lincRNA	1673	chr22	51023725	51025917	+	3	1.31603	0.76858	1.46974	0.608526	1.46221	0.812113	0.913249	0.76555	0.823901
TCONS_00190483	XLOC_016587	-	lincRNA	251	chr22	51111230	51112702	+	2	5.31266	1.34801	1.40252	1.17496	1.18737	3.914	3.67418	0	1.11154
TCONS_00207661	XLOC_017661	-	lincRNA	224	chr3	3164133	3168277	-	2	0	0	0	0	1.97453	0	0	2.7336	0
TCONS_00196498	XLOC_017666	-	lincRNA	2665	chr3	3817103	3824384	-	3	1.837	1.38079	0.74625	0.56122	0.697927	0.771433	0.761984	0.782226	1.07275
TCONS_00203815	XLOC_017666	-	lincRNA	564	chr3	3819290	3822983	-	3	3.8073	4.09433	3.24327	1.30349	1.71073	1.54656	2.76546	0	2.59766
TCONS_00201149	XLOC_017666	-	lincRNA	534	chr3	3968544	4003654	-	2	1.51981	2.78919	0.64034	1.34772	0.933511	1.05978	0.900835	0	1.40224
ENST00000439325.1	ENSG00000233912.1	AC026202.3	anti-sense_lncRNA	672	chr3	5198590	5229014	-	4	1.50846	1.81317	1.64849	4.35252	1.18309	2.39431	1.51345	6.40043	5.30734
TCONS_00211609	XLOC_016909	-	lincRNA	651	chr3	8723562	8725209	+	2	2.25481	5.22121	2.82563	0.569504	1.23888	0.156653	2.90739	0.906045	1.93868
TCONS_00207675	XLOC_017696	-	intronic_lncRNA	673	chr3	11291935	11296513	-	2	0	0.45232	0.95527	0.407182	1.4429	0	2.8036	0	2.80584
TCONS_00211982	XLOC_017696	-	intronic_lncRNA	753	chr3	11292017	11296547	-	3	0.55216	0	0.46073	0	0	0.255244	2.11492	0.883274	0.86428
TCONS_00196527	XLOC_017701	-	anti-sense_lncRNA	423	chr3	12196393	12196870	-	2	4.08369	0	0.64778	0	0	0.318635	0.549545	0.374325	0
TCONS_00211988	XLOC_017705	-	lincRNA	318	chr3	12815649	12818121	-	2	0	0	0	0	0	0.604814	0.539895	0	1.01551
TCONS_00214024	XLOC_017707	-	intronic_lncRNA	2648	chr3	13039668	13059241	-	2	0.95914	1.24104	1.423	1.77758	0.928681	2.01072	1.04781	2.10048	2.23933
TCONS_00207680	XLOC_017712	-	lincRNA	4806	chr3	14078690	14088679	-	2	0.77605	0.39841	0.30201	1.99993	3.54456	7.72102	0.340937	0.78354	0.362927
TCONS_00203139	XLOC_016950	-	anti-sense_lncRNA	893	chr3	14186112	14189501	+	3	1.12347	3.08652	2.64204	0.555133	1.15944	0.81394	2.12967	1.28774	0.960585
ENST00000428681.3	ENSG00000228242.3	AC093495.4	anti-sense_lncRNA	947	chr3	14186223	14189662	+	3	0.86696	0.37043	0.74847	0	0	0	0	0	0.739999
TCONS_00196551	XLOC_017715	-	lincRNA	1840	chr3	14409876	14412022	-	2	3.23547	1.76826	1.4895	0.351314	1.50034	0.815083	0.855198	0.68715	0.634045
TCONS_00196578	XLOC_017736	-	intronic_lncRNA	7157	chr3	17611591	17628550	-	2	5.21216	3.30597	5.68494	3.04314	4.61215	4.48462	3.31603	3.92833	3.8181
TCONS_00199257	XLOC_016971	-	lincRNA	2715	chr3	18506367	18572715	+	4	0.89302	1.06745	1.82815	1.2722	1.61233	2.01301	1.08929	1.0541	1.90423
TCONS_00197558	XLOC_016977	-	lincRNA	378	chr3	20227815	20235883	+	2	1.13366	3.6718	1.63832	3.26546	0.710655	0.800311	0.697526	1.8944	1.66746
TCONS_00197569	XLOC_016987	-	anti-sense_lncRNA	1244	chr3	24129253	24231825	+	4	2.53938	0.33939	1.20705	0.0613594	0.64942	0.269802	0.224853	1.62444	0
TCONS_00204802	XLOC_017007	-	lincRNA	1587	chr3	30333951	30341154	+	2	0.51487	0.35718	0.15111	1.33878	0.488162	1.06552	0.37971	0.290478	0.625137
TCONS_00205678	XLOC_017008	-	lincRNA	743	chr3	30391827	30431337	+	2	1.87428	0.6557	0.64973	0.945357	0.570423	0.649917	1.09255	1.19977	1.39258
TCONS_00195765	XLOC_017014	-	lincRNA	2044	chr3	32039671	32045560	+	2	1.60501	0.72852	1.66437	1.38848	2.46761	1.25883	1.58326	2.13761	1.91028
TCONS_00205711	XLOC_017034	-	lincRNA	7658	chr3	37420906	37431348	+	2	3.39664	3.30129	3.1126	2.58996	3.50418	3.74986	1.74512	3.04089	3.35283
TCONS_00201271	XLOC_017772	-	anti-sense_lncRNA	764	chr3	37845314	37903271	-	6	1.98524	4.41735	2.87571	2.95763	1.53711	1.7517	1.89177	1.58723	2.37115
TCONS_00195809	XLOC_017051	-	lincRNA	1229	chr3	39219394	39221197	+	2	2.37814	2.54831	3.13004	3.9221	1.91692	2.46371	2.11056	2.0408	1.63136
TCONS_00196638	XLOC_017787	-	anti-sense_lncRNA	4152	chr3	39540284	39544663	-	2	2.00882	2.88333	1.22029	0.444297	0.434079	0.249112	1.4329	1.11636	1.11147
TCONS_00196639	XLOC_017787	-	anti-sense_lncRNA	3867	chr3	39540358	39544416	-	2	9.44739	2.20606	3.9791	0.445824	1.34036	0.9775	2.77238	0.853567	2.02547
TCONS_00199315	XLOC_017063	-	lincRNA	2547	chr3	40807068	40817129	+	2	0.17398	2.43862	0.53441	0.245401	0.23564	0.119848	0.248439	0.376617	0.614717
TCONS_00200592	XLOC_017067	-	lincRNA	1889	chr3	40912863	40915591	+	2	0.60418	1.84242	0.12395	0.227407	0.0728332	0.16661	0.242144	0.619454	0.341995
TCONS_00199333	XLOC_017081	-	lincRNA	2137	chr3	44145551	44154836	+	2	0.5796	1.49637	0.97206	0.165219	0.126962	0.653626	0.39176	0.87192	0.834631
TCONS_00199925	XLOC_017819	-	lincRNA	682	chr3	45954377	45957301	-	2	0.21104	1.33134	0.73348	1.5982	0.257389	0.293046	0.493747	0.33865	1.6917
TCONS_00213758	XLOC_017100	-	lincRNA	632	chr3	46042409	46043168	+	2	0.9414	0.16527	1.14819	0	0.575151	0.490753	0.966931	0.946771	1.21503
TCONS_00196678	XLOC_017823	-	anti-sense_lncRNA	1486	chr3	46405474	46413157	-	2	1.1898	1.26617	1.79363	0.995971	0.718188	0.875711	1.36605	1.06576	1.57371
ENST00000435419.1	ENSG00000224424.7	PRKAR2A-AS1	lincRNA	843	chr3	48885005	48885926	+	2	0.79188	0.11057	0.54729	0.698165	0.480921	0.548431	0.459697	0.252609	0.180634
TCONS_00207377	XLOC_017153	-	lincRNA	491	chr3	50650980	50651773	+	2	0.69709	0.98738	0.24595	2.43279	0.859317	1.46131	1.66332	0	0.403349
ENST00000609204.1	ENSG00000273356.1	RP11-804H8.6	lincRNA	845	chr3	50708123	50709161	+	2	1.10521	1.54316	0.54557	1.19313	1.1506	1.53083	0.549893	1.13317	1.17045
TCONS_00199419	XLOC_017176	-	anti-sense_lncRNA	445	chr3	52568013	52569111	+	2	0	1.76517	1.17513	1.57795	0	0.579487	0.746687	1.01839	0.240389
TCONS_00196901	XLOC_017947	-	lincRNA	481	chr3	63690946	63697525	-	2	1.4434	0.2558	1.78482	0	0.445256	0.756889	0.215636	0.58909	0.626978
ENST00000485770.1	ENSG00000243993.1	RP11-14D22.2	lincRNA	341	chr3	64430158	64431152	-	2	1.42458	2.58917	2.08996	2.29246	1.35406	1.01232	4.02222	1.81345	1.6941
TCONS_00202192	XLOC_017222	-	lincRNA	11403	chr3	64998292	65011634	+	2	12.2685	7.49388	9.29434	2.68945	2.257	1.64112	3.79464	3.4675	2.86953
TCONS_00203387	XLOC_017222	-	lincRNA	5174	chr3	65003580	65009660	+	2	1.63374	3.04584	1.33195	0.680144	0.144779	0.470788	0.548882	0.710159	0.230663
TCONS_00199461	XLOC_017224	-	lincRNA	391	chr3	65099241	65112274	+	2	1.58636	3.79549	0.76107	0.337966	0.330516	0	0.323632	1.31983	0.620435
TCONS_00202751	XLOC_017956	-	intronic_lncRNA	1276	chr3	65705713	65712455	-	2	3.69614	1.05363	2.73205	1.48847	2.9782	3.7301	1.09048	1.42567	2.25895
TCONS_00202755	XLOC_017959	-	lincRNA	2625	chr3	66633663	66647914	-	2	0.50516	0.64099	1.12051	0.184723	0.278719	0.0579945	0.384652	0.530119	0.428328
TCONS_00204090	XLOC_017961	-	lincRNA	1150	chr3	67291535	67292913	-	2	0.9658	0.6717	0.95882	0.741871	1.7527	0.667286	1.05139	2.04202	1.3411
TCONS_00199462	XLOC_017228	-	lincRNA	5031	chr3	67705148	67805032	+	5	4.69319	2.3082	4.34758	1.15199	2.1978	2.735	2.72052	3.3531	3.35266
TCONS_00196017	XLOC_017229	-	lincRNA	2984	chr3	67890078	67896454	+	2	1.68694	0.68503	0.82547	0.367742	0.551601	0.631363	0.397617	0.692295	0.642362

TCONS_00198635	XLOC_017968	-	intronic_lncRNA	5451	chr3	71082789	71088901	-	2	5.58223	2.59376	4.55544	5.60364	4.92015	5.46054	2.75709	3.72574	3.96228
TCONS_00202769	XLOC_017973	-	lincRNA	240	chr3	72734915	72741438	-	2	4.22319	3.24156	5.09672	1.41089	0	3.13672	0	0	1.34056
TCONS_00196024	XLOC_017236	-	lincRNA	261	chr3	72897711	72936318	+	2	3.07132	3.48224	0	0	0	1.12413	1.04524	0	0.955114
TCONS_00196029	XLOC_017238	-	lincRNA	2296	chr3	74662890	74673544	+	3	1.46094	0.74186	2.66078	0.335885	0.586522	0.603981	0.584559	0.115069	0.385574
TCONS_00202206	XLOC_017242	-	lincRNA	6755	chr3	75549381	75587510	+	2	1.66843	0.59111	2.67673	0.386236	0.578249	1.39269	0.57931	0.990135	0.649665
TCONS_00211775	XLOC_017242	-	lincRNA	3766	chr3	75552084	75561552	+	2	4.52231	2.10032	3.71941	0.294548	0.950782	1.00705	2.94879	1.21573	2.47558
TCONS_00202213	XLOC_017255	-	lincRNA	2005	chr3	87841487	87872656	+	2	1.63965	1.01852	1.00485	2.30509	0.91986	0.584579	0.550018	0.802298	0.863861
TCONS_00205378	XLOC_018001	-	lincRNA	462	chr3	98433177	98434643	-	3	0	0.54945	0	0.737435	0	0	1.39201	0	0.448933
TCONS_00212172	XLOC_018004	-	lincRNA	888	chr3	99141706	99143366	-	3	1.48049	0.72297	0.81764	0.186393	0.449204	1.22982	2.3171	0.35379	0.337447
TCONS_00215527	XLOC_017270	-	anti-sense_lncRNA	9847	chr3	99739800	99806573	+	2	2.09665	1.27223	1.00116	1.19997	1.36701	1.58048	0.596049	0.921815	1.32567
TCONS_00212175	XLOC_018010	-	intronic_lncRNA	2958	chr3	100529914	100534675	-	3	2.92443	2.25377	2.02007	0.835224	0.623629	0.662814	1.5844	0.902672	0.334673
TCONS_00212177	XLOC_018009	-	intronic_lncRNA	1084	chr3	100553326	100557737	-	4	4.84274	1.04312	1.90361	0.434933	0.628136	0.557864	2.1289	1.37256	0.262156
TCONS_00202808	XLOC_018015	-	lincRNA	4351	chr3	104076057	104239528	-	3	0.62051	0.01024	1.49155	0.639218	0.594635	1.08758	1.06905	1.65739	2.26567
TCONS_00207901	XLOC_018015	-	lincRNA	4636	chr3	104076064	104175877	-	2	0.36324	0.46775	1.91E-05	0.454591	0.218473	0.249857	0.20617	1.41455	0.842911
TCONS_00212186	XLOC_018022	-	lincRNA	363	chr3	106404560	106407612	-	2	4.94468	2.23242	2.24519	0.792858	0.388957	1.3121	1.91492	1.03865	0.730061
TCONS_00200131	XLOC_018024	-	lincRNA	2628	chr3	106468575	106559771	-	2	2.18633	1.83328	1.52085	0.448059	0.354294	0.492344	0.552251	0.364005	0.261432
TCONS_00202813	XLOC_018025	-	lincRNA	11391	chr3	106528002	106539791	-	2	2.89369	1.44955	2.297	0.48576	0.515167	0.897782	0.653832	0.536843	0.458098
TCONS_00200132	XLOC_018026	-	lincRNA	619	chr3	106561820	106562549	-	2	1.6983	1.19323	0.33845	0.153299	0.44493	0.506074	0.142545	0	1.11398
TCONS_00212188	XLOC_018030	-	anti-sense_lncRNA	787	chr3	107018907	107140357	-	4	0	0	0.34072	0.207398	0.220679	0	1.28127	0	0.207321
TCONS_00202817	XLOC_018030	-	lincRNA	455	chr3	107099451	107103554	-	3	3.57587	1.69453	2.58249	0.783637	1.69798	1.66963	3.17457	2.60538	1.82443
TCONS_00205933	XLOC_017307	-	lincRNA	1797	chr3	109826567	109829339	+	3	0.44726	0.39864	0.21855	1.5634	0.231132	1.27763	0.3294	0.70576	1.59177
TCONS_00200138	XLOC_018047	-	lincRNA	371	chr3	110787985	110790375	-	2	0.58966	1.70039	0.42711	0.755558	0.370277	1.25013	1.45581	1.48173	0.695025
TCONS_00196096	XLOC_017317	-	lincRNA	726	chr3	112020886	112025810	+	2	1.74115	0.54169	0.13422	0	0.235624	0.402616	0.451477	0.309811	0.221241
TCONS_00211812	XLOC_017320	-	lincRNA	1626	chr3	112520994	112523261	+	2	0.00206	2.45E-05	0.88878	0.220727	4.55019	2.69397	0.645535	0.320296	1.71636
TCONS_00204981	XLOC_017320	-	lincRNA	1722	chr3	112520994	112523261	+	3	0.86429	0.5113	0.0001	0.66107	1.7391	0.0013027	1.04748	2.76808	2.44086
ENST00000519700.1	ENSG00000242770.2	RP11-180K7.1	lincRNA	592	chr3	112521443	112522451	+	2	1.05734	0	0.69799	1.37169	6.27648	4.21773	0	1.0488	5.26816
TCONS_00201551	XLOC_018068	-	intronic_lncRNA	4533	chr3	114300532	114305836	-	2	7.56566	5.27795	6.98343	5.97935	5.35477	5.87196	3.62477	5.64598	5.21525
TCONS_00212212	XLOC_018073	-	lincRNA	4920	chr3	117571653	117716424	-	2	1.92573	1.0467	2.34289	0.298162	3.06875	4.27348	2.98181	2.41267	0.940331
TCONS_00211230	XLOC_018081	-	lincRNA	1817	chr3	119383092	119386503	-	2	2.65039	1.31232	1.98563	1.18772	2.85329	3.13274	1.40961	2.24029	1.71492
TCONS_00202849	XLOC_018091	-	lincRNA	1246	chr3	120530396	120543596	-	2	0.87734	0.40649	1.07086	0.183726	0.235699	0.0673213	0.89767	0.308821	0.442677
ENST00000511301.1	ENSG00000250012.1	RP11-124N2.1	intronic_lncRNA	327	chr3	125803063	125804295	+	2	0	1.15193	2.91325	1.0178	0	2.81048	0	0.674246	0.471241
TCONS_00198809	XLOC_018137	-	intronic_lncRNA	1221	chr3	128210058	128211374	-	2	1.99728	1.3189	1.57739	1.69409	0.724507	0.620765	0.574946	0.712017	0.510272
TCONS_00215575	XLOC_017392	-	lincRNA	717	chr3	128903233	128915541	+	2	0.78726	0.27561	1.09275	0.248268	0.479542	0.955874	0.459524	0.472955	1.12567
TCONS_00207515	XLOC_017400	-	lincRNA	324	chr3	129898440	129920425	+	2	0.80682	1.77026	1.79191	0.521128	0.514657	1.72702	0	0	0.48284
TCONS_00200231	XLOC_018153	-	anti-sense_lncRNA	328	chr3	130363509	130366860	-	2	0	3.42841	0	0	0.996546	0	0	0	0
TCONS_00210155	XLOC_018166	-	intronic_lncRNA	2004	chr3	133079390	133082258	-	2	0.396	0.31357	0.11601	0.0709652	0.102264	0.155974	0.453209	0.356776	1.05642
TCONS_00210161	XLOC_018180	-	anti-sense_lncRNA	5561	chr3	137737501	137750668	-	2	6.30599	3.75149	4.6434	2.24317	2.07187	2.70044	2.45096	2.03396	3.04237
TCONS_00196244	XLOC_017447	-	lincRNA	391	chr3	138695928	138700170	+	2	2.11514	0	0	0	0	0	0.970896	0	0.310218
TCONS_00196249	XLOC_017451	-	intronic_lncRNA	412	chr3	140053794	140078624	+	2	2.86213	0.68219	1.0241	0	0	0.335348	0.289826	0	0.5575
TCONS_00209809	XLOC_017457	-	lincRNA	302	chr3	141439828	141448773	+	3	0	0	1.46077	0	0	0.698245	1.25821	1.69128	0.587819
TCONS_00205060	XLOC_017466	-	lincRNA	1655	chr3	143956704	143990624	+	3	1.54264	1.26445	0.76802	1.0121	0.67674	0.483634	1.28646	0.996534	0.714971
TCONS_00199661	XLOC_017466	-	lincRNA	866	chr3	144186933	144190130	+	2	1.83509	0.74705	1.26752	1.05912	1.02117	0.95295	0.709646	0.853167	0.174344
TCONS_00199662	XLOC_017467	-	lincRNA	289	chr3	144241381	144242302	+	2	1.10352	4.10039	0.83805	0.719745	0.71713	0	1.44795	0.970587	0
ENST00000482351.1	ENSG00000244358.1	RP11-88H10.2	lincRNA	624	chr3	145657755	145675618	+	2	0.23977	0	0.33437	0.151483	3.22382	2.00028	0	0	0
TCONS_00200297	XLOC_018213	-	lincRNA	382	chr3	148991287	148991952	-	2	0	0	0.80038	0.3548	0.694618	1.17381	1.02189	0.462715	1.30388
ENST00000471093.1	ENSG00000240137.1	RP11-103G8.2	anti-sense_lncRNA	302	chr3	150421351	150437775	+	3	11.6648	8.61197	10.2254	2.52559	8.1482	6.2842	5.03282	6.76512	2.9391
TCONS_00215946	XLOC_018236	-	anti-sense_lncRNA	2244	chr3	151143202	151150712	-	2	4.1477	1.91966	1.69889	0.409597	0.677842	0.849459	1.80309	0.960087	1.77787
TCONS_00204490	XLOC_018236	-	anti-sense_lncRNA	2579	chr3	151146653	151150581	-	2	3.90222	1.88565	1.32364	0.347519	0.580164	0.659555	1.12348	0.729316	0.923049
TCONS_00201765	XLOC_018236	-	lincRNA	8017	chr3	151252632	151322016	-	3	7.73584	2.79761	4.01859	1.1864	1.96978	2.18431	2.20741	2.12198	2.35115
TCONS_00209354	XLOC_018239	-	lincRNA	1552	chr3	151891208	151923258	-	2	0.67908	0.52353	1.03364	1.13668	1.54809	1.14521	0.173168	0.53649	1.02622
TCONS_00202395	XLOC_017497	-	lincRNA	2141	chr3	151921721	151964213	+	2	2.1033	1.74823	3.41314	2.67103	2.85073	2.42792	1.17284	4.06053	7.10945
TCONS_00204494	XLOC_018243	-	lincRNA	1205	chr3	152212976	152215272	-	3	3.75367	0.7758	2.22987	0.063745	0.736107	1.05112	0.934736	0.723459	1.2097
TCONS_00211900	XLOC_017501	-	lincRNA	662	chr3	152921440	152969555	+	4	0.44028	1.69847	0.76585	1.25075	0.671674	0.458705	3.60986	0.884029	0.630652
ENST00000462300.1	ENSG00000241912.1	RP11-292E2.2	lincRNA	382	chr3	153094620	153095351	+	2	0.55456	1.19638	2.00094	0	0	0.782538	0.34063	1.38814	1.95582
TCONS_00214313	XLOC_018245	-	lincRNA	404	chr3	153659535	153661048	-	2	0.49551	0.3548	0.71061	0	1.85352	1.04593	0	1.23215	0.289959
TCONS_00208041	XLOC_018248	-	lincRNA	339	chr3	154968518	154976648	-	2	0	0	0	0	0	0	1.36128	0	0
ENST00000492937.1	ENSG00000243926.1	TIPARP-AS1	anti-sense_lncRNA	441	chr3	156389651	156392167	-	2	0.84036	2.69265	2.39067	1.06955	2.34391	1.76761	4.5582	2.4172	1.95574
TCONS_00209829	XLOC_017516	-	lincRNA	2200	chr3	156892078	156894531	+	2	2.57163	1.9382	3.24228	2.27539	2.54649	2.32927	2.18853	3.15328	2.2516
ENST00000460796.1	ENSG00000243176.1	RP11-550I24.2	lincRNA	542	chr3	156893012	156957753	+	2	0.77442	2.96626	1.04161	0.732956	0.210363	1.60723	1.23061	1.34045	1.3689
TCONS_00212315	XLOC_018263	-	lincRNA	273	chr3	158350633	158352077	-	2	1.31826	0	0	0	0.86655	0	0	0	1.62405
TCONS_00198985	XLOC_018268	-	intronic_lncRNA	2976	chr3	160060219	160067022	-	2	2.79514	1.22134	2.18254	1.08336	1.48256	2.05152	1.1963	1.38859	1.95343

TCONS_00198125	XLOC_017527	-	lincRNA	464	chr3	160283600	160340668	+	2	2.30334	1.363	1.3597	0.731811	0.474537	1.61218	1.8413	0.628362	1.78185
TCONS_00202980	XLOC_018272	-	lincRNA	268	chr3	162408072	162408655	-	2	1.4017	2.10925	1.08708	0	0	0	0	2.51938	0
TCONS_00201801	XLOC_018277	-	lincRNA	534	chr3	166620855	166647343	-	3	0	0.42911	0	0	0.746809	0.211956	0.900835	0	0.17528
TCONS_00200332	XLOC_018277	-	lincRNA	560	chr3	166678511	166689483	-	2	1.69284	1.59059	0.3953	0.535663	0.865022	0.393077	0.666841	0.456451	0.162425
TCONS_00197271	XLOC_018278	-	lincRNA	10260	chr3	166792966	166817509	-	3	4.88131	1.35624	1.69243	0.245037	0.809641	1.3185	1.83783	1.77726	1.76209
TCONS_00198995	XLOC_018278	-	lincRNA	7767	chr3	166795474	166858543	-	5	4.49881	1.40857	2.97168	0.455267	0.986355	0.596966	1.54884	0.607488	1.95934
TCONS_00211450	XLOC_018278	-	lincRNA	1323	chr3	166805009	166858557	-	4	13.0785	3.37332	5.81998	0.604267	2.2792	2.44069	3.70454	2.40456	3.75804
TCONS_00199707	XLOC_017547	-	lincRNA	425	chr3	170130370	170131014	+	2	0	0.32095	0	0.286546	0	0.315773	0.272201	1.48346	0.524535
TCONS_00198140	XLOC_017547	-	lincRNA	1519	chr3	170425861	170579167	+	2	1.62411	0.64401	0.68879	0.388386	0.60679	0.21344	0.621392	1.10002	0.350667
TCONS_00213573	XLOC_018294	-	lincRNA	4740	chr3	170427443	170454040	-	2	1.97955	2.06766	3.06409	0.71861	1.02747	4.83004	1.28114	1.99622	0.799902
TCONS_00204613	XLOC_018301	-	intronic_lncRNA	2998	chr3	171411220	171414339	-	2	6.16668	2.22149	4.10665	2.33262	3.68818	4.57331	2.22796	1.57854	2.35039
TCONS_00196351	XLOC_017552	-	lincRNA	2737	chr3	171700776	171715447	+	2	2.17296	1.22526	1.12579	0.100893	0.290604	0.33259	0.505436	0.538294	0.523093
TCONS_00202428	XLOC_017554	-	intronic_lncRNA	420	chr3	172409770	172411122	+	2	0.91983	0.98523	0.98544	0.586214	0	0.323035	1.11489	0.379639	0.805115
TCONS_00211925	XLOC_017558	-	lincRNA	603	chr3	174158731	174410141	+	4	0.99309	0.60819	1.06917	0.189684	0.707455	0.556857	1.75372	0.203328	0.155272
TCONS_00196360	XLOC_017558	-	lincRNA	3742	chr3	174158770	174458987	+	4	0.63175	0.26398	0.15754	7.80E-05	0.002701	0.225034	0.531454	0	0
TCONS_00201029	XLOC_017558	-	lincRNA	898	chr3	174240942	174271889	+	3	0.63904	1.8747	0.69408	1.74532	0.907381	0.884905	1.13123	0.926941	0.704588
TCONS_00201030	XLOC_017558	-	lincRNA	855	chr3	174241136	174271977	+	4	2.54995	1.51205	1.6578	0	0.692236	0.807015	0.680735	1.85503	0.78196
TCONS_00213957	XLOC_017558	-	lincRNA	4594	chr3	174241152	174459768	+	3	0	0.06975	0.00017	0	0	0.0001074	7.77E-05	0.0078061	0.534783
TCONS_00207596	XLOC_017558	-	lincRNA	1870	chr3	174241246	174250867	+	2	1.71092	1.05878	1.37903	0.536678	1.32597	1.3902	0.9097	0.578352	0.933936
TCONS_00199716	XLOC_017558	-	lincRNA	7458	chr3	174241247	174462664	+	4	3.49541	1.66832	2.18891	1.74314	2.14425	1.53184	1.46436	1.98343	1.15798
TCONS_00208641	XLOC_017561	-	lincRNA	1790	chr3	177468882	177485108	+	4	1.92524	0.80081	0.70245	10.7102	4.91391	7.65531	6.28636	3.49379	3.88799
TCONS_00205099	XLOC_017564	-	lincRNA	3514	chr3	177613418	177617718	+	2	0.40055	0.38353	0.08401	0.984607	0.463234	1.40002	0.650022	0.605485	0.382841
TCONS_00209848	XLOC_017564	-	lincRNA	639	chr3	177624618	177643071	+	2	0.23162	0	0	0.292569	0.28293	1.93152	0.679342	0.931348	0.531293
ENST00000418585.1	ENSG00000223930.1	RP11-33A14.1	lincRNA	640	chr3	178052810	178103164	-	3	0.46218	0	0.48295	1.98829	1.69367	1.92711	2.46026	0.185837	0.132517
TCONS_00203001	XLOC_018314	-	lincRNA	688	chr3	178100527	178103325	-	2	0.41692	0.43826	1.01402	1.23345	0.508374	0.723559	1.80992	0.334405	0.596666
TCONS_00196366	XLOC_017565	-	lincRNA	4542	chr3	178137060	178205342	+	2	4.49236	0.63399	0.88113	0.235731	0.791653	1.00361	0.468902	0.609608	0.570894
TCONS_00197309	XLOC_018316	-	lincRNA	768	chr3	178625728	178627428	-	2	1.61289	0.75188	0.37241	0.112951	0.109013	0	0.313027	0.573041	0.614161
TCONS_00210231	XLOC_018324	-	lincRNA	6439	chr3	180424839	180459706	-	5	0.63619	0.27191	3.56E-05	0.0099489	0.0003959	1.95493	6.93E-05	0.0006307	0.0002101
TCONS_00200365	XLOC_018324	-	lincRNA	6460	chr3	180424839	180459706	-	5	0.71385	1.23027	1.11902	1.17141	1.87136	1.94249	0.971865	0.592521	0.475886
TCONS_00200366	XLOC_018324	-	lincRNA	4026	chr3	180426667	180458980	-	6	0.54461	0.79508	0.13765	0.664944	1.26239	0.980874	0.3752	0.0174502	0.0671075
TCONS_00202442	XLOC_017574	-	lincRNA	685	chr3	180774494	180893864	+	3	2.51693	0.88201	1.89504	0.529425	0.383667	1.16489	0.245308	0.504771	0.36024
TCONS_00202443	XLOC_017575	-	lincRNA	352	chr3	181281397	181319130	+	2	2.64761	0.47958	1.44917	1.27574	0	0.4693	0	0.558762	0
TCONS_00202469	XLOC_017598	-	lincRNA	362	chr3	184225971	184226454	+	2	0	0.44931	0	0	0	0	1.1565	0	0
TCONS_00213997	XLOC_017615	-	lincRNA	482	chr3	187661749	187662466	+	2	2.15748	1.52924	2.79447	0	0.443645	0.251394	1.71871	1.7608	0.624711
ENST00000444488.1	ENSG00000234076.1	TPRG1-AS1	lincRNA	697	chr3	188659504	188665428	-	2	3.27522	4.5892	11.9439	1.16234	3.74297	2.55739	4.78475	1.80529	1.17148
TCONS_00201098	XLOC_017618	-	lincRNA	2323	chr3	188839138	188841750	+	2	1.10582	0.7657	1.54324	1.38673	0.694851	1.22571	0.934324	0.71946	0.652557
TCONS_00203048	XLOC_018381	-	lincRNA	444	chr3	193307798	193310893	-	2	0.83013	0.29543	1.77017	0	0.514319	1.45471	0.499933	0.681817	1.20698
TCONS_00196470	XLOC_017629	-	lincRNA	328	chr3	193509192	193509739	+	2	1.56489	0	0	0	0.498273	0	0	0	0
ENST00000445168.1	ENSG00000229155.1	RP11-528A4.2	lincRNA	788	chr3	193560600	193561813	-	2	1.03882	0.48404	1.07865	0.109097	1.36849	0.239983	0.604314	0.829854	0.889609
TCONS_00197412	XLOC_018406	-	anti-sense_lncRNA	379	chr3	195941054	195946320	-	3	1.12743	0.81128	1.62877	0.360788	0.353289	0	0.693388	0	0.663158
TCONS_00225312	XLOC_018432	-	lincRNA	3326	chr4	190706	194140	+	2	1.27386	1.26508	1.24712	0.961986	1.82705	1.61923	0.968698	1.36106	1.05167
TCONS_00222099	XLOC_018991	-	lincRNA	1650	chr4	300556	331172	-	2	0.56289	0.29281	1.30053	0.441565	0.551754	0.436774	0.847173	0.499996	0.239149
TCONS_00220737	XLOC_018457	-	lincRNA	1201	chr4	2464760	2467160	+	3	2.13896	2.12431	1.67915	0.320001	1.04701	1.05533	1.70106	1.04921	0.983213
TCONS_00218725	XLOC_018462	-	anti-sense_lncRNA	2429	chr4	2936496	2939915	+	3	2.78104	2.04151	1.66621	1.86976	2.41731	1.03199	1.32844	1.91266	1.52133
TCONS_00226964	XLOC_018462	-	lincRNA	1806	chr4	2936655	2939610	+	2	2.93994	1.83257	2.72561	1.0714	1.77456	2.50982	2.30117	1.8525	2.63492
TCONS_00227507	XLOC_018471	-	lincRNA	1774	chr4	4037173	4076484	+	4	10.6315	10.9661	9.80174	1.83031	4.92495	2.41385	9.32187	3.2226	5.10227
TCONS_00221780	XLOC_018489	-	lincRNA	353	chr4	6765225	6769312	+	2	0	0.95275	1.91903	0	0	0.466209	0	1.10987	0
TCONS_00217466	XLOC_018499	-	lincRNA	416	chr4	8512435	8517158	+	2	0.46829	1.0039	1.67399	0.597152	0.582674	0	0	0.386961	1.09385
TCONS_00219349	XLOC_019061	-	lincRNA	2754	chr4	9035403	9075423	-	3	3.7574	3.29167	2.31836	0.576269	1.51549	0.908528	2.30489	2.07589	1.55881
TCONS_00218084	XLOC_019062	-	lincRNA	796	chr4	9068157	9071399	-	2	2.22067	1.79057	2.12792	0.215256	1.03842	0.710243	0.198678	1.22781	1.07257
TCONS_00219350	XLOC_019063	-	lincRNA	929	chr4	9152601	9153982	-	2	0.97823	1.26658	1.63858	0.439707	1.10173	0.967002	0.323644	1.11212	0.477484
TCONS_00218085	XLOC_019065	-	lincRNA	2851	chr4	9600688	9606655	-	2	3.54301	1.35899	1.97051	0.337929	1.3904	0.424365	0.92324	0.908937	0.848761
TCONS_00219941	XLOC_018506	-	lincRNA	3743	chr4	11470662	11479771	+	3	1.55451	0.82549	7.05E-05	0	0.671818	0.569476	0.0001266	0	0.518347
TCONS_00222518	XLOC_018506	-	lincRNA	1028	chr4	11470858	11553932	+	2	1.33615	5.45562	1.91021	2.99825	2.11147	3.968	4.12934	3.52032	6.27546
ENST00000515343.1	ENSG00000251152.1	RP11-281P23.1	lincRNA	4019	chr4	11470874	11479820	+	2	0.54034	3.82501	1.44762	2.95275	3.48692	3.21491	6.04799	6.72269	7.1034
TCONS_00228001	XLOC_019079	-	intronic_lncRNA	818	chr4	15362400	15364353	-	2	0.32952	0.46034	0.68369	0.830279	0.300334	1.3697	1.05306	0.525969	0.376015
TCONS_00225659	XLOC_019084	-	lincRNA	437	chr4	16126338	16127434	-	2	0.42721	0.30434	0	0	0.264931	0.299624	0	2.45957	0.497376
TCONS_00222527	XLOC_018523	-	lincRNA	1039	chr4	16289204	16325006	+	4	1.43165	1.78044	1.14965	0.0257509	0.821055	2.00489	2.89608	1.56612	0.769087
TCONS_00222528	XLOC_018523	-	lincRNA	880	chr4	16289249	16324839	+	5	3.32735	4.17479	2.09442	0.722578	0.62167	0.531678	2.23325	0.807345	1.01294
TCONS_00220371	XLOC_019087	-	lincRNA	305	chr4	17078892	17079386	-	2	0.94584	2.09197	3.54544	0	0	0	0	1.64185	0
TCONS_00220788	XLOC_018529	-	intronic_lncRNA	12307	chr4	20398569	20411864	+	2	13.6524	7.78267	6.34057	1.01422	0.942213	1.93823	1.20383	1.42904	1.07045

TCONS_00229101	XLOC_018551	-	lincRNA	678	chr4	26846604	26849619	+	2	2.76642	0.29836	0.73973	0.671511	0.519137	0.443267	1.12047	0.68308	0.365577
TCONS_00220393	XLOC_019116	-	lincRNA	2293	chr4	36013528	36049457	-	4	2.73099	1.11439	2.39815	0.461067	0.54014	0.680391	1.31019	2.72693	2.39951
TCONS_00223944	XLOC_019116	-	lincRNA	510	chr4	36014141	36020962	-	2	0	0	0	0.398864	0.928155	1.08581	0	0.26812	0
TCONS_00223119	XLOC_019117	-	lincRNA	1701	chr4	36270623	36283136	-	3	8.69117	5.15862	5.13904	0.810431	1.59546	1.2068	5.20013	2.84618	3.42125
TCONS_00219413	XLOC_019117	-	lincRNA	1081	chr4	36275206	36283184	-	2	2.56153	0.85029	1.87306	0.290945	0.916137	0.420386	1.437	1.83223	0.534894
TCONS_00225364	XLOC_018563	-	lincRNA	284	chr4	38157110	38158816	+	2	1.1633	0	2.66244	0	3.03406	0.841551	1.53525	1.02796	1.42208
TCONS_00218840	XLOC_018567	-	lincRNA	2102	chr4	38749953	38754549	+	3	1.04372	2.16304	4.40751	2.63662	0.82371	0.772211	1.43774	3.94488	7.29692
TCONS_00221822	XLOC_018567	-	lincRNA	2571	chr4	38752213	38755475	+	2	1.78889	0.94789	1.93369	0.0715688	0.246695	0.092507	1.01212	1.10866	2.2548
TCONS_00219996	XLOC_018573	-	lincRNA	397	chr4	39640749	39643544	+	2	0.51289	1.47085	0.73693	0.655196	0.320188	0.722336	1.25281	0.851917	0.601058
ENST00000510551.1	ENSG00000249241.1	AC195454.1	lincRNA	957	chr4	40318502	40332436	+	3	1.19886	0.41792	1.18977	0.115904	0.870258	0.155302	0.228117	0.456373	0.459277
TCONS_00220004	XLOC_018578	-	lincRNA	454	chr4	40318519	40332403	+	3	1.23358	1.28864	4.5782	0.413704	1.06964	1.21029	0.256563	0.240902	0.0012579
TCONS_00224870	XLOC_019135	-	lincRNA	2003	chr4	43980861	44027709	-	2	0.90564	0.35296	0.23215	2.27215	0.648029	2.45796	0.226731	0.580084	0.352335
TCONS_00229926	XLOC_018587	-	lincRNA	367	chr4	44509319	44510003	+	2	0	0.87107	0	0	0	0	0	1.51892	0
TCONS_00218882	XLOC_018599	-	lincRNA	275	chr4	49104151	49110227	+	2	3.86209	2.89313	2.97264	0	0	0	2.57723	1.148	0
TCONS_00225704	XLOC_019151	-	lincRNA	861	chr4	49289048	49302226	-	2	0.46223	0	0.10644	0	0.187085	0	0.804503	0.122816	0
TCONS_00226575	XLOC_019162	-	lincRNA	2098	chr4	54488288	54649343	-	3	1.35253	0.427	0.19978	0.332828	0.43999	0.608984	0.344135	0.332164	1.27866
TCONS_00224899	XLOC_019162	-	lincRNA	585	chr4	54540615	54648861	-	3	0	0.5722	0	2.00035	2.56463	1.70572	0.524107	0.480584	7.7995
TCONS_00227308	XLOC_019162	-	lincRNA	1100	chr4	54561526	54648737	-	2	0.4138	0.24736	0.46964	0.458889	1.06699	1.11105	0.0988062	0.614938	2.11454
TCONS_00223977	XLOC_019162	-	lincRNA	8859	chr4	54628785	54648739	-	2	0.74549	0.5321	0.70249	1.69663	0.991645	0.860852	0.375211	0.750092	1.25555
TCONS_00224909	XLOC_019172	-	anti-sense_lncRNA	730	chr4	56756389	56814216	-	4	0.38401	0.53756	0.70335	2.87464	0.394771	0.295389	0.67755	0.516024	1.4271
TCONS_00218217	XLOC_019172	-	lincRNA	1460	chr4	56805080	56808100	-	2	0.56668	0.6743	0.15086	0.419801	0.616925	0.993376	0.601977	0.873082	0.596635
TCONS_00220457	XLOC_019175	-	lincRNA	402	chr4	57391836	57396610	-	3	1.0007	3.22553	1.07691	0	0	0	0	0.414959	0.585806
TCONS_00222224	XLOC_019184	-	lincRNA	1701	chr4	61639023	61660766	-	2	0.74772	0.47137	3.30302	0.213271	0.245971	0.375029	1.16879	0.536557	0.500485
TCONS_00225715	XLOC_019189	-	lincRNA	4994	chr4	68204943	68242174	-	3	0.2255	0.11785	0.21316	0.496971	2.14246	1.05025	0.369975	0.58264	0.120234
TCONS_00224912	XLOC_019189	-	lincRNA	2589	chr4	68238204	68242367	-	2	0.5305	0.05901	0.73809	1.84176	0.273903	0.746483	0.158664	0.1066	0
TCONS_00229192	XLOC_018631	-	anti-sense_lncRNA	3127	chr4	68618228	68622302	+	2	1.11623	0.86866	0.64229	0.502786	1.5738	2.73824	0.35821	1.42646	1.89188
TCONS_00228605	XLOC_018631	-	lincRNA	872	chr4	68656136	68668590	+	4	1.21245	0.35027	0.02191	0.280097	0	0.95683	1.03693	1.81166	1.87486
TCONS_00218939	XLOC_018631	-	lincRNA	465	chr4	68661271	68668082	+	3	0.00013	0	1.06683	0	0	1.39176	0.0465761	0	0.445756
TCONS_00226112	XLOC_018631	-	lincRNA	660	chr4	68664324	68668555	+	2	0	0.57197	0.43882	0.0090222	1.75404	1.64046	0	0	0.0364777
TCONS_00227658	XLOC_018649	-	lincRNA	534	chr4	71544394	71552589	+	4	0	0.21454	0.21344	0.768594	0.560098	2.19615	0.464647	2.57204	1.15008
TCONS_00229953	XLOC_018649	-	lincRNA	712	chr4	71544650	71552589	+	3	0	7.72E-06	6.36E-06	0.2517	5.85E-06	0.91543	0.74503	1.84146	0.731927
TCONS_00223704	XLOC_018656	-	lincRNA	640	chr4	74215510	74219596	+	2	0.23109	0.32445	0.80492	0.291901	0.423418	1.12415	0.271102	0.371674	0.53007
TCONS_00222694	XLOC_018668	-	intronic_lncRNA	1231	chr4	75045690	75047739	+	2	1.78015	1.44352	1.62987	1.11842	1.3751	1.63928	1.19554	1.48843	1.23517
TCONS_00224009	XLOC_019223	-	lincRNA	568	chr4	76028662	76047227	-	2	4.96877	1.16662	4.25144	1.39722	1.52266	3.07577	1.62965	2.90078	2.85912
TCONS_00220501	XLOC_019231	-	lincRNA	460	chr4	76649084	76649642	-	2	2.72821	2.7688	3.03892	3.96345	2.16897	3.27444	5.61287	3.83024	3.8459
TCONS_00231365	XLOC_019241	-	lincRNA	4161	chr4	77159608	77172806	-	3	0.67159	0.33138	0	0	0.739443	0.164445	0.286556	2.01E-05	1.18342
TCONS_00222260	XLOC_019241	-	lincRNA	3734	chr4	77159970	77172812	-	2	3.40706	1.66379	4.33229	0.39814	0.0169463	0.750386	2.68982	1.16938	2.09203
TCONS_00228885	XLOC_019241	-	lincRNA	3701	chr4	77159996	77172770	-	3	0.00107	0.54709	6.09E-05	0.0001394	0.72456	0.0001614	2.49704	0.997167	0.156195
TCONS_00218305	XLOC_019246	-	lincRNA	613	chr4	77830087	77831628	-	2	1.72284	0.51891	0.17174	0.155536	0.451484	0	0.144678	0	0
TCONS_00227075	XLOC_018696	-	lincRNA	2191	chr4	80506310	80519754	+	2	2.05053	0.67458	0.84052	1.0287	0.833681	1.30708	0.674201	1.17127	0.376968
TCONS_00227078	XLOC_018699	-	intronic_lncRNA	2007	chr4	81302470	81304815	+	2	11.3519	2.77826	4.594	0.885598	1.83768	1.82965	1.90676	1.95899	2.52479
ENST00000514050.1	ENSG00000251331.2	RP11-689K5.3	lincRNA	472	chr4	82523951	82965397	-	4	0	0.26443	2.37316	0.236715	1.61102	3.38938	3.12327	0.60925	0.432096
TCONS_00222281	XLOC_019267	-	lincRNA	1162	chr4	84059774	84111030	-	2	2.50072	3.01463	2.33046	0.665999	0.705105	0.805371	2.19836	0.840125	2.21514
TCONS_00218339	XLOC_019267	-	lincRNA	700	chr4	84110157	84111421	-	2	1.70978	0.156	0	0.128371	0.124008	0.14122	0	0	0.256185
TCONS_00225762	XLOC_019272	-	lincRNA	1659	chr4	85501910	85503781	-	2	2.44749	1.26097	1.96262	0.35107	1.68719	1.54338	1.12254	1.71152	1.46562
TCONS_00229226	XLOC_018711	-	lincRNA	2071	chr4	85574246	85584352	+	2	1.69097	0.79355	1.11835	0.205264	0.295771	0.263167	0.93616	0.773855	1.41969
TCONS_00219619	XLOC_019274	-	lincRNA	1508	chr4	87373124	87515359	-	2	4.99122	5.35798	4.38144	0.783314	2.58887	2.63667	4.52095	2.7117	3.09425
TCONS_00218383	XLOC_019281	-	lincRNA	351	chr4	88813346	88814070	-	2	5.99571	3.37981	1.9456	0.856147	1.68291	0	2.07734	0	1.97409
TCONS_00218384	XLOC_019284	-	lincRNA	397	chr4	88921260	88924089	-	2	1.02578	1.10313	4.79004	1.63799	0	0	0.313202	0	2.70476
TCONS_00222306	XLOC_019287	-	intronic_lncRNA	1114	chr4	89340126	89342060	-	3	0.78069	0.31048	1.45718	0.490733	0.337599	0.231215	1.02904	1.06164	0.697123
TCONS_00224432	XLOC_018727	-	anti-sense_lncRNA	12708	chr4	89851254	89879402	+	3	0.45737	0.28543	0.18328	0.348547	0.326866	0.333792	0.570074	2.24248	2.51166
TCONS_00225464	XLOC_018727	-	intronic_lncRNA	691	chr4	89862653	89866835	+	2	0	0.20024	0	1.28526	0.16874	0.370316	2.68862	3.62456	5.76094
TCONS_00220107	XLOC_018729	-	lincRNA	788	chr4	90602609	90612996	+	2	0.69255	0.48404	0.11985	0	0	1.4399	0.201438	0.276618	0.296536
TCONS_00225788	XLOC_019308	-	lincRNA	746	chr4	100304624	100325981	-	3	0.93201	0.39124	0.51688	0.352554	0.907609	0.646329	0.977774	0.447398	0
TCONS_00218452	XLOC_019322	-	lincRNA	745	chr4	105412763	105416148	-	2	3.36138	0.15328	0.98942	0.147155	0.454646	0	0.544227	0.448225	0.258555
TCONS_00222337	XLOC_019322	-	lincRNA	445	chr4	105413063	105416072	-	3	0	0.83165	2.16148	0.460264	0	0	0.497792	2.07E-05	0.379179
TCONS_00221952	XLOC_018761	-	lincRNA	712	chr4	105510682	105541619	+	3	0.39746	0.13917	1.37951	0	0.484278	0.137894	0.23206	0	0.227357
ENST00000504082.1	ENSG00000251259.1	AC004069.2	lincRNA	549	chr4	106058437	106061776	-	2	0.29098	0.61556	0.40807	0.368425	0.357093	0.202779	0.344301	0.706843	0.83813
TCONS_00227793	XLOC_018779	-	lincRNA	2059	chr4	109589943	109593595	+	2	1.53718	1.44524	1.1256	0.654203	2.38149	2.64868	1.35055	1.90392	1.95695
TCONS_00218470	XLOC_019338	-	lincRNA	1015	chr4	110302626	110317414	-	2	2.12567	0.87079	0.77495	0.471841	0.681688	0.259391	0.57796	1.29143	0.426755
TCONS_00231187	XLOC_018799	-	anti-sense_lncRNA	1102	chr4	114794160	115040277	+	3	0.33902	0.23589	0.07772	0.0710393	0	0	0.130365	0	1.09179

TCONS_00224080	XLOC_019351	-	lincRNA	286	chr4	118735026	118738832	-	3	1.13868	0	0	1.48693	0.741452	1.64605	0.749615	0	0.695085
TCONS_00219113	XLOC_018807	-	lincRNA	689	chr4	119512890	119517594	+	2	1.24821	0.43736	0.72279	1.31269	1.01465	1.44414	0.851376	0.500562	1.19086
TCONS_00217814	XLOC_018815	-	lincRNA	624	chr4	120298794	120324263	+	2	1.67837	0.6737	0	0	0.293075	0.16669	0.28163	0.193007	0.137584
TCONS_00221570	XLOC_019362	-	lincRNA	618	chr4	120362195	120366943	-	4	1.94553	1.36699	2.37494	1.383	1.04069	0.338195	0.428689	1.37088	0.9771
TCONS_00218519	XLOC_019380	-	intronic_lncRNA	2810	chr4	123802213	123807074	-	2	1.79931	0.89319	1.04082	0.539379	0.753222	0.781256	1.25041	0.800164	0.46422
TCONS_00230036	XLOC_018827	-	lincRNA	257	chr4	124443193	124448380	+	3	1.6228	0	1.27519	1.0731	5.40825	0	2.22282	1.4782	3.03864
TCONS_00221588	XLOC_019383	-	lincRNA	2463	chr4	125420093	125477693	-	5	1.12769	1.4052	0.76989	0.593874	0.407348	0.497207	0.566961	0.710281	0.484569
TCONS_00230284	XLOC_019383	-	lincRNA	449	chr4	125458903	125464852	-	3	0.40681	1.15748	1.73339	0	0.503762	0.570087	0.978815	2.33667	0.472885
TCONS_00219182	XLOC_018838	-	anti-sense_lncRNA	4112	chr4	129209352	129214471	+	2	1.90606	1.22582	3.58312	1.34294	1.65934	1.89889	2.43009	1.83449	1.19606
TCONS_00225530	XLOC_018838	-	anti-sense_lncRNA	5092	chr4	129209886	129225824	+	3	3.38171	2.03515	1.8415	0.560711	1.73455	1.22569	1.50523	1.48429	1.12927
ENST00000514265.1	ENSG00000248187.1	RP11-184M15.1	lincRNA	1623	chr4	129489127	129491686	-	2	0.35847	0.04973	0.24544	1.30487	0.302758	1.38468	0.57559	0.283081	0.0406173
TCONS_00220620	XLOC_019396	-	lincRNA	983	chr4	134869395	134932197	-	5	1.56163	1.7226	1.34497	0.491171	0.473167	0.360036	0.451462	0.517248	0.518373
TCONS_00220622	XLOC_019406	-	lincRNA	396	chr4	139823303	139831550	-	2	1.03094	1.84817	0	0	0	0	0.944681	0.428228	0.302102
ENST00000608345.1	ENSG00000273247.1	RP11-83A24.2	lincRNA	433	chr4	140334956	140337300	-	2	4.34466	2.16779	0.92843	0.276636	0.80876	0.609641	0.524753	1.43054	2.02445
TCONS_00222008	XLOC_018848	-	intronic_lncRNA	569	chr4	141187985	141190761	+	2	1.01303	0.58171	1.1564	0.92471	0.636071	0.191928	0	0	0.304134
TCONS_00223803	XLOC_018848	-	intronic_lncRNA	369	chr4	141188279	141190761	+	3	0.19144	0	0	1.41106	0.0861991	0.422168	2.94828	1.00007	0.0281388
ENST00000511213.1	ENSG00000248810.1	RP11-362F19.1	lincRNA	719	chr4	142244625	142253772	-	3	0.58818	0.68635	0.68029	1.73116	2.14953	1.90434	2.74627	1.09923	1.90618
TCONS_00220200	XLOC_018854	-	intronic_lncRNA	1990	chr4	143486927	143506177	+	2	0.51312	0.75056	1.48099	0.500801	0.51533	0.314392	0.587286	0.898951	1.35508
ENST00000507486.1	ENSG00000250326.1	RP11-284M14.1	lincRNA	759	chr4	144098813	144105982	-	2	2.73126	1.5282	3.15418	1.03298	1.99414	1.76744	2.75754	2.4753	3.22477
TCONS_00220205	XLOC_018858	-	lincRNA	360	chr4	144481196	144493819	+	2	0	0.45506	4.12057	1.6156	3.17159	0.445642	1.17189	2.64766	0
TCONS_00217871	XLOC_018859	-	anti-sense_lncRNA	1954	chr4	144779021	144834187	+	4	1.15458	0.73022	1.08228	0.646495	0.614146	0.62728	0.439096	0.925207	0.551221
TCONS_00223807	XLOC_018859	-	lincRNA	1074	chr4	144779948	144787771	+	3	0.9511	0.55904	0.94609	0.754131	0.598967	0.351437	0.391312	0.354701	0.149814
TCONS_00227454	XLOC_019441	-	intronic_lncRNA	2552	chr4	146829497	146832807	-	2	2.47393	2.10295	2.81447	1.19719	2.40363	1.7341	2.18159	2.66486	2.18373
TCONS_00219771	XLOC_019447	-	lincRNA	819	chr4	149742020	149756510	-	3	2.13844	4.25117	2.84405	0.31085	0.399792	0.455828	1.81592	0.525106	1.97086
TCONS_00226860	XLOC_019452	-	lincRNA	1638	chr4	152326594	152330111	-	2	1.98654	1.37787	1.7486	0.712407	1.15555	1.12553	1.26111	0.616239	1.40672
ENST00000515789.1	ENSG00000248571.1	RP11-768B22.2	intronic_lncRNA	2371	chr4	153587520	153591259	+	2	0.23509	0.35809	0.28895	2.85948	0.0566207	0.90703	0.214953	0.444306	1.11666
TCONS_00220241	XLOC_018891	-	lincRNA	1171	chr4	154710717	154805614	+	2	0.41998	1.67924	0.36073	0.791777	0.190508	0.580281	1.27034	1.16517	0.775231
TCONS_00227909	XLOC_018891	-	lincRNA	2907	chr4	154924938	154949807	+	2	0.53639	0.63195	0.42495	2.43845	2.13059	1.94022	0.979716	1.00626	0.734627
TCONS_00223832	XLOC_018891	-	anti-sense_lncRNA	1680	chr4	154925336	155193226	+	5	1.41984	1.04843	1.37192	6.83767	5.43838	4.56076	5.4903	2.80937	1.51556
TCONS_00224645	XLOC_018891	-	anti-sense_lncRNA	1849	chr4	154925336	155193424	+	6	0	0.72254	0.48653	14.0171	2.61147	4.88128	2.96305	0.309045	2.73108
TCONS_00224644	XLOC_018891	-	anti-sense_lncRNA	4588	chr4	154925336	155193424	+	5	0	0.40619	0.59696	4.21479	1.44596	1.58641	1.66897	0	1.423
TCONS_00226358	XLOC_018891	-	anti-sense_lncRNA	1791	chr4	154926175	155160454	+	5	1.30268	0.83883	0.72913	8.5011	5.86617	5.74744	3.37518	2.54566	2.46231
TCONS_00223831	XLOC_018891	-	lincRNA	5712	chr4	154926192	155133143	+	5	2.23208	2.33506	2.46202	14.3907	11.9327	11.3959	10.3426	5.17446	3.89948
TCONS_00227468	XLOC_019466	-	lincRNA	1680	chr4	156441933	156443858	-	2	1.79237	1.05178	0.70778	0.259562	1.12263	0.76072	1.30406	1.08844	1.09332
TCONS_00221144	XLOC_018908	-	anti-sense_lncRNA	1595	chr4	159094020	159123393	+	6	2.42076	2.11659	1.9639	1.25159	0.898892	0.830476	0.555031	0.805399	0.595241
ENST00000508752.1	ENSG00000248429.1	RP11-597D13.9	lincRNA	2724	chr4	159097459	159122065	+	4	0.72273	0.50662	0.4287	0.266512	0.15692	0.307329	0.199555	0.297924	0.371158
TCONS_00230616	XLOC_018908	-	lincRNA	4691	chr4	159117219	159122126	+	2	2.86602	1.49588	1.64567	1.27089	0.672189	1.57293	1.4393	1.1376	1.84061
TCONS_00230089	XLOC_018917	-	lincRNA	1744	chr4	160618604	160768302	+	3	0.33042	0.36648	0.22609	1.02E-06	0.372912	1.39713	0.436093	0.878677	1.07436
TCONS_00228750	XLOC_018917	-	lincRNA	1013	chr4	160618982	160650989	+	2	0	0.00014	0	0.0788321	0.732117	1.9365	1.55594	1.30976	3.65717
TCONS_00222439	XLOC_019482	-	intronic_lncRNA	3352	chr4	164472242	164503920	-	2	1.00421	1.59071	4.10799	3.83649	3.62395	5.04065	2.43867	2.85244	6.1664
TCONS_00227473	XLOC_019488	-	lincRNA	1561	chr4	169245300	169247645	-	2	0.82451	0.62406	0.46204	0.329336	0.226149	0.465391	0.172013	0.414492	0.934455
TCONS_00230998	XLOC_019497	-	intronic_lncRNA	1796	chr4	173910283	173917131	-	2	1.91801	0.26593	0.61232	0.160449	0.231277	0.176335	1.13532	1.91684	1.30317
TCONS_00222455	XLOC_019498	-	lincRNA	4679	chr4	174083509	174088532	-	2	2.0065	0.99278	1.56848	0.885506	2.39815	1.86733	0.779178	1.41463	1.42857
TCONS_00226404	XLOC_018937	-	intronic_lncRNA	634	chr4	174144783	174145547	+	2	0.70281	0.1645	0.32651	1.18382	2.14675	1.13978	0.549913	1.13078	1.07499
TCONS_00224163	XLOC_019500	-	intronic_lncRNA	3455	chr4	174201606	174211598	-	2	0.34507	0.21694	0.21385	1.49387	3.43358	2.00855	0.375643	0.567172	0.318921
ENST00000608794.1	ENSG00000272870.1	RP11-798M19.6	anti-sense_lncRNA	587	chr4	174285145	174290966	-	2	6.03899	4.43414	10.6454	2.98866	1.4468	2.19281	6.18835	3.1787	3.01858
TCONS_00217976	XLOC_018953	-	lincRNA	2135	chr4	181069927	181159035	+	2	4.11413	0.54802	1.15327	0.0330781	0.0635466	0.0363501	0.452501	0.914389	0.35807
TCONS_00222461	XLOC_019515	-	lincRNA	495	chr4	183006236	183009848	-	2	0	0.24344	0.48503	0	0	1.2011	0.819916	0.280124	0
TCONS_00220701	XLOC_019523	-	lincRNA	804	chr4	184683133	184687017	-	2	1.68566	1.88441	1.74943	0.424792	0.512268	0.233598	1.37195	0.538351	0.0962027
TCONS_00231468	XLOC_019530	-	lincRNA	3627	chr4	185280799	185284563	-	2	0.53633	0.5769	0.85298	0.634749	0.573356	2.19484	0.424671	1.56905	1.90147
TCONS_00223557	XLOC_019530	-	lincRNA	5177	chr4	185294558	185305323	-	2	2.19571	2.15533	1.92826	2.17211	2.00995	4.57517	1.53057	3.62512	4.03011
ENST00000605270.1	ENSG00000271538.1	RP11-326I11.4	lincRNA	1059	chr4	185427614	185458708	-	3	0.4746	0.41295	0.08165	0.820635	0.431025	0.410106	1.23285	0.753573	1.01188
TCONS_00227226	XLOC_018976	-	intronic_lncRNA	1004	chr4	187124473	187125540	+	2	8.36522	2.82492	3.49186	3.58734	4.30006	3.68132	1.83121	1.81289	2.95633
TCONS_00222476	XLOC_019552	-	lincRNA	1749	chr4	189354482	189361065	-	2	0.85632	0.41104	1.84794	0.0413309	0.873842	0.272541	1.17003	1.50751	0.932539
TCONS_00222477	XLOC_019554	-	lincRNA	270	chr4	190018201	190045617	-	2	1.36721	0	0	0	0	0	0.918523	0	0
TCONS_00225610	XLOC_018987	-	lincRNA	329	chr4	190802370	190802784	+	2	3.10631	1.70072	0	0	1.97733	0	1.96499	0	0
TCONS_00237892	XLOC_019567	-	anti-sense_lncRNA	2174	chr5	478745	481063	+	2	2.01647	2.2919	4.02699	2.85331	4.42241	3.84815	1.53749	1.58878	1.57939
TCONS_00233173	XLOC_020209	-	lincRNA	2058	chr5	571075	580406	-	3	0.85147	0.65359	2.38637	0.673455	2.92613	1.58857	0.222507	0.106844	0.15124
TCONS_00234506	XLOC_020209	-	lincRNA	1303	chr5	571907	580397	-	2	3.09709	1.20908	5.35657	2.40715	7.62997	6.19442	1.96312	0.258488	0.0070168
TCONS_00242745	XLOC_019570	-	intronic_lncRNA	416	chr5	628589	629138	+	2	0.93657	0	0.3348	0.298576	0.874011	1.31629	0.284166	0.386961	1.64078
TCONS_00232454	XLOC_019569	-	anti-sense_lncRNA	2333	chr5	666186	669282	+	2	3.30193	2.38569	3.43144	1.44023	4.55293	2.17613	1.75044	1.80902	1.48841

TCONS_00244566	XLOC_020229	-	lincRNA	296	chr5	1546901	1548539	-	2	2.05773	0	0	0	0	0	0	0	1.24848
TCONS_00235118	XLOC_019578	-	lincRNA	2185	chr5	1931467	1936070	+	2	1.02836	1.31764	0.52692	0.128976	0.991062	0.283466	0.26462	0.32409	0.145427
TCONS_00237088	XLOC_019581	-	lincRNA	290	chr5	2267021	2278422	+	2	0	0	2.48627	0	0	0	2.14733	0	0
TCONS_00239562	XLOC_020238	-	lincRNA	1183	chr5	5390987	5422278	-	3	2.18753	0.54024	1.27539	0.749095	0.581056	1.1208	1.11936	1.18872	0.857023
TCONS_00233192	XLOC_020238	-	lincRNA	1895	chr5	5419891	5422347	-	2	1.43939	1.14778	1.40394	0.434557	1.26048	0.761892	1.07764	1.63992	0.935434
TCONS_00233196	XLOC_020242	-	lincRNA	4380	chr5	6356265	6363752	-	2	2.14995	0.40529	0.68235	0.27548	0.925173	0.723045	0.306194	0.652477	1.06197
TCONS_00235130	XLOC_019601	-	lincRNA	435	chr5	6777117	6777726	+	2	0.86161	2.14894	0.92023	0	0.534478	0.3022	0	0.354473	0
ENST00000416930.2	ENSG00000246016.2	RP11-1C1.7	lincRNA	483	chr5	10479483	10482421	+	2	7.16638	2.53955	3.54364	1.13727	1.32614	2.25448	3.63879	2.33916	3.1123
TCONS_00235609	XLOC_020272	-	lincRNA	230	chr5	17145199	17147509	-	2	0	0	2.068	0	1.73801	0	1.81885	0	0
TCONS_00241362	XLOC_020273	-	lincRNA	215	chr5	17827044	17828972	-	2	3.47207	0	14.5833	0	2.43715	0	20.6339	3.38666	0
TCONS_00233235	XLOC_020281	-	lincRNA	205	chr5	30788879	30790766	-	2	8.90118	0	0	0	3.17569	0	0	0	2.95998
TCONS_00239272	XLOC_019639	-	lincRNA	475	chr5	34588923	34590631	+	2	1.10598	1.30743	1.82501	0.468231	2.27584	1.03141	1.98495	1.80714	1.70915
ENST00000606401.1	ENSG00000272323.1	CTD-2517O10.6	anti-sense_lncRNA	1565	chr5	34837654	34839383	-	2	5.1568	3.37038	4.86272	1.31348	2.6156	1.64986	4.67349	2.65673	3.09157
ENST00000508745.1	ENSG00000250155.1	CTD-2353F22.1	lincRNA	589	chr5	36702732	36725297	-	3	0.26122	0.73517	1.09545	2.80804	1.75909	5.45366	0.923328	0	6.00568
TCONS_00233277	XLOC_020309	-	anti-sense_lncRNA	1873	chr5	37065561	37069008	-	2	2.92775	1.47962	1.58511	0.803546	1.21328	1.3877	0.977908	0.962171	1.34658
ENST00000514532.2	ENSG00000248587.2	GDNF-AS1	lincRNA	2913	chr5	37870009	37874996	+	3	0.67175	0.44262	0.61954	0.585703	2.03376	3.23252	0.380705	0.444047	0.399723
ENST00000510986.2	ENSG00000248587.2	GDNF-AS1	lincRNA	1941	chr5	37873527	37875901	+	2	0.41906	0.52804	0.47555	0.373823	1.34873	2.63733	0.143403	0.554633	0.172593
TCONS_00241060	XLOC_019666	-	anti-sense_lncRNA	362	chr5	41015834	41020228	+	2	0	0	0	0	1.56569	0.880181	0.3855	0	0.367343
ENST00000510349.1	ENSG00000250360.1	CTD-2089N3.1	lincRNA	569	chr5	50261521	50262842	-	2	0.2753	0.58171	0.57813	0	0	0	0	0	0
TCONS_00239615	XLOC_020358	-	lincRNA	276	chr5	53961958	53999223	-	2	0	0	0	1.66791	0.834464	0	0.847987	0	0.782038
TCONS_00235207	XLOC_019695	-	lincRNA	3243	chr5	54001807	54033179	+	2	0.46975	1.02115	1.83033	1.45082	0.222009	0.277238	0.784696	0.63319	2.25558
TCONS_00241761	XLOC_019701	-	anti-sense_lncRNA	1294	chr5	54575053	54591352	+	4	2.60997	2.00758	3.39034	0.64405	1.91508	1.28688	2.03738	1.5555	2.38025
TCONS_00238041	XLOC_019701	-	anti-sense_lncRNA	1158	chr5	54579184	54591352	+	2	0	0	0	0	0	0	0	0.246305	0
TCONS_00242817	XLOC_019712	-	anti-sense_lncRNA	872	chr5	56749006	56829260	+	4	0.90938	0.63456	0.7327	0.0954216	0.551989	1.67895	1.75799	0.724665	0.518322
TCONS_00244333	XLOC_019719	-	lincRNA	1378	chr5	60475912	60478760	+	2	2.33762	0.90182	2.01868	0.978633	1.46412	1.0757	1.74134	1.30122	2.11151
TCONS_00234687	XLOC_020386	-	lincRNA	870	chr5	60546334	60600673	-	3	7.6007	4.13708	4.82943	0.861365	0.830476	1.26299	2.20416	1.09029	2.7727
ENST00000515184.1	ENSG00000250978.1	RP11-357D18.1	lincRNA	685	chr5	66760863	66771420	-	4	0.41949	0	0.29154	0.264712	1.79045	2.62101	1.5945	0.336514	0.12008
ENST00000515199.1	ENSG00000248359.1	CTC-537E7.2	lincRNA	340	chr5	67827517	67829357	-	2	0.71725	0.52161	6.31665	0.461767	1.36397	1.52938	3.15219	1.827	0
TCONS_00237635	XLOC_020407	-	lincRNA	2289	chr5	68324252	68339819	-	2	1.22152	0.84586	1.93555	0.459561	0.853314	1.58235	1.75959	0.808193	1.21588
ENST00000502659.2	ENSG00000250387.2	RP11-136K7.2	lincRNA	2767	chr5	70647682	70742169	-	2	0.19885	0.02752	0	0	0.0478667	0.246524	0.0227046	0.0312929	0.179814
TCONS_00241114	XLOC_019754	-	lincRNA	1147	chr5	70878694	70879921	+	2	0.75355	0.22461	0.36996	0.135314	1.30244	0.297514	0.496361	0.682851	0.244614
TCONS_00247059	XLOC_019758	-	anti-sense_lncRNA	2034	chr5	71852249	71884834	+	5	0.55871	0.93951	1.96732	0.0381165	0.0234466	0.490346	1.35557	0.317776	1.0134
TCONS_00238141	XLOC_019758	-	anti-sense_lncRNA	2650	chr5	71852449	71885574	+	5	1.17428	1.02723	1.12583	0.311029	1.04832	0.231128	1.21333	0.62706	0.1869
TCONS_00237217	XLOC_019758	-	anti-sense_lncRNA	3543	chr5	71865373	71886218	+	4	1.21498	0.14806	0.8887	0	0.137758	0.23473	0.216496	0.279309	0.549955
TCONS_00246085	XLOC_020426	-	lincRNA	519	chr5	71986071	71989435	-	2	1.27264	0.89925	1.79009	0.201638	0	0	0.377931	0.775287	0.918327
TCONS_00241429	XLOC_020427	-	lincRNA	572	chr5	72542146	72570744	-	2	1.36546	0.19232	0.38224	0.345531	1.67338	1.71139	0.161154	1.10338	0.471319
TCONS_00234750	XLOC_020452	-	anti-sense_lncRNA	364	chr5	78419096	78531806	-	2	4.91507	2.21848	2.67705	0.393999	1.9326	1.73869	1.522	0.51601	0.725492
TCONS_00235721	XLOC_020454	-	lincRNA	1317	chr5	78839396	78843753	-	2	1.18687	0.76105	0.18792	0.22937	0.551602	0.189096	2.36247	1.08385	0.569798
TCONS_00235723	XLOC_020463	-	lincRNA	906	chr5	80243593	80244832	-	2	0.86572	1.10691	0.49783	1.72546	0.262595	0.499311	1.00311	0.804182	0.164388
TCONS_00235304	XLOC_019805	-	lincRNA	510	chr5	81153436	81188596	+	3	0.65466	2.08308	1.15222	0	0.604266	0.457003	1.94669	0.798479	0.189093
TCONS_00234151	XLOC_019814	-	lincRNA	4203	chr5	83785113	83834229	+	2	2.16843	0.77593	1.94658	1.08674	0.843455	0.825272	0.581407	0.741518	0.374465
TCONS_00235315	XLOC_019821	-	lincRNA	10327	chr5	87571244	87586031	+	3	13.2042	6.21271	11.5332	3.56187	9.64823	9.72012	8.17236	8.51186	8.94762
TCONS_00233445	XLOC_020489	-	lincRNA	531	chr5	90538744	90541091	-	2	1.8402	0	0	0.388573	0.188424	0.641677	0.545582	0.497586	0.35379
TCONS_00232773	XLOC_019828	-	lincRNA	688	chr5	90890829	90892319	+	2	2.50152	0.29218	1.73832	0.526158	0.635468	0.434135	0.243761	1.00322	0.715999
TCONS_00242886	XLOC_019828	-	lincRNA	2288	chr5	90986371	91019842	+	5	1.66805	0.64316	2.85922	0.441776	1.25736	1.35774	2.79645	0.616062	0.584106
TCONS_00239369	XLOC_019828	-	lincRNA	717	chr5	90986650	91017705	+	4	2.21647	0.27561	1.10192	0.506196	0.274429	0.165601	1.26187	1.26121	0.998675
TCONS_00232774	XLOC_019828	-	lincRNA	391	chr5	91017155	91018390	+	2	0.85443	0	0.87366	0.172216	0	0.178211	1.9225	0	0.0002308
TCONS_00242887	XLOC_019830	-	lincRNA	279	chr5	91090811	91124334	+	3	0	0.91874	1.88482	0.804782	0	0.891369	1.63236	0	2.26193
TCONS_00233452	XLOC_020497	-	intronic_lncRNA	13060	chr5	93639080	93653662	-	2	9.07189	3.87727	6.86576	2.18813	3.30009	2.79717	4.21706	4.06706	4.03994
TCONS_00237296	XLOC_019837	-	intronic_lncRNA	288	chr5	94492213	94510377	+	2	0	1.65822	1.69507	2.18254	0.725173	1.61055	0	0	1.35961
TCONS_00237687	XLOC_020501	-	intronic_lncRNA	571	chr5	95006327	95010696	-	2	0.82147	0.77137	2.10812	1.0394	0.503387	1.14402	0.484809	0.663858	0.787682
TCONS_00240671	XLOC_020503	-	lincRNA	1658	chr5	95168161	95170184	-	2	0.2799	0.48532	0.62272	2.547	0.675342	0.772219	0.441302	0.497235	0.55494
TCONS_00235334	XLOC_019844	-	lincRNA	1987	chr5	95411891	95716858	+	4	1.99879	0.94967	2.14716	0.895512	1.78948	2.59809	2.12434	2.56634	1.35736
TCONS_00244410	XLOC_019854	-	lincRNA	343	chr5	98307681	98323260	+	3	0.70258	0	0.51486	0	1.33466	0	3.08184	0	0.834928
TCONS_00232795	XLOC_019856	-	lincRNA	359	chr5	98382341	98394616	+	2	3.80003	0.45799	0.46085	0	0.39901	0.44847	0	0.53302	0.748914
TCONS_00237301	XLOC_019859	-	lincRNA	4643	chr5	100239103	100278041	+	2	0.85047	0.82608	2.09785	1.10866	1.2986	1.01242	1.283	2.5273	1.24554
TCONS_00240695	XLOC_020534	-	intronic_lncRNA	6294	chr5	107631660	107640091	-	2	4.99466	2.99881	4.74575	3.69465	3.48375	3.90918	3.71025	4.5118	3.91485
TCONS_00235344	XLOC_019865	-	lincRNA	237	chr5	107806114	107821517	+	2	2.22423	1.71126	0	0	0	0	0	2.08594	0
TCONS_00236817	XLOC_020536	-	lincRNA	2164	chr5	108656283	108659204	-	2	4.05345	2.69953	1.95278	0.847318	1.90947	2.29201	2.82326	1.88354	3.58647
TCONS_00241483	XLOC_020539	-	lincRNA	636	chr5	110350270	110402571	-	3	0.9328	0.16373	0.32498	0.147296	0	0	0.273659	1.12548	0.133749
TCONS_00237711	XLOC_020542	-	lincRNA	356	chr5	111033205	111034450	-	2	0.64528	0.93401	1.41036	1.24291	1.22067	0	0.401303	1.08753	0

ENST00000504004.1	ENSG00000251076.1	RP11-526F3.1	anti-sense_lncRNA	711	chr5	111563980	111593006	+	4	4.38056	6.13541	5.9437	1.75836	3.15403	0.82899	4.53423	4.3075	1.13903
TCONS_00232836	XLOC_019891	-	lincRNA	708	chr5	115952819	115964212	+	2	1.6023	0.98192	1.25151	0.758049	0.488134	0.55594	0.935726	0.320994	0.229167
TCONS_00239402	XLOC_019892	-	lincRNA	287	chr5	116142598	116143833	+	2	2.25346	0	0	0.7354	1.46636	0	2.22267	0	0
TCONS_00232837	XLOC_019894	-	lincRNA	7090	chr5	117260727	117338292	+	2	3.97306	0.94205	3.01643	3.27697	2.1994	1.6828	1.79646	2.36134	1.36314
TCONS_00243165	XLOC_020559	-	intronic_lncRNA	5725	chr5	121650289	121659331	-	4	0.65501	0.56653	0.58072	0.0562637	0.293291	0.0294473	0.30137	0.440092	0.458016
TCONS_00233526	XLOC_020559	-	intronic_lncRNA	4976	chr5	121652494	121659117	-	3	20.6262	5.6692	18.0403	1.38145	2.19221	2.95318	7.09121	5.51873	5.62513
TCONS_00234886	XLOC_020563	-	lincRNA	1738	chr5	123467515	123473764	-	3	1.43036	0.58246	1.92006	0.166508	0.0415286	0.430454	0.553095	0.375961	0.209555
TCONS_00233531	XLOC_020563	-	lincRNA	1364	chr5	123469212	123473201	-	3	1.76527	1.17665	1.12174	0.16507	0.333674	0.256627	0.786155	0.264735	0.517964
TCONS_00244720	XLOC_020563	-	lincRNA	642	chr5	123470735	123739317	-	4	1.55792	0	1.53109	0	0.732528	0.375333	1.80394	0.249176	0
TCONS_00233532	XLOC_020563	-	lincRNA	695	chr5	123721410	123739050	-	2	1.06875	0.30322	0.75565	0.16828	0	0	0.51584	0.642817	1.21992
TCONS_00235789	XLOC_020563	-	lincRNA	302	chr5	123733233	123739072	-	2	3.22709	2.79477	4.52085	2.3376	2.15798	0	3.88421	0.636072	0.369654
TCONS_00244431	XLOC_019911	-	lincRNA	609	chr5	123738249	123745875	+	3	0	8.30E-05	0.08451	0	0	0.0786437	1.37323	0.0935438	0
TCONS_00237329	XLOC_019911	-	lincRNA	942	chr5	123738913	123745875	+	3	0	0.38273	1.07033	0.172793	0.501488	0	1.39891	0.977012	0
TCONS_00232862	XLOC_019911	-	lincRNA	607	chr5	123743736	123745713	+	2	2.24755	0.00011	0.90785	0	0.454606	0.0946684	0	0.118545	0.286821
TCONS_00245230	XLOC_019931	-	lincRNA	1702	chr5	130358431	130446503	+	4	5.63812	1.08342	3.71922	0.0852517	1.47484	1.17118	2.53069	1.55497	1.38503
TCONS_00232891	XLOC_019932	-	lincRNA	1359	chr5	130588730	130592900	+	3	1.40793	1.40557	4.16441	1.32627	1.38194	2.18674	1.71961	2.92391	3.6437
TCONS_00243173	XLOC_020579	-	lincRNA	935	chr5	130752292	130753453	-	2	1.3861	1.35279	1.14709	0.436108	1.26077	1.53453	0.882634	0.441169	1.34174
TCONS_00241516	XLOC_020602	-	lincRNA	384	chr5	133441188	133443077	-	2	0	0.39435	0	0	2.0606	0.386971	0	0	0
TCONS_00235399	XLOC_019947	-	lincRNA	773	chr5	133747662	133756145	+	2	1.24356	0.62104	0.49215	0.111962	1.08052	0.246294	0.723886	0.993927	0.202916
TCONS_00232919	XLOC_019956	-	lincRNA	1136	chr5	134540798	134551434	+	2	3.04954	1.06059	1.12304	0.136907	0.922486	0.30102	0.313919	0.431848	1.17565
TCONS_00233597	XLOC_020614	-	intronic_lncRNA	845	chr5	137021898	137024066	-	2	1.26309	0.55113	0.54557	0.298283	1.53413	0.328034	0.366595	0.503629	0.180069
TCONS_00239441	XLOC_019960	-	anti-sense_lncRNA	464	chr5	137031488	137039604	+	3	0	0	0	3.90299	2.37268	0.80609	0.230163	0	0
TCONS_00233598	XLOC_020613	-	lincRNA	2069	chr5	137148503	137194975	-	3	4.93106	1.47051	2.55356	0.93343	2.48083	2.95944	1.04671	1.4257	1.67761
TCONS_00245649	XLOC_020613	-	anti-sense_lncRNA	2500	chr5	137149261	137225060	-	4	1.1492	1.13426	1.47144	0.240014	1.54942	0.876334	1.38164	1.14797	1.30745
ENST00000514616.1	ENSG00000250159.2	RP11-381K20.2	anti-sense_lncRNA	827	chr5	137150022	137225006	-	5	0.4554	1.2712	1.23616	1.2396	1.34194	1.31668	0	0.520714	0.333154
ENST00000520838.1	ENSG00000253404.1	AC034243.1	lincRNA	587	chr5	138080123	138088998	-	2	0	0.92378	0.91771	0.166036	0.160756	0.365469	0.464126	0.211913	0.150929
ENST00000606674.1	ENSG00000272070.1	AC005618.6	lincRNA	544	chr5	140705807	140707482	+	2	1.77109	0.62465	1.0354	0.560713	0.724739	0.822995	1.39794	1.4348	0.340204
TCONS_00244471	XLOC_020004	-	intronic_lncRNA	552	chr5	140896578	140897457	+	2	0.57703	0.61024	0	0.182634	0.531	0.201038	1.36505	0	0
TCONS_00237394	XLOC_020011	-	lincRNA	223	chr5	141783824	141845366	+	3	0	0	2.40848	0	4.03775	0	0	0	0
TCONS_00244487	XLOC_020032	-	lincRNA	4927	chr5	148213914	148245383	+	2	3.21921	2.583	2.83966	3.19373	3.4667	3.58322	2.37452	4.90301	5.10928
TCONS_00234401	XLOC_020032	-	lincRNA	2416	chr5	148257831	148261443	+	2	2.2571	1.24373	2.23305	0.750881	1.44217	1.39635	1.18442	2.32131	3.20365
ENST00000499521.2	ENSG00000230551.3	CTB-89H12.4	intronic_lncRNA	8636	chr5	148873877	148884233	-	2	23.8204	14.7907	22.8799	23.1185	23.3791	32.6431	19.287	19.9492	23.4806
TCONS_00235875	XLOC_020687	-	intronic_lncRNA	491	chr5	150002059	150002922	-	2	0.34854	1.72792	0	0.221163	0	0	0.208084	1.13639	0
TCONS_00233025	XLOC_020061	-	lincRNA	486	chr5	151326116	151327356	+	2	2.12754	0.75372	0.50076	0.450107	0.218659	0.743541	1.27023	0.578455	0.205268
TCONS_00237428	XLOC_020067	-	lincRNA	612	chr5	153476366	153514757	+	3	0.77575	0.21065	1.72132	0.413171	0.691387	0.540574	0.645723	1.39124	0.881327
TCONS_00235487	XLOC_020067	-	lincRNA	338	chr5	153476594	153501377	+	2	0.6224	0.9449	0.00095	0.163974	1.5767	1.47373	2.0796	0.0002205	0.769763
TCONS_00237440	XLOC_020076	-	lincRNA	2373	chr5	157084502	157087391	+	2	0.70461	0.71551	0.96229	1.5315	0.87681	0.550189	1.3959	0.850799	1.38126
TCONS_00237441	XLOC_020080	-	lincRNA	437	chr5	158527585	158539058	+	2	0	1.07413	1.68418	3.419	1.397	0.815709	2.49297	0	0.994752
ENST00000523301.1	ENSG00000245812.2	RP11-175K6.1	lincRNA	568	chr5	158527630	158534225	+	2	0.82813	0.86927	0.47559	0.598549	0.799726	1.39901	1.19404	1.33882	0
TCONS_00233046	XLOC_020082	-	lincRNA	504	chr5	158737882	158738578	+	2	1.00124	1.18051	1.17562	0.423295	0.410943	0.233049	2.38399	2.44418	0.964467
ENST00000517927.1	ENSG00000253522.2	MIR146A	lincRNA	2301	chr5	159895275	159914433	+	2	1.70037	1.64839	1.19449	2.13236	1.69685	0.836863	1.52732	5.01266	6.48418
TCONS_00239809	XLOC_020740	-	lincRNA	366	chr5	161961981	161966112	-	2	0	0	0	1.16768	0	0	0	0.5095	0.358261
TCONS_00233756	XLOC_020742	-	lincRNA	378	chr5	162050351	162051221	-	2	1.13366	0.40798	0	2.17697	1.42131	2.00078	1.04629	0.473599	0.333491
TCONS_00241593	XLOC_020747	-	lincRNA	2844	chr5	164757250	164788631	-	4	0.38613	0.10687	0.42149	0.0726052	2.04033	0.797792	2.02552	1.45816	1.91117
TCONS_00241592	XLOC_020747	-	lincRNA	3066	chr5	164758039	164763511	-	2	0.39187	0.07393	0.24297	0.0446517	1.33275	0.539689	1.60777	0.952599	1.3569
TCONS_00237464	XLOC_020121	-	lincRNA	292	chr5	171010170	171010931	+	2	0	0.79415	3.24289	0.697385	0	0.771693	0	1.87774	0
TCONS_00235047	XLOC_020763	-	lincRNA	1254	chr5	171268203	171269548	-	2	1.06418	0.80677	0.59775	0.911643	0.701701	0.734899	0.334042	0.689525	0.933511
TCONS_00246975	XLOC_020777	-	lincRNA	1222	chr5	173135898	173137210	-	2	0.2993	0.34674	0.06851	1.56707	0.241265	1.92937	0.287188	1.89684	2.03908
TCONS_00241604	XLOC_020777	-	lincRNA	781	chr5	173141775	173146455	-	2	0.35043	0.85738	0.12132	0.993741	0.213099	0.850106	0	2.80005	0.800382
TCONS_00233089	XLOC_020133	-	lincRNA	2857	chr5	173184508	173200562	+	2	3.15077	2.63207	3.3816	0.216749	1.04044	0.582182	1.25013	0.513897	0.759985
TCONS_00235922	XLOC_020779	-	lincRNA	370	chr5	173297393	173300114	-	2	1.18614	2.1383	0	0	0	0	1.46492	0	0
TCONS_00234486	XLOC_020171	-	lincRNA	453	chr5	177384293	177386801	+	2	2.80302	0.2847	1.42087	1.5277	0.743434	0.84148	0	0.328341	0.232624
TCONS_00233155	XLOC_020188	-	lincRNA	1192	chr5	179285965	179287973	+	2	2.98054	2.21514	1.27089	0.516656	1.18086	0.780951	1.4207	1.38461	1.40073
TCONS_00259896	XLOC_021530	-	lincRNA	394	chr6	1552353	1555393	-	2	2.08287	0	2.24642	0.332702	1.95162	1.83407	0.318332	0.43284	1.22119
TCONS_00257760	XLOC_020870	-	intronic_lncRNA	7382	chr6	5466149	5475215	+	2	2.6169	1.4047	2.24523	1.57631	1.93907	2.08398	1.21359	1.50642	2.2062
TCONS_00253534	XLOC_020879	-	lincRNA	202	chr6	7673008	7674672	+	2	0	0	0	0	0	3.72597	3.70254	0	0
TCONS_00255817	XLOC_020883	-	lincRNA	603	chr6	8790296	8792408	+	2	0.50439	0	0.88046	0.47824	0.617129	0.350865	0.593513	1.42311	1.01397
TCONS_00249239	XLOC_021575	-	lincRNA	5196	chr6	9462869	9468495	-	2	5.29464	1.07356	2.39418	0.230469	0.257964	0.267276	0.872939	0.208653	0.219197
TCONS_00259561	XLOC_020886	-	lincRNA	1059	chr6	10141533	10143930	+	3	0.7119	0.24777	0.24495	0.373016	1.29308	0.574149	1.23285	1.78973	1.55155
TCONS_00261538	XLOC_021585	-	lincRNA	587	chr6	10508989	10510614	-	2	0.26257	0.36951	0.73416	0	0.482267	0.913672	1.08296	0.63574	1.0565
TCONS_00255824	XLOC_020897	-	intronic_lncRNA	5093	chr6	11552675	11558087	+	2	1.39847	0.51925	0.66802	2.23556	1.25429	1.6087	0.867591	1.14721	0.789248

TCONS_00257778	XLOC_020900	-	lincRNA	278	chr6	11993141	11995180	+	2	1.24341	0	0	0	0.814231	1.80396	0	3.31598	0.763127
TCONS_00248440	XLOC_020911	-	lincRNA	5134	chr6	14431351	14513019	+	4	2.22042	1.49335	1.48937	0.32589	0.559262	0.57017	1.03365	0.58214	0.502171
TCONS_00262801	XLOC_020911	-	lincRNA	1629	chr6	14431587	14458115	+	2	1.79922	0.67843	1.47337	0.164551	0.274603	0.280006	0.515299	0.588873	0.804791
TCONS_00254495	XLOC_020911	-	lincRNA	1180	chr6	14431749	14509567	+	3	2.61319	1.98796	1.92435	4.41E-05	1.24037	1.05134	1.32329	0.364001	0.328698
TCONS_00254496	XLOC_020911	-	lincRNA	2747	chr6	14510262	14513790	+	2	1.17881	0.89906	0.71842	0.105276	0.167584	0.100939	0.746697	0.199369	0.239028
ENST00000450930.1	ENSG00000229931.1	RP1-151F17.1	lincRNA	635	chr6	16762143	16762849	+	2	1.86987	0.65645	1.14011	1.47636	1.28505	0.649797	0.822919	2.06822	2.68121
TCONS_00254051	XLOC_021614	-	lincRNA	356	chr6	19151892	19193874	-	2	0.64528	0.93401	2.35059	0	0	0.914341	1.20391	0.543763	0.381847
TCONS_00250747	XLOC_021616	-	lincRNA	2589	chr6	19533302	19559835	-	4	1.82451	1.41756	1.19581	0.292265	0.526865	0.234704	0.391573	0.633705	0.960909
TCONS_00249272	XLOC_021616	-	lincRNA	3781	chr6	19533394	19540820	-	3	1.94766	0.21827	0.38865	0.0909249	0.128867	0.353851	0.584409	0.518959	0.63153
TCONS_00258535	XLOC_020926	-	intronic_lncRNA	1991	chr6	20764757	20767067	+	2	2.73509	1.22391	2.06443	1.78702	1.47645	2.00313	0.847825	0.673835	1.70902
TCONS_00254507	XLOC_020925	-	intronic_lncRNA	3423	chr6	20790478	20797973	+	2	2.50278	1.70896	2.0518	1.60797	1.3909	2.05032	1.13816	1.59393	2.09361
TCONS_00259582	XLOC_020927	-	intronic_lncRNA	908	chr6	21082116	21083100	+	2	0.57552	0.70239	2.1842	1.53946	0.785542	1.79242	0.416762	1.48921	1.6392
ENST00000606723.2	ENSG00000272462.2	U91328.19	lincRNA	4130	chr6	25992890	26002003	+	2	1.71498	1.32924	1.52253	1.15577	1.82764	1.64545	0.858748	1.53106	0.748191
ENST00000424968.1	ENSG00000224843.2	LINC00240	lincRNA	507	chr6	26988232	26991703	+	3	2.64406	0.27366	2.17009	0.838268	0.696998	1.54878	1.76983	1.51435	0.219259
ENST00000420081.1	ENSG00000224843.2	LINC00240	lincRNA	339	chr6	26988415	26991703	+	3	0	0.43573	3.54094	0	1.63678	0.661442	1.36128	0.838833	2.94467
TCONS_00256244	XLOC_021648	-	lincRNA	287	chr6	27374722	27375543	-	2	1.12673	0.83824	1.71442	0	0.73318	2.44211	0	0	0
ENST00000444986.1	ENSG00000224157.1	HCG14	lincRNA	413	chr6	28864307	28865099	+	2	0.94962	1.69728	3.39702	0	1.18217	1.66878	4.32585	1.17792	0.554819
TCONS_00252976	XLOC_021662	-	lincRNA	808	chr6	29459054	29462335	-	2	0.83731	1.63792	0.34755	0.316491	0.508866	0.696165	1.26541	0.401066	0.095564
TCONS_00253600	XLOC_020978	-	lincRNA	822	chr6	29465286	29475679	+	3	0.81852	2.97277	0.79244	1.13427	0.397846	0.22681	1.33147	0.522535	0.560362
ENST00000434086.1	ENSG00000214922.5	HLA-F-AS1	lincRNA	553	chr6	29705536	29706221	-	2	1.72621	3.65088	3.42833	2.36748	2.11789	1.20279	1.02079	2.09586	2.15405
TCONS_00250789	XLOC_021670	-	lincRNA	947	chr6	29853803	29855607	-	2	6.13752	0.76051	4.13762	0.514901	2.48072	1.60415	0.947152	1.30198	1.08708
TCONS_00252486	XLOC_021016	-	lincRNA	561	chr6	31789958	31795419	+	2	0.56273	1.58611	0.78835	0.890286	0.862587	0.19599	1.49609	1.36546	1.13377
TCONS_00255903	XLOC_021036	-	lincRNA	231	chr6	32844889	32847416	+	2	0	0	2.0256	1.66945	0	0	1.78092	4.70182	3.18422
ENST00000415875.2	ENSG00000223837.2	BRD2-IT1	intronic_lncRNA	338	chr6	32938009	32938663	+	2	1.45542	1.061	2.1391	0.939655	1.84562	2.58607	1.37421	0	1.30126
TCONS_00255906	XLOC_021041	-	lincRNA	3168	chr6	33090892	33094390	+	2	0.61921	0.66628	0.3988	0.150907	0.186242	0.355313	0.196245	0.568086	0.932848
ENST00000606432.1	ENSG00000272217.1	XXbac-BPG157A1	lincRNA	594	chr6	33213852	33214633	-	2	0.77378	0.54428	0.18021	0.815362	0	0.538399	0	0.416115	0
TCONS_00250853	XLOC_021744	-	intronic_lncRNA	896	chr6	33676758	33678584	-	2	5.12235	3.47089	2.02031	1.38183	4.52879	2.32995	1.78111	2.33112	1.41753
TCONS_00250862	XLOC_021756	-	anti-sense_lncRNA	707	chr6	35198790	35227222	-	3	2.00681	0.84332	0.69668	0.253183	0.611384	1.25335	0.468808	0.964916	1.60736
TCONS_00251535	XLOC_021086	-	lincRNA	785	chr6	37484382	37503661	+	4	0	1.45964	1.20474	0.328975	0	0.482438	0.404986	1.80739	1.78843
TCONS_00261616	XLOC_021774	-	lincRNA	1893	chr6	37783286	37786757	-	2	1.2055	0.71016	0.70071	0.49155	0.799266	0.498649	1.10431	1.14089	0.682368
TCONS_00248709	XLOC_021115	-	lincRNA	1870	chr6	42725878	42749651	+	2	2.93301	2.24461	1.62976	1.30332	1.28913	2.02211	1.01467	2.40977	1.27982
TCONS_00257921	XLOC_021145	-	lincRNA	2378	chr6	45779901	45784279	+	2	0.98419	0.45428	0.35203	0.117537	0.197534	0.129159	0.187476	1.10718	0.821546
TCONS_00258269	XLOC_021827	-	lincRNA	406	chr6	45794378	45799117	-	2	0.9815	0.41534	0	0	1.22338	0	0.424151	0	0
TCONS_00254192	XLOC_021827	-	lincRNA	2082	chr6	45796718	45799049	-	2	3.30797	1.68364	2.14944	0.306095	0.0653416	0.186876	0.54537	1.11122	0.368183
ENST00000444038.2	ENSG00000231769.2	RP1-8B1.4	lincRNA	462	chr6	46064830	46097328	-	3	8.89629	2.74724	11.7857	2.21231	3.82589	4.33227	9.04806	3.79997	4.48933
TCONS_00249568	XLOC_021830	-	lincRNA	1398	chr6	46423582	46459173	-	2	14.4731	5.9113	7.53031	7.53778	7.40346	7.46266	12.1306	6.46395	3.04172
ENST00000415787.1	ENSG00000236466.1	RP11-795J1.1	lincRNA	622	chr6	46459789	46500495	+	3	0	0.27772	4.88E-05	0.0001467	0	0	0.490092	0	4.11E-05
TCONS_00253743	XLOC_021148	-	lincRNA	733	chr6	46497830	46500558	+	2	0.57279	0.58247	1.58906	0.842645	0.697488	0.927039	0.505152	1.37551	0.327425
ENST00000446733.1	ENSG00000226594.1	RP11-550C4.6	lincRNA	197	chr6	47697563	47708477	-	2	5.57228	0	4.86975	0	0	0	0	0	3.75676
TCONS_00257323	XLOC_021840	-	lincRNA	1212	chr6	48036629	48078962	-	2	0.806	1.05055	1.38395	2.27892	2.86303	3.20109	2.55253	1.83593	0.858059
TCONS_00250926	XLOC_021862	-	lincRNA	1689	chr6	52644912	52647214	-	2	1.43895	0.99788	0.60968	0.0429985	0.661228	0.236286	0.981908	0.324545	0.232861
ENST00000448327.1	ENSG00000235899.1	RP11-345L23.1	lincRNA	566	chr6	53493178	53496192	+	3	1.9428	0.39103	0.58299	0.175612	2.55186	0.386588	0.655541	0.224387	0
TCONS_00254228	XLOC_021875	-	lincRNA	852	chr6	53611194	53658505	-	3	0	0.45023	1.43231	0.172438	0.996836	1.62447	0.269365	0.605668	0.307965
TCONS_00250939	XLOC_021875	-	lincRNA	1153	chr6	53611305	53658726	-	2	2.24645	0.73435	1.15639	0.285514	1.64943	0.885556	1.11144	0.774909	0.336768
ENST00000425089.1	ENSG00000224984.1	RP11-524H19.2	lincRNA	586	chr6	54704916	54705653	-	2	1.22369	1.48193	2.21462	0.499394	0.967069	0.820782	1.70658	0.849903	1.05928
TCONS_00249605	XLOC_021876	-	lincRNA	371	chr6	54705431	54711254	-	2	0.79691	0	2.54805	2.66E-05	0.370277	1.05011	0.727353	0.493911	0
TCONS_00261346	XLOC_021181	-	lincRNA	239	chr6	55823147	55848861	+	2	0	0	0	0	0	0	1.51753	4.01614	0
ENST00000422882.1	ENSG00000215190.4	LINC00680	lincRNA	2622	chr6	58272361	58276677	-	2	3.24547	1.86701	2.01367	1.84952	3.06972	1.91619	2.43114	2.22263	1.59631
TCONS_00260042	XLOC_021884	-	intronic_lncRNA	1461	chr6	62678004	62681315	-	2	2.58847	0.67376	1.82944	0.355492	1.90448	1.78622	0.418017	0.51155	0.550301
TCONS_00257939	XLOC_021191	-	intronic_lncRNA	5859	chr6	64463180	64470313	+	2	1.93119	1.01001	1.17949	0.565163	1.43133	1.22941	1.10945	1.60084	2.30153
ENST00000429530.1	ENSG00000236345.1	RP11-59D5__B.2	anti-sense_lncRNA	1454	chr6	64516729	64532535	+	2	2.11485	1.97616	1.89548	0.561753	3.09371	2.24526	3.17615	1.8648	0.876196
TCONS_00249628	XLOC_021895	-	lincRNA	1529	chr6	69339873	69341507	-	2	2.533	1.11851	0.8939	0.626372	0.741175	0.529576	1.45359	1.21298	0.652533
TCONS_00259730	XLOC_021205	-	intronic_lncRNA	5844	chr6	72694571	72701515	+	2	9.37396	1.85036	2.83338	0.793331	4.15319	3.83501	4.0067	2.32953	5.31985
TCONS_00249647	XLOC_021919	-	lincRNA	4636	chr6	77857578	77983649	-	4	2.31231	1.52222	1.79018	0.887613	0.774836	0.825281	1.27309	1.86564	0.877227
TCONS_00260055	XLOC_021919	-	lincRNA	1605	chr6	77860252	78026620	-	5	2.07387	0.83775	1.68218	0.256751	0	1.6485	0.598362	2.68471	0.471964
TCONS_00252136	XLOC_021919	-	lincRNA	1403	chr6	77860389	77983383	-	3	0	2.51353	0	1.05492	7.33E-05	0	2.60795	0	0.0010746
TCONS_00254761	XLOC_021222	-	lincRNA	765	chr6	80131512	80191228	+	3	4.32379	5.03938	3.49458	0.681317	1.86315	1.62368	4.61597	1.15227	2.05818
ENST00000452402.1	ENSG00000233967.2	RP11-250B2.3	lincRNA	682	chr6	81153061	81175644	+	4	2.95264	1.27792	2.24653	0.39955	1.07737	1.19656	3.20491	2.85289	1.29654
ENST00000427048.2	ENSG00000233967.2	RP11-250B2.3	lincRNA	554	chr6	81153107	81172770	+	4	3.7323	2.70223	2.95307	0.363193	2.39839	1.1661	2.04172	4.67909	1.36641
TCONS_00252143	XLOC_021928	-	lincRNA	1756	chr6	82200032	82223754	-	3	0.91805	1.18208	0.62815	0.246868	0.830355	0.40697	0.638722	0.620973	0.482731
TCONS_00248877	XLOC_021235	-	lincRNA	9975	chr6	84683615	84694324	+	2	1.35846	0.41601	2.07878	0.904438	2.59138	1.70446	0.925736	1.42183	1.50893

TCONS_00258744	XLOC_021235	-	lincRNA	3955	chr6	84683883	84694385	+	2	0.1362	0.28855	0.08437	0.319735	0.212924	0.371974	0.266547	0.139251	0.398518
TCONS_00256029	XLOC_021237	-	intronic_lncRNA	922	chr6	84854037	84856267	+	2	0.70549	0.49191	0.38933	0.532777	1.54038	0.195282	0.163418	0.336912	0.401791
ENST00000427501.1	ENSG00000203875.6	SNHG5	lincRNA	680	chr6	86386800	86387711	-	2	14.6224	12.6267	16.9417	15.1127	12.4068	18.3921	14.9997	10.8828	12.8626
TCONS_00252676	XLOC_021244	-	lincRNA	962	chr6	88101671	88108032	+	2	2.00568	0.9318	1.56679	1.34597	1.53996	1.57247	1.39231	1.91405	1.44607
TCONS_00249712	XLOC_021962	-	lincRNA	922	chr6	89841393	89853935	-	3	1.41098	0.68867	0.292	0.177592	0.171153	0.292923	0.65367	0.112304	0.642866
TCONS_00249726	XLOC_021972	-	lincRNA	531	chr6	93298854	93299829	-	2	2.4536	1.73224	0.86173	0	0	0	1.81861	0.74638	0.530685
TCONS_00256423	XLOC_021973	-	lincRNA	381	chr6	93650692	93654660	-	2	0	0	0	0	0	0	1.02788	0.465389	0
TCONS_00258771	XLOC_021261	-	lincRNA	812	chr6	101329315	101579901	+	5	2.49821	0.70712	1.99012	1.30986	3.38116	1.2479	0.859049	2.95691	1.97594
TCONS_00257981	XLOC_021261	-	lincRNA	2339	chr6	101329337	101581512	+	5	0.81048	0.42676	0.54445	0.254383	1.33781	0.4989	0.466796	0.591324	0.328854
TCONS_00263238	XLOC_021996	-	lincRNA	2491	chr6	106126180	106341693	-	4	0.07323	0.15635	0.18458	0.487171	0.136714	0.369376	0.104274	1.18505	0.705665
TCONS_00256437	XLOC_021996	-	lincRNA	800	chr6	106277634	106340929	-	3	0	0.43335	0.36017	1.6643	0.630054	1.70742	0.415695	2.05141	2.89144
TCONS_00260089	XLOC_021996	-	lincRNA	1073	chr6	106312298	106341316	-	3	0.15838	0.18494	0.14976	0.513261	0.197524	0.600838	0.24864	0.748532	0.340663
TCONS_00256052	XLOC_021270	-	lincRNA	360	chr6	107150835	107161887	+	2	1.25891	0	1.83136	0	1.18935	0	1.17189	1.05906	0.372056
TCONS_00253855	XLOC_021286	-	lincRNA	1437	chr6	110736917	110742113	+	2	1.48421	1.20206	2.99561	0.3624	0.696983	0.341427	2.13104	1.56465	2.01034
TCONS_00248952	XLOC_021286	-	lincRNA	847	chr6	110736958	110740102	+	2	1.8888	0.54941	1.95788	0	0.382337	0.109005	0.730881	1.0041	0.718031
TCONS_00258010	XLOC_021301	-	lincRNA	219	chr6	113448006	113458830	+	2	0	0	0	0	0	0	0	3.06988	0
TCONS_00251154	XLOC_022030	-	lincRNA	2127	chr6	113943154	113953894	-	4	2.19794	0.78688	1.88716	0.120698	1.41772	0.393057	1.47612	1.30853	1.33254
TCONS_00249801	XLOC_022030	-	lincRNA	1540	chr6	113943231	113944979	-	3	0.95525	2.77E-05	0	1.88E-05	0.0231646	0	0	0	0.823275
TCONS_00249802	XLOC_022030	-	lincRNA	2549	chr6	113943231	113949769	-	3	0.82178	0	0.16936	0.134664	0.204767	0.320591	0.0850101	0.346669	0.269009
TCONS_00256464	XLOC_022030	-	lincRNA	1457	chr6	113943592	113945164	-	2	0.57156	0.31266	0.67544	0.531008	0.501327	0.813228	0.505415	0.353715	1.725
TCONS_00251694	XLOC_021304	-	lincRNA	849	chr6	114312961	114317249	+	3	1.09842	1.42403	0.21686	0.494065	1.81046	0.434672	0.637513	1.12608	1.16317
TCONS_00256070	XLOC_021323	-	lincRNA	1119	chr6	119855041	119872362	+	3	1.22014	1.00316	1.60161	1.53389	1.14101	1.83962	2.04674	2.02362	1.44964
TCONS_00256904	XLOC_021326	-	lincRNA	2800	chr6	122088454	122286654	+	5	2.8664	2.90726	2.59869	5.11921	2.6937	2.70436	1.52423	1.54472	2.37443
TCONS_00258381	XLOC_022057	-	lincRNA	449	chr6	125694290	125695466	-	2	0.39752	0.86811	0	0.258737	1.25941	0.278438	0.734111	1.00143	0.293009
TCONS_00249834	XLOC_022057	-	lincRNA	335	chr6	125694413	125695451	-	2	2.98928	0	0	0.478854	0.471898	1.06975	0	0	0.77963
TCONS_00255608	XLOC_022058	-	lincRNA	2016	chr6	126041153	126068081	-	3	1.23635	0.5451	1.07556	0.458222	1.8624	1.51068	1.09333	1.32902	0.222603
TCONS_00250485	XLOC_021342	-	lincRNA	4850	chr6	126428102	126437160	+	5	4.94168	2.61064	3.44019	1.49951	2.65303	3.14381	2.06374	2.67327	3.03706
TCONS_00259808	XLOC_021344	-	lincRNA	289	chr6	126653929	126656983	+	2	0	0	0	0	0	3.18639	0	0.970587	0.67234
TCONS_00256484	XLOC_022061	-	lincRNA	712	chr6	127320997	127325509	-	2	1.19238	0.835	0.41385	0.626754	0.242139	0.413681	0.928239	2.70673	0.795748
TCONS_00260124	XLOC_022068	-	anti-sense_lncRNA	29700	chr6	129774304	129804095	-	2	1.62083	1.07078	1.22876	0.784717	1.53387	2.21702	1.44787	2.18205	1.94225
ENST00000440246.1	ENSG00000236166.1	RP3-523C21.2	lincRNA	490	chr6	132411673	132420366	+	2	1.04921	1.23856	0.74046	0	0.646753	0.733196	0.834649	0.28511	1.01192
TCONS_00256504	XLOC_022082	-	lincRNA	286	chr6	133125622	133126016	-	2	2.27737	0	0	2.97386	0	0.823023	2.24884	0	1.39017
TCONS_00256511	XLOC_022086	-	lincRNA	916	chr6	134768293	134775659	-	2	0.28455	0.29764	0	1.07451	0.431497	0	0	0.113258	0.0810369
TCONS_00254385	XLOC_022088	-	lincRNA	521	chr6	134844952	134845588	-	2	0.63238	0.67017	1.77863	1.20225	0.388795	0.441218	0.187743	0	0.365003
ENST00000418837.1	ENSG00000232310.2	RP11-557H15.4	lincRNA	892	chr6	134846463	134861482	-	3	0	0.61608	0.30482	0.555937	0.714538	1.12079	0.0853138	0.351717	0.16774
TCONS_00249910	XLOC_022099	-	intronic_lncRNA	1676	chr6	137357932	137365567	-	2	1.86635	0.86289	1.08822	0.173511	0.541998	0.572089	0.871752	1.41883	0.508994
TCONS_00258056	XLOC_021382	-	lincRNA	1314	chr6	137893115	137897527	+	2	3.57018	0.31796	1.38185	0.114995	1.10619	1.07444	1.0002	0.869433	0.986861
TCONS_00252764	XLOC_021384	-	lincRNA	4313	chr6	138145358	138185334	+	3	3.48797	1.11478	0.67968	0.276922	0.631798	0.451107	0.72495	0.312061	1.24148
TCONS_00250520	XLOC_021384	-	lincRNA	488	chr6	138145448	138146030	+	2	1.59193	1.00917	0.94874	1.38468	0.362141	0.886646	2.04914	0	1.31038
TCONS_00253917	XLOC_021393	-	intronic_lncRNA	433	chr6	139592465	139593006	+	2	0.43447	1.23874	2.16633	0.553272	0.269587	1.21928	2.09901	0	1.01222
TCONS_00259832	XLOC_021398	-	lincRNA	724	chr6	141298057	141361546	+	2	0.97098	0.13595	0.40421	0.24494	0.354799	2.0208	14.8436	0.466522	0.111047
TCONS_00261456	XLOC_021399	-	lincRNA	514	chr6	141725299	141767546	+	2	0	0.22845	0	0	0	0.451134	2.88138	0	0.186642
TCONS_00263287	XLOC_022128	-	lincRNA	2803	chr6	143376545	143381787	-	2	1.17662	0.51565	1.3915	0.565414	1.18008	1.21556	0.78362	1.85152	0.997438
TCONS_00254409	XLOC_022134	-	lincRNA	2813	chr6	145203501	145283897	-	4	0.68862	0.27035	2.26572	0.0244891	0.258622	0.269089	0.18098	0.789978	0.456115
TCONS_00254410	XLOC_022134	-	lincRNA	1190	chr6	145257220	145284046	-	3	1.13283	0.50121	2.71307	0	0.379313	0.284557	0.303336	0.430907	0.740873
TCONS_00261799	XLOC_022134	-	lincRNA	530	chr6	145257856	145283904	-	4	0.42296	0	4.2478	0	0.36086	0.214546	1.41779	2.75063	1.00689
TCONS_00249100	XLOC_021413	-	lincRNA	535	chr6	145850892	145857407	+	2	1.21224	1.92512	1.48954	0.191954	0	0	0.179609	0	0.349496
TCONS_00251804	XLOC_021442	-	intronic_lncRNA	5478	chr6	152084269	152090097	+	2	1.68352	1.61781	1.5151	3.44195	1.17433	2.19701	1.50919	1.88371	1.3322
TCONS_00259853	XLOC_021443	-	intronic_lncRNA	1137	chr6	152388954	152392334	+	2	1.74074	1.05945	0.44876	0.683889	0.460778	0.676612	0.564495	0.949075	0.865351
TCONS_00256553	XLOC_022163	-	lincRNA	3585	chr6	156486210	156497258	-	2	4.13268	1.27243	2.1384	0.756034	0.925143	0.747619	1.28988	1.58857	1.02218
TCONS_00258452	XLOC_022167	-	lincRNA	298	chr6	157628587	157638979	-	2	0	0	0	0	1.30449	0	0	0	0
TCONS_00258115	XLOC_021488	-	intronic_lncRNA	1156	chr6	161774193	161775742	+	2	0.42656	0.14835	0.07329	0	0.193515	0.147373	1.10619	0.760942	0.242297
TCONS_00257724	XLOC_022202	-	intronic_lncRNA	3064	chr6	167170374	167184598	-	3	0.74863	0.49329	0.26745	1.87675	1.73725	1.86572	0.244082	0.476633	0.342404
TCONS_00252882	XLOC_021502	-	lincRNA	786	chr6	167654877	167660717	+	3	1.3898	1.09285	0.3608	0	0.105631	0.120403	0.10107	0.277577	0.694302
TCONS_00251868	XLOC_021514	-	lincRNA	322	chr6	169112554	169117008	+	2	0	1.19975	0	0.5296	0	0	0	0.703034	0
TCONS_00259540	XLOC_022220	-	intronic_lncRNA	4560	chr6	169957744	169962470	-	2	5.0594	2.25027	3.06322	1.39387	2.8438	3.06287	1.56098	2.07881	2.922
TCONS_00254005	XLOC_021522	-	lincRNA	769	chr6	170462342	170466316	+	3	3.34921	0.46669	3.08305	0.191272	2.93844	0.378482	1.27E-05	0.868366	0.409267
TCONS_00254004	XLOC_021522	-	lincRNA	623	chr6	170462474	170466316	+	3	0.78792	0.21427	1.36078	0.349785	1.76223	0.325507	0.282298	0.372958	0.55091
TCONS_00271801	XLOC_022911	-	lincRNA	2178	chr7	145842	149421	-	3	2.20958	1.11975	3.47368	1.18712	2.60147	0.682683	1.4775	3.2678	1.77986
TCONS_00275861	XLOC_022911	-	lincRNA	2433	chr7	146026	149150	-	2	1.48837	1.29431	3.51013	0.999665	2.43515	1.91484	2.94955	0.490288	1.71535
ENST00000497320.1	ENSG00000242474.1	AC093627.9	lincRNA	429	chr7	148328	149466	-	2	0	0.92257	0.78978	0.966456	1.64954	0	0.489746	1.71256	0.0005158

ENST00000487884.1	ENSG00000242474.1	AC093627.9	lincRNA	496	chr7	148329	149438	-	2	2.95389	0.68972	3.74414	0	2.28205	0.957609	5.75336	1.4786	0.922644
TCONS_00277694	XLOC_022251	-	lincRNA	689	chr7	1609732	1615524	+	3	1.87232	1.45785	1.87925	1.57523	1.14148	1.44414	2.31088	1.83539	2.73899
ENST00000430844.1	ENSG00000228010.1	AC073343.13	lincRNA	532	chr7	6703605	6713232	+	3	2.44626	1.07933	0.85906	0.774792	0.751385	1.4927	0.906463	1.48813	0.52906
TCONS_00270356	XLOC_022284	-	lincRNA	700	chr7	6746736	6748031	+	2	2.44174	1.85305	0.7066	2.05393	0.868059	1.27098	0.951067	0.652454	1.04793
TCONS_00267197	XLOC_022289	-	lincRNA	696	chr7	7030893	7034026	+	2	8.4096	4.16744	3.27706	0.388229	1.12518	1.99311	2.51719	2.96017	1.99557
TCONS_00265779	XLOC_022289	-	lincRNA	3876	chr7	7030911	7141289	+	3	1.91379	0.59535	0.81963	0.165345	0.651341	0.524932	0.340785	0.425552	0.755895
TCONS_00272278	XLOC_022289	-	lincRNA	380	chr7	7134514	7137751	+	2	6.22658	2.82392	4.33259	0.896747	0.0006474	1.79961	7.40152	1.17238	2.24507
TCONS_00271820	XLOC_022969	-	lincRNA	3554	chr7	7588737	7595205	-	3	2.55307	0.8	2.05446	0.934551	1.19866	1.44619	0.985386	1.00553	1.45576
ENST00000609307.1	ENSG00000272894.1	RP5-1159O4.1	lincRNA	248	chr7	7592368	7594380	-	2	3.96644	1.41439	1.47439	0.0100458	4.39958	0	9.31902	1.6753	0.388829
ENST00000608807.1	ENSG00000272894.1	RP5-1159O4.1	lincRNA	194	chr7	7592484	7603957	-	3	0	0	5.37444	0	0	0	0	0	0
TCONS_00275583	XLOC_022292	-	lincRNA	1031	chr7	7826190	7828928	+	2	0.98111	0.59774	0.75987	1.15684	0.594198	1.3567	0.708336	0.779252	0.97646
TCONS_00270807	XLOC_022972	-	lincRNA	1420	chr7	7985237	7990313	-	2	1.42126	1.04476	1.43286	0.734915	1.00963	0.923187	0.528251	1.32218	0.995539
TCONS_00272637	XLOC_022974	-	lincRNA	207	chr7	10130290	10139852	-	2	4.22346	0	0	0	0	0	0	4.18681	0
TCONS_00267222	XLOC_022300	-	lincRNA	703	chr7	12543785	12570725	+	4	13.8812	10.068	20.9403	0.968537	5.60867	1.89436	10.765	7.45965	7.07745
ENST00000424453.1	ENSG00000226690.2	AC005281.1	lincRNA	562	chr7	12544050	12580761	+	4	5.99702	9.47811	19.8337	2.38106	4.90249	1.85768	7.93851	10.663	3.36719
TCONS_00271251	XLOC_022307	-	lincRNA	1608	chr7	17029581	17062110	+	2	1.73897	0.90472	1.43879	0.864033	1.35513	1.24952	1.03888	1.60225	0.985242
TCONS_00271258	XLOC_022309	-	lincRNA	2443	chr7	17413922	17507393	+	3	1.10179	0	1.16288	0.124956	1.21231	0.47452	0.759061	0.266065	0.282618
TCONS_00271840	XLOC_023000	-	lincRNA	8769	chr7	17470089	17479304	-	2	1.32321	0.58448	1.32998	0.432904	0.873346	2.09095	0.759269	1.28102	1.57994
TCONS_00277431	XLOC_023000	-	lincRNA	877	chr7	17475793	17497813	-	3	0.75224	0.10497	0.93499	0.284145	0.365252	2.3956	1.39582	0.958985	1.20043
TCONS_00265803	XLOC_022309	-	lincRNA	1418	chr7	17490397	17506904	+	2	1.99217	0.29069	5.14236	0.295528	1.45393	0.454562	1.38572	0.898897	0.52315
TCONS_00270819	XLOC_023000	-	lincRNA	4858	chr7	17498933	17523274	-	5	1.09372	0.40809	0.90601	0.680194	0.583395	1.84129	0.669094	1.55794	1.13761
TCONS_00274347	XLOC_023000	-	lincRNA	1675	chr7	17499222	17598558	-	6	1.40714	0.17584	1.60141	0.0278066	1.2974	2.55244	1.53657	1.01095	2.11844
TCONS_00266566	XLOC_023000	-	lincRNA	560	chr7	17502744	17504930	-	2	0	0	0.4717	1.02414	0.0008207	0.877362	0.943734	0	0
TCONS_00268894	XLOC_023000	-	lincRNA	374	chr7	17522147	17523192	-	2	0.44825	2.2555	0.04743	0.40643	3.49986	0	0	0	0
TCONS_00268386	XLOC_022310	-	intronic_lncRNA	8775	chr7	18327943	18336810	+	2	2.39441	2.59953	4.60306	1.67819	2.82566	2.04033	2.24234	1.75667	1.54524
TCONS_00266572	XLOC_023005	-	lincRNA	331	chr7	19867569	19868038	-	2	0	0	0	0	0	1.08986	0	0.652788	0.456584
TCONS_00268900	XLOC_023006	-	lincRNA	1731	chr7	19999163	20008035	-	2	0.5109	0.11771	0.39955	1.63877	0.737003	1.35998	0.585758	0.946763	1.14194
TCONS_00274352	XLOC_023006	-	lincRNA	516	chr7	20000507	20008181	-	2	0.42839	0.32976	0.73181	0.572283	1.71089	1.21222	0.698839	0	0.508726
ENST00000432668.1	ENSG00000228649.4	AC005682.5	lincRNA	574	chr7	22895705	22896694	+	2	2.44476	1.14763	1.71066	2.92123	2.16358	4.91739	1.92317	1.09733	2.50006
TCONS_00272986	XLOC_022328	-	lincRNA	2936	chr7	22898148	22909459	+	2	1.11946	1.44577	2.06182	2.47906	2.06558	4.44576	1.17128	1.37961	1.81336
TCONS_00266580	XLOC_023015	-	lincRNA	277	chr7	22928141	22929162	-	2	2.51551	0	0	0	0.824236	0	0	0	0
TCONS_00266591	XLOC_023026	-	lincRNA	1890	chr7	25397732	25419075	-	2	2.29452	0.79512	1.65166	0.0378787	1.01906	0.333023	0.311143	0.619085	0.375971
TCONS_00276698	XLOC_023030	-	anti-sense_lncRNA	4908	chr7	26211781	26227104	-	2	4.16497	2.12872	2.30455	1.60331	2.56816	2.47807	2.49531	2.06112	2.718
TCONS_00275602	XLOC_022345	-	lincRNA	563	chr7	26600571	26609737	+	3	0	0	0.11972	0	0	3.48472	0	0	0
TCONS_00275601	XLOC_022345	-	lincRNA	1600	chr7	26600571	26607380	+	3	0.21861	0.40441	0.06931	0.0914712	0	2.41888	0.125385	0.115105	0.0412873
ENST00000411479.1	ENSG00000233517.1	AC005162.5	lincRNA	578	chr7	29026644	29028515	+	2	1.07512	0	0.18801	0.170009	0.658565	0.748461	0.158513	1.08543	0.772879
TCONS_00265849	XLOC_022352	-	lincRNA	2491	chr7	29186217	29188810	+	2	1.9605	2.37499	2.67678	1.8157	2.06543	1.96449	2.18907	1.78899	3.12371
TCONS_00268924	XLOC_023041	-	intronic_lncRNA	553	chr7	29336813	29337836	-	2	1.43851	3.24523	1.815	0.364228	0	0	0.340263	0	0
TCONS_00271878	XLOC_023042	-	anti-sense_lncRNA	1211	chr7	29552180	29603533	-	3	2.82379	2.73414	4.15598	3.10493	6.34152	5.15468	3.31	2.15738	1.317
TCONS_00270394	XLOC_022361	-	lincRNA	6913	chr7	30256980	30267297	+	2	2.75403	1.35578	1.62569	0.895636	1.26124	1.08865	1.22062	1.36108	1.15877
TCONS_00268928	XLOC_023046	-	lincRNA	2335	chr7	30283397	30295869	-	2	0.76495	1.12552	1.20804	0.779394	1.41066	0.757638	0.819744	1.01662	1.35183
TCONS_00268946	XLOC_023069	-	lincRNA	2571	chr7	35652731	35655396	-	2	2.7555	1.63905	1.49878	1.75439	2.28066	3.02516	1.18037	1.45727	2.14185
TCONS_00266639	XLOC_023070	-	lincRNA	587	chr7	35790018	35792592	-	2	2.62565	1.29329	0.55062	0.166036	0.321511	1.46187	0.154709	0.211913	0.301858
TCONS_00270852	XLOC_023070	-	lincRNA	743	chr7	35795392	35833990	-	4	2.624	1.96711	2.98876	0.590848	1.59718	2.20972	1.63883	1.34974	2.03531
TCONS_00268449	XLOC_022384	-	lincRNA	401	chr7	37723392	37725348	+	2	1.00562	3.9624	2.52581	3.85237	1.56826	1.41561	2.14628	0.834228	3.53277
TCONS_00265890	XLOC_022389	-	lincRNA	2835	chr7	37997840	38000989	+	2	2.24719	0.75065	0.92518	0.194276	0.419659	0.026684	0.619248	0.335308	0.131373
TCONS_00268452	XLOC_022392	-	lincRNA	1998	chr7	38370635	38374027	+	2	0.62435	0.4326	0.58199	0.534017	0.889251	0.391239	0.649617	0.760691	0.867234
ENST00000577700.1	ENSG00000232956.4	SNHG15	lincRNA	3116	chr7	45022622	45026236	-	2	2.17085	2.37363	2.81768	5.22238	3.24375	3.64066	2.35716	2.42314	2.67051
TCONS_00277484	XLOC_023109	-	lincRNA	571	chr7	45034461	45039166	-	2	0.82147	0.77137	0.38329	0.173233	0	0	0.323206	1.549	0.157536
TCONS_00272349	XLOC_022420	-	lincRNA	2083	chr7	45225429	45229751	+	2	0.9214	0.71338	0.51855	1.15574	1.17552	0.635041	0.868145	0.854332	1.28796
TCONS_00270441	XLOC_022421	-	lincRNA	220	chr7	45242537	45243892	+	2	0	0	2.58187	0	2.16171	2.34657	2.27906	2.99772	0
TCONS_00272351	XLOC_022424	-	lincRNA	249	chr7	45817845	45821119	+	3	0	0	0	1.21267	0	2.69361	1.2668	0	0
TCONS_00278495	XLOC_022425	-	lincRNA	3358	chr7	46017971	46033516	+	4	0.84064	0.93918	1.23445	0.364685	0.252802	0.578791	0.682255	1.24555	0.949677
TCONS_00268990	XLOC_023119	-	lincRNA	3294	chr7	46812949	46825848	-	3	0.33001	1.01796	0.07449	0.206796	0.661744	0.59857	2.50992	2.41239	1.18131
TCONS_00279368	XLOC_023119	-	lincRNA	1724	chr7	46823697	46825768	-	2	0.33475	0.90066	0.16903	0.294068	1.31868	1.13841	2.17354	2.5377	1.69217
TCONS_00274068	XLOC_022428	-	lincRNA	488	chr7	47009293	47012310	+	3	0.35215	0.49893	0	0.223481	1.30267	0.492221	0.630518	0.287154	1.01908
TCONS_00269006	XLOC_023132	-	intronic_lncRNA	696	chr7	50794860	50795912	-	2	1.23067	2.01187	1.28238	2.97642	2.12534	1.70838	0	0.493362	0.35216
TCONS_00272364	XLOC_022448	-	lincRNA	770	chr7	55832457	55841462	+	2	12.1437	0	8.90599	23.4111	15.4249	28.1022	34.8294	13.9894	13.6677
TCONS_00269016	XLOC_023155	-	lincRNA	1588	chr7	63384145	63386418	-	2	0.441	1.12177	1.15766	0.691973	0.443463	1.01405	0.590231	0.17416	0.791267
TCONS_00268501	XLOC_022464	-	lincRNA	929	chr7	64338160	64340701	+	2	2.6552	1.16915	1.83136	0.37606	1.01698	0.676899	0.890021	1.1121	0.761915
TCONS_00272371	XLOC_022464	-	anti-sense_lncRNA	367	chr7	64340375	64354006	+	3	0	0	0	1.05376	0	0	0	0	0.863761

TCONS_00269521	XLOC_022464	-	anti-sense_lncRNA	381	chr7	64342928	64354027	+	3	3.34546	1.6042	2.81747	0.713548	1.04784	1.96727	1.37051	2.32697	1.96691
TCONS_00268502	XLOC_022464	-	anti-sense_lncRNA	3528	chr7	64350393	64354013	+	2	7.30229	1.99443	2.96988	0.653642	3.17362	5.13293	2.67658	1.80879	2.46065
TCONS_00270469	XLOC_022475	-	lincRNA	675	chr7	65268361	65279175	+	4	0.64242	0	1.48899	0.810922	0.261228	1.04086	0.37591	2.40618	0.981102
TCONS_00270470	XLOC_022476	-	lincRNA	491	chr7	65292495	65293859	+	2	0	0	1.47569	0.88465	0.214829	0	0	0.284099	0.201675
TCONS_00269024	XLOC_023163	-	lincRNA	840	chr7	65952929	65956590	-	3	5.88729	2.44407	3.8492	2.00413	2.99581	2.09385	3.41801	2.66501	2.81307
ENST00000412091.1	ENSG00000223473.1	GS1-124K5.3	lincRNA	682	chr7	65956996	65958553	-	2	2.74357	3.10646	3.52071	5.06096	2.57389	1.75827	1.48124	3.04785	2.29588
TCONS_00266731	XLOC_023165	-	lincRNA	748	chr7	65994463	65996863	-	2	4.08595	2.72857	2.1886	1.99049	2.37369	2.44704	1.1906	2.52585	2.33495
TCONS_00269532	XLOC_022485	-	lincRNA	2089	chr7	66347588	66359936	+	7	0.44202	0.92938	0.16804	0.943362	0.334059	0.469583	0.221635	0.779422	0.553164
TCONS_00277203	XLOC_022485	-	lincRNA	690	chr7	66347706	66350337	+	3	1.23472	0.86935	1.26485	0.544505	1.36072	1.30849	2.00062	1.4643	0.733587
TCONS_00267407	XLOC_022485	-	lincRNA	941	chr7	66347893	66358650	+	6	3.54914	0.48988	2.19256	1.5385	2.39693	2.457	0.171101	2.34747	0.644745
TCONS_00278513	XLOC_022485	-	lincRNA	1341	chr7	66349567	66359225	+	5	1.45817	0.61934	0.31308	2.08883	1.24365	0.960132	0.342103	0.731855	1.48316
TCONS_00277204	XLOC_022485	-	lincRNA	968	chr7	66350814	66358994	+	4	0.23134	0.19458	0.58958	0.668404	1.06152	0.818729	1.88291	0.748264	0.622506
TCONS_00270483	XLOC_022493	-	intronic_lncRNA	804	chr7	70131895	70132889	+	2	2.19136	1.17776	2.09932	0.212396	1.94662	1.63519	0.391987	0.807527	0.866012
ENST00000435932.1	ENSG00000232415.1	CTB-51J22.1	anti-sense_lncRNA	532	chr7	73473906	73476614	-	2	21.7105	17.0535	31.3557	3.29287	5.63539	5.33108	23.7493	18.1057	11.2866
TCONS_00269058	XLOC_023207	-	lincRNA	946	chr7	75717407	75723328	-	3	1.09257	1.3327	1.3183	0.687458	1.15923	0.472444	0.395185	1.30374	0.699783
TCONS_00270514	XLOC_022533	-	lincRNA	945	chr7	76381632	76400569	+	3	0.68377	0.66725	1.41439	0.774431	0.165829	0.567697	0.712303	0.652758	0.856447
TCONS_00268557	XLOC_022534	-	lincRNA	2129	chr7	76404272	76408672	+	2	1.164	1.06281	0.25307	0.597259	0.541831	0.911583	0.453918	0.542009	0.478916
TCONS_00270523	XLOC_022540	-	lincRNA	638	chr7	77282848	77285243	+	2	0.92856	1.14082	1.29387	0.87972	1.41792	1.77463	0.680937	0.373408	1.46443
ENST00000438324.1	ENSG00000232756.1	RP5-1185I7.1	lincRNA	558	chr7	77619737	77624488	+	2	11.9157	5.79858	9.54144	1.43646	7.6554	4.15053	7.71299	5.96789	5.71712
TCONS_00274902	XLOC_022543	-	anti-sense_lncRNA	531	chr7	77842164	77894282	+	3	0.6134	1.29918	0.64629	0	2.07266	1.06946	0.909303	0.248793	0.176895
ENST00000422093.1	ENSG00000234456.3	MAGI2-AS3	lincRNA	762	chr7	79083308	79089085	+	4	14.8515	16.0863	18.0696	14.7268	10.1373	10.0455	19.4085	10.7163	7.34593
ENST00000442765.1	ENSG00000234456.3	MAGI2-AS3	lincRNA	679	chr7	79099743	79100524	+	2	16.564	9.97387	10.9248	2.94845	5.43943	4.57081	14.1622	5.79382	3.52645
TCONS_00266780	XLOC_023230	-	lincRNA	1390	chr7	81222961	81238032	-	4	1.54273	0.4761	0.88155	0.0538194	0.0517602	0	0.344721	0.271156	0.0972147
TCONS_00272744	XLOC_023236	-	lincRNA	597	chr7	84118190	84122063	-	2	1.79191	0.36008	1.43051	5.1787	4.85608	2.31531	4.97279	4.3354	0.441221
TCONS_00266822	XLOC_023249	-	lincRNA	791	chr7	89598678	89599784	-	2	1.89487	0.36117	0.23847	0.217083	0.209458	0	0.300591	1.51355	0.196678
ENST00000433534.1	ENSG00000227646.3	STEAP2-AS1	lincRNA	1005	chr7	89838584	89840949	-	2	1.1393	0.26451	1.39499	0.159239	0.843597	0.350165	0.438937	1.10648	0.288062
TCONS_00274149	XLOC_022565	-	lincRNA	243	chr7	90941836	90945488	+	2	0	0	0	0	4.07434	0	0	1.86574	0
TCONS_00272437	XLOC_022571	-	lincRNA	744	chr7	92535113	92546872	+	2	0.74834	1.04718	0.51881	0.353855	1.36647	1.68666	0.8724	0.299381	0.106923
TCONS_00277547	XLOC_023273	-	lincRNA	2040	chr7	93925730	93929280	-	2	0.94291	1.03752	0.75819	0.313089	0.701796	0.382295	1.17418	0.918119	0.313844
ENST00000432265.1	ENSG00000231170.1	AC002451.3	lincRNA	771	chr7	95225994	95243031	+	2	1.06963	0.49859	0.37042	0	0.325302	0.247161	0.830259	0.56998	0.71272
ENST00000448513.1	ENSG00000227053.1	RP11-395B7.4	anti-sense_lncRNA	372	chr7	100657606	100660882	-	3	0	0	2.12257	0.375569	1.47226	0	9.40432	1.96362	2.41807
TCONS_00268119	XLOC_023343	-	lincRNA	1486	chr7	102339661	102389278	-	3	9.67701	3.30305	3.15245	1.44408	3.20791	1.53249	6.55702	2.63305	3.19238
TCONS_00274195	XLOC_022655	-	intronic_lncRNA	258	chr7	102628507	102628906	+	2	0	0	0	1.0576	0	4.69412	0	0	0
TCONS_00277970	XLOC_022657	-	lincRNA	717	chr7	102789908	102855164	+	3	1.70513	0.82683	1.15304	0.322693	1.38957	1.06659	1.19541	1.02329	0.724656
TCONS_00267582	XLOC_022657	-	lincRNA	612	chr7	102835038	102855145	+	2	1.31656	0.52019	1.47347	0.218351	0.966921	0.547193	0.521329	0.498715	0.7879
ENST00000417026.1	ENSG00000237513.1	RP11-325F22.2	lincRNA	891	chr7	104581653	104602781	+	2	0.44223	0.30849	1.52634	5.75302	0.805028	1.93872	0.683516	1.40893	1.76384
TCONS_00269183	XLOC_023353	-	lincRNA	1266	chr7	104613966	104615567	-	2	1.14788	1.46235	0.5253	0.480727	0.867199	0.792661	0.165119	0.454463	0.380037
TCONS_00274201	XLOC_022667	-	lincRNA	1999	chr7	105549250	105560024	+	4	2.89312	1.5722	0.89188	1.17418	1.67496	1.56408	2.14254	0.983874	0.674139
TCONS_00277593	XLOC_023360	-	lincRNA	1215	chr7	106011715	106025945	-	2	0.40181	0.62846	0.34496	1.76724	0.303673	0.416296	0.578385	0.47751	1.36881
TCONS_00266947	XLOC_023362	-	lincRNA	327	chr7	106088636	106106270	-	2	0	0	0	1.0178	0.502274	1.68629	0.499465	0	0.471241
TCONS_00266968	XLOC_023383	-	lincRNA	428	chr7	114830046	114871375	-	2	5.32675	0.31663	4.43125	0	0.27564	0.934725	1.61059	1.82895	0.776208
TCONS_00278332	XLOC_023384	-	lincRNA	1144	chr7	115731582	115799936	-	5	1.50772	3.10246	3.5644	4.36892	2.22122	1.22249	4.72101	7.15382	12.5412
TCONS_00270196	XLOC_023384	-	lincRNA	722	chr7	115750485	115799993	-	3	2.73663	4.87133	7.84265	4.0116	0	1.97667	3.77145	4.59865	14.8906
TCONS_00272516	XLOC_022705	-	lincRNA	874	chr7	121120246	121130267	+	3	0.15112	0.42178	0.20871	0.475688	1.1924	0.62773	0.350535	0.481656	0.258386
TCONS_00276536	XLOC_022724	-	lincRNA	771	chr7	128263250	128266474	+	3	8.02223	5.23521	4.3216	13.1456	4.55423	8.7742	3.73616	4.27485	7.63628
TCONS_00269798	XLOC_022737	-	intronic_lncRNA	2759	chr7	128952479	128958049	+	2	4.86711	2.31898	3.45711	1.87547	4.27348	4.64366	2.73311	3.23333	4.71237
TCONS_00275085	XLOC_022753	-	lincRNA	2196	chr7	133754462	133757463	+	2	2.24994	1.09787	0.97814	1.18645	2.27916	2.1495	0.789459	1.8532	1.56193
TCONS_00272848	XLOC_023437	-	lincRNA	240	chr7	134948480	134951003	-	2	0	0	0	1.41089	2.86585	0	2.97797	0	0
TCONS_00270260	XLOC_023456	-	lincRNA	1559	chr7	139945534	139960485	-	2	3.97848	3.07283	2.05656	1.64911	5.07321	3.83219	2.45482	2.54994	1.65898
TCONS_00272860	XLOC_023458	-	lincRNA	1389	chr7	139994204	139996224	-	2	1.20091	0.23825	0.4443	0.0538647	0.182507	0.445215	0.492877	0.407064	1.10963
TCONS_00271120	XLOC_023458	-	lincRNA	406	chr7	139995784	139997303	-	2	0	0.70254	5.08107	0	0.45172	1.54672	2.38999	8.73E-05	1.77724
TCONS_00270691	XLOC_022789	-	intronic_lncRNA	680	chr7	141770850	141778421	+	5	0.21192	0.44565	1.03123	0	2.19703	0.147137	0.867751	0.340089	1.09211
ENST00000471935.1	ENSG00000241657.1	TRBV11-2	lincRNA	408	chr7	142197570	142198069	-	2	0	0.34781	11.4906	1.86088	0.302829	0.341867	2.66143	2.01241	1.42122
TCONS_00274625	XLOC_023500	-	lincRNA	375	chr7	143210637	143216521	-	2	1.15279	0.41515	0.41692	0.73818	0.723171	2.84961	0.355118	1.9284	0.339361
TCONS_00274271	XLOC_022828	-	lincRNA	3118	chr7	148582300	148586290	+	2	2.09939	0.94396	2.6487	0.328916	2.33642	0.626438	2.27569	1.78859	1.58144
TCONS_00269314	XLOC_023520	-	intronic_lncRNA	1517	chr7	150458326	150463066	-	2	1.05135	1.97826	0.10613	0.978553	1.4785	0.718426	0.482405	0.734465	3.71543
TCONS_00276126	XLOC_023520	-	intronic_lncRNA	1562	chr7	150458373	150478989	-	2	0.78095	0.47783	0.3078	1.73359	0.559191	0.752173	1.08064	0.591693	1.92553
TCONS_00268828	XLOC_022882	-	lincRNA	2169	chr7	152921816	152926082	+	2	1.91577	2.06334	0.81116	0.487595	0.621851	0.335762	0.842352	0.939366	0.415141
TCONS_00275854	XLOC_022882	-	lincRNA	1102	chr7	152923213	152935860	+	4	0.79572	0.59197	0.08513	0.142079	0.484456	0.515505	1.27647	0	0.310982
TCONS_00267790	XLOC_022887	-	lincRNA	1986	chr7	153554434	153562701	+	2	8.85675	2.33593	2.18743	1.07522	0.55092	0.393871	0.294305	0.855927	0.129345
TCONS_00275542	XLOC_023546	-	anti-sense_lncRNA	2450	chr7	156449461	156452330	-	2	1.20854	0.91082	1.26995	1.0524	1.55097	1.78155	1.22551	1.35736	1.43835

TCONS_00278412	XLOC_023546	-	anti-sense_lncRNA	528	chr7	156450235	156450946	-	2	0.73083	0	0	0	0.231353	0	0.683077	0	0.701091
TCONS_00276143	XLOC_023551	-	intronic_lncRNA	294	chr7	158211151	158211497	-	2	0	0	0	0.683313	0	3.77939	0	1.83751	0.637322
TCONS_00268841	XLOC_022907	-	lincRNA	1765	chr7	158785477	158795441	+	2	1.76036	2.53133	0.66912	0.613618	0.196563	0.584459	0.597667	0.411587	0.701458
TCONS_00282816	XLOC_023566	-	intronic_lncRNA	1195	chr8	1821716	1823044	+	2	4.50859	5.05801	1.19663	1.80283	3.09811	3.11436	2.53766	1.3804	1.51286
TCONS_00291779	XLOC_024088	-	lincRNA	401	chr8	2199482	2199953	-	2	3.01685	1.08066	0	0	0.313651	0	0	0.417114	1.76638
TCONS_00284596	XLOC_024098	-	lincRNA	1487	chr8	4969991	5003332	-	3	0.31703	0.27504	0.27155	0.298543	2.10504	1.36723	0.955482	1.25285	0.22464
TCONS_00288432	XLOC_023582	-	lincRNA	693	chr8	5382735	5596231	+	2	0.82542	0.14459	0	0	0.880527	1.5756	0.241227	0.496422	0
TCONS_00282823	XLOC_023605	-	lincRNA	383	chr8	8777533	8793274	+	3	0	0.97528	0	0.0003106	0.34536	0	0.404948	2.99425	0.554804
TCONS_00280891	XLOC_023605	-	lincRNA	1554	chr8	8790787	8794031	+	2	1.65753	0.86466	1.18692	1.13496	0.454645	1.19549	0.380586	0.862569	1.33588
TCONS_00280916	XLOC_023633	-	lincRNA	708	chr8	16546161	16546968	+	2	0.20029	1.12219	0	0	0	0.138985	0	0.160497	0
TCONS_00287663	XLOC_023639	-	lincRNA	2626	chr8	17658849	17679934	+	4	8.17725	3.67079	6.75782	1.51484	8.17782	6.66786	5.60225	3.58819	5.08114
ENST00000520646.1	ENSG00000253671.1	RP11-806O11.1	lincRNA	667	chr8	17666450	17678377	+	3	3.33687	2.81502	4.5044	0	1.03079	1.00761	5.84818	2.91221	0.632636
ENST00000520156.1	ENSG00000253671.1	RP11-806O11.1	lincRNA	745	chr8	17666720	17678134	+	2	8.84233	3.85798	7.24466	1.71567	8.793	9.23308	5.11641	2.98877	2.48795
TCONS_00283549	XLOC_023644	-	lincRNA	4674	chr8	18027957	18032801	+	2	5.27294	2.47683	5.48806	0.300265	1.3719	1.3353	1.67711	1.14732	1.72651
TCONS_00287410	XLOC_024151	-	lincRNA	299	chr8	21745041	21765911	-	2	0	0	0.7529	0	2.5828	0.718865	0	0	0.605521
TCONS_00291642	XLOC_023680	-	lincRNA	260	chr8	23866439	24063102	+	2	0	0	1.21865	0	1.03465	0	1.06115	0	5.81409
TCONS_00281479	XLOC_024174	-	intronic_lncRNA	2144	chr8	24242826	24245378	-	2	0.73502	0.54548	1.97296	1.15238	0.695771	1.15781	1.65144	1.90301	3.62348
TCONS_00281007	XLOC_023693	-	lincRNA	3051	chr8	27447167	27450940	+	2	1.71895	0.27251	0.78159	0.0897735	0.473979	0.320586	0.2452	0.591476	0.384435
TCONS_00281515	XLOC_024201	-	lincRNA	3708	chr8	30430513	30435516	-	2	3.37659	2.43536	3.13453	1.89673	2.78297	2.30443	1.54317	1.37259	2.86021
TCONS_00284348	XLOC_023710	-	intronic_lncRNA	359	chr8	31875375	31882617	+	2	0.63334	0	0.46085	0	1.99505	1.79388	1.96623	2.13208	0
TCONS_00282927	XLOC_023713	-	lincRNA	766	chr8	32785521	32902306	+	4	0.53952	1.29535	0.7042	3.17385	0.65641	0.498708	0.418876	0.762936	1.64361
TCONS_00281029	XLOC_023713	-	lincRNA	307	chr8	32853735	32899359	+	2	0	0.47889	0.93725	0	0	0	0	0.558423	0
TCONS_00284654	XLOC_024214	-	lincRNA	3336	chr8	37373133	37380921	-	3	1.79446	0.87131	1.1182	0.368535	0.450124	0.564466	0.665975	0.483601	0.337269
TCONS_00291805	XLOC_024214	-	lincRNA	1977	chr8	37373291	37412271	-	5	2.29062	0.52952	1.00614	0.213856	0.450401	0.745036	0.563196	0.50295	0.671512
TCONS_00283931	XLOC_024216	-	lincRNA	1417	chr8	37417349	37457673	-	3	0.51518	1.10506	0	0	0	0.117437	0.400341	0.144603	0
TCONS_00281525	XLOC_024216	-	lincRNA	3339	chr8	37425007	37457412	-	2	3.79987	2.2337	2.639	0.663908	0.54372	1.28057	1.41626	1.10147	0.99645
TCONS_00289272	XLOC_024216	-	lincRNA	1561	chr8	37454516	37457400	-	2	3.15073	1.96161	1.2324	0.208343	0.235577	1.12683	1.13278	0.87325	0.372413
TCONS_00281541	XLOC_024237	-	lincRNA	2711	chr8	41298053	41305453	-	2	1.38231	1.4912	0.69359	0.688084	1.37023	2.24025	0.88211	1.18376	1.28683
ENST00000521802.1	ENSG00000253408.1	RP11-231D20.2	lincRNA	415	chr8	42112846	42123216	-	3	1.39292	0.67245	0	0.29993	1.1709	0.0002687	1.99892	1.94418	0.67898
TCONS_00281549	XLOC_024244	-	lincRNA	483	chr8	42112855	42121151	-	2	1.44729	0	0.25312	3.41E-05	0	0.751289	0	0	0.939683
TCONS_00283960	XLOC_024255	-	lincRNA	1195	chr8	49531907	49533566	-	2	1.43455	1.35355	0.84468	1.03019	0.743548	0.77859	0.649169	0.324799	0.232747
TCONS_00281560	XLOC_024256	-	lincRNA	1326	chr8	49623766	49625433	-	2	3.16993	0.88076	1.86409	0.113767	0.93019	0.43769	0.676977	0.215022	0.667989
TCONS_00284378	XLOC_023769	-	lincRNA	935	chr8	50080585	50105816	+	2	0.13861	0.28988	1.81622	0	0.252154	1.05499	0.641916	1.4338	2.05208
TCONS_00287479	XLOC_024264	-	lincRNA	281	chr8	54179940	54190937	-	2	0	0	0	2.35903	1.57097	0	1.59224	0	0
ENST00000521558.1	ENSG00000253369.1	RP11-1081M5.1	lincRNA	594	chr8	54307771	54308506	-	2	0.25793	1.45142	0.36041	0	0	0.358932	0.607496	1.04029	0.444628
ENST00000426023.1	ENSG00000237807.3	RP11-400K9.4	lincRNA	2387	chr8	54427731	54436491	-	2	8.16787	8.9511	9.52919	6.64277	6.23879	7.42818	7.97477	9.0046	5.33122
TCONS_00282964	XLOC_023779	-	lincRNA	370	chr8	55422023	55445539	+	2	0.59307	0.42766	0	2.28009	0	1.67674	0	0	0
ENST00000602362.1	ENSG00000254142.2	RP11-53M11.3	lincRNA	241	chr8	55467143	55471480	+	2	4.15232	0	0	0	0	0	2.92258	0	2.63325
TCONS_00282968	XLOC_023779	-	intronic_lncRNA	4918	chr8	55697332	55702818	+	2	6.99185	2.64777	3.99492	0.366081	1.6521	2.01103	4.10481	3.39957	2.82219
TCONS_00282969	XLOC_023780	-	lincRNA	3213	chr8	55795367	55800796	+	2	6.37081	2.46115	2.38009	0.488426	0.387295	1.89001	3.09284	1.65208	2.25894
TCONS_00284386	XLOC_023786	-	lincRNA	558	chr8	58768019	58871026	+	3	3.40449	0.7998	4.9695	0.359114	0.521959	0.395289	2.17976	0.688603	0.816731
TCONS_00282974	XLOC_023788	-	lincRNA	431	chr8	59116021	59131659	+	2	2.62914	2.81183	1.56127	0.558097	0.271976	0	0.794298	0	0.765894
TCONS_00281112	XLOC_023796	-	lincRNA	376	chr8	61945736	61946260	+	2	2.29269	0.82546	0	0.366979	0	0.809501	0	0.479232	0.337382
TCONS_00283288	XLOC_024299	-	lincRNA	6864	chr8	67116174	67133447	-	3	3.50999	2.36812	3.19384	0.986962	1.05549	1.3	1.99832	1.89395	2.49888
TCONS_00281610	XLOC_024299	-	lincRNA	553	chr8	67123643	67133526	-	2	0	0.26852	1.7771	0.213329	0.239457	0.745036	0.655286	0.355924	0
TCONS_00282530	XLOC_024305	-	anti-sense_lncRNA	1376	chr8	68087531	68093471	-	2	2.25487	2.10784	1.36792	0.871378	1.46662	2.27479	0.946925	1.23484	1.27891
TCONS_00283307	XLOC_024312	-	lincRNA	4046	chr8	72379487	72460010	-	3	7.8765	4.43751	5.16377	1.3754	3.77109	3.71847	3.6758	2.73677	2.03127
TCONS_00283308	XLOC_024312	-	lincRNA	2226	chr8	72400513	72404688	-	2	4.88673	2.12825	2.34014	0.789774	1.79019	1.90934	1.15215	1.54782	1.11131
TCONS_00284034	XLOC_024312	-	lincRNA	4153	chr8	72438566	72460010	-	4	7.2425	3.62962	4.56861	1.07322	2.86918	2.73014	2.91534	2.41126	1.58252
TCONS_00283719	XLOC_023817	-	anti-sense_lncRNA	628	chr8	72974042	73164965	+	2	1.18769	2.00196	0.99355	1.50061	0.870898	0.66049	0.697306	0.764653	1.90794
TCONS_00289951	XLOC_024322	-	lincRNA	3108	chr8	73427679	73445907	-	3	0.63198	0.31574	0.40706	0.0440063	0.422428	0.21759	1.42221	0.800738	1.44803
ENST00000518355.1	ENSG00000253339.1	RP11-434I12.3	lincRNA	468	chr8	74271148	74275023	+	2	0.25471	0.17912	0.3558	0.322038	4.75459	2.29953	8.09408	5.12536	7.62497
ENST00000519134.1	ENSG00000253339.1	RP11-434I12.3	lincRNA	599	chr8	74271148	74274710	+	2	0	0	0	0.480543	3.38628	1.59403	5.89262	5.27268	6.11748
TCONS_00289953	XLOC_024326	-	lincRNA	253	chr8	74289722	74295665	-	2	3.438	0	0	0	2.30051	2.52944	5.92452	4.7226	4.30761
TCONS_00282213	XLOC_023841	-	anti-sense_lncRNA	2947	chr8	77912575	77919560	+	2	3.49362	0	0	0.352769	1.39099	0	1.78138	0	1.10995
TCONS_00283731	XLOC_023841	-	anti-sense_lncRNA	2951	chr8	77912575	77919560	+	2	0	2.46474	3.03785	0.136199	0.129427	1.09898	0.127358	2.71493	1.03065
TCONS_00286522	XLOC_023844	-	anti-sense_lncRNA	6786	chr8	79635296	79647480	+	2	5.99506	3.11088	6.10558	1.27221	3.18719	2.64662	1.77957	2.38443	3.23953
TCONS_00287275	XLOC_023844	-	lincRNA	5550	chr8	79635349	79641850	+	2	9.43929	4.374	8.68895	2.00874	4.52934	3.51978	3.5572	3.20817	4.27381
TCONS_00284441	XLOC_023847	-	lincRNA	486	chr8	80655434	80660296	+	2	0	0.50248	0.50076	0	1.53061	0.991387	0.423411	0.578455	0
TCONS_00281674	XLOC_024355	-	lincRNA	415	chr8	86374945	86375489	-	2	2.3522	0.33623	0.67283	0.299975	1.1709	0	0	1.16651	0.549532
TCONS_00282589	XLOC_024364	-	lincRNA	1418	chr8	90659750	90661878	-	2	1.67481	2.03483	1.66486	1.36709	1.41581	2.83191	1.58738	1.7879	1.23461

TCONS_00284457	XLOC_023884	-	lincRNA	3090	chr8	94840969	94844325	+	2	2.40234	1.24639	1.49383	0.154982	0.765115	0.705495	0.765944	0.583468	1.23751
TCONS_00283034	XLOC_023887	-	lincRNA	784	chr8	95565886	95582871	+	2	1.01528	1.34032	1.14024	0.65908	0.847974	0.724902	1.01423	1.67126	1.32408
ENST00000523011.1	ENSG00000253704.1	RP11-267M23.4	lincRNA	1789	chr8	95565950	95581973	+	2	1.3598	0.89035	1.29824	0.241735	0.619466	0.75273	1.43464	1.064	0.679793
TCONS_00285931	XLOC_023891	-	lincRNA	703	chr8	95969851	95988190	+	2	0.20227	1.41683	0.84278	2.16923	1.10934	0.561495	0.472628	1.4591	0.810145
TCONS_00284086	XLOC_024404	-	intronic_lncRNA	1241	chr8	99675590	99680924	-	2	2.93848	2.11033	2.1521	2.27685	2.96046	4.12637	1.74769	2.48254	2.33528
ENST00000521696.1	ENSG00000253948.1	RP11-410L14.2	lincRNA	380	chr8	100008991	100025272	-	2	1.68188	2.82333	2.83381	2.51139	1.40509	1.18699	3.79114	4.21285	0.659376
TCONS_00282273	XLOC_023911	-	intronic_lncRNA	2026	chr8	100595503	100598929	+	3	3.12985	1.31655	2.8269	1.68262	2.45834	2.69662	2.04669	2.46719	1.86592
TCONS_00285606	XLOC_024419	-	anti-sense_lncRNA	1820	chr8	102503009	102505017	-	2	7.49554	4.71556	6.63517	1.06697	5.27827	2.73606	4.00449	3.42872	3.10252
TCONS_00287304	XLOC_023924	-	lincRNA	306	chr8	103668748	103678469	+	2	0	0	0	0	1.20623	0	0	0	0
ENST00000517910.1	ENSG00000253669.3	KB-1732A1.1	lincRNA	2770	chr8	103819050	103822199	+	2	0.35751	0.4673	0.75892	0.896367	0.478107	0.62927	0.657662	0.531357	0.516362
TCONS_00284500	XLOC_023927	-	lincRNA	2139	chr8	103979360	104004705	+	3	3.78983	1.89587	2.1579	1.25437	1.90245	1.85003	1.65573	2.1568	1.31021
TCONS_00283374	XLOC_024427	-	intronic_lncRNA	620	chr8	104169240	104178645	-	2	2.17836	2.21071	3.54508	1.07052	1.92338	1.68287	0.995371	0.584661	2.36151
TCONS_00285627	XLOC_024427	-	anti-sense_lncRNA	2886	chr8	104256739	104311223	-	3	2.50863	1.73573	2.593	1.42938	2.17272	1.83238	2.29958	2.36224	2.89966
TCONS_00285628	XLOC_024427	-	lincRNA	1891	chr8	104278311	104281071	-	2	3.62079	1.75658	2.88869	1.02212	1.52768	1.83054	2.28036	2.76043	3.0743
TCONS_00284770	XLOC_024434	-	anti-sense_lncRNA	1865	chr8	107531171	107535193	-	2	0.4903	0.50974	0.62872	0.307585	1.03441	0.676063	0.526415	0.870138	0.451023
TCONS_00289991	XLOC_024443	-	lincRNA	1259	chr8	110246395	110247726	-	2	2.98492	1.73971	1.58641	1.0888	2.03691	1.79531	2.38253	1.06749	1.6942
TCONS_00281764	XLOC_024456	-	lincRNA	899	chr8	117271767	117272980	-	2	1.60299	0.10164	0.40231	0.458629	0.265246	0.302598	0.422222	1.27654	0.664188
TCONS_00285961	XLOC_023957	-	lincRNA	359	chr8	117466370	117480195	+	3	2.53336	1.37397	1.38255	0	0.39901	0.896941	1.17974	0.53302	2.6212
TCONS_00281771	XLOC_024462	-	lincRNA	2633	chr8	118722530	118725913	-	4	2.10557	0.62431	1.0309	0.420836	0.984929	0.549134	0.71263	0.930659	0.609476
TCONS_00291052	XLOC_024462	-	lincRNA	451	chr8	118725048	118726036	-	3	0.73369	0.71796	0.57303	0	0	0.28275	0.305544	1.59498	0.305517
TCONS_00289156	XLOC_023963	-	lincRNA	1686	chr8	119915695	119918585	+	2	0.6866	0.23807	0.61092	0.215427	1.49078	2.88851	0.472275	0.650409	0.622222
TCONS_00284520	XLOC_023963	-	lincRNA	637	chr8	120049799	120050886	+	2	0.23267	0.16335	0.32422	0.440871	0.852726	1.13191	0.273016	0.374281	0.266878
TCONS_00283398	XLOC_024472	-	lincRNA	399	chr8	121084531	121092871	-	3	0	1.45571	0.36461	0.324281	0	0	0.619743	0	0
TCONS_00282716	XLOC_024475	-	lincRNA	731	chr8	122381396	122397197	-	2	2.10812	0.4024	1.86105	0.483398	0.466762	0	1.11782	1.68763	0.328703
TCONS_00284793	XLOC_024483	-	lincRNA	600	chr8	124868806	124870711	-	2	0	1.60802	1.06466	0.321229	0.155456	0.353513	0.897153	1.22918	0.29191
ENST00000533496.1	ENSG00000255080.1	RP11-1082L8.3	lincRNA	553	chr8	125847126	125869758	-	3	0.5754	0	0	0.364228	1.58842	0.400929	0.680526	0	0
TCONS_00284534	XLOC_023983	-	lincRNA	315	chr8	125910684	125923948	+	2	0.86741	0	2.58313	0	0	0.620469	1.10954	0	0
TCONS_00288601	XLOC_023987	-	lincRNA	342	chr8	126660682	126692442	+	3	0	1.02823	0.51865	0.455261	0.896193	2.01031	0	0.600029	2.10237
TCONS_00284537	XLOC_023990	-	lincRNA	860	chr8	127878079	128018965	+	4	0.30862	0.44944	2.23859	0.194323	0.749482	0.510706	0.179054	0.492019	0.43985
TCONS_00281325	XLOC_023990	-	lincRNA	387	chr8	127889731	127938644	+	2	1.61982	2.26008	0	0.690496	0.337776	0.845274	0	0.449785	1.65E-05
TCONS_00281811	XLOC_024497	-	lincRNA	444	chr8	128302004	128307041	-	2	1.66026	2.36341	0.88509	1.32038	0.771479	1.16377	0.249966	0.681817	1.44837
TCONS_00288789	XLOC_024500	-	lincRNA	753	chr8	128926548	128939069	-	2	0.3681	0.77247	0.12756	2.08846	0.672001	1.40384	0.643414	0.588851	0.21033
TCONS_00283098	XLOC_023995	-	lincRNA	5995	chr8	129009454	129027277	+	3	2.74951	2.4231	3.32332	3.49901	4.17193	7.25179	2.24696	3.8181	5.14132
TCONS_00291072	XLOC_024511	-	lincRNA	449	chr8	132855796	132897118	-	3	0	0.57874	0.5778	0	0.251881	0	0	1.66905	0.945771
TCONS_00281834	XLOC_024516	-	lincRNA	1085	chr8	133696457	133699897	-	2	1.15173	0.1603	0.31691	0.724068	1.11542	0.318418	0.0664519	0.913998	0.196394
TCONS_00282750	XLOC_024517	-	intronic_lncRNA	1733	chr8	134139147	134141605	-	2	2.39608	1.01529	1.45743	0.96055	0.561911	0.367193	0.381406	0.840466	0.942312
TCONS_00284812	XLOC_024520	-	lincRNA	254	chr8	134375500	134376852	-	2	0	0	0	0	0	3.73658	0	0	0
TCONS_00281341	XLOC_024012	-	lincRNA	1466	chr8	137501561	137549927	+	5	2.04894	0.1879	1.76999	6.49E-05	0.567274	1.08618	0.692508	0.605854	1.37904
TCONS_00290383	XLOC_024012	-	lincRNA	2601	chr8	137501672	137533582	+	3	2.4175	0.58972	1.17459	0	0.622729	0.594191	1.49554	0.558064	1.14171
ENST00000523232.1	ENSG00000253839.1	RP11-431D12.1	lincRNA	564	chr8	137501691	137542119	+	4	5.59426	2.87073	4.00004	0.988276	1.19812	1.58902	3.00514	1.13274	1.17336
TCONS_00283116	XLOC_024012	-	lincRNA	1622	chr8	137527832	137549891	+	4	0.47808	0.99526	0	0.243237	2.30E-05	0	0.625677	0.271464	0
ENST00000520885.1	ENSG00000253210.1	RP11-809O17.1	lincRNA	483	chr8	142136143	142138220	-	2	1.07496	1.77769	0.75935	5.68637	1.54716	4.25846	2.7826	4.38592	4.35722
TCONS_00290030	XLOC_024543	-	lincRNA	824	chr8	143262067	143264093	-	2	0.65273	0.3419	0.45135	0	0.19828	0.33912	1.04273	0.260418	0.279275
TCONS_00283138	XLOC_024047	-	lincRNA	341	chr8	144871466	144872038	+	2	0	0.51783	0.52249	0.458492	1.35406	0	0	0	0.423526
TCONS_00288632	XLOC_024063	-	intronic_lncRNA	294	chr8	145229813	145230458	+	2	2.09842	1.55536	0.79339	1.36636	2.03907	3.77939	1.36918	4.59379	0.637322
TCONS_00288840	XLOC_024583	-	intronic_lncRNA	401	chr8	145628265	145630837	-	2	0.50281	0.36022	0	0.642062	1.56826	0.707807	0	2.50268	0.588795
TCONS_00300901	XLOC_024616	-	lincRNA	556	chr9	1460223	1476858	+	4	0.28529	0	0.19993	0	0.680647	1.1027	0.168648	1.15429	0
TCONS_00296511	XLOC_024616	-	lincRNA	385	chr9	1475367	1476631	+	2	0.54569	0	0	0.349007	1.40372	0.943743	0	0	1.60273
TCONS_00295272	XLOC_025154	-	lincRNA	2524	chr9	2450964	2493570	-	2	1.71306	1.09458	2.03894	0.220295	0.528839	0.151294	0.526911	0.760719	0.347657
TCONS_00296862	XLOC_025155	-	lincRNA	608	chr9	2502913	2506535	-	2	1.74385	2.27654	2.7822	0.314902	0.457095	0.346537	0.29301	0.200752	0.143054
TCONS_00298088	XLOC_024620	-	lincRNA	2686	chr9	3602523	3610232	+	3	3.94222	1.53464	2.71828	1.69891	3.55895	3.84676	2.3448	1.74508	2.52998
TCONS_00297235	XLOC_024620	-	lincRNA	7634	chr9	3645341	3653842	+	2	4.72837	2.28107	3.18066	2.00689	4.00433	3.95396	2.75819	2.39487	2.91096
TCONS_00295698	XLOC_024620	-	lincRNA	2851	chr9	3647731	3653850	+	2	4.0774	0.86949	2.80592	1.07996	3.3547	2.27269	2.26645	1.84331	1.66293
ENST00000457566.1	ENSG00000232104.2	RP11-509J21.1	lincRNA	496	chr9	3668086	3671646	+	2	10.2312	5.75116	7.40449	3.04365	9.21574	5.89385	8.78309	3.77611	4.88444
TCONS_00292647	XLOC_024620	-	lincRNA	11328	chr9	3669500	3683592	+	2	5.67459	1.85258	3.29871	1.37932	4.7584	3.44971	3.99219	3.12034	2.53307
TCONS_00292649	XLOC_024621	-	lincRNA	1842	chr9	3734629	3738992	+	2	3.0451	0.60306	1.06263	0.194937	1.23627	0.728395	1.17445	0.73533	0.809179
TCONS_00296519	XLOC_024623	-	lincRNA	241	chr9	3800757	3801599	+	2	0	0	1.66788	0	0	0	0	0	1.31663
TCONS_00293232	XLOC_025159	-	lincRNA	604	chr9	4347111	4348762	-	2	2.0126	0.35375	0.52694	0.159017	1.07727	0.699981	0	0.405573	0
TCONS_00301158	XLOC_025162	-	lincRNA	406	chr9	4676470	4679434	-	2	0.49075	1.40507	0.35172	0.626362	1.22338	2.07133	0.597497	2.8459	0.574148
ENST00000607997.1	ENSG00000273061.1	RP11-6J24.6	lincRNA	463	chr9	4678819	4679502	-	2	3.0827	0.82097	3.54912	0.734612	1.19095	0.539456	1.84863	1.26167	1.11798
TCONS_00302402	XLOC_025170	-	anti-sense_lncRNA	1057	chr9	5877269	5911432	-	3	1.69848	1.34351	1.48884	1.22558	1.46796	1.87464	1.15107	0.859133	1.86476

TCONS_00302403	XLOC_025170	-	anti-sense_lncRNA	1930	chr9	5898144	5911432	-	2	2.81479	2.68856	2.57352	2.68673	4.2879	3.17984	1.62869	2.87931	2.98547
TCONS_00295288	XLOC_025176	-	lincRNA	2917	chr9	11088612	11194546	-	4	1.35207	2.06124	0.41005	0.347151	0.495165	0.568075	0.842706	0.186568	0.593101
TCONS_00294386	XLOC_025176	-	lincRNA	2795	chr9	11162218	11194402	-	3	5.50772	1.49768	1.92389	0.390411	0.643236	0.7096	1.29171	0.652816	0.934786
TCONS_00299811	XLOC_025176	-	lincRNA	418	chr9	11172061	11194381	-	2	7.89778	4.87331	5.41508	0.718024	0.556731	1.25819	2.02323	1.76246	1.90274
TCONS_00302412	XLOC_025184	-	lincRNA	369	chr9	13432439	13433213	-	2	0.59652	0.86051	0	3.82277	0.749558	0	1.10561	1.5001	1.0552
TCONS_00299817	XLOC_025185	-	lincRNA	288	chr9	13939889	13942844	-	3	0	0	0.84753	0	0.725074	3.22092	0.732346	0.981594	0.679769
TCONS_00296888	XLOC_025185	-	lincRNA	515	chr9	13994010	14018321	-	4	2.2552	0.91087	2.49351	1.22528	1.38717	0	1.34021	0.523617	0.186039
ENST00000416996.1	ENSG00000225472.1	RP11-120J1.1	intronic_lncRNA	964	chr9	14317084	14357853	+	2	2.26717	1.20814	2.20687	0.672168	1.5359	1.56834	2.31435	1.69686	1.21453
TCONS_00294876	XLOC_024646	-	intronic_lncRNA	1492	chr9	15885366	15886958	+	2	2.84227	0.9315	1.62295	0.743464	0.905462	1.30745	0.770464	0.998374	1.02933
TCONS_00295727	XLOC_024647	-	lincRNA	513	chr9	16035406	16060801	+	4	0.97265	1.60438	0.22817	0.411052	0.398913	0.905175	0.19273	0.790596	0.187249
TCONS_00299826	XLOC_025193	-	intronic_lncRNA	6161	chr9	16682057	16689147	-	2	5.14033	3.43272	3.09068	4.13121	3.0057	2.56998	2.36953	2.46096	1.96247
TCONS_00293958	XLOC_024659	-	lincRNA	1061	chr9	22653261	22654695	+	2	1.89401	0.1648	0.32583	0.223289	0.0716696	0.327321	0.204991	0.563852	0.134602
TCONS_00295309	XLOC_025212	-	lincRNA	1099	chr9	23659436	23663682	-	2	1.02046	1.02561	0.46789	0.213831	2.05863	1.72395	0.850238	0.539769	0.386633
TCONS_00299833	XLOC_025212	-	lincRNA	3109	chr9	23671120	23675643	-	2	1.15823	0.72837	1.00532	0.373925	1.43576	1.93347	0.800969	0.579645	0.277609
TCONS_00292709	XLOC_024669	-	lincRNA	514	chr9	27701996	27703296	+	2	1.61595	1.82764	0.22743	0	0.39762	0.225567	0	0.262668	0
TCONS_00297708	XLOC_025227	-	anti-sense_lncRNA	1643	chr9	32449996	32452924	-	2	2.33318	0.98077	1.7425	1.0649	1.32213	1.60929	0.567545	0.669917	2.00261
TCONS_00293308	XLOC_025228	-	lincRNA	1831	chr9	32885457	32891890	-	2	1.18871	0.52041	0.77028	0.392506	0.98062	0.90586	1.2541	0.592254	0.850075
TCONS_00298122	XLOC_024682	-	lincRNA	402	chr9	33603363	33605224	+	2	1.0007	1.07518	0	0.31943	0.624117	0.704265	0.305006	0	1.46452
TCONS_00302451	XLOC_025260	-	lincRNA	403	chr9	35592753	35605117	-	2	0.99583	0.71317	1.78564	0	0.931445	0.350379	0.910251	0	0.582848
ENST00000574939.1	ENSG00000227388.2	RP11-112J3.16	lincRNA	546	chr9	35772160	35780595	+	2	1.76092	0.62099	0.4117	0.371637	0.540361	1.02275	0.521089	1.42628	0.507308
TCONS_00294490	XLOC_025290	-	lincRNA	672	chr9	40724860	40750172	-	3	10.8326	7.96769	5.67374	0.272032	2.14918	1.19715	3.59367	1.44441	5.31471
TCONS_00293389	XLOC_025290	-	lincRNA	1407	chr9	40729404	40750347	-	2	1.32933	0.19172	0.18195	0	0.0842333	0	0.0731125	0.110269	0.620091
TCONS_00296184	XLOC_025292	-	lincRNA	329	chr9	41948714	41954668	-	2	3.10631	2.26762	4.01279	2.00421	1.97733	4.42707	4.42124	3.31676	0.927602
TCONS_00296186	XLOC_025308	-	lincRNA	390	chr9	67332461	67340114	-	4	3.18919	4.19755	3.061	2.03855	2.65841	1.87311	2.92894	2.21182	1.87136
TCONS_00294953	XLOC_024750	-	anti-sense_lncRNA	6915	chr9	68745857	68754821	+	2	3.95488	3.48825	2.55686	1.66691	1.74511	1.24529	1.60969	1.56359	3.09772
TCONS_00297345	XLOC_024751	-	lincRNA	886	chr9	69450012	69451050	+	2	1.03936	0.93227	1.23009	0.84122	0.18021	0.925062	0.25822	0.473115	0.423048
TCONS_00296190	XLOC_025321	-	lincRNA	1969	chr9	70502782	70505072	-	2	5.1343	1.71887	3.31283	1.33886	2.22492	3.77779	1.3537	2.72904	2.93832
TCONS_00302202	XLOC_024756	-	lincRNA	1709	chr9	70920915	70927789	+	2	1.21706	0.75018	0	0.0848557	0.0407769	0.0466297	0.348764	0.106739	0.344646
TCONS_00300585	XLOC_025325	-	intronic_lncRNA	1603	chr9	71568724	71590858	-	3	0.9453	1.5637	0.94604	2.09933	1.53546	1.75557	1.41796	1.14857	0.90637
TCONS_00297361	XLOC_024773	-	lincRNA	1955	chr9	74954945	74960941	+	2	3.7215	0.80587	2.66356	1.82372	3.18888	2.28478	0.798772	0.596034	0.954369
TCONS_00302212	XLOC_024774	-	lincRNA	456	chr9	75093177	75094501	+	2	0.39578	0.2813	0.56146	0	0	0.277175	0.950787	0.324354	0.22984
TCONS_00295395	XLOC_025338	-	intronic_lncRNA	958	chr9	75138421	75143020	-	3	1.88182	1.21777	1.11188	0.507411	0.325927	0.371951	1.08872	0.427616	1.30073
TCONS_00302479	XLOC_025340	-	lincRNA	1300	chr9	75485873	75487967	-	2	2.87388	3.86434	2.48106	0.524073	2.01661	2.56034	1.17303	1.39427	0.420836
TCONS_00301236	XLOC_025348	-	intronic_lncRNA	510	chr9	78833517	78834173	-	2	0	0.46291	0	0	0.402844	1.14251	0.194669	0.26616	0
TCONS_00295823	XLOC_024785	-	lincRNA	1404	chr9	79131018	79179942	+	3	0.79437	1.2103	1.41997	1.81767	1.01996	1.74146	0.469697	0.859061	1.73936
TCONS_00294066	XLOC_024785	-	lincRNA	832	chr9	79167592	79179568	+	2	1.55045	0.83514	1.064	0.895912	1.18052	1.69803	0.126213	0.793986	0.991926
TCONS_00299614	XLOC_024794	-	lincRNA	7010	chr9	83232730	83260278	+	3	0.29998	0.14504	0.4287	0.422653	0.360366	0.784215	0.2304	0.812064	2.18297
TCONS_00292835	XLOC_024796	-	lincRNA	612	chr9	84304754	84354258	+	2	3.20729	0.69358	0.86081	0.935491	1.65952	1.02945	1.88552	0.993792	0.708231
TCONS_00300995	XLOC_024796	-	lincRNA	547	chr9	84354245	84363401	+	2	0.58529	0	0.20524	0.37056	0.897963	0.61187	0.519534	2.13308	0.168608
TCONS_00295412	XLOC_025360	-	lincRNA	277	chr9	85733097	85834149	-	3	0	3.76427	0	5.76815	4.12118	0.912821	0.837145	0	6.95231
TCONS_00299616	XLOC_024801	-	lincRNA	2918	chr9	85844535	85847512	+	2	3.00499	1.01356	2.28051	0.423762	1.53686	0.95713	0.45019	1.06374	0.955149
TCONS_00296651	XLOC_024802	-	intronic_lncRNA	689	chr9	85902164	85903189	+	2	1.04018	0.29157	2.16837	0.262539	0.634153	0.288828	0.729751	0.333708	0.357259
TCONS_00295430	XLOC_025373	-	lincRNA	1251	chr9	88416541	88418399	-	2	1.94039	0.80909	1.13233	0	0.527786	0.402001	1.34003	0.99885	0.33042
TCONS_00299625	XLOC_024811	-	lincRNA	4367	chr9	89599938	89605490	+	2	2.76934	1.21966	2.42074	0.767611	1.88559	2.10844	1.17276	1.27053	0.304368
ENST00000425666.1	ENSG00000231107.1	RP11-389K14.3	lincRNA	1013	chr9	93063184	93195771	-	3	0.37604	0.92944	1.02357	0.315336	0.907511	1.0318	0.252163	0.49794	1.28E-06
TCONS_00293478	XLOC_025389	-	lincRNA	592	chr9	93145530	93195830	-	2	0.7777	0.79382	0.56919	0.163908	0.642343	0.378103	1.30052	0.836579	0.148974
ENST00000445280.1	ENSG00000227603.1	RP11-165J3.6	lincRNA	691	chr9	96197614	96199403	-	2	1.65754	1.45185	1.58354	0.392198	1.642	0.431468	1.33229	1.16313	2.13472
TCONS_00299643	XLOC_024848	-	lincRNA	232	chr9	96738680	96742734	+	3	0	0	0	0	1.66909	0	0	0	0
TCONS_00301283	XLOC_025448	-	lincRNA	621	chr9	102567720	102582169	-	2	7.24403	1.01784	1.51563	0.915403	2.36154	2.68616	1.84403	1.74972	0.692889
TCONS_00299956	XLOC_025449	-	lincRNA	1133	chr9	102647893	102657209	-	2	1.63892	0.38001	0.82623	0.274697	1.3221	0.528483	0.440919	1.47305	0.682841
TCONS_00295485	XLOC_025464	-	lincRNA	281	chr9	106723029	106740798	-	3	1.20213	7.17876	0	0.786344	0	0	1.59224	0	0
TCONS_00296316	XLOC_025465	-	lincRNA	1827	chr9	106839583	106854799	-	4	1.18684	1.76131	0.71288	0.0313825	0.260358	0.238645	1.16207	0.036873	0
TCONS_00293562	XLOC_025465	-	lincRNA	11650	chr9	106839619	106856263	-	3	4.49733	2.70713	2.76914	0.319506	0.830863	0.70861	1.10383	0.568447	0.865277
TCONS_00298531	XLOC_025468	-	lincRNA	299	chr9	109880371	109884051	-	2	0	0	0	1.29986	0	0.718865	0.648918	0	0.605521
TCONS_00299961	XLOC_025470	-	lincRNA	211	chr9	109951886	109953368	-	2	0	3.00169	0	0	2.69802	0	0	3.7552	2.51662
TCONS_00295084	XLOC_024896	-	lincRNA	787	chr9	109984462	109991512	+	2	3.05795	2.66681	5.64271	0.218567	1.37085	0.120197	1.10984	1.10839	1.2872
TCONS_00292943	XLOC_024896	-	lincRNA	682	chr9	109991243	109994429	+	2	2.8212	0.44378	1.90705	0.532733	0.514778	0.146523	0	0.507975	0.845852
TCONS_00301976	XLOC_025474	-	lincRNA	2606	chr9	111075422	111402389	-	3	0.78896	0.61986	1.23281	0.420011	1.39794	0.500323	1.18871	1.16333	2.68503
TCONS_00294628	XLOC_025474	-	lincRNA	902	chr9	111137336	111402482	-	2	1.21965	1.20357	2.84688	0.475726	0.99142	0.590668	2.60167	2.6766	4.79881
TCONS_00301303	XLOC_025491	-	lincRNA	460	chr9	113875831	114047167	-	3	0	0.8804	2.53889	3.19118	1.55684	5.31435	1.96369	3.38649	11.1872
TCONS_00297081	XLOC_025491	-	lincRNA	9630	chr9	114025958	114047241	-	2	0.41164	0.48486	0.47019	0.543377	0.367768	1.23331	0.367123	0.759454	2.48969

TCONS_00297087	XLOC_025498	-	lincRNA	1159	chr9	114617179	114658794	-	3	0.74414	0.44358	0.94974	0.0668086	0.385813	0.0734452	1.10266	0.421389	0.301919
TCONS_00296722	XLOC_024916	-	intronic_lncRNA	345	chr9	115642635	115643812	+	2	0	0	2.0298	0.445831	0.438596	0.984238	0.8676	0	0
TCONS_00297106	XLOC_025520	-	intronic_lncRNA	4979	chr9	117177598	117183477	-	3	4.01764	1.77216	1.97919	1.1913	1.90068	2.25006	1.20473	1.59414	1.85742
TCONS_00293640	XLOC_025525	-	intronic_lncRNA	6270	chr9	118010811	118025078	-	2	4.91344	3.67326	3.51614	0.569034	1.10183	1.15771	3.33235	0.871597	1.79429
TCONS_00292982	XLOC_024939	-	intronic_lncRNA	5428	chr9	119475169	119480930	+	2	2.18802	1.09336	0.98427	0.624011	1.04471	1.12918	0.923084	1.1656	0.881936
TCONS_00293644	XLOC_025531	-	lincRNA	425	chr9	120562975	120576039	-	2	0.89951	0.64189	0.6418	0.286546	0	0	0.272201	2.22519	0.262267
TCONS_00297114	XLOC_025532	-	lincRNA	2770	chr9	120630916	120708728	-	4	0.73299	0.3246	1.58921	7.79E-06	0.346089	0.205395	0.637055	0.590926	1.20823
TCONS_00293645	XLOC_025532	-	lincRNA	3165	chr9	120630964	120638859	-	2	1.5099	0.39401	2.19247	0.1607	1.61749	1.25518	1.53956	1.35792	1.98786
TCONS_00301322	XLOC_025532	-	lincRNA	1143	chr9	120631813	120708076	-	3	0.40006	0.0489	0.00015	0.105516	0.486433	1.31143	0.0335041	0.33841	0
TCONS_00301058	XLOC_024947	-	lincRNA	885	chr9	123970974	123973150	+	3	0.42745	0.42241	0.5824	0	0	0	0.531092	0.664881	0.445989
TCONS_00295114	XLOC_024947	-	lincRNA	1271	chr9	123971297	124028369	+	6	1.14749	0.75324	0.54993	0.293781	1.28901	0.283977	0.218206	0.308422	0.535213
TCONS_00298260	XLOC_024947	-	lincRNA	1147	chr9	123971350	123994085	+	4	0.86909	0.78935	0.95539	1.08847	0.494842	0.94317	0.425358	1.22024	0.417794
TCONS_00297992	XLOC_025549	-	lincRNA	513	chr9	124241556	124262384	-	2	1.29686	2.52117	2.73807	2.05526	1.19674	3.16811	3.8546	2.37179	4.49397
TCONS_00297546	XLOC_024956	-	intronic_lncRNA	4078	chr9	126243680	126256656	+	4	0.59892	0.48581	0.74955	0.142909	0.0113665	0	0.616771	0.565413	0.153994
TCONS_00294239	XLOC_024956	-	intronic_lncRNA	4629	chr9	126243680	126256656	+	3	0.6682	0.6441	0.99964	0	0.655906	0.143313	0.880641	0.202847	0.272213
TCONS_00296743	XLOC_024956	-	intronic_lncRNA	1933	chr9	126243776	126252047	+	2	1.03188	0.48545	0.77248	0.119649	0.939259	0.137083	1.76495	0.522572	0.807552
TCONS_00304412	XLOC_024956	-	intronic_lncRNA	4737	chr9	126249600	126256345	+	2	0.60222	0	0.12857	0.0577776	0.101291	0.117598	0.160012	0.498764	0.603468
TCONS_00300015	XLOC_025567	-	lincRNA	245	chr9	127614922	127615528	-	2	0	0	0	0	1.31175	1.43834	0	0	0
ENST00000454034.1	ENSG00000233721.1	RP11-205K6.1	lincRNA	719	chr9	129278696	129281087	+	2	0.78424	0.27454	2.31299	1.23654	1.07477	0.680123	2.0597	0.157033	0.224257
ENST00000443631.1	ENSG00000223478.1	RP11-545E17.3	anti-sense_lncRNA	544	chr9	131486724	131495473	+	2	1.47591	1.2493	2.0708	1.86904	3.98607	3.29198	2.79588	1.4348	3.23194
TCONS_00293093	XLOC_025010	-	lincRNA	1315	chr9	132044621	132046172	+	2	0.28553	1.29704	0.34608	0.262518	0.681356	1.12309	0.13041	0.531803	0.402023
ENST00000455981.1	ENSG00000224307.1	RP11-344B5.2	lincRNA	583	chr9	132044737	132048007	+	3	1.02885	2.53656	2.68703	2.42108	2.22081	2.07079	3.05254	2.49593	3.54672
TCONS_00297590	XLOC_025012	-	lincRNA	1357	chr9	132094568	132097425	+	2	1.23406	0.61218	1.0278	0.221428	1.11812	0.365088	0.506652	0.418429	0.450004
TCONS_00300042	XLOC_025622	-	intronic_lncRNA	358	chr9	135555740	135558527	-	2	0.63727	0.92191	0.4639	0	1.20481	0	0.791868	0.536555	0.753778
TCONS_00293773	XLOC_025628	-	lincRNA	968	chr9	136108483	136113169	-	2	1.19403	1.10936	2.2859	0.166929	0.723716	0.917729	1.53473	1.47693	1.13265
TCONS_00295615	XLOC_025629	-	lincRNA	3213	chr9	136125795	136130332	-	2	2.23656	1.1251	1.8255	0.998087	4.62715	5.25002	1.12116	1.62543	1.34005
TCONS_00296839	XLOC_025091	-	lincRNA	279	chr9	138026373	138027351	+	2	0	0	1.88482	0	0	0	0	0	0
TCONS_00293789	XLOC_025643	-	lincRNA	484	chr9	138144291	138147251	-	2	4.99894	1.26522	1.26099	0.453298	0.220229	0	0.21326	0.291332	0.620226
TCONS_00295239	XLOC_025100	-	lincRNA	1779	chr9	138799730	138805425	+	2	4.13575	3.18082	3.31579	1.29743	2.45465	1.64869	3.10972	2.49849	1.75634
ENST00000563018.1	ENSG00000260193.1	RP11-83N9.5	lincRNA	775	chr9	138999951	139001270	-	2	0	0	0.2452	0.111572	0.215345	0.245433	0.515225	0.424463	0.202204
TCONS_00298639	XLOC_025661	-	lincRNA	889	chr9	139535694	139538924	-	2	0.44351	0.72191	0.71439	1.48897	0.448547	0.716348	1.0283	0.35327	0.0842383
TCONS_00295245	XLOC_025110	-	intronic_lncRNA	487	chr9	139703713	139704457	+	2	1.06009	2.5035	1.2474	1.12132	1.52518	0.987903	1.05468	1.72943	1.22724
TCONS_00293219	XLOC_025145	-	lincRNA	1730	chr9	141067697	141069916	+	2	3.40107	1.47968	1.27777	0.125534	0.522797	1.74757	0.573232	0.894753	1.01969
TCONS_00307366	XLOC_026145	-	lincRNA	384	chrX	2737281	2742413	-	2	1.64585	1.5774	0	0	0.343433	0	0.336702	0.914912	0.322334
TCONS_00307372	XLOC_026151	-	lincRNA	2870	chrX	5635474	5644400	-	2	0.76474	1.13764	1.01735	1.74951	1.7026	1.6327	2.0297	1.35367	0.885925
TCONS_00307380	XLOC_026166	-	lincRNA	1014	chrX	11073565	11129329	-	3	0.86537	1.62335	1.16026	0.223429	1.10247	0.750283	0.104379	0.869341	0.782821
TCONS_00311476	XLOC_026166	-	lincRNA	438	chrX	11124911	11129240	-	2	0.0373	0.1154	0.76952	0.0439585	1.44101	1.59118	0.399509	0.790542	0.0018447
TCONS_00308264	XLOC_025757	-	intronic_lncRNA	245	chrX	19068926	19083860	+	2	11.6642	4.45928	6.21025	6.47289	1.31178	2.87675	2.71694	1.80065	0
TCONS_00309616	XLOC_026213	-	lincRNA	956	chrX	23768779	23784307	-	3	0.26954	0.46962	0.27871	1.01751	0.490193	0.839112	0.311898	0.750332	2.53168
TCONS_00305451	XLOC_025771	-	lincRNA	790	chrX	26275303	26344221	+	2	1.89807	0.36179	0	0	0	0	0	0.551331	0
TCONS_00305476	XLOC_025787	-	lincRNA	1485	chrX	38657745	38659715	+	2	1.58763	0.9366	0.21758	0	0.0958333	0.0547746	0.227853	0.313704	0.224991
TCONS_00311310	XLOC_025805	-	lincRNA	273	chrX	42992266	43024024	+	3	1.31826	0	0	0.865099	0.86655	2.87591	0	0	0
TCONS_00311934	XLOC_026241	-	lincRNA	590	chrX	45606352	45629700	-	3	0	0.46627	0	0.395526	0.232475	2.25441	0	0.562044	1.36726
TCONS_00309638	XLOC_026241	-	lincRNA	8138	chrX	45623977	45633259	-	2	4.68316	3.07394	3.67869	4.26917	4.48331	3.41279	1.67323	2.74028	2.93475
TCONS_00312338	XLOC_026241	-	lincRNA	5267	chrX	45624580	45630073	-	3	5.25219	4.04914	7.04768	10.933	6.74038	6.51508	4.30606	6.62492	4.87408
ENST00000602507.1	ENSG00000270069.1	RP6-99M1.2	lincRNA	1758	chrX	45627783	45629672	-	2	2.91389	4.39686	6.06279	10.6317	5.05021	3.35522	3.34517	6.0069	3.524
TCONS_00306033	XLOC_026241	-	lincRNA	834	chrX	45708333	45710220	-	2	1.5821	0.93128	1.89202	0.355904	2.02032	0.551456	0	0.707004	1.00384
TCONS_00306034	XLOC_026241	-	lincRNA	888	chrX	45708333	45711097	-	2	1.65072	2.75726	1.52897	1.90882	2.09257	1.54169	1.02982	1.70785	1.01591
TCONS_00311319	XLOC_025819	-	lincRNA	232	chrX	47225694	47229977	+	2	0	0	0	0	0	1.8212	0	0	0
ENST00000366185.2	ENSG00000232593.2	RP11-258C19.5	lincRNA	1063	chrX	53123339	53171889	+	3	8.59568	5.06855	7.02523	5.04945	4.79066	4.4902	2.9313	4.7273	4.23008
TCONS_00308928	XLOC_025856	-	lincRNA	626	chrX	53123345	53129082	+	3	2.43856	1.06318	2.75433	1.20615	1.31256	1.65902	0.700675	2.03189	0.958509
TCONS_00312984	XLOC_025864	-	lincRNA	8545	chrX	55934008	56048879	+	2	3.49101	1.82715	3.30112	2.17322	2.45555	2.6169	1.6716	3.37824	1.91621
ENST00000452532.1	ENSG00000227486.1	RP13-188A5.1	lincRNA	1007	chrX	55934556	56041606	+	2	2.87959	3.85656	3.44317	2.17993	3.13268	2.11358	2.47207	3.52841	1.46824
ENST00000439622.1	ENSG00000226310.1	RP3-323P24.3	lincRNA	665	chrX	57148095	57153676	+	2	2.33229	0.66135	2.09186	1.02027	1.14692	1.18419	1.78679	2.18563	0.674505
TCONS_00311344	XLOC_025868	-	lincRNA	3531	chrX	57148370	57157502	+	3	4.57781	1.35008	3.30681	1.43308	2.5699	3.5076	1.87101	2.78438	2.74613
TCONS_00305597	XLOC_025872	-	lincRNA	1336	chrX	64255016	64333837	+	5	3.26575	2.97701	4.55191	3.11241	1.6086	1.1505	6.66912	5.86744	6.60025
TCONS_00306553	XLOC_025872	-	lincRNA	2018	chrX	64270526	64333837	+	4	1.70041	0.65757	1.10042	0.275139	0.669777	0.734476	0.256405	1.47844	0.669533
TCONS_00308361	XLOC_025872	-	lincRNA	2425	chrX	64271089	64328062	+	4	2.76796	4.11388	2.61935	1.1375	1.22043	1.99921	0.670441	2.38047	2.16724
TCONS_00307183	XLOC_025872	-	lincRNA	2017	chrX	64271171	64589517	+	5	1.29168	1.54975	0.99	0.649476	1.23032	0.511088	0.360747	0.978302	0.81933
ENST00000424241.1	ENSG00000237311.1	RP6-159A1.3	lincRNA	857	chrX	65219593	65220913	+	2	1.08509	0.21639	0.74965	1.4641	1.88234	1.18074	0.449718	1.23575	0.176752
ENST00000450860.1	ENSG00000228427.1	RP5-1091N2.9	lincRNA	726	chrX	70403409	70418025	-	2	0.58038	1.21881	2.4159	3.53807	1.06031	1.07364	0.677215	1.70396	2.65489

ENST00000416330.1	ENSG00000229807.5	XIST	lincRNA	750	chrX	73040491	73046179	-	3	76.7966	46.9921	71.572	0.11666	0	0	0	62.4685	69.051
TCONS_00305647	XLOC_025911	-	lincRNA	1699	chrX	74822309	74826057	+	2	1.63356	0.236	0.46584	0.1822	0.328401	0.375531	0.524729	0.859647	0.308403
TCONS_00308392	XLOC_025911	-	lincRNA	2811	chrX	74824547	74847035	+	2	2.55928	0.64322	1.62732	0.50084	0.776462	0.780956	0.637234	0.799855	0.508236
TCONS_00309485	XLOC_025911	-	lincRNA	438	chrX	74824752	74875778	+	3	1.08316	0.97774	0	0.893101	0	0	0	0	0
TCONS_00307540	XLOC_026361	-	lincRNA	1259	chrX	75366767	75368854	-	2	0.57773	1.47207	0.5288	0.544399	0.349185	0.731421	0.277039	0.76249	0.765125
TCONS_00307219	XLOC_025922	-	lincRNA	554	chrX	80065554	80067972	+	2	0.57379	0.80898	0.60325	0.181597	1.0559	0.799577	0.848171	1.62538	0.991326
TCONS_00311370	XLOC_025923	-	lincRNA	17482	chrX	80566443	80587754	+	2	3.09762	1.94637	2.53597	1.64669	2.67185	2.66672	1.59551	2.24274	2.50269
TCONS_00306605	XLOC_025925	-	lincRNA	901	chrX	84057192	84093617	+	2	1.59843	0.40541	0.80229	0.54878	0.528967	3.21847	3.45217	2.5457	0.413924
TCONS_00306199	XLOC_026379	-	lincRNA	509	chrX	90669700	90673873	-	3	2.62706	3.25102	3.69937	1.24921	0.606258	1.37548	5.46907	1.60228	2.08688
ENST00000445990.1	ENSG00000234405.1	LL0XNC01-250H1	anti-sense_lncRNA	632	chrX	102752451	102755245	+	2	0.23535	0	0	0	0	0	0.276266	0	0.135003
TCONS_00313194	XLOC_026406	-	lincRNA	2588	chrX	102780032	102786934	-	4	0.89772	0.73971	0.93369	0.294781	1.15792	0.912858	0.708039	1.27862	0.724966
TCONS_00310224	XLOC_026407	-	lincRNA	1210	chrX	102809981	102811770	-	2	2.92753	1.68422	1.52538	1.07829	1.58696	1.74318	1.74385	1.03977	0.745132
TCONS_00309283	XLOC_026407	-	anti-sense_lncRNA	761	chrX	102825153	102840552	-	2	1.27006	1.01513	2.13707	0.343096	1.6558	0.880554	1.05673	0.145078	0.725551
TCONS_00307575	XLOC_026410	-	lincRNA	427	chrX	103049585	103050998	-	2	0	1.59028	0.31797	0.284002	0	0	0.539354	0.367466	0
TCONS_00306933	XLOC_026415	-	intronic_lncRNA	3459	chrX	103374549	103378631	-	2	4.71405	1.48308	3.35918	0.923489	2.23011	2.50037	1.59345	1.97449	2.11609
TCONS_00309730	XLOC_026415	-	intronic_lncRNA	1587	chrX	103374589	103389457	-	2	6.51241	7.22288	6.63493	6.73823	4.55524	5.02748	5.39229	5.91597	4.10175
TCONS_00308436	XLOC_025988	-	lincRNA	1097	chrX	107980749	107982394	+	2	0.79545	0.39535	1.40681	0.142872	0.618974	0.0785365	0.0655508	0.0901642	0
TCONS_00311400	XLOC_025997	-	lincRNA	280	chrX	112291384	112292121	+	2	0	0	2.79283	0	0	2.64295	0	2.15683	1.49004
TCONS_00311623	XLOC_026432	-	lincRNA	2466	chrX	113204617	113235173	-	2	0.18019	0.15593	0.03075	0.706056	4.93593	4.18963	0.15442	0.532003	0.0254698
ENST00000446986.1	ENSG00000224281.4	SLC25A5-AS1	lincRNA	1874	chrX	118599997	118602225	-	2	2.31642	1.14074	1.50078	0.535377	1.39627	1.51295	1.53579	2.21166	1.20774
TCONS_00309535	XLOC_026016	-	lincRNA	840	chrX	119125585	119127223	+	2	0.15912	0.66657	0.10998	0.60124	1.06303	0	0.184757	0.380716	0.362976
TCONS_00309746	XLOC_026449	-	lincRNA	581	chrX	119148775	119149563	-	2	0.26668	0.37542	0	2.69863	0.979952	1.85632	0	0.215334	0
TCONS_00313801	XLOC_026456	-	lincRNA	379	chrX	121219661	121220936	-	2	2.25485	1.21693	2.03596	0	0.353289	1.59159	0.693388	0.470831	2.32105
TCONS_00305795	XLOC_026030	-	lincRNA	976	chrX	126272090	126273599	+	2	1.70698	0.18297	0	0.165204	0	0	0.0759332	0.730759	0.0747261
TCONS_00305796	XLOC_026031	-	lincRNA	9171	chrX	128268222	128282288	+	2	2.51679	0.59898	1.83429	0.0926222	0.712377	0.477348	1.18802	0.672853	0.952324
TCONS_00306683	XLOC_026031	-	lincRNA	6832	chrX	128273706	128332861	+	3	3.83452	0.01939	1.38556	0.115813	0.858643	0.466779	1.57311	0.624349	1.04871
TCONS_00310719	XLOC_026472	-	lincRNA	410	chrX	128809923	128812442	-	2	0.96303	0.68884	0.34475	0.307171	1.19949	0.677152	1.17096	1.59395	1.12588
TCONS_00309544	XLOC_026038	-	lincRNA	312	chrX	129115120	129115770	+	2	0	0	0	2.305	0	0	0	0	0.535236
TCONS_00307620	XLOC_026482	-	lincRNA	412	chrX	130883159	130889709	-	2	0	2.38767	0.68274	1.21701	0.890918	2.68278	1.44913	1.57828	1.39375
TCONS_00307322	XLOC_026065	-	lincRNA	916	chrX	135991597	136007978	+	2	2.70326	2.18266	2.25765	1.16405	1.63969	1.77232	1.73051	1.81212	2.91733
TCONS_00306709	XLOC_026071	-	anti-sense_lncRNA	1866	chrX	139865127	139867176	+	2	4.65506	6.19815	1.5918	1.6907	0.627659	1.09794	0.140292	0.144935	0.416078
TCONS_00308797	XLOC_026515	-	lincRNA	1895	chrX	148615169	148617874	-	2	3.43164	4.42278	2.676	2.34155	1.92323	1.45267	1.27534	1.7093	1.97652
TCONS_00313241	XLOC_026517	-	intronic_lncRNA	2262	chrX	148686564	148688957	-	2	3.564	1.85104	2.06244	0.993271	2.92148	1.94418	2.00915	2.14454	1.84776
TCONS_00313672	XLOC_026080	-	lincRNA	1850	chrX	149165688	149168762	+	2	12.1833	6.12985	7.57134	2.21149	4.62276	4.86081	4.53321	5.02464	4.16625
TCONS_00309563	XLOC_026081	-	lincRNA	789	chrX	149281702	149282795	+	2	0.86422	0.96643	0.71786	0.435645	0	0.359361	0.402178	0.13807	1.08544
TCONS_00307338	XLOC_026091	-	intronic_lncRNA	322	chrX	151284687	151285187	+	2	0	1.19975	0	0.5296	0.523242	0	0	0	0.490894
TCONS_00307343	XLOC_026098	-	lincRNA	313	chrX	152201059	152204172	+	2	0	2.59204	0	1.14247	0	0	0	0.761161	1.59139
TCONS_00313247	XLOC_026529	-	lincRNA	382	chrX	152951862	152952405	-	2	0.55456	0	0	0	1.38924	0.391269	0	1.38814	0.65194
TCONS_00306401	XLOC_026549	-	lincRNA	695	chrX	153652624	153656823	-	2	5.13812	4.89595	6.71038	3.2418	3.50771	4.42229	9.12868	4.4494	5.52845
TCONS_00311691	XLOC_026562	-	lincRNA	1342	chrX	154581215	154585106	-	2	1.25024	0.2481	0.42881	0.504767	0.0539466	1.23298	0.0513378	0.494632	0.405283
TCONS_00313830	XLOC_026563	-	lincRNA	550	chrX	154644967	154686728	-	3	0.96585	0.78113	2.22607	1.13326	0.80602	1.20595	2.37398	0.0002017	2.04342
TCONS_00307691	XLOC_026563	-	lincRNA	847	chrX	154645220	154649838	-	2	0.78571	0.94388	1.31854	0.743499	0.409452	0.173836	0.290872	1.00399	1.00689
TCONS_00306419	XLOC_026563	-	lincRNA	748	chrX	154648506	154651247	-	2	0.68337	0.33679	1.40787	0.272731	0.473477	0.443169	0.539573	0	0.483522
ENST00000426699.1	ENSG00000229308.1	AC010084.1	lincRNA	559	chrY	3904538	3968361	+	2	0	1.3957	0	4.65541	1.04096	2.16799	6.35324	0	0
TCONS_00314327	XLOC_026577	-	lincRNA	346	chrY	7655401	7658396	+	2	0	0	0	2.21386	0	0	0.430637	0	0
TCONS_00314598	XLOC_026580	-	lincRNA	3111	chrY	14366587	14372642	+	2	0	0.48525	0	1.25289	1.39257	3.33294	4.06211	0	0
TCONS_00314292	XLOC_026588	-	lincRNA	2851	chrY	15978987	15999800	+	3	0	1.35728	0	1.46615	1.8337	2.00804	0.86102	0	0
TCONS_00314337	XLOC_026588	-	lincRNA	1225	chrY	15980822	15984550	+	2	0	0.76502	0	0.89117	0.834153	0.706961	0.505703	0	0
TCONS_00314606	XLOC_026588	-	lincRNA	1572	chrY	15999939	16020127	+	3	0	2.37297	0	2.24017	2.54906	2.41085	1.85873	0	0
TCONS_00314293	XLOC_026588	-	lincRNA	427	chrY	16014498	16020127	+	2	0	0	0	0	0.604826	0.312962	2.00223	0	0
TCONS_00314309	XLOC_026611	-	lincRNA	1578	chrY	21093648	21095740	-	2	0	2.87621	0	1.5334	2.32281	2.8089	0.891828	0	0
TCONS_00314614	XLOC_026613	-	lincRNA	3674	chrY	21202955	21207174	-	2	0	3.31285	0	2.50463	1.99723	2.54958	1.64211	0	0
TCONS_00314262	XLOC_026617	-	lincRNA	250	chrY	22539067	22539973	-	2	0	4.10865	0	1.19356	1.2067	0	1.24547	0	0
TCONS_00314342	XLOC_026594	-	lincRNA	646	chrY	23388378	23390599	+	2	0	0	0	2.04217	0	1.21E-05	0.266762	0	0
TCONS_00314343	XLOC_026594	-	lincRNA	671	chrY	23389752	23390599	+	2	0	0.15142	0	1.2017	0.263473	0.899772	0.0005595	0	0
TCONS_00000027	XLOC_000004	-	lincRNA	451	GL000192.1	511245	519419	+	2	0.40359	0.57403	1.14606	0	0.24983	0	0.24266	1.32421	1.17259
TCONS_00000087	XLOC_000020	-	lincRNA	656	GL000193.1	88632	92105	+	2	6.37795	1.6869	0.57928	0.281617	0.0002303	0.774626	1.73879	2.13949	0.511242
TCONS_00000088	XLOC_000020	-	lincRNA	620	GL000193.1	90077	92105	+	2	1.79156	0.20721	0.21408	0.305864	0.147702	0.168287	0.525105	1.18093	0.972385
TCONS_00000106	XLOC_000024	-	lincRNA	586	GL000195.1	174109	176856	+	2	3.94862	2.59338	6.62499	4.66112	2.90121	0.732844	5.27416	4.24952	5.2964
TCONS_00000123	XLOC_000026	-	lincRNA	2415	GL000198.1	69092	72108	+	2	2.99547	1.49953	0.94397	0.346717	0.277465	0.126999	0.789975	1.05232	0.625385
TCONS_00000172	XLOC_000042	-	lincRNA	1923	GL000214.1	121337	135553	-	2	11.0162	4.2686	1.70086	0.743049	0.856695	0.408294	2.2038	8.73381	1.60908

TCONS_00000176	XLOC_000038	-	lincRNA	284	GL000214.1	125633	131908	+	2	10.4697	4.33441	0	0.760092	0	0.841551	3.07049	15.4194	4.26624
TCONS_00000222	XLOC_000045	-	lincRNA	2791	GL000219.1	51516	55892	-	2	9.08632	5.14438	7.02166	5.4836	6.74197	3.86114	5.37692	2.85294	1.62442
TCONS_00000210	XLOC_000045	-	lincRNA	2002	GL000219.1	54017	56093	-	2	3.25046	2.83782	2.62535	2.62742	3.77645	1.8238	1.78099	0.668614	1.02721
TCONS_00000209	XLOC_000044	-	lincRNA	4800	GL000219.1	114470	121823	+	2	1.64292	0.8132	2.97869	0.31985	0.76054	0.336135	0.834491	0.401	0.162904
TCONS_00000272	XLOC_000056	-	lincRNA	696	GL000224.1	1255	14576	+	2	6.76871	5.60449	1.99474	1.03528	0.37506	0.711827	1.67812	2.30236	2.34774
TCONS_00000276	XLOC_000058	-	lincRNA	1132	GL000224.1	1272	15029	-	2	9.29717	3.80417	1.95503	0.824976	0.992645	0.302315	1.82865	4.51069	2.11288

Table S3. Differentially expressed lncRNA and mRNA transcripts for ECRSwNP *vs* CTRL.

Transcript ID	Gene ID	Gene name	Length	ECRSwNP FPKM	CTRL FPKM	log_2(foldchange)	P value	Q value
NM_022136.3	64092	SAMSN1	1888	74.1493	12.2431	2.59847	5.00E-05	0.00170056
NM_003248.4	7060	THBS4	3223	1.12346	10.474	-3.22079	5.00E-05	0.00170056
NM_001242699.1	387712	ENO4	2865	1.8675	5.50671	-1.56008	5.00E-05	0.00170056
NM_014685.2	9709	HERPUD1	2176	111.498	49.9015	1.15986	5.00E-05	0.00170056
NM_153614.2	374407	DNAJB13	1875	2.36469	8.23567	-1.80024	5.00E-05	0.00170056
NM_003364.2	7378	UPP1	1777	4.91896	1.01749	2.27333	5.00E-05	0.00170056
NM_003467.2	7852	CXCR4	1674	139.662	37.9932	1.87813	5.00E-05	0.00170056
NM_003608.3	8477	GPR65	4522	18.477	4.10697	2.16958	5.00E-05	0.00170056
NM_003670.2	8553	BHLHE40	3035	98.1131	23.8213	2.04219	5.00E-05	0.00170056
NM_001454.3	2302	FOXJ1	2641	19.355	60.2567	-1.63842	5.00E-05	0.00170056
NM_005097.2	9211	LGI1	2366	2.39658	0.473709	2.33890	5.00E-05	0.00170056
NM_001040272.5	92949	ADAMTSL1	7843	1.25487	5.358	-2.09416	5.00E-05	0.00170056
NM_182911.3	80705	TSGA10	3664	6.51924	20.1705	-1.62947	5.00E-05	0.00170056
NM_000789.3	1636	ACE	4969	6.38162	2.14659	1.57188	5.00E-05	0.00170056
NM_016206.2	389136	VGLL3	10396	20.62	6.58233	1.64737	5.00E-05	0.00170056
NM_021155.3	30835	CD209	4328	10.654	1.76931	2.59013	5.00E-05	0.00170056
NM_000527.4	3949	LDLR	5284	15.7748	5.65941	1.47889	5.00E-05	0.00170056
NM_001099772.1	1580	CYP4B1	2158	41.2942	128.138	-1.63369	5.00E-05	0.00170056
NM_145000.3	202151	RANBP3L	2543	0.511382	2.49278	-2.28528	5.00E-05	0.00170056
NM_194071.2	64764	CREB3L2	7455	33.1735	14.7643	1.16792	5.00E-05	0.00170056
NM_003974.2	9046	DOK2	1770	9.87414	3.05875	1.69072	5.00E-05	0.00170056
NM_130435.3	5791	PTPRE	5039	19.0631	5.41947	1.81456	5.00E-05	0.00170056
NM_018897.2	56171	DNAH7	12394	9.65151	44.9611	-2.21985	5.00E-05	0.00170056
NM_003407.2	7538	ZFP36	1745	45.3106	4.80738	3.23653	5.00E-05	0.00170056
NM_032131.4	84071	ARMC2	3413	6.61246	18.9244	-1.51698	5.00E-05	0.00170056
NM_178171.4	284110	GSDMA	2164	2.54552	0.312455	3.02624	5.00E-05	0.00170056
NM_033124.4	85478	CCDC65	1815	11.1135	31.3423	-1.49580	5.00E-05	0.00170056
NM_144980.3	168090	C6orf118	1839	6.11382	25.5535	-2.06337	5.00E-05	0.00170056
NM_000389.4	1026	CDKN1A	2159	27.6653	8.91486	1.63379	5.00E-05	0.00170056
NM_000395.2	1439	CSF2RB	4848	21.9423	3.70911	2.56457	5.00E-05	0.00170056
XM_001716834.2	642131	LOC642131	570	49.3887	11.6645	2.08206	5.00E-05	0.00170056
NM_033389.2	85464	SSH2	9166	15.5236	7.34737	1.07916	5.00E-05	0.00170056
NM_018286.2	55273	TMEM100	1755	11.2743	55.7012	-2.30467	5.00E-05	0.00170056
NM_001010855.2	146850	PIK3R6	3040	4.40492	1.10817	1.99093	5.00E-05	0.00170056
NM_033364.3	89876	C3orf15	4433	4.77248	23.3256	-2.28911	5.00E-05	0.00170056
NM_006142.3	2810	SFN	1315	29.6634	6.7159	2.14303	5.00E-05	0.00170056
NM_182906.2	10462	CLEC10A	1771	16.2476	3.39882	2.25712	5.00E-05	0.00170056
NM_003177.5	6850	SYK	5073	19.3682	8.23804	1.23332	5.00E-05	0.00170056
NM_001135217.1	10233	LRRC23	1800	15.7882	48.6042	-1.62224	5.00E-05	0.00170056
NM_023940.2	65997	RASL11B	1957	5.5833	19.4123	-1.79778	5.00E-05	0.00170056
NM_001135091.1	143662	MUC15	3392	7.76574	27.42	-1.82003	5.00E-05	0.00170056
ENST00000457157.2	ENSG00000231106.2	AP000688.8	1048	2.87908	27.6064	-3.26132	5.00E-05	0.00170056
NM_002737.2	5578	PRKCA	8770	11.6082	4.46167	1.37949	5.00E-05	0.00170056
NM_017596.2	23046	KIF21B	9895	5.5784	1.61248	1.79057	5.00E-05	0.00170056
NM_145172.3	126820	WDR63	3007	6.83313	31.7857	-2.21776	5.00E-05	0.00170056
NM_001657.2	374	AREG	1238	11.8994	0	inf	5.00E-05	0.00170056
NM_000291.3	5230	PGK1	2423	107.952	57.1127	0.91851	5.00E-05	0.00170056
NM_000275.2	4948	OCA2	3140	0.380531	1.91235	-2.32926	5.00E-05	0.00170056
NM_001945.2	1839	HBEGF	2358	20.1238	1.81423	3.47147	5.00E-05	0.00170056
NM_177401.4	90007	MIDN	3793	9.88113	3.72268	1.40834	5.00E-05	0.00170056
NM_001037339.1	5142	PDE4B	3876	20.0895	4.28492	2.22910	5.00E-05	0.00170056

NM_002104.2	3003	GZMK	1045	11.455	32.4814	-1.50364	5.00E-05	0.00170056
NM_001136103.2	92293	TMEM132C	4947	0.257211	5.482	-4.41368	5.00E-05	0.00170056
NM_002997.4	6382	SDC1	3201	64.9469	26.6973	1.28257	5.00E-05	0.00170056
NM_021928.3	60559	SPCS3	4596	88.1756	42.2901	1.06006	5.00E-05	0.00170056
NM_173543.2	199221	DZIP1L	3498	4.02872	10.6107	-1.39713	5.00E-05	0.00170056
NM_002961.2	6275	S100A4	512	254.659	102.697	1.31017	5.00E-05	0.00170056
NM_006933.4	6526	SLC5A3	11576	95.1134	28.0502	1.76164	5.00E-05	0.00170056
NM_030938.3	81671	VMP1	2176	117.521	42.9597	1.45186	5.00E-05	0.00170056
NM_001854.3	1301	COL11A1	7291	6.26187	32.4633	-2.37415	5.00E-05	0.00170056
NM_205855.3	389558	FAM180A	1801	0.482871	3.18214	-2.72029	5.00E-05	0.00170056
NM_001008781.2	120114	FAT3	19048	0.287291	5.86196	-4.35080	5.00E-05	0.00170056
TCONS_00081181	XLOC_006945	-	4801	2.54681	11.6812	-2.19742	5.00E-05	0.00170056
NM_001042625.1	133690	CAPSL	972	25.9003	92.5622	-1.83745	5.00E-05	0.00170056
NM_000598.4	3486	IGFBP3	2613	203.466	71.5229	1.50831	5.00E-05	0.00170056
NM_001073.1	10720	UGT2B11	1722	5.06682	0.275117	4.20296	5.00E-05	0.00170056
TCONS_00003861	XLOC_000716	-	2713	1.28984	4.33183	-1.74778	5.00E-05	0.00170056
TCONS_00293640	XLOC_025525	-	6270	0.942857	4.03428	-2.09720	5.00E-05	0.00170056
NM_032873.4	84959	UBASH3B	6903	10.321	5.05188	1.03069	5.00E-05	0.00170056
NM_005980.2	6286	S100P	510	43.0612	5.77578	2.89830	5.00E-05	0.00170056
TCONS_00252764	XLOC_021384	-	4313	0.453276	1.76081	-1.95778	5.00E-05	0.00170056
TCONS_00012088	XLOC_000773	-	793	78.1617	439.976	-2.49289	5.00E-05	0.00170056
TCONS_00227507	XLOC_018471	-	1774	3.05637	10.4664	-1.77588	5.00E-05	0.00170056
NM_000631.4	4689	NCF4	1398	13.9908	3.12951	2.16047	5.00E-05	0.00170056
NM_152879.2	8527	DGKD	6294	8.68417	3.8769	1.16348	5.00E-05	0.00170056
NM_018407.4	55353	LAPTM4B	2238	39.8211	15.4569	1.36528	5.00E-05	0.00170056
NM_001036.3	6263	RYR3	15559	0.794222	2.2559	-1.50609	5.00E-05	0.00170056
NM_004004.5	2706	GJB2	2334	14.2501	5.78519	1.30054	5.00E-05	0.00170056
NM_145263.2	132671	SPATA18	4300	14.9987	43.5816	-1.53888	5.00E-05	0.00170056
NM_002467.4	4609	MYC	2366	27.3251	8.32247	1.71514	5.00E-05	0.00170056
NM_007361.3	22795	NID2	5046	8.98417	21.1646	-1.23619	5.00E-05	0.00170056
NM_002514.3	4856	NOV	2601	5.74545	34.4704	-2.58487	5.00E-05	0.00170056
NM_007038.3	11096	ADAMTS5	9663	1.9889	6.34585	-1.67384	5.00E-05	0.00170056
NM_033280.2	90701	SEC11C	782	151.468	70.3127	1.10716	5.00E-05	0.00170056
NM_000046.3	411	ARSB	6076	10.7126	3.85151	1.47582	5.00E-05	0.00170056
NM_175884.4	168455	FLJ36031	4424	17.9776	8.38	1.10118	5.00E-05	0.00170056
NM_001080424.1	23135	KDM6B	6704	3.96764	1.62728	1.28581	5.00E-05	0.00170056
NM_173695.2	286464	CXorf59	1956	6.9319	24.0057	-1.79205	5.00E-05	0.00170056
NM_145045.4	115948	CCDC151	2162	1.92587	8.61973	-2.16213	5.00E-05	0.00170056
NM_198964.1	5744	PTHLH	1854	12.9291	0.810568	3.99554	5.00E-05	0.00170056
NM_021149.2	23406	COTL1	1827	63.6715	17.5866	1.85617	5.00E-05	0.00170056
NM_145912.5	150372	NFAM1	5605	2.98475	1.12952	1.40190	5.00E-05	0.00170056
NM_003948.3	8999	CDKL2	4715	0.666814	2.62983	-1.97961	5.00E-05	0.00170056
NM_153235.3	167838	TXLNB	4685	2.56226	7.72893	-1.59285	5.00E-05	0.00170056
NM_144719.3	152206	CCDC13	2830	2.64926	7.80251	-1.55835	5.00E-05	0.00170056
NM_002122.3	3117	HLA-DQA1	1542	94.8367	33.6839	1.49339	5.00E-05	0.00170056
TCONS_00223831	XLOC_018891	-	5712	12.5731	2.34305	2.42388	5.00E-05	0.00170056
NM_024884.2	79944	L2HGDH	6095	14.0919	27.0104	-0.93865	5.00E-05	0.00170056
NM_018076.2	55130	ARMC4	3572	9.8742	41.4973	-2.07128	5.00E-05	0.00170056
NM_004425.3	1893	ECM1	2144	11.4724	2.44236	2.23182	5.00E-05	0.00170056
NM_005304.3	2865	FFAR3	1674	8.77307	0.452675	4.27654	5.00E-05	0.00170056
NM_005384.2	4783	NFIL3	2085	59.6873	9.82135	2.60343	5.00E-05	0.00170056
XM_003118966.1	100506711	LOC100506711	1898	4.06174	20.2473	-2.31756	5.00E-05	0.00170056
NM_001079910.1	84125	LRRIQ1	5444	13.4672	54.0678	-2.00532	5.00E-05	0.00170056
NM_001031745.1	158787	RIBC1	1416	2.28167	14.4864	-2.66654	5.00E-05	0.00170056

NM_001629.3	241	ALOX5AP	906	48.3052	13.3778	1.85234	5.00E-05	0.00170056
NM_153704.5	91147	TMEM67	4726	8.38558	21.2782	-1.34340	5.00E-05	0.00170056
NM_001079878.1	1261	CNGA3	3794	0.241541	1.2203	-2.33690	5.00E-05	0.00170056
NM_002988.2	6362	CCL18	770	279.754	0.653166	8.74249	5.00E-05	0.00170056
NM_000101.2	1535	CYBA	688	162.801	76.725	1.08534	5.00E-05	0.00170056
NM_003897.3	8870	IER3	1240	35.0216	10.9513	1.67714	5.00E-05	0.00170056
NM_152598.2	162333	MARCH10	3101	1.5887	8.47365	-2.41514	5.00E-05	0.00170056
NM_001080448.2	285220	EPHA6	3677	0.186602	1.03577	-2.47267	5.00E-05	0.00170056
NM_001030060.2	389432	SAMD5	6324	12.1427	4.69825	1.36990	5.00E-05	0.00170056
NM_005708.3	10082	GPC6	7103	2.27758	8.49701	-1.89945	5.00E-05	0.00170056
NM_002003.3	2219	FCN1	1283	17.2839	3.82727	2.17504	5.00E-05	0.00170056
NM_024867.3	79925	SPEF2	5638	5.444	22.2826	-2.03318	5.00E-05	0.00170056
NM_012288.3	9697	TRAM2	7057	19.9076	9.93161	1.00322	5.00E-05	0.00170056
NM_144698.3	148741	ANKRD35	3363	1.85108	5.09502	-1.46072	5.00E-05	0.00170056
NM_181711.2	160622	GRASP	1932	8.23946	2.87952	1.51672	5.00E-05	0.00170056
NM_052863.2	92304	SCGB3A1	466	4.88362	80.549	-4.04384	5.00E-05	0.00170056
TCONS_00201765	XLOC_018236	-	8017	1.78016	4.85068	-1.44618	5.00E-05	0.00170056
NM_145170.3	118491	TTC18	3704	10.4075	40.6025	-1.96394	5.00E-05	0.00170056
NM_000265.4	653361	NCF1	1398	13.3119	2.59711	2.35774	5.00E-05	0.00170056
NM_000139.4	2206	MS4A2	3648	27.5668	11.4365	1.26929	5.00E-05	0.00170056
NM_178504.4	201625	DNAH12	9542	12.8389	45.6945	-1.83149	5.00E-05	0.00170056
NM_014466.2	27285	TEKT2	1509	5.07481	24.9418	-2.29714	5.00E-05	0.00170056
NM_025015.2	259217	HSPA12A	5722	1.47978	3.58952	-1.27841	5.00E-05	0.00170056
NM_005627.3	6446	SGK1	2407	134.757	42.5093	1.66450	5.00E-05	0.00170056
NM_207317.1	133923	ZNF474	1957	6.47632	20.8802	-1.68889	5.00E-05	0.00170056
NM_000702.3	477	ATP1A2	5464	6.65188	38.7615	-2.54279	5.00E-05	0.00170056
NM_001085429.1	155006	TMEM213	3390	5.64097	26.4686	-2.23027	5.00E-05	0.00170056
NM_014903.4	89795	NAV3	9758	2.21698	5.85657	-1.40146	5.00E-05	0.00170056
NM_173494.1	139212	CXorf41	1652	3.14892	15.2259	-2.27360	5.00E-05	0.00170056
NM_002581.3	5069	PAPPA	10970	32.4343	7.75811	2.06375	5.00E-05	0.00170056
NM_031935.2	83872	HMCN1	18212	27.1518	11.1751	1.28077	5.00E-05	0.00170056
NM_006639.2	10800	CYSLTR1	1514	20.5954	7.66382	1.42618	5.00E-05	0.00170056
NM_024807.2	79865	TREML2	3758	2.55859	0.469347	2.44662	5.00E-05	0.00170056
NM_178827.4	154865	IQUB	3115	8.04017	31.1307	-1.95304	5.00E-05	0.00170056
NM_178837.3	352909	C19orf51	2168	2.62417	8.7638	-1.73970	5.00E-05	0.00170056
NM_031956.2	83894	TTC29	1786	8.44536	27.9181	-1.72497	5.00E-05	0.00170056
NM_003014.3	6424	SFRP4	2974	7.14851	153.406	-4.42357	5.00E-05	0.00170056
NM_002727.2	5552	SRGN	1254	600.399	112.672	2.41379	5.00E-05	0.00170056
NM_015662.1	26160	IFT172	5360	14.0191	31.0676	-1.14802	5.00E-05	0.00170056
NM_033160.5	26149	ZNF658	4025	4.696	10.1793	-1.11614	5.00E-05	0.00170056
NM_020693.2	57453	DSCAML1	6899	0.235658	1.03849	-2.13973	5.00E-05	0.00170056
NM_012144.2	27019	DNAI1	2521	7.08097	24.9454	-1.81675	5.00E-05	0.00170056
NM_032606.3	84698	CAPS2	2135	6.00691	21.1214	-1.81401	5.00E-05	0.00170056
NM_001040429.2	27253	PCDH17	8009	6.79971	2.6493	1.35986	5.00E-05	0.00170056
NM_015931.1	51066	C3orf32	1545	0.348944	2.79847	-3.00357	5.00E-05	0.00170056
NM_001077710.2	642273	FAM110C	3963	10.7553	2.43354	2.14393	5.00E-05	0.00170056
NM_000962.2	5742	PTGS1	5093	8.82318	2.88737	1.61154	5.00E-05	0.00170056
NM_014632.2	9645	MICAL2	3906	10.2637	3.88019	1.40335	5.00E-05	0.00170056
NM_002649.2	5294	PIK3CG	5379	18.0233	7.36006	1.29208	5.00E-05	0.00170056
NM_000044.2	367	AR	4314	0.790178	3.04562	-1.94649	5.00E-05	0.00170056
NM_173216.2	6480	ST6GAL1	4604	43.8891	17.7964	1.30228	5.00E-05	0.00170056
NM_015693.3	27152	INTU	3275	7.3657	20.2313	-1.45770	5.00E-05	0.00170056
NM_001039844.2	414149	ACBD7	3370	1.42869	4.72131	-1.72450	5.00E-05	0.00170056
TCONS_00249726	XLOC_021972	-	531	0	1.68252	#NAME?	5.00E-05	0.00170056

NM_031415.2	56169	GSDMC	2714	6.78051	1.90975	1.82801	5.00E-05	0.00170056
NM_000065.2	729	C6	3606	3.27154	21.8203	-2.73763	5.00E-05	0.00170056
NM_000417.2	3559	IL2RA	3216	5.75801	1.52618	1.91564	5.00E-05	0.00170056
NM_178456.2	128602	C20orf85	760	39.5108	158.377	-2.00305	5.00E-05	0.00170056
NM_003821.5	8767	RIPK2	2585	12.6198	3.62347	1.80025	5.00E-05	0.00170056
NM_001198557.1	4001	LMNB1	2250	17.5965	6.30134	1.48156	5.00E-05	0.00170056
TCONS_00057873	XLOC_005084	-	5007	3.53054	8.61976	-1.28776	5.00E-05	0.00170056
NM_002984.2	6351	CCL4	667	33.9214	9.08694	1.90033	5.00E-05	0.00170056
NM_024888.1	79948	LPPR3	2357	1.02215	4.00613	-1.97060	5.00E-05	0.00170056
NM_007153.3	7757	ZNF208	9088	2.41327	6.95137	-1.52631	5.00E-05	0.00170056
NM_152680.2	201799	TMEM154	3216	34.4422	14.4901	1.24911	5.00E-05	0.00170056
NM_003619.3	8492	PRSS12	4573	11.6658	27.5263	-1.23853	5.00E-05	0.00170056
NM_001100159.1	136288	C7orf57	2125	3.93158	12.3181	-1.64759	5.00E-05	0.00170056
NM_032824.2	84910	TMEM87B	5212	39.4996	20.7994	0.92530	5.00E-05	0.00170056
NM_024560.2	79611	ACSS3	3033	5.82008	19.9558	-1.77770	5.00E-05	0.00170056
NM_145047.4	127700	OSCP1	1489	11.4481	29.7953	-1.37998	5.00E-05	0.00170056
NM_005204.2	1326	MAP3K8	3096	30.9818	10.5383	1.55578	5.00E-05	0.00170056
NM_012190.2	10840	ALDH1L1	3125	2.22124	9.22464	-2.05413	5.00E-05	0.00170056
NM_000061.2	695	BTK	2611	22.4299	6.12768	1.87201	5.00E-05	0.00170056
NM_144715.3	151651	EFHB	2857	8.40831	35.2173	-2.06640	5.00E-05	0.00170056
NM_001013647.1	646851	LOC646851	10322	1.77391	5.49157	-1.63028	5.00E-05	0.00170056
NM_013272.3	28232	SLCO3A1	5106	7.86589	3.09837	1.34410	5.00E-05	0.00170056
NM_018584.5	55450	CAMK2N1	2351	10.5932	3.24987	1.70468	5.00E-05	0.00170056
NM_003979.3	9052	GPRC5A	2851	6.94941	2.34565	1.56690	5.00E-05	0.00170056
NM_012328.2	4189	DNAJB9	2538	39.975	18.8914	1.08136	5.00E-05	0.00170056
NM_001174072.1	256987	SERINC5	6479	24.783	10.2662	1.27146	5.00E-05	0.00170056
NM_014674.2	9695	EDEM1	6153	29.5093	12.1409	1.28130	5.00E-05	0.00170056
NM_001010940.1	138255	C9orf135	851	11.6641	34.1389	-1.54934	5.00E-05	0.00170056
NM_007074.3	11151	CORO1A	1815	36.571	12.1786	1.58636	5.00E-05	0.00170056
NM_001170820.3	402778	IFITM10	3709	0.698434	2.86259	-2.03513	5.00E-05	0.00170056
NM_020212.1	56964	WDR93	2409	1.50382	5.25529	-1.80514	5.00E-05	0.00170056
NM_032576.4	84663	CYorf15B	3315	21.5738	4.0505	2.41311	5.00E-05	0.00170056
NM_033274.3	8728	ADAM19	6481	10.8558	3.69234	1.55586	5.00E-05	0.00170056
NM_000396.3	1513	CTSK	1810	46.1817	127.079	-1.46033	5.00E-05	0.00170056
NM_012395.2	5218	CDK14	4953	13.6129	27.1805	-0.99760	5.00E-05	0.00170056
NM_006280.2	6748	SSR4	725	199.183	78.1322	1.35011	5.00E-05	0.00170056
NM_031898.2	64518	TEKT3	1784	0.90014	3.70959	-2.04304	5.00E-05	0.00170056
NM_054110.4	117248	GALNTL2	4641	1.2918	3.83698	-1.57059	5.00E-05	0.00170056
NM_000655.4	6402	SELL	2442	23.0295	7.72278	1.57629	5.00E-05	0.00170056
NM_178821.1	164781	WDR69	1669	9.13292	39.8756	-2.12636	5.00E-05	0.00170056
NM_001064.3	7086	TKT	2164	48.736	21.9879	1.14828	5.00E-05	0.00170056
NM_005213.3	1475	CSTA	828	43.2697	10.0048	2.11267	5.00E-05	0.00170056
NM_001130162.2	219736	STOX1	3112	10.9188	26.7714	-1.29387	5.00E-05	0.00170056
XM_003118942.2	374467	C12orf63	2401	6.2977	24.4605	-1.95756	5.00E-05	0.00170056
NM_001067.3	7153	TOP2A	5753	14.6506	4.72787	1.63169	5.00E-05	0.00170056
NM_144644.2	132851	SPATA4	1233	1.66859	7.80859	-2.22644	5.00E-05	0.00170056
TCONS_00000209	XLOC_000044	-	4800	0.472175	1.8116	-1.93987	5.00E-05	0.00170056
NM_012443.2	9576	SPAG6	2594	16.7179	57.6194	-1.78516	5.00E-05	0.00170056
NM_153183.2	170685	NUDT10	2001	22.8247	3.37292	2.75853	5.00E-05	0.00170056
NM_002432.1	4332	MNDA	1667	31.3812	13.1432	1.25559	5.00E-05	0.00170056
NM_173572.3	255352	C10orf93	1587	2.55775	8.79777	-1.78226	5.00E-05	0.00170056
NM_001080850.2	728621	CCDC30	3098	3.12308	9.41758	-1.59239	5.00E-05	0.00170056
NM_001109.4	101	ADAM8	3301	12.3708	1.47301	3.07009	5.00E-05	0.00170056
NM_001040058.1	6696	SPP1	1625	23.5519	102.889	-2.12717	5.00E-05	0.00170056

TCONS_00283288	XLOC_024299	-	6864	1.11415	3.02398	-1.44051	5.00E-05	0.00170056
NM_001030287.2	467	ATF3	1899	45.0643	6.03787	2.89987	5.00E-05	0.00170056
NM_002659.3	5329	PLAUR	1553	17.4066	1.37001	3.66738	5.00E-05	0.00170056
NM_013250.2	7762	ZNF215	3658	8.10533	2.64557	1.61529	5.00E-05	0.00170056
NM_002546.3	4982	TNFRSF11B	2346	7.52949	2.03945	1.88437	5.00E-05	0.00170056
TCONS_00070847	XLOC_005757	-	1130	5.74113	0.477057	3.58910	5.00E-05	0.00170056
NM_001010922.2	440603	BCL2L15	4973	4.73389	1.73169	1.45085	5.00E-05	0.00170056
NM_020377.2	57105	CYSLTR2	2548	6.56887	2.15613	1.60720	5.00E-05	0.00170056
NM_004460.2	2191	FAP	2780	19.0462	7.25056	1.39334	5.00E-05	0.00170056
NM_000217.2	3736	KCNA1	7983	0.253352	2.53179	-3.32095	5.00E-05	0.00170056
NM_152376.3	127733	UBXN10	2972	12.5561	47.6268	-1.92338	5.00E-05	0.00170056
NM_145026.3	221409	SPATS1	1108	1.25357	5.21639	-2.05701	5.00E-05	0.00170056
NM_001156474.1	60494	CCDC81	2781	3.36532	13.5553	-2.01004	5.00E-05	0.00170056
NM_145054.4	146845	WDR16	2193	5.82124	27.0806	-2.21786	5.00E-05	0.00170056
TCONS_00249239	XLOC_021575	-	5196	0.251903	2.92079	-3.53542	5.00E-05	0.00170056
NM_002229.2	3726	JUNB	1816	148.001	12.9681	3.51257	5.00E-05	0.00170056
NM_001679.2	483	ATP1B3	1853	62.2088	21.1698	1.55511	5.00E-05	0.00170056
NM_001080427.1	80731	THSD7B	5846	0.413448	2.51427	-2.60436	5.00E-05	0.00170056
NM_033027.3	64651	CSRNP1	3188	35.6808	4.02159	3.14931	5.00E-05	0.00170056
NM_145286.2	161003	STOML3	1914	10.6788	25.2226	-1.23997	5.00E-05	0.00170056
NM_002048.2	2619	GAS1	2828	30.6588	15.1699	1.01509	5.00E-05	0.00170056
TCONS_00003383	XLOC_000243	-	4161	0.938795	3.75326	-1.99926	5.00E-05	0.00170056
NM_002825.5	5764	PTN	1549	15.0362	60.3928	-2.00594	5.00E-05	0.00170056
NM_002046.3	2597	GAPDH	1310	738.082	290.794	1.34379	5.00E-05	0.00170056
NM_001003443.2	390083	OR56A3	948	0	0.937648	#NAME?	5.00E-05	0.00170056
NM_006636.3	10797	MTHFD2	2188	21.7496	8.87822	1.29265	5.00E-05	0.00170056
NM_001944.2	1830	DSG3	5551	4.54055	1.05254	2.10899	5.00E-05	0.00170056
NM_002460.3	3662	IRF4	5327	10.6825	3.36478	1.66667	5.00E-05	0.00170056
NM_004525.2	4036	LRP2	15735	15.161	4.92522	1.62210	5.00E-05	0.00170056
NM_000219.3	3753	KCNE1	3321	3.96127	10.7817	-1.44455	5.00E-05	0.00170056
TCONS_00000172	XLOC_000042	-	1923	0.669346	5.6619	-3.08046	5.00E-05	0.00170056
NM_001039706.2	79846	C7orf63	3902	6.30568	17.8984	-1.50510	5.00E-05	0.00170056
NM_001080484.1	85452	KIAA1751	4710	0.878993	4.02044	-2.19343	5.00E-05	0.00170056
NM_032571.3	84658	EMR3	2301	15.9376	0.30838	5.69158	5.00E-05	0.00170056
NM_052941.4	115361	GBP4	6145	12.4544	30.1788	-1.27688	5.00E-05	0.00170056
NM_144717.3	53833	IL20RB	2047	10.9365	3.4085	1.68194	5.00E-05	0.00170056
NM_001911.2	1511	CTSG	924	46.5036	6.01945	2.94964	5.00E-05	0.00170056
NM_001144960.1	389161	ANKUB1	2476	10.4254	27.8536	-1.41775	5.00E-05	0.00170056
NM_004352.3	869	CBLN1	2435	0.296986	3.92345	-3.72365	5.00E-05	0.00170056
TCONS_00000123	XLOC_000026	-	2415	0.250394	1.81299	-2.85610	5.00E-05	0.00170056
NM_024574.3	79625	NDNF	2871	3.33565	12.2793	-1.88018	5.00E-05	0.00170056
NM_000615.6	4684	NCAM1	5962	1.0277	5.78442	-2.49275	5.00E-05	0.00170056
NM_002125.3	3127	HLA-DRB5	1171	5.43834	22.1431	-2.02562	5.00E-05	0.00170056
NM_001012642.2	196996	GRAMD2	3292	3.82356	9.40145	-1.29796	5.00E-05	0.00170056
NM_000526.4	3861	KRT14	1636	5.29778	0.611884	3.11406	5.00E-05	0.00170056
NM_005566.3	3939	LDHA	2208	234.909	77.9652	1.59120	5.00E-05	0.00170056
NM_032211.6	84171	LOXL4	3657	25.2029	8.47835	1.57174	5.00E-05	0.00170056
NM_152362.2	126282	TNFAIP8L1	3817	6.40115	16.0006	-1.32172	5.00E-05	0.00170056
NM_030926.4	81618	ITM2C	2073	59.6746	24.8347	1.26476	5.00E-05	0.00170056
NM_145252.2	124220	ZG16B	828	13.1537	61.1025	-2.21576	5.00E-05	0.00170056
NM_025184.3	80258	EFHC2	3252	5.64227	18.893	-1.74350	5.00E-05	0.00170056
NM_007350.3	22822	PHLDA1	5913	26.4457	5.7379	2.20444	5.00E-05	0.00170056
NM_015675.3	4616	GADD45B	1375	52.339	21.8018	1.26344	5.00E-05	0.00170056
NM_012387.2	23569	PADI4	2265	4.85325	0.317879	3.93240	5.00E-05	0.00170056

NM_001554.4	3491	CYR61	2295	110.679	13.7271	3.01128	5.00E-05	0.00170056
NM_020299.4	57016	AKR1B10	1596	6.47911	1.1011	2.55685	5.00E-05	0.00170056
NM_012397.3	5275	SERPINB13	3180	17.1446	4.23549	2.01715	5.00E-05	0.00170056
NM_000112.3	1836	SLC26A2	8075	56.1722	22.1299	1.34386	5.00E-05	0.00170056
NM_020869.3	100506627	DCDC5	4759	2.80936	9.17827	-1.70798	5.00E-05	0.00170056
NM_004508.2	3422	IDI1	2137	65.5286	29.5263	1.15012	5.00E-05	0.00170056
NM_182500.2	130813	C2orf50	1056	1.11889	6.83274	-2.61039	5.00E-05	0.00170056
NM_002115.2	3101	HK3	3068	15.1287	0.663501	4.51105	5.00E-05	0.00170056
NM_001039763.3	642987	TMEM232	3193	9.65451	24.1181	-1.32084	5.00E-05	0.00170056
TCONS_00165587	XLOC_013757	-	437	2.9392	0	inf	5.00E-05	0.00170056
NM_004363.2	1048	CEACAM5	3600	41.1753	0.671976	5.93723	5.00E-05	0.00170056
NM_001098514.1	146556	C16orf89	1514	3.06909	14.9426	-2.28354	5.00E-05	0.00170056
NM_207437.3	196385	DNAH10	13680	6.57225	23.8282	-1.85821	5.00E-05	0.00170056
NM_004058.3	828	CAPS	1609	47.3822	131.512	-1.47278	5.00E-05	0.00170056
NM_006691.3	10894	LYVE1	2500	44.01	19.3941	1.18221	5.00E-05	0.00170056
NM_001145018.1	283152	CCDC153	767	4.90997	16.3248	-1.73328	5.00E-05	0.00170056
NM_178813.5	158798	AKAP14	841	6.71294	30.2542	-2.17212	5.00E-05	0.00170056
NM_000419.3	3674	ITGA2B	3334	1.06	0.199398	2.41035	5.00E-05	0.00170056
NM_002977.3	6335	SCN9A	9762	4.71871	1.78019	1.40636	5.00E-05	0.00170056
TCONS_00032728	XLOC_003418	-	2076	4.3234	47.5869	-3.46033	5.00E-05	0.00170056
NM_003955.3	9021	SOCS3	2734	117.977	3.55188	5.05378	5.00E-05	0.00170056
NM_001127391.1	130540	ALS2CR12	1944	3.72552	16.0725	-2.10908	5.00E-05	0.00170056
XM_003118889.1	100506859	LOC100506859	703	5.49813	22.1987	-2.01346	5.00E-05	0.00170056
NM_017855.3	54959	ODAM	1288	54.2032	7.88492	2.78121	5.00E-05	0.00170056
NM_000130.4	2153	F5	9179	5.81776	2.19784	1.40438	5.00E-05	0.00170056
NM_002974.2	6318	SERPINB4	1719	103.029	3.23896	4.99138	5.00E-05	0.00170056
NM_017886.2	54986	ULK4	4359	9.24386	21.088	-1.18985	5.00E-05	0.00170056
NM_145020.3	220136	CCDC11	1824	6.70235	28.2129	-2.07362	5.00E-05	0.00170056
NM_017589.2	54766	BTG4	999	2.75616	10.9686	-1.99266	5.00E-05	0.00170056
NM_032611.1	11156	PTP4A3	1332	16.5478	4.44912	1.89505	5.00E-05	0.00170056
NM_152687.2	202309	GAPT	2216	28.9959	5.16463	2.48911	5.00E-05	0.00170056
NM_080390.3	140597	TCEAL2	1101	1.34963	9.36736	-2.79508	5.00E-05	0.00170056
NM_003360.3	7368	UGT8	2898	10.1334	20.8189	-1.03878	5.00E-05	0.00170056
NM_003370.3	7408	VASP	2291	31.042	11.2423	1.46528	5.00E-05	0.00170056
NM_004907.2	9592	IER2	2050	29.3684	5.29304	2.47210	5.00E-05	0.00170056
NM_152770.2	255119	C4orf22	895	6.7066	28.0882	-2.06631	5.00E-05	0.00170056
NM_145175.2	151354	FAM84A	6355	9.19153	4.57183	1.00753	5.00E-05	0.00170056
NM_000358.2	7045	TGFBI	2805	82.931	39.0258	1.08749	5.00E-05	0.00170056
TCONS_00282969	XLOC_023780	-	3213	0.921909	3.73735	-2.01932	5.00E-05	0.00170056
NM_017839.4	54947	LPCAT2	5388	20.1654	6.29546	1.67950	5.00E-05	0.00170056
NM_197941.2	11174	ADAMTS6	7268	3.10697	1.34298	1.21007	5.00E-05	0.00170056
NM_014079.3	28999	KLF15	2525	1.22461	4.99131	-2.02710	5.00E-05	0.00170056
NM_004994.2	4318	MMP9	2336	0.916865	14.5385	-3.98702	5.00E-05	0.00170056
NM_001033602.2	23281	MTUS2	6939	0.319814	1.43098	-2.16171	5.00E-05	0.00170056
NM_024418.1	9256	BZRAP1	7514	2.20831	5.13842	-1.21838	5.00E-05	0.00170056
NM_001870.2	1359	CPA3	1686	264.984	36.5306	2.85873	5.00E-05	0.00170056
NM_003043.3	6533	SLC6A6	6508	126.986	50.2661	1.33701	5.00E-05	0.00170056
NM_013381.2	29953	TRHDE	5552	0.335649	3.75703	-3.48457	5.00E-05	0.00170056
NM_203339.2	1191	CLU	2860	122.375	279.351	-1.19077	5.00E-05	0.00170056
NM_053285.1	83659	TEKT1	1413	20.1326	79.21	-1.97615	5.00E-05	0.00170056
NM_000930.3	5327	PLAT	3173	5.84999	28.5812	-2.28856	5.00E-05	0.00170056
NM_002123.3	3119	HLA-DQB1	1224	89.406	22.0882	2.01709	5.00E-05	0.00170056
NM_153264.5	256076	COL6A5	8878	6.48335	0.219015	4.88764	5.00E-05	0.00170056
NM_001113547.1	51474	LIMA1	3622	102.866	27.722	1.89166	5.00E-05	0.00170056

NM_001145442.1	641455	POTEM	6666	0.27445	1.23478	-2.16964	5.00E-05	0.00170056
NM_020406.2	57126	CD177	2364	9.13642	1.36081	2.74716	5.00E-05	0.00170056
NM_007231.3	11254	SLC6A14	4564	106.149	29.2591	1.85914	5.00E-05	0.00170056
NM_199347.2	152110	NEK10	2639	11.1674	45.0304	-2.01160	5.00E-05	0.00170056
NM_001130958.1	2172	FABP6	743	7.31356	48.3289	-2.72424	5.00E-05	0.00170056
NM_002205.2	3678	ITGA5	4250	15.2216	6.36978	1.25681	5.00E-05	0.00170056
NM_000851.3	2949	GSTM5	1570	0.357057	7.12814	-4.31930	5.00E-05	0.00170056
NM_025052.3	80122	YSK4	4165	7.89697	29.9628	-1.92380	5.00E-05	0.00170056
NM_018015.5	55086	CXorf57	3904	0.482906	2.29723	-2.25008	5.00E-05	0.00170056
NM_001218.3	771	CA12	3975	21.661	3.83575	2.49752	5.00E-05	0.00170056
NM_001005473.2	345557	PLCXD3	7739	0.342657	2.00238	-2.54688	5.00E-05	0.00170056
NM_001030059.1	196051	PPAPDC1A	1521	4.88901	1.12718	2.11682	5.00E-05	0.00170056
NM_018986.3	54436	SH3TC1	4260	7.47804	2.66184	1.49024	5.00E-05	0.00170056
NM_173565.3	222967	RSPH10B	3106	1.18031	5.25776	-2.15529	5.00E-05	0.00170056
NM_024913.4	79974	C7orf58	5320	5.19445	14.1535	-1.44612	5.00E-05	0.00170056
NM_052901.2	114789	SLC25A25	3240	21.564	5.28853	2.02769	5.00E-05	0.00170056
NM_014737.2	9770	RASSF2	5426	44.2518	12.0377	1.87818	5.00E-05	0.00170056
TCONS_00037389	XLOC_003418	-	566	11.1642	128.948	-3.52984	5.00E-05	0.00170056
TCONS_00037388	XLOC_003418	-	788	10.2059	107.376	-3.39521	5.00E-05	0.00170056
NM_005217.3	1668	DEFA3	491	0	16.4563	#NAME?	5.00E-05	0.00170056
NM_152750.4	222256	CDHR3	6500	11.7392	50.6512	-2.10926	5.00E-05	0.00170056
NM_001200049.1	54777	C10orf92	3164	1.01772	4.63382	-2.18686	5.00E-05	0.00170056
TCONS_00053981	XLOC_004304	-	6951	2.09702	0.590623	1.82804	5.00E-05	0.00170056
NM_176677.1	283948	NHLRC4	2044	2.27652	6.93709	-1.60750	5.00E-05	0.00170056
NM_002438.2	4360	MRC1	5171	1.85437	0.491562	1.91549	5.00E-05	0.00170056
NM_003226.3	7033	TFF3	1054	104.977	17.8191	2.55858	5.00E-05	0.00170056
NM_018365.2	55329	MNS1	2023	10.8548	34.2402	-1.65736	5.00E-05	0.00170056
NM_033397.2	85450	ITPRIP	4364	10.4546	2.66226	1.97341	5.00E-05	0.00170056
NM_001162435.2	100287718	LOC100287718	1058	6.02541	23.6331	-1.97168	5.00E-05	0.00170056
TCONS_00202192	XLOC_017222	-	11403	2.19586	9.68556	-2.14105	5.00E-05	0.00170056
NM_004469.4	2277	FIGF	2074	0.477196	3.66238	-2.94013	5.00E-05	0.00170056
NM_001008723.1	159686	CCDC147	3313	3.64312	14.4689	-1.98971	5.00E-05	0.00170056
XM_003403450.1	4586	MUC5AC	6325	52.1879	13.861	1.91268	5.00E-05	0.00170056
NM_014467.2	27286	SRPX2	2190	11.3609	3.79832	1.58065	5.00E-05	0.00170056
NM_025195.2	10221	TRIB1	3633	39.9782	7.00342	2.51308	5.00E-05	0.00170056
NM_004654.3	8287	USP9Y	10040	18.879	5.59332	1.75500	5.00E-05	0.00170056
XM_003403441.1	401258	RAB44	2557	4.65397	0.638754	2.86513	5.00E-05	0.00170056
NM_152997.2	260436	C4orf7	539	100.371	19.3682	2.37358	5.00E-05	0.00170056
NM_145038.2	92749	CCDC164	2491	12.9269	46.5596	-1.84870	5.00E-05	0.00170056
NM_024800.4	79858	NEK11	2953	7.30507	24.9861	-1.77415	5.00E-05	0.00170056
NM_001805.2	1053	CEBPE	1201	8.15414	0.574705	3.82664	5.00E-05	0.00170056
NM_014226.1	5891	RAGE	1934	4.32682	14.0841	-1.70269	5.00E-05	0.00170056
NM_003253.2	7074	TIAM1	7198	7.1131	2.3964	1.56961	5.00E-05	0.00170056
NM_174892.2	124599	CD300LB	2294	5.65704	0.641764	3.13993	5.00E-05	0.00170056
NM_004049.3	597	BCL2A1	887	63.1543	4.45226	3.82627	5.00E-05	0.00170056
NM_002167.3	3399	ID3	1288	52.1784	18.2642	1.51443	5.00E-05	0.00170056
NM_002318.2	4017	LOXL2	3810	11.6407	2.18705	2.41212	5.00E-05	0.00170056
NM_004670.3	9060	PAPSS2	3843	15.4756	5.99851	1.36732	5.00E-05	0.00170056
XM_001725354.4	4584	MUC3A	5765	0.132806	0.753566	-2.50441	5.00E-05	0.00170056
TCONS_00267790	XLOC_022887	-	1986	0.673336	4.46003	-2.72766	5.00E-05	0.00170056
NM_000963.2	5743	PTGS2	4493	41.232	2.13877	4.26891	5.00E-05	0.00170056
NM_002545.3	4978	OPCML	6300	0.253342	1.04371	-2.04256	5.00E-05	0.00170056
NM_001098526.1	120425	AMICA1	1957	62.688	14.4784	2.11429	5.00E-05	0.00170056
NM_004274.4	9472	AKAP6	10387	5.29712	11.8848	-1.16584	5.00E-05	0.00170056

NM_005130.3	9982	FGFBP1	1182	7.63529	0.744421	3.35849	5.00E-05	0.00170056
NM_002648.3	5292	PIM1	2709	31.7851	6.26396	2.34321	5.00E-05	0.00170056
NM_006528.2	7980	TFPI2	1170	0.891779	4.94669	-2.47171	5.00E-05	0.00170056
NM_004621.5	7225	TRPC6	4612	7.72301	2.66885	1.53294	5.00E-05	0.00170056
NM_012309.3	22941	SHANK2	10495	2.13441	4.74797	-1.15347	5.00E-05	0.00170056
NM_012108.2	26228	STAP1	1511	13.8144	5.70392	1.27615	5.00E-05	0.00170056
NM_001039845.1	130752	MDH1B	2332	8.98692	26.8397	-1.57847	5.00E-05	0.00170056
NM_000922.3	5140	PDE3B	6091	8.94566	2.87868	1.63578	5.00E-05	0.00170056
NM_000860.4	3248	HPGD	2770	45.0813	13.1485	1.77763	5.00E-05	0.00170056
NM_001004330.2	440107	PLEKHG7	3267	8.36482	24.0215	-1.52192	5.00E-05	0.00170056
NM_000064.2	718	C3	5101	23.4449	85.8372	-1.87233	5.00E-05	0.00170056
NM_182628.2	348807	CCDC37	2086	3.32923	10.6533	-1.67804	5.00E-05	0.00170056
NM_003357.4	7356	SCGB1A1	452	48.8071	2998.95	-5.94122	5.00E-05	0.00170056
NM_005195.3	1052	CEBPD	1251	99.0476	45.7298	1.11499	5.00E-05	0.00170056
NM_001002912.4	127254	C1orf173	7159	12.8494	40.3861	-1.65215	5.00E-05	0.00170056
NM_000602.3	5054	SERPINE1	3181	22.7471	0.726664	4.96825	5.00E-05	0.00170056
NM_030817.2	81575	APOLD1	4646	76.8949	6.02826	3.67307	5.00E-05	0.00170056
NM_001146187.1	5178	PEG3	8375	2.35229	5.29089	-1.16944	5.00E-05	0.00170056
NM_001139490.1	26146	TRAF3IP1	3978	11.3496	22.1968	-0.96771	5.00E-05	0.00170056
NM_005737.3	10123	ARL4C	4009	27.1432	10.0159	1.43829	5.00E-05	0.00170056
NM_032048.2	84034	EMILIN2	4009	16.7502	5.86863	1.51308	5.00E-05	0.00170056
NM_005560.3	3911	LAMA5	11426	7.83795	3.92468	0.99790	5.00E-05	0.00170056
NM_133493.3	135228	CD109	9447	10.9258	4.64795	1.23307	5.00E-05	0.00170056
NM_182665.2	83593	RASSF5	3514	24.6482	7.55208	1.70653	5.00E-05	0.00170056
NM_000189.4	3099	HK2	7093	13.488	2.62488	2.36135	5.00E-05	0.00170056
NM_016459.3	51237	MZB1	827	76.7114	25.3635	1.59669	5.00E-05	0.00170056
NM_020826.2	57586	SYT13	5157	0.166881	0.963175	-2.52898	5.00E-05	0.00170056
NM_005582.2	4064	CD180	2716	11.7325	3.95623	1.56831	5.00E-05	0.00170056
NM_173812.4	283417	DPY19L2	4057	2.20282	8.58391	-1.96228	5.00E-05	0.00170056
NM_015964.2	51673	TPPP3	1041	43.9425	137.903	-1.64996	5.00E-05	0.00170056
NM_001992.3	2149	F2R	3810	35.0886	14.6214	1.26292	5.00E-05	0.00170056
NM_001145252.1	5199	CFP	1598	5.01983	1.39269	1.84977	5.00E-05	0.00170056
NM_139285.2	246176	GAS2L2	3007	2.55213	8.46353	-1.72956	5.00E-05	0.00170056
NM_001001413.3	283767	GOLGA6L1	3822	0.173604	0.889192	-2.35669	5.00E-05	0.00170056
NM_005623.2	6355	CCL8	1250	8.91826	1.16458	2.93696	5.00E-05	0.00170056
NM_032821.2	54768	HYDIN	15685	3.15632	9.86485	-1.64406	5.00E-05	0.00170056
NM_015668.3	26166	RGS22	4184	9.38407	28.5281	-1.60410	5.00E-05	0.00170056
NM_004235.4	9314	KLF4	2933	28.586	7.09946	2.00952	5.00E-05	0.00170056
NM_001257.4	1012	CDH13	4021	9.97041	3.34009	1.57777	5.00E-05	0.00170056
NM_032581.3	84668	FAM126A	6151	24.8422	13.0555	0.92813	5.00E-05	0.00170056
NM_018010.3	55081	IFT57	3203	26.8654	57.986	-1.10996	5.00E-05	0.00170056
NM_001101376.2	440585	FAM183A	517	14.872	43.9075	-1.56186	5.00E-05	0.00170056
NM_001001557.2	392255	GDF6	3701	0.151771	1.32606	-3.12718	5.00E-05	0.00170056
NM_152572.2	158067	AK8	2106	2.24374	6.85179	-1.61057	5.00E-05	0.00170056
NM_004946.2	1794	DOCK2	6097	34.0545	17.1549	0.98922	5.00E-05	0.00170056
TCONS_00155673	XLOC_013982	-	9015	0.546383	3.12484	-2.51580	5.00E-05	0.00170056
NM_001014440.3	440836	ODF3B	970	4.62358	16.2577	-1.81404	5.00E-05	0.00170056
NM_003999.2	9180	OSMR	5539	36.0999	13.1124	1.46106	5.00E-05	0.00170056
NM_052904.3	114792	KLHL32	3807	1.03989	4.80965	-2.20949	5.00E-05	0.00170056
NM_004165.2	6236	RRAD	1467	7.86083	24.4382	-1.63638	5.00E-05	0.00170056
NM_000801.4	2280	FKBP1A	1633	73.5474	34.8132	1.07904	5.00E-05	0.00170056
NM_020183.3	56938	ARNTL2	1930	12.6242	3.62337	1.80079	5.00E-05	0.00170056
NM_014485.2	27306	HPGDS	1615	16.8697	5.17919	1.70364	5.00E-05	0.00170056
NM_005253.3	2355	FOSL2	3991	51.0057	14.0562	1.85945	5.00E-05	0.00170056

NM_001184744.1	5745	PTH1R	1991	1.98806	6.44491	-1.69680	5.00E-05	0.00170056
NM_004174.2	6550	SLC9A3	2777	6.17222	1.08635	2.50630	5.00E-05	0.00170056
NM_182943.2	5352	PLOD2	4060	32.1354	12.016	1.41921	5.00E-05	0.00170056
TCONS_00220457	XLOC_019175	-	402	0	1.76771	#NAME?	5.00E-05	0.00170056
NM_002112.3	3067	HDC	2644	16.561	3.65468	2.17997	5.00E-05	0.00170056
NM_000361.2	7056	THBD	4032	14.9135	3.60757	2.04751	5.00E-05	0.00170056
NM_025250.2	80727	TTYH3	4822	7.22617	2.63583	1.45498	5.00E-05	0.00170056
NM_005164.3	225	ABCD2	5341	2.69717	6.283	-1.22001	5.00E-05	0.00170056
NM_015515.3	25984	KRT23	2147	25.4554	10.4588	1.28325	5.00E-05	0.00170056
NM_002228.3	3725	JUN	3323	61.3097	21.7503	1.49508	5.00E-05	0.00170056
NM_199478.1	5354	PLP1	2921	2.1592	8.45052	-1.96854	5.00E-05	0.00170056
NM_001031743.2	154313	C6orf165	2215	11.9943	45.2977	-1.91709	5.00E-05	0.00170056
TCONS_00208641	XLOC_017561	-	1790	7.7598	1.14283	2.76341	5.00E-05	0.00170056
NM_032385.3	10826	C5orf4	2997	9.1989	20.1378	-1.13037	5.00E-05	0.00170056
NM_024677.4	79730	NSUN7	3698	6.82306	21.2879	-1.64155	5.00E-05	0.00170056
NM_004914.2	9609	RAB36	3822	3.21493	7.64637	-1.24999	5.00E-05	0.00170056
NM_014996.2	23007	PLCH1	6128	3.26263	9.38505	-1.52433	5.00E-05	0.00170056
NM_002863.4	5836	PYGL	2846	44.2717	17.2338	1.36114	5.00E-05	0.00170056
NM_007360.3	22914	KLRK1	1589	15.9313	41.124	-1.36811	5.00E-05	0.00170056
NM_001783.3	973	CD79A	1258	33.8568	10.9739	1.62537	5.00E-05	0.00170056
NM_003155.2	6781	STC1	3877	19.4096	6.10944	1.66766	5.00E-05	0.00170056
NM_003777.3	8701	DNAH11	14189	10.0375	33.749	-1.74945	5.00E-05	0.00170056
NM_000433.3	4688	NCF2	2412	59.3687	8.00231	2.89121	5.00E-05	0.00170056
NM_012194.2	25758	C11orf41	11622	0.430519	1.69954	-1.98099	5.00E-05	0.00170056
NM_030820.3	81578	COL21A1	4173	4.14476	12.6011	-1.60419	5.00E-05	0.00170056
NM_002661.2	5336	PLCG2	4241	21.303	8.93604	1.25335	5.00E-05	0.00170056
NM_000239.2	4069	LYZ	1516	905.233	199.578	2.18134	5.00E-05	0.00170056
NM_199280.2	165186	FAM179A	3548	3.82214	10.5229	-1.46108	5.00E-05	0.00170056
NM_017691.3	54839	LRRC49	2852	7.11655	17.1808	-1.27155	5.00E-05	0.00170056
NM_021073.2	653	BMP5	2172	27.5571	9.13111	1.59356	5.00E-05	0.00170056
TCONS_00218882	XLOC_018599	-	275	0	3.24262	#NAME?	5.00E-05	0.00170056
NM_024593.3	79645	EFCAB1	2144	11.5378	35.1188	-1.60588	5.00E-05	0.00170056
NM_002133.2	3162	HMOX1	1590	19.0023	4.10273	2.21152	5.00E-05	0.00170056
XM_002342853.2	100287651	LOC100287651	2337	7.29777	22.8158	-1.64451	5.00E-05	0.00170056
NM_001007540.2	389118	CDHR4	2476	2.02586	7.31362	-1.85205	5.00E-05	0.00170056
NM_001763.2	909	CD1A	2096	2.13867	0.263223	3.02236	5.00E-05	0.00170056
NM_002259.4	3821	KLRC1	1439	2.49625	9.98725	-2.00032	5.00E-05	0.00170056
NM_001007525.3	284434	NWD1	7645	19.8493	43.0126	-1.11567	5.00E-05	0.00170056
NM_000669.3	126	ADH1C	1473	241.235	106.534	1.17912	5.00E-05	0.00170056
NM_006981.3	8013	NR4A3	5634	32.0708	0.910099	5.13909	5.00E-05	0.00170056
NM_174858.2	26289	AK5	3325	21.9522	4.88983	2.16651	5.00E-05	0.00170056
NM_031938.4	83875	BCO2	2912	2.9009	8.27003	-1.51139	5.00E-05	0.00170056
NM_017423.2	51809	GALNT7	4307	27.9977	11.8354	1.24221	5.00E-05	0.00170056
NM_138796.2	128153	SPATA17	1235	13.7523	45.3862	-1.72258	5.00E-05	0.00170056
NM_178499.3	160777	CCDC60	2450	4.11661	11.6155	-1.49652	5.00E-05	0.00170056
NM_181807.3	341019	DCDC1	1742	4.88631	21.0786	-2.10896	5.00E-05	0.00170056
NM_032866.3	84952	CGNL1	7157	5.08269	11.4595	-1.17288	5.00E-05	0.00170056
NM_001011552.3	389015	SLC9A4	4138	3.07666	7.86574	-1.35422	5.00E-05	0.00170056
NM_002427.3	4322	MMP13	2719	0.874896	159.113	-7.50672	5.00E-05	0.00170056
NM_173672.4	285755	PPIL6	4128	5.46926	20.3886	-1.89835	5.00E-05	0.00170056
NM_001565.3	3627	CXCL10	1216	2.8455	16.4535	-2.53164	5.00E-05	0.00170056
NM_031456.3	10517	FBXW10	3439	0.687069	3.57936	-2.38117	5.00E-05	0.00170056
NM_002429.4	4327	MMP19	3254	13.0461	3.55587	1.87534	5.00E-05	0.00170056
NM_153490.2	3860	KRT13	1715	3.097	0.675297	2.19728	5.00E-05	0.00170056

NM_182493.2	91807	MYLK3	7998	0.309853	1.24804	-2.01001	5.00E-05	0.00170056
NM_024806.2	79864	C11orf63	2911	4.32178	11.4772	-1.40908	5.00E-05	0.00170056
XM_003403447.1	100652938	LOC100652938	5295	15.0552	4.01624	1.90634	5.00E-05	0.00170056
NM_014470.3	27289	RND1	1665	3.59408	0.640076	2.48931	5.00E-05	0.00170056
NM_002976.3	6332	SCN7A	7186	0.456659	3.11981	-2.77227	5.00E-05	0.00170056
NM_001530.3	3091	HIF1A	4059	109.259	37.4411	1.54506	5.00E-05	0.00170056
NM_000953.2	5729	PTGDR	2942	3.84594	11.9824	-1.63951	5.00E-05	0.00170056
NM_005507.2	1072	CFL1	1260	183.278	85.6049	1.09827	5.00E-05	0.00170056
NM_015444.2	25907	TMEM158	1859	3.13113	0.548307	2.51363	5.00E-05	0.00170056
TCONS_00233089	XLOC_020133	-	2857	0.613124	3.05482	-2.31683	5.00E-05	0.00170056
NM_198559.1	375307	C2orf62	1323	1.10026	5.26243	-2.25789	5.00E-05	0.00170056
NM_018965.2	54209	TREM2	1051	11.5915	2.19329	2.40190	5.00E-05	0.00170056
NM_000528.3	4125	MAN2B1	3224	31.3591	14.3094	1.13192	5.00E-05	0.00170056
NM_023036.4	64446	DNAI2	2279	3.21297	11.0471	-1.78169	5.00E-05	0.00170056
NM_172218.2	6674	SPAG1	3749	10.1689	22.9635	-1.17518	5.00E-05	0.00170056
NM_004288.4	9595	CYTIP	2210	59.8032	26.2065	1.19030	5.00E-05	0.00170056
NM_002357.3	4084	MXD1	5617	28.2796	4.50872	2.64897	5.00E-05	0.00170056
NM_019074.3	54567	DLL4	3420	6.17687	2.12183	1.54156	5.00E-05	0.00170056
NM_001781.2	969	CD69	1676	260.814	14.1771	4.20138	5.00E-05	0.00170056
NM_020877.2	146754	DNAH2	13505	3.60199	10.7199	-1.57343	5.00E-05	0.00170056
NM_001124.1	133	ADM	1449	21.7287	2.81998	2.94584	5.00E-05	0.00170056
NM_014429.3	27136	MORC1	3768	1.35126	0.142655	3.24370	5.00E-05	0.00170056
NM_032532.2	84624	FNDC1	6551	1.57238	4.36865	-1.47424	5.00E-05	0.00170056
NM_025145.5	80217	WDR96	5365	18.3587	83.2516	-2.18101	5.00E-05	0.00170056
NM_130830.4	131578	LRRC15	5883	0.928249	4.55081	-2.29354	5.00E-05	0.00170056
NM_133459.3	147372	CCBE1	6260	0.377326	3.31121	-3.13348	5.00E-05	0.00170056
TCONS_00220371	XLOC_019087	-	305	0	2.19441	#NAME?	5.00E-05	0.00170056
NM_001145128.2	221264	AKD1	6326	4.47272	13.4382	-1.58711	5.00E-05	0.00170056
NM_006406.1	10549	PRDX4	921	87.0289	34.3174	1.34255	5.00E-05	0.00170056
NM_001164496.1	55779	WDR52	10259	11.5324	27.2283	-1.23941	5.00E-05	0.00170056
NM_001098843.3	645090	CXorf30	2863	3.5013	15.3101	-2.12852	5.00E-05	0.00170056
NM_018492.2	55872	PBK	1854	5.34837	1.08881	2.29634	5.00E-05	0.00170056
TCONS_00053726	XLOC_003947	-	4699	1.79331	7.38542	-2.04206	5.00E-05	0.00170056
NM_006235.2	5450	POU2AF1	3032	9.76899	3.01664	1.69526	5.00E-05	0.00170056
NM_015417.4	25876	SPEF1	1587	2.90409	8.50344	-1.54996	5.00E-05	0.00170056
NM_005554.3	3853	KRT6A	2450	21.7183	1.69885	3.67628	5.00E-05	0.00170056
NM_012293.1	7837	PXDN	6821	12.625	6.32055	0.99816	5.00E-05	0.00170056
NM_001765.2	911	CD1C	2592	10.6383	2.86812	1.89109	5.00E-05	0.00170056
TCONS_00081893	XLOC_006697	-	5183	1.2034	4.44295	-1.88441	5.00E-05	0.00170056
NM_024430.3	9050	PSTPIP2	3024	15.6929	6.09828	1.36364	5.00E-05	0.00170056
NM_022486.3	64420	SUSD1	2996	20.4848	8.2787	1.30708	5.00E-05	0.00170056
NM_001089584.1	134121	C5orf49	1026	9.71501	34.1739	-1.81461	5.00E-05	0.00170056
NM_000677.3	140	ADORA3	2245	11.4677	2.2349	2.35929	5.00E-05	0.00170056
NM_017539.1	55567	DNAH3	12351	9.27592	26.9087	-1.53651	5.00E-05	0.00170056
NM_145793.3	2674	GFRA1	9134	8.34266	27.8384	-1.73850	5.00E-05	0.00170056
NM_001172129.1	3055	HCK	2155	14.9061	4.50093	1.72761	5.00E-05	0.00170056
NM_000313.3	5627	PROS1	3580	22.5316	60.5745	-1.42676	5.00E-05	0.00170056
NM_001152.4	292	SLC25A5	1351	99.3271	45.4862	1.12676	5.00E-05	0.00170056
NM_182705.2	359845	FAM101B	3622	24.8298	7.04938	1.81650	5.00E-05	0.00170056
NM_005651.2	6999	TDO2	1693	4.21725	0.56747	2.89369	5.00E-05	0.00170056
NM_000640.2	3598	IL13RA2	1373	2.78497	0.485056	2.52144	5.00E-05	0.00170056
NM_182791.2	80125	CCDC33	2104	2.22516	10.9483	-2.29872	5.00E-05	0.00170056
NM_001164436.1	389177	TMEM212	1881	7.27232	25.8788	-1.83128	5.00E-05	0.00170056
NM_000959.3	5737	PTGFR	5360	7.57653	16.833	-1.15168	5.00E-05	0.00170056

NM_001007156.1	4916	NTRK3	3982	1.92985	6.62195	-1.77877	5.00E-05	0.00170056
NM_002928.3	6004	RGS16	2432	23.6673	1.82997	3.69300	5.00E-05	0.00170056
NM_020974.2	57758	SCUBE2	4536	1.66733	6.48128	-1.95874	5.00E-05	0.00170056
NM_020893.2	100499483	C9orf174	5635	5.77593	14.0872	-1.28626	5.00E-05	0.00170056
NM_032119.3	84059	GPR98	19333	3.9328	18.2589	-2.21497	5.00E-05	0.00170056
NM_001814.4	1075	CTSC	1907	359.574	63.5718	2.49983	5.00E-05	0.00170056
NM_031422.4	83539	CHST9	2246	49.8727	118.083	-1.24347	5.00E-05	0.00170056
NM_000916.3	5021	OXTR	4361	46.165	13.7939	1.74277	5.00E-05	0.00170056
NM_152327.2	122481	AK7	2648	12.4514	33.1068	-1.41082	5.00E-05	0.00170056
XM_003403451.1	100508736	LOC100508736	1567	5.48834	19.0839	-1.79792	5.00E-05	0.00170056
NM_002029.3	2357	FPR1	1317	15.3973	2.93228	2.39258	5.00E-05	0.00170056
NM_001254.3	990	CDC6	3053	2.98069	0.813722	1.87304	5.00E-05	0.00170056
NM_199327.1	10252	SPRY1	2348	63.8487	23.0045	1.47274	5.00E-05	0.00170056
NM_006169.2	4837	NNMT	1578	37.273	9.3379	1.99696	5.00E-05	0.00170056
NM_147195.2	253650	ANKRD18A	3738	6.18273	14.4507	-1.22483	5.00E-05	0.00170056
NM_153362.2	167681	PRSS35	2503	1.18412	5.56998	-2.23386	5.00E-05	0.00170056
NM_030782.3	81037	CLPTM1L	2148	42.3752	20.7687	1.02881	5.00E-05	0.00170056
NM_025132.3	57728	WDR19	4520	12.4784	26.7533	-1.10029	5.00E-05	0.00170056
NM_003373.3	7414	VCL	5286	61.1001	28.3476	1.10795	5.00E-05	0.00170056
NM_147168.1	84688	C9orf24	712	14.3379	52.5111	-1.87279	5.00E-05	0.00170056
NM_001136570.1	100129583	FAM47E	1550	5.55954	14.9665	-1.42870	5.00E-05	0.00170056
NM_021254.2	56683	C21orf59	1427	32.1281	64.5586	-1.00677	5.00E-05	0.00170056
NM_199166.1	211	ALAS1	2258	45.5571	15.8019	1.52758	5.00E-05	0.00170056
TCONS_00207680	XLOC_017712	-	4806	4.42183	0.492154	3.16746	5.00E-05	0.00170056
NM_001031716.2	64859	OBFC2A	3849	56.9536	22.2308	1.35723	5.00E-05	0.00170056
NM_144577.3	93233	CCDC114	3220	2.15727	9.73158	-2.17346	5.00E-05	0.00170056
NM_152732.4	221421	RSPH9	962	6.50942	19.4198	-1.57693	5.00E-05	0.00170056
NM_001195811.1	200150	PLD5	3018	0.587491	7.00164	-3.57505	5.00E-05	0.00170056
NM_001884.3	1404	HAPLN1	4678	1.84705	6.04425	-1.71034	5.00E-05	0.00170056
NM_025153.2	23120	ATP10B	7566	2.20884	0.633624	1.80159	5.00E-05	0.00170056
NM_016644.1	51334	PRR16	1764	2.36126	0.422426	2.48279	5.00E-05	0.00170056
NM_001085447.1	129881	C2orf77	2169	6.0993	21.6062	-1.82473	5.00E-05	0.00170056
NM_001080508.1	9096	TBX18	4070	2.05212	5.73194	-1.48191	5.00E-05	0.00170056
TCONS_00154706	XLOC_014975	-	16334	1.49395	6.16537	-2.04506	5.00E-05	0.00170056
NM_001080534.1	440279	UNC13C	8140	1.18901	4.96343	-2.06158	5.00E-05	0.00170056
NM_022073.3	112399	EGLN3	2722	22.4964	6.64	1.76044	5.00E-05	0.00170056
NM_005630.2	6578	SLCO2A1	4223	5.5696	2.19121	1.34585	5.00E-05	0.00170056
NM_024753.3	79809	TTC21B	4896	16.9501	36.2129	-1.09521	5.00E-05	0.00170056
NM_002922.3	5996	RGS1	1403	201.782	19.2747	3.38802	5.00E-05	0.00170056
NM_001080537.1	132203	SNTN	1586	40.4648	154.448	-1.93238	5.00E-05	0.00170056
NM_002985.2	6352	CCL5	1230	43.0121	110.747	-1.36446	5.00E-05	0.00170056
NM_152367.2	126868	MAB21L3	3229	14.9894	37.8134	-1.33495	5.00E-05	0.00170056
NM_002864.2	5858	PZP	4610	2.85142	10.6603	-1.90249	5.00E-05	0.00170056
NM_174975.4	266629	SEC14L3	2084	10.9827	54.4952	-2.31090	5.00E-05	0.00170056
NM_006183.4	4922	NTS	1239	61.706	20.3172	1.60271	5.00E-05	0.00170056
NM_005354.4	3727	JUND	1870	42.6208	15.8948	1.42300	5.00E-05	0.00170056
NM_004065.2	1038	CDR1	1299	8.47655	40.7513	-2.26530	5.00E-05	0.00170056
NM_170776.4	222487	GPR97	2670	6.75829	0.353209	4.25806	5.00E-05	0.00170056
NM_001263.3	1040	CDS1	4538	14.0527	32.1531	-1.19411	5.00E-05	0.00170056
NM_006108.2	10418	SPON1	5384	52.9175	16.66	1.66735	5.00E-05	0.00170056
NM_030906.2	65975	STK33	2707	8.00722	29.7608	-1.89404	5.00E-05	0.00170056
NM_145071.2	1154	CISH	2111	10.8068	1.84172	2.55282	5.00E-05	0.00170056
NM_032411.2	84417	C2orf40	784	5.69538	22.1256	-1.95786	5.00E-05	0.00170056
NM_005978.3	6273	S100A2	963	129.743	23.2854	2.47816	5.00E-05	0.00170056

NM_152548.2	153643	FAM81B	1563	13.7941	48.326	-1.80875	5.00E-05	0.00170056
NM_178483.2	140856	C20orf79	667	0	0.740227	#NAME?	5.00E-05	0.00170056
NM_017625.2	55600	ITLN1	1182	27.4677	2.54582	3.43153	5.00E-05	0.00170056
NM_004258.3	9398	CD101	3547	6.52588	2.13291	1.61335	5.00E-05	0.00170056
NM_024329.5	79180	EFHD2	2419	29.5684	12.1345	1.28494	5.00E-05	0.00170056
NM_005114.2	9957	HS3ST1	1965	25.9978	6.63289	1.97068	5.00E-05	0.00170056
28526	28526	-	1510	8.43269	2.50311	1.75227	5.00E-05	0.00170056
NM_022097.2	63928	CHP2	2366	2.0191	12.9581	-2.68206	5.00E-05	0.00170056
3494	3494	-	1019	726.672	93.4102	2.95965	5.00E-05	0.00170056
NM_006379.3	10512	SEMA3C	5174	30.4788	60.0215	-0.97767	5.00E-05	0.00170056
NM_001048265.1	138162	C9orf116	722	15.5363	43.0468	-1.47026	5.00E-05	0.00170056
NM_178824.3	151790	WDR49	2594	12.7196	46.9505	-1.88409	5.00E-05	0.00170056
NM_152325.1	122046	C13orf26	998	13.8285	45.9125	-1.73124	5.00E-05	0.00170056
NM_198285.2	349136	WDR86	2041	8.14617	25.095	-1.62320	5.00E-05	0.00170056
NM_178550.4	339512	C1orf110	1552	13.6729	39.6337	-1.53541	5.00E-05	0.00170056
NM_024721.4	79776	ZFHX4	13958	3.27587	9.54205	-1.54242	5.00E-05	0.00170056
NM_004944.2	1776	DNASE1L3	1463	5.40095	19.5908	-1.85889	5.00E-05	0.00170056
NM_024422.3	1824	DSC2	5199	12.3131	2.49327	2.30409	5.00E-05	0.00170056
NM_014957.2	22898	DENND3	5444	8.37843	3.99823	1.06732	5.00E-05	0.00170056
NM_001077418.1	79583	TMEM231	2891	12.4892	30.8602	-1.30506	5.00E-05	0.00170056
NM_012301.3	9863	MAGI2	6880	2.93304	6.31978	-1.10748	5.00E-05	0.00170056
NM_005306.2	2867	FFAR2	2053	9.19102	0.522918	4.13557	5.00E-05	0.00170056
NM_025159.2	80231	CXorf21	1927	9.49256	3.16143	1.58622	5.00E-05	0.00170056
TCONS_00293562	XLOC_025465	-	11650	0.61966	3.32453	-2.42360	5.00E-05	0.00170056
NM_173354.3	150094	SIK1	4706	45.1877	4.40303	3.35936	5.00E-05	0.00170056
NM_001099677.1	57604	KIAA1456	9300	3.24096	9.92966	-1.61532	5.00E-05	0.00170056
NM_174896.2	128346	C1orf162	917	23.6861	6.99427	1.75980	5.00E-05	0.00170056
NM_001792.3	1000	CDH2	4367	1.61473	5.61369	-1.79766	5.00E-05	0.00170056
NM_004588.4	6327	SCN2B	4922	0.499338	1.60203	-1.68181	5.00E-05	0.00170056
NM_001101312.1	28959	TMEM176B	1298	57.5183	19.574	1.55508	5.00E-05	0.00170056
NM_006269.1	6101	RP1	7100	6.13558	18.6022	-1.60020	5.00E-05	0.00170056
NM_016270.2	10365	KLF2	1655	26.6601	8.23676	1.69453	5.00E-05	0.00170056
NM_173615.3	146177	VWA3A	4600	3.97837	11.1983	-1.49303	5.00E-05	0.00170056
NM_001558.3	3587	IL10RA	3656	24.6936	8.54456	1.53106	5.00E-05	0.00170056
NM_000211.3	3689	ITGB2	2958	37.2093	12.9395	1.52388	5.00E-05	0.00170056
NM_022785.3	64800	EFCAB6	4929	3.73487	11.1985	-1.58417	5.00E-05	0.00170056
NM_005252.3	2353	FOS	2158	486.04	7.21553	6.07382	5.00E-05	0.00170056
NM_017950.2	55036	CCDC40	4294	2.94344	8.18152	-1.47486	5.00E-05	0.00170056
NM_000576.2	3553	IL1B	1498	3.1396	0.462931	2.76171	5.00E-05	0.00170056
NM_001099697.1	728194	RSPH10B2	3106	0.805435	3.44001	-2.09457	5.00E-05	0.00170056
NM_006763.2	7832	BTG2	2712	210.617	46.769	2.17100	5.00E-05	0.00170056
NM_024763.4	79819	WDR78	3848	15.1305	49.2454	-1.70253	5.00E-05	0.00170056
NM_030810.3	81567	TXNDC5	3231	313.722	108.516	1.53158	5.00E-05	0.00170056
NM_002457.2	4583	MUC2	8623	1.39396	0.208277	2.74262	5.00E-05	0.00170056
NM_000146.3	2512	FTL	871	1494.3	576.283	1.37462	5.00E-05	0.00170056
NM_000766.3	1553	CYP2A13	1747	5.61842	19.9971	-1.83156	5.00E-05	0.00170056
NM_003486.5	8140	SLC7A5	4543	2.58157	0.764083	1.75645	5.00E-05	0.00170056
NM_032525.1	84617	TUBB6	1815	22.4238	9.817	1.19168	5.00E-05	0.00170056
NM_005290.1	2838	GPR15	1083	5.75576	0.753787	2.93278	5.00E-05	0.00170056
NM_006875.3	11040	PIM2	2187	61.1768	7.95323	2.94337	5.00E-05	0.00170056
NM_153221.2	148113	CILP2	4199	0.0646116	2.31852	-5.16527	5.00E-05	0.00170056
NM_021103.3	9168	TMSB10	482	1244.13	601.074	1.04952	5.00E-05	0.00170056
NM_021902.2	5348	FXYD1	513	3.476	16.6008	-2.25576	5.00E-05	0.00170056
NM_024783.3	79841	AGBL2	3593	4.97866	16.3753	-1.71769	5.00E-05	0.00170056

NM_004077.2	1431	CS	2976	36.9664	19.2655	0.94019	5.00E-05	0.00170056
NM_001199201.1	79740	ZBBX	3363	13.8194	62.7037	-2.18186	5.00E-05	0.00170056
NM_182508.2	144809	C13orf30	3302	35.1799	131.98	-1.90750	5.00E-05	0.00170056
NM_001085.4	12	SERPINA3	1590	469.152	14.9471	4.97211	5.00E-05	0.00170056
TCONS_00070151	XLOC_006346	-	298	3.1271	0	inf	5.00E-05	0.00170056
NM_001206485.1	467	ATF3	3172	15.2178	0.950699	4.00063	5.00E-05	0.00170056
NM_145278.3	148823	C1orf150	2626	3.94641	0.969398	2.02538	5.00E-05	0.00170056
NM_020672.1	57402	S100A14	1043	36.358	13.6965	1.40847	5.00E-05	0.00170056
NM_207647.2	83856	FSD1L	4295	5.24929	13.64	-1.37765	5.00E-05	0.00170056
NM_000698.2	240	ALOX5	2554	21.4268	6.86329	1.64244	5.00E-05	0.00170056
NM_014648.3	9666	DZIP3	5542	20.1262	41.8695	-1.05683	5.00E-05	0.00170056
NM_017576.1	55582	KIF27	4653	13.1358	30.8192	-1.23033	5.00E-05	0.00170056
NM_006088.5	10383	TUBB2C	1591	115.61	282.231	-1.28761	5.00E-05	0.00170056
NM_005655.2	7071	KLF10	2952	34.0612	15.306	1.15404	5.00E-05	0.00170056
NM_002089.3	2920	CXCL2	1205	67.4621	1.4232	5.56687	5.00E-05	0.00170056
NM_000949.5	5618	PRLR	11817	4.6013	15.3758	-1.74054	5.00E-05	0.00170056
NM_001010892.2	345895	RSPH4A	2840	19.0418	66.7788	-1.81022	5.00E-05	0.00170056
NM_020817.1	57577	KIAA1407	3976	6.68479	15.1402	-1.17943	5.00E-05	0.00170056
XM_003403420.1	100652824	LOC100652824	2446	4.16815	11.9282	-1.51689	5.00E-05	0.00170056
NM_012337.2	25790	CCDC19	1795	10.0455	36.9839	-1.88036	5.00E-05	0.00170056
NM_001898.2	1469	CST1	760	985.484	0.853001	10.17410	5.00E-05	0.00170056
NM_003537.3	8358	HIST1H3B	472	50.0093	15.5455	1.68570	5.00E-05	0.00170056
NM_001442.2	2167	FABP4	838	4.04084	34.3176	-3.08622	5.00E-05	0.00170056
NM_001145450.1	729967	MORN2	741	43.5044	120.284	-1.46721	5.00E-05	0.00170056
NM_000668.4	125	ADH1B	2682	1.79125	10.3051	-2.52432	5.00E-05	0.00170056
NM_001974.3	2015	EMR1	3123	19.6724	0.317868	5.95161	5.00E-05	0.00170056
NM_004040.2	388	RHOB	2367	151.131	50.4514	1.58283	5.00E-05	0.00170056
NM_003105.5	6653	SORL1	10973	52.9616	25.5343	1.05251	5.00E-05	0.00170056
NM_058180.3	54058	C21orf58	2968	2.206	5.67624	-1.36350	5.00E-05	0.00170056
NM_001190766.1	401236	FLJ23152	1322	4.34761	20.5359	-2.23985	5.00E-05	0.00170056
NM_014220.2	4071	TM4SF1	1691	88.6085	43.5195	1.02578	5.00E-05	0.00170056
TCONS_00062536	XLOC_005694	-	16273	8.1145	2.13204	1.92826	5.00E-05	0.00170056
NM_138426.2	113263	GLCCI1	4745	16.8508	7.93569	1.08639	5.00E-05	0.00170056
NM_015567.1	26050	SLITRK5	4447	0.440055	1.84651	-2.06905	5.00E-05	0.00170056
NM_144992.4	200403	VWA3B	4747	5.95631	18.6574	-1.64726	5.00E-05	0.00170056
NM_080283.3	10350	ABCA9	6301	3.89484	10.4484	-1.42365	5.00E-05	0.00170056
NM_148975.1	51338	MS4A4A	1524	37.2877	15.2907	1.28605	5.00E-05	0.00170056
NM_016529.4	51761	ATP8A2	5006	4.69777	0.805169	2.54461	5.00E-05	0.00170056
NM_031457.1	83661	MS4A8B	1353	15.5796	72.4867	-2.21806	5.00E-05	0.00170056
NM_000573.3	1378	CR1	8616	13.4739	1.75544	2.94027	5.00E-05	0.00170056
NM_031421.2	83538	TTC25	2078	6.28726	19.8752	-1.66047	5.00E-05	0.00170056
NM_173554.2	219621	C10orf107	1252	16.1576	39.0965	-1.27482	5.00E-05	0.00170056
NM_003469.4	7857	SCG2	2572	88.0674	20.8374	2.07944	5.00E-05	0.00170056
NM_001012502.2	286207	C9orf117	1722	5.48466	28.102	-2.35720	5.00E-05	0.00170056
NM_000574.3	1604	CD55	2796	68.7664	21.4055	1.68372	5.00E-05	0.00170056
NM_000204.3	3426	CFI	2161	127.844	59.5322	1.10264	5.00E-05	0.00170056
NM_001031715.2	64799	IQCH	4239	2.66142	7.52865	-1.50019	5.00E-05	0.00170056
NM_016010.2	51101	FAM164A	3352	14.1508	28.8579	-1.02809	5.00E-05	0.00170056
NM_003613.3	8483	CILP	4468	0.386228	1.67811	-2.11931	5.00E-05	0.00170056
NM_017617.3	4851	NOTCH1	9295	8.52912	4.21805	1.01582	5.00E-05	0.00170056
NM_004344.1	1069	CETN2	1080	73.5583	198.216	-1.43012	5.00E-05	0.00170056
NM_173808.2	257194	NEGR1	5669	2.27603	5.74145	-1.33490	5.00E-05	0.00170056
NM_004826.2	9427	ECEL1	2859	1.96723	0.19955	3.30135	5.00E-05	0.00170056
NM_001029996.3	200373	PCDP1	2968	6.57611	20.5304	-1.64245	5.00E-05	0.00170056

NM_003139.3	6734	SRPR	3092	38.4817	20.0113	0.94336	5.00E-05	0.00170056
NM_032704.3	84790	TUBA1C	1553	38.9999	14.6569	1.41189	5.00E-05	0.00170056
NM_001008226.1	283726	FAM154B	3131	13.4542	48.7466	-1.85724	5.00E-05	0.00170056
NM_004599.2	6721	SREBF2	4306	20.4238	9.9371	1.03935	5.00E-05	0.00170056
NM_006072.4	10344	CCL26	562	91.7771	3.7565	4.61067	5.00E-05	0.00170056
NM_178452.4	123872	DNAAF1	2436	12.3997	40.303	-1.70058	5.00E-05	0.00170056
NM_003277.3	7122	CLDN5	1702	15.9801	4.58989	1.79975	5.00E-05	0.00170056
NM_004744.3	9227	LRAT	4888	0.144844	7.35898	-5.66694	5.00E-05	0.00170056
NM_182758.2	256764	WDR72	7309	1.47999	5.5291	-1.90146	5.00E-05	0.00170056
NM_004061.3	1010	CDH12	4345	0.634424	2.5427	-2.00284	5.00E-05	0.00170056
XM_001715090.3	144535	C12orf55	5721	7.68912	29.9599	-1.96214	5.00E-05	0.00170056
NM_018690.2	55911	APOBR	3733	5.62036	1.6302	1.78562	5.00E-05	0.00170056
NM_000694.2	221	ALDH3B1	2815	12.1305	37.5336	-1.62955	5.00E-05	0.00170056
TCONS_00156810	XLOC_015004	-	2828	1.59004	4.75247	-1.57962	5.00E-05	0.00170056
NM_001105556.1	9473	C1orf38	2723	16.0843	4.60949	1.80297	5.00E-05	0.00170056
NM_015101.2	23127	GLT25D2	5182	1.03445	3.51641	-1.76524	5.00E-05	0.00170056
NM_018136.4	259266	ASPM	10888	4.61777	1.98663	1.21687	5.00E-05	0.00170056
XM_002343299.3	319089	TTC6	2913	6.0073	14.5005	-1.27132	5.00E-05	0.00170056
NM_015714.3	50486	G0S2	963	16.3631	3.30018	2.30983	5.00E-05	0.00170056
NM_025059.3	80129	C6orf97	5283	19.8813	49.2414	-1.30846	5.00E-05	0.00170056
NM_001428.3	2023	ENO1	2187	172.998	74.8777	1.20815	5.00E-05	0.00170056
NM_024726.3	79781	IQCA1	3188	5.35544	12.6918	-1.24482	5.00E-05	0.00170056
NM_012072.3	22918	CD93	6701	46.816	16.534	1.50157	5.00E-05	0.00170056
NM_001145077.1	390205	LRRC10B	2219	5.45324	15.9905	-1.55203	5.00E-05	0.00170056
NM_007050.5	11122	PTPRT	12637	0.840909	3.06933	-1.86790	5.00E-05	0.00170056
NM_019050.2	54532	USP53	6595	32.0426	91.3625	-1.51161	5.00E-05	0.00170056
NM_001964.2	1958	EGR1	3136	73.3271	4.00823	4.19331	5.00E-05	0.00170056
TCONS_00223119	XLOC_019117	-	1701	1.20423	6.32961	-2.39401	5.00E-05	0.00170056
NM_004233.3	9308	CD83	2478	16.9528	3.93296	2.10783	5.00E-05	0.00170056
NM_001321.1	1466	CSRP2	901	68.4496	290.382	-2.08484	5.00E-05	0.00170056
NM_172239.4	254958	REXO1L1	7032	3.62701	24.4886	-2.75526	5.00E-05	0.00170056
NM_001432.2	2069	EREG	4628	6.58287	0.223087	4.88304	5.00E-05	0.00170056
NM_020130.3	56892	C8orf4	1833	124.593	15.0847	3.04607	5.00E-05	0.00170056
NM_181426.1	339829	CCDC39	3854	10.2866	30.2295	-1.55519	5.00E-05	0.00170056
NM_020802.2	57562	KIAA1377	7043	6.8155	20.8934	-1.61616	5.00E-05	0.00170056
NM_032269.5	84229	CCDC135	2911	2.35336	8.51123	-1.85465	5.00E-05	0.00170056
NM_000129.3	2162	F13A1	3863	283.732	50.3977	2.49310	5.00E-05	0.00170056
NM_002416.1	4283	CXCL9	2545	2.33524	12.9622	-2.47267	5.00E-05	0.00170056
NM_002275.3	3866	KRT15	1840	114.291	40.1227	1.51021	5.00E-05	0.00170056
NM_145235.3	92565	FANK1	1296	13.2293	31.9607	-1.27257	5.00E-05	0.00170056
TCONS_00047584	XLOC_003947	-	3909	3.15883	14.2751	-2.17604	5.00E-05	0.00170056
NM_001018071.3	143162	FRMPD2	5024	2.91976	10.6401	-1.86558	5.00E-05	0.00170056
NM_001099294.1	85352	KIAA1644	6741	0.722345	3.00503	-2.05662	5.00E-05	0.00170056
NM_032160.2	92126	DSEL	9281	2.92292	6.57699	-1.17001	5.00E-05	0.00170056
NM_138451.1	115811	IQCD	1350	4.24084	15.1969	-1.84136	5.00E-05	0.00170056
TCONS_00196639	XLOC_017787	-	3867	0.921229	5.21085	-2.49989	5.00E-05	0.00170056
NM_052947.3	115701	ALPK2	7303	6.96329	0.905442	2.94308	5.00E-05	0.00170056
NM_004681.2	9086	EIF1AY	1390	24.3086	6.92326	1.81194	5.00E-05	0.00170056
NM_198320.3	1368	CPM	6655	8.65585	3.88827	1.15455	5.00E-05	0.00170056
NM_001369.2	1767	DNAH5	15573	14.6696	86.2259	-2.55529	5.00E-05	0.00170056
NM_024787.2	79845	RNF122	1868	9.51535	2.84717	1.74073	5.00E-05	0.00170056
NM_001017424.2	3776	KCNK2	3597	0.464832	4.18624	-3.17087	5.00E-05	0.00170056
3539	3539	-	318	646.944	166.569	1.95752	5.00E-05	0.00170056
3538	3538	-	320	2118.9	391.077	2.43779	5.00E-05	0.00170056

NM_004106.1	2207	FCER1G	591	56.3504	20.4269	1.46396	5.00E-05	0.00170056
NM_014432.2	53832	IL20RA	3682	3.56984	8.83636	-1.30759	5.00E-05	0.00170056
NM_016232.4	9173	IL1RL1	2058	21.8276	4.43645	2.29867	5.00E-05	0.00170056
NM_194302.2	255101	CCDC108	5945	1.19487	5.20581	-2.12327	5.00E-05	0.00170056
NM_004751.2	9245	GCNT3	2222	8.69937	1.53688	2.50091	5.00E-05	0.00170056
NM_197965.2	345274	SLC10A6	1502	5.36734	0.869628	2.62574	5.00E-05	0.00170056
NM_000442.4	5175	PECAM1	4449	36.6826	13.0084	1.49566	5.00E-05	0.00170056
NM_001102608.1	131873	COL6A6	8470	6.18578	2.25225	1.45759	5.00E-05	0.00170056
NM_014375.2	26998	FETUB	1627	4.32611	0.179476	4.59121	5.00E-05	0.00170056
NM_004418.3	1844	DUSP2	1685	13.1242	2.1462	2.61238	5.00E-05	0.00170056
NM_001039112.2	654463	FER1L6	5890	16.5655	8.05787	1.03971	5.00E-05	0.00170056
NM_002664.2	5341	PLEK	2852	35.5301	12.9319	1.45811	5.00E-05	0.00170056
NM_181643.4	128344	C1orf88	2346	18.504	68.8523	-1.89567	5.00E-05	0.00170056
NM_002005.3	2242	FES	2783	10.3519	3.7547	1.46312	5.00E-05	0.00170056
NM_005559.3	284217	LAMA1	9657	2.7555	1.10972	1.31212	5.00E-05	0.00170056
NM_173081.3	219681	ARMC3	2808	14.6867	56.0279	-1.93164	5.00E-05	0.00170056
NM_001769.3	928	CD9	1314	232.588	115.582	1.00886	5.00E-05	0.00170056
NM_002923.3	5997	RGS2	1350	282.433	16.1875	4.12495	5.00E-05	0.00170056
NM_000632.3	3684	ITGAM	4742	23.0795	3.5118	2.71633	5.00E-05	0.00170056
NM_152290.2	93190	C1orf158	1019	3.24005	13.1781	-2.02405	5.00E-05	0.00170056
NM_213606.3	387700	SLC16A12	4622	3.39611	19.0353	-2.48672	5.00E-05	0.00170056
NM_033050.4	56670	SUCNR1	1650	12.2573	1.22793	3.31934	5.00E-05	0.00170056
NM_024790.6	79848	CSPP1	4718	13.9441	28.1323	-1.01257	5.00E-05	0.00170056
NM_006743.4	5935	RBM3	4432	83.635	32.7683	1.35181	5.00E-05	0.00170056
NM_198552.2	375061	FAM89A	1498	9.85642	2.97647	1.72746	5.00E-05	0.00170056
NM_198491.1	339145	FAM92B	1897	7.07712	18.7218	-1.40349	5.00E-05	0.00170056
NM_206996.2	200162	SPAG17	7125	7.84834	32.7739	-2.06209	5.00E-05	0.00170056
NM_175610.2	7082	TJP1	6925	17.6747	54.4488	-1.62321	5.00E-05	0.00170056
NM_000399.3	1959	EGR2	2979	11.0657	0.558829	4.30754	5.00E-05	0.00170056
NM_001035.2	6262	RYR2	16365	1.49442	3.63456	-1.28220	5.00E-05	0.00170056
NM_001372.3	1770	DNAH9	13750	6.92764	24.7797	-1.83872	5.00E-05	0.00170056
TCONS_00111412	XLOC_009731	-	2716	0.808264	9.19673	-3.50822	5.00E-05	0.00170056
NM_130445.2	80781	COL18A1	5391	22.0442	6.12056	1.84866	5.00E-05	0.00170056
NM_001011657.3	84460	ZMAT1	3489	8.54761	18.0936	-1.08189	5.00E-05	0.00170056
NM_001142966.1	80000	GREB1L	6052	1.16724	5.39672	-2.20898	5.00E-05	0.00170056
NM_080860.2	89765	RSPH1	1367	24.2134	91.1443	-1.91235	5.00E-05	0.00170056
TCONS_00311623	XLOC_026432	-	2466	3.2772	0.122291	4.74408	5.00E-05	0.00170056
NM_153228.2	162282	ANKFN1	2426	3.15844	10.9039	-1.78755	5.00E-05	0.00170056
NM_145244.3	115265	DDIT4L	2634	3.93055	14.2051	-1.85361	5.00E-05	0.00170056
NM_003294.3	7177	TPSAB1	1194	31.0668	7.79991	1.99384	5.00E-05	0.00170056
NM_004683.4	9104	RGN	1615	1.45765	9.81361	-2.75114	5.00E-05	0.00170056
NM_015429.3	25890	ABI3BP	4473	7.94241	16.7159	-1.07357	5.00E-05	0.00170056
NM_174897.2	128859	BPIL3	1362	12.3393	0	inf	5.00E-05	0.00170056
NM_032133.4	84073	MYCBPAP	3190	1.12218	4.22123	-1.91136	5.00E-05	0.00170056
NM_005328.2	3037	HAS2	3275	6.21144	1.47874	2.07056	5.00E-05	0.00170056
NM_001100388.1	399949	C11orf88	714	23.0691	119.6	-2.37419	5.00E-05	0.00170056
NM_177980.2	60437	CDH26	3193	59.9684	4.95779	3.59643	5.00E-05	0.00170056
NM_016230.3	51167	CYB5R4	2247	26.2549	11.1377	1.23713	5.00E-05	0.00170056
NM_152860.1	121340	SP7	2974	0.15683	2.13551	-3.76731	5.00E-05	0.00170056
NM_145243.3	115209	OMA1	1909	27.9154	54.4779	-0.96461	5.00E-05	0.00170056
NM_001004303.4	199920	C1orf168	3460	2.06803	10.1833	-2.29988	5.00E-05	0.00170056
NM_022093.1	63923	TNN	5008	0.688977	2.94223	-2.09438	5.00E-05	0.00170056
NM_006536.5	9635	CLCA2	4025	9.59893	2.5704	1.90088	5.00E-05	0.00170056
NM_031310.1	83483	PLVAP	2276	34.2818	12.0634	1.50680	5.00E-05	0.00170056

NM_020546.2	108	ADCY2	6553	2.7012	9.7531	-1.85226	5.00E-05	0.00170056
NM_138806.3	131450	CD200R1	2272	18.6071	4.05556	2.19788	5.00E-05	0.00170056
NM_004417.3	1843	DUSP1	2024	356.027	27.4142	3.69899	5.00E-05	0.00170056
NM_002578.3	5063	PAK3	2754	1.69967	6.02646	-1.82606	5.00E-05	0.00170056
NM_001128148.1	7037	TFRC	5083	39.0907	15.7885	1.30795	5.00E-05	0.00170056
NM_014918.4	22856	CHSY1	4550	32.3763	14.3278	1.17613	5.00E-05	0.00170056
NM_007072.2	11148	HHLA2	2660	2.21665	7.80557	-1.81612	5.00E-05	0.00170056
NM_006332.3	10437	IFI30	1032	108.255	25.0932	2.10906	5.00E-05	0.00170056
NM_024694.3	79747	C6orf103	5290	4.22462	14.1751	-1.74647	5.00E-05	0.00170056
NM_002986.2	6356	CCL11	925	26.4263	2.42561	3.44556	5.00E-05	0.00170056
TCONS_00143182	XLOC_012967	-	403	4.05286	39.6362	-3.28981	5.00E-05	0.00170056
NM_006469.4	10625	IVNS1ABP	4199	125.692	49.9464	1.33144	5.00E-05	0.00170056
NM_006288.3	7070	THY1	2397	56.7715	27.7046	1.03504	5.00E-05	0.00170056
NM_033143.2	2250	FGF5	5285	2.41944	5.97552	-1.30439	5.00E-05	0.00170056
NM_001736.3	728	C5AR1	2342	15.3561	3.21669	2.25516	5.00E-05	0.00170056
NM_017709.3	54855	FAM46C	5720	61.9506	27.8846	1.15165	5.00E-05	0.00170056
NM_001062.3	6947	TCN1	1567	227.086	24.9139	3.18821	5.00E-05	0.00170056
NM_001003845.2	389058	SP5	1947	0.499975	2.53688	-2.34313	5.00E-05	0.00170056
NM_004795.3	9365	KL	5006	4.16757	1.61424	1.36835	5.00E-05	0.00170056
NM_013377.3	29951	PDZRN4	3243	0.229262	3.19894	-3.80253	5.00E-05	0.00170056
NM_004787.1	9353	SLIT2	4950	9.02891	55.0618	-2.60843	5.00E-05	0.00170056
NM_172337.1	5015	OTX2	2069	11.9891	2.2998	2.38214	5.00E-05	0.00170056
NM_002162.3	3385	ICAM3	1780	19.6828	7.05418	1.48038	5.00E-05	0.00170056
NM_145010.2	219670	ENKUR	3318	7.58547	29.2479	-1.94702	5.00E-05	0.00170056
NM_000250.1	4353	MPO	3215	0.112594	0.94959	-3.07618	5.00E-05	0.00170056
TCONS_00095311	XLOC_007699	-	5854	0.553437	1.88318	-1.76668	5.00E-05	0.00170056
TCONS_00046409	XLOC_004317	-	8317	0.41225	1.6632	-2.01237	5.00E-05	0.00170056
NM_018226.4	57140	RNPEPL1	3018	9.47805	4.1952	1.17585	5.00E-05	0.00170056
NM_144702.2	149499	LRRC71	1971	2.20457	6.77299	-1.61929	5.00E-05	0.00170056
NM_001140.3	246	ALOX15	2684	641.058	133.916	2.25913	5.00E-05	0.00170056
3501	3501	-	980	488.109	85.1202	2.51963	5.00E-05	0.00170056
3503	3503	-	983	3703.86	21.8126	7.40772	5.00E-05	0.00170056
3505	3505	-	1188	11.741	0.520062	4.49673	5.00E-05	0.00170056
NM_001004431.1	284207	METRNL	1333	27.9765	10.6392	1.39482	5.00E-05	0.00170056
NM_001377.2	79659	DYNC2H1	13678	22.3651	55.5998	-1.31383	5.00E-05	0.00170056
NM_006290.2	7128	TNFAIP3	4432	54.1129	15.3184	1.82071	5.00E-05	0.00170056
NM_001002796.2	79772	MCTP1	3613	26.7032	14.0251	0.92901	5.00E-05	0.00170056
NM_001173463.1	55605	KIF21A	6530	23.7067	50.1285	-1.08034	5.00E-05	0.00170056
NM_000572.2	3586	IL10	1629	8.45793	0.584201	3.85577	5.00E-05	0.00170056
NM_000887.3	3687	ITGAX	4649	9.69334	3.13523	1.62842	5.00E-05	0.00170056
NM_001195037.2	345930	ECT2L	4343	7.36833	31.9083	-2.11452	5.00E-05	0.00170056
NM_003856.2	9173	IL1RL1	2508	34.9321	3.82156	3.19232	5.00E-05	0.00170056
NM_173549.2	203111	C8orf47	1550	8.76943	26.9158	-1.61790	5.00E-05	0.00170056
NM_007115.3	7130	TNFAIP6	1424	10.3747	1.43809	2.85083	5.00E-05	0.00170056
NM_002266.2	3838	KPNA2	1980	20.5454	7.89084	1.38057	5.00E-05	0.00170056
NM_018557.2	53353	LRP1B	16531	6.23523	31.27	-2.32627	5.00E-05	0.00170056
NM_152447.3	145581	LRFN5	3732	1.26236	5.45421	-2.11124	5.00E-05	0.00170056
NM_152632.3	170063	CXorf22	3613	7.50052	28.8899	-1.94550	5.00E-05	0.00170056
NM_000584.3	3576	IL8	1705	17.5023	4.72844	1.88811	5.00E-05	0.00170056
NM_001025370.2	7422	VEGFA	3407	13.3736	3.15868	2.08199	5.00E-05	0.00170056
NM_000783.3	1592	CYP26A1	2119	1.00104	6.28125	-2.64956	5.00E-05	0.00170056
NM_005415.4	6574	SLC20A1	3379	35.341	17.1992	1.03900	5.00E-05	0.00170056
NM_002001.2	2205	FCER1A	1165	35.7487	8.02895	2.15461	5.00E-05	0.00170056
NM_002575.2	5055	SERPINB2	1908	17.0978	2.09067	3.03177	5.00E-05	0.00170056

NM_002569.2	5045	FURIN	4180	12.5931	5.6162	1.16497	5.00E-05	0.00170056
TCONS_00053150	XLOC_004440	-	13299	2.35416	0.555141	2.08429	5.00E-05	0.00170056
NM_005322.2	3009	HIST1H1B	790	61.894	20.4327	1.59892	5.00E-05	0.00170056
NM_198428.2	27241	BBS9	4019	12.957	27.5062	-1.08603	5.00E-05	0.00170056
NM_003045.4	6541	SLC7A1	7343	12.3015	5.38778	1.19107	5.00E-05	0.00170056
NM_001098212.1	3269	HRH1	4278	8.42974	2.1195	1.99176	5.00E-05	0.00170056
NM_001195610.1	51473	DCDC2	4615	2.15673	5.14216	-1.25353	5.00E-05	0.00170056
NM_130782.2	64407	RGS18	2145	27.0136	8.23576	1.71371	5.00E-05	0.00170056
NM_002631.2	5226	PGD	1937	78.0959	26.4082	1.56426	5.00E-05	0.00170056
NM_178329.2	1232	CCR3	1717	11.2616	0.586317	4.26358	5.00E-05	0.00170056
NM_001548.3	3434	IFIT1	1861	20.1525	50.5275	-1.32611	5.00E-05	0.00170056
NM_198469.2	254956	MORN5	705	13.9086	53.541	-1.94466	5.00E-05	0.00170056
NM_152723.1	220388	CCDC89	2428	2.79367	8.37432	-1.58381	5.00E-05	0.00170056
NM_205545.1	137797	LYPD2	534	29.9235	82.1161	-1.45639	5.00E-05	0.00170056
NM_014157.3	29070	CCDC113	5272	7.57459	29.9856	-1.98503	5.00E-05	0.00170056
NM_006988.3	9510	ADAMTS1	4651	107.144	11.7692	3.18646	5.00E-05	0.00170056
NM_000681.3	150	ADRA2A	3876	23.6584	8.47532	1.48102	5.00E-05	0.00170056
NM_000494.3	1308	COL17A1	5610	6.47481	1.50136	2.10857	5.00E-05	0.00170056
NM_054114.3	117289	TAGAP	3363	17.2271	7.31083	1.23657	5.00E-05	0.00170056
NM_003745.1	8651	SOCS1	1216	8.32931	2.14757	1.95549	5.00E-05	0.00170056
NM_002725.3	5549	PRELP	5820	0.70576	4.20122	-2.57356	5.00E-05	0.00170056
NM_207517.2	57188	ADAMTSL3	7336	1.00455	5.16367	-2.36184	5.00E-05	0.00170056
NM_001927.3	1674	DES	2248	0.408419	11.2504	-4.78378	5.00E-05	0.00170056
TCONS_00114410	XLOC_009730	-	4688	3.40649	32.4166	-3.25038	5.00E-05	0.00170056
TCONS_00233526	XLOC_020559	-	4976	2.17561	14.7785	-2.76401	5.00E-05	0.00170056
NM_005024.1	5273	SERPINB10	1194	14.3433	3.85368	1.89607	5.00E-05	0.00170056
NM_015914.5	51061	TXNDC11	3047	32.1322	13.9276	1.20607	5.00E-05	0.00170056
NM_173528.2	161502	C15orf26	1575	4.11994	10.3361	-1.32700	5.00E-05	0.00170056
NM_030955.2	81792	ADAMTS12	4955	7.86652	3.08893	1.34862	5.00E-05	0.00170056
NM_014442.2	27181	SIGLEC8	2949	6.0318	0.828849	2.86341	5.00E-05	0.00170056
NM_000625.4	4843	NOS2	4206	138.964	32.9436	2.07664	5.00E-05	0.00170056
NM_005613.5	5999	RGS4	3371	43.9162	3.37979	3.69975	5.00E-05	0.00170056
NM_173833.5	286133	SCARA5	4151	3.36922	11.485	-1.76926	5.00E-05	0.00170056
NM_004036.3	109	ADCY3	4397	41.1765	10.7229	1.94113	5.00E-05	0.00170056
NM_005420.2	6783	SULT1E1	1780	5.87511	20.0132	-1.76827	5.00E-05	0.00170056
NM_003617.3	8490	RGS5	5927	127.864	27.46	2.21921	5.00E-05	0.00170056
NM_152643.6	85442	KNDC1	6773	0.615115	2.22139	-1.85253	5.00E-05	0.00170056
NM_004310.3	399	RHOH	1999	13.7441	5.03523	1.44868	5.00E-05	0.00170056
NM_006186.3	4929	NR4A2	3531	36.2997	2.52602	3.84502	5.00E-05	0.00170056
NM_018100.3	114327	EFHC1	5582	13.809	31.5883	-1.19378	5.00E-05	0.00170056
NM_004877.2	9535	GMFG	657	54.6316	20.698	1.40025	5.00E-05	0.00170056
NM_002291.2	3912	LAMB1	5846	80.7467	35.4162	1.18899	5.00E-05	0.00170056
NM_005337.4	3071	NCKAP1L	3893	32.9666	15.6878	1.07136	5.00E-05	0.00170056
NM_015896.2	51364	ZMYND10	1773	12.5612	40.1134	-1.67510	5.00E-05	0.00170056
NM_002934.2	6036	RNASE2	735	13.7152	1.89386	2.85638	5.00E-05	0.00170056
NM_018602.3	55466	DNAJA4	3186	6.74825	27.6561	-2.03501	5.00E-05	0.00170056
NM_004283.3	9545	RAB3D	4273	17.0659	7.80532	1.12859	5.00E-05	0.00170056
XM_003118518.1	728763	LOC728763	4773	0.625649	2.42598	-1.95514	5.00E-05	0.00170056
NM_025208.4	80310	PDGFD	3993	17.927	50.6722	-1.49906	5.00E-05	0.00170056
NM_003245.3	7053	TGM3	2677	0.373215	2.08807	-2.48409	5.00E-05	0.00170056
NM_003551.2	8382	NME5	1226	15.7707	41.1749	-1.38452	5.00E-05	0.00170056
NM_004522.1	3800	KIF5C	6933	4.06583	0.995353	2.03027	5.00E-05	0.00170056
NM_005099.4	9507	ADAMTS4	4332	7.26144	0.769313	3.23861	5.00E-05	0.00170056
NM_052929.1	114827	FHAD1	5090	7.22349	21.7711	-1.59164	5.00E-05	0.00170056

NM_139164.1	134429	STARD4	2264	36.0735	9.85789	1.87159	5.00E-05	0.00170056
NM_002010.2	2254	FGF9	4530	0.735149	3.95586	-2.42788	5.00E-05	0.00170056
NM_003037.2	6504	SLAMF1	2001	8.36112	3.22793	1.37309	5.00E-05	0.00170056
NM_001099434.1	149069	DCDC2B	1349	3.97787	11.7438	-1.56183	5.00E-05	0.00170056
NM_032372.4	84332	DYDC2	1842	3.08081	15.5502	-2.33555	5.00E-05	0.00170056
TCONS_00283549	XLOC_023644	-	4674	1.00249	4.41261	-2.13805	5.00E-05	0.00170056
NM_021732.2	60370	AVPI1	1392	16.5954	5.65012	1.55443	5.00E-05	0.00170056
NM_005233.5	2042	EPHA3	5809	0.591364	6.61111	-3.48277	5.00E-05	0.00170056
TCONS_00002363	XLOC_001856	-	3621	0.222229	1.21471	-2.45050	5.00E-05	0.00170056
NM_002309.3	3976	LIF	3919	10.3753	1.67161	2.63384	5.00E-05	0.00170056
NM_003890.2	8857	FCGBP	16390	5.4615	0.968251	2.49584	5.00E-05	0.00170056
NM_001170700.1	401124	DTHD1	4336	11.1902	42.8185	-1.93600	5.00E-05	0.00170056
NM_000450.2	6401	SELE	3875	15.0178	1.0889	3.78573	5.00E-05	0.00170056
NM_000616.4	920	CD4	3116	19.5486	7.53192	1.37598	5.00E-05	0.00170056
NM_080826.1	140862	ISM1	2592	0.361083	3.53493	-3.29128	5.00E-05	0.00170056
NM_014675.3	9696	CROCC	6656	4.96803	11.6895	-1.23446	5.00E-05	0.00170056
NM_015529.2	26002	MOXD1	3039	5.96796	15.3568	-1.36357	5.00E-05	0.00170056
TCONS_00048685	XLOC_003947	-	3649	0.750647	3.90145	-2.37780	5.00E-05	0.00170056
NM_012472.3	23639	LRRC6	1582	7.53666	29.596	-1.97341	5.00E-05	0.00170056
NM_001901.2	1490	CTGF	2344	245.499	73.5072	1.73976	5.00E-05	0.00170056
NM_018837.3	55959	SULF2	3899	32.3896	16.244	0.99562	5.00E-05	0.00170056
NM_198505.2	344905	ATP13A5	3657	7.9423	0.202547	5.29323	5.00E-05	0.00170056
NM_000397.3	1536	CYBB	4318	72.5272	32.2032	1.17132	5.00E-05	0.00170056
NM_001008539.3	6542	SLC7A2	7571	20.0817	81.1648	-2.01497	5.00E-05	0.00170056
NM_001300.5	1316	KLF6	4662	51.0315	21.3798	1.25514	5.00E-05	0.00170056
NM_207310.1	91409	CCDC74B	1471	1.97281	7.4269	-1.91251	5.00E-05	0.00170056
TCONS_00196351	XLOC_017552	-	2737	0.241362	1.508	-2.64337	5.00E-05	0.00170056
NM_001010978.2	388633	LDLRAD1	785	4.76103	21.8932	-2.20114	5.00E-05	0.00170056
NM_002629.2	5223	PGAM1	1720	76.1938	37.1557	1.03609	5.00E-05	0.00170056
NM_001136023.1	4778	NFE2	1648	3.5887	0.790395	2.18282	5.00E-05	0.00170056
XM_003118626.2	100506209	LOC100506209	3519	1.21469	4.65572	-1.93842	5.00E-05	0.00170056
NM_199289.1	341676	NEK5	2912	5.07284	22.3474	-2.13924	5.00E-05	0.00170056
NM_002982.3	6347	CCL2	749	116.617	27.8862	2.06415	5.00E-05	0.00170056
NM_203418.1	1827	RCAN1	2408	52.0108	8.46773	2.61876	5.00E-05	0.00170056
NM_016132.3	50804	MYEF2	5403	3.15685	7.26697	-1.20287	5.00E-05	0.00170056
NM_080607.2	128434	VSTM2L	1966	2.37048	9.42846	-1.99184	5.00E-05	0.00170056
NM_004319.1	460	ASTN1	7314	1.72593	4.67112	-1.43639	5.00E-05	0.00170056
NM_182519.2	149954	C20orf186	2174	215.456	0.410857	9.03454	5.00E-05	0.00170056
NM_153355.3	154215	NKAIN2	3114	0.313373	1.86897	-2.57629	5.00E-05	0.00170056
NM_033049.3	56667	MUC13	2876	0.770846	4.95522	-2.68443	5.00E-05	0.00170056
NM_014518.2	7772	ZNF229	4964	0.80532	2.9793	-1.88734	5.00E-05	0.00170056
TCONS_00156537	XLOC_014786	-	2200	1.41952	10.4718	-2.88303	5.00E-05	0.00170056
NM_004419.3	1847	DUSP5	2528	51.4685	3.02362	4.08934	5.00E-05	0.00170056
NM_012198.3	25801	GCA	3226	17.687	6.82261	1.37430	5.00E-05	0.00170056
NM_000774.3	1572	CYP2F1	1826	2.28882	10.1934	-2.15496	5.00E-05	0.00170056
NM_001037763.2	340267	COL28A1	3515	2.24992	7.67993	-1.77122	5.00E-05	0.00170056
NM_001692.3	525	ATP6V1B1	1939	1.13725	6.22053	-2.45149	5.00E-05	0.00170056
NM_144687.2	91662	NLRP12	3871	2.62713	0.432728	2.60196	5.00E-05	0.00170056
NM_005467.3	10003	NAALAD2	3196	3.64783	9.39162	-1.36434	5.00E-05	0.00170056
NM_002855.4	5818	PVRL1	5481	6.69305	2.89439	1.20940	5.00E-05	0.00170056
NM_005746.2	10135	NAMPT	4582	109.804	33.1088	1.72964	5.00E-05	0.00170056
NM_001855.3	1306	COL15A1	5273	116.82	47.651	1.29371	5.00E-05	0.00170056
TCONS_00202813	XLOC_018025	-	11391	0.632903	2.21341	-1.80622	5.00E-05	0.00170056
NM_004573.2	5330	PLCB2	4677	17.4669	5.9517	1.55325	5.00E-05	0.00170056

TCONS_00130407	XLOC_011249	-	5924	3.79528	0.945837	2.00454	5.00E-05	0.00170056
NM_207303.2	26033	ATRNL1	8732	3.52654	8.98249	-1.34886	5.00E-05	0.00170056
NM_002658.3	5328	PLAU	2378	9.93527	2.54705	1.96373	5.00E-05	0.00170056
TCONS_00220788	XLOC_018529	-	12307	1.29822	9.25856	-2.83425	5.00E-05	0.00170056
NM_003463.3	7803	PTP4A1	5082	42.6519	18.4763	1.20693	5.00E-05	0.00170056
NM_020398.3	57119	SPINLW1	1989	2.12928	9.24972	-2.11904	5.00E-05	0.00170056
NM_001046.2	6558	SLC12A2	6860	58.2409	26.3532	1.14405	5.00E-05	0.00170056
NM_080876.3	142679	DUSP19	5379	3.34223	7.97699	-1.25503	5.00E-05	0.00170056
NM_006931.2	6515	SLC2A3	3938	64.4682	6.6203	3.28362	5.00E-05	0.00170056
NM_024164.5	64499	TPSB2	1166	15.8194	5.04708	1.64818	5.00E-05	0.00170056
NM_152377.2	127795	C1orf87	2028	5.88121	29.0239	-2.30306	5.00E-05	0.00170056
NM_183376.2	91947	ARRDC4	4067	27.9758	13.9718	1.00166	5.00E-05	0.00170056
NM_021572.4	59084	ENPP5	2943	18.8783	46.0303	-1.28585	5.00E-05	0.00170056
NM_001198.3	639	PRDM1	5165	32.3901	7.43687	2.12278	5.00E-05	0.00170056
NM_145027.4	221458	KIF6	3972	1.55591	6.42114	-2.04507	5.00E-05	0.00170056
NM_032291.2	84251	SGIP1	4694	6.60444	1.11917	2.56101	5.00E-05	0.00170056
NM_016240.2	51435	SCARA3	3631	8.19438	19.6595	-1.26252	5.00E-05	0.00170056
NM_153837.1	221188	GPR114	3761	3.09949	1.00844	1.61991	5.00E-05	0.00170056
NM_001370.1	1768	DNAH6	12795	8.36713	26.4719	-1.66166	5.00E-05	0.00170056
NM_001142699.1	1740	DLG2	8046	0.732339	2.50481	-1.77412	0.0001	0.00312189
NM_032855.2	84941	HSH2D	2367	9.8204	4.01723	1.28958	0.0001	0.00312189
NM_198492.3	339390	CLEC4G	1360	7.34315	0.188927	5.28050	0.0001	0.00312189
NM_138983.2	116448	OLIG1	2279	1.56839	0.277315	2.49969	0.0001	0.00312189
NM_014600.2	30845	EHD3	3906	9.27172	4.4657	1.05395	0.0001	0.00312189
NM_022454.3	64321	SOX17	2350	6.34451	1.98995	1.67277	0.0001	0.00312189
NM_001128178.1	4867	NPHP1	2583	6.04621	18.0636	-1.57898	0.0001	0.00312189
NM_021982.1	10802	SEC24A	6387	24.0523	12.7041	0.92088	0.0001	0.00312189
NM_015892.3	51363	CHST15	4772	33.6386	16.3352	1.04213	0.0001	0.00312189
NM_207365.3	344752	AADACL2	1500	2.46449	7.16366	-1.53941	0.0001	0.00312189
NM_020726.4	57486	NLN	8661	5.07155	2.57144	0.97985	0.0001	0.00312189
NM_005308.2	2869	GRK5	2558	20.7655	9.83868	1.07765	0.0001	0.00312189
NM_012450.2	26266	SLC13A4	2896	0.323442	1.41974	-2.13405	0.0001	0.00312189
NM_001747.2	822	CAPG	1442	36.599	17.5555	1.05988	0.0001	0.00312189
NM_001010860.1	161394	SAMD15	2025	5.89604	15.4027	-1.38536	0.0001	0.00312189
NM_003290.2	7171	TPM4	2645	185.917	74.5558	1.31827	0.0001	0.00312189
NM_002466.2	4605	MYBL2	2713	1.71879	0.356106	2.27101	0.0001	0.00312189
NM_152891.2	260429	PRSS33	1694	3.18508	0.199729	3.99521	0.0001	0.00312189
NM_016184.3	50856	CLEC4A	1281	11.5149	3.838	1.58508	0.0001	0.00312189
NM_014452.3	27242	TNFRSF21	3634	23.9361	12.6753	0.91717	0.0001	0.00312189
NM_003775.3	8698	S1PR4	1595	5.05165	1.56026	1.69497	0.0001	0.00312189
NM_052913.2	114801	TMEM200A	5023	12.771	6.14934	1.05437	0.0001	0.00312189
NM_152403.3	133584	EGFLAM	4870	3.71132	0.305354	3.60338	0.0001	0.00312189
NM_005178.4	602	BCL3	1864	3.99111	1.01686	1.97267	0.0001	0.00312189
NM_021202.1	58476	TP53INP2	4124	8.59809	4.14375	1.05308	0.0001	0.00312189
NM_006762.2	7805	LAPTM5	2240	98.3309	45.4328	1.11391	0.0001	0.00312189
TCONS_00256553	XLOC_022163	-	3585	0.809599	2.5145	-1.63500	0.0001	0.00312189
NM_144668.4	144406	WDR66	3755	8.86727	25.3145	-1.51340	0.0001	0.00312189
NM_022159.3	64123	ELTD1	3619	41.0828	20.0317	1.03625	0.0001	0.00312189
NM_001130058.1	204962	SLC44A5	3894	0.0925231	1.35922	-3.87682	0.0001	0.00312189
NM_001101.3	60	ACTB	1812	546.491	247.482	1.14288	0.0001	0.00312189
NM_000235.2	3988	LIPA	2624	114.142	56.9854	1.00217	0.0001	0.00312189
NM_006207.2	5157	PDGFRL	1905	5.0509	12.6782	-1.32774	0.0001	0.00312189
NM_001987.4	2120	ETV6	5989	28.8239	14.5064	0.99058	0.0001	0.00312189
NM_001145033.1	387763	C11orf96	1328	9.44974	2.90322	1.70262	0.0001	0.00312189

NM_005188.2	867	CBL	11227	12.0489	5.94293	1.01965	0.0001	0.00312189
NM_005080.3	7494	XBP1	1820	443.071	168.768	1.39249	0.0001	0.00312189
NM_004267.3	9435	CHST2	3043	5.30288	1.83738	1.52913	0.0001	0.00312189
NM_005763.3	10157	AASS	5871	21.2372	40.6068	-0.93513	0.0001	0.00312189
NM_004490.2	2888	GRB14	2387	2.43755	0.594225	2.03635	0.0001	0.00312189
NM_006217.3	5276	SERPINI2	1430	6.15067	15.8819	-1.36857	0.0001	0.00312189
NM_006820.2	10964	IFI44L	5874	13.0691	26.0828	-0.99694	0.0001	0.00312189
NM_017899.3	54997	TESC	1019	6.08521	1.55267	1.97056	0.0001	0.00312189
NM_012128.3	22802	CLCA4	3213	3.14861	0.856218	1.87867	0.0001	0.00312189
NM_021965.3	5239	PGM5	3338	5.3593	11.5312	-1.10542	0.0001	0.00312189
NM_004666.2	8876	VNN1	3844	1.11416	3.34588	-1.58642	0.0001	0.00312189
NM_001136536.3	401124	DTHD1	4174	6.53736	23.4793	-1.84461	0.0001	0.00312189
NM_152222.1	84957	RELT	3441	4.11863	1.36706	1.59109	0.0001	0.00312189
NM_012335.3	4542	MYO1F	4173	11.8334	5.46903	1.11351	0.0001	0.00312189
NM_014214.2	3613	IMPA2	1537	12.6433	5.21072	1.27881	0.0001	0.00312189
NM_144696.4	126859	AXDND1	3642	0.373224	1.53681	-2.04183	0.0001	0.00312189
NM_003637.3	8515	ITGA10	5170	1.76991	4.08453	-1.20649	0.0001	0.00312189
NM_032960.2	9261	MAPKAPK2	3052	27.0919	13.431	1.01230	0.0001	0.00312189
NM_144607.4	124637	CYB5D1	3746	10.7276	22.1838	-1.04817	0.0001	0.00312189
NM_001171.5	368	ABCC6	5117	0.391075	1.5347	-1.97244	0.0001	0.00312189
NM_173050.3	80274	SCUBE1	3868	0.409544	1.63163	-1.99422	0.0001	0.00312189
NM_025057.2	80127	C14orf45	3106	10.2278	27.3281	-1.41789	0.0001	0.00312189
NM_019609.4	56265	CPXM1	2400	30.893	62.6989	-1.02116	0.0001	0.00312189
TCONS_00294386	XLOC_025176	-	2795	0.581082	2.97643	-2.35677	0.0001	0.00312189
NM_023037.2	10129	FRY	10717	16.9511	9.04581	0.90605	0.0001	0.00312189
TCONS_00234687	XLOC_020386	-	870	0.984942	5.5224	-2.48719	0.0001	0.00312189
NM_019594.3	56262	LRRC8A	4355	11.6663	5.56225	1.06861	0.0001	0.00312189
NM_000859.2	3156	HMGCR	4582	27.5603	13.8039	0.99752	0.0001	0.00312189
NM_003798.2	8727	CTNNAL1	2445	72.7985	37.1197	0.97173	0.0001	0.00312189
NM_007035.3	11081	KERA	2533	0.236443	1.56337	-2.72510	0.0001	0.00312189
NM_005472.4	10008	KCNE3	3070	32.6935	15.9687	1.03376	0.0001	0.00312189
NM_005717.2	10092	ARPC5	1982	103.652	51.6959	1.00362	0.0001	0.00312189
NM_080596.1	85235	HIST1H2AH	439	40.0337	13.0957	1.61213	0.0001	0.00312189
NM_001004306.1	339184	CCDC144NL	2806	0.156135	1.40141	-3.16602	0.0001	0.00312189
NM_001099692.1	143244	EIF5AL1	3840	0.132443	0.792116	-2.58034	0.0001	0.00312189
NM_173054.2	5649	RELN	11565	0.71523	2.0522	-1.52069	0.0001	0.00312189
NM_001145710.1	375190	LOC375190	1357	17.9748	35.5513	-0.98392	0.0001	0.00312189
TCONS_00049984	XLOC_004304	-	3774	2.87027	0.991739	1.53315	0.0001	0.00312189
NM_001004354.2	441478	NRARP	2621	7.36206	2.88738	1.35035	0.0001	0.00312189
NM_001145029.1	374860	ANKRD30B	4617	1.54551	0.436948	1.82255	0.0001	0.00312189
NM_004093.3	1948	EFNB2	4387	11.0504	5.4565	1.01805	0.00015	0.00437065
NM_003930.3	8935	SKAP2	3971	13.6123	6.95233	0.96935	0.00015	0.00437065
NM_001083537.1	85002	FAM86B1	2438	1.01585	3.30979	-1.70405	0.00015	0.00437065
NM_014791.2	9833	MELK	2439	3.48268	0.952747	1.87003	0.00015	0.00437065
NM_033423.3	2999	GZMH	902	3.91429	11.1628	-1.51188	0.00015	0.00437065
NM_001098519.1	254050	LRRC43	2025	1.89852	5.70931	-1.58844	0.00015	0.00437065
NM_016185.2	51155	HN1	1602	21.6678	9.76826	1.14938	0.00015	0.00437065
NM_001040147.1	8710	SERPINB7	1973	1.82443	7.3731	-2.01483	0.00015	0.00437065
NM_002009.3	2252	FGF7	3936	71.2442	32.1	1.15020	0.00015	0.00437065
NM_024886.1	79946	C10orf95	1469	2.28051	7.86922	-1.78686	0.00015	0.00437065
NM_178815.3	221079	ARL5B	3589	29.5572	15.0757	0.97129	0.00015	0.00437065
NM_012413.3	25797	QPCT	1703	10.6364	4.26102	1.31974	0.00015	0.00437065
NM_020844.2	57604	KIAA1456	9590	2.82762	8.11618	-1.52121	0.00015	0.00437065
NM_144648.1	136332	LRGUK	2700	4.61097	10.4557	-1.18115	0.00015	0.00437065

ENST00000528986.1	ENSG00000255248.2	RP11-166D19.1	3287	22.0809	41.2004	-0.89986	0.00015	0.00437065
NM_018430.2	55815	TSNAXIP1	2446	2.50327	6.71949	-1.42454	0.00015	0.00437065
NM_152550.3	153769	SH3RF2	3043	5.96655	2.31054	1.36867	0.00015	0.00437065
NM_001946.2	1848	DUSP6	2820	42.7067	20.9117	1.03015	0.00015	0.00437065
NM_001634.4	262	AMD1	3421	55.9037	29.3008	0.93200	0.00015	0.00437065
TCONS_00196366	XLOC_017565	-	4542	0.676998	2.00249	-1.56457	0.00015	0.00437065
NM_015653.3	26150	RIBC2	1396	3.66533	9.78658	-1.41686	0.00015	0.00437065
NM_007364.2	23423	TMED3	1388	39.2003	19.8637	0.98073	0.00015	0.00437065
NM_001908.3	1508	CTSB	3783	135.595	61.0981	1.15011	0.00015	0.00437065
NM_001165.3	330	BIRC3	5229	77.5134	27.1518	1.51340	0.00015	0.00437065
NM_013340.2	29930	PCDHB1	2534	0.551842	2.04343	-1.88866	0.00015	0.00437065
NM_032785.3	84871	AGBL4	2988	1.18264	4.94793	-2.06481	0.00015	0.00437065
NM_020801.2	57561	ARRDC3	4161	154.907	54.3784	1.51030	0.00015	0.00437065
NM_013249.2	7761	ZNF214	2668	6.94882	14.8654	-1.09712	0.00015	0.00437065
NM_015065.2	23086	EXPH5	10187	20.5618	40.9518	-0.99396	0.00015	0.00437065
NM_002704.3	5473	PPBP	1307	1.0146	5.13818	-2.34035	0.00015	0.00437065
XM_003403516.1	285754	FLJ37396	1633	0.628533	2.73586	-2.12193	0.00015	0.00437065
NM_013336.3	29927	SEC61A1	3636	88.7533	47.2719	0.90882	0.00015	0.00437065
3493	3493	-	1061	1474.25	410.424	1.84480	0.00015	0.00437065
NM_000175.3	2821	GPI	4194	31.833	17.2698	0.88227	0.00015	0.00437065
NM_001136482.1	255809	C19orf38	1089	4.53113	0.9874	2.19817	0.00015	0.00437065
NM_001134363.1	282996	RBM20	7233	1.92979	4.45379	-1.20659	0.00015	0.00437065
NM_138787.2	119710	C11orf74	820	20.5905	46.46	-1.17401	0.00015	0.00437065
NM_014399.3	27075	TSPAN13	1902	71.3301	37.5247	0.92667	0.00015	0.00437065
NM_153209.3	124602	KIF19	3643	2.52015	5.66254	-1.16794	0.00015	0.00437065
NM_032793.3	84879	MFSD2A	2162	6.96654	2.42036	1.52522	0.00015	0.00437065
NM_024893.1	79953	SYNDIG1	2437	1.91458	0.447981	2.09552	0.00015	0.00437065
NM_176813.3	155465	AGR3	750	23.579	56.8907	-1.27069	0.00015	0.00437065
NM_004428.2	1942	EFNA1	1566	17.6753	7.35142	1.26564	0.00015	0.00437065
NM_001452.1	2295	FOXF2	2187	2.91885	0.910452	1.68075	0.00015	0.00437065
NM_006948.4	6782	HSPA13	3987	35.16	19.5935	0.84356	0.00015	0.00437065
NM_178527.3	284525	SLC9A11	4427	0.874666	3.02734	-1.79125	0.00015	0.00437065
NM_022122.2	64066	MMP27	1647	2.18426	0.376845	2.53510	0.00015	0.00437065
NM_199242.2	201294	UNC13D	4393	5.54494	2.20844	1.32815	0.00015	0.00437065
NM_032965.4	6359	CCL15	1080	1.17134	5.01141	-2.09706	0.00015	0.00437065
NM_014568.1	11227	GALNT5	3131	22.0673	11.1739	0.98177	0.00015	0.00437065
TCONS_00295272	XLOC_025154	-	2524	0.300143	1.61553	-2.42828	0.00015	0.00437065
NM_001031737.2	124093	CCDC78	1611	6.69397	19.1913	-1.51952	0.00015	0.00437065
ENST00000544591.1	ENSG00000256039.1	RP11-291B21.2	868	0.79541	8.20819	-3.36729	0.00015	0.00437065
ENST00000448680.1	ENSG00000233730.1	RP4-666F24.3	491	4.49577	19.8548	-2.14285	0.00015	0.00437065
TCONS_00294490	XLOC_025290	-	672	1.20612	8.15802	-2.75784	0.00015	0.00437065
NM_000849.4	2947	GSTM3	4127	8.9259	17.5574	-0.97601	0.00015	0.00437065
NM_003919.2	8910	SGCE	1700	19.4801	37.4585	-0.94330	0.00015	0.00437065
NM_002515.2	4857	NOVA1	3918	1.5878	4.41218	-1.47446	0.00015	0.00437065
NM_014325.2	23603	CORO1C	3815	44.1993	23.725	0.89762	0.00015	0.00437065
NM_002334.3	4038	LRP4	8227	2.85108	6.31228	-1.14665	0.00015	0.00437065
NM_012248.2	22928	SEPHS2	2273	15.0175	7.19117	1.06234	0.00015	0.00437065
TCONS_00272364	XLOC_022448	-	770	22.3127	7.01655	1.66903	0.00015	0.00437065
NM_004635.3	7867	MAPKAPK3	2500	17.7582	8.69068	1.03094	0.00015	0.00437065
NM_000270.3	4860	PNP	2421	12.3639	5.71547	1.11319	0.00015	0.00437065
TCONS_00187673	XLOC_016076	-	900	0.709876	4.93536	-2.79752	0.00015	0.00437065
NM_153449.2	144195	SLC2A14	2195	2.0701	0.313593	2.72274	0.00015	0.00437065
NM_080617.4	140689	CBLN4	2508	0.125269	4.46969	-5.15707	0.00015	0.00437065
NM_019856.1	8736	MYOM1	5559	0.915933	3.51762	-1.94129	0.00015	0.00437065

NM_198507.1	345757	FAM174A	1236	21.8669	45.0498	-1.04277	0.00015	0.00437065
NM_003039.2	6518	SLC2A5	2438	2.47123	0.667059	1.88935	0.0002	0.00554203
NM_173814.4	283659	PRTG	11973	2.97784	5.65892	-0.92626	0.0002	0.00554203
NM_032865.5	84951	TNS4	4072	6.5225	2.72352	1.25995	0.0002	0.00554203
NM_198474.3	283298	OLFML1	2905	7.09282	13.9819	-0.97913	0.0002	0.00554203
NM_024837.2	79895	ATP8B4	5677	13.918	7.003	0.99090	0.0002	0.00554203
NM_001731.2	694	BTG1	4680	75.7144	36.3124	1.06010	0.0002	0.00554203
NM_005194.2	1051	CEBPB	1837	26.5616	13.4922	0.97722	0.0002	0.00554203
NM_001007544.1	440712	C1orf186	1129	13.5028	5.01827	1.42800	0.0002	0.00554203
NM_003531.2	8352	HIST1H3C	459	36.2829	11.4829	1.65980	0.0002	0.00554203
NM_001813.2	1062	CENPE	8612	3.42286	1.44054	1.24859	0.0002	0.00554203
NM_014822.2	9871	SEC24D	4063	27.8309	15.1497	0.87739	0.0002	0.00554203
NM_001425.2	2014	EMP3	829	35.1956	15.126	1.21837	0.0002	0.00554203
NM_012391.1	25803	SPDEF	1894	7.57825	2.79498	1.43903	0.0002	0.00554203
NM_002530.2	4916	NTRK3	2818	1.93064	8.48226	-2.13537	0.0002	0.00554203
NM_018371.4	55790	CSGALNACT1	3872	12.7338	5.25233	1.27763	0.0002	0.00554203
NM_003529.2	8350	HIST1H3A	469	30.6004	10.3643	1.56194	0.0002	0.00554203
NM_032040.3	83987	CCDC8	3334	2.47606	6.2283	-1.33079	0.0002	0.00554203
NM_003874.3	8832	CD84	8228	18.6677	9.73998	0.93856	0.0002	0.00554203
NM_015055.2	23075	SWAP70	4848	42.8351	23.316	0.87748	0.0002	0.00554203
NM_152505.3	150082	LCA5L	2364	3.26647	8.35169	-1.35433	0.0002	0.00554203
NM_015272.2	23322	RPGRIP1L	6182	3.09297	8.38534	-1.43888	0.0002	0.00554203
NM_213607.1	388389	CCDC103	1256	4.29614	11.6767	-1.44252	0.0002	0.00554203
NM_001079872.1	8450	CUL4B	5184	68.2226	33.8128	1.01268	0.0002	0.00554203
NM_001145451.2	100271715	ARHGEF33	4304	0.930281	2.60222	-1.48401	0.0002	0.00554203
TCONS_00155291	XLOC_013582	-	1719	0.308636	2.36377	-2.93711	0.0002	0.00554203
NM_182539.3	202500	TCTE1	1683	1.41797	4.64689	-1.71244	0.0002	0.00554203
NM_014330.3	23645	PPP1R15A	2383	47.0427	24.4647	0.94327	0.0002	0.00554203
NM_014876.5	9929	JOSD1	3435	27.1316	14.6161	0.89242	0.0002	0.00554203
NM_006265.2	5885	RAD21	3750	115.353	60.121	0.94012	0.0002	0.00554203
NM_001204376.1	4883	NPR3	6380	4.74809	1.65885	1.51716	0.0002	0.00554203
NM_006079.3	10370	CITED2	1930	58.2174	28.8156	1.01460	0.0002	0.00554203
NM_000165.3	2697	GJA1	3130	139.442	58.3974	1.25569	0.0002	0.00554203
NM_031912.4	83849	SYT15	5508	6.15942	11.8717	-0.94665	0.0002	0.00554203
NM_138803.3	130940	CCDC148	2846	2.53612	6.60708	-1.38139	0.0002	0.00554203
NM_005065.4	6400	SEL1L	6579	81.8322	40.679	1.00838	0.0002	0.00554203
NM_005906.4	4117	MAK	3883	2.38789	7.00752	-1.55316	0.0002	0.00554203
NM_031294.3	83450	LRRC48	2080	9.02474	20.9808	-1.21711	0.0002	0.00554203
NM_012081.5	22936	ELL2	6046	47.9627	24.7787	0.95281	0.0002	0.00554203
TCONS_00314598	XLOC_026580	-	3111	1.9928	0.161749	3.62297	0.0002	0.00554203
NM_001008.3	6192	RPS4Y1	897	235.471	86.9187	1.43781	0.0002	0.00554203
NM_020530.4	5008	OSM	1854	11.2806	0.132587	6.41076	0.0002	0.00554203
NM_004962.3	2662	GDF10	2650	0.340603	1.84113	-2.43443	0.0002	0.00554203
TCONS_00079878	XLOC_006712	-	3633	0.374562	2.39433	-2.67635	0.0002	0.00554203
NM_031419.3	64332	NFKBIZ	3926	26.6871	3.11054	3.10091	0.0002	0.00554203
NM_005063.4	6319	SCD	5468	10.1292	5.18864	0.96509	0.0002	0.00554203
NM_004710.3	9144	SYNGR2	1694	27.0738	13.3931	1.01541	0.0002	0.00554203
NM_181265.3	116966	WDR17	7353	0.774493	2.15587	-1.47695	0.0002	0.00554203
NM_001005852.2	246126	CYorf15A	865	30.3339	7.37494	2.04023	0.0002	0.00554203
NM_139021.2	225689	MAPK15	1888	2.39778	6.77467	-1.49845	0.0002	0.00554203
NM_018098.4	1894	ECT2	3910	11.1194	5.43422	1.03293	0.0002	0.00554203
NM_018290.3	55276	PGM2	3238	22.5432	11.8047	0.93333	0.0002	0.00554203
NM_176824.2	55212	BBS7	3863	4.0925	9.52179	-1.21825	0.00025	0.00665625
NM_173662.2	285533	RNF175	1569	0.968539	3.54207	-1.87071	0.00025	0.00665625

NM_014708.4	9735	KNTC1	6965	13.1954	7.32504	0.84913	0.00025	0.00665625
NM_152357.2	126070	ZNF440	4181	18.9243	34.1796	-0.85289	0.00025	0.00665625
NM_017982.3	55061	SUSD4	3027	2.39564	6.00956	-1.32685	0.00025	0.00665625
NM_000847.4	2940	GSTA3	915	0.650144	4.12801	-2.66661	0.00025	0.00665625
NM_001145204.2	729993	SHISA9	6727	28.5477	61.3641	-1.10402	0.00025	0.00665625
NM_001143779.1	28981	IFT81	2730	18.6581	36.3828	-0.96346	0.00025	0.00665625
NM_139284.2	163175	LGI4	2888	1.02399	2.90776	-1.50571	0.00025	0.00665625
NM_016639.2	51330	TNFRSF12A	1033	16.4938	5.44979	1.59765	0.00025	0.00665625
NM_007365.2	11240	PADI2	4363	4.091	1.65124	1.30890	0.00025	0.00665625
NM_024817.2	79875	THSD4	9145	24.1337	46.0633	-0.93257	0.00025	0.00665625
TCONS_00219349	XLOC_019061	-	2754	1.0001	3.12248	-1.64255	0.00025	0.00665625
NM_174899.4	130888	FBXO36	2815	5.9596	12.6261	-1.08312	0.00025	0.00665625
NM_152864.3	128414	NKAIN4	1430	0.489232	2.45336	-2.32617	0.00025	0.00665625
TCONS_00087575	XLOC_007492	-	3315	0.0804485	1.27903	-3.99084	0.00025	0.00665625
NM_000600.3	3569	IL6	1184	13.8138	0.225503	5.93682	0.00025	0.00665625
NM_000641.2	3589	IL11	2354	1.87655	0.433672	2.11341	0.00025	0.00665625
NM_002087.2	2896	GRN	2305	47.392	25.241	0.90887	0.00025	0.00665625
NM_018012.3	55083	KIF26B	7287	3.0548	1.36061	1.16682	0.00025	0.00665625
NM_015492.4	56905	C15orf39	4427	5.89063	2.73349	1.10768	0.00025	0.00665625
NM_001289.4	1193	CLIC2	2686	17.9547	9.31415	0.94687	0.00025	0.00665625
NM_002826.4	5768	QSOX1	3316	17.7402	8.1741	1.11789	0.00025	0.00665625
NM_145740.3	2938	GSTA1	1259	34.6795	72.8064	-1.06998	0.00025	0.00665625
TCONS_00000276	XLOC_000058	-	1132	0.706646	5.01879	-2.82828	0.00025	0.00665625
NM_024667.2	79720	VPS37B	2749	11.6403	5.36962	1.11623	0.00025	0.00665625
NM_006685.3	10879	SMR3B	785	5.9163	0.904048	2.71022	0.00025	0.00665625
NM_014890.2	11259	FILIP1L	3274	42.7503	20.3451	1.07125	0.00025	0.00665625
NM_153274.2	266675	BEST4	2096	0.959201	3.22982	-1.75155	0.00025	0.00665625
NM_004458.2	2182	ACSL4	5023	55.5212	24.4454	1.18348	0.00025	0.00665625
NM_201286.3	158880	USP51	4403	3.00494	6.41735	-1.09464	0.00025	0.00665625
NM_020848.2	57608	KIAA1462	9300	11.7107	21.0118	-0.84338	0.00025	0.00665625
NM_004313.3	409	ARRB2	1919	19.7064	9.01804	1.12778	0.00025	0.00665625
NM_021977.2	6581	SLC22A3	5624	0.754229	2.0226	-1.42314	0.00025	0.00665625
NM_024734.3	79789	CLMN	12750	10.5408	19.9674	-0.92166	0.00025	0.00665625
NM_032744.3	84830	C6orf105	1804	5.39781	1.50068	1.84676	0.00025	0.00665625
NM_032977.3	843	CASP10	5906	8.93825	4.33585	1.04368	0.00025	0.00665625
TCONS_00281525	XLOC_024216	-	3339	0.829399	2.89085	-1.80136	0.00025	0.00665625
NM_001097589.1	6707	SPRR3	902	5.92007	0.327627	4.17549	0.00025	0.00665625
NM_078483.2	206358	SLC36A1	5773	5.94467	3.06848	0.95407	0.00025	0.00665625
NM_016235.1	51704	GPRC5B	2845	27.1547	50.93	-0.90731	0.00025	0.00665625
TCONS_00023764	XLOC_000192	-	17064	4.18686	10.3915	-1.31146	0.00025	0.00665625
TCONS_00180095	XLOC_015624	-	1504	0.454408	2.43413	-2.42134	0.00025	0.00665625
TCONS_00173159	XLOC_014288	-	1849	0.482068	2.46775	-2.35589	0.00025	0.00665625
NM_005508.4	1233	CCR4	1657	8.70532	3.11228	1.48393	0.00025	0.00665625
NM_001160130.1	56479	KCNQ5	6556	0.71944	1.97105	-1.45402	0.0003	0.00772176
NM_012431.2	9723	SEMA3E	6629	0.205248	1.91219	-3.21979	0.0003	0.00772176
NM_033130.4	89790	SIGLEC10	3394	3.64689	0.928569	1.97358	0.0003	0.00772176
NM_016623.3	51571	FAM49B	2196	41.3185	21.7082	0.92854	0.0003	0.00772176
NM_000059.3	675	BRCA2	11386	5.26711	2.76446	0.93001	0.0003	0.00772176
NM_001061.4	6916	TBXAS1	2066	12.0886	4.17404	1.53413	0.0003	0.00772176
NM_003006.4	6404	SELPLG	2573	15.6738	7.42515	1.07787	0.0003	0.00772176
NM_138770.1	90557	CCDC74A	1457	4.02284	10.5368	-1.38915	0.0003	0.00772176
NM_004468.3	2275	FHL3	1704	5.23442	1.93055	1.43902	0.0003	0.00772176
NM_003878.2	8836	GGH	1493	14.276	6.233	1.19560	0.0003	0.00772176
XM_003403521.1	100652860	LOC100652860	1520	2.63232	7.85582	-1.57743	0.0003	0.00772176

NM_001017420.2	157570	ESCO2	3359	1.75667	0.500615	1.81107	0.0003	0.00772176
NM_001114.3	113	ADCY7	6138	12.806	6.73072	0.92798	0.0003	0.00772176
NM_152754.2	223117	SEMA3D	6261	6.78436	12.4977	-0.88138	0.0003	0.00772176
NM_000478.4	249	ALPL	2596	4.1073	9.76129	-1.24888	0.0003	0.00772176
NM_001093772.1	3815	KIT	5164	26.3922	13.3	0.98869	0.0003	0.00772176
TCONS_00112232	XLOC_009588	-	3307	3.65708	9.16718	-1.32579	0.0003	0.00772176
NM_006367.3	10487	CAP1	2773	98.7364	44.2391	1.15826	0.0003	0.00772176
NM_003840.3	8793	TNFRSF10D	3535	8.11859	3.70223	1.13283	0.0003	0.00772176
NM_025201.4	80301	PLEKHO2	3721	16.6043	8.5641	0.95518	0.0003	0.00772176
NM_007191.4	11197	WIF1	2240	0.104994	3.76249	-5.16331	0.0003	0.00772176
NM_015900.3	51365	PLA1A	1780	10.9356	3.08154	1.82731	0.0003	0.00772176
NM_022355.2	64174	DPEP2	1709	3.63164	0.942105	1.94666	0.0003	0.00772176
NM_023009.5	65108	MARCKSL1	1546	22.2247	11.0331	1.01033	0.0003	0.00772176
NM_005542.4	3638	INSIG1	3010	40.8928	19.2653	1.08584	0.0003	0.00772176
NM_006083.3	3550	IK	1935	72.0982	134.404	-0.89855	0.0003	0.00772176
NM_005239.4	2114	ETS2	3672	80.8129	44.3188	0.86667	0.0003	0.00772176
TCONS_00245230	XLOC_019931	-	1702	0.910424	3.48025	-1.93458	0.0003	0.00772176
NM_021135.4	6196	RPS6KA2	5817	12.6044	6.54418	0.94564	0.0003	0.00772176
NM_006061.2	10321	CRISP3	2219	0.234158	2.28608	-3.28732	0.0003	0.00772176
NM_001199149.1	4057	LTF	2508	243.987	102.844	1.24635	0.0003	0.00772176
NM_001175.4	397	ARHGDIB	1216	150.498	79.4677	0.92130	0.0003	0.00772176
NM_001122769.2	167691	LCA5	4564	11.3306	22.1913	-0.96976	0.0003	0.00772176
NM_015448.1	25911	DPCD	799	11.9766	28.6734	-1.25949	0.0003	0.00772176
NM_139072.3	92737	DNER	3272	0.930212	2.86434	-1.62257	0.0003	0.00772176
NM_001497.3	2683	B4GALT1	4199	35.1162	18.777	0.90317	0.0003	0.00772176
NM_144965.1	158248	TTC16	2884	1.50091	5.56205	-1.88977	0.0003	0.00772176
NM_001063.3	7018	TF	2791	1.42997	4.31891	-1.59468	0.00035	0.00869612
NM_152793.2	222166	C7orf41	5814	16.2493	29.7241	-0.87125	0.00035	0.00869612
NM_001192.2	608	TNFRSF17	994	18.0928	7.18924	1.33151	0.00035	0.00869612
NM_003564.1	8407	TAGLN2	1360	136.581	73.5734	0.89250	0.00035	0.00869612
NM_001206602.1	1123	CHN1	2294	18.4304	6.87212	1.42326	0.00035	0.00869612
NM_001025195.1	1066	CES1	2013	22.3099	56.7756	-1.34758	0.00035	0.00869612
NM_001130918.1	284076	TTLL6	3454	0.372245	1.61275	-2.11520	0.00035	0.00869612
NM_015364.4	23643	LY96	622	31.0977	11.2909	1.46165	0.00035	0.00869612
NM_006887.4	678	ZFP36L2	3693	65.4823	34.4541	0.92643	0.00035	0.00869612
NM_017510.4	54732	TMED9	1402	41.9046	22.3241	0.90850	0.00035	0.00869612
NM_004778.2	11251	GPR44	2910	6.43424	0.421858	3.93094	0.00035	0.00869612
NM_001128227.2	10020	GNE	5298	18.2042	7.74155	1.23358	0.00035	0.00869612
NM_012479.3	7532	YWHAG	3747	35.5102	20.0541	0.82434	0.00035	0.00869612
NM_001823.3	1152	CKB	1431	10.0316	21.7765	-1.11822	0.00035	0.00869612
NM_144629.2	130132	RFTN2	3113	6.25175	12.9838	-1.05438	0.00035	0.00869612
NM_001114357.1	441054	C4orf47	974	3.82704	20.4128	-2.41517	0.00035	0.00869612
NM_145739.2	114880	OSBPL6	6811	1.05267	7.64767	-2.86096	0.00035	0.00869612
NM_002483.4	4680	CEACAM6	2631	17.4981	32.4239	-0.88986	0.00035	0.00869612
NM_004675.2	9077	DIRAS3	1631	7.05424	2.58486	1.44840	0.00035	0.00869612
3497	3497	-	1286	12.7504	0.203681	5.96810	0.00035	0.00869612
NM_198565.1	375387	LRRC33	2465	4.4759	1.56693	1.51424	0.00035	0.00869612
NM_004219.2	9232	PTTG1	712	19.1929	6.80822	1.49522	0.00035	0.00869612
NM_005100.3	9590	AKAP12	8434	19.1537	47.4702	-1.30940	0.00035	0.00869612
NM_033063.1	4135	MAP6	2648	6.27087	14.6765	-1.22677	0.00035	0.00869612
NM_032782.3	84868	HAVCR2	2320	13.6584	6.50569	1.07001	0.00035	0.00869612
NM_015041.1	23059	CLUAP1	1661	20.9951	45.4983	-1.11576	0.00035	0.00869612
NM_014331.3	23657	SLC7A11	9648	1.93159	0.862837	1.16263	0.00035	0.00869612
NM_017424.2	51816	CECR1	3927	23.899	9.39168	1.34749	0.00035	0.00869612

NM_001145808.1	3684	ITGAM	4745	6.56814	1.10866	2.56667	0.00035	0.00869612
NM_013345.2	29933	GPR132	3636	3.60995	1.37817	1.38922	0.00035	0.00869612
NM_020675.3	57405	SPC25	1346	15.2114	31.0655	-1.03016	0.00035	0.00869612
NM_003509.2	8329	HIST1H2AI	469	19.511	4.52326	2.10885	0.00035	0.00869612
TCONS_00305796	XLOC_026031	-	9171	0.427449	1.65002	-1.94866	0.00035	0.00869612
NM_003383.3	7436	VLDLR	3636	3.39324	13.4397	-1.98577	0.00035	0.00869612
TCONS_00294953	XLOC_024750	-	6915	1.55244	3.33333	-1.10243	0.00035	0.00869612
TCONS_00106634	XLOC_008067	-	5143	1.39528	5.32095	-1.93112	0.00035	0.00869612
NM_004566.3	5209	PFKFB3	4550	12.7629	3.01094	2.08367	0.00035	0.00869612
NM_007356.2	22798	LAMB4	5858	1.02813	0.274688	1.90415	0.00035	0.00869612
NM_006500.2	4162	MCAM	3332	37.3498	20.2808	0.88099	0.00035	0.00869612
NM_006009.2	7846	TUBA1A	1666	131.023	245.314	-0.90480	0.00035	0.00869612
NM_173855.4	283385	MORN3	1158	2.43195	7.54159	-1.63275	0.0004	0.00971957
NM_001995.2	2180	ACSL1	3805	28.196	15.5685	0.85687	0.0004	0.00971957
NM_018725.3	55540	IL17RB	2040	9.24144	3.93406	1.23210	0.0004	0.00971957
NM_001114753.1	2022	ENG	3060	14.5795	6.74605	1.11183	0.0004	0.00971957
NM_002086.4	2885	GRB2	3303	40.1911	22.6549	0.82705	0.0004	0.00971957
NM_004196.3	8814	CDKL1	1177	3.03262	8.55833	-1.49676	0.0004	0.00971957
NM_002558.2	5023	P2RX1	2864	3.54157	1.29774	1.44839	0.0004	0.00971957
NM_001118890.1	2745	GLRX	1079	30.1375	13.2694	1.18346	0.0004	0.00971957
NM_006516.2	6513	SLC2A1	3670	16.4512	8.73311	0.91362	0.0004	0.00971957
NM_182658.1	359710	C20orf185	1454	12.1176	3.4163	1.82659	0.0004	0.00971957
NM_015120.4	7840	ALMS1	12922	16.779	32.2293	-0.94172	0.0004	0.00971957
NM_022468.4	64386	MMP25	3554	2.09125	0.342762	2.60909	0.0004	0.00971957
NM_018947.5	54205	CYCS	5538	24.6501	13.9702	0.81924	0.0004	0.00971957
NM_002831.5	5777	PTPN6	2246	18.7746	6.36875	1.55970	0.0004	0.00971957
NM_005924.4	4223	MEOX2	2371	1.25624	3.52826	-1.48984	0.0004	0.00971957
NM_033413.3	90506	LRRC46	1922	4.83268	16.8054	-1.79803	0.0004	0.00971957
NM_000343.3	6523	SLC5A1	5061	2.30198	0.800905	1.52317	0.0004	0.00971957
NM_014840.2	9891	NUAK1	6821	20.8067	11.7354	0.82618	0.0004	0.00971957
NM_015687.2	27145	FILIP1	4622	7.91738	14.749	-0.89752	0.0004	0.00971957
NM_199355.2	170692	ADAMTS18	5913	2.74299	1.04327	1.39464	0.0004	0.00971957
XM_003403417.1	339457	C1orf222	2762	0.437882	1.60667	-1.87546	0.0004	0.00971957
NM_004047.3	533	ATP6V0B	1022	35.7312	16.9467	1.07618	0.0004	0.00971957
NM_003816.2	8754	ADAM9	4099	76.7869	41.2413	0.89677	0.0004	0.00971957
NM_002627.4	5214	PFKP	2657	24.4175	13.2778	0.87890	0.0004	0.00971957
NM_032554.3	27198	HCAR1	2977	2.06794	4.83009	-1.22386	0.0004	0.00971957
NM_130386.2	81035	COLEC12	3134	31.4666	57.4232	-0.86781	0.0004	0.00971957
3507	3507	-	1486	135.167	61.5317	1.13534	0.0004	0.00971957
NM_014734.3	9766	KIAA0247	5393	20.1116	11.159	0.84983	0.0004	0.00971957
NM_006295.2	7407	VARS	4294	7.31425	3.52078	1.05481	0.0004	0.00971957
NM_206918.2	123099	DEGS2	1405	2.9938	8.04766	-1.42659	0.0004	0.00971957
TCONS_00222224	XLOC_019184	-	1701	0.27809	1.50737	-2.43841	0.0004	0.00971957
NM_002124.2	3123	HLA-DRB1	1166	207.494	112.018	0.88933	0.00045	0.0106989
NM_004362.2	1047	CLGN	2738	1.16271	3.45497	-1.57118	0.00045	0.0106989
TCONS_00229226	XLOC_018711	-	2071	0.254734	1.20096	-2.23712	0.00045	0.0106989
NM_001005738.1	2358	FPR2	1913	2.70072	0.818838	1.72169	0.00045	0.0106989
NM_022475.2	64399	HHIP	3555	2.48985	0.89534	1.47555	0.00045	0.0106989
NM_005529.5	3339	HSPG2	14288	19.8768	10.9071	0.86582	0.00045	0.0106989
NM_005940.3	4320	MMP11	2261	6.91969	2.48215	1.47912	0.00045	0.0106989
NM_001827.1	1164	CKS2	613	15.6111	5.01207	1.63909	0.00045	0.0106989
NM_000441.1	5172	SLC26A4	4930	2.74147	1.16855	1.23023	0.00045	0.0106989
NM_003256.3	7079	TIMP4	1633	1.1539	4.97141	-2.10713	0.00045	0.0106989
NM_033646.1	1005	CDH7	2728	0.202864	1.13984	-2.49025	0.00045	0.0106989

TCONS_00160811	XLOC_013647	-	2061	3.43993	1.11116	1.63032	0.00045	0.0106989
NM_000328.2	6103	RPGR	3072	8.51408	17.4858	-1.03826	0.00045	0.0106989
NM_004951.4	1880	GPR183	1785	63.8621	17.0316	1.90675	0.00045	0.0106989
NM_021237.3	58515	SELK	818	54.368	26.596	1.03155	0.00045	0.0106989
NM_139239.1	84807	NFKBID	1834	3.01556	0.961743	1.64870	0.00045	0.0106989
NM_153267.4	256691	MAMDC2	3618	5.04728	10.0174	-0.98893	0.00045	0.0106989
NM_001623.3	199	AIF1	639	68.8854	32.3159	1.09195	0.00045	0.0106989
NM_004716.2	9159	PCSK7	3484	16.9457	9.18128	0.88415	0.00045	0.0106989
NM_001105248.1	79838	TMC5	4917	18.3726	33.7987	-0.87941	0.00045	0.0106989
NM_000370.3	7274	TTPA	2633	1.74409	0.473305	1.88163	0.00045	0.0106989
NM_003446.3	7712	ZNF157	1695	1.71162	4.81562	-1.49236	0.00045	0.0106989
NM_013231.4	23768	FLRT2	7179	13.521	25.2854	-0.90311	0.00045	0.0106989
NM_138780.2	94122	SYTL5	4726	4.16459	1.08603	1.93911	0.00045	0.0106989
NM_022338.3	53838	C11orf24	2102	9.02373	3.89624	1.21164	0.00045	0.0106989
NM_015683.1	27106	ARRDC2	2503	4.21371	12.8754	-1.61146	0.00045	0.0106989
NM_024022.2	64699	TMPRSS3	2441	2.68059	6.56976	-1.29329	0.00045	0.0106989
NM_001042406.1	54511	HMGCLL1	2471	0.186472	1.10454	-2.56641	0.00045	0.0106989
TCONS_00110343	XLOC_009597	-	211	0	6.70357	#NAME?	0.0005	0.0116249
NM_001122961.1	127003	C1orf194	731	2.47585	9.08611	-1.87574	0.0005	0.0116249
NM_020980.3	366	AQP9	3021	3.48455	1.30761	1.41404	0.0005	0.0116249
TCONS_00265890	XLOC_022389	-	2835	0.21354	1.30767	-2.61443	0.0005	0.0116249
TCONS_00220393	XLOC_019116	-	2293	0.560533	2.08117	-1.89253	0.0005	0.0116249
TCONS_00137738	XLOC_011479	-	1463	1.91641	5.56089	-1.53691	0.0005	0.0116249
ENST00000558411.1	ENSG00000259641.1	RP11-279F6.3	2121	0.165951	1.19357	-2.84645	0.0005	0.0116249
NM_001197294.1	1809	DPYSL3	5480	30.2441	11.0359	1.45444	0.0005	0.0116249
NM_005675.4	8214	DGCR6	1214	2.59933	7.40628	-1.51061	0.0005	0.0116249
NM_000474.3	7291	TWIST1	1666	22.5829	42.654	-0.91745	0.0005	0.0116249
NM_004827.2	9429	ABCG2	4431	7.87008	14.5931	-0.89084	0.0005	0.0116249
NM_001142806.1	6535	SLC6A8	3114	11.4544	3.06337	1.90271	0.0005	0.0116249
NM_005911.5	4144	MAT2A	3006	93.8205	44.6326	1.07181	0.0005	0.0116249
NM_017866.5	54968	TMEM70	2085	10.9542	5.01626	1.12680	0.0005	0.0116249
NM_001845.4	1282	COL4A1	6532	64.9969	33.6388	0.95025	0.0005	0.0116249
NM_004999.3	4646	MYO6	8662	32.2758	18.063	0.83742	0.0005	0.0116249
NM_016021.2	51465	UBE2J1	4342	57.9104	31.4501	0.88076	0.0005	0.0116249
NM_198493.2	339416	ANKRD45	2671	3.99316	8.84328	-1.14705	0.0005	0.0116249
NM_000775.2	1573	CYP2J2	1857	12.5142	25.523	-1.02823	0.0005	0.0116249
ENST00000442765.1	ENSG00000234456.3	MAGI2-AS3	679	4.31956	12.4876	-1.53153	0.0005	0.0116249
NM_006531.3	8100	IFT88	2931	11.5669	23.0606	-0.99544	0.0005	0.0116249
NM_001007595.2	388125	C2CD4B	1576	1.86885	0.186148	3.32763	0.0005	0.0116249
NM_178568.2	146760	RTN4RL1	3164	0.702349	2.17195	-1.62873	0.0005	0.0116249
TCONS_00155060	XLOC_015232	-	1058	0.533702	2.81792	-2.40053	0.0005	0.0116249
NM_012329.2	23531	MMD	2723	11.5101	5.67744	1.01958	0.0005	0.0116249
NM_145307.2	219790	RTKN2	6641	3.15004	1.42742	1.14196	0.0005	0.0116249
NM_031286.3	83442	SH3BGRL3	1115	39.3848	20.0332	0.97525	0.00055	0.0124813
NM_002118.4	3109	HLA-DMB	1397	58.0641	31.0085	0.90498	0.00055	0.0124813
NM_145256.2	126364	LRRC25	2403	4.71784	1.85519	1.34656	0.00055	0.0124813
NM_000882.3	3592	IL12A	1443	0.52224	2.11017	-2.01457	0.00055	0.0124813
NM_021181.3	57823	SLAMF7	2657	27.8412	14.606	0.93066	0.00055	0.0124813
NM_007168.2	10351	ABCA8	5721	9.86593	18.1548	-0.87983	0.00055	0.0124813
NM_018689.1	57214	KIAA1199	7080	8.91771	4.80909	0.89091	0.00055	0.0124813
NM_006097.3	10398	MYL9	1187	85.8833	45.9514	0.90227	0.00055	0.0124813
NM_015000.3	23012	STK38L	5098	31.3333	17.5419	0.83690	0.00055	0.0124813
NM_004430.2	1960	EGR3	4334	2.41756	0.243423	3.31202	0.00055	0.0124813
NM_014256.3	10331	B3GNT3	2720	4.72058	1.93042	1.29005	0.00055	0.0124813

NM_003254.2	7076	TIMP1	916	127.571	61.3496	1.05618	0.00055	0.0124813
NM_006825.3	10970	CKAP4	3036	29.5724	16.6217	0.83119	0.00055	0.0124813
NM_152765.3	254778	C8orf46	3465	0.504583	1.60975	-1.67367	0.00055	0.0124813
NM_003611.2	8481	OFD1	3651	16.2443	29.4224	-0.85698	0.00055	0.0124813
TCONS_00199315	XLOC_017063	-	2547	0.200296	1.049	-2.38881	0.00055	0.0124813
NM_004961.3	2564	GABRE	3152	2.83253	6.26439	-1.14508	0.00055	0.0124813
NM_001144956.1	284485	RIIAD1	455	6.99389	20.8801	-1.57796	0.00055	0.0124813
NM_014618.2	1620	DBC1	3196	4.26732	1.64308	1.37693	0.00055	0.0124813
NM_017551.2	2894	GRID1	5849	1.36616	3.10595	-1.18491	0.00055	0.0124813
NM_031311.3	54504	CPVL	1691	54.4294	29.4203	0.88758	0.00055	0.0124813
NM_152784.3	257062	TMEM146	2513	0.470021	1.66889	-1.82809	0.00055	0.0124813
NM_031308.1	83481	EPPK1	7273	6.60137	3.57998	0.88281	0.00055	0.0124813
NM_004104.4	2194	FASN	8458	4.86973	2.48737	0.96922	0.00055	0.0124813
NM_018931.2	56125	PCDHB11	3271	1.01013	2.81169	-1.47690	0.00055	0.0124813
NM_002158.3	3344	FOXN2	5463	25.1634	14.1164	0.83396	0.00055	0.0124813
NM_025257.2	80736	SLC44A4	2634	23.7482	42.5119	-0.84005	0.00055	0.0124813
NM_002372.2	4124	MAN2A1	5128	32.7002	18.5905	0.81474	0.00055	0.0124813
NM_153269.2	140680	C20orf96	1604	4.73242	10.6097	-1.16473	0.00055	0.0124813
NM_002951.3	6185	RPN2	2508	95.2731	52.148	0.86946	0.00055	0.0124813
NM_001080556.1	146845	WDR16	1989	2.39422	16.0146	-2.74175	0.00055	0.0124813
NM_002049.3	2623	GATA1	1480	1.96682	0.422505	2.21882	0.0006	0.013408
NM_001195597.1	285733	LOC285733	2513	0.59566	1.92067	-1.68905	0.0006	0.013408
NM_018699.2	11107	PRDM5	2480	10.4146	20.0417	-0.94440	0.0006	0.013408
NM_006952.3	7348	UPK1B	2058	22.9899	11.0677	1.05465	0.0006	0.013408
NM_032283.2	84243	ZDHHC18	3139	8.09842	3.80949	1.08804	0.0006	0.013408
NM_001079906.1	55422	ZNF331	4131	21.6414	8.25556	1.39035	0.0006	0.013408
NM_006982.2	8092	ALX1	1294	1.59448	4.80268	-1.59076	0.0006	0.013408
NM_007257.5	10687	PNMA2	4846	0.294481	0.957245	-1.70072	0.0006	0.013408
NM_175922.3	285800	PRR18	2704	0.577218	2.00695	-1.79782	0.0006	0.013408
TCONS_00248440	XLOC_020911	-	5134	0.485107	1.73438	-1.83804	0.0006	0.013408
NM_000833.3	2903	GRIN2A	14446	0.293903	1.61105	-2.45459	0.0006	0.013408
NM_021814.4	60481	ELOVL5	3000	111.558	61.494	0.85928	0.0006	0.013408
TCONS_00218085	XLOC_019065	-	2851	0.717565	2.29084	-1.67469	0.0006	0.013408
NM_001039492.2	2274	FHL2	1521	32.9379	15.4451	1.09259	0.0006	0.013408
NM_016531.5	51274	KLF3	5615	44.8915	25.187	0.83377	0.0006	0.013408
NM_007261.2	11314	CD300A	1889	9.71124	4.33473	1.16371	0.0006	0.013408
NM_080489.4	27111	SDCBP2	1581	4.48281	1.00872	2.15187	0.0006	0.013408
NM_012152.2	23566	LPAR3	1721	7.94305	17.0084	-1.09848	0.0006	0.013408
NM_006120.3	3108	HLA-DMA	1122	71.571	38.8935	0.87985	0.0006	0.013408
TCONS_00062936	XLOC_006061	-	3825	1.08705	3.82407	-1.81469	0.00065	0.0142695
NM_001080522.2	57545	CC2D2A	5240	11.7128	22.2435	-0.92530	0.00065	0.0142695
TCONS_00219771	XLOC_019447	-	819	0.388823	3.07789	-2.98475	0.00065	0.0142695
NM_020711.1	57471	ERMN	3775	6.1896	2.49242	1.31230	0.00065	0.0142695
NM_138774.3	91300	C19orf22	1809	11.2593	5.18897	1.11760	0.00065	0.0142695
TCONS_00180182	XLOC_015712	-	1504	0.352317	1.70663	-2.27620	0.00065	0.0142695
ENST00000439928.2	ENSG00000228741.2	RP11-309I15.1	1721	0.959638	4.10629	-2.09727	0.00065	0.0142695
NM_153210.3	124739	USP43	4069	1.77594	4.03754	-1.18489	0.00065	0.0142695
NM_002872.3	5880	RAC2	1468	23.6746	11.4187	1.05195	0.00065	0.0142695
TCONS_00233452	XLOC_020497	-	13060	2.7618	6.60497	-1.25794	0.00065	0.0142695
NM_007335.2	9940	DLEC1	5614	3.98604	9.3766	-1.23411	0.00065	0.0142695
NM_020911.1	91584	PLXNA4	13061	0.55838	1.29855	-1.21758	0.00065	0.0142695
NM_005022.2	5216	PFN1	807	183.725	106.079	0.79241	0.00065	0.0142695
NM_032108.3	10501	SEMA6B	3942	3.40705	1.41654	1.26616	0.00065	0.0142695
NM_003026.2	6456	SH3GL2	2728	0.0779284	1.60812	-4.36709	0.00065	0.0142695

NM_004277.4	9481	SLC25A27	2986	4.92029	11.5652	-1.23297	0.00065	0.0142695
NM_058179.2	29968	PSAT1	2188	4.43589	1.73563	1.35377	0.00065	0.0142695
NM_023068.3	6614	SIGLEC1	6720	3.42888	1.67939	1.02980	0.00065	0.0142695
NM_001039580.1	79884	MAP9	7333	9.03576	16.2149	-0.84360	0.00065	0.0142695
NM_006422.2	10566	AKAP3	3020	0.423078	1.33146	-1.65401	0.00065	0.0142695
NM_001029858.3	222553	SLC35F1	4852	0.30956	1.02805	-1.73161	0.00065	0.0142695
NM_004864.2	9518	GDF15	1200	2.14643	0.431044	2.31603	0.0007	0.015106
NM_006666.1	10856	RUVBL2	1488	23.5467	42.3483	-0.84678	0.0007	0.015106
NM_000673.4	131	ADH7	2289	76.0395	37.4903	1.02023	0.0007	0.015106
NM_138468.4	130026	ICA1L	7996	3.5873	6.77273	-0.91684	0.0007	0.015106
NM_024832.3	79890	RIN3	3849	4.9286	2.26171	1.12376	0.0007	0.015106
NM_005892.3	752	FMNL1	3963	8.02866	3.74882	1.09872	0.0007	0.015106
NM_001099407.1	55184	C20orf12	3500	2.42389	5.16365	-1.09107	0.0007	0.015106
NM_014312.3	23584	VSIG2	1121	12.0712	4.81151	1.32700	0.0007	0.015106
NM_001042517.1	81624	DIAPH3	4802	1.42367	0.455508	1.64407	0.0007	0.015106
NM_000720.2	776	CACNA1D	7768	3.50119	8.05376	-1.20182	0.0007	0.015106
NM_001142946.1	29125	C11orf21	2262	4.95623	1.88486	1.39479	0.0007	0.015106
NM_002090.2	2921	CXCL3	1159	2.09477	0.313661	2.73951	0.0007	0.015106
NM_021647.6	9848	MFAP3L	6189	2.45327	5.02428	-1.03421	0.0007	0.015106
NM_002188.2	3596	IL13	1282	5.26831	0.20073	4.71401	0.0007	0.015106
NM_007054.5	11127	KIF3A	6257	9.251	16.1645	-0.80515	0.0007	0.015106
NM_030643.3	80832	APOL4	3415	3.41784	8.57044	-1.32628	0.0007	0.015106
TCONS_00122042	XLOC_011173	-	2395	0.614531	2.72025	-2.14618	0.0007	0.015106
NM_001100396.1	129852	C2orf73	1921	1.18375	3.37732	-1.51252	0.0007	0.015106
NM_000929.2	5322	PLA2G5	1894	0.164039	1.32441	-3.01324	0.0007	0.015106
NM_005158.4	27	ABL2	11940	9.50476	4.34975	1.12772	0.0007	0.015106
NM_018509.3	55379	LRRC59	2915	36.5714	20.8116	0.81333	0.00075	0.0159498
NM_014668.3	9687	GREB1	8482	0.580229	1.36422	-1.23338	0.00075	0.0159498
NM_017681.2	54830	NUP62CL	1762	1.52703	4.22254	-1.46738	0.00075	0.0159498
NM_005408.2	6357	CCL13	851	168.111	0.321955	9.02834	0.00075	0.0159498
NM_007351.2	22915	MMRN1	4969	42.9437	21.1681	1.02055	0.00075	0.0159498
NM_001008409.2	164395	TTLL9	1607	1.22099	3.41669	-1.48454	0.00075	0.0159498
NM_020347.2	54585	LZTFL1	3398	20.0918	34.8746	-0.79557	0.00075	0.0159498
NM_007107.3	6747	SSR3	3716	86.4506	48.0381	0.84770	0.00075	0.0159498
NM_016116.2	51666	ASB4	3824	0.173897	0.794438	-2.19170	0.00075	0.0159498
NM_002298.4	3936	LCP1	3790	105.542	54.3309	0.95798	0.00075	0.0159498
NM_130897.1	83657	DYNLRB2	497	8.03493	21.7803	-1.43867	0.00075	0.0159498
NM_001098814.1	6345	SRL	4214	0.50721	1.45556	-1.52092	0.00075	0.0159498
NM_032512.2	57595	PDZD4	3745	1.78328	4.0643	-1.18847	0.00075	0.0159498
TCONS_00081896	XLOC_006697	-	2735	0.908681	3.29391	-1.85795	0.0008	0.0168217
NM_173535.2	165530	CLEC4F	2475	0.916906	2.46907	-1.42912	0.0008	0.0168217
NM_019016.2	192666	KRT24	1881	1.6991	0.170365	3.31808	0.0008	0.0168217
NM_198390.2	80790	CMIP	4357	10.2118	5.58114	0.87160	0.0008	0.0168217
NM_177455.3	168620	BHLHA15	706	6.34898	1.9044	1.73719	0.0008	0.0168217
NM_001178097.2	338809	C12orf74	2369	2.06194	7.02269	-1.76802	0.0008	0.0168217
NM_001290.3	9079	LDB2	2540	25.9535	54.161	-1.06133	0.0008	0.0168217
XM_002342862.3	100288562	LOC100288562	2186	0.161353	1.39479	-3.11175	0.0008	0.0168217
NM_025087.2	80157	CWH43	2471	0.184127	1.16896	-2.66645	0.0008	0.0168217
NM_152862.1	10109	ARPC2	1448	177.541	102.67	0.79013	0.0008	0.0168217
NM_014800.10	9844	ELMO1	5180	9.37102	3.99007	1.23179	0.0008	0.0168217
NM_004212.3	9153	SLC28A2	2463	0.114975	0.82683	-2.84628	0.00085	0.0176689
NM_030632.1	80816	ASXL3	7642	0.896831	1.97286	-1.13738	0.00085	0.0176689
NM_138288.3	171546	C14orf147	2534	28.4448	15.8157	0.84681	0.00085	0.0176689
NM_012264.4	25829	TMEM184B	3611	7.47307	3.58356	1.06031	0.00085	0.0176689

XM_001726961.3	388523	ZNF728	2654	0.257463	1.24208	-2.27032	0.00085	0.0176689
NM_020813.2	57573	ZNF471	4967	4.51928	8.26025	-0.87009	0.00085	0.0176689
NM_000918.3	5034	P4HB	2578	112.846	63.2556	0.83509	0.00085	0.0176689
NM_001764.2	910	CD1B	1396	2.09678	0.468873	2.16090	0.00085	0.0176689
NM_014750.4	9787	DLGAP5	2993	3.6814	1.18664	1.63337	0.00085	0.0176689
NM_021637.2	59353	TMEM35	2071	0.797866	2.43547	-1.60998	0.00085	0.0176689
NM_005719.2	10094	ARPC3	884	240.933	137.249	0.81184	0.00085	0.0176689
NM_002417.4	4288	MKI67	12497	3.27975	1.27328	1.36504	0.00085	0.0176689
NM_001013698.2	440087	C12orf69	2104	0.812945	3.42741	-2.07589	0.00085	0.0176689
NM_138392.3	92799	SHKBP1	2365	7.78312	3.49923	1.15331	0.0009	0.0184743
NM_024532.3	79582	SPAG16	2174	5.70617	17.0394	-1.57828	0.0009	0.0184743
NM_004967.3	3381	IBSP	1591	0.127051	22.5586	-7.47213	0.0009	0.0184743
NM_013390.2	23670	TMEM2	6523	40.7194	22.4702	0.85770	0.0009	0.0184743
NM_004336.3	699	BUB1	3502	4.36725	1.89247	1.20645	0.0009	0.0184743
TCONS_00002457	XLOC_001928	-	2829	1.06488	2.8882	-1.43948	0.0009	0.0184743
NM_138812.2	143241	DYDC1	750	3.545	11.2753	-1.66931	0.0009	0.0184743
NM_184041.1	226	ALDOA	1523	157.545	81.3694	0.95321	0.0009	0.0184743
NM_182832.2	191585	PLAC4	10009	9.24676	3.55949	1.37728	0.0009	0.0184743
NM_052953.2	116135	LRRC3B	1693	0.521277	1.76688	-1.76108	0.0009	0.0184743
NM_003629.3	8503	PIK3R3	5675	35.7586	19.6031	0.86721	0.0009	0.0184743
NM_152721.5	220164	DOK6	8897	4.35317	2.37748	0.87263	0.0009	0.0184743
NM_139244.4	134957	STXBP5	9244	20.2077	10.5717	0.93470	0.0009	0.0184743
TCONS_00117328	XLOC_009816	-	4752	1.6332	0.597648	1.45033	0.0009	0.0184743
NM_014339.5	23765	IL17RA	8606	8.94948	5.14456	0.79876	0.0009	0.0184743
NM_002304.2	3955	LFNG	2058	9.81878	2.53406	1.95409	0.00095	0.0192772
NM_005625.3	6386	SDCBP	2167	94.7742	54.9317	1.04662	0.00095	0.0192772
NM_004701.3	9133	CCNB2	1549	4.77723	1.76062	1.44009	0.00095	0.0192772
NM_002965.3	6280	S100A9	577	31.6324	11.6548	1.44047	0.00095	0.0192772
NM_001178003.1	144406	WDR66	3455	5.79694	19.4299	-1.74492	0.00095	0.0192772
NM_080792.2	140885	SIRPA	3868	13.0512	5.65389	1.20686	0.00095	0.0192772
NM_032832.5	84918	LRP11	3618	31.939	55.513	-0.79751	0.00095	0.0192772
TCONS_00284457	XLOC_023884	-	3090	0.541864	1.71419	-1.66152	0.00095	0.0192772
NM_032991.2	836	CASP3	2506	38.8253	19.9246	0.96244	0.00095	0.0192772
NM_003960.3	9027	NAT8	1073	0.556262	2.59098	-2.21966	0.00095	0.0192772
NM_138554.3	7099	TLR4	5653	27.102	15.526	0.80372	0.00095	0.0192772
NM_004840.2	9459	ARHGEF6	5272	36.69	21.3491	0.78121	0.00095	0.0192772
TCONS_00210161	XLOC_018180	-	5561	2.33849	4.9003	-1.06729	0.00095	0.0192772
NM_003120.2	6688	SPI1	1405	9.41908	3.28303	1.52056	0.00095	0.0192772
NM_032229.2	84189	SLITRK6	4181	14.7531	8.15912	0.85453	0.00095	0.0192772
NM_013402.4	3992	FADS1	4420	13.798	7.75481	0.83130	0.00095	0.0192772
NM_152547.4	153579	BTNL9	3500	2.02779	4.53135	-1.16003	0.00095	0.0192772
NM_053001.2	116039	OSR2	1791	5.72828	1.68348	1.76665	0.00095	0.0192772
NM_207417.1	389799	C9orf171	1789	0.851208	2.81522	-1.72567	0.001	0.0199964
NM_144616.3	126306	JSRP1	1145	4.94652	1.56697	1.65843	0.001	0.0199964
NM_003511.2	8332	HIST1H2AL	470	24.9788	8.03838	1.63573	0.001	0.0199964
NM_002729.4	3087	HHEX	1759	8.89039	3.97839	1.16006	0.001	0.0199964
NM_006082.2	10376	TUBA1B	1753	145.728	84.1687	0.79192	0.001	0.0199964
NM_001013626.2	286187	LRRC67	1084	2.01251	5.87536	-1.54568	0.001	0.0199964
NM_001198543.1	54768	HYDIN	3122	3.02855	14.7955	-2.28846	0.001	0.0199964
NM_152558.3	23288	IQCE	6844	4.35995	8.11865	-0.89693	0.001	0.0199964
NM_021214.1	58489	FAM108C1	2360	15.6146	8.24272	0.92170	0.001	0.0199964
NM_182609.2	342926	ZNF677	3493	2.18207	4.82351	-1.14439	0.001	0.0199964
NM_002924.4	6000	RGS7	2440	2.90624	0.998181	1.54178	0.001	0.0199964
NM_001191321.1	2562	GABRB3	5543	0.299375	1.50565	-2.33036	0.001	0.0199964

NM_025212.1	80319	CXXC4	761	1.02113	4.13327	-2.01711	0.001	0.0199964
NM_025079.2	80149	ZC3H12A	2716	5.504	2.36571	1.21821	0.001	0.0199964
NM_015292.2	23344	ESYT1	4247	24.8403	11.9394	1.05696	0.001	0.0199964
NM_018058.5	55118	CRTAC1	2713	0.154487	2.46175	-3.99413	0.001	0.0199964
NM_006808.2	10952	SEC61B	562	79.7751	41.3159	0.94924	0.001	0.0199964
NM_000348.3	6716	SRD5A2	2445	4.02154	8.70016	-1.11330	0.001	0.0199964
NM_025258.2	80737	C6orf27	2914	0.893045	2.3035	-1.36702	0.00105	0.0207193
NM_138573.3	145957	NRG4	2195	1.95516	4.81632	-1.30065	0.00105	0.0207193
NM_001129908.2	729085	FAM198A	3433	1.63022	3.76574	-1.20787	0.00105	0.0207193
TCONS_00211775	XLOC_017242	-	3766	0.750793	3.44735	-2.19900	0.00105	0.0207193
NM_007178.3	11171	STRAP	1907	35.8347	20.2932	0.82036	0.00105	0.0207193
NM_000405.4	2760	GM2A	3690	33.4896	19.6752	0.76733	0.00105	0.0207193
NM_032930.2	85016	C11orf70	2198	8.8689	17.1715	-0.95319	0.00105	0.0207193
NM_000231.2	6445	SGCG	1655	0.160525	1.60134	-3.31841	0.00105	0.0207193
NM_001178088.1	8871	SYNJ2	7332	4.77869	1.83901	1.37768	0.00105	0.0207193
NM_003355.2	7351	UCP2	1646	17.3081	8.42733	1.03830	0.00105	0.0207193
NM_138788.3	120224	TMEM45B	2207	7.38134	14.4108	-0.96519	0.00105	0.0207193
NM_001123396.1	729230	CCR2	2335	18.7917	8.4493	1.15320	0.00105	0.0207193
NM_139172.1	147744	TMEM190	593	16.509	37.1796	-1.17126	0.00105	0.0207193
NM_025239.3	80380	PDCD1LG2	2418	14.0206	7.43164	0.91580	0.00105	0.0207193
NM_014584.1	30001	ERO1L	3310	49.0854	28.498	0.78443	0.00105	0.0207193
NM_020239.3	56882	CDC42SE1	3047	51.1608	27.5194	0.89459	0.00105	0.0207193
NM_003244.2	7050	TGIF1	1606	23.8996	9.31239	1.35976	0.00105	0.0207193
NM_000499.3	1543	CYP1A1	2608	0.804333	0.177293	2.18166	0.00105	0.0207193
NM_207401.1	388759	C1orf229	2258	0.391259	1.48368	-1.92299	0.00105	0.0207193
NM_004728.2	9188	DDX21	4153	48.717	28.2103	0.78820	0.00105	0.0207193
NM_019858.1	27239	GPR162	2516	1.14178	3.68162	-1.68906	0.00105	0.0207193
NM_022162.1	64127	NOD2	4485	3.87105	1.81943	1.08923	0.00105	0.0207193
TCONS_00027175	XLOC_001231	-	1229	0.734807	2.98263	-2.02115	0.00105	0.0207193
NM_001127496.1	81848	SPRY4	4941	4.25907	2.07851	1.03499	0.00105	0.0207193
NM_001079911.2	1758	DMP1	2639	0.103842	0.806231	-2.95680	0.0011	0.0214605
NM_032623.3	84709	C4orf49	1339	28.92	15.7078	0.88059	0.0011	0.0214605
NM_003051.3	6566	SLC16A1	3910	12.3099	6.45505	0.93132	0.0011	0.0214605
NM_020768.3	57528	KCTD16	5183	0.477178	1.27439	-1.41721	0.0011	0.0214605
NM_032800.2	84886	C1orf198	3761	21.5597	12.159	0.82631	0.0011	0.0214605
NM_020203.3	56955	MEPE	2007	0.36773	1.66713	-2.18065	0.0011	0.0214605
NM_006196.3	5093	PCBP1	1751	42.3965	24.2548	0.80567	0.0011	0.0214605
NM_001040274.2	221711	SYCP2L	3130	2.2879	0.876352	1.38444	0.0011	0.0214605
NM_004237.3	9319	TRIP13	2390	4.01102	8.50262	-1.08394	0.0011	0.0214605
NM_000198.3	3284	HSD3B2	1710	1.798	0.437997	2.03740	0.0011	0.0214605
NM_001085384.1	7710	ZNF154	2747	5.11825	10.2001	-0.99486	0.0011	0.0214605
NM_003841.3	8794	TNFRSF10C	1494	4.32694	1.58315	1.45054	0.0011	0.0214605
NM_001198689.1	3953	LEPR	5100	13.3861	64.7144	-2.27335	0.0011	0.0214605
NM_004931.4	926	CD8B	1433	3.90112	10.5375	-1.43358	0.0011	0.0214605
NM_005576.2	4016	LOXL1	2361	13.1862	6.55089	1.00927	0.0011	0.0214605
NM_199139.1	54739	XAF1	3569	7.4952	17.848	-1.25173	0.0011	0.0214605
NM_019073.2	54558	SPATA6	5004	8.28265	14.7221	-0.82982	0.00115	0.0222874
NM_001001852.3	415116	PIM3	2379	16.9792	9.37923	0.85623	0.00115	0.0222874
NM_016293.2	51411	BIN2	2211	22.592	12.0695	0.90444	0.00115	0.0222874
NM_030949.2	81706	PPP1R14C	2211	4.56089	9.55784	-1.06737	0.00115	0.0222874
NM_182482.2	85319	BAGE2	1567	0.233075	1.45099	-2.63817	0.00115	0.0222874
NM_002436.3	4354	MPP1	2048	20.3714	10.7929	0.91647	0.00115	0.0222874
NM_001001548.2	948	CD36	4727	14.2394	6.62186	1.10458	0.00115	0.0222874
NM_032679.2	84765	ZNF577	3096	6.02419	11.5661	-0.94106	0.00115	0.0222874

NM_007210.3	11226	GALNT6	4520	6.91363	3.5889	0.94590	0.00115	0.0222874
NM_014326.3	23604	DAPK2	2628	6.63162	3.15749	1.07058	0.00115	0.0222874
NM_006914.3	6096	RORB	3604	6.01489	11.2635	-0.90505	0.00115	0.0222874
NM_000029.3	183	AGT	2584	0.585887	1.81263	-1.62939	0.0012	0.0230244
NM_001135733.1	94241	TP53INP1	5652	49.9421	23.9929	1.05765	0.0012	0.0230244
NM_006418.4	10562	OLFM4	2919	0.254717	0.940112	-1.88394	0.0012	0.0230244
TCONS_00205099	XLOC_017564	-	3514	0.949288	0.289363	1.71396	0.0012	0.0230244
NM_001005474.2	64332	NFKBIZ	3793	17.8273	3.50004	2.34864	0.0012	0.0230244
NM_173798.2	170261	ZCCHC12	2197	0.185213	0.951334	-2.36077	0.0012	0.0230244
NM_001137560.1	441151	TMEM151B	4619	0.219879	0.786607	-1.83893	0.0012	0.0230244
NM_031916.4	83853	ROPN1L	1078	8.69182	21.6893	-1.31925	0.0012	0.0230244
NM_016307.3	51450	PRRX2	1311	13.268	6.00684	1.14327	0.0012	0.0230244
NM_005572.3	4000	LMNA	2070	58.7804	24.2547	1.27707	0.0012	0.0230244
NM_000379.3	7498	XDH	5717	3.00296	1.36851	1.13378	0.0012	0.0230244
NM_003408.1	7539	ZFP37	2752	3.1667	6.50761	-1.03915	0.0012	0.0230244
NM_001130040.1	6464	SHC1	3481	19.821	7.34791	1.43162	0.0012	0.0230244
NM_004747.3	9231	DLG5	7479	17.2647	10.0866	0.77539	0.0012	0.0230244
NM_004378.2	1381	CRABP1	772	19.3238	8.18441	1.23943	0.00125	0.02372
NM_001416.3	1973	EIF4A1	1877	213.323	119.451	0.83662	0.00125	0.02372
NM_002970.2	6303	SAT1	1064	534.363	294.105	0.86149	0.00125	0.02372
NM_016343.3	1063	CENPF	10296	7.71716	4.15555	0.89303	0.00125	0.02372
NM_005079.2	7163	TPD52	3961	27.0348	14.9664	0.85309	0.00125	0.02372
NM_001145848.1	8842	PROM1	3973	41.6027	81.0101	-0.96143	0.00125	0.02372
NM_001082537.2	79600	TCTN1	2009	9.31389	26.3227	-1.49885	0.00125	0.02372
NM_001100917.1	144448	TSPAN19	1020	6.99581	15.5608	-1.15335	0.00125	0.02372
NM_000391.3	1200	TPP1	3533	17.19	9.6488	0.83315	0.00125	0.02372
NM_012325.2	22919	MAPRE1	2623	65.122	39.1144	0.73545	0.00125	0.02372
NM_016447.2	51678	MPP6	2187	26.9018	47.1138	-0.80845	0.00125	0.02372
NM_012112.4	22974	TPX2	3662	4.74981	2.11557	1.16682	0.00125	0.02372
NM_002030.3	2359	FPR3	2517	24.1341	13.4726	0.84104	0.00125	0.02372
NM_130810.3	161582	DYX1C1	1993	4.95038	13.4104	-1.43774	0.00125	0.02372
NM_003793.3	8722	CTSF	2014	19.3251	34.152	-0.82150	0.00125	0.02372
TCONS_00197271	XLOC_018278	-	10260	0.79106	2.64333	-1.74050	0.00125	0.02372
NM_001171626.1	7422	VEGFA	3539	17.2245	3.5456	2.28036	0.00125	0.02372
NM_006745.3	6307	SC4MOL	2139	34.7417	19.17	0.85782	0.00125	0.02372
NM_003329.2	7295	TXN	508	366.501	206.195	0.82981	0.00125	0.02372
NM_178172.3	338328	GPIHBP1	2281	1.90679	4.58965	-1.26724	0.0013	0.0244278
NM_007244.2	11272	PRR4	564	10.6538	3.11021	1.77628	0.0013	0.0244278
NM_022834.4	64856	VWA1	4659	5.77136	2.96648	0.96016	0.0013	0.0244278
TCONS_00268119	XLOC_023343	-	1486	2.06149	5.3775	-1.38325	0.0013	0.0244278
NM_080657.4	91543	RSAD2	3512	4.52626	8.50485	-0.90997	0.0013	0.0244278
NM_005212.2	1448	CSN3	808	5.49987	0.356717	3.94655	0.0013	0.0244278
NM_182920.1	56999	ADAMTS9	7313	20.4174	11.7794	0.79353	0.0013	0.0244278
NM_006010.4	7873	MANF	939	54.7061	30.5012	0.84283	0.0013	0.0244278
NM_006755.1	6888	TALDO1	1243	81.0379	47.1825	0.78035	0.0013	0.0244278
NM_178554.4	339855	KY	5704	0.338184	0.912367	-1.43181	0.0013	0.0244278
TCONS_00267222	XLOC_022300	-	703	2.82386	14.9632	-2.40568	0.0013	0.0244278
NM_005475.2	10019	SH2B3	5406	14.2339	8.13118	0.80780	0.0013	0.0244278
NM_020356.3	57091	CASS4	3155	3.3797	1.31025	1.36706	0.0013	0.0244278
NM_012342.2	25805	BAMBI	1724	10.9842	20.6423	-0.91017	0.00135	0.0250595
NM_012474.4	7371	UCK2	4920	8.93271	5.06019	0.81991	0.00135	0.0250595
TCONS_00313672	XLOC_026080	-	1850	3.89835	8.62815	-1.14619	0.00135	0.0250595
NM_006259.1	5593	PRKG2	3328	0.535246	1.65502	-1.62857	0.00135	0.0250595
NM_020412.4	57132	CHMP1B	3060	54.351	28.633	0.92463	0.00135	0.0250595

NM_006219.1	5291	PIK3CB	3213	23.6889	13.9102	0.76807	0.00135	0.0250595
NM_001873.2	1363	CPE	2428	288.969	147.827	0.96700	0.00135	0.0250595
NM_018639.3	55884	WSB2	2822	27.2912	15.9357	0.77617	0.00135	0.0250595
NM_019111.4	3122	HLA-DRA	1280	1019.83	472.152	1.11100	0.00135	0.0250595
XM_003118995.1	100506191	LOC100506191	2525	3.66721	7.73331	-1.07641	0.00135	0.0250595
TCONS_00254409	XLOC_022134	-	2813	0.184067	1.0749	-2.54590	0.00135	0.0250595
NM_003270.2	7105	TSPAN6	2069	39.2457	67.0703	-0.77314	0.00135	0.0250595
NM_145239.2	112476	PRRT2	2567	1.63494	3.78784	-1.21214	0.00135	0.0250595
NM_032802.3	84888	SPPL2A	2010	48.3474	28.7016	0.75231	0.00135	0.0250595
NM_015236.4	23284	LPHN3	6125	8.42316	14.4939	-0.78301	0.00135	0.0250595
XM_003118524.1	389197	C4orf50	3184	0.540962	1.56884	-1.53610	0.00135	0.0250595
TCONS_00225715	XLOC_019189	-	4994	1.22989	0.1855	2.72904	0.0014	0.0257815
NM_033375.4	4641	MYO1C	4736	13.4527	5.69537	1.24004	0.0014	0.0257815
NM_001037329.3	1262	CNGA4	1911	1.51634	4.09179	-1.43213	0.0014	0.0257815
NM_004465.1	2255	FGF10	627	5.66094	15.1383	-1.41908	0.0014	0.0257815
NM_144999.2	201255	LRRC45	2599	3.52746	7.20279	-1.02993	0.0014	0.0257815
TCONS_00227078	XLOC_018699	-	2007	1.51764	6.24138	-2.04003	0.0014	0.0257815
NM_000476.2	203	AK1	2266	4.68506	9.31414	-0.99136	0.0014	0.0257815
TCONS_00048192	XLOC_004688	-	4458	0.382839	1.3063	-1.77068	0.0014	0.0257815
NM_003186.3	6876	TAGLN	1160	344.277	189.93	0.85811	0.0014	0.0257815
NM_181644.4	148808	MFSD4	4088	8.48492	4.50309	0.91399	0.0014	0.0257815
NM_003888.3	8854	ALDH1A2	3603	25.1261	47.9487	-0.93231	0.0014	0.0257815
NM_020741.2	57501	KIAA1257	1715	1.40731	3.6688	-1.38237	0.0014	0.0257815
NM_003862.2	8817	FGF18	1982	0.296395	1.22981	-2.05284	0.0014	0.0257815
NM_004434.2	2009	EML1	4465	7.01777	12.3336	-0.81351	0.00145	0.0264203
NM_002381.4	4148	MATN3	2583	3.62209	7.7199	-1.09176	0.00145	0.0264203
NM_013358.2	29943	PADI1	3847	2.10339	0.842389	1.32016	0.00145	0.0264203
NM_006033.2	9388	LIPG	4141	0.922035	0.292751	1.65515	0.00145	0.0264203
NM_145056.2	147906	DACT3	2834	1.85159	4.27064	-1.20569	0.00145	0.0264203
NM_017723.2	54863	C9orf167	4069	3.17954	1.47826	1.10491	0.00145	0.0264203
TCONS_00001771	XLOC_001339	-	1501	0.282988	1.46284	-2.36996	0.00145	0.0264203
NM_000346.3	6662	SOX9	3935	10.6744	5.91297	0.85220	0.00145	0.0264203
NM_003258.4	7083	TK1	1595	2.67105	0.762101	1.80935	0.00145	0.0264203
NM_006555.3	10652	YKT6	2766	21.526	12.3181	0.80530	0.00145	0.0264203
NM_001112718.1	22998	LIMCH1	6084	12.8545	24.3684	-0.92274	0.00145	0.0264203
NM_004227.3	9265	CYTH3	4469	14.1338	23.9407	-0.76032	0.00145	0.0264203
NM_005722.3	10097	ACTR2	3919	145.837	80.3251	0.86043	0.00145	0.0264203
NM_012319.3	25800	SLC39A6	3620	83.6459	49.1667	0.76661	0.00145	0.0264203
NM_003749.2	8660	IRS2	6998	7.63997	4.34961	0.81268	0.00145	0.0264203
NM_013364.4	29944	PNMA3	3751	0.485639	1.30797	-1.42938	0.00145	0.0264203
NM_024940.6	80005	DOCK5	7555	15.4059	9.03818	0.76938	0.00145	0.0264203
TCONS_00196029	XLOC_017238	-	2296	0.508796	1.62119	-1.67190	0.00145	0.0264203
NM_014790.3	9832	JAKMIP2	3338	1.1803	2.87059	-1.28219	0.00145	0.0264203
NM_030627.2	80315	CPEB4	7769	29.6778	17.4491	0.76624	0.0015	0.0270821
NM_021958.3	3142	HLX	2279	4.13149	1.7535	1.23643	0.0015	0.0270821
NM_145053.4	143630	UBQLNL	2334	0.510909	1.53987	-1.59167	0.0015	0.0270821
NM_000554.4	1406	CRX	4467	0.399264	1.12068	-1.48897	0.0015	0.0270821
NM_182538.4	201305	SPNS3	1893	2.90269	0.845872	1.77888	0.0015	0.0270821
NM_001125.2	141	ADPRH	3439	6.72959	3.35311	1.00502	0.0015	0.0270821
NM_153612.3	222537	HS3ST5	2744	0.212564	0.783482	-1.88201	0.0015	0.0270821
NM_017436.4	53947	A4GALT	2092	19.1131	10.4289	0.87398	0.0015	0.0270821
NM_153208.1	124152	IQCK	2554	12.0965	24.4537	-1.01546	0.0015	0.0270821
NM_000371.3	7276	TTR	929	2.86045	0.748366	1.93442	0.0015	0.0270821
ENST00000514532.2	ENSG00000248587.2	GDNF-AS1	2913	1.95066	0.577972	1.75489	0.0015	0.0270821

NM_005409.4	6373	CXCL11	1606	1.36176	6.67678	-2.29368	0.0015	0.0270821
NM_198481.3	284415	VSTM1	1023	9.22641	0.210054	5.45693	0.0015	0.0270821
NM_178033.1	260293	CYP4X1	2357	8.23848	14.88	-0.85292	0.0015	0.0270821
TCONS_00268841	XLOC_022907	-	1765	0.46488	1.6536	-1.83068	0.0015	0.0270821
NM_024809.3	79867	TCTN2	2910	5.83943	12.4415	-1.09126	0.0015	0.0270821
NM_001005163.2	390066	OR52D1	1065	0.485358	2.23353	-2.20220	0.00155	0.0278206
NM_004836.5	9451	EIF2AK3	4648	32.2178	19.1087	0.75363	0.00155	0.0278206
NM_000377.2	7454	WAS	1831	7.30363	3.26251	1.16263	0.00155	0.0278206
NM_001547.4	3433	IFIT2	3495	9.62328	16.7428	-0.79894	0.00155	0.0278206
TCONS_00224870	XLOC_019135	-	2003	1.79271	0.496913	1.85108	0.00155	0.0278206
NM_032784.3	84870	RSPO3	2141	2.69012	6.00178	-1.15772	0.00155	0.0278206
NM_031427.3	83544	DNAL1	8524	6.98183	13.282	-0.92780	0.0016	0.0284826
NM_006006.4	7704	ZBTB16	2407	4.35511	11.3056	-1.37626	0.0016	0.0284826
NM_032777.9	25960	GPR124	6034	14.0118	24.1094	-0.78295	0.0016	0.0284826
NM_000246.3	4261	CIITA	4654	11.3234	6.35282	0.83384	0.0016	0.0284826
NM_001678.3	482	ATP1B2	3332	0.995796	2.46752	-1.30914	0.0016	0.0284826
NM_021064.4	8969	HIST1H2AG	498	59.9713	29.175	1.03954	0.0016	0.0284826
NM_021109.3	7114	TMSB4X	629	684.607	374.059	0.87201	0.0016	0.0284826
NM_014714.3	9742	IFT140	5268	5.37717	10.3984	-0.95145	0.0016	0.0284826
NM_012463.3	23545	ATP6V0A2	6542	14.3382	8.6965	0.72136	0.0016	0.0284826
NM_005524.3	3280	HES1	1461	27.2982	14.9181	0.87174	0.0016	0.0284826
NM_182633.1	349075	ZNF713	2065	5.14634	10.3758	-1.01160	0.0016	0.0284826
NM_001122962.1	284759	SIRPB2	2833	2.8641	0.815921	1.81158	0.0016	0.0284826
NM_006065.3	10326	SIRPB1	2413	1.93664	0.485706	1.99540	0.0016	0.0284826
NM_018102.3	55713	ZNF334	2455	3.58018	8.65895	-1.27416	0.0016	0.0284826
NM_001172779.1	151827	LRRC34	1840	5.01262	11.8738	-1.24415	0.0016	0.0284826
NM_023073.3	65250	C5orf42	11199	19.4587	33.5119	-0.78426	0.0016	0.0284826
NM_002952.3	6187	RPS2	942	673.144	372.328	0.85434	0.0016	0.0284826
NM_024848.1	79906	MORN1	1641	3.20908	7.25234	-1.17629	0.0016	0.0284826
NM_018043.5	55107	ANO1	4807	11.5602	6.33064	0.86874	0.00165	0.0290957
NM_001025598.1	257106	ARHGAP30	4425	15.2027	8.74023	0.79858	0.00165	0.0290957
NM_003332.3	7305	TYROBP	591	29.4854	11.7458	1.32786	0.00165	0.0290957
NM_001160306.1	8867	SYNJ1	6942	7.81988	4.52052	0.79066	0.00165	0.0290957
TCONS_00132177	XLOC_010677	-	3357	3.1968	1.3971	1.19420	0.00165	0.0290957
NM_002577.4	5062	PAK2	6139	35.8223	21.1269	0.76178	0.00165	0.0290957
NM_000201.2	3383	ICAM1	3246	12.0421	6.61486	0.86430	0.00165	0.0290957
NM_001163.3	320	APBA1	6584	2.69559	1.32419	1.02549	0.00165	0.0290957
NM_005067.5	6478	SIAH2	2620	22.2064	12.6633	0.81032	0.00165	0.0290957
NM_002839.3	5789	PTPRD	10110	3.64831	7.55436	-1.05008	0.00165	0.0290957
NM_001042599.1	2066	ERBB4	11875	1.83523	7.40774	-2.01307	0.00165	0.0290957
NM_001080444.1	150084	IGSF5	2066	0.115253	0.907261	-2.97671	0.00165	0.0290957
NM_005688.2	10057	ABCC5	5845	17.1981	9.1921	0.90378	0.00165	0.0290957
NM_024581.4	79632	FAM184A	4044	2.7369	7.33535	-1.42232	0.00165	0.0290957
NM_177972.2	7275	TUB	6158	4.99689	11.2715	-1.17358	0.00165	0.0290957
NM_001042784.1	339965	CCDC158	3626	0.588166	1.55677	-1.40426	0.0017	0.0297668
NM_002405.3	4242	MFNG	2129	7.10347	3.27754	1.11591	0.0017	0.0297668
NM_007223.1	11245	GPR176	2816	8.41638	4.19125	1.00582	0.0017	0.0297668
NM_001242908.1	284654	RSPO1	2910	6.16402	12.2863	-0.99510	0.0017	0.0297668
NM_000422.2	3872	KRT17	1574	89.3656	52.2859	0.77330	0.0017	0.0297668
NM_001846.2	1284	COL4A2	6264	53.7907	30.0001	0.84239	0.0017	0.0297668
NM_001114086.1	53405	CLIC5	5976	1.42588	4.12619	-1.53296	0.0017	0.0297668
NM_022443.4	4291	MLF1	2202	8.95539	18.4954	-1.04633	0.0017	0.0297668
NM_144993.1	200424	TET3	10983	8.97817	5.34272	0.74885	0.0017	0.0297668
NM_004949.3	1824	DSC2	5245	4.18563	1.10952	1.91550	0.0017	0.0297668

NM_002787.4	5683	PSMA2	1466	42.1195	24.3587	0.79005	0.0017	0.0297668
NM_005442.2	8320	EOMES	2754	2.49774	5.42319	-1.11852	0.0017	0.0297668
NM_001882.3	1393	CRHBP	1838	0.784935	2.22215	-1.50131	0.0017	0.0297668
NM_001481.2	2622	GAS8	3185	4.58849	8.68878	-0.92113	0.00175	0.0304286
NM_001004067.3	408050	NOMO3	4316	2.47637	0.990041	1.32267	0.00175	0.0304286
NM_138706.3	192134	B3GNT6	2513	0.881246	0.168465	2.38710	0.00175	0.0304286
NM_006845.3	11004	KIF2C	2881	2.40072	0.887555	1.43556	0.00175	0.0304286
TCONS_00222281	XLOC_019267	-	1162	0.725492	2.61527	-1.84993	0.00175	0.0304286
TCONS_00198995	XLOC_018278	-	7767	0.679529	2.95969	-2.12284	0.00175	0.0304286
NM_001039573.2	6397	SEC14L1	3017	41.5329	18.8912	1.13654	0.00175	0.0304286
NM_080388.1	140576	S100A16	1068	33.8482	18.3125	0.88625	0.00175	0.0304286
NM_004334.2	683	BST1	1480	9.51768	4.11828	1.20857	0.00175	0.0304286
NM_033119.4	85407	NKD1	8716	4.78997	8.38439	-0.80769	0.00175	0.0304286
NM_021071.2	420	ART4	1371	0.704189	2.45888	-1.80397	0.00175	0.0304286
NM_175862.4	942	CD86	2727	13.1315	6.90471	0.92738	0.00175	0.0304286
NM_205839.2	7940	LST1	764	18.0237	2.39104	2.91418	0.00175	0.0304286
NM_002754.3	5603	MAPK13	1888	12.2112	6.09402	1.00274	0.00175	0.0304286
TCONS_00202755	XLOC_017959	-	2625	0.173812	0.755553	-2.12000	0.0018	0.0310732
NM_001001794.3	414918	FAM116B	2006	2.73041	6.09827	-1.15928	0.0018	0.0310732
NM_004309.4	396	ARHGDIA	1906	20.7878	11.6734	0.83252	0.0018	0.0310732
NM_001122951.2	2532	DARC	1242	14.9191	5.26575	1.50245	0.0018	0.0310732
NM_003005.3	6403	SELP	3172	28.0713	16.3145	0.78294	0.0018	0.0310732
NM_000063.4	717	C2	2838	15.8909	7.39082	1.10440	0.0018	0.0310732
NM_173567.4	253152	EPHX4	1443	1.7312	0.437835	1.98332	0.0018	0.0310732
NM_003115.4	6675	UAP1	2327	75.8248	44.9218	0.75525	0.0018	0.0310732
NM_144650.2	137872	ADHFE1	1895	2.5768	5.88896	-1.19244	0.0018	0.0310732
NM_005266.5	2702	GJA5	3177	6.8794	2.81517	1.28906	0.0018	0.0310732
NM_014302.3	23480	SEC61G	466	140.836	77.2675	0.86609	0.0018	0.0310732
NM_020733.1	57493	HEG1	9156	54.613	27.0621	1.01297	0.0018	0.0310732
NM_003246.2	7057	THBS1	5790	511.283	108.638	2.23459	0.0018	0.0310732
NM_145287.3	162655	ZNF519	6810	2.33647	4.32797	-0.88936	0.0018	0.0310732
NM_003921.4	8915	BCL10	3105	15.7959	9.04308	0.80467	0.00185	0.0317167
NM_001949.3	1871	E2F3	5009	4.31557	2.23697	0.94801	0.00185	0.0317167
NM_005347.4	3309	HSPA5	3970	107.736	62.5368	0.78472	0.00185	0.0317167
NM_207361.4	341640	FREM2	16163	3.21662	5.68093	-0.82058	0.00185	0.0317167
NM_001080383.1	10052	GJC1	7640	5.39705	1.88381	1.51851	0.00185	0.0317167
NM_017639.3	54798	DCHS2	8912	4.76398	1.68035	1.50341	0.00185	0.0317167
NM_025106.3	80176	SPSB1	3118	13.3125	7.36462	0.85410	0.00185	0.0317167
NM_018217.2	55741	EDEM2	1913	13.4159	6.5724	1.02945	0.00185	0.0317167
TCONS_00284596	XLOC_024098	-	1487	1.25694	0.287874	2.12640	0.00185	0.0317167
ENST00000608794.1	ENSG00000272870.1	RP11-798M19.6	587	2.20942	7.03951	-1.67180	0.0019	0.0323187
NM_032437.2	84455	EFCAB7	2361	7.87108	14.3294	-0.86435	0.0019	0.0323187
NM_021229.3	59277	NTN4	3615	25.9555	43.1475	-0.73324	0.0019	0.0323187
NM_052909.3	153478	PLEKHG4B	11515	0.604714	1.24942	-1.04693	0.0019	0.0323187
NM_006335.2	10440	TIMM17A	1659	27.8967	15.938	0.80763	0.0019	0.0323187
NM_014752.2	9789	SPCS2	2708	20.437	11.7304	0.80093	0.0019	0.0323187
NM_022777.2	64792	RABL5	2428	6.96595	13.078	-0.90875	0.0019	0.0323187
NM_006063.2	10324	KBTBD10	2464	1.53299	3.80224	-1.31050	0.0019	0.0323187
NM_001135148.1	64116	SLC39A8	2607	25.458	11.8874	1.09869	0.0019	0.0323187
NM_001002.3	6175	RPLP0	1204	981.226	500.079	0.97243	0.0019	0.0323187
NM_006741.3	5502	PPP1R1A	1834	0.118861	1.20575	-3.34258	0.0019	0.0323187
NM_144968.1	158787	RIBC1	1949	1.0521	4.57366	-2.12008	0.0019	0.0323187
NM_001849.3	1292	COL6A2	3439	84.7591	38.1024	1.15349	0.0019	0.0323187
NM_001091.2	26	ABP1	2428	1.04662	0.3053	1.77744	0.00195	0.0329693

NM_021179.1	57821	C1orf114	1866	5.01895	21.8987	-2.12539	0.00195	0.0329693
NM_001005850.2	90485	ZNF835	2774	0.526694	1.54335	-1.55103	0.00195	0.0329693
NM_080659.2	91894	C11orf52	1140	6.17407	13.3858	-1.11641	0.00195	0.0329693
NM_033031.2	85417	CCNB3	4513	0.339658	0.979016	-1.52725	0.00195	0.0329693
NM_001193628.1	147670	LOC147670	2542	0.955016	2.38876	-1.32266	0.002	0.0335868
NM_001127651.2	4688	NCF2	2249	20.6553	5.7269	1.85069	0.002	0.0335868
NM_001645.3	341	APOC1	461	23.4597	8.67208	1.43574	0.002	0.0335868
NM_005319.3	3006	HIST1H1C	732	215.547	127.629	0.75605	0.002	0.0335868
TCONS_00087932	XLOC_007810	-	4484	0.652519	1.99556	-1.61270	0.002	0.0335868
NM_019015.1	54480	CHPF2	3970	13.83	7.91414	0.80529	0.002	0.0335868
TCONS_00177473	XLOC_013688	-	1368	0.30709	1.79126	-2.54424	0.002	0.0335868
NM_002068.2	2769	GNA15	2132	10.5594	5.41581	0.96328	0.002	0.0335868
NM_021963.3	4674	NAP1L2	2576	2.73541	5.7586	-1.07396	0.002	0.0335868
NM_007256.4	11309	SLCO2B1	4345	18.7533	10.9451	0.77686	0.002	0.0335868
NM_144508.3	57082	CASC5	7607	3.50902	1.82641	0.94206	0.002	0.0335868
NM_183075.2	113612	CYP2U1	4752	6.00529	10.4134	-0.79414	0.002	0.0335868
NM_052923.1	114821	SCAND3	4877	0.629546	1.53673	-1.28748	0.002	0.0335868
NM_000418.2	3566	IL4R	3668	17.09	10.0018	0.77289	0.002	0.0335868
NM_014139.2	11280	SCN11A	6500	0.272002	0.728756	-1.42182	0.00205	0.0341961
NM_152312.3	120071	GYLTL1B	2528	1.96602	0.746638	1.39679	0.00205	0.0341961
TCONS_00254192	XLOC_021827	-	2082	0.186104	2.38035	-3.67699	0.00205	0.0341961
NM_207362.2	343990	C2orf55	3869	5.95742	10.7506	-0.85166	0.00205	0.0341961
NM_001161573.1	23764	MAFF	2224	5.19088	1.3189	1.97665	0.00205	0.0341961
NM_001005567.2	282763	OR51B5	1370	0.388468	1.44035	-1.89055	0.00205	0.0341961
NM_152588.1	160335	TMTC2	4840	48.6985	28.9545	0.75009	0.00205	0.0341961
NM_016370.2	51209	RAB9B	3799	1.1384	2.56204	-1.17029	0.0021	0.0348574
NM_002117.4	3107	HLA-C	1525	271.276	154.551	0.81167	0.0021	0.0348574
NM_004567.2	5210	PFKFB4	3490	2.9009	1.2401	1.22605	0.0021	0.0348574
NM_004255.3	9377	COX5A	770	44.426	23.8837	0.89538	0.0021	0.0348574
NM_001025266.1	285382	C3orf70	5901	4.42686	2.2555	0.97284	0.0021	0.0348574
NM_001100623.1	55041	PLEKHB2	4308	29.5845	17.3336	0.77127	0.0021	0.0348574
NM_031924.4	83861	RSPH3	2177	11.6865	20.5768	-0.81618	0.0021	0.0348574
NM_002635.3	5250	SLC25A3	1678	139.515	82.7466	0.75364	0.0021	0.0348574
NM_012445.3	10417	SPON2	1865	12.5583	5.8464	1.10302	0.0021	0.0348574
NM_001164315.1	375248	ANKRD36	6269	16.2714	27.2513	-0.74399	0.0021	0.0348574
NM_001037671.3	338809	C12orf74	1858	3.24662	8.74731	-1.42990	0.0021	0.0348574
NM_152361.1	126272	EID2B	1865	1.74682	4.13907	-1.24457	0.00215	0.0355296
NM_004177.4	6809	STX3	6475	9.48532	5.36261	0.82276	0.00215	0.0355296
NM_014037.2	28968	SLC6A16	2936	0.414459	1.22907	-1.56826	0.00215	0.0355296
NM_006343.2	10461	MERTK	3626	15.3279	25.5078	-0.73478	0.00215	0.0355296
NM_022059.2	58191	CXCL16	2324	13.6643	6.71544	1.02486	0.00215	0.0355296
NM_024926.2	79989	TTC26	2151	7.88482	14.4445	-0.87337	0.00215	0.0355296
NM_173562.3	222658	KCTD20	5018	38.9632	23.7523	0.71405	0.00215	0.0355296
NM_001242729.1	54848	ARHGEF38	5456	2.68672	5.34409	-0.99210	0.00215	0.0355296
NM_002178.2	3489	IGFBP6	966	45.0021	77.1555	-0.77778	0.00215	0.0355296
NM_020779.3	57539	WDR35	6916	10.8128	17.9311	-0.72972	0.0022	0.0361165
NM_016006.4	51099	ABHD5	5353	11.4924	6.88171	0.73984	0.0022	0.0361165
NM_004099.4	2040	STOM	3074	119.299	68.3688	0.80317	0.0022	0.0361165
NM_003304.4	7220	TRPC1	4069	7.45559	12.8979	-0.79074	0.0022	0.0361165
NM_005250.2	2300	FOXL1	3190	0.520875	1.40835	-1.43500	0.0022	0.0361165
NM_004394.2	1611	DAP	2342	36.6997	21.8193	0.75016	0.0022	0.0361165
NM_001031709.2	55328	RNLS	2416	5.5012	11.2693	-1.03457	0.0022	0.0361165
TCONS_00254761	XLOC_021222	-	765	1.38939	4.28591	-1.62516	0.0022	0.0361165
NM_145913.3	160728	SLC5A8	3286	60.7557	35.3996	0.77929	0.0022	0.0361165

NM_001998.2	2199	FBLN2	4183	9.56368	4.47021	1.09722	0.0022	0.0361165
NM_014365.2	26353	HSPB8	2002	10.5962	5.46362	0.95561	0.0022	0.0361165
NM_006986.3	9500	MAGED1	2744	40.0258	21.5942	0.89029	0.0022	0.0361165
NM_016619.2	51316	PLAC8	1403	48.4397	82.614	-0.77020	0.0022	0.0361165
NM_018482.2	50807	ASAP1	6042	29.2342	17.4535	0.74414	0.00225	0.0367046
NM_016113.4	51393	TRPV2	2808	6.88418	3.49456	0.97818	0.00225	0.0367046
NM_014441.2	27180	SIGLEC9	1718	2.33866	0.745135	1.65011	0.00225	0.0367046
NM_003714.2	8614	STC2	5343	1.74891	0.756937	1.20821	0.00225	0.0367046
NM_153498.2	57118	CAMK1D	2242	15.4831	7.49063	1.04754	0.00225	0.0367046
NM_014298.3	23475	QPRT	1555	6.08016	2.60317	1.22384	0.00225	0.0367046
NM_033105.4	85479	DNAJC5B	1380	0.717161	2.29644	-1.67903	0.00225	0.0367046
NM_182603.2	338699	ANKRD42	2562	6.16383	11.2156	-0.86361	0.00225	0.0367046
NM_001145636.1	339541	C1orf228	1685	2.17127	5.01943	-1.20898	0.00225	0.0367046
NM_006759.3	7360	UGP2	2168	69.2424	27.9467	1.30898	0.00225	0.0367046
TCONS_00012089	XLOC_000775	-	4929	0.82346	1.84909	-1.16705	0.0023	0.0373663
NM_000905.3	4852	NPY	560	1.11647	5.04522	-2.17597	0.0023	0.0373663
NM_002146.4	3213	HOXB3	3618	0.828945	2.11612	-1.35207	0.0023	0.0373663
NM_006178.3	4905	NSF	3983	29.5909	18.1373	0.70620	0.0023	0.0373663
NM_153714.2	256815	C10orf67	2943	0.531848	1.52933	-1.52381	0.0023	0.0373663
NM_001190201.1	283848	CES4A	2167	2.60357	7.01003	-1.42893	0.00235	0.0379132
TCONS_00253855	XLOC_021286	-	1437	0.466937	1.89396	-2.02011	0.00235	0.0379132
NM_006843.2	10993	SDS	1605	1.22725	0.322729	1.92704	0.00235	0.0379132
NM_001166114.1	10908	PNPLA6	4389	8.08424	3.49178	1.21115	0.00235	0.0379132
NM_005557.3	3868	KRT16	1718	1.56211	0.1655	3.23859	0.00235	0.0379132
NM_021960.4	4170	MCL1	4085	214.979	75.5478	1.50873	0.00235	0.0379132
NM_001142958.1	201456	FBXO15	1721	3.4157	8.4114	-1.30016	0.00235	0.0379132
NM_001793.4	1001	CDH3	4276	15.7556	8.94233	0.81715	0.00235	0.0379132
NM_002728.4	5553	PRG2	857	4.98496	1.23998	2.00726	0.00235	0.0379132
NM_003027.3	6457	SH3GL3	1695	0.130498	1.0983	-3.07317	0.00235	0.0379132
NM_004069.3	1175	AP2S1	890	28.4455	14.4736	0.97478	0.00235	0.0379132
NM_172365.1	145376	C14orf50	1405	4.56697	9.90947	-1.11757	0.00235	0.0379132
NM_033054.2	64005	MYO1G	3279	6.89137	3.57664	0.94619	0.0024	0.03848
NM_006588.2	27233	SULT1C4	1664	1.17284	3.13391	-1.41796	0.0024	0.03848
NM_001145073.1	389856	USP27X	2700	2.23496	4.75066	-1.08788	0.0024	0.03848
NM_014287.3	23420	NOMO1	4247	17.1197	10.1733	0.75087	0.0024	0.03848
NM_005806.2	10215	OLIG2	2488	1.08761	0.295285	1.88098	0.0024	0.03848
NM_015210.3	23255	CCDC165	6092	1.90827	0.900782	1.08301	0.0024	0.03848
NM_145018.3	220042	C11orf82	3565	2.03961	0.856252	1.25218	0.0024	0.03848
NM_003814.4	8748	ADAM20	2792	0.87386	2.15419	-1.30167	0.0024	0.03848
NM_004195.2	8784	TNFRSF18	1200	2.07902	0.534908	1.95855	0.0024	0.03848
NM_152649.2	197259	MLKL	2506	12.4706	6.76554	0.88225	0.0024	0.03848
NM_031476.3	83716	CRISPLD2	4589	32.2142	18.6098	0.79163	0.00245	0.039114
NM_001136572.1	441317	FAM90A7	1957	0.42689	1.36013	-1.67181	0.00245	0.039114
NM_018957.3	23616	SH3BP1	2576	5.86324	2.90004	1.01562	0.00245	0.039114
NM_152773.4	255758	TCTEX1D2	679	9.06833	20.028	-1.14311	0.00245	0.039114
NM_003937.2	8942	KYNU	1672	16.7319	6.80954	1.29697	0.00245	0.039114
NM_005321.2	3008	HIST1H1E	785	161.519	98.6839	0.71082	0.00245	0.039114
NM_018485.1	27202	GPR77	1287	2.52364	0.753032	1.74472	0.00245	0.039114
NM_030965.1	81849	ST6GALNAC5	2032	1.68723	0.489561	1.78510	0.00245	0.039114
NM_017491.3	9948	WDR1	3143	73.5608	45.0292	0.70808	0.00245	0.039114
TCONS_00281560	XLOC_024256	-	1326	0.493882	1.97159	-1.99712	0.00245	0.039114
NM_001624.2	202	AIM1	7553	29.6105	17.8316	0.73168	0.0025	0.0397144
NM_006600.2	10726	NUDC	1327	30.6356	51.7624	-0.75670	0.0025	0.0397144
NM_012310.4	24137	KIF4A	4501	1.8999	0.800005	1.24784	0.0025	0.0397144

XM_003403513.1	220416	LRRC63	2187	0.369106	2.60232	-2.81769	0.0025	0.0397144
NM_153695.3	195828	ZNF367	3704	3.39158	1.5645	1.11625	0.0025	0.0397144
NM_001040456.1	57414	RHBDD2	1756	16.3382	7.59804	1.10455	0.0025	0.0397144
NM_002526.3	4907	NT5E	4068	16.6419	27.6155	-0.73066	0.00255	0.0402995
NM_139055.2	170689	ADAMTS15	5676	2.80396	1.3738	1.02930	0.00255	0.0402995
NM_005380.6	4681	NBL1	2026	89.8563	166.887	-0.89318	0.00255	0.0402995
NM_001001389.1	960	CD44	5603	77.5753	8.38604	3.20954	0.00255	0.0402995
ENST00000418025.1	ENSG00000228162.1	AC097713.3	562	4.45833	1.06907	2.06015	0.00255	0.0402995
NM_006211.3	5179	PENK	1203	2.12553	0.510716	2.05723	0.00255	0.0402995
NM_001775.2	952	CD38	1491	23.3754	12.6906	0.88123	0.00255	0.0402995
NM_152665.2	200132	TCTEX1D1	2264	6.17739	11.4523	-0.89057	0.00255	0.0402995
NM_153712.4	150465	TTL	5164	10.8557	6.27765	0.79016	0.00255	0.0402995
NM_001102469.1	643418	LIPN	1197	7.02151	0.0708131	6.63162	0.0026	0.040888
NM_022153.1	64115	C10orf54	4774	20.5515	11.162	0.88064	0.0026	0.040888
NM_025204.2	80305	TRABD	2315	9.16677	4.75302	0.94757	0.0026	0.040888
TCONS_00282968	XLOC_023779	-	4918	1.34307	4.54485	-1.75870	0.0026	0.040888
NM_005013.2	4925	NUCB2	1612	123.968	215.883	-0.80028	0.0026	0.040888
NM_003855.2	8809	IL18R1	3522	10.1074	5.72651	0.81968	0.0026	0.040888
NM_001242759.1	100505676	LOC100505676	4368	0.966707	2.18847	-1.17878	0.0026	0.040888
NM_022044.2	23753	SDF2L1	874	12.5753	5.65895	1.15199	0.0026	0.040888
NM_003387.4	7456	WIPF1	4585	33.6363	19.2123	0.80799	0.0026	0.040888
XM_003403440.1	100652802	LOC100652802	575	7.8789	0.189954	5.37427	0.0026	0.040888
NM_001080836.2	644890	MEIG1	625	0.515749	3.75448	-2.86387	0.0026	0.040888
NM_138328.2	162494	RHBDL3	4661	0.336494	0.892206	-1.40679	0.00265	0.0415288
NM_000057.2	641	BLM	4528	3.41435	1.66242	1.03833	0.00265	0.0415288
NM_032564.3	84649	DGAT2	2452	2.62874	1.11256	1.24049	0.00265	0.0415288
NM_015549.1	26030	PLEKHG3	4400	7.27324	4.13705	0.81400	0.00265	0.0415288
NM_138736.2	2775	GNAO1	6211	3.51673	1.72249	1.02974	0.00265	0.0415288
TCONS_00021576	XLOC_001928	-	3961	0.820436	1.94824	-1.24771	0.0027	0.0421065
NM_018984.3	54434	SSH1	8535	10.1446	6.06703	0.74165	0.0027	0.0421065
NM_139018.3	146722	CD300LF	1759	10.171	1.59191	2.67563	0.0027	0.0421065
NM_015932.5	51371	POMP	1462	43.8595	25.9147	0.75912	0.0027	0.0421065
NM_001144892.1	2246	FGF1	3660	5.53292	0.814182	2.76462	0.0027	0.0421065
NM_001660.3	378	ARF4	1740	132.994	81.3809	0.70860	0.0027	0.0421065
NM_003059.2	6583	SLC22A4	2192	8.28216	14.7167	-0.82938	0.0027	0.0421065
NM_198391.2	23767	FLRT3	4024	4.14387	11.3487	-1.45348	0.0027	0.0421065
NM_145273.3	146894	CD300LG	2786	0.0748997	0.777111	-3.37509	0.00275	0.0426687
NM_001172626.1	270	AMPD1	2395	5.45253	1.01457	2.42606	0.00275	0.0426687
NM_005990.3	6793	STK10	6019	13.4354	8.05933	0.73731	0.00275	0.0426687
NM_016323.2	51191	HERC5	3513	5.74998	9.90241	-0.78422	0.00275	0.0426687
TCONS_00287275	XLOC_023844	-	5550	3.35262	7.50074	-1.16174	0.00275	0.0426687
NM_014397.5	10783	NEK6	2635	21.234	7.59237	1.48375	0.0028	0.0432452
NM_001242531.1	55769	ZNF83	2965	6.38417	19.8156	-1.63407	0.0028	0.0432452
NM_001669.2	414	ARSD	5159	11.5384	18.9618	-0.71665	0.0028	0.0432452
NM_021227.2	58505	OSTC	1078	83.2892	49.9734	0.73697	0.0028	0.0432452
NM_012092.3	29851	ICOS	2645	6.43773	3.03202	1.08627	0.0028	0.0432452
TCONS_00219619	XLOC_019274	-	1508	2.00295	4.91021	-1.29366	0.0028	0.0432452
NM_001145966.1	4288	MKI67	11417	2.66654	0.782924	1.76803	0.0028	0.0432452
NM_030796.3	81552	VOPP1	2944	46.6351	28.2484	0.72325	0.0028	0.0432452
NM_022909.3	64946	CENPH	1389	6.46662	2.82724	1.19362	0.00285	0.0437663
XM_001722384.2	100130589	LOC100130589	1356	0.186654	1.17206	-2.65061	0.00285	0.0437663
NM_032849.3	84935	C13orf33	2390	2.79697	1.21091	1.20777	0.00285	0.0437663
NM_173464.3	91133	L3MBTL4	3587	4.81802	8.61905	-0.83909	0.00285	0.0437663
XM_003403515.1	389422	C6orf183	2081	1.23713	3.09749	-1.32411	0.00285	0.0437663

NM_175726.2	3568	IL5RA	2346	8.17471	17.5844	-1.10505	0.00285	0.0437663
NM_138408.3	112495	GTF3C6	950	36.4937	20.0765	0.86214	0.00285	0.0437663
NM_153649.3	7170	TPM3	3212	99.2458	57.4959	0.78755	0.00285	0.0437663
NM_001841.2	1269	CNR2	1775	2.02296	0.713825	1.50282	0.00285	0.0437663
NM_024324.3	79174	CRELD2	1428	19.9681	10.57	0.91773	0.00285	0.0437663
NM_013416.3	4689	NCF4	1643	7.01952	2.3205	1.59694	0.0029	0.0443924
NM_002547.2	4983	OPHN1	7531	29.5533	51.5959	-0.80394	0.0029	0.0443924
NM_005558.3	3898	LAD1	2839	5.64895	2.73005	1.04906	0.0029	0.0443924
NM_003633.2	8507	ENC1	5517	15.4701	9.47383	0.70746	0.0029	0.0443924
NM_001199622.1	135112	NCOA7	4052	27.0207	3.32381	3.02315	0.0029	0.0443924
NM_006344.2	10462	CLEC10A	1699	14.3431	2.22441	2.68887	0.0029	0.0443924
NM_018424.2	54566	EPB41L4B	3783	8.69365	15.1216	-0.79858	0.0029	0.0443924
NM_002524.4	4893	NRAS	4454	27.8864	17.1298	0.70306	0.00295	0.0449329
NM_018487.2	55365	TMEM176A	1038	30.2817	8.92259	1.76291	0.00295	0.0449329
NM_000714.4	706	TSPO	866	31.503	16.6691	0.91831	0.00295	0.0449329
NM_001001872.2	145407	C14orf37	3082	4.74715	8.73804	-0.88025	0.00295	0.0449329
NM_006863.1	11024	LILRA1	1910	1.31357	0.395172	1.73294	0.00295	0.0449329
NM_016332.2	51734	SEPX1	1367	7.3396	3.32089	1.14413	0.00295	0.0449329
NM_001003.2	6176	RPLP1	512	1036.09	623.104	0.73361	0.00295	0.0449329
NM_004133.4	3174	HNF4G	4101	1.70434	3.51507	-1.04434	0.00295	0.0449329
TCONS_00183144	XLOC_015454	-	851	2.38247	0.590287	2.01297	0.00295	0.0449329
NM_152515.3	150468	CKAP2L	3273	1.98622	0.845568	1.23203	0.00295	0.0449329
NM_182472.2	2044	EPHA5	7792	0.269495	0.787961	-1.54786	0.003	0.0454886
NM_144705.2	150483	TEKT4	1455	1.08178	3.08735	-1.51296	0.003	0.0454886
NM_144590.2	118932	ANKRD22	3986	0.682676	1.79234	-1.39257	0.003	0.0454886
NM_032140.1	84080	C16orf48	1538	4.70851	9.48455	-1.01031	0.003	0.0454886
NM_006682.2	10875	FGL2	4268	100.65	37.1138	1.43932	0.003	0.0454886
NM_024997.2	80063	ATF7IP2	3597	5.53363	9.57063	-0.79039	0.003	0.0454886
TCONS_00080459	XLOC_006697	-	1752	0.783673	3.70694	-2.24191	0.003	0.0454886
NM_021197.2	58189	WFDC1	1378	10.2396	4.80341	1.09203	0.00305	0.04606
NM_001011720.1	389668	XKR9	3174	3.11323	5.97591	-0.94075	0.00305	0.04606
NM_001010919.1	441168	FAM26F	1109	3.51969	8.11979	-1.20599	0.00305	0.04606
NM_020747.2	57507	ZNF608	5645	8.19366	13.5527	-0.72600	0.00305	0.04606
NM_005801.3	10209	EIF1	1326	197.258	121.592	0.69804	0.00305	0.04606
NM_002610.3	5163	PDK1	4576	34.0586	9.17787	1.89179	0.00305	0.04606
NM_031271.3	56154	TEX15	10112	0.869623	0.3842	1.17853	0.0031	0.0465955
NM_001130414.1	321	APBA2	3603	2.19784	4.34045	-0.98176	0.0031	0.0465955
NM_001543.4	3340	NDST1	8030	17.1326	10.5834	0.69495	0.0031	0.0465955
TCONS_00065904	XLOC_006375	-	1374	0.29261	1.27872	-2.12765	0.0031	0.0465955
NM_001080393.1	727936	GXYLT2	1547	10.1353	18.4843	-0.86691	0.0031	0.0465955
NM_004655.3	8313	AXIN2	4234	12.4475	20.4522	-0.71640	0.0031	0.0465955
NM_003013.2	6423	SFRP2	1990	139.326	83.2395	0.74312	0.0031	0.0465955
NM_004091.3	1870	E2F2	5193	1.10074	0.457063	1.26801	0.0031	0.0465955
NM_152677.2	201516	ZSCAN4	2246	0.232173	0.936532	-2.01213	0.0031	0.0465955
NM_000104.3	1545	CYP1B1	5153	33.753	20.4022	0.72630	0.0031	0.0465955
NM_001136032.2	11012	KLK11	1181	2.2524	7.77663	-1.78768	0.00315	0.0471261
TCONS_00114649	XLOC_009051	-	3848	2.64177	1.02919	1.36000	0.00315	0.0471261
NM_014669.4	9688	NUP93	2922	16.1983	9.26515	0.80596	0.00315	0.0471261
NM_001173523.1	5099	PCDH7	8728	6.19838	13.4355	-1.11608	0.00315	0.0471261
NM_007030.2	11076	TPPP	6022	2.68081	5.18745	-0.95236	0.00315	0.0471261
NM_001039350.1	1804	DPP6	4705	1.16702	3.57046	-1.61328	0.00315	0.0471261
NM_145756.2	252884	ZNF396	2788	2.71131	5.44318	-1.00546	0.00315	0.0471261
TCONS_00204494	XLOC_018243	-	1205	0.616991	2.25311	-1.86860	0.00315	0.0471261
NM_001144874.1	399968	PATE4	1986	0.208086	0.865615	-2.05654	0.0032	0.0476939

NM_001004310.2	343413	FCRL6	1943	1.50774	3.94811	-1.38877	0.0032	0.0476939
ENST00000419628.1	ENSG00000225431.1	AP001626.1	1775	0.595341	1.71796	-1.52891	0.0032	0.0476939
NM_001113490.1	154796	AMOT	6945	6.57497	12.2304	-0.89541	0.0032	0.0476939
ENST00000417026.1	ENSG00000237513.1	RP11-325F22.2	891	2.83226	0.759017	1.89975	0.0032	0.0476939
NM_004932.3	1004	CDH6	8571	9.63535	5.7505	0.74465	0.0032	0.0476939
NM_006438.3	10584	COLEC10	1708	1.28931	0.375388	1.78014	0.0032	0.0476939
NM_022818.4	81631	MAP1LC3B	2288	43.2491	26.6058	0.70093	0.00325	0.0483534
NM_001100818.1	55022	PID1	2611	14.866	26.2013	-0.81762	0.0033	0.0489028
NM_001444.2	2171	FABP5	731	61.637	35.0997	0.81234	0.0033	0.0489028
NM_018665.2	55510	DDX43	2696	1.43748	0.453827	1.66333	0.0033	0.0489028
NM_138731.6	145282	MIPOL1	6120	3.37155	10.32	-1.61396	0.0033	0.0489028
NM_153827.4	50488	MINK1	5017	7.71705	3.36916	1.19566	0.0033	0.0489028
NM_000218.2	3784	KCNQ1	3246	4.40075	1.9526	1.17235	0.00335	0.0494369
NM_002941.3	6091	ROBO1	6839	10.1585	3.1091	1.70812	0.00335	0.0494369
NM_018425.2	55361	PI4K2A	4185	10.9131	6.3252	0.78687	0.00335	0.0494369
NM_001127361.1	127544	RNF19B	2603	10.481	3.25572	1.68673	0.00335	0.0494369
NM_001172624.1	4756	NEO1	7055	12.1324	20.2755	-0.74088	0.00335	0.0494369
NM_003525.2	8346	HIST1H2BI	437	55.8263	27.0655	1.04449	0.00335	0.0494369
NM_022763.3	64778	FNDC3B	7015	37.3943	17.7838	1.07226	0.00335	0.0494369
NM_001010979.1	388701	C1orf189	387	5.65013	18.2564	-1.69205	0.00335	0.0494369
NM_003462.3	7802	DNALI1	2649	11.5664	32.8806	-1.50730	0.00335	0.0494369
NM_004131.4	3002	GZMB	927	8.68444	3.68794	1.23562	0.00335	0.0494369
NM_018990.3	54440	SASH3	2694	13.8878	7.6961	0.85162	0.00335	0.0494369

Table S4. Differentially expressed lncRNA and mRNA transcripts for non-ECRSwNP vs CTRL.

Transcript ID	Gene ID	Gene name	Length	non-ECRSwNP FPKM	CTRL FPKM	log_2(foldchange)	P value	Q value
NM_022136.3	64092	SAMSN1	1888	34.2492	12.2431	1.48411	5.00E-05	0.00170056
NM_003248.4	7060	THBS4	3223	0.367177	10.474	-4.83419	5.00E-05	0.00170056
NM_001160130.1	56479	KCNQ5	6556	0.604168	1.97105	-1.70594	5.00E-05	0.00170056
NM_014685.2	9709	HERPUD1	2176	106.739	49.9015	1.09693	5.00E-05	0.00170056
NM_025074.6	80144	FRAS1	15624	1.70678	7.59287	-2.15336	5.00E-05	0.00170056
NM_003608.3	8477	GPR65	4522	9.4031	4.10697	1.19506	5.00E-05	0.00170056
NM_005097.2	9211	LGI1	2366	4.21462	0.473709	3.15333	5.00E-05	0.00170056
NM_001040272.5	92949	ADAMTSL1	7843	1.80434	5.358	-1.57022	5.00E-05	0.00170056
NM_003039.2	6518	SLC2A5	2438	3.48966	0.667059	2.3872	5.00E-05	0.00170056
NM_002381.4	4148	MATN3	2583	1.41571	7.7199	-2.44706	5.00E-05	0.00170056
NM_015187.3	23231	SEL1L3	4549	94.5213	44.7299	1.0794	5.00E-05	0.00170056
NM_000395.2	1439	CSF2RB	4848	12.4538	3.70911	1.74744	5.00E-05	0.00170056
XM_001716834.2	642131	LOC642131	570	81.0706	11.6645	2.79705	5.00E-05	0.00170056
NM_018286.2	55273	TMEM100	1755	20.2749	55.7012	-1.45801	5.00E-05	0.00170056
NM_001080437.1	25992	SNED1	6834	6.24351	13.205	-1.08065	5.00E-05	0.00170056
NM_001422.2	2001	ELF5	2302	8.69459	23.5601	-1.43815	5.00E-05	0.00170056
NM_023940.2	65997	RASL11B	1957	5.9534	19.4123	-1.70519	5.00E-05	0.00170056
ENST00000457157.2	ENSG00000231106.2	AP000688.8	1048	8.02273	27.6064	-1.78284	5.00E-05	0.00170056
NM_020980.3	366	AQP9	3021	5.48304	1.30761	2.06805	5.00E-05	0.00170056
NM_173814.4	283659	PRTG	11973	2.89744	5.65892	-0.96575	5.00E-05	0.00170056
NM_013358.2	29943	PADI1	3847	2.86203	0.842389	1.76448	5.00E-05	0.00170056
NM_001037339.1	5142	PDE4B	3876	13.6349	4.28492	1.66997	5.00E-05	0.00170056
NM_001136103.2	92293	TMEM132C	4947	0.626457	5.482	-3.12942	5.00E-05	0.00170056
NM_002997.4	6382	SDC1	3201	56.1283	26.6973	1.07203	5.00E-05	0.00170056
NM_021928.3	60559	SPCS3	4596	91.5712	42.2901	1.11458	5.00E-05	0.00170056
NM_001008781.2	120114	FAT3	19048	0.611261	5.86196	-3.26152	5.00E-05	0.00170056
TCONS_00081181	XLOC_006945	-	4801	2.92453	11.6812	-1.99791	5.00E-05	0.00170056
NM_001251.2	968	CD68	1856	45.5251	21.1771	1.10416	5.00E-05	0.00170056
NM_001010906.1	389643	C8orf80	3887	6.11576	2.11896	1.52918	5.00E-05	0.00170056
NM_000598.4	3486	IGFBP3	2613	365.724	71.5229	2.35428	5.00E-05	0.00170056
NM_001063.3	7018	TF	2791	44.1466	4.31891	3.35356	5.00E-05	0.00170056
NM_005980.2	6286	S100P	510	53.9113	5.77578	3.2225	5.00E-05	0.00170056
NM_014708.4	9735	KNTC1	6965	14.2564	7.32504	0.960702	5.00E-05	0.00170056
NM_004079.4	1520	CTSS	4107	127.782	58.434	1.12881	5.00E-05	0.00170056
NM_001036.3	6263	RYR3	15559	0.556124	2.2559	-2.02023	5.00E-05	0.00170056
NM_004004.5	2706	GJB2	2334	14.811	5.78519	1.35624	5.00E-05	0.00170056
NM_007361.3	22795	NID2	5046	5.57244	21.1646	-1.92527	5.00E-05	0.00170056
NM_002514.3	4856	NOV	2601	6.88939	34.4704	-2.32291	5.00E-05	0.00170056
NM_002163.2	3394	IRF8	2662	25.0222	9.67969	1.37017	5.00E-05	0.00170056
NM_033280.2	90701	SEC11C	782	211.442	70.3127	1.58841	5.00E-05	0.00170056
NM_198964.1	5744	PTHLH	1854	11.2623	0.810568	3.79642	5.00E-05	0.00170056
NM_021149.2	23406	COTL1	1827	38.2875	17.5866	1.1224	5.00E-05	0.00170056
NM_002118.4	3109	HLA-DMB	1397	93.8486	31.0085	1.59767	5.00E-05	0.00170056
NM_178565.4	340419	RSPO2	3149	2.6254	0.317752	3.04657	5.00E-05	0.00170056
NM_000101.2	1535	CYBA	688	235.814	76.725	1.61988	5.00E-05	0.00170056
NM_003897.3	8870	IER3	1240	27.5977	10.9513	1.33345	5.00E-05	0.00170056
NM_152598.2	162333	MARCH10	3101	3.47258	8.47365	-1.28697	5.00E-05	0.00170056
NM_178135.3	345275	HSD17B13	2395	1.33494	10.1553	-2.92738	5.00E-05	0.00170056
NM_002003.3	2219	FCN1	1283	30.9812	3.82727	3.01701	5.00E-05	0.00170056
NM_018699.2	11107	PRDM5	2480	8.92824	20.0417	-1.16656	5.00E-05	0.00170056
NM_001385.2	1807	DPYS	2123	2.31227	26.7461	-3.53195	5.00E-05	0.00170056

NM_052863.2	92304	SCGB3A1	466	8.45061	80.549	-3.25274	5.00E-05	0.00170056
TCONS_00143168	XLOC_012949	-	546	0	2.52567	#NAME?	5.00E-05	0.00170056
NM_145170.3	118491	TTC18	3704	20.0308	40.6025	-1.01935	5.00E-05	0.00170056
NM_005127.2	9976	CLEC2B	2038	33.8776	12.217	1.47144	5.00E-05	0.00170056
NM_000059.3	675	BRCA2	11386	5.66493	2.76446	1.03506	5.00E-05	0.00170056
NM_002117.4	3107	HLA-C	1525	386.143	154.551	1.32105	5.00E-05	0.00170056
NM_002964.4	6279	S100A8	523	93.4571	20.3068	2.20234	5.00E-05	0.00170056
NM_000702.3	477	ATP1A2	5464	8.24277	38.7615	-2.23342	5.00E-05	0.00170056
NM_031935.2	83872	HMCN1	18212	37.6918	11.1751	1.75397	5.00E-05	0.00170056
NM_003014.3	6424	SFRP4	2974	25.1784	153.406	-2.60709	5.00E-05	0.00170056
NM_001192.2	608	TNFRSF17	994	21.6875	7.18924	1.59295	5.00E-05	0.00170056
NM_022469.3	64388	GREM2	4183	8.6881	17.6887	-1.02571	5.00E-05	0.00170056
NM_001005738.1	2358	FPR2	1913	4.1006	0.818838	2.32418	5.00E-05	0.00170056
NM_021181.3	57823	SLAMF7	2657	50.9278	14.606	1.80189	5.00E-05	0.00170056
NM_020056.4	3118	HLA-DQA2	1152	82.7047	11.1448	2.89159	5.00E-05	0.00170056
NM_000491.3	713	C1QB	1044	159.757	69.6135	1.19844	5.00E-05	0.00170056
NM_001711.4	633	BGN	2444	15.6835	35.3612	-1.17292	5.00E-05	0.00170056
NM_153717.2	2121	EVC	6430	4.39357	8.61744	-0.971867	5.00E-05	0.00170056
NM_002970.2	6303	SAT1	1064	1018.91	294.105	1.79263	5.00E-05	0.00170056
NM_000065.2	729	C6	3606	5.57945	21.8203	-1.96747	5.00E-05	0.00170056
TCONS_00057873	XLOC_005084	-	5007	2.87482	8.61976	-1.58417	5.00E-05	0.00170056
NM_006646.5	10810	WASF3	4718	2.2989	5.59466	-1.28311	5.00E-05	0.00170056
NM_007153.3	7757	ZNF208	9088	1.8439	6.95137	-1.91454	5.00E-05	0.00170056
NM_006424.2	10568	SLC34A2	4153	12.6765	3.01516	2.07185	5.00E-05	0.00170056
NM_144616.3	126306	JSRP1	1145	6.16069	1.56697	1.97511	5.00E-05	0.00170056
NM_024560.2	79611	ACSS3	3033	7.52203	19.9558	-1.40761	5.00E-05	0.00170056
NM_000593.5	6890	TAP1	2952	51.1937	16.7384	1.6128	5.00E-05	0.00170056
NM_032777.9	25960	GPR124	6034	10.73	24.1094	-1.16795	5.00E-05	0.00170056
NM_002965.3	6280	S100A9	577	86.7818	11.6548	2.89646	5.00E-05	0.00170056
NM_001098484.2	8671	SLC4A4	7693	15.3342	6.76483	1.18063	5.00E-05	0.00170056
NM_012328.2	4189	DNAJB9	2538	59.0113	18.8914	1.64326	5.00E-05	0.00170056
NM_002127.5	3135	HLA-G	1578	4.88624	0.389339	3.64963	5.00E-05	0.00170056
NM_006280.2	6748	SSR4	725	253.8	78.1322	1.6997	5.00E-05	0.00170056
NM_003167.3	6822	SULT2A1	1987	0.519138	18.6869	-5.16977	5.00E-05	0.00170056
NM_022475.2	64399	HHIP	3555	3.08747	0.89534	1.78592	5.00E-05	0.00170056
NM_172369.3	714	C1QC	1168	65.5465	29.6141	1.14623	5.00E-05	0.00170056
NM_000655.4	6402	SELL	2442	22.236	7.72278	1.5257	5.00E-05	0.00170056
NM_024764.2	79820	CATSPERB	3606	3.31545	0.835004	1.98935	5.00E-05	0.00170056
NM_004967.3	3381	IBSP	1591	0.283272	22.5586	-6.31535	5.00E-05	0.00170056
NM_005213.3	1475	CSTA	828	39.4759	10.0048	1.98029	5.00E-05	0.00170056
TCONS_00000209	XLOC_000044	-	4800	0.466131	1.8116	-1.95846	5.00E-05	0.00170056
NM_006398.3	10537	UBD	990	152.781	20.3812	2.90616	5.00E-05	0.00170056
NM_138636.4	51311	TLR8	4216	9.59225	2.54637	1.91343	5.00E-05	0.00170056
NM_153183.2	170685	NUDT10	2001	13.0267	3.37292	1.9494	5.00E-05	0.00170056
NM_002432.1	4332	MNDA	1667	39.4322	13.1432	1.58506	5.00E-05	0.00170056
NM_001040058.1	6696	SPP1	1625	13.1633	102.889	-2.9665	5.00E-05	0.00170056
NM_013250.2	7762	ZNF215	3658	10.744	2.64557	2.02188	5.00E-05	0.00170056
NM_001010922.2	440603	BCL2L15	4973	6.0172	1.73169	1.79691	5.00E-05	0.00170056
NM_000217.2	3736	KCNA1	7983	0.525153	2.53179	-2.26935	5.00E-05	0.00170056
NM_001156474.1	60494	CCDC81	2781	3.25861	13.5553	-2.05652	5.00E-05	0.00170056
NM_001127380.2	6289	SAA2	2043	45.6897	2.2202	4.36311	5.00E-05	0.00170056
TCONS_00249239	XLOC_021575	-	5196	0.433597	2.92079	-2.75194	5.00E-05	0.00170056
NM_002229.2	3726	JUNB	1816	27.4683	12.9681	1.0828	5.00E-05	0.00170056
NM_001080427.1	80731	THSD7B	5846	0.298006	2.51427	-3.07673	5.00E-05	0.00170056

NM_006636.3	10797	MTHFD2	2188	22.7176	8.87822	1.35547	5.00E-05	0.00170056
NM_001944.2	1830	DSG3	5551	3.93888	1.05254	1.9039	5.00E-05	0.00170056
NM_015364.4	23643	LY96	622	42.3746	11.2909	1.90804	5.00E-05	0.00170056
NM_002460.3	3662	IRF4	5327	16.4181	3.36478	2.2867	5.00E-05	0.00170056
NM_002164.5	3620	IDO1	1931	102.462	7.64251	3.7449	5.00E-05	0.00170056
NM_144717.3	53833	IL20RB	2047	11.4139	3.4085	1.74358	5.00E-05	0.00170056
NM_001911.2	1511	CTSG	924	19.4127	6.01945	1.6893	5.00E-05	0.00170056
NM_024574.3	79625	NDNF	2871	2.32929	12.2793	-2.39826	5.00E-05	0.00170056
NM_000526.4	3861	KRT14	1636	9.3015	0.611884	3.92613	5.00E-05	0.00170056
NM_005566.3	3939	LDHA	2208	195.49	77.9652	1.32619	5.00E-05	0.00170056
NM_030926.4	81618	ITM2C	2073	57.9318	24.8347	1.222	5.00E-05	0.00170056
NM_007350.3	22822	PHLDA1	5913	14.7214	5.7379	1.35932	5.00E-05	0.00170056
NM_001554.4	3491	CYR61	2295	33.3372	13.7271	1.2801	5.00E-05	0.00170056
TCONS_00137738	XLOC_011479	-	1463	1.75389	5.56089	-1.66476	5.00E-05	0.00170056
NM_001122679.1	57451	ODZ2	9645	0.666911	1.98435	-1.5731	5.00E-05	0.00170056
NM_000112.3	1836	SLC26A2	8075	71.5924	22.1299	1.69381	5.00E-05	0.00170056
NM_004363.2	1048	CEACAM5	3600	12.0636	0.671976	4.16611	5.00E-05	0.00170056
NM_000441.1	5172	SLC26A4	4930	23.743	1.16855	4.34471	5.00E-05	0.00170056
NM_024817.2	79875	THSD4	9145	17.6737	46.0633	-1.38202	5.00E-05	0.00170056
NM_007199.2	11213	IRAK3	8344	25.5484	12.1384	1.07366	5.00E-05	0.00170056
NM_003955.3	9021	SOCS3	2734	15.2486	3.55188	2.10202	5.00E-05	0.00170056
NM_032505.2	84541	KBTBD8	4684	6.50356	3.00515	1.11379	5.00E-05	0.00170056
NM_002974.2	6318	SERPINB4	1719	94.5907	3.23896	4.86809	5.00E-05	0.00170056
TCONS_00084037	XLOC_006761	-	5365	1.77578	0.522978	1.76363	5.00E-05	0.00170056
NM_001135254.1	5081	PAX7	6053	2.94581	0.381149	2.95024	5.00E-05	0.00170056
NM_001098816.2	26011	ODZ4	13548	1.70121	4.08818	-1.26489	5.00E-05	0.00170056
TCONS_00087575	XLOC_007492	-	3315	0.228634	1.27903	-2.48393	5.00E-05	0.00170056
NM_004994.2	4318	MMP9	2336	4.45143	14.5385	-1.70753	5.00E-05	0.00170056
NM_001033602.2	23281	MTUS2	6939	0.311843	1.43098	-2.19812	5.00E-05	0.00170056
NM_002120.3	3112	HLA-DOB	1372	13.5204	4.28167	1.65889	5.00E-05	0.00170056
NM_001870.2	1359	CPA3	1686	74.1552	36.5306	1.02144	5.00E-05	0.00170056
NM_013381.2	29953	TRHDE	5552	0.875037	3.75703	-2.10218	5.00E-05	0.00170056
NM_152754.2	223117	SEMA3D	6261	29.9333	12.4977	1.26009	5.00E-05	0.00170056
NM_005564.3	3934	LCN2	822	603.048	99.7193	2.59633	5.00E-05	0.00170056
NM_020406.2	57126	CD177	2364	13.8532	1.36081	3.34768	5.00E-05	0.00170056
NM_182658.1	359710	C20orf185	1454	14.108	3.4163	2.04601	5.00E-05	0.00170056
NM_007231.3	11254	SLC6A14	4564	59.3198	29.2591	1.01963	5.00E-05	0.00170056
NM_000965.3	5915	RARB	3052	14.7021	28.7589	-0.96799	5.00E-05	0.00170056
NM_018950.2	3134	HLA-F	1284	91.3257	36.2195	1.33425	5.00E-05	0.00170056
NM_001218.3	771	CA12	3975	27.1506	3.83575	2.8234	5.00E-05	0.00170056
NM_014737.2	9770	RASSF2	5426	25.4755	12.0377	1.08155	5.00E-05	0.00170056
TCONS_00053981	XLOC_004304	-	6951	2.05471	0.590623	1.79863	5.00E-05	0.00170056
NM_003667.2	8549	LGR5	2880	0.522087	2.83296	-2.43995	5.00E-05	0.00170056
NM_014256.3	10331	B3GNT3	2720	6.00884	1.93042	1.63818	5.00E-05	0.00170056
NM_000478.4	249	ALPL	2596	3.94506	9.76129	-1.30702	5.00E-05	0.00170056
NM_003226.3	7033	TFF3	1054	64.5205	17.8191	1.85634	5.00E-05	0.00170056
TCONS_00202192	XLOC_017222	-	11403	3.37722	9.68556	-1.52	5.00E-05	0.00170056
NM_025195.2	10221	TRIB1	3633	19.9818	7.00342	1.51256	5.00E-05	0.00170056
NM_002994.3	6374	CXCL5	2458	3.02738	0.43758	2.79045	5.00E-05	0.00170056
NM_152997.2	260436	C4orf7	539	600.743	19.3682	4.95499	5.00E-05	0.00170056
NM_014080.4	50506	DUOX2	6346	3.22798	0.336585	3.26159	5.00E-05	0.00170056
NM_000720.2	776	CACNA1D	7768	2.20781	8.05376	-1.86704	5.00E-05	0.00170056
NM_000570.3	2215	FCGR3B	2295	12.5334	4.60318	1.44507	5.00E-05	0.00170056
TCONS_00160811	XLOC_013647	-	2061	4.6873	1.11116	2.0767	5.00E-05	0.00170056

TCONS_00267790	XLOC_022887	-	1986	0.426526	4.46003	-3.38635	5.00E-05	0.00170056
NM_000963.2	5743	PTGS2	4493	15.1943	2.13877	2.82868	5.00E-05	0.00170056
NM_002458.2	727897	MUC5B	17916	33.8068	7.31359	2.20866	5.00E-05	0.00170056
NM_005130.3	9982	FGFBP1	1182	4.68987	0.744421	2.65536	5.00E-05	0.00170056
NM_012309.3	22941	SHANK2	10495	1.83589	4.74797	-1.37083	5.00E-05	0.00170056
NM_012108.2	26228	STAP1	1511	14.2708	5.70392	1.32304	5.00E-05	0.00170056
NM_000922.3	5140	PDE3B	6091	9.26319	2.87868	1.6861	5.00E-05	0.00170056
NM_018004.1	55076	TMEM45A	1564	38.1525	4.70917	3.01823	5.00E-05	0.00170056
NM_003357.4	7356	SCGB1A1	452	20.9764	2998.95	-7.15955	5.00E-05	0.00170056
NM_000602.3	5054	SERPINE1	3181	4.26985	0.726664	2.55482	5.00E-05	0.00170056
NM_130853.2	5802	PTPRS	6006	5.94949	15.0599	-1.33988	5.00E-05	0.00170056
NM_002090.2	2921	CXCL3	1159	4.80877	0.313661	3.93839	5.00E-05	0.00170056
NM_000189.4	3099	HK2	7093	7.87652	2.62488	1.58531	5.00E-05	0.00170056
NM_000237.2	4023	LPL	3747	7.22783	2.70361	1.41868	5.00E-05	0.00170056
NM_016459.3	51237	MZB1	827	95.5045	25.3635	1.91282	5.00E-05	0.00170056
NM_030764.3	79368	FCRL2	2589	4.58836	0.984152	2.22102	5.00E-05	0.00170056
NM_173812.4	283417	DPY19L2	4057	1.55026	8.58391	-2.46912	5.00E-05	0.00170056
NM_000474.3	7291	TWIST1	1666	19.1523	42.654	-1.15516	5.00E-05	0.00170056
NM_014361.2	53942	CNTN5	4164	5.83789	0.592407	3.30078	5.00E-05	0.00170056
NM_005623.2	6355	CCL8	1250	6.99974	1.16458	2.5875	5.00E-05	0.00170056
NM_001001557.2	392255	GDF6	3701	0.214651	1.32606	-2.62708	5.00E-05	0.00170056
NM_002116.7	3105	HLA-A	1611	560.15	198.985	1.49315	5.00E-05	0.00170056
NM_020183.3	56938	ARNTL2	1930	9.51237	3.62337	1.39247	5.00E-05	0.00170056
NM_199161.3	6288	SAA1	531	405.123	22.2964	4.18348	5.00E-05	0.00170056
NM_030754.4	6289	SAA2	594	160.834	3.86528	5.37886	5.00E-05	0.00170056
NM_001184744.1	5745	PTH1R	1991	1.56498	6.44491	-2.04202	5.00E-05	0.00170056
NM_005532.3	3429	IFI27	652	171.702	65.0863	1.39948	5.00E-05	0.00170056
NM_015515.3	25984	KRT23	2147	48.4544	10.4588	2.21191	5.00E-05	0.00170056
TCONS_00087932	XLOC_007810	-	4484	0.471366	1.99556	-2.08187	5.00E-05	0.00170056
NM_007226.2	11249	NXPH2	2560	9.77365	1.81471	2.42916	5.00E-05	0.00170056
TCONS_00208641	XLOC_017561	-	1790	4.55605	1.14283	1.99517	5.00E-05	0.00170056
NM_024677.4	79730	NSUN7	3698	9.58667	21.2879	-1.15094	5.00E-05	0.00170056
NM_001783.3	973	CD79A	1258	62.0491	10.9739	2.49933	5.00E-05	0.00170056
NM_030761.4	54361	WNT4	3905	1.6174	6.4267	-1.9904	5.00E-05	0.00170056
NM_021237.3	58515	SELK	818	60.4969	26.596	1.18565	5.00E-05	0.00170056
NM_003263.3	7096	TLR1	2851	23.8167	12.0264	0.985766	5.00E-05	0.00170056
NM_021073.2	653	BMP5	2172	28.3511	9.13111	1.63454	5.00E-05	0.00170056
NM_002133.2	3162	HMOX1	1590	11.0675	4.10273	1.43167	5.00E-05	0.00170056
NM_004484.3	2719	GPC3	2319	2.08326	7.05285	-1.75936	5.00E-05	0.00170056
TCONS_00075737	XLOC_006473	-	4288	1.71035	5.77918	-1.75657	5.00E-05	0.00170056
NM_025220.2	80332	ADAM33	3519	4.10709	10.1957	-1.31178	5.00E-05	0.00170056
NM_007191.4	11197	WIF1	2240	0.430398	3.76249	-3.12794	5.00E-05	0.00170056
NM_000669.3	126	ADH1C	1473	52.9387	106.534	-1.00893	5.00E-05	0.00170056
NM_006039.3	9902	MRC2	5983	13.9776	31.3635	-1.16597	5.00E-05	0.00170056
NM_003116.1	6676	SPAG4	1452	5.87019	1.32174	2.15097	5.00E-05	0.00170056
NM_004227.3	9265	CYTH3	4469	11.8152	23.9407	-1.01882	5.00E-05	0.00170056
NM_018456.4	55840	EAF2	1020	35.4981	13.1116	1.4369	5.00E-05	0.00170056
NM_006207.2	5157	PDGFRL	1905	5.04637	12.6782	-1.32904	5.00E-05	0.00170056
NM_006103.3	10406	WFDC2	566	395.577	93.0004	2.08865	5.00E-05	0.00170056
NM_001085384.1	7710	ZNF154	2747	3.6318	10.2001	-1.48982	5.00E-05	0.00170056
NM_020801.2	57561	ARRDC3	4161	105.071	54.3784	0.950254	5.00E-05	0.00170056
NM_006486.2	2192	FBLN1	2947	47.6831	122.745	-1.36412	5.00E-05	0.00170056
NM_000093.3	1289	COL5A1	8439	9.28306	19.7935	-1.09235	5.00E-05	0.00170056
NM_006274.2	6363	CCL19	684	26.5221	7.33818	1.8537	5.00E-05	0.00170056

NM_002427.3	4322	MMP13	2719	0.879573	159.113	-7.49903	5.00E-05	0.00170056
NM_001565.3	3627	CXCL10	1216	82.0924	16.4535	2.31886	5.00E-05	0.00170056
NM_153490.2	3860	KRT13	1715	6.46307	0.675297	3.25863	5.00E-05	0.00170056
NM_005764.3	10158	PDZK1IP1	894	11.4724	1.55138	2.88655	5.00E-05	0.00170056
TCONS_00233089	XLOC_020133	-	2857	0.841336	3.05482	-1.86033	5.00E-05	0.00170056
NM_033119.4	85407	NKD1	8716	3.15174	8.38439	-1.41156	5.00E-05	0.00170056
NM_144575.2	92291	CAPN13	2682	85.7017	35.4882	1.27199	5.00E-05	0.00170056
NM_001124.1	133	ADM	1449	10.1365	2.81998	1.8458	5.00E-05	0.00170056
NM_197955.1	84419	C15orf48	803	26.0488	1.50857	4.10997	5.00E-05	0.00170056
NM_032532.2	84624	FNDC1	6551	1.0843	4.36865	-2.01042	5.00E-05	0.00170056
NM_130830.4	131578	LRRC15	5883	0.527111	4.55081	-3.10995	5.00E-05	0.00170056
NM_133459.3	147372	CCBE1	6260	0.319426	3.31121	-3.37381	5.00E-05	0.00170056
NM_006406.1	10549	PRDX4	921	86.3149	34.3174	1.33067	5.00E-05	0.00170056
NM_001010919.1	441168	FAM26F	1109	23.2319	8.11979	1.5166	5.00E-05	0.00170056
TCONS_00053726	XLOC_003947	-	4699	2.40073	7.38542	-1.62121	5.00E-05	0.00170056
NM_006235.2	5450	POU2AF1	3032	9.3752	3.01664	1.6359	5.00E-05	0.00170056
NM_005554.3	3853	KRT6A	2450	12.5934	1.69885	2.89004	5.00E-05	0.00170056
NM_000063.4	717	C2	2838	21.2954	7.39082	1.52673	5.00E-05	0.00170056
NM_173701.1	7453	WARS	2660	71.0735	22.9934	1.62809	5.00E-05	0.00170056
NM_002993.3	6372	CXCL6	1659	16.9881	4.18377	2.02165	5.00E-05	0.00170056
NM_000343.3	6523	SLC5A1	5061	4.11193	0.800905	2.36011	5.00E-05	0.00170056
NM_015907.2	51056	LAP3	2100	135.428	55.0969	1.29749	5.00E-05	0.00170056
NM_014479.3	27299	ADAMDEC1	2348	32.8639	3.18629	3.36655	5.00E-05	0.00170056
NM_145793.3	2674	GFRA1	9134	7.77643	27.8384	-1.8399	5.00E-05	0.00170056
NM_000313.3	5627	PROS1	3580	20.8197	60.5745	-1.54076	5.00E-05	0.00170056
NM_005651.2	6999	TDO2	1693	3.39385	0.56747	2.58031	5.00E-05	0.00170056
NM_002704.3	5473	PPBP	1307	0.9285	5.13818	-2.46828	5.00E-05	0.00170056
NM_005218.3	1672	DEFB1	463	46.4878	7.04696	2.72178	5.00E-05	0.00170056
NM_001007156.1	4916	NTRK3	3982	1.87484	6.62195	-1.82049	5.00E-05	0.00170056
NM_020974.2	57758	SCUBE2	4536	1.92054	6.48128	-1.75476	5.00E-05	0.00170056
NM_001002235.2	5265	SERPINA1	3199	17.1309	4.31088	1.99055	5.00E-05	0.00170056
NM_032119.3	84059	GPR98	19333	4.91004	18.2589	-1.89479	5.00E-05	0.00170056
NM_001977.3	2028	ENPEP	4995	13.6196	27.588	-1.01836	5.00E-05	0.00170056
NM_001002029.3	721	C4B	5427	3.28823	1.1247	1.54777	5.00E-05	0.00170056
NM_001814.4	1075	CTSC	1907	147.917	63.5718	1.21833	5.00E-05	0.00170056
NM_001039.3	6340	SCNN1G	3499	8.62819	1.69514	2.34766	5.00E-05	0.00170056
NM_002029.3	2357	FPR1	1317	17.3148	2.93228	2.56191	5.00E-05	0.00170056
NM_004655.3	8313	AXIN2	4234	7.79652	20.4522	-1.39135	5.00E-05	0.00170056
NM_001145026.1	374462	PTPRQ	7612	0.614365	2.31692	-1.91504	5.00E-05	0.00170056
NM_006573.4	10673	TNFSF13B	2671	14.1867	4.04337	1.8109	5.00E-05	0.00170056
NM_153362.2	167681	PRSS35	2503	1.60902	5.56998	-1.79149	5.00E-05	0.00170056
NM_001623.3	199	AIF1	639	89.5452	32.3159	1.47037	5.00E-05	0.00170056
NM_020872.1	5067	CNTN3	4997	1.99485	7.85864	-1.978	5.00E-05	0.00170056
NM_003264.3	7097	TLR2	3403	23.0616	10.5935	1.12231	5.00E-05	0.00170056
NM_001195811.1	200150	PLD5	3018	0.576669	7.00164	-3.60188	5.00E-05	0.00170056
NM_148919.3	5696	PSMB8	1116	91.0255	40.1837	1.17966	5.00E-05	0.00170056
NM_001884.3	1404	HAPLN1	4678	0.565044	6.04425	-3.41913	5.00E-05	0.00170056
NM_025153.2	23120	ATP10B	7566	10.5547	0.633624	4.05811	5.00E-05	0.00170056
TCONS_00154706	XLOC_014975	-	16334	2.58297	6.16537	-1.25516	5.00E-05	0.00170056
NM_001080534.1	440279	UNC13C	8140	1.09559	4.96343	-2.17963	5.00E-05	0.00170056
NM_022073.3	112399	EGLN3	2722	40.2903	6.64	2.60118	5.00E-05	0.00170056
NM_002922.3	5996	RGS1	1403	67.9109	19.2747	1.81694	5.00E-05	0.00170056
NM_004675.2	9077	DIRAS3	1631	10.8335	2.58486	2.06734	5.00E-05	0.00170056
NM_174975.4	266629	SEC14L3	2084	1.07783	54.4952	-5.65992	5.00E-05	0.00170056

NM_006183.4	4922	NTS	1239	0.853768	20.3172	-4.57272	5.00E-05	0.00170056
NM_004065.2	1038	CDR1	1299	3.38912	40.7513	-3.58786	5.00E-05	0.00170056
NM_001105248.1	79838	TMC5	4917	72.1613	33.7987	1.09426	5.00E-05	0.00170056
NM_005978.3	6273	S100A2	963	90.9409	23.2854	1.9655	5.00E-05	0.00170056
NM_024329.5	79180	EFHD2	2419	25.4191	12.1345	1.0668	5.00E-05	0.00170056
NM_005114.2	9957	HS3ST1	1965	23.8248	6.63289	1.84476	5.00E-05	0.00170056
28526	28526	-	1510	7.67714	2.50311	1.61685	5.00E-05	0.00170056
3494	3494	-	1019	766.09	93.4102	3.03586	5.00E-05	0.00170056
NM_002800.4	5698	PSMB9	1048	63.8106	25.5587	1.31998	5.00E-05	0.00170056
NM_002423.3	4316	MMP7	1119	110.857	9.53539	3.53927	5.00E-05	0.00170056
NM_005763.3	10157	AASS	5871	21.0205	40.6068	-0.949921	5.00E-05	0.00170056
NM_198285.2	349136	WDR86	2041	6.01338	25.095	-2.06115	5.00E-05	0.00170056
NM_024721.4	79776	ZFHX4	13958	3.57141	9.54205	-1.4178	5.00E-05	0.00170056
NM_021127.2	5366	PMAIP1	1936	9.03686	3.30606	1.45071	5.00E-05	0.00170056
NM_001447.2	2196	FAT2	14536	8.4315	16.6754	-0.98386	5.00E-05	0.00170056
NM_024422.3	1824	DSC2	5199	6.96374	2.49327	1.48182	5.00E-05	0.00170056
NM_012301.3	9863	MAGI2	6880	3.06891	6.31978	-1.04215	5.00E-05	0.00170056
TCONS_00293562	XLOC_025465	-	11650	0.845853	3.32453	-1.97467	5.00E-05	0.00170056
NM_173354.3	150094	SIK1	4706	14.1567	4.40303	1.68492	5.00E-05	0.00170056
NM_001099677.1	57604	KIAA1456	9300	3.90013	9.92966	-1.34822	5.00E-05	0.00170056
NM_001101312.1	28959	TMEM176B	1298	51.8125	19.574	1.40436	5.00E-05	0.00170056
NM_006269.1	6101	RP1	7100	8.64943	18.6022	-1.1048	5.00E-05	0.00170056
NM_001558.3	3587	IL10RA	3656	23.2529	8.54456	1.44433	5.00E-05	0.00170056
NM_005252.3	2353	FOS	2158	44.4	7.21553	2.62138	5.00E-05	0.00170056
NM_003026.2	6456	SH3GL2	2728	0.176579	1.60812	-3.18699	5.00E-05	0.00170056
NM_000576.2	3553	IL1B	1498	3.9513	0.462931	3.09346	5.00E-05	0.00170056
NM_030810.3	81567	TXNDC5	3231	328.051	108.516	1.59601	5.00E-05	0.00170056
NM_002457.2	4583	MUC2	8623	1.72799	0.208277	3.05252	5.00E-05	0.00170056
NM_000766.3	1553	CYP2A13	1747	4.96824	19.9971	-2.00899	5.00E-05	0.00170056
NM_015419.3	25878	MXRA5	9795	18.1292	60.3428	-1.73487	5.00E-05	0.00170056
NM_006875.3	11040	PIM2	2187	52.7422	7.95323	2.72935	5.00E-05	0.00170056
NM_153221.2	148113	CILP2	4199	0.229136	2.31852	-3.33893	5.00E-05	0.00170056
NM_021103.3	9168	TMSB10	482	1339.84	601.074	1.15645	5.00E-05	0.00170056
NM_020848.2	57608	KIAA1462	9300	6.01114	21.0118	-1.80549	5.00E-05	0.00170056
NM_001085.4	12	SERPINA3	1590	89.048	14.9471	2.57471	5.00E-05	0.00170056
NM_003013.2	6423	SFRP2	1990	214.651	83.2395	1.36665	5.00E-05	0.00170056
NM_019105.6	7148	TNXB	13125	0.876743	2.48924	-1.50548	5.00E-05	0.00170056
NM_024893.1	79953	SYNDIG1	2437	2.4692	0.447981	2.46254	5.00E-05	0.00170056
NM_002089.3	2920	CXCL2	1205	6.53973	1.4232	2.20009	5.00E-05	0.00170056
NM_000949.5	5618	PRLR	11817	3.93311	15.3758	-1.96692	5.00E-05	0.00170056
NM_024592.4	79644	SRD5A3	4082	10.7479	4.77954	1.16911	5.00E-05	0.00170056
NM_014584.1	30001	ERO1L	3310	62.8114	28.498	1.14016	5.00E-05	0.00170056
NM_001442.2	2167	FABP4	838	3.9004	34.3176	-3.13725	5.00E-05	0.00170056
NM_001974.3	2015	EMR1	3123	1.82071	0.317868	2.518	5.00E-05	0.00170056
NM_013231.4	23768	FLRT2	7179	10.6433	25.2854	-1.24836	5.00E-05	0.00170056
NM_006010.4	7873	MANF	939	68.3469	30.5012	1.16401	5.00E-05	0.00170056
NM_021978.3	6768	ST14	3300	23.342	12.0487	0.954054	5.00E-05	0.00170056
TCONS_00062536	XLOC_005694	-	16273	6.33767	2.13204	1.57171	5.00E-05	0.00170056
NM_138426.2	113263	GLCCI1	4745	17.5286	7.93569	1.14328	5.00E-05	0.00170056
NM_005065.4	6400	SEL1L	6579	100.92	40.679	1.31086	5.00E-05	0.00170056
NM_012128.3	22802	CLCA4	3213	9.70005	0.856218	3.50194	5.00E-05	0.00170056
NM_080283.3	10350	ABCA9	6301	4.62366	10.4484	-1.17618	5.00E-05	0.00170056
NM_002426.4	4321	MMP12	1876	8.25199	0.966055	3.09456	5.00E-05	0.00170056
NM_031457.1	83661	MS4A8B	1353	37.2902	72.4867	-0.95892	5.00E-05	0.00170056

NM_000573.3	1378	CR1	8616	6.72801	1.75544	1.93835	5.00E-05	0.00170056
NM_003469.4	7857	SCG2	2572	83.0054	20.8374	1.99403	5.00E-05	0.00170056
NM_000379.3	7498	XDH	5717	6.25099	1.36851	2.19148	5.00E-05	0.00170056
NM_001012502.2	286207	C9orf117	1722	11.108	28.102	-1.33907	5.00E-05	0.00170056
NM_000574.3	1604	CD55	2796	94.4863	21.4055	2.14212	5.00E-05	0.00170056
NM_000782.4	1591	CYP24A1	3266	17.0522	2.81343	2.59956	5.00E-05	0.00170056
NM_001178126.1	100423062	IGLL5	1059	689.764	142.043	2.27977	5.00E-05	0.00170056
NM_014302.3	23480	SEC61G	466	180.896	77.2675	1.22723	5.00E-05	0.00170056
NM_006948.4	6782	HSPA13	3987	44.7465	19.5935	1.1914	5.00E-05	0.00170056
NM_178527.3	284525	SLC9A11	4427	1.10057	3.02734	-1.45979	5.00E-05	0.00170056
NM_004744.3	9227	LRAT	4888	0.406415	7.35898	-4.17848	5.00E-05	0.00170056
NM_000694.2	221	ALDH3B1	2815	13.9328	37.5336	-1.4297	5.00E-05	0.00170056
NM_001039350.1	1804	DPP6	4705	0.414929	3.57046	-3.10517	5.00E-05	0.00170056
NM_001105556.1	9473	C1orf38	2723	13.0308	4.60949	1.49925	5.00E-05	0.00170056
XM_002343299.3	319089	TTC6	2913	6.07558	14.5005	-1.25501	5.00E-05	0.00170056
NM_005555.3	3854	KRT6B	2282	3.19854	0.567703	2.49421	5.00E-05	0.00170056
NM_001848.2	1291	COL6A1	4225	23.3507	49.3671	-1.08008	5.00E-05	0.00170056
NM_007028.3	11074	TRIM31	2027	2.91783	0.561369	2.37788	5.00E-05	0.00170056
NM_014622.4	4013	VWA5A	3422	35.852	16.2463	1.14194	5.00E-05	0.00170056
NM_138433.3	113730	KLHDC7B	2991	3.75932	0.786473	2.257	5.00E-05	0.00170056
NM_019050.2	54532	USP53	6595	24.8874	91.3625	-1.87618	5.00E-05	0.00170056
NM_014331.3	23657	SLC7A11	9648	2.45937	0.862837	1.51113	5.00E-05	0.00170056
NM_001321.1	1466	CSRP2	901	107.577	290.382	-1.43258	5.00E-05	0.00170056
NM_198461.3	164832	LONRF2	13912	2.31864	4.91353	-1.08348	5.00E-05	0.00170056
NM_017551.2	2894	GRID1	5849	1.07676	3.10595	-1.52834	5.00E-05	0.00170056
NM_020130.3	56892	C8orf4	1833	69.3577	15.0847	2.20097	5.00E-05	0.00170056
NM_020802.2	57562	KIAA1377	7043	9.97199	20.8934	-1.0671	5.00E-05	0.00170056
NM_002272.3	3851	KRT4	2147	5.1576	19.585	-1.92498	5.00E-05	0.00170056
NM_000129.3	2162	F13A1	3863	145.465	50.3977	1.52924	5.00E-05	0.00170056
NM_002416.1	4283	CXCL9	2545	59.1654	12.9622	2.19044	5.00E-05	0.00170056
NM_002053.2	2633	GBP1	3050	107.536	41.1968	1.38421	5.00E-05	0.00170056
NM_002275.3	3866	KRT15	1840	84.4943	40.1227	1.07443	5.00E-05	0.00170056
NM_031311.3	54504	CPVL	1691	70.0336	29.4203	1.25124	5.00E-05	0.00170056
NM_003793.3	8722	CTSF	2014	16.1762	34.152	-1.0781	5.00E-05	0.00170056
NM_001099294.1	85352	KIAA1644	6741	0.920432	3.00503	-1.707	5.00E-05	0.00170056
NM_052947.3	115701	ALPK2	7303	6.02987	0.905442	2.73543	5.00E-05	0.00170056
NM_022068.2	63895	FAM38B	9713	2.73723	6.50433	-1.24868	5.00E-05	0.00170056
NM_033255.2	94240	EPSTI1	1508	55.2258	25.393	1.12091	5.00E-05	0.00170056
NM_145016.3	219970	GLYATL2	1380	10.1025	0.700837	3.84949	5.00E-05	0.00170056
3539	3539	-	318	996.829	166.569	2.58122	5.00E-05	0.00170056
3538	3538	-	320	1653.31	391.077	2.07984	5.00E-05	0.00170056
NM_004106.1	2207	FCER1G	591	51.6052	20.4269	1.33705	5.00E-05	0.00170056
NM_004751.2	9245	GCNT3	2222	5.7908	1.53688	1.91376	5.00E-05	0.00170056
NM_000442.4	5175	PECAM1	4449	25.6531	13.0084	0.979693	5.00E-05	0.00170056
NM_001102608.1	131873	COL6A6	8470	0.765362	2.25225	-1.55715	5.00E-05	0.00170056
NM_002664.2	5341	PLEK	2852	31.5762	12.9319	1.2879	5.00E-05	0.00170056
NM_000775.2	1573	CYP2J2	1857	10.2191	25.523	-1.32053	5.00E-05	0.00170056
NM_032977.3	843	CASP10	5906	9.55204	4.33585	1.13949	5.00E-05	0.00170056
NM_006674.3	10866	HCP5	2539	45.6173	21.5056	1.08487	5.00E-05	0.00170056
NM_002923.3	5997	RGS2	1350	49.8778	16.1875	1.62351	5.00E-05	0.00170056
NM_213606.3	387700	SLC16A12	4622	3.4552	19.0353	-2.46183	5.00E-05	0.00170056
NM_003637.3	8515	ITGA10	5170	0.969669	4.08453	-2.0746	5.00E-05	0.00170056
NM_006743.4	5935	RBM3	4432	68.2714	32.7683	1.05898	5.00E-05	0.00170056
NM_175610.2	7082	TJP1	6925	13.7364	54.4488	-1.98689	5.00E-05	0.00170056

NM_170744.3	219699	UNC5B	4998	3.42136	8.21604	-1.26387	5.00E-05	0.00170056
NM_000636.2	6648	SOD2	1593	184.076	75.8287	1.27949	5.00E-05	0.00170056
NM_001372.3	1770	DNAH9	13750	11.576	24.7797	-1.09802	5.00E-05	0.00170056
NM_016523.1	51348	KLRF1	1242	5.05444	0.967638	2.38501	5.00E-05	0.00170056
NM_001142966.1	80000	GREB1L	6052	0.909657	5.39672	-2.56869	5.00E-05	0.00170056
NM_130386.2	81035	COLEC12	3134	28.7509	57.4232	-0.998026	5.00E-05	0.00170056
NM_032446.2	84466	MEGF10	7689	0.55008	2.26397	-2.04114	5.00E-05	0.00170056
NM_004683.4	9104	RGN	1615	1.84038	9.81361	-2.41478	5.00E-05	0.00170056
NM_001127595.1	2214	FCGR3A	2155	65.3784	17.2765	1.92	5.00E-05	0.00170056
NM_152860.1	121340	SP7	2974	0.382827	2.13551	-2.47982	5.00E-05	0.00170056
NM_020546.2	108	ADCY2	6553	4.16552	9.7531	-1.22736	5.00E-05	0.00170056
NM_002578.3	5063	PAK3	2754	1.90389	6.02646	-1.66236	5.00E-05	0.00170056
NM_004120.3	2634	GBP2	2595	102.505	48.1214	1.09094	5.00E-05	0.00170056
NM_001128148.1	7037	TFRC	5083	43.4388	15.7885	1.46011	5.00E-05	0.00170056
NM_007072.2	11148	HHLA2	2660	1.35291	7.80557	-2.52844	5.00E-05	0.00170056
NM_006332.3	10437	IFI30	1032	104.067	25.0932	2.05214	5.00E-05	0.00170056
NM_002986.2	6356	CCL11	925	10.2261	2.42561	2.07584	5.00E-05	0.00170056
NM_003012.4	6422	SFRP1	4467	71.5409	197.444	-1.4646	5.00E-05	0.00170056
NM_002201.4	3669	ISG20	956	34.3377	11.7002	1.55326	5.00E-05	0.00170056
NM_000849.4	2947	GSTM3	4127	7.44923	17.5574	-1.23691	5.00E-05	0.00170056
NM_017709.3	54855	FAM46C	5720	73.8697	27.8846	1.40551	5.00E-05	0.00170056
NM_004787.1	9353	SLIT2	4950	6.46368	55.0618	-3.09062	5.00E-05	0.00170056
NM_002162.3	3385	ICAM3	1780	18.9177	7.05418	1.42319	5.00E-05	0.00170056
NM_000047.2	415	ARSE	2220	1.17565	3.49228	-1.57071	5.00E-05	0.00170056
NM_003104.5	6652	SORD	2813	40.6416	18.3127	1.15011	5.00E-05	0.00170056
NM_001097638.2	2524	FUT2	3089	18.942	8.33853	1.18373	5.00E-05	0.00170056
NM_001140.3	246	ALOX15	2684	62.3893	133.916	-1.10196	5.00E-05	0.00170056
NM_002119.3	3111	HLA-DOA	3485	38.7255	12.2806	1.6569	5.00E-05	0.00170056
3501	3501	-	980	753.4	85.1202	3.14584	5.00E-05	0.00170056
3502	3502	-	1348	1054.44	298.513	1.82061	5.00E-05	0.00170056
3503	3503	-	983	173.058	21.8126	2.98802	5.00E-05	0.00170056
3507	3507	-	1486	322.04	61.5317	2.38784	5.00E-05	0.00170056
NM_003383.3	7436	VLDLR	3636	1.9108	13.4397	-2.81425	5.00E-05	0.00170056
NM_001033047.2	255743	NPNT	4592	5.20316	21.7058	-2.06062	5.00E-05	0.00170056
NM_015991.2	712	C1QA	1098	144.189	69.9382	1.04381	5.00E-05	0.00170056
NM_016235.1	51704	GPRC5B	2845	24.6947	50.93	-1.04432	5.00E-05	0.00170056
NM_002334.3	4038	LRP4	8227	1.47768	6.31228	-2.09483	5.00E-05	0.00170056
NM_000100.2	1476	CSTB	658	122.62	53.7932	1.1887	5.00E-05	0.00170056
NM_007115.3	7130	TNFAIP6	1424	8.50815	1.43809	2.56469	5.00E-05	0.00170056
NM_018557.2	53353	LRP1B	16531	5.0119	31.27	-2.64135	5.00E-05	0.00170056
NM_000584.3	3576	IL8	1705	34.9818	4.72844	2.88717	5.00E-05	0.00170056
NM_002178.2	3489	IGFBP6	966	26.0181	77.1555	-1.56825	5.00E-05	0.00170056
NM_006798.2	10941	UGT2A1	2602	19.8036	119.231	-2.58993	5.00E-05	0.00170056
NM_004710.3	9144	SYNGR2	1694	28.3693	13.3931	1.08284	5.00E-05	0.00170056
NM_005322.2	3009	HIST1H1B	790	55.6771	20.4327	1.4462	5.00E-05	0.00170056
NM_006073.2	10345	TRDN	4475	0.120289	1.8518	-3.94435	5.00E-05	0.00170056
NM_004115.3	2259	FGF14	2720	7.83595	17.1291	-1.12827	5.00E-05	0.00170056
NM_006988.3	9510	ADAMTS1	4651	46.9244	11.7692	1.99532	5.00E-05	0.00170056
NM_000494.3	1308	COL17A1	5610	6.93792	1.50136	2.20823	5.00E-05	0.00170056
NM_001242.4	939	CD27	1320	17.3508	5.43311	1.67515	5.00E-05	0.00170056
NM_054114.3	117289	TAGAP	3363	17.5662	7.31083	1.2647	5.00E-05	0.00170056
NM_002725.3	5549	PRELP	5820	1.29927	4.20122	-1.69311	5.00E-05	0.00170056
NM_207517.2	57188	ADAMTSL3	7336	2.21176	5.16367	-1.2232	5.00E-05	0.00170056
NM_003695.2	8581	LY6D	802	8.65824	0.616338	3.81228	5.00E-05	0.00170056

NM_004131.4	3002	GZMB	927	15.4069	3.68794	2.06269	5.00E-05	0.00170056
NM_173833.5	286133	SCARA5	4151	4.76469	11.485	-1.26929	5.00E-05	0.00170056
NM_147189.2	90362	FAM110B	4368	1.78971	5.03693	-1.49282	5.00E-05	0.00170056
NM_001710.5	629	CFB	2627	173.38	34.0062	2.35007	5.00E-05	0.00170056
TCONS_00023764	XLOC_000192	-	17064	3.03785	10.3915	-1.77428	5.00E-05	0.00170056
NM_025208.4	80310	PDGFD	3993	14.3447	50.6722	-1.82067	5.00E-05	0.00170056
NM_000518.4	3043	HBB	626	77.3997	324.147	-2.06625	5.00E-05	0.00170056
NM_002010.2	2254	FGF9	4530	1.26164	3.95586	-1.64869	5.00E-05	0.00170056
TCONS_00283549	XLOC_023644	-	4674	1.51698	4.41261	-1.54043	5.00E-05	0.00170056
NM_020646.1	56676	ASCL3	650	11.9154	2.86395	2.05675	5.00E-05	0.00170056
NM_005233.5	2042	EPHA3	5809	1.05007	6.61111	-2.65441	5.00E-05	0.00170056
NM_173054.2	5649	RELN	11565	0.083582	2.0522	-4.61784	5.00E-05	0.00170056
NM_003890.2	8857	FCGBP	16390	2.30969	0.968251	1.25425	5.00E-05	0.00170056
TCONS_00065541	XLOC_005937	-	424	0	1.39769	#NAME?	5.00E-05	0.00170056
NM_000450.2	6401	SELE	3875	3.73904	1.0889	1.7798	5.00E-05	0.00170056
NM_002126.4	3131	HLF	5599	12.6699	26.3107	-1.05425	5.00E-05	0.00170056
NM_080826.1	140862	ISM1	2592	0.451919	3.53493	-2.96754	5.00E-05	0.00170056
NM_014675.3	9696	CROCC	6656	5.33402	11.6895	-1.13191	5.00E-05	0.00170056
TCONS_00049984	XLOC_004304	-	3774	3.43389	0.991739	1.79181	5.00E-05	0.00170056
NM_198505.2	344905	ATP13A5	3657	1.33878	0.202547	2.72459	5.00E-05	0.00170056
NM_000397.3	1536	CYBB	4318	96.4217	32.2032	1.58215	5.00E-05	0.00170056
NM_001008539.3	6542	SLC7A2	7571	27.3407	81.1648	-1.5698	5.00E-05	0.00170056
NM_001511.2	2919	CXCL1	1109	88.6328	26.2248	1.75691	5.00E-05	0.00170056
NM_006914.3	6096	RORB	3604	4.13765	11.2635	-1.44478	5.00E-05	0.00170056
NM_080617.4	140689	CBLN4	2508	0.302009	4.46969	-3.88751	5.00E-05	0.00170056
NM_080607.2	128434	VSTM2L	1966	3.29723	9.42846	-1.51577	5.00E-05	0.00170056
NM_030666.2	1992	SERPINB1	1293	90.8528	45.2849	1.0045	5.00E-05	0.00170056
NM_033049.3	56667	MUC13	2876	13.5798	4.95522	1.45444	5.00E-05	0.00170056
NM_052942.3	115362	GBP5	4052	63.3245	13.9567	2.18181	5.00E-05	0.00170056
NM_004419.3	1847	DUSP5	2528	9.9697	3.02362	1.72127	5.00E-05	0.00170056
NM_020927.1	57687	VAT1L	3766	0.855003	2.96006	-1.79163	5.00E-05	0.00170056
NM_005746.2	10135	NAMPT	4582	78.8086	33.1088	1.25114	5.00E-05	0.00170056
TCONS_00202813	XLOC_018025	-	11391	0.549591	2.21341	-2.00984	5.00E-05	0.00170056
TCONS_00220788	XLOC_018529	-	12307	1.23444	9.25856	-2.90693	5.00E-05	0.00170056
NM_003463.3	7803	PTP4A1	5082	35.7465	18.4763	0.952128	5.00E-05	0.00170056
NM_021572.4	59084	ENPP5	2943	22.3109	46.0303	-1.04483	5.00E-05	0.00170056
NM_001198.3	639	PRDM1	5165	24.887	7.43687	1.74262	5.00E-05	0.00170056
NM_016240.2	51435	SCARA3	3631	8.51071	19.6595	-1.20787	5.00E-05	0.00170056
NM_006120.3	3108	HLA-DMA	1122	105.769	38.8935	1.44331	5.00E-05	0.00170056
NM_001135091.1	143662	MUC15	3392	14.0165	27.42	-0.968098	0.0001	0.00312189
NM_006933.4	6526	SLC5A3	11576	61.8618	28.0502	1.14104	0.0001	0.00312189
TCONS_00003861	XLOC_000716	-	2713	1.22155	4.33183	-1.82627	0.0001	0.00312189
NM_021199.2	58472	SQRDL	1672	47.732	23.4867	1.02311	0.0001	0.00312189
NM_004770.2	9312	KCNB2	3582	2.1343	0.589069	1.85726	0.0001	0.00312189
TCONS_00201765	XLOC_018236	-	8017	2.22685	4.85068	-1.12318	0.0001	0.00312189
NM_002727.2	5552	SRGN	1254	222.297	112.672	0.980361	0.0001	0.00312189
NM_015931.1	51066	C3orf32	1545	0.50087	2.79847	-2.48213	0.0001	0.00312189
NM_014674.2	9695	EDEM1	6153	23.8921	12.1409	0.976661	0.0001	0.00312189
NM_207365.3	344752	AADACL2	1500	2.54804	7.16366	-1.49131	0.0001	0.00312189
NM_005215.3	1630	DCC	10210	2.03395	0.701912	1.53492	0.0001	0.00312189
NM_001118890.1	2745	GLRX	1079	32.0612	13.2694	1.27273	0.0001	0.00312189
NM_207437.3	196385	DNAH10	13680	11.213	23.8282	-1.08749	0.0001	0.00312189
NM_024641.3	79694	MANEA	4616	20.8958	10.3783	1.00965	0.0001	0.00312189
NM_016247.3	50939	IMPG2	8352	3.30593	1.41247	1.22684	0.0001	0.00312189

NM_003358.1	7357	UGCG	1637	62.39	30.6513	1.02537	0.0001	0.00312189
NM_005079.2	7163	TPD52	3961	32.4916	14.9664	1.11834	0.0001	0.00312189
NM_001678.3	482	ATP1B2	3332	0.762437	2.46752	-1.69437	0.0001	0.00312189
NM_000930.3	5327	PLAT	3173	13.9735	28.5812	-1.03237	0.0001	0.00312189
NM_006982.2	8092	ALX1	1294	1.12406	4.80268	-2.09512	0.0001	0.00312189
TCONS_00196366	XLOC_017565	-	4542	0.549801	2.00249	-1.86482	0.0001	0.00312189
NM_003937.2	8942	KYNU	1672	25.4138	6.80954	1.89998	0.0001	0.00312189
NM_002801.3	5699	PSMB10	1006	51.9012	24.4448	1.08624	0.0001	0.00312189
NM_019111.4	3122	HLA-DRA	1280	1499.11	472.152	1.66678	0.0001	0.00312189
TCONS_00155291	XLOC_013582	-	1719	0.185567	2.36377	-3.67108	0.0001	0.00312189
NM_182762.3	346389	MACC1	9159	18.9415	10.211	0.891423	0.0001	0.00312189
NM_002616.2	5187	PER1	4709	4.96497	9.60308	-0.951713	0.0001	0.00312189
NM_004665.2	8875	VNN2	2004	25.001	5.6722	2.14	0.0001	0.00312189
NM_001204376.1	4883	NPR3	6380	5.73845	1.65885	1.79048	0.0001	0.00312189
3495	3495	-	1154	76.9939	31.0776	1.30887	0.0001	0.00312189
3493	3493	-	1061	2466.19	410.424	2.5871	0.0001	0.00312189
NM_025159.2	80231	CXorf21	1927	7.87925	3.16143	1.31748	0.0001	0.00312189
NM_000211.3	3689	ITGB2	2958	27.7845	12.9395	1.10249	0.0001	0.00312189
NM_003613.3	8483	CILP	4468	0.455992	1.67811	-1.87976	0.0001	0.00312189
NM_002030.3	2359	FPR3	2517	26.7632	13.4726	0.990223	0.0001	0.00312189
NM_025079.2	80149	ZC3H12A	2716	6.66306	2.36571	1.49391	0.0001	0.00312189
NM_001199149.1	4057	LTF	2508	485.255	102.844	2.23829	0.0001	0.00312189
TCONS_00047584	XLOC_003947	-	3909	5.36261	14.2751	-1.41249	0.0001	0.00312189
ENST00000604271.2	ENSG00000270571.2	RP11-355F16.1	3438	2.54713	9.54114	-1.90529	0.0001	0.00312189
TCONS_00005779	XLOC_000192	-	16777	2.02085	6.62714	-1.71342	0.0001	0.00312189
NM_000888.3	3694	ITGB6	2397	9.80774	3.97843	1.30172	0.0001	0.00312189
NM_178026.2	2686	GGT7	2640	4.77749	10.8024	-1.17702	0.0001	0.00312189
NM_005996.3	6926	TBX3	4723	13.626	26.7393	-0.972606	0.0001	0.00312189
NM_001004303.4	199920	C1orf168	3460	4.61639	10.1833	-1.14137	0.0001	0.00312189
NM_014850.2	9901	SRGAP3	8901	4.53266	9.40501	-1.05307	0.0001	0.00312189
NM_001003845.2	389058	SP5	1947	0.622839	2.53688	-2.02613	0.0001	0.00312189
NM_016953.3	50940	PDE11A	9278	8.35974	3.84891	1.11901	0.0001	0.00312189
NM_080475.2	89778	SERPINB11	1731	6.30464	19.618	-1.63769	0.0001	0.00312189
NM_020760.1	57520	HECW2	6926	2.89578	6.19719	-1.09766	0.0001	0.00312189
NM_015914.5	51061	TXNDC11	3047	30.3862	13.9276	1.12547	0.0001	0.00312189
NM_003661.3	8542	APOL1	2863	42.9307	20.3028	1.08033	0.0001	0.00312189
NM_004877.2	9535	GMFG	657	50.1102	20.698	1.27562	0.0001	0.00312189
NM_005337.4	3071	NCKAP1L	3893	29.9674	15.6878	0.933746	0.0001	0.00312189
NM_004522.1	3800	KIF5C	6933	2.5609	0.995353	1.36337	0.0001	0.00312189
NM_144966.5	158326	FREM1	10086	1.86734	4.38803	-1.23259	0.0001	0.00312189
NM_032855.2	84941	HSH2D	2367	9.39671	4.01723	1.22595	0.00015	0.00437065
TCONS_00006208	XLOC_000770	-	3554	2.83672	6.42519	-1.17952	0.00015	0.00437065
NM_001860.2	1318	SLC31A2	1774	20.5591	9.24688	1.15274	0.00015	0.00437065
NM_003619.3	8492	PRSS12	4573	14.7944	27.5263	-0.895756	0.00015	0.00437065
NM_000587.2	730	C7	4012	21.6512	44.2936	-1.03265	0.00015	0.00437065
NM_020844.2	57604	KIAA1456	9590	3.14456	8.11618	-1.36794	0.00015	0.00437065
NM_001067.3	7153	TOP2A	5753	10.5628	4.72787	1.15972	0.00015	0.00437065
NM_020361.4	57094	CPA6	1904	0.339249	1.60206	-2.23951	0.00015	0.00437065
NM_000851.3	2949	GSTM5	1570	2.35722	7.12814	-1.59644	0.00015	0.00437065
NM_024913.4	79974	C7orf58	5320	7.07342	14.1535	-1.00068	0.00015	0.00437065
NM_007315.3	6772	STAT1	4308	294.177	94.0657	1.64495	0.00015	0.00437065
NM_005582.2	4064	CD180	2716	9.4064	3.95623	1.24951	0.00015	0.00437065
NM_052904.3	114792	KLHL32	3807	1.82112	4.80965	-1.40111	0.00015	0.00437065
NM_005447.3	9182	RASSF9	1796	14.7397	28.8602	-0.96937	0.00015	0.00437065

NM_000220.2	3758	KCNJ1	2332	0.875611	3.68891	-2.07483	0.00015	0.00437065
NM_001098843.3	645090	CXorf30	2863	6.73563	15.3101	-1.1846	0.00015	0.00437065
NM_003956.3	9023	CH25H	1378	7.25585	17.7909	-1.29392	0.00015	0.00437065
NM_144577.3	93233	CCDC114	3220	4.41656	9.73158	-1.13975	0.00015	0.00437065
NM_016445.1	26499	PLEK2	1460	9.43797	3.17207	1.57305	0.00015	0.00437065
NM_012252.2	22797	TFEC	6631	11.403	5.35801	1.08964	0.00015	0.00437065
NM_016021.2	51465	UBE2J1	4342	62.4642	31.4501	0.989968	0.00015	0.00437065
NM_001018071.3	143162	FRMPD2	5024	5.3588	10.6401	-0.989524	0.00015	0.00437065
NM_001369.2	1767	DNAH5	15573	34.1222	86.2259	-1.33741	0.00015	0.00437065
NM_003810.3	8743	TNFSF10	1953	176.591	90.7801	0.959968	0.00015	0.00437065
NM_005328.2	3037	HAS2	3275	4.18533	1.47874	1.50097	0.00015	0.00437065
NM_018475.3	55858	TMEM165	1965	71.651	38.0149	0.914423	0.00015	0.00437065
NM_001080471.1	375033	PEAR1	4866	4.64335	9.18066	-0.983434	0.00015	0.00437065
NM_015932.5	51371	POMP	1462	51.127	25.9147	0.980316	0.00015	0.00437065
NM_173050.3	80274	SCUBE1	3868	0.462918	1.63163	-1.81749	0.00015	0.00437065
NM_005202.2	1296	COL8A2	4447	10.0104	21.2432	-1.0855	0.00015	0.00437065
NM_152547.4	153579	BTNL9	3500	1.6415	4.53135	-1.46493	0.00015	0.00437065
NM_003221.3	7021	TFAP2B	5770	1.31863	3.17204	-1.26637	0.00015	0.00437065
NM_138456.3	116071	BATF2	2140	5.63184	1.88532	1.57879	0.00015	0.00437065
NM_003881.2	8839	WISP2	1404	4.02802	1.10189	1.87009	0.00015	0.00437065
NM_003278.2	7123	CLEC3B	852	93.9382	41.7291	1.17066	0.00015	0.00437065
NM_007356.2	22798	LAMB4	5858	1.11046	0.274688	2.01529	0.00015	0.00437065
TCONS_00187673	XLOC_016076	-	900	0.853881	4.93536	-2.53105	0.00015	0.00437065
TCONS_00048685	XLOC_003947	-	3649	1.38522	3.90145	-1.4939	0.00015	0.00437065
NM_002982.3	6347	CCL2	749	61.4655	27.8862	1.14022	0.00015	0.00437065
NM_004319.1	460	ASTN1	7314	1.88765	4.67112	-1.30718	0.00015	0.00437065
NM_012198.3	25801	GCA	3226	14.0182	6.82261	1.0389	0.00015	0.00437065
NM_030627.2	80315	CPEB4	7769	32.7505	17.4491	0.908368	0.0002	0.00554203
NM_001099772.1	1580	CYP4B1	2158	45.0329	128.138	-1.50865	0.0002	0.00554203
NM_003540.3	8361	HIST1H4F	368	123.763	48.6913	1.34584	0.0002	0.00554203
NM_203416.2	9332	CD163	4110	49.2608	21.4885	1.19688	0.0002	0.00554203
TCONS_00012089	XLOC_000775	-	4929	0.573268	1.84909	-1.68954	0.0002	0.00554203
NM_005654.4	7025	NR2F1	3199	1.25699	3.96515	-1.65741	0.0002	0.00554203
NM_001195286.1	3662	IRF4	5324	8.36624	2.36087	1.82526	0.0002	0.00554203
NM_012244.2	23428	SLC7A8	4216	4.97323	10.0652	-1.01712	0.0002	0.00554203
NM_145051.3	138065	RNF183	1210	2.60384	0.469263	2.47217	0.0002	0.00554203
NM_001225.3	837	CASP4	1319	112.223	62.3028	0.848998	0.0002	0.00554203
NM_032048.2	84034	EMILIN2	4009	11.5183	5.86863	0.972827	0.0002	0.00554203
NM_000161.2	2643	GCH1	2926	16.5023	7.82924	1.07572	0.0002	0.00554203
NM_006762.2	7805	LAPTM5	2240	93.036	45.4328	1.03405	0.0002	0.00554203
NM_021129.3	5464	PPA1	1302	107.666	54.4734	0.982944	0.0002	0.00554203
NM_001112718.1	22998	LIMCH1	6084	12.719	24.3684	-0.938024	0.0002	0.00554203
NM_004431.3	1969	EPHA2	3964	5.11571	10.0569	-0.975176	0.0002	0.00554203
NM_015900.3	51365	PLA1A	1780	11.4027	3.08154	1.88765	0.0002	0.00554203
NM_138554.3	7099	TLR4	5653	29.0756	15.526	0.905122	0.0002	0.00554203
NM_199204.1	10170	DHRS9	1917	47.4187	20.6595	1.19865	0.0002	0.00554203
NM_001775.2	952	CD38	1491	28.9395	12.6906	1.18928	0.0002	0.00554203
NM_032793.3	84879	MFSD2A	2162	6.77281	2.42036	1.48453	0.0002	0.00554203
TCONS_00299614	XLOC_024794	-	7010	1.07514	0.291239	1.88426	0.0002	0.00554203
ENST00000517927.1	ENSG00000253522.2	MIR146A	2301	4.34139	1.51442	1.51939	0.0002	0.00554203
NM_018086.2	55137	FIGN	4535	1.8845	4.33604	-1.2022	0.0002	0.00554203
NM_004711.4	9145	SYNGR1	4438	3.01869	6.96169	-1.20551	0.0002	0.00554203
NM_017688.2	54836	BSPRY	2348	9.6654	3.86445	1.32257	0.0002	0.00554203
TCONS_00196029	XLOC_017238	-	2296	0.361734	1.62119	-2.16405	0.0002	0.00554203

NM_001953.3	1890	TYMP	1659	26.8081	5.18487	2.37029	0.0002	0.00554203
NM_004417.3	1843	DUSP1	2024	53.0246	27.4142	0.951737	0.0002	0.00554203
TCONS_00114410	XLOC_009730	-	4688	16.0291	32.4166	-1.01604	0.0002	0.00554203
NM_001692.3	525	ATP6V1B1	1939	1.94418	6.22053	-1.67788	0.0002	0.00554203
NM_001046.2	6558	SLC12A2	6860	55.2115	26.3532	1.06699	0.0002	0.00554203
NM_145000.3	202151	RANBP3L	2543	0.806251	2.49278	-1.62846	0.00025	0.00665625
NM_001778.2	962	CD48	1070	53.7253	27.1837	0.98286	0.00025	0.00665625
NM_001032.3	6235	RPS29	296	1922.25	1037.46	0.889739	0.00025	0.00665625
NM_017773.3	54900	LAX1	3298	15.515	4.36716	1.8289	0.00025	0.00665625
NM_003332.3	7305	TYROBP	591	33.9205	11.7458	1.53002	0.00025	0.00665625
NM_145290.2	166647	GPR125	4560	10.5656	19.6396	-0.894393	0.00025	0.00665625
NM_001045.4	6532	SLC6A4	4535	0.287194	1.08847	-1.9222	0.00025	0.00665625
NM_173462.3	89932	PAPLN	5834	2.13347	4.51797	-1.08247	0.00025	0.00665625
NM_005958.3	4543	MTNR1A	1105	3.61336	0.556358	2.69925	0.00025	0.00665625
NM_052846.1	90187	EMILIN3	3827	0.798878	3.64216	-2.18875	0.00025	0.00665625
NM_016341.3	51196	PLCE1	7992	7.8013	14.6711	-0.911193	0.00025	0.00665625
NM_197941.2	11174	ADAMTS6	7268	2.94526	1.34298	1.13295	0.00025	0.00665625
NM_002530.2	4916	NTRK3	2818	1.84269	8.48226	-2.20263	0.00025	0.00665625
NM_014571.3	26508	HEYL	4114	1.07222	2.86891	-1.4199	0.00025	0.00665625
NM_001033723.2	619279	ZNF704	14386	7.70442	14.0041	-0.862086	0.00025	0.00665625
NM_153840.2	266977	GPR110	3078	16.1124	7.43966	1.11486	0.00025	0.00665625
NM_016562.3	51284	TLR7	4992	7.00989	3.41397	1.03794	0.00025	0.00665625
NM_002661.2	5336	PLCG2	4241	16.7802	8.93604	0.909057	0.00025	0.00665625
NM_017423.2	51809	GALNT7	4307	22.7749	11.8354	0.944341	0.00025	0.00665625
NM_001530.3	3091	HIF1A	4059	69.53	37.4411	0.893015	0.00025	0.00665625
NM_031422.4	83539	CHST9	2246	59.9675	118.083	-0.977543	0.00025	0.00665625
NM_052909.3	153478	PLEKHG4B	11515	0.513103	1.24942	-1.28394	0.00025	0.00665625
NM_001460.2	2327	FMO2	5181	41.7565	116.347	-1.47835	0.00025	0.00665625
NM_002198.2	3659	IRF1	3554	29.911	12.3937	1.27107	0.00025	0.00665625
NM_022097.2	63928	CHP2	2366	5.60138	12.9581	-1.21	0.00025	0.00665625
NM_144650.2	137872	ADHFE1	1895	2.20059	5.88896	-1.42013	0.00025	0.00665625
NM_173615.3	146177	VWA3A	4600	5.90151	11.1983	-0.92412	0.00025	0.00665625
NM_144975.3	162394	SLFN5	4654	75.6605	39.5832	0.934653	0.00025	0.00665625
NM_032744.3	84830	C6orf105	1804	5.54516	1.50068	1.88562	0.00025	0.00665625
NM_153228.2	162282	ANKFN1	2426	4.73041	10.9039	-1.2048	0.00025	0.00665625
NM_133460.1	147686	ZNF418	3694	2.59181	5.88712	-1.1836	0.00025	0.00665625
NM_001559.2	3595	IL12RB2	4040	3.1567	1.17559	1.42503	0.00025	0.00665625
NM_201563.4	9103	FCGR2C	1510	15.6102	6.40509	1.2852	0.00025	0.00665625
NM_000898.4	4129	MAOB	2595	6.04954	13.685	-1.17769	0.00025	0.00665625
NM_005467.3	10003	NAALAD2	3196	4.35563	9.39162	-1.10849	0.00025	0.00665625
NM_006890.3	1087	CEACAM7	2289	2.41161	0.632481	1.9309	0.00025	0.00665625
NM_018235.2	55748	CNDP2	5073	26.5522	14.4329	0.87947	0.0003	0.00772176
NM_144980.3	168090	C6orf118	1839	12.7925	25.5535	-0.998217	0.0003	0.00772176
NM_001854.3	1301	COL11A1	7291	12.5191	32.4633	-1.37468	0.0003	0.00772176
NM_001170820.3	402778	IFITM10	3709	1.00254	2.86259	-1.51367	0.0003	0.00772176
NM_001676.5	479	ATP12A	3716	8.72366	3.85656	1.17762	0.0003	0.00772176
NM_014692.1	9717	SEC14L5	6456	0.349343	1.09483	-1.648	0.0003	0.00772176
NM_198795.1	56165	TDRD1	4510	0.829198	2.36099	-1.5096	0.0003	0.00772176
NM_002232.3	3738	KCNA3	3346	14.7216	7.64595	0.945172	0.0003	0.00772176
NM_032991.2	836	CASP3	2506	41.8237	19.9246	1.06977	0.0003	0.00772176
TCONS_00032236	XLOC_003060	-	7128	0.659161	1.74886	-1.40771	0.0003	0.00772176
NM_014058.3	28983	TMPRSS11E	2136	2.63046	0.6013	2.12916	0.0003	0.00772176
XM_003403505.1	100652743	LOC100652743	456	22.3162	6.28177	1.82885	0.0003	0.00772176
NM_014786.3	9828	ARHGEF17	7813	4.15059	7.80547	-0.911167	0.0003	0.00772176

NM_002864.2	5858	PZP	4610	5.29491	10.6603	-1.00957	0.0003	0.00772176
NM_006622.2	10769	PLK2	2786	81.3219	44.1348	0.881729	0.0003	0.00772176
NM_006068.4	10333	TLR6	5891	7.24723	3.68006	0.977702	0.0003	0.00772176
NM_198595.2	60312	AFAP1	7518	4.89365	9.42891	-0.946179	0.0003	0.00772176
NM_014746.3	9781	RNF144A	5740	6.54994	12.3283	-0.912421	0.0003	0.00772176
NM_007035.3	11081	KERA	2533	0.404639	1.56337	-1.94996	0.0003	0.00772176
NM_198391.2	23767	FLRT3	4024	2.97372	11.3487	-1.93219	0.0003	0.00772176
NM_000096.3	1356	CP	4666	424.023	146.162	1.53657	0.0003	0.00772176
NM_152866.2	931	MS4A1	3594	20.5402	6.09159	1.75356	0.00035	0.00869612
NM_130783.4	90139	TSPAN18	4235	1.5668	5.4193	-1.79028	0.00035	0.00869612
NM_001995.2	2180	ACSL1	3805	27.8277	15.5685	0.837898	0.00035	0.00869612
XM_002343882.1	100287163	LOC100287163	2715	2.99999	6.98949	-1.22023	0.00035	0.00869612
NM_020693.2	57453	DSCAML1	6899	0.346787	1.03849	-1.58237	0.00035	0.00869612
NM_021982.1	10802	SEC24A	6387	22.5906	12.7041	0.830426	0.00035	0.00869612
NM_020299.4	57016	AKR1B10	1596	3.95944	1.1011	1.84635	0.00035	0.00869612
NM_000130.4	2153	F5	9179	4.60575	2.19784	1.06735	0.00035	0.00869612
NM_033225.5	64478	CSMD1	14317	0.550455	1.28835	-1.22683	0.00035	0.00869612
NM_005514.6	3106	HLA-B	1572	960.619	389.021	1.30411	0.00035	0.00869612
NM_020925.2	57685	CACHD1	5274	13.8471	25.3886	-0.874599	0.00035	0.00869612
NM_152688.2	202559	KHDRBS2	2300	2.44316	6.29703	-1.36592	0.00035	0.00869612
NM_001165.3	330	BIRC3	5229	53.0886	27.1518	0.967354	0.00035	0.00869612
NM_145739.2	114880	OSBPL6	6811	2.65405	7.64767	-1.52683	0.00035	0.00869612
NM_173799.3	201633	TIGIT	2968	8.63215	3.80273	1.18268	0.00035	0.00869612
NM_014752.2	9789	SPCS2	2708	22.2905	11.7304	0.92617	0.00035	0.00869612
NM_031912.4	83849	SYT15	5508	6.28864	11.8717	-0.9167	0.00035	0.00869612
NM_001077624.1	162993	ZNF846	2133	8.50817	17.3222	-1.0257	0.00035	0.00869612
TCONS_00297081	XLOC_025491	-	9630	1.20542	0.455561	1.40382	0.00035	0.00869612
XM_001715090.3	144535	C12orf55	5721	16.3407	29.9599	-0.874561	0.00035	0.00869612
NM_181449.2	342510	CD300E	3525	2.35534	0.779358	1.59558	0.00035	0.00869612
NM_001175.4	397	ARHGDIB	1216	145.249	79.4677	0.870092	0.00035	0.00869612
NM_001144058.1	50863	NTM	3408	1.42491	5.62309	-1.98049	0.00035	0.00869612
NM_006536.5	9635	CLCA2	4025	6.52949	2.5704	1.34498	0.00035	0.00869612
NM_003509.2	8329	HIST1H2AI	469	19.7312	4.52326	2.12505	0.00035	0.00869612
NM_001719.2	655	BMP7	4031	3.1461	6.93736	-1.14082	0.00035	0.00869612
NM_004445.3	2051	EPHB6	4043	2.8412	6.27992	-1.14424	0.00035	0.00869612
TCONS_00180095	XLOC_015624	-	1504	0.545734	2.43413	-2.15713	0.00035	0.00869612
NM_145287.3	162655	ZNF519	6810	1.98114	4.32797	-1.12736	0.00035	0.00869612
NM_170736.1	3772	KCNJ15	2919	14.2357	1.83368	2.9567	0.0004	0.00971957
NM_002009.3	2252	FGF7	3936	72.2474	32.1	1.17037	0.0004	0.00971957
NM_002818.2	5721	PSME2	829	139.51	73.7399	0.919854	0.0004	0.00971957
XM_003118942.2	374467	C12orf63	2401	12.83	24.4605	-0.930935	0.0004	0.00971957
NM_000615.6	4684	NCAM1	5962	2.3043	5.78442	-1.32784	0.0004	0.00971957
NM_014848.4	9899	SV2B	11343	0.357224	1.02024	-1.51401	0.0004	0.00971957
NM_021647.6	9848	MFAP3L	6189	1.97214	5.02428	-1.34915	0.0004	0.00971957
NM_007364.2	23423	TMED3	1388	37.7388	19.8637	0.925914	0.0004	0.00971957
NM_015541.2	26018	LRIG1	4762	25.607	47.6389	-0.895604	0.0004	0.00971957
NM_144590.2	118932	ANKRD22	3986	4.57059	1.79234	1.35054	0.0004	0.00971957
NM_019107.3	56005	C19orf10	1051	37.113	18.688	0.989814	0.0004	0.00971957
NM_147175.3	90161	HS6ST2	4447	0.563872	1.77242	-1.65228	0.0004	0.00971957
NM_002084.3	2878	GPX3	1761	18.9579	36.172	-0.93207	0.0004	0.00971957
NM_002526.3	4907	NT5E	4068	15.2516	27.6155	-0.856522	0.00045	0.0106989
NM_014903.4	89795	NAV3	9758	3.01626	5.85657	-0.957295	0.00045	0.0106989
NM_001409.3	1953	MEGF6	7439	3.12613	5.80969	-0.894082	0.00045	0.0106989
NM_178232.2	145864	HAPLN3	1893	8.36026	3.63138	1.20303	0.00045	0.0106989

NM_001195278.1	100507421	LOC100507421	10558	1.73683	0.716474	1.27747	0.00045	0.0106989
NM_014079.3	28999	KLF15	2525	2.07674	4.99131	-1.2651	0.00045	0.0106989
NM_001146187.1	5178	PEG3	8375	2.27494	5.29089	-1.21769	0.00045	0.0106989
NM_052972.2	116844	LRG1	1780	12.4003	5.41354	1.19573	0.00045	0.0106989
NM_020877.2	146754	DNAH2	13505	5.95256	10.7199	-0.848715	0.00045	0.0106989
NM_144992.4	200403	VWA3B	4747	10.3993	18.6574	-0.843257	0.00045	0.0106989
NM_032704.3	84790	TUBA1C	1553	28.469	14.6569	0.957816	0.00045	0.0106989
NM_181077.3	23015	GOLGA8A	4264	15.1018	27.2026	-0.849023	0.00045	0.0106989
NM_000168.5	2737	GLI3	8215	3.49226	6.43156	-0.881007	0.00045	0.0106989
NM_006228.3	5368	PNOC	1196	3.49132	0.867359	2.00907	0.00045	0.0106989
NM_002250.2	3783	KCNN4	2240	4.41996	1.674	1.40073	0.00045	0.0106989
TCONS_00234151	XLOC_019814	-	4203	0.565797	1.63031	-1.5268	0.0005	0.0116249
NM_001037160.2	192668	CYS1	2719	2.09495	5.23765	-1.32201	0.0005	0.0116249
NM_005668.4	7903	ST8SIA4	6320	17.4191	9.68114	0.847424	0.0005	0.0116249
NM_025015.2	259217	HSPA12A	5722	1.6324	3.58952	-1.1368	0.0005	0.0116249
NM_012400.2	26279	PLA2G2D	2630	1.58886	0.467278	1.76564	0.0005	0.0116249
NM_012413.3	25797	QPCT	1703	9.90897	4.26102	1.21754	0.0005	0.0116249
NM_000396.3	1513	CTSK	1810	58.0294	127.079	-1.13087	0.0005	0.0116249
TCONS_00012664	XLOC_001501	-	1925	0.393922	1.65222	-2.06842	0.0005	0.0116249
TCONS_00180182	XLOC_015712	-	1504	0.2368	1.70663	-2.84941	0.0005	0.0116249
NM_001098623.1	84033	OBSCN	24030	0.525467	1.33465	-1.34479	0.0005	0.0116249
NM_000231.2	6445	SGCG	1655	0.216968	1.60134	-2.88372	0.0005	0.0116249
NM_014398.3	27074	LAMP3	3341	8.24553	4.09786	1.00874	0.0005	0.0116249
NM_001031683.2	3437	IFIT3	2454	40.5776	17.1504	1.24244	0.0005	0.0116249
NM_032679.2	84765	ZNF577	3096	5.49141	11.5661	-1.07465	0.0005	0.0116249
NM_001792.3	1000	CDH2	4367	2.49161	5.61369	-1.17187	0.0005	0.0116249
NM_017424.2	51816	CECR1	3927	23.5747	9.39168	1.32778	0.0005	0.0116249
NM_021800.2	56521	DNAJC12	1212	3.9366	0.426096	3.2077	0.0005	0.0116249
NM_018445.4	55829	SELS	1234	55.8223	27.1305	1.04092	0.0005	0.0116249
NM_003514.2	8336	HIST1H2AM	487	75.3388	32.2639	1.22347	0.0005	0.0116249
TCONS_00272364	XLOC_022448	-	770	20.8288	7.01655	1.56975	0.0005	0.0116249
NM_052843.2	84033	OBSCN	20402	0.74862	1.81462	-1.27736	0.0005	0.0116249
NM_005335.4	3059	HCLS1	2033	62.7727	33.2565	0.916501	0.00055	0.0124813
NM_194071.2	64764	CREB3L2	7455	25.6763	14.7643	0.798327	0.00055	0.0124813
NM_014791.2	9833	MELK	2439	2.86879	0.952747	1.59028	0.00055	0.0124813
NM_001129908.2	729085	FAM198A	3433	1.54762	3.76574	-1.28288	0.00055	0.0124813
NM_002825.5	5764	PTN	1549	33.1337	60.3928	-0.866077	0.00055	0.0124813
NM_145252.2	124220	ZG16B	828	142.959	61.1025	1.2263	0.00055	0.0124813
NM_001113490.1	154796	AMOT	6945	6.06735	12.2304	-1.01133	0.00055	0.0124813
NM_152864.3	128414	NKAIN4	1430	0.66951	2.45336	-1.87358	0.00055	0.0124813
NM_021064.4	8969	HIST1H2AG	498	66.2763	29.175	1.18376	0.00055	0.0124813
NM_182920.1	56999	ADAMTS9	7313	6.44703	11.7794	-0.869562	0.00055	0.0124813
NM_004049.3	597	BCL2A1	887	14.7406	4.45226	1.72719	0.00055	0.0124813
NM_001362.3	1735	DIO3	2102	2.70345	0.849685	1.6698	0.00055	0.0124813
NM_005221.5	1749	DLX5	1424	0.861593	3.08079	-1.83822	0.00055	0.0124813
NM_003468.3	7855	FZD5	6564	5.57217	10.1758	-0.868836	0.00055	0.0124813
NM_015444.2	25907	TMEM158	1859	2.17294	0.548307	1.98659	0.00055	0.0124813
NM_030641.3	80830	APOL6	10139	67.834	31.8179	1.09217	0.00055	0.0124813
NM_005080.3	7494	XBP1	1820	436.087	168.768	1.36957	0.00055	0.0124813
NM_001242908.1	284654	RSPO1	2910	5.62266	12.2863	-1.12772	0.00055	0.0124813
NM_138780.2	94122	SYTL5	4726	4.09938	1.08603	1.91634	0.00055	0.0124813
NM_024420.2	5321	PLA2G4A	2928	21.8761	11.9493	0.872426	0.00055	0.0124813
NM_001793.4	1001	CDH3	4276	16.3987	8.94233	0.874856	0.00055	0.0124813
NM_002951.3	6185	RPN2	2508	94.0982	52.148	0.851556	0.00055	0.0124813

NM_032184.1	84144	SYDE2	4711	4.40173	8.36443	-0.926196	0.00055	0.0124813
NM_205855.3	389558	FAM180A	1801	0.856059	3.18214	-1.89421	0.0006	0.013408
NM_000683.3	152	ADRA2C	1958	1.9681	5.46691	-1.47392	0.0006	0.013408
NM_001194986.1	388630	LOC388630	7029	0.314465	0.885073	-1.49289	0.0006	0.013408
NM_001747.2	822	CAPG	1442	32.8522	17.5555	0.904069	0.0006	0.013408
NM_152750.4	222256	CDHR3	6500	27.2359	50.6512	-0.895088	0.0006	0.013408
NM_002408.3	4247	MGAT2	2711	35.0707	19.6615	0.834889	0.0006	0.013408
NM_003004.2	6398	SECTM1	2282	7.55129	3.23225	1.22419	0.0006	0.013408
TCONS_00192180	XLOC_016641	-	1912	1.6858	0.372616	2.17768	0.0006	0.013408
NM_033278.2	10612	TRIM3	2872	2.47889	5.67588	-1.19515	0.0006	0.013408
NM_002539.1	4953	ODC1	2062	40.4057	22.4983	0.844744	0.0006	0.013408
NM_144973.3	160518	DENND5B	9391	10.101	5.61932	0.846035	0.0006	0.013408
NM_001080508.1	9096	TBX18	4070	2.66174	5.73194	-1.10665	0.0006	0.013408
NM_002610.3	5163	PDK1	4576	43.9574	9.17787	2.25988	0.0006	0.013408
NM_152744.3	221935	SDK1	10397	4.77616	8.73352	-0.870712	0.0006	0.013408
NM_000820.2	2621	GAS6	2505	17.3378	31.4432	-0.858825	0.0006	0.013408
NM_002627.4	5214	PFKP	2657	24.1835	13.2778	0.865005	0.0006	0.013408
NM_014141.5	26047	CNTNAP2	9894	0.454973	1.14194	-1.32763	0.0006	0.013408
NM_019609.4	56265	CPXM1	2400	34.8521	62.6989	-0.847192	0.0006	0.013408
TCONS_00259832	XLOC_021398	-	724	5.1404	0.503714	3.3512	0.00065	0.0142695
NM_001025598.1	257106	ARHGAP30	4425	15.7537	8.74023	0.849952	0.00065	0.0142695
NM_003564.1	8407	TAGLN2	1360	130.903	73.5734	0.831245	0.00065	0.0142695
TCONS_00254192	XLOC_021827	-	2082	0.674926	2.38035	-1.81837	0.00065	0.0142695
NM_004585.3	5920	RARRES3	765	118.171	64.0438	0.88374	0.00065	0.0142695
NM_017923.3	55016	MARCH1	5389	24.0092	13.0466	0.879915	0.00065	0.0142695
NM_031455.3	83643	CCDC3	2738	31.0493	56.3623	-0.860168	0.00065	0.0142695
NM_001289.4	1193	CLIC2	2686	17.2866	9.31415	0.89216	0.00065	0.0142695
NM_207361.4	341640	FREM2	16163	3.11999	5.68093	-0.864583	0.00065	0.0142695
NM_021018.2	8968	HIST1H3F	466	25.8534	7.88994	1.71226	0.00065	0.0142695
NM_080489.4	27111	SDCBP2	1581	4.17642	1.00872	2.04974	0.00065	0.0142695
NM_173549.2	203111	C8orf47	1550	12.5778	26.9158	-1.09757	0.00065	0.0142695
NM_198428.2	27241	BBS9	4019	15.278	27.5062	-0.8483	0.00065	0.0142695
NM_018990.3	54440	SASH3	2694	15.6595	7.6961	1.02484	0.00065	0.0142695
NM_025227.1	80341	BPIL1	1897	1.44468	0.202507	2.8347	0.00065	0.0142695
NM_006808.2	10952	SEC61B	562	82.3147	41.3159	0.994454	0.00065	0.0142695
NM_001135733.1	94241	TP53INP1	5652	52.3853	23.9929	1.12655	0.0007	0.015106
NM_000336.2	6338	SCNN1B	2597	10.0763	4.76613	1.08007	0.0007	0.015106
NM_001001395.1	55885	LMO3	3450	0.585699	3.07721	-2.39339	0.0007	0.015106
NM_018836.3	55966	AJAP1	2923	1.05643	3.15856	-1.58007	0.0007	0.015106
NM_019016.2	192666	KRT24	1881	1.71115	0.170365	3.32826	0.0007	0.015106
NM_002046.3	2597	GAPDH	1310	579.618	290.794	0.995108	0.0007	0.015106
NM_015461.2	25925	ZNF521	4971	7.74157	14.1903	-0.874204	0.0007	0.015106
NM_001200049.1	54777	C10orf92	3164	1.94165	4.63382	-1.25492	0.0007	0.015106
NM_004273.4	9469	CHST3	6973	4.81123	8.60662	-0.839043	0.0007	0.015106
NM_001130058.1	204962	SLC44A5	3894	0.334828	1.35922	-2.02128	0.0007	0.015106
NM_001152.4	292	SLC25A5	1351	79.9183	45.4862	0.813096	0.0007	0.015106
NM_002612.3	5166	PDK4	3700	26.9624	51.8992	-0.944764	0.0007	0.015106
NM_000698.2	240	ALOX5	2554	13.8077	6.86329	1.0085	0.0007	0.015106
NM_152666.2	200150	PLD5	3305	0.562996	3.89267	-2.78956	0.0007	0.015106
NM_003519.3	8340	HIST1H2BL	453	23.7767	7.87515	1.59417	0.0007	0.015106
NM_024324.3	79174	CRELD2	1428	21.7835	10.57	1.04327	0.0007	0.015106
NM_178012.4	347733	TUBB2B	2019	1.94341	0.574239	1.75887	0.00075	0.0159498
NM_001110514.1	57593	EBF4	2911	0.999062	2.62065	-1.39128	0.00075	0.0159498
NM_003474.4	8038	ADAM12	6093	6.3368	11.5608	-0.867415	0.00075	0.0159498

NM_004574.3	5414	SEPT4	1856	3.4041	12.6001	-1.88809	0.00075	0.0159498
NM_000061.2	695	BTK	2611	12.1575	6.12768	0.988437	0.00075	0.0159498
NM_001013647.1	646851	LOC646851	10322	2.98194	5.49157	-0.880965	0.00075	0.0159498
NM_005618.3	28514	DLL1	3310	2.19691	4.87823	-1.15089	0.00075	0.0159498
TCONS_00266591	XLOC_023026	-	1890	0.4354	1.58043	-1.85991	0.00075	0.0159498
NM_004288.4	9595	CYTIP	2210	48.1469	26.2065	0.877521	0.00075	0.0159498
NM_001608.3	33	ACADL	2492	0.727229	2.24865	-1.62858	0.00075	0.0159498
NM_001001872.2	145407	C14orf37	3082	4.22888	8.73804	-1.04703	0.00075	0.0159498
NM_012409.2	23627	PRND	3980	0.375937	1.15811	-1.6232	0.00075	0.0159498
NM_003719.3	8622	PDE8B	4405	3.69607	12.2651	-1.7305	0.00075	0.0159498
NM_001167738.1	89796	NAV1	11683	5.59882	10.3894	-0.89192	0.00075	0.0159498
NM_003062.2	6586	SLIT3	5380	6.12088	11.258	-0.879137	0.00075	0.0159498
NM_001031709.2	55328	RNLS	2416	5.18267	11.2693	-1.12063	0.00075	0.0159498
3505	3505	-	1188	2.29208	0.520062	2.1399	0.00075	0.0159498
NM_016472.3	51527	C14orf129	2072	21.4193	11.4352	0.905437	0.00075	0.0159498
NM_080596.1	85235	HIST1H2AH	439	34.9728	13.0957	1.41714	0.00075	0.0159498
NM_032291.2	84251	SGIP1	4694	2.73182	1.11917	1.28743	0.00075	0.0159498
NM_018407.4	55353	LAPTM4B	2238	28.0431	15.4569	0.859399	0.0008	0.0168217
NM_173535.2	165530	CLEC4F	2475	0.774704	2.46907	-1.67225	0.0008	0.0168217
TCONS_00265890	XLOC_022389	-	2835	0.361976	1.30767	-1.85303	0.0008	0.0168217
NM_020208.3	54716	SLC6A20	5432	4.27097	2.04512	1.06237	0.0008	0.0168217
NM_144665.2	143686	SESN3	1987	60.62	104.963	-0.792016	0.0008	0.0168217
ENST00000558411.1	ENSG00000259641.1	RP11-279F6.3	2121	0.248418	1.19357	-2.26445	0.0008	0.0168217
NM_032646.5	94015	TTYH2	3447	1.83208	4.85409	-1.40572	0.0008	0.0168217
NM_181644.4	148808	MFSD4	4088	8.60961	4.50309	0.935034	0.0008	0.0168217
NM_002424.2	4317	MMP8	3056	0.13973	0.880773	-2.65613	0.0008	0.0168217
NM_015065.2	23086	EXPH5	10187	22.3952	40.9518	-0.870737	0.0008	0.0168217
NM_005761.2	10154	PLXNC1	7346	17.8052	10.1593	0.809489	0.0008	0.0168217
NM_001254.3	990	CDC6	3053	2.2121	0.813722	1.44281	0.0008	0.0168217
NM_153274.2	266675	BEST4	2096	1.20193	3.22982	-1.4261	0.0008	0.0168217
NM_177455.3	168620	BHLHA15	706	6.56593	1.9044	1.78567	0.0008	0.0168217
NM_152513.3	150365	MEI1	4006	2.28946	0.889458	1.36401	0.0008	0.0168217
NM_001062.3	6947	TCN1	1567	59.922	24.9139	1.26613	0.0008	0.0168217
NM_018402.1	55801	IL26	1047	2.5518	0.573828	2.15282	0.0008	0.0168217
TCONS_00241593	XLOC_020747	-	2844	1.79828	0.30483	2.56054	0.00085	0.0176689
NM_004223.4	9246	UBE2L6	1261	70.8983	39.8198	0.832266	0.00085	0.0176689
NM_000618.3	3479	IGF1	7321	6.99408	3.57479	0.968276	0.00085	0.0176689
TCONS_00153471	XLOC_013915	-	2214	2.07115	0.563462	1.87804	0.00085	0.0176689
NM_001077427.2	116372	LYPD1	1974	4.64666	12.782	-1.45985	0.00085	0.0176689
NM_001172624.1	4756	NEO1	7055	11.2077	20.2755	-0.855251	0.00085	0.0176689
NM_031456.3	10517	FBXW10	3439	1.51755	3.57936	-1.23796	0.00085	0.0176689
NM_001008784.2	344807	CD200R1L	1296	0.275457	2.00386	-2.86288	0.00085	0.0176689
NM_021965.3	5239	PGM5	3338	5.92989	11.5312	-0.959459	0.00085	0.0176689
28834	28834	-	318	65.6492	17.1441	1.93706	0.00085	0.0176689
NM_003737.2	8642	DCHS1	10756	3.13831	5.60928	-0.837831	0.00085	0.0176689
NM_002266.2	3838	KPNA2	1980	15.3342	7.89084	0.958505	0.00085	0.0176689
NM_014265.4	10863	ADAM28	3220	125.277	61.258	1.03215	0.0009	0.0184743
NM_016267.3	51442	VGLL1	1215	0.738734	2.72714	-1.88426	0.0009	0.0184743
NM_004833.1	9447	AIM2	1484	10.6108	4.48489	1.24239	0.0009	0.0184743
NM_003511.2	8332	HIST1H2AL	470	25.4854	8.03838	1.66469	0.0009	0.0184743
NM_000528.3	4125	MAN2B1	3224	26.2749	14.3094	0.876721	0.0009	0.0184743
NM_021052.2	3012	HIST1H2AE	564	95.4411	49.8457	0.937142	0.0009	0.0184743
TCONS_00156810	XLOC_015004	-	2828	2.02673	4.75247	-1.22953	0.0009	0.0184743
NM_032960.2	9261	MAPKAPK2	3052	25.1577	13.431	0.905433	0.0009	0.0184743

NM_002298.4	3936	LCP1	3790	105.497	54.3309	0.957356	0.0009	0.0184743
NM_138806.3	131450	CD200R1	2272	9.34796	4.05556	1.20475	0.0009	0.0184743
NM_014631.2	9644	SH3PXD2A	11247	7.90235	13.7492	-0.798989	0.0009	0.0184743
NM_005024.1	5273	SERPINB10	1194	1.07376	3.85368	-1.84356	0.0009	0.0184743
NM_003238.3	7042	TGFB2	5870	4.7119	8.80374	-0.901806	0.00095	0.0192772
NM_003006.4	6404	SELPLG	2573	14.2582	7.42515	0.941299	0.00095	0.0192772
NM_000216.2	3730	KAL1	6314	9.11964	16.305	-0.838269	0.00095	0.0192772
NM_002445.3	4481	MSR1	2960	22.435	9.84989	1.18757	0.00095	0.0192772
NM_013227.3	176	ACAN	8840	0.270111	1.97711	-2.87177	0.00095	0.0192772
NM_005409.4	6373	CXCL11	1606	25.3491	6.67678	1.92471	0.00095	0.0192772
NM_012081.5	22936	ELL2	6046	43.621	24.7787	0.815919	0.00095	0.0192772
NM_007107.3	6747	SSR3	3716	83.7081	48.0381	0.80119	0.00095	0.0192772
NM_152862.1	10109	ARPC2	1448	178.896	102.67	0.801107	0.00095	0.0192772
NM_023073.3	65250	C5orf42	11199	18.5595	33.5119	-0.852517	0.00095	0.0192772
NM_005508.4	1233	CCR4	1657	7.70633	3.11228	1.30807	0.00095	0.0192772
NM_001168368.1	57452	GALNTL1	4309	5.01003	10.4107	-1.05517	0.001	0.0199964
NM_007038.3	11096	ADAMTS5	9663	3.5609	6.34585	-0.833573	0.001	0.0199964
NM_152793.2	222166	C7orf41	5814	17.0546	29.7241	-0.801472	0.001	0.0199964
NM_152989.2	6660	SOX5	4546	2.58735	5.94106	-1.19924	0.001	0.0199964
NM_006639.2	10800	CYSLTR1	1514	15.7628	7.66382	1.04039	0.001	0.0199964
NM_001142504.2	9754	STARD8	4840	2.54048	5.84299	-1.20161	0.001	0.0199964
NM_012450.2	26266	SLC13A4	2896	0.459755	1.41974	-1.62669	0.001	0.0199964
NM_002168.2	3418	IDH2	1733	53.3653	30.9814	0.7845	0.001	0.0199964
NM_000362.4	7078	TIMP3	5486	30.6424	54.3823	-0.827606	0.001	0.0199964
NM_080593.2	85236	HIST1H2BK	839	85.0896	45.2578	0.910816	0.001	0.0199964
TCONS_00297361	XLOC_024773	-	1955	0.783058	2.39698	-1.61402	0.001	0.0199964
NM_003966.2	9037	SEMA5A	11808	14.6351	26.5099	-0.857097	0.001	0.0199964
NM_020813.2	57573	ZNF471	4967	4.45557	8.26025	-0.890575	0.001	0.0199964
NM_001843.2	1272	CNTN1	3427	12.8639	22.902	-0.832144	0.001	0.0199964
NM_001584.2	744	MPPED2	2402	5.38066	11.8013	-1.13309	0.001	0.0199964
XM_003118524.1	389197	C4orf50	3184	0.533624	1.56884	-1.5558	0.001	0.0199964
XM_003403521.1	100652860	LOC100652860	1520	3.23298	7.85582	-1.2809	0.00105	0.0207193
NM_004836.5	9451	EIF2AK3	4648	32.7705	19.1087	0.778173	0.00105	0.0207193
NM_005270.4	2736	GLI2	6769	0.455364	1.13125	-1.31282	0.00105	0.0207193
NM_005507.2	1072	CFL1	1260	147.256	85.6049	0.782559	0.00105	0.0207193
NM_006843.2	10993	SDS	1605	1.38941	0.322729	2.10607	0.00105	0.0207193
NM_004588.4	6327	SCN2B	4922	0.610597	1.60203	-1.39161	0.00105	0.0207193
NM_182497.3	125115	KRT40	1938	2.93038	1.0394	1.49535	0.00105	0.0207193
NM_001428.3	2023	ENO1	2187	134.316	74.8777	0.843021	0.00105	0.0207193
NM_002208.4	3682	ITGAE	3861	6.84969	3.1224	1.13338	0.00105	0.0207193
NM_001199622.1	135112	NCOA7	4052	36.6319	3.32381	3.46219	0.00105	0.0207193
NM_207303.2	26033	ATRNL1	8732	5.13191	8.98249	-0.807618	0.00105	0.0207193
NM_002338.3	4045	LSAMP	9478	7.39798	12.7356	-0.783666	0.0011	0.0214605
NM_030938.3	81671	VMP1	2176	73.8535	42.9597	0.781681	0.0011	0.0214605
NM_002988.2	6362	CCL18	770	3.92204	0.653166	2.58608	0.0011	0.0214605
NM_033632.2	55294	FBXW7	3896	40.6381	23.5459	0.787354	0.0011	0.0214605
NM_005320.2	3007	HIST1H1D	777	113.451	60.976	0.895762	0.0011	0.0214605
NM_016306.4	51726	DNAJB11	1689	54.5798	31.4398	0.795777	0.0011	0.0214605
NM_145239.2	112476	PRRT2	2567	1.48518	3.78784	-1.35074	0.0011	0.0214605
TCONS_00111412	XLOC_009731	-	2716	4.50001	9.19673	-1.03119	0.0011	0.0214605
NM_003329.2	7295	TXN	508	349.612	206.195	0.76174	0.0011	0.0214605
TCONS_00233526	XLOC_020559	-	4976	6.07835	14.7785	-1.28175	0.0011	0.0214605
NM_004267.3	9435	CHST2	3043	4.15804	1.83738	1.17825	0.00115	0.0222874
TCONS_00314598	XLOC_026580	-	3111	1.35404	0.161749	3.06544	0.00115	0.0222874

NM_001144759.1	23187	PHLDB1	5298	5.39679	9.73775	-0.851486	0.00115	0.0222874
NM_018058.5	55118	CRTAC1	2713	0.293499	2.46175	-3.06826	0.00115	0.0222874
NM_152377.2	127795	C1orf87	2028	16.2069	29.0239	-0.840636	0.00115	0.0222874
TCONS_00293640	XLOC_025525	-	6270	1.99941	4.03428	-1.01274	0.0012	0.0230244
NM_001908.3	1508	CTSB	3783	118.423	61.0981	0.95475	0.0012	0.0230244
NM_002788.3	5684	PSMA3	998	78.6893	45.6207	0.786479	0.0012	0.0230244
NM_007237.4	11262	SP140	3250	10.4627	5.1974	1.00939	0.0012	0.0230244
NM_020672.1	57402	S100A14	1043	27.7038	13.6965	1.01628	0.0012	0.0230244
NM_001199138.1	58484	NLRC4	3360	3.11694	1.20491	1.3712	0.0012	0.0230244
NM_001006605.3	388650	FAM69A	2599	20.0278	11.2551	0.831431	0.0012	0.0230244
NM_199420.3	10721	POLQ	8780	2.15647	4.05829	-0.912203	0.0012	0.0230244
NM_014518.2	7772	ZNF229	4964	1.40975	2.9793	-1.07953	0.0012	0.0230244
TCONS_00217976	XLOC_018953	-	2135	0.574987	1.93847	-1.75332	0.00125	0.02372
NM_000275.2	4948	OCA2	3140	0.665624	1.91235	-1.52257	0.00125	0.02372
NM_000847.4	2940	GSTA3	915	1.26298	4.12801	-1.70862	0.00125	0.02372
NM_001025195.1	1066	CES1	2013	22.0134	56.7756	-1.36689	0.00125	0.02372
NM_001431.3	2037	EPB41L2	4457	20.9575	40.6145	-0.954529	0.00125	0.02372
NM_005306.2	2867	FFAR2	2053	1.82213	0.522918	1.80097	0.00125	0.02372
NM_152309.2	118788	PIK3AP1	4811	19.6488	10.9904	0.838195	0.00125	0.02372
NM_006469.4	10625	IVNS1ABP	4199	87.5578	49.9464	0.809855	0.00125	0.02372
NM_005719.2	10094	ARPC3	884	237.652	137.249	0.792055	0.00125	0.02372
TCONS_00087753	XLOC_007659	-	5536	0.786727	1.82988	-1.21782	0.00125	0.02372
NM_000291.3	5230	PGK1	2423	97.5128	57.1127	0.77178	0.0013	0.0244278
NM_001145206.1	85379	KIAA1671	10494	6.06938	10.728	-0.821756	0.0013	0.0244278
NM_015886.3	51050	PI15	6732	2.41897	4.98907	-1.04438	0.0013	0.0244278
NM_004200.2	9066	SYT7	4532	2.02056	0.831907	1.28026	0.0013	0.0244278
NM_003525.2	8346	HIST1H2BI	437	60.0578	27.0655	1.1499	0.0013	0.0244278
NM_001134363.1	282996	RBM20	7233	2.37751	4.45379	-0.905582	0.0013	0.0244278
NM_006178.3	4905	NSF	3983	31.1925	18.1373	0.782241	0.0013	0.0244278
NM_177937.2	51280	GOLM1	3080	50.4444	20.2156	1.31922	0.0013	0.0244278
NM_005100.3	9590	AKAP12	8434	21.3303	47.4702	-1.15412	0.0013	0.0244278
TCONS_00295272	XLOC_025154	-	2524	0.545096	1.61553	-1.56742	0.0013	0.0244278
NM_003037.2	6504	SLAMF1	2001	6.94837	3.22793	1.10607	0.0013	0.0244278
NM_018897.2	56171	DNAH7	12394	24.3824	44.9611	-0.882837	0.00135	0.0250595
TCONS_00223831	XLOC_018891	-	5712	6.4722	2.34305	1.46587	0.00135	0.0250595
NM_001629.3	241	ALOX5AP	906	27.1364	13.3778	1.0204	0.00135	0.0250595
NM_021240.2	58524	DMRT3	2184	3.33897	7.38812	-1.1458	0.00135	0.0250595
NM_001185.3	563	AZGP1	1265	60.1863	27.3472	1.13804	0.00135	0.0250595
NM_015693.3	27152	INTU	3275	11.515	20.2313	-0.813071	0.00135	0.0250595
NM_152380.2	6913	TBX15	3495	1.1225	2.76104	-1.2985	0.00135	0.0250595
NM_014056.3	25994	HIGD1A	2767	47.9312	28.3027	0.760024	0.00135	0.0250595
NM_001127608.1	9413	FAM189A2	2107	1.1574	4.11849	-1.83123	0.00135	0.0250595
NM_007210.3	11226	GALNT6	4520	6.94078	3.5889	0.951555	0.00135	0.0250595
NM_005022.2	5216	PFN1	807	179.468	106.079	0.758591	0.00135	0.0250595
NM_004289.6	9603	NFE2L3	3711	33.1204	17.8602	0.890971	0.00135	0.0250595
TCONS_00218085	XLOC_019065	-	2851	0.893646	2.29084	-1.3581	0.00135	0.0250595
NM_002705.4	5493	PPL	6238	13.8095	23.0782	-0.740865	0.00135	0.0250595
NM_020873.5	57633	LRRN1	3823	14.0396	7.7762	0.852362	0.00135	0.0250595
NM_003269.3	7101	NR2E1	3259	2.0176	4.65981	-1.20763	0.00135	0.0250595
NM_006290.2	7128	TNFAIP3	4432	26.7074	15.3184	0.801976	0.00135	0.0250595
NM_001013659.2	390927	ZNF793	7121	2.49708	4.58164	-0.87562	0.0014	0.0257815
NM_005931.3	4277	MICB	2483	5.26915	2.34162	1.17006	0.0014	0.0257815
NM_005101.3	9636	ISG15	667	23.4989	9.88108	1.24986	0.0014	0.0257815
NM_002593.3	5118	PCOLCE	1620	30.3273	54.0996	-0.835002	0.0014	0.0257815

NM_178172.3	338328	GPIHBP1	2281	1.95069	4.58965	-1.2344	0.00145	0.0264203
TCONS_00052037	XLOC_004301	-	11166	1.79817	3.33356	-0.890531	0.00145	0.0264203
NM_004944.2	1776	DNASE1L3	1463	10.271	19.5908	-0.931603	0.00145	0.0264203
NM_001276.2	1116	CHI3L1	1837	2.65246	0.86636	1.61429	0.00145	0.0264203
NM_006151.2	4025	LPO	2979	2.05025	0.549473	1.89968	0.0015	0.0270821
NM_016623.3	51571	FAM49B	2196	38.1114	21.7082	0.811981	0.0015	0.0270821
NM_000631.4	4689	NCF4	1398	9.52919	3.12951	1.60641	0.0015	0.0270821
NM_005380.6	4681	NBL1	2026	88.0353	166.887	-0.92272	0.0015	0.0270821
XM_003118889.1	100506859	LOC100506859	703	9.52558	22.1987	-1.2206	0.0015	0.0270821
NM_000405.4	2760	GM2A	3690	33.2595	19.6752	0.757386	0.0015	0.0270821
NM_014767.2	9806	SPOCK2	5329	10.7684	6.15144	0.807813	0.0015	0.0270821
NM_001165257.1	29106	SCG3	3257	1.63918	0.588646	1.4775	0.0015	0.0270821
NM_002443.3	4477	MSMB	494	148.471	69.221	1.1009	0.00155	0.0278206
NM_001128227.2	10020	GNE	5298	18.5581	7.74155	1.26136	0.00155	0.0278206
NM_001877.4	1380	CR2	3993	1.07636	0.365179	1.55948	0.00155	0.0278206
NM_001295.2	1230	CCR1	2676	14.8614	8.05541	0.883537	0.00155	0.0278206
NM_152632.3	170063	CXorf22	3613	16.8435	28.8899	-0.778376	0.00155	0.0278206
TCONS_00092641	XLOC_007763	-	1842	2.33692	5.44236	-1.21963	0.00155	0.0278206
NM_002934.2	6036	RNASE2	735	7.39762	1.89386	1.96573	0.00155	0.0278206
TCONS_00196351	XLOC_017552	-	2737	0.522274	1.508	-1.52976	0.00155	0.0278206
NM_024572.2	79623	GALNT14	2712	4.61289	2.08884	1.14297	0.0016	0.0284826
TCONS_00196551	XLOC_017715	-	1840	0.725464	2.16441	-1.577	0.0016	0.0284826
NM_000730.2	886	CCKAR	1682	0.177543	1.31598	-2.8899	0.0016	0.0284826
NM_017918.4	55013	CCDC109B	1274	27.0918	14.6038	0.891513	0.0016	0.0284826
TCONS_00281560	XLOC_024256	-	1326	0.519996	1.97159	-1.92279	0.0016	0.0284826
NM_138455.2	115908	CTHRC1	1220	10.7615	4.83871	1.15318	0.00165	0.0290957
TCONS_00001771	XLOC_001339	-	1501	0.310214	1.46284	-2.23743	0.00165	0.0290957
TCONS_00187975	XLOC_016196	-	614	0.787259	4.3203	-2.45622	0.00165	0.0290957
NM_032040.3	83987	CCDC8	3334	3.14983	6.2283	-0.983566	0.00165	0.0290957
NM_199478.1	5354	PLP1	2921	4.03874	8.45052	-1.06513	0.00165	0.0290957
TCONS_00049749	XLOC_003979	-	1775	0.399247	1.59804	-2.00095	0.00165	0.0290957
NM_003585.3	8447	DOC2B	1075	5.70845	2.16193	1.40078	0.00165	0.0290957
TCONS_00299616	XLOC_024801	-	2918	0.823028	2.09969	-1.35116	0.00165	0.0290957
NM_002862.3	5834	PYGB	4131	20.6476	11.1422	0.889938	0.00165	0.0290957
NM_020830.3	57590	WDFY1	4607	67.8244	39.6932	0.772915	0.00165	0.0290957
NM_002658.3	5328	PLAU	2378	5.91869	2.54705	1.21645	0.00165	0.0290957
NM_015719.3	50509	COL5A3	6174	1.34373	2.80879	-1.06371	0.0017	0.0297668
NM_032242.3	5361	PLXNA1	9066	4.87442	8.28885	-0.765941	0.0017	0.0297668
NM_001865.3	1347	COX7A2	701	94.5744	54.0966	0.805911	0.0017	0.0297668
NM_001172626.1	270	AMPD1	2395	6.33604	1.01457	2.64271	0.0017	0.0297668
TCONS_00250789	XLOC_021670	-	947	1.11207	3.67855	-1.72589	0.0017	0.0297668
NM_001004470.1	338596	ST8SIA6	1398	5.52223	2.26557	1.28538	0.0017	0.0297668
NM_005073.3	6564	SLC15A1	3127	0.461299	1.34532	-1.54418	0.00175	0.0304286
NM_002207.2	3680	ITGA9	3962	7.75131	13.4873	-0.79909	0.00175	0.0304286
NM_021066.2	8331	HIST1H2AJ	439	28.1016	10.76	1.38497	0.00175	0.0304286
NM_001031746.3	196740	VSTM4	6354	5.42774	9.44469	-0.799151	0.00175	0.0304286
TCONS_00235118	XLOC_019578	-	2185	0.244712	0.95764	-1.9684	0.00175	0.0304286
TCONS_00302479	XLOC_025340	-	1300	0.996047	3.07309	-1.62541	0.00175	0.0304286
NM_030569.6	80760	ITIH5	6749	3.22086	6.35474	-0.980386	0.00175	0.0304286
NM_001135170.1	114905	C1QTNF7	4518	0.324526	2.92089	-3.17	0.00175	0.0304286
NM_004864.2	9518	GDF15	1200	1.76635	0.431044	2.03486	0.0018	0.0310732
NM_001394.6	1846	DUSP4	5621	4.60363	2.17915	1.07901	0.0018	0.0310732
NM_020436.3	57167	SALL4	3486	0.436627	1.19812	-1.4563	0.0018	0.0310732
NM_021021.3	6641	SNTB1	4975	11.7369	19.763	-0.751759	0.0018	0.0310732

NM_001290.3	9079	LDB2	2540	28.1854	54.161	-0.942304	0.0018	0.0310732
NM_172239.4	254958	REXO1L1	7032	13.632	24.4886	-0.845111	0.0018	0.0310732
NM_018284.2	2635	GBP3	3040	55.7273	32.6487	0.77136	0.0018	0.0310732
NM_024164.5	64499	TPSB2	1166	11.7505	5.04708	1.2192	0.0018	0.0310732
NM_001005162.2	340980	OR52B6	1008	0.659352	2.58288	-1.96986	0.00185	0.0317167
NM_001010905.1	352999	C6orf58	1200	0.350801	1.90582	-2.44169	0.00185	0.0317167
NM_003829.3	8777	MPDZ	7438	17.0625	29.0989	-0.770134	0.00185	0.0317167
NM_015668.3	26166	RGS22	4184	16.2235	28.5281	-0.814297	0.00185	0.0317167
TCONS_00133407	XLOC_010538	-	2599	1.78318	4.104	-1.20258	0.00185	0.0317167
NM_006367.3	10487	CAP1	2773	83.5729	44.2391	0.917714	0.00185	0.0317167
NM_001198689.1	3953	LEPR	5100	16.5626	64.7144	-1.96616	0.00185	0.0317167
NM_030782.3	81037	CLPTM1L	2148	35.1958	20.7687	0.760991	0.00185	0.0317167
NM_020747.2	57507	ZNF608	5645	7.90934	13.5527	-0.776951	0.00185	0.0317167
NM_016010.2	51101	FAM164A	3352	17.1619	28.8579	-0.749754	0.00185	0.0317167
NM_001080444.1	150084	IGSF5	2066	0.186116	0.907261	-2.28531	0.00185	0.0317167
NM_002372.2	4124	MAN2A1	5128	30.9774	18.5905	0.736651	0.00185	0.0317167
NM_002350.2	4067	LYN	3092	31.3584	16.6674	0.911827	0.0019	0.0323187
NM_001423.2	2012	EMP1	2804	33.5609	58.9101	-0.811734	0.0019	0.0323187
NM_080387.4	338339	CLEC4D	1936	1.80565	0.536234	1.75159	0.0019	0.0323187
NM_014143.3	29126	CD274	3686	7.42843	3.89331	0.932059	0.0019	0.0323187
NM_000027.3	175	AGA	2103	17.7639	9.54395	0.896288	0.0019	0.0323187
NM_001838.3	1236	CCR7	2185	6.63526	3.03398	1.12894	0.0019	0.0323187
NM_213599.2	203859	ANO5	6661	0.43282	1.7271	-1.99651	0.0019	0.0323187
NM_003243.4	7049	TGFBR3	6467	11.0479	20.7131	-0.906769	0.0019	0.0323187
NM_178140.2	23037	PDZD2	11642	5.40804	9.05298	-0.743288	0.0019	0.0323187
NM_000616.4	920	CD4	3116	14.5046	7.53192	0.945418	0.0019	0.0323187
NM_002649.2	5294	PIK3CG	5379	12.7896	7.36006	0.797184	0.00195	0.0329693
NM_152376.3	127733	UBXN10	2972	28.6263	47.6268	-0.734432	0.00195	0.0329693
NM_014867.2	9920	KBTBD11	6717	1.37335	2.69693	-0.973621	0.00195	0.0329693
NM_025107.2	80177	MYCT1	3045	8.33713	14.5865	-0.80701	0.00195	0.0329693
TCONS_00000463	XLOC_000192	-	4312	1.01879	2.29882	-1.17404	0.00195	0.0329693
NM_004911.4	9601	PDIA4	2952	75.2829	45.242	0.734659	0.00195	0.0329693
NM_152869.2	9104	RGN	2235	0.532413	4.33111	-3.02412	0.00195	0.0329693
NM_006648.3	65268	WNK2	6834	0.648537	1.46654	-1.17715	0.00195	0.0329693
NM_133457.2	136227	EMID2	3005	2.30186	4.94397	-1.10287	0.00195	0.0329693
NM_006033.2	9388	LIPG	4141	0.876609	0.292751	1.58226	0.002	0.0335868
NM_000044.2	367	AR	4314	1.22889	3.04562	-1.30938	0.002	0.0335868
NM_182500.2	130813	C2orf50	1056	2.72954	6.83274	-1.3238	0.002	0.0335868
NM_006082.2	10376	TUBA1B	1753	140.646	84.1687	0.740715	0.002	0.0335868
NM_016274.4	51177	PLEKHO1	1615	25.1083	13.7301	0.870825	0.002	0.0335868
NM_002976.3	6332	SCN7A	7186	1.61744	3.11981	-0.947749	0.002	0.0335868
TCONS_00176190	XLOC_013455	-	1936	0.878254	2.53544	-1.52953	0.002	0.0335868
NM_130847.2	154810	AMOTL1	8999	11.9087	20.0838	-0.754015	0.002	0.0335868
NM_000057.2	641	BLM	4528	3.50817	1.66242	1.07744	0.00205	0.0341961
NM_198474.3	283298	OLFML1	2905	7.86364	13.9819	-0.830293	0.00205	0.0341961
NM_014759.3	9796	PHYHIP	3215	0.412173	1.22527	-1.57177	0.00205	0.0341961
NM_002648.3	5292	PIM1	2709	11.6142	6.26396	0.890749	0.00205	0.0341961
ENST00000538665.1	ENSG00000255733.1	IFNG-AS1	1791	13.2136	6.46886	1.03044	0.00205	0.0341961
NM_003155.2	6781	STC1	3877	10.8552	6.10944	0.829278	0.00205	0.0341961
NM_001031716.2	64859	OBFC2A	3849	37.2567	22.2308	0.744942	0.00205	0.0341961
NM_001136482.1	255809	C19orf38	1089	3.26357	0.9874	1.72474	0.00205	0.0341961
NM_001081438.1	11006	LILRB4	2084	4.17331	0.940981	2.14896	0.00205	0.0341961
NM_014420.2	27121	DKK4	818	2.40412	0.491183	2.29118	0.00205	0.0341961
NM_000297.3	5311	PKD2	5079	15.8803	26.4233	-0.734569	0.0021	0.0348574

NM_004255.3	9377	COX5A	770	44.4846	23.8837	0.897277	0.0021	0.0348574
NM_199280.2	165186	FAM179A	3548	5.7623	10.5229	-0.868811	0.0021	0.0348574
NM_000918.3	5034	P4HB	2578	105.702	63.2556	0.740736	0.0021	0.0348574
NM_014488.3	27314	RAB30	1602	26.1247	14.6455	0.834956	0.0021	0.0348574
NM_006142.3	2810	SFN	1315	13.6089	6.7159	1.01889	0.00215	0.0355296
NM_003257.3	7082	TJP1	7165	12.1953	33.8133	-1.47127	0.00215	0.0355296
NM_173695.2	286464	CXorf59	1956	13.6792	24.0057	-0.811391	0.00215	0.0355296
NM_005222.3	1750	DLX6	1875	0.579731	1.73597	-1.58229	0.00215	0.0355296
XM_003118966.1	100506711	LOC100506711	1898	11.1922	20.2473	-0.855238	0.0022	0.0361165
NM_019885.2	56603	CYP26B1	4528	5.14771	2.61029	0.97972	0.0022	0.0361165
TCONS_00241592	XLOC_020747	-	3066	1.30575	0.236254	2.46647	0.0022	0.0361165
NM_018013.3	55084	SOBP	6232	2.31791	4.33429	-0.90297	0.0022	0.0361165
NM_001098526.1	120425	AMICA1	1957	28.6569	14.4784	0.984977	0.0022	0.0361165
NM_005408.2	6357	CCL13	851	2.65448	0.321955	3.0435	0.0022	0.0361165
NM_001172129.1	3055	HCK	2155	9.49841	4.50093	1.07746	0.0022	0.0361165
NM_013281.3	23767	FLRT3	3830	8.16528	17.3855	-1.09031	0.0022	0.0361165
NM_000088.3	1277	COL1A1	5927	40.5953	144.43	-1.83099	0.0022	0.0361165
NM_000517.4	3040	HBA2	605	3.43494	11.0146	-1.68106	0.0022	0.0361165
NM_173543.2	199221	DZIP1L	3498	5.60001	10.6107	-0.922023	0.00225	0.0367046
NM_017982.3	55061	SUSD4	3027	2.82254	6.00956	-1.09027	0.00225	0.0367046
NM_001198557.1	4001	LMNB1	2250	13.3513	6.30134	1.08324	0.00225	0.0367046
NM_006492.2	257	ALX3	1478	1.75103	4.3006	-1.29634	0.00225	0.0367046
NM_003061.2	6585	SLIT1	7931	0.406315	0.956269	-1.23482	0.00225	0.0367046
NM_001982.3	2065	ERBB3	5757	19.2291	11.6105	0.727865	0.00225	0.0367046
NM_001097589.1	6707	SPRR3	902	3.49682	0.327627	3.41592	0.00225	0.0367046
NM_004355.2	972	CD74	1311	1347.82	519.206	1.37624	0.00225	0.0367046
NM_001111097.1	4067	LYN	3029	19.2899	6.89525	1.48417	0.0023	0.0373663
NM_017481.2	50613	UBQLN3	2345	0.394743	1.23448	-1.64492	0.0023	0.0373663
NM_001128423.1	255027	MPV17L	2309	12.9416	5.37442	1.26784	0.0023	0.0373663
NM_018982.4	54432	YIPF1	1852	19.7609	11.1554	0.824908	0.0023	0.0373663
NM_003537.3	8358	HIST1H3B	472	38.4051	15.5455	1.3048	0.0023	0.0373663
NM_001010000.2	79822	ARHGAP28	5534	3.93763	6.97306	-0.824464	0.0023	0.0373663
NM_001165038.1	2675	GFRA2	3015	1.05812	3.8669	-1.86968	0.0023	0.0373663
NM_006287.4	7035	TFPI	3916	13.3169	23.1363	-0.796906	0.00235	0.0379132
NM_014395.2	27071	DAPP1	2938	35.8757	21.4632	0.741141	0.00235	0.0379132
TCONS_00299625	XLOC_024811	-	4367	0.915888	2.13658	-1.22206	0.00235	0.0379132
NM_001109809.2	346171	ZFP57	1870	1.60841	0.512756	1.64929	0.00235	0.0379132
NM_004170.5	6505	SLC1A1	3739	5.42526	9.68407	-0.835921	0.00235	0.0379132
NM_013340.2	29930	PCDHB1	2534	0.775551	2.04343	-1.3977	0.00235	0.0379132
NM_003096.2	6637	SNRPG	575	83.0615	45.9426	0.854347	0.00235	0.0379132
NM_006432.3	10577	NPC2	897	200.489	122.732	0.708014	0.00235	0.0379132
NM_182701.1	257202	GPX6	1712	0.21105	1.3706	-2.69915	0.00235	0.0379132
ENST00000606242.1	ENSG00000267530.2	AC006273.5	710	2.66517	9.55615	-1.8422	0.0024	0.03848
NM_000619.2	3458	IFNG	1210	5.42209	1.65962	1.70799	0.0024	0.03848
NM_013401.2	5866	RAB3IL1	2284	2.03433	4.49611	-1.14412	0.0024	0.03848
NM_173651.2	401024	FSIP2	21054	2.59354	4.49562	-0.793599	0.0024	0.03848
TCONS_00289951	XLOC_024322	-	3108	1.22366	0.451591	1.43811	0.0024	0.03848
NM_134424.2	5893	RAD52	2673	3.59547	6.82609	-0.924882	0.0024	0.03848
NM_000239.2	4069	LYZ	1516	1996.13	199.578	3.32218	0.0024	0.03848
XM_003403451.1	100508736	LOC100508736	1567	10.1482	19.0839	-0.91113	0.0024	0.03848
NM_178554.4	339855	KY	5704	0.341729	0.912367	-1.41676	0.0024	0.03848
NM_003524.2	8345	HIST1H2BH	425	56.4484	26.7689	1.07637	0.0024	0.03848
NM_153366.3	79987	SVEP1	12344	16.9042	30.1266	-0.833659	0.0024	0.03848
NM_000544.3	6891	TAP2	5679	16.5558	9.32042	0.82887	0.0024	0.03848

NM_198181.2	440295	GOLGA6L9	4375	2.6674	5.05678	-0.922783	0.00245	0.039114
NM_000803.4	2350	FOLR2	1131	29.8546	14.7432	1.01791	0.00245	0.039114
NM_053056.2	595	CCND1	4289	41.7768	68.8619	-0.721003	0.00245	0.039114
TCONS_00268990	XLOC_023119	-	3294	2.03454	0.474153	2.10128	0.00245	0.039114
NM_001004067.3	408050	NOMO3	4316	2.37045	0.990041	1.2596	0.0025	0.0397144
NM_000673.4	131	ADH7	2289	21.6311	37.4903	-0.793411	0.0025	0.0397144
NM_004274.4	9472	AKAP6	10387	7.24343	11.8848	-0.714375	0.0025	0.0397144
NM_015069.2	23090	ZNF423	4818	1.91296	3.76383	-0.976397	0.0025	0.0397144
NM_001781.2	969	CD69	1676	25.6891	14.1771	0.857589	0.0025	0.0397144
NM_001093.3	32	ACACB	9250	4.4828	7.509	-0.744221	0.0025	0.0397144
NM_018102.3	55713	ZNF334	2455	3.90401	8.65895	-1.14923	0.0025	0.0397144
NM_001173463.1	55605	KIF21A	6530	29.0202	50.1285	-0.788572	0.0025	0.0397144
NM_000929.2	5322	PLA2G5	1894	0.413318	1.32441	-1.68003	0.0025	0.0397144
NM_199289.1	341676	NEK5	2912	13.2112	22.3474	-0.758343	0.0025	0.0397144
NM_021074.4	4729	NDUFV2	930	109.097	65.9683	0.725769	0.0025	0.0397144
NM_005412.5	6472	SHMT2	2295	12.9131	6.64642	0.958181	0.00255	0.0402995
NM_005912.2	4160	MC4R	1438	2.38977	0.763864	1.64549	0.00255	0.0402995
NM_001099221.1	497189	TIFAB	1273	2.10709	0.659298	1.67625	0.00255	0.0402995
NM_005615.4	6039	RNASE6	1061	34.2103	18.8929	0.856587	0.00255	0.0402995
NM_005688.2	10057	ABCC5	5845	4.82494	9.1921	-0.929885	0.00255	0.0402995
3500	3500	-	992	8237.46	1088.54	2.91981	0.00255	0.0402995
NM_015692.2	27151	CPAMD8	5988	0.487301	1.19218	-1.29072	0.00255	0.0402995
NM_173505.2	147463	ANKRD29	2274	3.46836	7.15142	-1.04397	0.00255	0.0402995
NM_020203.3	56955	MEPE	2007	0.568371	1.66713	-1.55246	0.0026	0.040888
XM_001715897.2	408186	OVOS	4119	1.20382	0.392361	1.61737	0.0026	0.040888
NM_001039396.1	219972	MPEG1	4512	48.9505	25.394	0.946836	0.0026	0.040888
NM_005443.4	9061	PAPSS1	2542	76.18	45.9811	0.72837	0.00265	0.0415288
NM_152663.3	55103	RALGPS2	5834	23.9994	12.3326	0.960517	0.00265	0.0415288
NM_001142699.1	1740	DLG2	8046	0.822409	2.50481	-1.60678	0.0027	0.0421065
NM_032888.2	85301	COL27A1	5583	1.2692	2.58742	-1.02759	0.0027	0.0421065
NM_024761.4	79817	MOBKL2B	6525	2.29394	4.32309	-0.914236	0.0027	0.0421065
NM_004433.4	1999	ELF3	3134	60.3889	36.3806	0.731113	0.0027	0.0421065
NM_005290.1	2838	GPR15	1083	2.71639	0.753787	1.84946	0.0027	0.0421065
TCONS_00046009	XLOC_004001	-	817	0.418225	2.37186	-2.50367	0.0027	0.0421065
NM_016529.4	51761	ATP8A2	5006	1.86093	0.805169	1.20866	0.0027	0.0421065
NM_014618.2	1620	DBC1	3196	3.7226	1.64308	1.17991	0.0027	0.0421065
NM_020762.2	57522	SRGAP1	8475	2.59334	4.45138	-0.779443	0.0027	0.0421065
NM_032131.4	84071	ARMC2	3413	11.2294	18.9244	-0.75297	0.00275	0.0426687
NM_178011.3	347731	LRRTM3	5609	0.305832	1.03505	-1.75888	0.00275	0.0426687
NM_000417.2	3559	IL2RA	3216	3.33699	1.52618	1.12862	0.00275	0.0426687
NM_031898.2	64518	TEKT3	1784	1.5255	3.70959	-1.28197	0.00275	0.0426687
NM_001145664.1	731220	RFX8	1729	0.187325	0.929495	-2.3109	0.00275	0.0426687
NM_001127391.1	130540	ALS2CR12	1944	8.60666	16.0725	-0.901067	0.00275	0.0426687
NM_017839.4	54947	LPCAT2	5388	10.5208	6.29546	0.740861	0.00275	0.0426687
NM_003446.3	7712	ZNF157	1695	2.12303	4.81562	-1.1816	0.00275	0.0426687
NM_003518.3	8339	HIST1H2BG	445	64.081	31.6684	1.01685	0.00275	0.0426687
NM_024943.1	80008	TMEM156	1878	7.29762	3.41753	1.09447	0.00275	0.0426687
NM_006931.2	6515	SLC2A3	3938	11.5063	6.6203	0.797461	0.00275	0.0426687
NM_145172.3	126820	WDR63	3007	18.9917	31.7857	-0.743008	0.0028	0.0432452
NM_006135.2	829	CAPZA1	2740	111.067	67.533	0.71776	0.0028	0.0432452
NM_012242.2	22943	DKK1	1805	1.56218	0.473831	1.72112	0.0028	0.0432452
NM_006714.3	10924	SMPDL3A	2195	12.9641	7.11525	0.865535	0.0028	0.0432452
NM_004961.3	2564	GABRE	3152	3.25493	6.26439	-0.944549	0.0028	0.0432452
NM_175862.4	942	CD86	2727	12.8441	6.90471	0.895448	0.0028	0.0432452

TCONS_00103444	XLOC_008124	-	1958	40.7763	23.9274	0.769064	0.0028	0.0432452
NM_021058.3	8970	HIST1H2BJ	481	54.2158	26.7473	1.01932	0.0028	0.0432452
NM_003742.2	8647	ABCB11	4775	3.21867	1.57584	1.03035	0.0028	0.0432452
NM_005779.2	10184	LHFPL2	5045	17.6872	28.5613	-0.691352	0.00285	0.0437663
NM_006516.2	6513	SLC2A1	3670	14.7054	8.73311	0.751778	0.00285	0.0437663
NM_001628.2	231	AKR1B1	1398	18.0487	31.7768	-0.816074	0.00285	0.0437663
NM_001029864.1	85449	KIAA1755	6412	0.801336	1.75274	-1.12913	0.00285	0.0437663
NM_000201.2	3383	ICAM1	3246	11.6094	6.61486	0.811511	0.00285	0.0437663
NM_000434.3	4758	NEU1	2071	19.0537	11.0421	0.787055	0.00285	0.0437663
NM_001025266.1	285382	C3orf70	5901	4.33434	2.2555	0.942364	0.0029	0.0443924
NM_194302.2	255101	CCDC108	5945	2.83059	5.20581	-0.879021	0.0029	0.0443924
NM_002652.2	5304	PIP	576	16.7682	4.27924	1.9703	0.0029	0.0443924
NM_017594.3	54769	DIRAS2	4107	0.322685	0.902626	-1.484	0.0029	0.0443924
NM_013378.2	29802	VPREB3	585	4.86699	1.30176	1.90257	0.00295	0.0449329
NM_145912.5	150372	NFAM1	5605	2.30641	1.12952	1.02994	0.00295	0.0449329
NM_015000.3	23012	STK38L	5098	28.6834	17.5419	0.709413	0.00295	0.0449329
NM_018323.3	55300	PI4K2B	3601	22.2284	13.3654	0.733905	0.00295	0.0449329
TCONS_00278332	XLOC_023384	-	1144	8.13869	2.72486	1.57861	0.00295	0.0449329
NM_145018.3	220042	C11orf82	3565	2.00233	0.856252	1.22557	0.00295	0.0449329
NM_015595.3	26084	ARHGEF26	5181	3.82647	6.77951	-0.825165	0.00295	0.0449329
NM_032160.2	92126	DSEL	9281	3.8337	6.57699	-0.77869	0.00295	0.0449329
NM_001677.3	481	ATP1B1	2200	163.207	92.0368	0.826418	0.003	0.0454886
NM_178504.4	201625	DNAH12	9542	25.6993	45.6945	-0.830292	0.003	0.0454886
NM_001557.3	3579	CXCR2	2870	3.47956	1.20187	1.53362	0.003	0.0454886
NM_001085447.1	129881	C2orf77	2169	12.3097	21.6062	-0.811648	0.003	0.0454886
NM_001136219.1	2212	FCGR2A	2429	17.1264	8.22393	1.05832	0.003	0.0454886
NM_178537.4	338707	B4GALNT4	3454	0.362156	1.03149	-1.51004	0.003	0.0454886
NM_001136018.2	2052	EPHX1	1664	35.6195	70.088	-0.976501	0.003	0.0454886
NM_019856.1	8736	MYOM1	5559	1.64154	3.51762	-1.09955	0.003	0.0454886
NM_001882.3	1393	CRHBP	1838	0.779504	2.22215	-1.51133	0.003	0.0454886
NM_001012455.1	222696	ZSCAN23	3161	1.60192	3.46756	-1.11412	0.00305	0.04606
NM_024989.3	80055	PGAP1	11113	7.09943	11.5644	-0.703916	0.00305	0.04606
NM_019844.2	28234	SLCO1B3	2712	6.05366	2.54797	1.24846	0.00305	0.04606
NM_001202553.1	8190	MIA	510	17.3703	2.19027	2.98744	0.00305	0.04606
TCONS_00106634	XLOC_008067	-	5143	2.18073	5.32095	-1.28687	0.00305	0.04606
XM_003118626.2	100506209	LOC100506209	3519	2.3406	4.65572	-0.992128	0.00305	0.04606
TCONS_00246975	XLOC_020777	-	1222	1.4077	0.238184	2.5632	0.0031	0.0465955
NM_001142807.1	55289	ACOXL	2373	0.829605	0.19711	2.07342	0.0031	0.0465955
NM_153267.4	256691	MAMDC2	3618	5.48322	10.0174	-0.869417	0.0031	0.0465955
NM_016644.1	51334	PRR16	1764	1.35465	0.422426	1.68115	0.0031	0.0465955
NM_024783.3	79841	AGBL2	3593	9.67783	16.3753	-0.758768	0.0031	0.0465955
NM_004878.4	9536	PTGES	1787	5.85614	2.68954	1.12259	0.0031	0.0465955
TCONS_00153526	XLOC_013969	-	3500	0.999016	2.24264	-1.16662	0.0031	0.0465955
NM_015570.2	26053	AUTS2	6418	6.97612	12.1044	-0.795032	0.0031	0.0465955
NM_003877.3	8835	SOCS2	2210	12.0339	20.717	-0.78371	0.00315	0.0471261
NM_058164.2	93145	OLFM2	1861	2.53232	5.69758	-1.16989	0.00315	0.0471261
NM_032385.3	10826	C5orf4	2997	11.8357	20.1378	-0.766755	0.00315	0.0471261
NM_148672.2	56477	CCL28	816	35.2447	18.3912	0.938388	0.00315	0.0471261
TCONS_00268841	XLOC_022907	-	1765	0.570237	1.6536	-1.53598	0.00315	0.0471261
NM_001956.3	1907	EDN2	1243	1.50871	0.30132	2.32395	0.00315	0.0471261
NM_004180.2	10010	TANK	2089	29.274	10.3237	1.50365	0.0032	0.0476939
NM_001866.2	1349	COX7B	456	289.188	176.668	0.710963	0.0032	0.0476939
NM_030913.4	10500	SEMA6C	3928	0.498911	1.28101	-1.36043	0.0032	0.0476939
TCONS_00234687	XLOC_020386	-	870	2.02238	5.5224	-1.44924	0.0032	0.0476939

TCONS_00012726	XLOC_001573	-	3939	0.407029	2.16769	-2.41295	0.0032	0.0476939
NM_000681.3	150	ADRA2A	3876	14.3173	8.47532	0.756424	0.00325	0.0483534
TCONS_00000222	XLOC_000045	-	2791	3.28476	7.08412	-1.1088	0.00325	0.0483534
NM_178568.2	146760	RTN4RL1	3164	0.922589	2.17195	-1.23523	0.00325	0.0483534
NM_004335.2	684	BST2	939	115.619	70.7397	0.708783	0.0033	0.0489028
NM_005725.4	10100	TSPAN2	3213	7.44976	4.01568	0.891549	0.0033	0.0489028
NM_206963.1	5918	RARRES1	1526	300.035	142.639	1.07276	0.0033	0.0489028
NM_007293.2	720	C4A	5426	2.63952	1.28264	1.04116	0.0033	0.0489028
NM_006770.3	8685	MARCO	1835	7.27374	2.55838	1.50747	0.0033	0.0489028
NM_003816.2	8754	ADAM9	4099	67.2931	41.2413	0.70637	0.0033	0.0489028
NM_005516.5	3133	HLA-E	2662	471.508	238.229	0.98493	0.00335	0.0494369
NM_021243.2	58527	C6orf115	845	34.1836	18.4048	0.893219	0.00335	0.0494369
NM_006763.2	7832	BTG2	2712	76.3341	46.769	0.706773	0.00335	0.0494369
XM_003403544.1	100652762	LOC100652762	1905	3.09247	6.39555	-1.04831	0.00335	0.0494369
NM_002507.3	4804	NGFR	3420	3.45483	6.53739	-0.920102	0.00335	0.0494369

Table S5. Differentially expressed lncRNA and mRNA transcripts for ECRSwNP *vs* non-ECRSwNP.

Transcript ID	Gene ID	Gene name	Length	ECRSwNP FPKM	non-ECRSwNP FPKM	log_2(foldchange)	P value	Q value
NM_025074.6	80144	FRAS1	15624	4.40124	1.70678	1.36663	5.00E-05	0.00170056
NM_003670.2	8553	BHLHE40	3035	98.1131	31.6047	1.63431	5.00E-05	0.00170056
NM_001454.3	2302	FOXJ1	2641	19.355	47.8944	-1.30715	5.00E-05	0.00170056
NM_031476.3	83716	CRISPLD2	4589	32.2142	15.7879	1.02888	5.00E-05	0.00170056
NM_000789.3	1636	ACE	4969	6.38162	2.61739	1.28579	5.00E-05	0.00170056
NM_016206.2	389136	VGLL3	10396	20.62	8.70453	1.2442	5.00E-05	0.00170056
NM_000527.4	3949	LDLR	5284	15.7748	4.48996	1.81284	5.00E-05	0.00170056
NM_130435.3	5791	PTPRE	5039	19.0631	8.79356	1.11626	5.00E-05	0.00170056
NM_018897.2	56171	DNAH7	12394	9.65151	24.3824	-1.33701	5.00E-05	0.00170056
NM_003407.2	7538	ZFP36	1745	45.3106	6.55648	2.78886	5.00E-05	0.00170056
NM_018043.5	55107	ANO1	4807	11.5602	5.25339	1.13784	5.00E-05	0.00170056
NM_178171.4	284110	GSDMA	2164	2.54552	0.387037	2.71741	5.00E-05	0.00170056
NM_033124.4	85478	CCDC65	1815	11.1135	28.5293	-1.36014	5.00E-05	0.00170056
NM_001010855.2	146850	PIK3R6	3040	4.40492	0.879517	2.32433	5.00E-05	0.00170056
NM_033364.3	89876	C3orf15	4433	4.77248	15.9909	-1.74444	5.00E-05	0.00170056
NM_001422.2	2001	ELF5	2302	22.3835	8.69459	1.36425	5.00E-05	0.00170056
NM_017596.2	23046	KIF21B	9895	5.5784	2.21288	1.33393	5.00E-05	0.00170056
NM_145172.3	126820	WDR63	3007	6.83313	18.9917	-1.47475	5.00E-05	0.00170056
NM_001945.2	1839	HBEGF	2358	20.1238	2.34266	3.10269	5.00E-05	0.00170056
NM_004982.2	3764	KCNJ8	2381	14.0334	4.97994	1.49466	5.00E-05	0.00170056
NM_030919.2	81610	FAM83D	2429	7.68453	2.951	1.38076	5.00E-05	0.00170056
NM_001042625.1	133690	CAPSL	972	25.9003	64.6824	-1.3204	5.00E-05	0.00170056
NM_001073.1	10720	UGT2B11	1722	5.06682	0.416719	3.60394	5.00E-05	0.00170056
NM_001063.3	7018	TF	2791	1.42997	44.1466	-4.94824	5.00E-05	0.00170056
TCONS_00012088	XLOC_000773	-	793	78.1617	268.729	-1.78162	5.00E-05	0.00170056
NM_145263.2	132671	SPATA18	4300	14.9987	33.033	-1.13907	5.00E-05	0.00170056
NM_002467.4	4609	MYC	2366	27.3251	8.93989	1.6119	5.00E-05	0.00170056
NM_006418.4	10562	OLFM4	2919	0.254717	1.81549	-2.8334	5.00E-05	0.00170056
NM_001080424.1	23135	KDM6B	6704	3.96764	1.35306	1.55206	5.00E-05	0.00170056
NM_153235.3	167838	TXLNB	4685	2.56226	7.29209	-1.50892	5.00E-05	0.00170056
NM_002122.3	3117	HLA-DQA1	1542	94.8367	28.9265	1.71305	5.00E-05	0.00170056
NM_004770.2	9312	KCNB2	3582	0.252283	2.1343	-3.08065	5.00E-05	0.00170056
NM_033423.3	2999	GZMH	902	3.91429	18.3289	-2.2273	5.00E-05	0.00170056
NM_018076.2	55130	ARMC4	3572	9.8742	29.713	-1.58936	5.00E-05	0.00170056
NM_004425.3	1893	ECM1	2144	11.4724	4.21046	1.44612	5.00E-05	0.00170056
NM_005304.3	2865	FFAR3	1674	8.77307	0.426888	4.36115	5.00E-05	0.00170056
NM_153236.3	168537	GIMAP7	1229	27.8463	56.8578	-1.02987	5.00E-05	0.00170056
NM_005384.2	4783	NFIL3	2085	59.6873	13.4964	2.14485	5.00E-05	0.00170056
XM_003118966.1	100506711	LOC100506711	1898	4.06174	11.1922	-1.46232	5.00E-05	0.00170056
NM_198492.3	339390	CLEC4G	1360	7.34315	0.666522	3.46167	5.00E-05	0.00170056
NM_001079910.1	84125	LRRIQ1	5444	13.4672	38.2467	-1.50589	5.00E-05	0.00170056
NM_002988.2	6362	CCL18	770	279.754	3.92204	6.15641	5.00E-05	0.00170056
NM_182904.3	283208	P4HA3	2269	0.895331	3.45677	-1.94893	5.00E-05	0.00170056
NM_001030060.2	389432	SAMD5	6324	12.1427	5.75933	1.07612	5.00E-05	0.00170056
NM_178135.3	345275	HSD17B13	2395	13.96	1.33494	3.38645	5.00E-05	0.00170056
NM_005708.3	10082	GPC6	7103	2.27758	7.78379	-1.77297	5.00E-05	0.00170056
NM_024867.3	79925	SPEF2	5638	5.444	14.8329	-1.44606	5.00E-05	0.00170056
NM_001385.2	1807	DPYS	2123	22.548	2.31227	3.28562	5.00E-05	0.00170056
NM_181711.2	160622	GRASP	1932	8.23946	2.6652	1.6283	5.00E-05	0.00170056
NM_000619.2	3458	IFNG	1210	1.22678	5.42209	-2.14397	5.00E-05	0.00170056
NM_000139.4	2206	MS4A2	3648	27.5668	12.6324	1.1258	5.00E-05	0.00170056

NM_006952.3	7348	UPK1B	2058	22.9899	9.55285	1.267	5.00E-05	0.00170056
NM_178504.4	201625	DNAH12	9542	12.8389	25.6993	-1.0012	5.00E-05	0.00170056
NM_014466.2	27285	TEKT2	1509	5.07481	14.8239	-1.5465	5.00E-05	0.00170056
NM_005627.3	6446	SGK1	2407	134.757	52.5569	1.3584	5.00E-05	0.00170056
NM_203347.1	389812	LCN15	762	125.98	0	inf	5.00E-05	0.00170056
NM_173494.1	139212	CXorf41	1652	3.14892	11.7898	-1.90462	5.00E-05	0.00170056
NM_002581.3	5069	PAPPA	10970	32.4343	8.36582	1.95494	5.00E-05	0.00170056
NM_024807.2	79865	TREML2	3758	2.55859	0.848817	1.59182	5.00E-05	0.00170056
NM_178827.4	154865	IQUB	3115	8.04017	20.2699	-1.33404	5.00E-05	0.00170056
NM_031956.2	83894	TTC29	1786	8.44536	24.6804	-1.54713	5.00E-05	0.00170056
NM_003014.3	6424	SFRP4	2974	7.14851	25.1784	-1.81647	5.00E-05	0.00170056
NM_012144.2	27019	DNAI1	2521	7.08097	18.7593	-1.40558	5.00E-05	0.00170056
NM_001040429.2	27253	PCDH17	8009	6.79971	2.85158	1.25371	5.00E-05	0.00170056
NM_020056.4	3118	HLA-DQA2	1152	12.8987	82.7047	-2.68074	5.00E-05	0.00170056
NM_001242480.1	389831	LOC389831	2413	19.4036	36.5778	-0.914641	5.00E-05	0.00170056
NM_021822.3	60489	APOBEC3G	1834	12.9428	26.6973	-1.04454	5.00E-05	0.00170056
NM_138295.3	168507	PKD1L1	9075	0.880432	0.267842	1.71683	5.00E-05	0.00170056
NM_018725.3	55540	IL17RB	2040	9.24144	2.84882	1.69775	5.00E-05	0.00170056
NM_000624.4	5104	SERPINA5	2312	2.31715	0.420878	2.46087	5.00E-05	0.00170056
NM_001077710.2	642273	FAM110C	3963	10.7553	3.19997	1.74892	5.00E-05	0.00170056
NM_004833.1	9447	AIM2	1484	3.5985	10.6108	-1.56007	5.00E-05	0.00170056
NM_000491.3	713	C1QB	1044	61.5335	159.757	-1.37643	5.00E-05	0.00170056
NM_001045.4	6532	SLC6A4	4535	1.48573	0.287194	2.37107	5.00E-05	0.00170056
NM_001114753.1	2022	ENG	3060	14.5795	6.1504	1.24519	5.00E-05	0.00170056
NM_178456.2	128602	C20orf85	760	39.5108	128.827	-1.70512	5.00E-05	0.00170056
NM_033102.2	85414	SLC45A3	3382	4.70164	1.60382	1.55165	5.00E-05	0.00170056
NM_012244.2	23428	SLC7A8	4216	13.9423	4.97323	1.48721	5.00E-05	0.00170056
NM_006424.2	10568	SLC34A2	4153	1.48279	12.6765	-3.09577	5.00E-05	0.00170056
NM_130852.2	51297	PLUNC	1078	220.459	1401.58	-2.66847	5.00E-05	0.00170056
NM_000673.4	131	ADH7	2289	76.0395	21.6311	1.81364	5.00E-05	0.00170056
NM_001005337.2	5317	PKP1	5384	6.19607	2.35289	1.39692	5.00E-05	0.00170056
NM_000561.3	2944	GSTM1	1185	0	6.21685	#NAME?	5.00E-05	0.00170056
NM_001100159.1	136288	C7orf57	2125	3.93158	10.6917	-1.44331	5.00E-05	0.00170056
NM_145047.4	127700	OSCP1	1489	11.4481	29.013	-1.3416	5.00E-05	0.00170056
NM_022454.3	64321	SOX17	2350	6.34451	2.03709	1.639	5.00E-05	0.00170056
NM_000593.5	6890	TAP1	2952	21.0098	51.1937	-1.28491	5.00E-05	0.00170056
NM_144715.3	151651	EFHB	2857	8.40831	21.6835	-1.36671	5.00E-05	0.00170056
NM_013272.3	28232	SLCO3A1	5106	7.86589	3.4666	1.18209	5.00E-05	0.00170056
NM_002965.3	6280	S100A9	577	31.6324	86.7818	-1.45599	5.00E-05	0.00170056
NM_001010940.1	138255	C9orf135	851	11.6641	33.2104	-1.50956	5.00E-05	0.00170056
NM_178821.1	164781	WDR69	1669	9.13292	27.0595	-1.56699	5.00E-05	0.00170056
NM_005529.5	3339	HSPG2	14288	19.8768	7.81614	1.34655	5.00E-05	0.00170056
NM_000587.2	730	C7	4012	54.5242	21.6512	1.33245	5.00E-05	0.00170056
NM_001064.3	7086	TKT	2164	48.736	24.1797	1.01119	5.00E-05	0.00170056
NM_012443.2	9576	SPAG6	2594	16.7179	40.3973	-1.27287	5.00E-05	0.00170056
NM_006398.3	10537	UBD	990	17.7337	152.781	-3.1069	5.00E-05	0.00170056
NM_004585.3	5920	RARRES3	765	40.8208	118.171	-1.53349	5.00E-05	0.00170056
NM_138636.4	51311	TLR8	4216	4.28323	9.59225	-1.16317	5.00E-05	0.00170056
NM_001109.4	101	ADAM8	3301	12.3708	3.4303	1.85053	5.00E-05	0.00170056
NM_001030287.2	467	ATF3	1899	45.0643	7.01839	2.68277	5.00E-05	0.00170056
NM_002659.3	5329	PLAUR	1553	17.4066	2.68152	2.69851	5.00E-05	0.00170056
NM_024832.3	79890	RIN3	3849	4.9286	1.68463	1.54874	5.00E-05	0.00170056
NM_004460.2	2191	FAP	2780	19.0462	5.92264	1.68519	5.00E-05	0.00170056
NM_152376.3	127733	UBXN10	2972	12.5561	28.6263	-1.18895	5.00E-05	0.00170056

NM_145054.4	146845	WDR16	2193	5.82124	20.0533	-1.78444	5.00E-05	0.00170056
NM_001127380.2	6289	SAA2	2043	2.72395	45.6897	-4.0681	5.00E-05	0.00170056
NM_002229.2	3726	JUNB	1816	148.001	27.4683	2.42976	5.00E-05	0.00170056
NM_001679.2	483	ATP1B3	1853	62.2088	31.0433	1.00284	5.00E-05	0.00170056
NM_033027.3	64651	CSRNP1	3188	35.6808	5.05652	2.81893	5.00E-05	0.00170056
NM_015474.3	25939	SAMHD1	3189	62.4625	119.501	-0.935957	5.00E-05	0.00170056
TCONS_00003383	XLOC_000243	-	4161	0.938795	3.65575	-1.96129	5.00E-05	0.00170056
NM_002825.5	5764	PTN	1549	15.0362	33.1337	-1.13986	5.00E-05	0.00170056
NM_003154.2	6779	STATH	600	25.9122	229.559	-3.14717	5.00E-05	0.00170056
TCONS_00000172	XLOC_000042	-	1923	0.669346	4.18223	-2.64345	5.00E-05	0.00170056
NM_002164.5	3620	IDO1	1931	11.7424	102.462	-3.12529	5.00E-05	0.00170056
NM_018689.1	57214	KIAA1199	7080	8.91771	3.09974	1.52453	5.00E-05	0.00170056
NM_032571.3	84658	EMR3	2301	15.9376	0.692458	4.52456	5.00E-05	0.00170056
NM_052941.4	115361	GBP4	6145	12.4544	47.9685	-1.94543	5.00E-05	0.00170056
NM_001911.2	1511	CTSG	924	46.5036	19.4127	1.26034	5.00E-05	0.00170056
NM_004352.3	869	CBLN1	2435	0.296986	3.4078	-3.52037	5.00E-05	0.00170056
NM_002125.3	3127	HLA-DRB5	1171	5.43834	18.4272	-1.7606	5.00E-05	0.00170056
NM_032211.6	84171	LOXL4	3657	25.2029	8.10854	1.63608	5.00E-05	0.00170056
NM_001010860.1	161394	SAMD15	2025	5.89604	17.4678	-1.56688	5.00E-05	0.00170056
NM_145252.2	124220	ZG16B	828	13.1537	142.959	-3.44207	5.00E-05	0.00170056
NM_025184.3	80258	EFHC2	3252	5.64227	12.8029	-1.18212	5.00E-05	0.00170056
NM_015675.3	4616	GADD45B	1375	52.339	22.245	1.23441	5.00E-05	0.00170056
NM_001554.4	3491	CYR61	2295	110.679	33.3372	1.73118	5.00E-05	0.00170056
NM_012397.3	5275	SERPINB13	3180	17.1446	4.67778	1.87386	5.00E-05	0.00170056
NM_001122679.1	57451	ODZ2	9645	2.22314	0.666911	1.73703	5.00E-05	0.00170056
NM_001423.2	2012	EMP1	2804	79.8619	33.5609	1.25073	5.00E-05	0.00170056
NM_004508.2	3422	IDI1	2137	65.5286	34.0123	0.946069	5.00E-05	0.00170056
NM_005215.3	1630	DCC	10210	0.681179	2.03395	-1.57818	5.00E-05	0.00170056
NM_002115.2	3101	HK3	3068	15.1287	2.26953	2.73682	5.00E-05	0.00170056
TCONS_00165587	XLOC_013757	-	437	2.9392	0	inf	5.00E-05	0.00170056
NM_004363.2	1048	CEACAM5	3600	41.1753	12.0636	1.77112	5.00E-05	0.00170056
NM_000441.1	5172	SLC26A4	4930	2.74147	23.743	-3.11448	5.00E-05	0.00170056
NM_006691.3	10894	LYVE1	2500	44.01	17.5119	1.32949	5.00E-05	0.00170056
NM_178813.5	158798	AKAP14	841	6.71294	25.4297	-1.9215	5.00E-05	0.00170056
NM_002977.3	6335	SCN9A	9762	4.71871	1.92345	1.29469	5.00E-05	0.00170056
TCONS_00032728	XLOC_003418	-	2076	4.3234	24.8047	-2.52037	5.00E-05	0.00170056
NM_003955.3	9021	SOCS3	2734	117.977	15.2486	2.95176	5.00E-05	0.00170056
NM_020361.4	57094	CPA6	1904	2.69584	0.339249	2.99032	5.00E-05	0.00170056
NM_017855.3	54959	ODAM	1288	54.2032	11.1002	2.28779	5.00E-05	0.00170056
TCONS_00084037	XLOC_006761	-	5365	0.460879	1.77578	-1.946	5.00E-05	0.00170056
NM_145020.3	220136	CCDC11	1824	6.70235	24.2448	-1.85494	5.00E-05	0.00170056
TCONS_00182914	XLOC_015690	-	3636	1.51196	0.327743	2.20578	5.00E-05	0.00170056
NM_004335.2	684	BST2	939	52.2967	115.619	-1.14458	5.00E-05	0.00170056
NM_152687.2	202309	GAPT	2216	28.9959	9.6277	1.59059	5.00E-05	0.00170056
NM_004907.2	9592	IER2	2050	29.3684	8.68455	1.75774	5.00E-05	0.00170056
NM_000358.2	7045	TGFBI	2805	82.931	35.0993	1.24047	5.00E-05	0.00170056
NM_006417.4	10561	IFI44	1726	35.34	70.0541	-0.987166	5.00E-05	0.00170056
NM_004994.2	4318	MMP9	2336	0.916865	4.45143	-2.27949	5.00E-05	0.00170056
NM_152891.2	260429	PRSS33	1694	3.18508	0.190408	4.06416	5.00E-05	0.00170056
NM_001870.2	1359	CPA3	1686	264.984	74.1552	1.83729	5.00E-05	0.00170056
NM_053285.1	83659	TEKT1	1413	20.1326	56.4848	-1.48833	5.00E-05	0.00170056
NM_000930.3	5327	PLAT	3173	5.84999	13.9735	-1.25619	5.00E-05	0.00170056
NM_002123.3	3119	HLA-DQB1	1224	89.406	16.6794	2.42231	5.00E-05	0.00170056
NM_152754.2	223117	SEMA3D	6261	6.78436	29.9333	-2.14147	5.00E-05	0.00170056

NM_153264.5	256076	COL6A5	8878	6.48335	0.114455	5.82389	5.00E-05	0.00170056
NM_005564.3	3934	LCN2	822	153.325	603.048	-1.97568	5.00E-05	0.00170056
NM_000600.3	3569	IL6	1184	13.8138	0.694924	4.31312	5.00E-05	0.00170056
NM_199347.2	152110	NEK10	2639	11.1674	30.0566	-1.42838	5.00E-05	0.00170056
NM_001130958.1	2172	FABP6	743	7.31356	28.9934	-1.98708	5.00E-05	0.00170056
NM_018950.2	3134	HLA-F	1284	38.2524	91.3257	-1.25547	5.00E-05	0.00170056
NM_025052.3	80122	YSK4	4165	7.89697	20.0636	-1.34521	5.00E-05	0.00170056
NM_001005473.2	345557	PLCXD3	7739	0.342657	1.50795	-2.13775	5.00E-05	0.00170056
NM_144701.2	149233	IL23R	2826	0.338423	1.52114	-2.16826	5.00E-05	0.00170056
NM_052901.2	114789	SLC25A25	3240	21.564	8.5941	1.32721	5.00E-05	0.00170056
TCONS_00037389	XLOC_003418	-	566	11.1642	95.7409	-3.10026	5.00E-05	0.00170056
TCONS_00037388	XLOC_003418	-	788	10.2059	88.8672	-3.12225	5.00E-05	0.00170056
NM_152750.4	222256	CDHR3	6500	11.7392	27.2359	-1.21418	5.00E-05	0.00170056
NM_033397.2	85450	ITPRIP	4364	10.4546	2.73862	1.93261	5.00E-05	0.00170056
NM_004469.4	2277	FIGF	2074	0.477196	2.78932	-2.54726	5.00E-05	0.00170056
NM_001008723.1	159686	CCDC147	3313	3.64312	10.3805	-1.51063	5.00E-05	0.00170056
NM_020689.3	57419	SLC24A3	3889	3.34955	1.15336	1.53813	5.00E-05	0.00170056
NM_004223.4	9246	UBE2L6	1261	31.5058	70.8983	-1.17014	5.00E-05	0.00170056
NM_002994.3	6374	CXCL5	2458	0.174158	3.02738	-4.1196	5.00E-05	0.00170056
XM_003403441.1	401258	RAB44	2557	4.65397	0.242598	4.26182	5.00E-05	0.00170056
NM_182920.1	56999	ADAMTS9	7313	20.4174	6.44703	1.66309	5.00E-05	0.00170056
NM_152997.2	260436	C4orf7	539	100.371	600.743	-2.58141	5.00E-05	0.00170056
NM_145038.2	92749	CCDC164	2491	12.9269	36.7338	-1.50674	5.00E-05	0.00170056
NM_001805.2	1053	CEBPE	1201	8.15414	0.463621	4.13652	5.00E-05	0.00170056
NM_019844.2	28234	SLCO1B3	2712	1.38932	6.05366	-2.12343	5.00E-05	0.00170056
NM_014080.4	50506	DUOX2	6346	0.811028	3.22798	-1.99281	5.00E-05	0.00170056
NM_174892.2	124599	CD300LB	2294	5.65704	0.978126	2.53195	5.00E-05	0.00170056
NM_004049.3	597	BCL2A1	887	63.1543	14.7406	2.09909	5.00E-05	0.00170056
NM_001145848.1	8842	PROM1	3973	41.6027	106.641	-1.35801	5.00E-05	0.00170056
NM_002167.3	3399	ID3	1288	52.1784	21.4764	1.2807	5.00E-05	0.00170056
NM_002318.2	4017	LOXL2	3810	11.6407	3.40112	1.7751	5.00E-05	0.00170056
NM_000570.3	2215	FCGR3B	2295	3.41392	12.5334	-1.87627	5.00E-05	0.00170056
ENST00000482351.1	ENSG00000244	RP11-88H10.2	624	1.79186	0	inf	5.00E-05	0.00170056
NM_000963.2	5743	PTGS2	4493	41.232	15.1943	1.44023	5.00E-05	0.00170056
NM_002458.2	727897	MUC5B	17916	5.19697	33.8068	-2.70157	5.00E-05	0.00170056
NM_001098526.1	120425	AMICA1	1957	62.688	28.6569	1.12931	5.00E-05	0.00170056
NM_002648.3	5292	PIM1	2709	31.7851	11.6142	1.45246	5.00E-05	0.00170056
NM_004621.5	7225	TRPC6	4612	7.72301	1.98776	1.95802	5.00E-05	0.00170056
NM_007315.3	6772	STAT1	4308	52.1415	294.177	-2.49618	5.00E-05	0.00170056
NM_001039845.1	130752	MDH1B	2332	8.98692	22.3971	-1.31741	5.00E-05	0.00170056
NM_000860.4	3248	HPGD	2770	45.0813	8.23367	2.45292	5.00E-05	0.00170056
NM_206963.1	5918	RARRES1	1526	111.904	300.035	-1.42287	5.00E-05	0.00170056
NM_000064.2	718	C3	5101	23.4449	140.483	-2.58305	5.00E-05	0.00170056
NM_018004.1	55076	TMEM45A	1564	7.37206	38.1525	-2.37164	5.00E-05	0.00170056
NM_153840.2	266977	GPR110	3078	6.44467	16.1124	-1.32199	5.00E-05	0.00170056
NM_001002912.4	127254	C1orf173	7159	12.8494	30.716	-1.25728	5.00E-05	0.00170056
NM_000602.3	5054	SERPINE1	3181	22.7471	4.26985	2.41343	5.00E-05	0.00170056
NM_152703.2	219285	SAMD9L	7133	17.7707	38.3726	-1.11058	5.00E-05	0.00170056
NM_030817.2	81575	APOLD1	4646	76.8949	6.42961	3.58008	5.00E-05	0.00170056
NM_005560.3	3911	LAMA5	11426	7.83795	3.17577	1.30337	5.00E-05	0.00170056
NM_001543.4	3340	NDST1	8030	17.1326	7.986	1.1012	5.00E-05	0.00170056
NM_133493.3	135228	CD109	9447	10.9258	5.31361	1.03997	5.00E-05	0.00170056
NM_015964.2	51673	TPPP3	1041	43.9425	111.546	-1.34395	5.00E-05	0.00170056
NM_006084.4	10379	IRF9	1678	31.5489	64.6565	-1.03521	5.00E-05	0.00170056

NM_152403.3	133584	EGFLAM	4870	3.71132	0.800839	2.21235	5.00E-05	0.00170056
NM_001992.3	2149	F2R	3810	35.0886	17.1369	1.0339	5.00E-05	0.00170056
NM_003966.2	9037	SEMA5A	11808	33.7242	14.6351	1.20435	5.00E-05	0.00170056
NM_017912.3	55008	HERC6	3889	7.19667	16.2423	-1.17436	5.00E-05	0.00170056
NM_001547.4	3433	IFIT2	3495	9.62328	21.6677	-1.17094	5.00E-05	0.00170056
NM_004235.4	9314	KLF4	2933	28.586	10.1	1.50095	5.00E-05	0.00170056
NM_001257.4	1012	CDH13	4021	9.97041	3.74921	1.41107	5.00E-05	0.00170056
TCONS_00155673	XLOC_013982	-	9015	0.546383	1.83943	-1.75128	5.00E-05	0.00170056
NM_003999.2	9180	OSMR	5539	36.0999	15.6798	1.20309	5.00E-05	0.00170056
NM_004165.2	6236	RRAD	1467	7.86083	22.5121	-1.51795	5.00E-05	0.00170056
NM_199161.3	6288	SAA1	531	25.3424	405.123	-3.99873	5.00E-05	0.00170056
NM_014485.2	27306	HPGDS	1615	16.8697	3.97371	2.08588	5.00E-05	0.00170056
NM_005253.3	2355	FOSL2	3991	51.0057	14.8043	1.78465	5.00E-05	0.00170056
NM_030754.4	6289	SAA2	594	6.43837	160.834	-4.64274	5.00E-05	0.00170056
NM_005532.3	3429	IFI27	652	63.9962	171.702	-1.42385	5.00E-05	0.00170056
NM_002112.3	3067	HDC	2644	16.561	4.25304	1.96122	5.00E-05	0.00170056
NM_000361.2	7056	THBD	4032	14.9135	3.7109	2.00677	5.00E-05	0.00170056
NM_006192.3	5075	PAX1	2838	0.251571	1.37662	-2.45209	5.00E-05	0.00170056
NM_007226.2	11249	NXPH2	2560	1.74995	9.77365	-2.48158	5.00E-05	0.00170056
NM_022159.3	64123	ELTD1	3619	41.0828	19.087	1.10594	5.00E-05	0.00170056
NM_001031743.2	154313	C6orf165	2215	11.9943	33.6332	-1.48754	5.00E-05	0.00170056
NM_014996.2	23007	PLCH1	6128	3.26263	8.39061	-1.36274	5.00E-05	0.00170056
NM_002863.4	5836	PYGL	2846	44.2717	22.7505	0.960484	5.00E-05	0.00170056
NM_007360.3	22914	KLRK1	1589	15.9313	37.949	-1.2522	5.00E-05	0.00170056
NM_002188.2	3596	IL13	1282	5.26831	0.239101	4.46165	5.00E-05	0.00170056
NM_001001548.2	948	CD36	4727	14.2394	3.65396	1.96236	5.00E-05	0.00170056
NM_000433.3	4688	NCF2	2412	59.3687	15.3069	1.95552	5.00E-05	0.00170056
NM_001031683.2	3437	IFIT3	2454	5.97555	40.5776	-2.76354	5.00E-05	0.00170056
NM_005408.2	6357	CCL13	851	168.111	2.65448	5.98484	5.00E-05	0.00170056
NM_024593.3	79645	EFCAB1	2144	11.5378	26.5466	-1.20216	5.00E-05	0.00170056
NM_004484.3	2719	GPC3	2319	6.72044	2.08326	1.68971	5.00E-05	0.00170056
TCONS_00075737	XLOC_006473	-	4288	4.48866	1.71035	1.39199	5.00E-05	0.00170056
NM_000669.3	126	ADH1C	1473	241.235	52.9387	2.18804	5.00E-05	0.00170056
NM_006981.3	8013	NR4A3	5634	32.0708	1.70819	4.23072	5.00E-05	0.00170056
NM_174858.2	26289	AK5	3325	21.9522	9.4081	1.22239	5.00E-05	0.00170056
NM_138796.2	128153	SPATA17	1235	13.7523	38.2346	-1.47521	5.00E-05	0.00170056
NM_006103.3	10406	WFDC2	566	130.577	395.577	-1.59906	5.00E-05	0.00170056
NM_003741.2	8646	CHRD	3521	8.22548	2.84222	1.53308	5.00E-05	0.00170056
NM_173672.4	285755	PPIL6	4128	5.46926	15.5037	-1.50319	5.00E-05	0.00170056
NM_001565.3	3627	CXCL10	1216	2.8455	82.0924	-4.85049	5.00E-05	0.00170056
NM_001828.4	1178	CLC	630	289.985	6.45223	5.49004	5.00E-05	0.00170056
NM_002429.4	4327	MMP19	3254	13.0461	3.13006	2.05935	5.00E-05	0.00170056
XM_003403447.1	100652938	LOC100652938	5295	15.0552	4.95798	1.60243	5.00E-05	0.00170056
NM_002976.3	6332	SCN7A	7186	0.456659	1.61744	-1.82452	5.00E-05	0.00170056
NM_005764.3	10158	PDZK1IP1	894	2.61407	11.4724	-2.1338	5.00E-05	0.00170056
NM_004657.5	8436	SDPR	3251	58.6547	25.6184	1.19507	5.00E-05	0.00170056
NM_018965.2	54209	TREM2	1051	11.5915	2.73135	2.08538	5.00E-05	0.00170056
NM_172218.2	6674	SPAG1	3749	10.1689	22.3618	-1.13688	5.00E-05	0.00170056
NM_002357.3	4084	MXD1	5617	28.2796	6.62297	2.09421	5.00E-05	0.00170056
NM_144590.2	118932	ANKRD22	3986	0.682676	4.57059	-2.74311	5.00E-05	0.00170056
NM_019074.3	54567	DLL4	3420	6.17687	1.78071	1.79442	5.00E-05	0.00170056
NM_001781.2	969	CD69	1676	260.814	25.6891	3.34379	5.00E-05	0.00170056
NM_197955.1	84419	C15orf48	803	2.07031	26.0488	-3.6533	5.00E-05	0.00170056
NM_025145.5	80217	WDR96	5365	18.3587	53.2485	-1.53628	5.00E-05	0.00170056

NM_001145128.2	221264	AKD1	6326	4.47272	10.3505	-1.21048	5.00E-05	0.00170056
NM_001010919.1	441168	FAM26F	1109	3.51969	23.2319	-2.72259	5.00E-05	0.00170056
NM_001765.2	911	CD1C	2592	10.6383	2.34992	2.17858	5.00E-05	0.00170056
NM_002993.3	6372	CXCL6	1659	2.43826	16.9881	-2.8006	5.00E-05	0.00170056
NM_014479.3	27299	ADAMDEC1	2348	1.80904	32.8639	-4.18321	5.00E-05	0.00170056
NM_000677.3	140	ADORA3	2245	11.4677	2.50683	2.19363	5.00E-05	0.00170056
NM_001025109.1	947	CD34	2616	17.8931	7.58058	1.23902	5.00E-05	0.00170056
NM_182705.2	359845	FAM101B	3622	24.8298	5.92615	2.0669	5.00E-05	0.00170056
NM_001164436.1	389177	TMEM212	1881	7.27232	16.9709	-1.22258	5.00E-05	0.00170056
NM_005218.3	1672	DEFB1	463	7.75791	46.4878	-2.58311	5.00E-05	0.00170056
NM_002928.3	6004	RGS16	2432	23.6673	2.29315	3.36749	5.00E-05	0.00170056
NM_001002235.2	5265	SERPINA1	3199	6.17486	17.1309	-1.47212	5.00E-05	0.00170056
NM_001002029.3	721	C4B	5427	0.761149	3.28823	-2.11106	5.00E-05	0.00170056
NM_000916.3	5021	OXTR	4361	46.165	10.6985	2.1094	5.00E-05	0.00170056
NM_152327.2	122481	AK7	2648	12.4514	29.7234	-1.25529	5.00E-05	0.00170056
NM_002483.4	4680	CEACAM6	2631	17.4981	48.6967	-1.47663	5.00E-05	0.00170056
NM_001039.3	6340	SCNN1G	3499	0.972854	8.62819	-3.14876	5.00E-05	0.00170056
NM_199327.1	10252	SPRY1	2348	63.8487	14.8285	2.10629	5.00E-05	0.00170056
NM_006169.2	4837	NNMT	1578	37.273	15.5147	1.26449	5.00E-05	0.00170056
NM_002616.2	5187	PER1	4709	11.9082	4.96497	1.2621	5.00E-05	0.00170056
NM_003956.3	9023	CH25H	1378	26.4124	7.25585	1.864	5.00E-05	0.00170056
NM_001145026.1	374462	PTPRQ	7612	2.97553	0.614365	2.27598	5.00E-05	0.00170056
NM_147168.1	84688	C9orf24	712	14.3379	42.1145	-1.55448	5.00E-05	0.00170056
NM_001136570.1	100129583	FAM47E	1550	5.55954	14.9331	-1.42548	5.00E-05	0.00170056
NM_021254.2	56683	C21orf59	1427	32.1281	70.4615	-1.133	5.00E-05	0.00170056
NM_199166.1	211	ALAS1	2258	45.5571	17.6648	1.3668	5.00E-05	0.00170056
TCONS_00207680	XLOC_017712	-	4806	4.42183	0.495801	3.15681	5.00E-05	0.00170056
NM_004665.2	8875	VNN2	2004	3.26765	25.001	-2.93566	5.00E-05	0.00170056
NM_025153.2	23120	ATP10B	7566	2.20884	10.5547	-2.25652	5.00E-05	0.00170056
NM_007293.2	720	C4A	5426	0.954242	2.63952	-1.46785	5.00E-05	0.00170056
28755	28755	-	954	14.429	33.4493	-1.213	5.00E-05	0.00170056
NM_001767.3	914	CD2	1531	14.4474	41.1552	-1.51026	5.00E-05	0.00170056
NM_002922.3	5996	RGS1	1403	201.782	67.9109	1.57108	5.00E-05	0.00170056
NM_001080537.1	132203	SNTN	1586	40.4648	114.313	-1.49825	5.00E-05	0.00170056
NM_002985.2	6352	CCL5	1230	43.0121	138.942	-1.69167	5.00E-05	0.00170056
NM_174975.4	266629	SEC14L3	2084	10.9827	1.07783	3.34903	5.00E-05	0.00170056
NM_006183.4	4922	NTS	1239	61.706	0.853768	6.17542	5.00E-05	0.00170056
NM_005354.4	3727	JUND	1870	42.6208	18.7078	1.18792	5.00E-05	0.00170056
NM_170776.4	222487	GPR97	2670	6.75829	0.600766	3.49178	5.00E-05	0.00170056
NM_006108.2	10418	SPON1	5384	52.9175	21.364	1.30856	5.00E-05	0.00170056
NM_030906.2	65975	STK33	2707	8.00722	18.0448	-1.17221	5.00E-05	0.00170056
NM_001105248.1	79838	TMC5	4917	18.3726	72.1613	-1.97367	5.00E-05	0.00170056
NM_145071.2	1154	CISH	2111	10.8068	3.40118	1.66783	5.00E-05	0.00170056
NM_152548.2	153643	FAM81B	1563	13.7941	39.9278	-1.53334	5.00E-05	0.00170056
NM_017625.2	55600	ITLN1	1182	27.4677	1.83576	3.90329	5.00E-05	0.00170056
3497	3497	-	1286	12.7504	0.369321	5.10953	5.00E-05	0.00170056
3495	3495	-	1154	17.7	76.9939	-2.121	5.00E-05	0.00170056
NM_138961.2	90952	ESAM	1869	9.79398	4.07708	1.26436	5.00E-05	0.00170056
NM_002800.4	5698	PSMB9	1048	22.3866	63.8106	-1.51116	5.00E-05	0.00170056
NM_001048265.1	138162	C9orf116	722	15.5363	41.1849	-1.40647	5.00E-05	0.00170056
NM_178824.3	151790	WDR49	2594	12.7196	33.0023	-1.37551	5.00E-05	0.00170056
NM_002423.3	4316	MMP7	1119	18.4867	110.857	-2.58414	5.00E-05	0.00170056
NM_152325.1	122046	C13orf26	998	13.8285	36.1467	-1.38621	5.00E-05	0.00170056
NM_005542.4	3638	INSIG1	3010	40.8928	16.2988	1.32708	5.00E-05	0.00170056

NM_139172.1	147744	TMEM190	593	16.509	50.7678	-1.62066	5.00E-05	0.00170056
NM_001843.2	1272	CNTN1	3427	28.3843	12.8639	1.14176	5.00E-05	0.00170056
NM_012409.2	23627	PRND	3980	2.1365	0.375937	2.50669	5.00E-05	0.00170056
NM_001447.2	2196	FAT2	14536	20.1554	8.4315	1.2573	5.00E-05	0.00170056
NM_005306.2	2867	FFAR2	2053	9.19102	1.82213	2.3346	5.00E-05	0.00170056
NM_173354.3	150094	SIK1	4706	45.1877	14.1567	1.67444	5.00E-05	0.00170056
NM_016270.2	10365	KLF2	1655	26.6601	9.00422	1.56601	5.00E-05	0.00170056
TCONS_00103444	XLOC_008124	-	1958	17.9778	40.7763	-1.18152	5.00E-05	0.00170056
NM_199355.2	170692	ADAMTS18	5913	2.74299	0.55061	2.31664	5.00E-05	0.00170056
NM_005252.3	2353	FOS	2158	486.04	44.4	3.45244	5.00E-05	0.00170056
NM_006820.2	10964	IFI44L	5874	13.0691	36.7922	-1.49323	5.00E-05	0.00170056
NM_006763.2	7832	BTG2	2712	210.617	76.3341	1.46422	5.00E-05	0.00170056
NM_024763.4	79819	WDR78	3848	15.1305	37.2977	-1.30163	5.00E-05	0.00170056
NM_020848.2	57608	KIAA1462	9300	11.7107	6.01114	0.962114	5.00E-05	0.00170056
NM_001199201.1	79740	ZBBX	3363	13.8194	36.1335	-1.38664	5.00E-05	0.00170056
NM_182508.2	144809	C13orf30	3302	35.1799	81.0755	-1.20451	5.00E-05	0.00170056
TCONS_00070151	XLOC_006346	-	298	3.1271	0	inf	5.00E-05	0.00170056
NM_006088.5	10383	TUBB2C	1591	115.61	247.579	-1.09863	5.00E-05	0.00170056
NM_002089.3	2920	CXCL2	1205	67.4621	6.53973	3.36677	5.00E-05	0.00170056
NM_005409.4	6373	CXCL11	1606	1.36176	25.3491	-4.2184	5.00E-05	0.00170056
NM_001010892.2	345895	RSPH4A	2840	19.0418	54.2393	-1.51017	5.00E-05	0.00170056
NM_012337.2	25790	CCDC19	1795	10.0455	33.2433	-1.72652	5.00E-05	0.00170056
NM_001898.2	1469	CST1	760	985.484	0.568934	10.7584	5.00E-05	0.00170056
NM_176813.3	155465	AGR3	750	23.579	59.9177	-1.34548	5.00E-05	0.00170056
NM_001145450.1	729967	MORN2	741	43.5044	114.162	-1.39185	5.00E-05	0.00170056
NM_000668.4	125	ADH1B	2682	1.79125	5.48494	-1.61451	5.00E-05	0.00170056
NM_001974.3	2015	EMR1	3123	19.6724	1.82071	3.4336	5.00E-05	0.00170056
NM_004040.2	388	RHOB	2367	151.131	52.2405	1.53256	5.00E-05	0.00170056
NM_212559.2	402415	XKRX	2851	2.05415	5.38637	-1.39077	5.00E-05	0.00170056
NM_003062.2	6586	SLIT3	5380	14.1848	6.12088	1.21253	5.00E-05	0.00170056
NM_001190766.1	401236	FLJ23152	1322	4.34761	15.7153	-1.85388	5.00E-05	0.00170056
NM_005424.2	7075	TIE1	3882	7.61257	3.14778	1.27405	5.00E-05	0.00170056
NM_148672.2	56477	CCL28	816	12.4647	35.2447	-1.49955	5.00E-05	0.00170056
NM_005338.4	3092	HIP1	7239	11.9612	5.0498	1.24406	5.00E-05	0.00170056
NM_012128.3	22802	CLCA4	3213	3.14861	9.70005	-1.62328	5.00E-05	0.00170056
NM_002426.4	4321	MMP12	1876	1.79022	8.25199	-2.2046	5.00E-05	0.00170056
NM_018326.2	55303	GIMAP4	1965	34.8173	81.54	-1.2277	5.00E-05	0.00170056
NM_016529.4	51761	ATP8A2	5006	4.69777	1.86093	1.33595	5.00E-05	0.00170056
NM_031457.1	83661	MS4A8B	1353	15.5796	37.2902	-1.25914	5.00E-05	0.00170056
NM_031421.2	83538	TTC25	2078	6.28726	22.0755	-1.81195	5.00E-05	0.00170056
NM_173554.2	219621	C10orf107	1252	16.1576	36.9883	-1.19485	5.00E-05	0.00170056
NM_000782.4	1591	CYP24A1	3266	2.89167	17.0522	-2.55998	5.00E-05	0.00170056
NM_017617.3	4851	NOTCH1	9295	8.52912	3.65149	1.22391	5.00E-05	0.00170056
NM_004344.1	1069	CETN2	1080	73.5583	170.124	-1.20963	5.00E-05	0.00170056
NM_004826.2	9427	ECEL1	2859	1.96723	0.30407	2.69369	5.00E-05	0.00170056
NM_001029996.3	200373	PCDP1	2968	6.57611	15.4191	-1.22941	5.00E-05	0.00170056
NM_001008226.1	283726	FAM154B	3131	13.4542	41.0976	-1.611	5.00E-05	0.00170056
NM_004599.2	6721	SREBF2	4306	20.4238	8.96612	1.18769	5.00E-05	0.00170056
NM_006072.4	10344	CCL26	562	91.7771	1.47311	5.9612	5.00E-05	0.00170056
NM_016084.4	51655	RASD1	1748	18.2422	1.84107	3.30867	5.00E-05	0.00170056
NM_003277.3	7122	CLDN5	1702	15.9801	6.1278	1.38284	5.00E-05	0.00170056
NM_000732.4	915	CD3D	741	15.1372	39.5828	-1.38678	5.00E-05	0.00170056
XM_001715090.3	144535	C12orf55	5721	7.68912	16.3407	-1.08758	5.00E-05	0.00170056
NM_153610.3	202333	CMYA5	12892	13.9632	7.07886	0.980043	5.00E-05	0.00170056

NM_018690.2	55911	APOBR	3733	5.62036	2.21831	1.3412	5.00E-05	0.00170056
NM_004666.2	8876	VNN1	3844	1.11416	5.40109	-2.27729	5.00E-05	0.00170056
NM_002084.3	2878	GPX3	1761	49.7881	18.9579	1.393	5.00E-05	0.00170056
NM_015714.3	50486	G0S2	963	16.3631	3.93563	2.05578	5.00E-05	0.00170056
NM_017662.4	140803	TRPM6	8425	3.13642	0.934859	1.7463	5.00E-05	0.00170056
NM_001848.2	1291	COL6A1	4225	75.3867	23.3507	1.69084	5.00E-05	0.00170056
NM_007028.3	11074	TRIM31	2027	0.241365	2.91783	-3.59561	5.00E-05	0.00170056
NM_007050.5	11122	PTPRT	12637	0.840909	3.37236	-2.00374	5.00E-05	0.00170056
NM_138433.3	113730	KLHDC7B	2991	0.677811	3.75932	-2.47152	5.00E-05	0.00170056
NM_001964.2	1958	EGR1	3136	73.3271	7.03404	3.38192	5.00E-05	0.00170056
NM_004233.3	9308	CD83	2478	16.9528	6.92537	1.29156	5.00E-05	0.00170056
NM_021979.3	3306	HSPA2	2770	13.7769	4.93047	1.48245	5.00E-05	0.00170056
NM_172239.4	254958	REXO1L1	7032	3.62701	13.632	-1.91015	5.00E-05	0.00170056
NM_001432.2	2069	EREG	4628	6.58287	0.215076	4.9358	5.00E-05	0.00170056
NM_032269.5	84229	CCDC135	2911	2.35336	6.64506	-1.49756	5.00E-05	0.00170056
NM_002416.1	4283	CXCL9	2545	2.33524	59.1654	-4.66311	5.00E-05	0.00170056
NM_002053.2	2633	GBP1	3050	26.3046	107.536	-2.03143	5.00E-05	0.00170056
NM_016583.3	51297	PLUNC	1023	224.289	1406.36	-2.64854	5.00E-05	0.00170056
NM_145235.3	92565	FANK1	1296	13.2293	37.0661	-1.48637	5.00E-05	0.00170056
NM_198320.3	1368	CPM	6655	8.65585	4.04036	1.09919	5.00E-05	0.00170056
NM_022068.2	63895	FAM38B	9713	7.05458	2.73723	1.36584	5.00E-05	0.00170056
NM_001369.2	1767	DNAH5	15573	14.6696	34.1222	-1.21788	5.00E-05	0.00170056
NM_000435.2	4854	NOTCH3	8071	11.359	4.95906	1.1957	5.00E-05	0.00170056
NM_033255.2	94240	EPSTI1	1508	19.9307	55.2258	-1.47035	5.00E-05	0.00170056
NM_145016.3	219970	GLYATL2	1380	1.8942	10.1025	-2.41505	5.00E-05	0.00170056
NM_024787.2	79845	RNF122	1868	9.51535	2.85743	1.73554	5.00E-05	0.00170056
NM_017631.5	55601	DDX60	6073	17.7018	42.8584	-1.27568	5.00E-05	0.00170056
NM_016232.4	9173	IL1RL1	2058	21.8276	4.78325	2.19009	5.00E-05	0.00170056
NM_197965.2	345274	SLC10A6	1502	5.36734	1.21404	2.14438	5.00E-05	0.00170056
NM_001102608.1	131873	COL6A6	8470	6.18578	0.765362	3.01474	5.00E-05	0.00170056
NM_004418.3	1844	DUSP2	1685	13.1242	4.18248	1.6498	5.00E-05	0.00170056
NM_181643.4	128344	C1orf88	2346	18.504	49.9486	-1.43261	5.00E-05	0.00170056
NM_173081.3	219681	ARMC3	2808	14.6867	36.9864	-1.33249	5.00E-05	0.00170056
NM_006674.3	10866	HCP5	2539	25.0226	45.6173	-0.866352	5.00E-05	0.00170056
NM_002923.3	5997	RGS2	1350	282.433	49.8778	2.50144	5.00E-05	0.00170056
NM_000632.3	3684	ITGAM	4742	23.0795	4.87594	2.24286	5.00E-05	0.00170056
NM_003810.3	8743	TNFSF10	1953	79.7278	176.591	-1.14726	5.00E-05	0.00170056
ENST00000544591.1	ENSG00000256	RP11-291B21.2	868	0.79541	9.04403	-3.50719	5.00E-05	0.00170056
NM_033050.4	56670	SUCNR1	1650	12.2573	1.10843	3.46706	5.00E-05	0.00170056
NM_001134486.2	115362	GBP5	3944	2.55857	20.6343	-3.01164	5.00E-05	0.00170056
NM_000399.3	1959	EGR2	2979	11.0657	1.38323	2.99998	5.00E-05	0.00170056
NM_000636.2	6648	SOD2	1593	76.6581	184.076	-1.26379	5.00E-05	0.00170056
TCONS_00111412	XLOC_009731	-	2716	0.808264	4.50001	-2.47703	5.00E-05	0.00170056
NM_080860.2	89765	RSPH1	1367	24.2134	71.1785	-1.55564	5.00E-05	0.00170056
TCONS_00311623	XLOC_026432	-	2466	3.2772	0.237297	3.7877	5.00E-05	0.00170056
NM_145244.3	115265	DDIT4L	2634	3.93055	14.2127	-1.85438	5.00E-05	0.00170056
NM_001127595.1	2214	FCGR3A	2155	14.5644	65.3784	-2.16637	5.00E-05	0.00170056
NM_001100388.1	399949	C11orf88	714	23.0691	68.9128	-1.57881	5.00E-05	0.00170056
NM_177980.2	60437	CDH26	3193	59.9684	7.08681	3.08099	5.00E-05	0.00170056
NM_022093.1	63923	TNN	5008	0.688977	2.43129	-1.81919	5.00E-05	0.00170056
NM_152721.5	220164	DOK6	8897	4.35317	1.74063	1.32246	5.00E-05	0.00170056
NM_031310.1	83483	PLVAP	2276	34.2818	11.5337	1.57159	5.00E-05	0.00170056
NM_020530.4	5008	OSM	1854	11.2806	0.386969	4.86548	5.00E-05	0.00170056
NM_004417.3	1843	DUSP1	2024	356.027	53.0246	2.74725	5.00E-05	0.00170056

NM_004120.3	2634	GBP2	2595	37.2669	102.505	-1.45972	5.00E-05	0.00170056
NM_001736.3	728	C5AR1	2342	15.3561	2.96619	2.37212	5.00E-05	0.00170056
NM_005688.2	10057	ABCC5	5845	17.1981	4.82494	1.83367	5.00E-05	0.00170056
NM_001062.3	6947	TCN1	1567	227.086	59.922	1.92208	5.00E-05	0.00170056
NM_172337.1	5015	OTX2	2069	11.9891	3.37485	1.82883	5.00E-05	0.00170056
NM_145010.2	219670	ENKUR	3318	7.58547	23.3226	-1.62042	5.00E-05	0.00170056
NM_014631.2	9644	SH3PXD2A	11247	18.3254	7.90235	1.21349	5.00E-05	0.00170056
NM_138456.3	116071	BATF2	2140	1.11191	5.63184	-2.34057	5.00E-05	0.00170056
NM_002652.2	5304	PIP	576	1.43896	16.7682	-3.54263	5.00E-05	0.00170056
NM_001165257.1	29106	SCG3	3257	0.290849	1.63918	-2.49463	5.00E-05	0.00170056
NM_002351.4	4068	SH2D1A	2517	4.80127	11.0563	-1.20338	5.00E-05	0.00170056
NM_001140.3	246	ALOX15	2684	641.058	62.3893	3.36108	5.00E-05	0.00170056
NM_002119.3	3111	HLA-DOA	3485	18.4497	38.7255	-1.06968	5.00E-05	0.00170056
3505	3505	-	1188	11.741	2.29208	2.35683	5.00E-05	0.00170056
NM_015576.1	26059	ERC2	6138	0.387774	1.448	-1.90077	5.00E-05	0.00170056
NM_022481.5	64411	ARAP3	5262	4.55992	1.69683	1.42617	5.00E-05	0.00170056
NM_015991.2	712	C1QA	1098	57.5953	144.189	-1.32394	5.00E-05	0.00170056
NM_173157.2	3164	NR4A1	2529	48.3937	2.01517	4.58585	5.00E-05	0.00170056
NM_003737.2	8642	DCHS1	10756	6.41969	3.13831	1.03252	5.00E-05	0.00170056
NM_000572.2	3586	IL10	1629	8.45793	1.65233	2.3558	5.00E-05	0.00170056
NM_000887.3	3687	ITGAX	4649	9.69334	3.94863	1.29564	5.00E-05	0.00170056
NM_001195037.2	345930	ECT2L	4343	7.36833	20.6472	-1.48654	5.00E-05	0.00170056
NM_003856.2	9173	IL1RL1	2508	34.9321	3.58984	3.28256	5.00E-05	0.00170056
NM_152447.3	145581	LRFN5	3732	1.26236	3.61184	-1.51661	5.00E-05	0.00170056
NM_152632.3	170063	CXorf22	3613	7.50052	16.8435	-1.16713	5.00E-05	0.00170056
NM_002001.2	2205	FCER1A	1165	35.7487	12.6149	1.50276	5.00E-05	0.00170056
NM_002575.2	5055	SERPINB2	1908	17.0978	2.74995	2.63633	5.00E-05	0.00170056
TCONS_00053150	XLOC_004440	-	13299	2.35416	0.890577	1.4024	5.00E-05	0.00170056
NM_006798.2	10941	UGT2A1	2602	117.118	19.8036	2.56413	5.00E-05	0.00170056
NM_014601.3	30846	EHD2	3584	11.4044	4.82548	1.24085	5.00E-05	0.00170056
NM_130782.2	64407	RGS18	2145	27.0136	12.1568	1.15192	5.00E-05	0.00170056
NM_003278.2	7123	CLEC3B	852	23.7274	93.9382	-1.98516	5.00E-05	0.00170056
NM_178329.2	1232	CCR3	1717	11.2616	0.568848	4.30722	5.00E-05	0.00170056
NM_198469.2	254956	MORN5	705	13.9086	43.3873	-1.64129	5.00E-05	0.00170056
NM_014157.3	29070	CCDC113	5272	7.57459	22.0149	-1.53924	5.00E-05	0.00170056
NM_001927.3	1674	DES	2248	0.408419	19.4006	-5.5699	5.00E-05	0.00170056
TCONS_00114410	XLOC_009730	-	4688	3.40649	16.0291	-2.23434	5.00E-05	0.00170056
NM_005024.1	5273	SERPINB10	1194	14.3433	1.07376	3.73963	5.00E-05	0.00170056
NM_000094.3	1294	COL7A1	9169	9.06823	3.6782	1.30182	5.00E-05	0.00170056
NM_001849.3	1292	COL6A2	3439	84.7591	24.0917	1.81483	5.00E-05	0.00170056
NM_003661.3	8542	APOL1	2863	18.5379	42.9307	-1.21153	5.00E-05	0.00170056
NM_030955.2	81792	ADAMTS12	4955	7.86652	2.4124	1.70526	5.00E-05	0.00170056
NM_014442.2	27181	SIGLEC8	2949	6.0318	1.02106	2.56252	5.00E-05	0.00170056
NM_000625.4	4843	NOS2	4206	138.964	21.9066	2.66527	5.00E-05	0.00170056
NM_003695.2	8581	LY6D	802	1.71025	8.65824	-2.33987	5.00E-05	0.00170056
NM_005613.5	5999	RGS4	3371	43.9162	4.64344	3.24149	5.00E-05	0.00170056
NM_001710.5	629	CFB	2627	24.1034	173.38	-2.84662	5.00E-05	0.00170056
NM_004036.3	109	ADCY3	4397	41.1765	11.4949	1.84083	5.00E-05	0.00170056
NM_003617.3	8490	RGS5	5927	127.864	36.3051	1.81637	5.00E-05	0.00170056
NM_006186.3	4929	NR4A2	3531	36.2997	3.97275	3.19175	5.00E-05	0.00170056
NM_002291.2	3912	LAMB1	5846	80.7467	31.3327	1.36573	5.00E-05	0.00170056
NM_015896.2	51364	ZMYND10	1773	12.5612	32.0479	-1.35125	5.00E-05	0.00170056
NM_002462.3	4599	MX1	2966	17.4412	42.115	-1.27184	5.00E-05	0.00170056
NM_003551.2	8382	NME5	1226	15.7707	41.4618	-1.39454	5.00E-05	0.00170056

NM_000518.4	3043	HBB	626	349.482	77.3997	2.17482	5.00E-05	0.00170056
NM_005099.4	9507	ADAMTS4	4332	7.26144	0.992717	2.8708	5.00E-05	0.00170056
NM_052929.1	114827	FHAD1	5090	7.22349	15.6616	-1.11646	5.00E-05	0.00170056
NM_139164.1	134429	STARD4	2264	36.0735	10.6328	1.76242	5.00E-05	0.00170056
NM_032372.4	84332	DYDC2	1842	3.08081	10.6912	-1.79505	5.00E-05	0.00170056
NM_021732.2	60370	AVPI1	1392	16.5954	5.55401	1.57918	5.00E-05	0.00170056
NM_002309.3	3976	LIF	3919	10.3753	2.5545	2.02205	5.00E-05	0.00170056
NM_001080420.1	85358	SHANK3	7145	3.39308	1.27257	1.41485	5.00E-05	0.00170056
NM_003890.2	8857	FCGBP	16390	5.4615	2.30969	1.2416	5.00E-05	0.00170056
NM_000450.2	6401	SELE	3875	15.0178	3.73904	2.00594	5.00E-05	0.00170056
NM_002126.4	3131	HLF	5599	27.5438	12.6699	1.12033	5.00E-05	0.00170056
NM_012472.3	23639	LRRC6	1582	7.53666	18.2451	-1.27551	5.00E-05	0.00170056
NM_001901.2	1490	CTGF	2344	245.499	96.9859	1.33987	5.00E-05	0.00170056
NM_198505.2	344905	ATP13A5	3657	7.9423	1.33878	2.56863	5.00E-05	0.00170056
NM_018284.2	2635	GBP3	3040	23.2895	55.7273	-1.2587	5.00E-05	0.00170056
NM_001511.2	2919	CXCL1	1109	39.4191	88.6328	-1.16895	5.00E-05	0.00170056
NM_199289.1	341676	NEK5	2912	5.07284	13.2112	-1.3809	5.00E-05	0.00170056
NM_203418.1	1827	RCAN1	2408	52.0108	12.997	2.00064	5.00E-05	0.00170056
NM_001276.2	1116	CHI3L1	1837	0.357531	2.65246	-2.89119	5.00E-05	0.00170056
NM_182519.2	149954	C20orf186	2174	215.456	0.743645	8.17857	5.00E-05	0.00170056
NM_001999.3	2201	FBN2	10724	3.52409	1.21924	1.53126	5.00E-05	0.00170056
NM_033049.3	56667	MUC13	2876	0.770846	13.5798	-4.13887	5.00E-05	0.00170056
NM_052942.3	115362	GBP5	4052	9.56774	63.3245	-2.72651	5.00E-05	0.00170056
TCONS_00156537	XLOC_014786	-	2200	1.41952	6.52565	-2.20072	5.00E-05	0.00170056
NM_004419.3	1847	DUSP5	2528	51.4685	9.9697	2.36807	5.00E-05	0.00170056
NM_000774.3	1572	CYP2F1	1826	2.28882	8.99271	-1.97415	5.00E-05	0.00170056
NM_001127496.1	81848	SPRY4	4941	4.25907	1.3965	1.60872	5.00E-05	0.00170056
NM_000096.3	1356	CP	4666	106.213	424.023	-1.99718	5.00E-05	0.00170056
NM_144687.2	91662	NLRP12	3871	2.62713	0.384799	2.77131	5.00E-05	0.00170056
NM_002855.4	5818	PVRL1	5481	6.69305	2.78372	1.26565	5.00E-05	0.00170056
NM_006144.3	3001	GZMA	896	18.3969	44.9368	-1.28843	5.00E-05	0.00170056
NM_000348.3	6716	SRD5A2	2445	4.02154	12.1995	-1.601	5.00E-05	0.00170056
NM_004573.2	5330	PLCB2	4677	17.4669	5.73761	1.6061	5.00E-05	0.00170056
NM_001007097.1	4915	NTRK2	7111	41.1655	16.9782	1.27776	5.00E-05	0.00170056
NM_002991.2	6369	CCL24	360	102.268	4.98571	4.35841	5.00E-05	0.00170056
NM_006931.2	6515	SLC2A3	3938	64.4682	11.5063	2.48616	5.00E-05	0.00170056
NM_001856.3	1307	COL16A1	5584	29.3916	11.8784	1.30707	5.00E-05	0.00170056
NM_152377.2	127795	C1orf87	2028	5.88121	16.2069	-1.46242	5.00E-05	0.00170056
NM_032291.2	84251	SGIP1	4694	6.60444	2.73182	1.27358	5.00E-05	0.00170056
NM_001370.1	1768	DNAH6	12795	8.36713	16.8756	-1.01214	5.00E-05	0.00170056
NM_022136.3	64092	SAMSN1	1888	74.1493	34.2492	1.11436	0.0001	0.00312189
NM_153614.2	374407	DNAJB13	1875	2.36469	6.78658	-1.52103	0.0001	0.00312189
TCONS_00075645	XLOC_006291	-	286	0	3.25314	#NAME?	0.0001	0.00312189
NM_025258.2	80737	C6orf27	2914	0.893045	2.94588	-1.72189	0.0001	0.00312189
NM_021958.3	3142	HLX	2279	4.13149	1.32048	1.6456	0.0001	0.00312189
NM_003877.3	8835	SOCS2	2210	24.7189	12.0339	1.03851	0.0001	0.00312189
NM_002104.2	3003	GZMK	1045	11.455	31.9186	-1.47842	0.0001	0.00312189
NM_002241.4	3766	KCNJ10	5306	0.233605	0.891323	-1.93187	0.0001	0.00312189
NM_000962.2	5742	PTGS1	5093	8.82318	3.46615	1.34797	0.0001	0.00312189
NM_080657.4	91543	RSAD2	3512	4.52626	10.3793	-1.19732	0.0001	0.00312189
NM_152680.2	201799	TMEM154	3216	34.4422	17.5873	0.969645	0.0001	0.00312189
NM_032576.4	84663	CYorf15B	3315	21.5738	7.31827	1.55971	0.0001	0.00312189
NM_144644.2	132851	SPATA4	1233	1.66859	5.94224	-1.83238	0.0001	0.00312189
NM_001079906.1	55422	ZNF331	4131	21.6414	7.4639	1.53579	0.0001	0.00312189

NM_004525.2	4036	LRP2	15735	15.161	7.16138	1.08205	0.0001	0.00312189
NM_014395.2	27071	DAPP1	2938	18.7676	35.8757	-0.934765	0.0001	0.00312189
NM_001039763.3	642987	TMEM232	3193	9.65451	21.0966	-1.12773	0.0001	0.00312189
NM_007199.2	11213	IRAK3	8344	13.3031	25.5484	-0.941472	0.0001	0.00312189
NM_001098816.2	26011	ODZ4	13548	3.47257	1.70121	1.02944	0.0001	0.00312189
NM_018986.3	54436	SH3TC1	4260	7.47804	3.29096	1.18415	0.0001	0.00312189
NM_001128431.2	23516	SLC39A14	4692	13.3851	6.51319	1.03919	0.0001	0.00312189
NM_181807.3	341019	DCDC1	1742	4.88631	12.0338	-1.30027	0.0001	0.00312189
NM_000093.3	1289	COL5A1	8439	19.2006	9.28306	1.04848	0.0001	0.00312189
NM_018384.4	55340	GIMAP5	1877	23.1139	54.3897	-1.23457	0.0001	0.00312189
NM_001795.3	1003	CDH5	4128	14.6776	7.34701	0.998385	0.0001	0.00312189
NM_012334.2	4651	MYO10	11430	12.9188	7.10137	0.863304	0.0001	0.00312189
TCONS_00081893	XLOC_006697	-	5183	1.2034	3.09154	-1.36122	0.0001	0.00312189
NM_173701.1	7453	WARS	2660	24.6088	71.0735	-1.53014	0.0001	0.00312189
NM_001977.3	2028	ENPEP	4995	27.8369	13.6196	1.03132	0.0001	0.00312189
NM_001814.4	1075	CTSC	1907	359.574	147.917	1.2815	0.0001	0.00312189
NM_030641.3	80830	APOL6	10139	29.2001	67.834	-1.21604	0.0001	0.00312189
NM_001085.4	12	SERPINA3	1590	469.152	89.048	2.3974	0.0001	0.00312189
NM_001206485.1	467	ATF3	3172	15.2178	1.62813	3.22447	0.0001	0.00312189
NM_014648.3	9666	DZIP3	5542	20.1262	41.1229	-1.03087	0.0001	0.00312189
NM_024592.4	79644	SRD5A3	4082	4.76951	10.7479	-1.17214	0.0001	0.00312189
NM_018215.3	55228	PNMAL1	3696	1.99899	5.23848	-1.38988	0.0001	0.00312189
NM_019055.5	54538	ROBO4	3837	3.5892	1.28779	1.47876	0.0001	0.00312189
NM_003742.2	8647	ABCB11	4775	1.19052	3.21867	-1.43487	0.0001	0.00312189
NM_001017424.2	3776	KCNK2	3597	0.464832	2.32354	-2.32154	0.0001	0.00312189
NM_198491.1	339145	FAM92B	1897	7.07712	16.2825	-1.20209	0.0001	0.00312189
NM_020733.1	57493	HEG1	9156	54.613	20.8332	1.39036	0.0001	0.00312189
NM_139244.4	134957	STXBP5	9244	20.2077	9.86349	1.03474	0.0001	0.00312189
NM_002986.2	6356	CCL11	925	26.4263	10.2261	1.36972	0.0001	0.00312189
NM_001097638.2	2524	FUT2	3089	8.62966	18.942	-1.13422	0.0001	0.00312189
3507	3507	-	1486	135.167	322.04	-1.25249	0.0001	0.00312189
NM_173054.2	5649	RELN	11565	0.71523	0.083582	3.09714	0.0001	0.00312189
NM_022168.2	64135	IFIH1	3421	13.7003	26.406	-0.946664	0.0001	0.00312189
NM_012261.3	24141	C20orf103	2042	88.4785	44.0897	1.00488	0.00015	0.00437065
NM_003480.2	8076	MFAP5	2882	0.301555	1.99581	-2.72648	0.00015	0.00437065
NM_031866.2	8325	FZD8	3186	2.55128	6.02128	-1.23885	0.00015	0.00437065
NM_001854.3	1301	COL11A1	7291	6.26187	12.5191	-0.99947	0.00015	0.00437065
NM_005581.3	4059	BCAM	2431	22.7947	11.348	1.00626	0.00015	0.00437065
NM_000265.4	653361	NCF1	1398	13.3119	5.2146	1.35209	0.00015	0.00437065
NM_002727.2	5552	SRGN	1254	600.399	222.297	1.43343	0.00015	0.00437065
NM_001039844.2	414149	ACBD7	3370	1.42869	4.13044	-1.5316	0.00015	0.00437065
NM_001128178.1	4867	NPHP1	2583	6.04621	17.4452	-1.52873	0.00015	0.00437065
NM_002546.3	4982	TNFRSF11B	2346	7.52949	3.24711	1.2134	0.00015	0.00437065
NM_080390.3	140597	TCEAL2	1101	1.34963	4.66188	-1.78835	0.00015	0.00437065
TCONS_00065152	XLOC_005437	-	2048	0.263389	1.53157	-2.53974	0.00015	0.00437065
NM_017839.4	54947	LPCAT2	5388	20.1654	10.5208	0.93864	0.00015	0.00437065
NM_007286.5	11346	SYNPO	5397	16.123	7.93292	1.0232	0.00015	0.00437065
NM_000851.3	2949	GSTM5	1570	0.357057	2.35722	-2.72286	0.00015	0.00437065
NM_012194.2	25758	C11orf41	11622	0.430519	1.21754	-1.49981	0.00015	0.00437065
NM_014058.3	28983	TMPRSS11E	2136	0.718762	2.63046	-1.87173	0.00015	0.00437065
NM_001199417.1	57636	ARHGAP23	5906	8.18209	4.05461	1.01291	0.00015	0.00437065
NM_001100917.1	144448	TSPAN19	1020	6.99581	19.1058	-1.44945	0.00015	0.00437065
NM_144575.2	92291	CAPN13	2682	41.2026	85.7017	-1.05659	0.00015	0.00437065
NM_003612.3	8482	SEMA7A	3376	4.77337	1.35053	1.82148	0.00015	0.00437065

NM_001124.1	133	ADM	1449	21.7287	10.1365	1.10004	0.00015	0.00437065
NM_015549.1	26030	PLEKHG3	4400	7.27324	3.27511	1.15106	0.00015	0.00437065
NM_001008784.2	344807	CD200R1L	1296	3.00135	0.275457	3.44571	0.00015	0.00437065
NM_001201.2	651	BMP3	5734	2.15607	5.3038	-1.29862	0.00015	0.00437065
NM_003373.3	7414	VCL	5286	61.1001	31.2114	0.969102	0.00015	0.00437065
NM_001263.3	1040	CDS1	4538	14.0527	26.3871	-0.90899	0.00015	0.00437065
NM_003950.2	9002	F2RL3	2731	1.55892	0.382255	2.02794	0.00015	0.00437065
NM_145278.3	148823	C1orf150	2626	3.94641	1.19912	1.71857	0.00015	0.00437065
NM_019105.6	7148	TNXB	13125	1.92248	0.876743	1.13274	0.00015	0.00437065
NM_012385.2	26471	NUPR1	858	25.1856	53.6217	-1.09022	0.00015	0.00437065
NM_001136528.1	5270	SERPINE2	2256	17.7188	7.49434	1.24141	0.00015	0.00437065
NM_005559.3	284217	LAMA1	9657	2.7555	1.21228	1.18459	0.00015	0.00437065
NM_018071.3	55701	ARHGEF40	5458	11.5379	5.98936	0.945909	0.00015	0.00437065
NM_001010854.1	145567	TTC7B	3434	6.09314	2.66599	1.19252	0.00015	0.00437065
NM_002728.4	5553	PRG2	857	4.98496	0.739096	2.75375	0.00015	0.00437065
NM_004104.4	2194	FASN	8458	4.86973	2.42839	1.00384	0.00015	0.00437065
NM_024694.3	79747	C6orf103	5290	4.22462	8.82672	-1.06306	0.00015	0.00437065
3502	3502	-	1348	329.99	1054.44	-1.67598	0.00015	0.00437065
NM_001172779.1	151827	LRRC34	1840	5.01262	13.5955	-1.43949	0.00015	0.00437065
NM_004445.3	2051	EPHB6	4043	6.99876	2.8412	1.3006	0.00015	0.00437065
NM_002631.2	5226	PGD	1937	78.0959	42.3541	0.882745	0.00015	0.00437065
NM_005420.2	6783	SULT1E1	1780	5.87511	14.3799	-1.29137	0.00015	0.00437065
NM_002463.1	4600	MX2	2961	7.79297	15.8927	-1.02812	0.00015	0.00437065
NM_152866.2	931	MS4A1	3594	9.24624	20.5402	-1.15151	0.0002	0.00554203
NM_002737.2	5578	PRKCA	8770	11.6082	6.43044	0.852151	0.0002	0.00554203
NM_032865.5	84951	TNS4	4072	6.5225	2.8342	1.20249	0.0002	0.00554203
NM_002964.4	6279	S100A8	523	44.1405	93.4571	-1.0822	0.0002	0.00554203
NM_001711.4	633	BGN	2444	30.5053	15.6835	0.95981	0.0002	0.00554203
NM_012190.2	10840	ALDH1L1	3125	2.22124	5.54327	-1.31937	0.0002	0.00554203
NM_172369.3	714	C1QC	1168	33.2844	65.5465	-0.977671	0.0002	0.00554203
NM_024764.2	79820	CATSPERB	3606	1.18819	3.31545	-1.48044	0.0002	0.00554203
NM_031271.3	56154	TEX15	10112	0.869623	0.269336	1.69098	0.0002	0.00554203
TCONS_00070847	XLOC_005757	-	1130	5.74113	1.67822	1.7744	0.0002	0.00554203
NM_002048.2	2619	GAS1	2828	30.6588	15.9476	0.942964	0.0002	0.00554203
NM_032930.2	85016	C11orf70	2198	8.8689	19.3708	-1.12706	0.0002	0.00554203
NM_000641.2	3589	IL11	2354	1.87655	0.355199	2.40139	0.0002	0.00554203
NM_004654.3	8287	USP9Y	10040	18.879	6.94327	1.44309	0.0002	0.00554203
NM_001004330.2	440107	PLEKHG7	3267	8.36482	16.5785	-0.986905	0.0002	0.00554203
NM_002116.7	3105	HLA-A	1611	247.044	560.15	-1.18104	0.0002	0.00554203
NM_002228.3	3725	JUN	3323	61.3097	29.5382	1.05353	0.0002	0.00554203
NM_003005.3	6403	SELP	3172	28.0713	14.3735	0.965682	0.0002	0.00554203
NM_138788.3	120224	TMEM45B	2207	7.38134	15.4231	-1.06314	0.0002	0.00554203
NM_178499.3	160777	CCDC60	2450	4.11661	9.87892	-1.2629	0.0002	0.00554203
NM_000198.3	3284	HSD3B2	1710	1.798	0.270367	2.7334	0.0002	0.00554203
NM_014840.2	9891	NUAK1	6821	20.8067	11.398	0.868263	0.0002	0.00554203
NM_020872.1	5067	CNTN3	4997	4.69864	1.99485	1.23597	0.0002	0.00554203
NM_148919.3	5696	PSMB8	1116	47.4499	91.0255	-0.939864	0.0002	0.00554203
NM_002198.2	3659	IRF1	3554	12.4595	29.911	-1.26343	0.0002	0.00554203
NM_022785.3	64800	EFCAB6	4929	3.73487	7.54714	-1.01487	0.0002	0.00554203
NM_015419.3	25878	MXRA5	9795	36.0294	18.1292	0.990864	0.0002	0.00554203
NM_004133.4	3174	HNF4G	4101	1.70434	4.132	-1.27763	0.0002	0.00554203
NM_006745.3	6307	SC4MOL	2139	34.7417	17.2295	1.01179	0.0002	0.00554203
NM_025057.2	80127	C14orf45	3106	10.2278	24.2225	-1.24385	0.0002	0.00554203
NM_016953.3	50940	PDE11A	9278	2.60291	8.35974	-1.68334	0.0002	0.00554203

NM_139072.3	92737	DNER	3272	0.930212	3.06025	-1.71802	0.0002	0.00554203
NM_001098212.1	3269	HRH1	4278	8.42974	2.13955	1.97818	0.0002	0.00554203
NM_004747.3	9231	DLG5	7479	17.2647	9.07877	0.927255	0.0002	0.00554203
NM_001037763.2	340267	COL28A1	3515	2.24992	5.41776	-1.26782	0.0002	0.00554203
NM_145027.4	221458	KIF6	3972	1.55591	3.86678	-1.31338	0.0002	0.00554203
NM_006142.3	2810	SFN	1315	29.6634	13.6089	1.12414	0.00025	0.00665625
NM_030622.6	29785	CYP2S1	2621	8.40721	3.41187	1.30106	0.00025	0.00665625
NM_001197249.1	11118	BTN3A2	3714	6.83979	19.8425	-1.53657	0.00025	0.00665625
NM_001098484.2	8671	SLC4A4	7693	7.56207	15.3342	-1.0199	0.00025	0.00665625
NM_006887.4	678	ZFP36L2	3693	65.4823	32.7293	1.00052	0.00025	0.00665625
NM_007015.2	11061	LECT1	1447	7.87089	0.481716	4.03027	0.00025	0.00665625
NM_003129.3	6713	SQLE	2989	26.5717	14.253	0.898623	0.00025	0.00665625
NM_004200.2	9066	SYT7	4532	0.726219	2.02056	-1.47628	0.00025	0.00665625
NM_015515.3	25984	KRT23	2147	25.4554	48.4544	-0.928656	0.00025	0.00665625
NM_015907.2	51056	LAP3	2100	69.7938	135.428	-0.956359	0.00025	0.00665625
NM_001089584.1	134121	C5orf49	1026	9.71501	23.1254	-1.25119	0.00025	0.00665625
ENST00000514532.2	ENSG00000248	GDNF-AS1	2913	1.95066	0.408158	2.25676	0.00025	0.00665625
NM_001077418.1	79583	TMEM231	2891	12.4892	25.0033	-1.00143	0.00025	0.00665625
NM_006083.3	3550	IK	1935	72.0982	137.998	-0.936615	0.00025	0.00665625
NM_206996.2	200162	SPAG17	7125	7.84834	19.8541	-1.33898	0.00025	0.00665625
NM_181844.3	255877	BCL6B	3525	5.72152	2.36081	1.27712	0.00025	0.00665625
NM_001004431.1	284207	METRNL	1333	27.9765	12.8821	1.11885	0.00025	0.00665625
NM_019594.3	56262	LRRC8A	4355	11.6663	5.68203	1.03787	0.00025	0.00665625
NM_012431.2	9723	SEMA3E	6629	0.205248	2.02283	-3.30094	0.0003	0.00772176
NM_015719.3	50509	COL5A3	6174	3.07053	1.34373	1.19225	0.0003	0.00772176
NM_152879.2	8527	DGKD	6294	8.68417	4.12902	1.07259	0.0003	0.00772176
NM_000336.2	6338	SCNN1B	2597	4.25172	10.0763	-1.24484	0.0003	0.00772176
NM_000297.3	5311	PKD2	5079	29.6157	15.8803	0.899122	0.0003	0.00772176
XM_003118942.2	374467	C12orf63	2401	6.2977	12.83	-1.02662	0.0003	0.00772176
NM_002821.3	5754	PTK7	4249	23.8089	11.3763	1.06547	0.0003	0.00772176
XM_003403450.1	4586	MUC5AC	6325	52.1879	21.5062	1.27896	0.0003	0.00772176
NM_004778.2	11251	GPR44	2910	6.43424	0.402489	3.99875	0.0003	0.00772176
NM_139285.2	246176	GAS2L2	3007	2.55213	5.69108	-1.157	0.0003	0.00772176
NM_001014440.3	440836	ODF3B	970	4.62358	13.1412	-1.50701	0.0003	0.00772176
NM_003263.3	7096	TLR1	2851	13.0177	23.8167	-0.871502	0.0003	0.00772176
NM_022486.3	64420	SUSD1	2996	20.4848	10.8175	0.921188	0.0003	0.00772176
NM_017539.1	55567	DNAH3	12351	9.27592	18.5056	-0.996401	0.0003	0.00772176
NM_001166114.1	10908	PNPLA6	4389	8.08424	2.78205	1.53896	0.0003	0.00772176
NM_080627.2	140710	KIAA0889	8036	5.66602	2.73365	1.05151	0.0003	0.00772176
NM_001884.3	1404	HAPLN1	4678	1.84705	0.565044	1.70879	0.0003	0.00772176
NM_005013.2	4925	NUCB2	1612	123.968	239.087	-0.947565	0.0003	0.00772176
NM_022097.2	63928	CHP2	2366	2.0191	5.60138	-1.47207	0.0003	0.00772176
NM_004490.2	2888	GRB14	2387	2.43755	0.308812	2.98063	0.0003	0.00772176
TCONS_00224870	XLOC_019135	-	2003	1.79271	0.386383	2.21404	0.0003	0.00772176
NM_025059.3	80129	C6orf97	5283	19.8813	36.7545	-0.886507	0.0003	0.00772176
NM_001145077.1	390205	LRRC10B	2219	5.45324	11.6081	-1.08995	0.0003	0.00772176
NM_057179.2	117581	TWIST2	1176	3.87553	0.927569	2.06287	0.0003	0.00772176
NM_002380.3	4147	MATN2	4110	7.87563	3.04024	1.37321	0.0003	0.00772176
TCONS_00143182	XLOC_012967	-	403	4.05286	20.7284	-2.3546	0.0003	0.00772176
NM_080475.2	89778	SERPINB11	1731	16.0947	6.30464	1.3521	0.0003	0.00772176
NM_003798.2	8727	CTNNAL1	2445	72.7985	39.3277	0.888362	0.0003	0.00772176
NM_005442.2	8320	EOMES	2754	2.49774	6.17175	-1.30505	0.0003	0.00772176
NM_019032.4	54507	ADAMTSL4	4202	3.58341	1.49578	1.26044	0.00035	0.00869612
NM_001102469.1	643418	LIPN	1197	7.02151	0.224737	4.96547	0.00035	0.00869612

NM_006259.1	5593	PRKG2	3328	0.535246	1.91374	-1.83812	0.00035	0.00869612
NM_007074.3	11151	CORO1A	1815	36.571	17.8798	1.03237	0.00035	0.00869612
NM_001142651.1	54492	NEURL1B	6417	13.1156	7.04277	0.897073	0.00035	0.00869612
TCONS_00289951	XLOC_024322	-	3108	0.228008	1.22366	-2.42405	0.00035	0.00869612
NM_080792.2	140885	SIRPA	3868	13.0512	5.41145	1.27009	0.00035	0.00869612
NM_007047.3	11118	BTN3A2	3812	8.26052	22.0597	-1.41711	0.00035	0.00869612
NM_025195.2	10221	TRIB1	3633	39.9782	19.9818	1.00052	0.00035	0.00869612
TCONS_00153471	XLOC_013915	-	2214	0.472395	2.07115	-2.13237	0.00035	0.00869612
NM_021202.1	58476	TP53INP2	4124	8.59809	4.2817	1.00583	0.00035	0.00869612
NM_003777.3	8701	DNAH11	14189	10.0375	19.8148	-0.981183	0.00035	0.00869612
NM_018012.3	55083	KIF26B	7287	3.0548	1.3824	1.1439	0.00035	0.00869612
NM_198460.2	163351	GBP6	4867	4.21823	8.84787	-1.06869	0.00035	0.00869612
NM_194298.2	220963	SLC16A9	3987	11.835	6.02939	0.97298	0.00035	0.00869612
NM_001846.2	1284	COL4A2	6264	53.7907	28.2307	0.93009	0.00035	0.00869612
NM_182758.2	256764	WDR72	7309	1.47999	3.37921	-1.1911	0.00035	0.00869612
NM_012072.3	22918	CD93	6701	46.816	23.9538	0.966748	0.00035	0.00869612
NM_014375.2	26998	FETUB	1627	4.32611	0.143275	4.91622	0.00035	0.00869612
NM_001144759.1	23187	PHLDB1	5298	10.2294	5.39679	0.922552	0.00035	0.00869612
NM_004962.3	2662	GDF10	2650	0.340603	1.86481	-2.45286	0.00035	0.00869612
NM_003221.3	7021	TFAP2B	5770	3.00171	1.31863	1.18675	0.00035	0.00869612
NM_006988.3	9510	ADAMTS1	4651	107.144	46.9244	1.19114	0.00035	0.00869612
NM_001855.3	1306	COL15A1	5273	116.82	57.4323	1.02436	0.00035	0.00869612
NM_198507.1	345757	FAM174A	1236	21.8669	42.2332	-0.949629	0.00035	0.00869612
NM_144980.3	168090	C6orf118	1839	6.11382	12.7925	-1.06516	0.0004	0.00971957
NM_145170.3	118491	TTC18	3704	10.4075	20.0308	-0.944588	0.0004	0.00971957
NM_213569.2	10529	NEBL	6941	14.6204	29.8108	-1.02786	0.0004	0.00971957
NM_014226.1	5891	RAGE	1934	4.32682	9.44141	-1.12569	0.0004	0.00971957
NM_016562.3	51284	TLR7	4992	3.58595	7.00989	-0.967037	0.0004	0.00971957
NM_002801.3	5699	PSMB10	1006	26.6902	51.9012	-0.959457	0.0004	0.00971957
NM_207313.1	124842	TMEM132E	4369	1.13778	0.301697	1.91504	0.0004	0.00971957
NM_001040274.2	221711	SYCP2L	3130	2.2879	0.774837	1.56206	0.0004	0.00971957
NM_005806.2	10215	OLIG2	2488	1.08761	0.171761	2.66268	0.0004	0.00971957
NM_001145033.1	387763	C11orf96	1328	9.44974	3.40884	1.47099	0.0004	0.00971957
NM_014330.3	23645	PPP1R15A	2383	47.0427	24.2297	0.957196	0.0004	0.00971957
NM_000379.3	7498	XDH	5717	3.00296	6.25099	-1.0577	0.0004	0.00971957
NM_013402.4	3992	FADS1	4420	13.798	7.16211	0.946005	0.0004	0.00971957
NM_145640.2	80833	APOL3	2124	20.5528	37.1896	-0.855565	0.0004	0.00971957
NM_145243.3	115209	OMA1	1909	27.9154	50.1397	-0.844893	0.0004	0.00971957
NM_001300.5	1316	KLF6	4662	51.0315	25.4495	1.00375	0.0004	0.00971957
NM_000691.4	218	ALDH3A1	1779	72.1661	32.63	1.14512	0.0004	0.00971957
NM_033502.2	55809	TRERF1	7580	7.22286	3.76842	0.938609	0.00045	0.0106989
NM_003036.3	6497	SKI	5707	12.5514	6.65812	0.914662	0.00045	0.0106989
TCONS_00220393	XLOC_019116	-	2293	0.560533	2.14554	-1.93647	0.00045	0.0106989
NM_000910.2	4887	NPY2R	3626	0.0687	0.776632	-3.49885	0.00045	0.0106989
NM_022147.2	64108	RTP4	1014	8.26535	19.5269	-1.24031	0.00045	0.0106989
NM_152505.3	150082	LCA5L	2364	3.26647	7.84089	-1.26328	0.00045	0.0106989
NM_213607.1	388389	CCDC103	1256	4.29614	10.599	-1.30281	0.00045	0.0106989
NM_023036.4	64446	DNAI2	2279	3.21297	9.18021	-1.51462	0.00045	0.0106989
NM_001080383.1	10052	GJC1	7640	5.39705	1.39741	1.94942	0.00045	0.0106989
NM_003054.4	6571	SLC18A2	3872	21.8743	12.0281	0.862829	0.00045	0.0106989
NM_153259.2	255231	MCOLN2	3044	1.94107	4.79802	-1.30559	0.00045	0.0106989
ENST00000517927.1	ENSG00000253	MIR146A	2301	1.55536	4.34139	-1.48091	0.00045	0.0106989
NM_199242.2	201294	UNC13D	4393	5.54494	2.57293	1.10776	0.00045	0.0106989
NM_130810.3	161582	DYX1C1	1993	4.95038	15.2245	-1.62079	0.00045	0.0106989

NM_030968.2	114897	C1QTNF1	2886	6.10816	2.40612	1.34403	0.00045	0.0106989
NM_152290.2	93190	C1orf158	1019	3.24005	9.91014	-1.61289	0.00045	0.0106989
TCONS_00051124	XLOC_004438	-	2822	1.0297	0.236757	2.12075	0.00045	0.0106989
NM_001136023.1	4778	NFE2	1648	3.5887	0.94211	1.9295	0.00045	0.0106989
NM_000389.4	1026	CDKN1A	2159	27.6653	13.9411	0.988735	0.0005	0.0116249
NM_021181.3	57823	SLAMF7	2657	27.8412	50.9278	-0.871232	0.0005	0.0116249
NM_005940.3	4320	MMP11	2261	6.91969	2.52046	1.45702	0.0005	0.0116249
NM_020869.3	100506627	DCDC5	4759	2.80936	5.95678	-1.08429	0.0005	0.0116249
NM_001145018.1	283152	CCDC153	767	4.90997	13.1147	-1.4174	0.0005	0.0116249
NM_001195278.1	100507421	LOC100507421	10558	0.766039	1.73683	-1.18097	0.0005	0.0116249
NM_005618.3	28514	DLL1	3310	5.08169	2.19691	1.20984	0.0005	0.0116249
NM_016341.3	51196	PLCE1	7992	14.7703	7.8013	0.92091	0.0005	0.0116249
NM_024870.2	80243	PREX2	5132	36.3002	17.9356	1.01715	0.0005	0.0116249
NM_001630.2	244	ANXA8L2	2030	3.88099	1.39666	1.47445	0.0005	0.0116249
NM_024783.3	79841	AGBL2	3593	4.97866	9.67783	-0.958927	0.0005	0.0116249
NM_000088.3	1277	COL1A1	5927	100.885	40.5953	1.31333	0.0005	0.0116249
TCONS_00295615	XLOC_025629	-	3213	3.62509	1.36221	1.41206	0.0005	0.0116249
28834	28834	-	318	24.9177	65.6492	-1.39761	0.0005	0.0116249
NM_173528.2	161502	C15orf26	1575	4.11994	9.90668	-1.26578	0.0005	0.0116249
NM_001170700.1	401124	DTHD1	4336	11.1902	23.9655	-1.09873	0.0005	0.0116249
NM_000552.3	7450	VWF	8833	48.438	22.4904	1.10683	0.0005	0.0116249
NM_181789.2	342035	GLDN	5084	3.7917	1.64141	1.20791	0.00055	0.0124813
NM_022350.3	64167	ERAP2	5691	6.15131	21.9504	-1.83528	0.00055	0.0124813
NM_000882.3	3592	IL12A	1443	0.52224	2.26183	-2.11471	0.00055	0.0124813
NM_001145442.1	641455	POTEM	6666	0.27445	0.823286	-1.58485	0.00055	0.0124813
NM_138373.3	91663	MYADM	3054	9.441	3.99434	1.24098	0.00055	0.0124813
NM_133639.3	171177	RHOV	1705	9.88452	19.826	-1.00415	0.00055	0.0124813
NM_018191.3	55213	RCBTB1	4033	45.4989	24.2525	0.907699	0.00055	0.0124813
NM_006573.4	10673	TNFSF13B	2671	7.06613	14.1867	-1.00554	0.00055	0.0124813
NM_004061.3	1010	CDH12	4345	0.634424	1.73076	-1.44788	0.00055	0.0124813
NM_017654.3	54809	SAMD9	6855	21.0549	38.3283	-0.864252	0.00055	0.0124813
NM_138319.2	5046	PCSK6	4513	4.43193	1.36744	1.69645	0.00055	0.0124813
NM_001008747.1	441294	CTAGE15P	2355	1.47239	0.29249	2.3317	0.00055	0.0124813
NM_004815.3	9411	ARHGAP29	9117	26.7145	14.313	0.900296	0.00055	0.0124813
NM_130445.2	80781	COL18A1	5391	22.0442	8.94175	1.30177	0.00055	0.0124813
NM_003294.3	7177	TPSAB1	1194	31.0668	15.2213	1.02928	0.00055	0.0124813
NM_016406.3	51506	UFC1	1122	56.2294	102.054	-0.859942	0.00055	0.0124813
NM_182985.3	140691	TRIM69	1901	0.8744	2.88147	-1.72044	0.00055	0.0124813
NM_002038.3	2537	IFI6	836	14.3923	35.7876	-1.31416	0.0006	0.013408
NM_002081.2	2817	GPC1	3686	5.11621	2.36313	1.11438	0.0006	0.013408
NM_007257.5	10687	PNMA2	4846	0.294481	0.987197	-1.74516	0.0006	0.013408
NM_001077594.1	91828	EXOC3L4	2581	0.188116	0.970132	-2.36656	0.0006	0.013408
NM_022073.3	112399	EGLN3	2722	22.4964	40.2903	-0.840738	0.0006	0.013408
NM_001764.2	910	CD1B	1396	2.09678	0.474486	2.14374	0.0006	0.013408
NM_032965.4	6359	CCL15	1080	1.17134	4.10135	-1.80794	0.0006	0.013408
NM_006074.4	10346	TRIM22	3091	53.6723	95.9699	-0.838404	0.0006	0.013408
NM_003745.1	8651	SOCS1	1216	8.32931	2.88059	1.53183	0.0006	0.013408
NM_020795.2	57555	NLGN2	4642	3.73661	1.69618	1.13944	0.00065	0.0142695
NM_138337.5	160364	CLEC12A	1555	11.8007	4.18697	1.49489	0.00065	0.0142695
NM_032606.3	84698	CAPS2	2135	6.00691	12.5271	-1.06035	0.00065	0.0142695
NM_030636.2	80820	EEPD1	4687	3.16523	1.39822	1.17872	0.00065	0.0142695
NM_173565.3	222967	RSPH10B	3106	1.18031	3.06025	-1.37449	0.00065	0.0142695
TCONS_00297361	XLOC_024773	-	1955	2.43246	0.783058	1.63522	0.00065	0.0142695
NM_015653.3	26150	RIBC2	1396	3.66533	9.2001	-1.32771	0.00065	0.0142695

NM_018984.3	54434	SSH1	8535	10.1446	5.6266	0.850375	0.00065	0.0142695
NM_005101.3	9636	ISG15	667	9.34711	23.4989	-1.33	0.00065	0.0142695
NM_018240.5	55243	KIRREL	3614	11.9791	6.42204	0.899411	0.00065	0.0142695
NM_020893.2	100499483	C9orf174	5635	5.77593	10.6185	-0.878451	0.00065	0.0142695
NM_030882.2	23780	APOL2	2545	19.4726	34.9894	-0.845475	0.00065	0.0142695
NM_003141.3	6737	TRIM21	1929	14.6069	27.0678	-0.889933	0.00065	0.0142695
NM_014220.2	4071	TM4SF1	1691	88.6085	49.9385	0.827291	0.00065	0.0142695
NM_001001.3	6166	RPL36AL	523	300.209	524.487	-0.80494	0.00065	0.0142695
NM_194302.2	255101	CCDC108	5945	1.19487	2.83059	-1.24425	0.00065	0.0142695
NM_001548.3	3434	IFIT1	1861	20.1525	36.7984	-0.868681	0.00065	0.0142695
NM_152419.2	138050	HGSNAT	5214	15.348	8.76072	0.808931	0.0007	0.015106
NM_005127.2	9976	CLEC2B	2038	18.8982	33.8776	-0.842085	0.0007	0.015106
NM_001174072.1	256987	SERINC5	6479	24.783	14.2033	0.80313	0.0007	0.015106
NM_003714.2	8614	STC2	5343	1.74891	0.706586	1.30752	0.0007	0.015106
NM_020812.2	57572	DOCK6	6387	3.42954	1.63271	1.07074	0.0007	0.015106
NM_003253.2	7074	TIAM1	7198	7.1131	3.84601	0.887117	0.0007	0.015106
XM_373925.8	388813	LOC388813	886	3.68341	0.278227	3.72671	0.0007	0.015106
NM_001099221.1	497189	TIFAB	1273	0.327221	2.10709	-2.68691	0.0007	0.015106
NM_017523.2	54739	XAF1	3626	8.32362	21.9837	-1.40115	0.0007	0.015106
NM_000573.3	1378	CR1	8616	13.4739	6.72801	1.00191	0.0007	0.015106
NM_173808.2	257194	NEGR1	5669	2.27603	4.85205	-1.09207	0.0007	0.015106
NM_001112802.1	6546	SLC8A1	6032	12.9548	23.0085	-0.828683	0.0007	0.015106
NM_001004303.4	199920	C1orf168	3460	2.06803	4.61639	-1.1585	0.0007	0.015106
NM_004795.3	9365	KL	5006	4.16757	2.00357	1.05663	0.0007	0.015106
NM_144993.1	200424	TET3	10983	8.97817	5.00923	0.841833	0.0007	0.015106
NM_006500.2	4162	MCAM	3332	37.3498	21.1585	0.819866	0.0007	0.015106
NM_001004306.1	339184	CCDC144NL	2806	0.156135	0.824593	-2.40089	0.0007	0.015106
NM_003608.3	8477	GPR65	4522	18.477	9.4031	0.97452	0.00075	0.0159498
NM_001135217.1	10233	LRRC23	1800	15.7882	40.1114	-1.34517	0.00075	0.0159498
ENST00000457157.2	ENSG00000231	AP000688.8	1048	2.87908	8.02273	-1.47848	0.00075	0.0159498
NM_001657.2	374	AREG	1238	11.8994	0.20792	5.83872	0.00075	0.0159498
NM_080387.4	338339	CLEC4D	1936	0.448695	1.80565	-2.00871	0.00075	0.0159498
NM_000639.1	356	FASLG	1860	2.1693	5.55165	-1.35569	0.00075	0.0159498
NM_001139490.1	26146	TRAF3IP1	3978	11.3496	20.5616	-0.857316	0.00075	0.0159498
NM_006685.3	10879	SMR3B	785	5.9163	0.425649	3.79696	0.00075	0.0159498
NM_198483.3	285180	RUFY4	2761	0.484136	1.45906	-1.59155	0.00075	0.0159498
NM_016323.2	51191	HERC5	3513	5.74998	11.1461	-0.954909	0.00075	0.0159498
NM_023037.2	10129	FRY	10717	16.9511	9.44066	0.844416	0.00075	0.0159498
NM_003045.4	6541	SLC7A1	7343	12.3015	6.85692	0.843197	0.00075	0.0159498
NM_002982.3	6347	CCL2	749	116.617	61.4655	0.923925	0.00075	0.0159498
NM_001080522.2	57545	CC2D2A	5240	11.7128	20.9343	-0.837778	0.0008	0.0168217
NM_000846.4	2939	GSTA2	1309	8.29282	3.1517	1.39573	0.0008	0.0168217
NM_001008220.1	10814	CPLX2	4676	0.859335	0.252785	1.76531	0.0008	0.0168217
NM_001085429.1	155006	TMEM213	3390	5.64097	14.6229	-1.37422	0.0008	0.0168217
NM_007351.2	22915	MMRN1	4969	42.9437	21.3202	1.01022	0.0008	0.0168217
TCONS_00011431	XLOC_002514	-	5004	0.258956	0.804755	-1.63585	0.0008	0.0168217
NM_032446.2	84466	MEGF10	7689	1.38892	0.55008	1.33625	0.0008	0.0168217
NM_032133.4	84073	MYCBPAP	3190	1.12218	2.88819	-1.36387	0.0008	0.0168217
NM_173855.4	283385	MORN3	1158	2.43195	6.64495	-1.45014	0.00085	0.0176689
NM_000046.3	411	ARSB	6076	10.7126	5.8005	0.885064	0.00085	0.0176689
NM_018482.2	50807	ASAP1	6042	29.2342	16.6334	0.813574	0.00085	0.0176689
NM_175571.2	155038	GIMAP8	3943	11.2936	20.4301	-0.855189	0.00085	0.0176689
NM_001007544.1	440712	C1orf186	1129	13.5028	5.76881	1.22692	0.00085	0.0176689
NM_014312.3	23584	VSIG2	1121	12.0712	4.8001	1.33043	0.00085	0.0176689

NM_000204.3	3426	CFI	2161	127.844	70.8531	0.851476	0.00085	0.0176689
NM_181449.2	342510	CD300E	3525	0.944227	2.35534	-1.31873	0.00085	0.0176689
NM_005401.4	5784	PTPN14	13443	13.8102	7.92609	0.801058	0.00085	0.0176689
NM_207517.2	57188	ADAMTSL3	7336	1.00455	2.21176	-1.13864	0.00085	0.0176689
TCONS_00268990	XLOC_023119	-	3294	0.489037	2.03454	-2.05669	0.00085	0.0176689
NM_018402.1	55801	IL26	1047	0.304866	2.5518	-3.06527	0.00085	0.0176689
NM_001206.2	687	KLF9	5208	46.8671	24.5521	0.93273	0.0009	0.0184743
TCONS_00017868	XLOC_001000	-	1620	1.66766	4.99146	-1.58164	0.0009	0.0184743
NM_003821.5	8767	RIPK2	2585	12.6198	6.28924	1.00474	0.0009	0.0184743
NM_002984.2	6351	CCL4	667	33.9214	14.8883	1.18802	0.0009	0.0184743
NM_005204.2	1326	MAP3K8	3096	30.9818	16.5043	0.908578	0.0009	0.0184743
NM_033274.3	8728	ADAM19	6481	10.8558	5.89477	0.88096	0.0009	0.0184743
NM_030764.3	79368	FCRL2	2589	1.88063	4.58836	-1.28676	0.0009	0.0184743
NM_024940.6	80005	DOCK5	7555	15.4059	8.69233	0.825665	0.0009	0.0184743
TCONS_00223119	XLOC_019117	-	1701	1.20423	3.82252	-1.66641	0.0009	0.0184743
NM_152275.3	92104	TTC30A	4669	6.95082	12.389	-0.833803	0.0009	0.0184743
NM_054027.4	56172	ANKH	8207	18.9332	10.6418	0.831182	0.0009	0.0184743
NM_144966.5	158326	FREM1	10086	3.61922	1.86734	0.954691	0.0009	0.0184743
NM_015529.2	26002	MOXD1	3039	5.96796	11.5219	-0.949073	0.0009	0.0184743
NM_001010978.2	388633	LDLRAD1	785	4.76103	13.1774	-1.46872	0.0009	0.0184743
NM_002381.4	4148	MATN3	2583	3.62209	1.41571	1.3553	0.00095	0.0192772
NM_001198568.1	196883	ADCY4	3720	2.99196	1.08817	1.45919	0.00095	0.0192772
NM_207317.1	133923	ZNF474	1957	6.47632	13.1987	-1.02715	0.00095	0.0192772
NM_001004439.1	22801	ITGA11	5009	4.91825	2.4233	1.02117	0.00095	0.0192772
NM_020212.1	56964	WDR93	2409	1.50382	3.97313	-1.40164	0.00095	0.0192772
NM_032108.3	10501	SEMA6B	3942	3.40705	1.47447	1.20832	0.00095	0.0192772
NM_001242729.1	54848	ARHGEF38	5456	2.68672	5.55674	-1.04839	0.00095	0.0192772
NM_138451.1	115811	IQCD	1350	4.24084	10.0952	-1.25125	0.00095	0.0192772
NM_201563.4	9103	FCGR2C	1510	7.31261	15.6102	-1.09403	0.00095	0.0192772
NM_001242699.1	387712	ENO4	2865	1.8675	4.29761	-1.20242	0.001	0.0199964
NM_182911.3	80705	TSGA10	3664	6.51924	14.655	-1.16861	0.001	0.0199964
NM_052939.3	115352	FCRL3	3019	0.754228	2.10801	-1.48281	0.001	0.0199964
NM_000061.2	695	BTK	2611	22.4299	12.1575	0.883575	0.001	0.0199964
NM_000419.3	3674	ITGA2B	3334	1.06	0.0736001	3.84821	0.001	0.0199964
NM_001127391.1	130540	ALS2CR12	1944	3.72552	8.60666	-1.20801	0.001	0.0199964
NM_004273.4	9469	CHST3	6973	8.48533	4.81123	0.818565	0.001	0.0199964
NM_001763.2	909	CD1A	2096	2.13867	0.614592	1.79901	0.001	0.0199964
NM_138805.2	131177	FAM3D	1308	19.2349	37.9367	-0.979866	0.001	0.0199964
NM_004065.2	1038	CDR1	1299	8.47655	3.38912	1.32257	0.001	0.0199964
TCONS_00314614	XLOC_026613	-	3674	2.35048	0.547369	2.10237	0.001	0.0199964
NM_022122.2	64066	MMP27	1647	2.18426	0.528107	2.04824	0.001	0.0199964
NM_018602.3	55466	DNAJA4	3186	6.74825	24.0916	-1.83594	0.001	0.0199964
NM_005502.3	19	ABCA1	10503	15.7723	27.5039	-0.802243	0.001	0.0199964
NM_006890.3	1087	CEACAM7	2289	0.729314	2.41161	-1.72538	0.001	0.0199964
NM_002049.3	2623	GATA1	1480	1.96682	0.172154	3.51409	0.00105	0.0207193
NM_139055.2	170689	ADAMTS15	5676	2.80396	1.27314	1.13908	0.00105	0.0207193
NM_002127.5	3135	HLA-G	1578	0.14741	4.88624	-5.05082	0.00105	0.0207193
NM_001009909.2	338645	LUZP2	5257	0.669382	1.8324	-1.45284	0.00105	0.0207193
NM_172341.1	55851	PSENEN	665	46.417	84.8574	-0.870388	0.00105	0.0207193
NM_182791.2	80125	CCDC33	2104	2.22516	5.96347	-1.42224	0.00105	0.0207193
NM_004265.2	9415	FADS2	3149	13.0949	6.83182	0.938662	0.00105	0.0207193
NM_001031715.2	64799	IQCH	4239	2.66142	5.6097	-1.07573	0.00105	0.0207193
NM_153449.2	144195	SLC2A14	2195	2.0701	0.576444	1.84445	0.00105	0.0207193
NM_001042406.1	54511	HMGCLL1	2471	0.186472	0.943962	-2.33977	0.00105	0.0207193

NM_021155.3	30835	CD209	4328	10.654	3.92386	1.44104	0.0011	0.0214605
NM_138983.2	116448	OLIG1	2279	1.56839	0.0871342	4.1699	0.0011	0.0214605
NM_004468.3	2275	FHL3	1704	5.23442	2.14656	1.28601	0.0011	0.0214605
NM_006094.4	10395	DLC1	6044	8.48575	2.43629	1.80036	0.0011	0.0214605
NM_014786.3	9828	ARHGEF17	7813	7.28284	4.15059	0.811183	0.0011	0.0214605
NM_178550.4	339512	C1orf110	1552	13.6729	26.3071	-0.94414	0.0011	0.0214605
NM_138787.2	119710	C11orf74	820	20.5905	41.3383	-1.0055	0.0011	0.0214605
NM_020817.1	57577	KIAA1407	3976	6.68479	12.0694	-0.852398	0.0011	0.0214605
NM_003707.2	8607	RUVBL1	1768	23.6104	41.3197	-0.80741	0.0011	0.0214605
NM_181426.1	339829	CCDC39	3854	10.2866	18.7134	-0.863298	0.0011	0.0214605
NM_002272.3	3851	KRT4	2147	12.8159	5.1576	1.31316	0.0011	0.0214605
NM_182832.2	191585	PLAC4	10009	9.24676	3.81147	1.2786	0.0011	0.0214605
NM_138806.3	131450	CD200R1	2272	18.6071	9.34796	0.993126	0.0011	0.0214605
NM_002391.3	4192	MDK	854	47.8818	84.0991	-0.812613	0.00115	0.0222874
NM_001105677.2	574537	UGT2A2	2568	22.1536	2.74928	3.01042	0.00115	0.0222874
NM_018010.3	55081	IFT57	3203	26.8654	46.4517	-0.789983	0.00115	0.0222874
NM_005911.5	4144	MAT2A	3006	93.8205	48.4813	0.952475	0.00115	0.0222874
NM_130848.2	140947	C5orf20	3135	0.504046	1.53527	-1.60687	0.00115	0.0222874
NM_021800.2	56521	DNAJC12	1212	1.06264	3.9366	-1.8893	0.00115	0.0222874
NM_078483.2	206358	SLC36A1	5773	5.94467	3.15992	0.911707	0.00115	0.0222874
NM_145045.4	115948	CCDC151	2162	1.92587	4.77357	-1.30956	0.0012	0.0230244
NM_015662.1	26160	IFT172	5360	14.0191	24.2051	-0.787917	0.0012	0.0230244
NM_001080484.1	85452	KIAA1751	4710	0.878993	2.1642	-1.29991	0.0012	0.0230244
NM_145175.2	151354	FAM84A	6355	9.19153	5.13571	0.839742	0.0012	0.0230244
TCONS_00156795	XLOC_014990	-	884	2.62064	0.387404	2.75801	0.0012	0.0230244
NM_198559.1	375307	C2orf62	1323	1.10026	3.33528	-1.59997	0.0012	0.0230244
NM_001144956.1	284485	RIIAD1	455	6.99389	19.4759	-1.47752	0.0012	0.0230244
NM_033063.1	4135	MAP6	2648	6.27087	13.326	-1.08751	0.0012	0.0230244
NM_014971.1	22979	EFR3B	7458	1.41823	0.600893	1.23892	0.0012	0.0230244
NM_001372.3	1770	DNAH9	13750	6.92764	11.576	-0.740705	0.0012	0.0230244
NM_001122962.1	284759	SIRPB2	2833	2.8641	0.75271	1.92792	0.0012	0.0230244
NM_000845.2	2918	GRM8	3559	1.6916	0.299634	2.49712	0.0012	0.0230244
NM_173695.2	286464	CXorf59	1956	6.9319	13.6792	-0.980663	0.00125	0.02372
NM_018584.5	55450	CAMK2N1	2351	10.5932	5.06614	1.06418	0.00125	0.02372
NM_206827.1	387496	RASL11A	1543	11.8176	5.68	1.05697	0.00125	0.02372
NM_003047.3	6548	SLC9A1	4572	4.06362	1.98031	1.03704	0.00125	0.02372
NM_001759.3	894	CCND2	6522	49.9887	27.4808	0.863177	0.00125	0.02372
NM_001009562.4	338821	SLCO1B7	1923	0.16444	1.03948	-2.66023	0.00125	0.02372
NM_012305.3	161	AP2A2	4653	9.43441	4.66213	1.01694	0.00125	0.02372
ENST00000604271.2	ENSG00000270	RP11-355F16.1	3438	8.94045	2.54713	1.81148	0.00125	0.02372
NM_003482.3	8085	MLL2	19419	8.02107	4.71166	0.767558	0.00125	0.02372
TCONS_00173159	XLOC_014288	-	1849	0.482068	1.66117	-1.78489	0.00125	0.02372
NM_000395.2	1439	CSF2RB	4848	21.9423	12.4538	0.817131	0.0013	0.0244278
28638	28638	-	541	27.7923	57.1797	-1.04082	0.0013	0.0244278
NM_022468.4	64386	MMP25	3554	2.09125	0.598633	1.80463	0.0013	0.0244278
NM_015210.3	23255	CCDC165	6092	1.90827	0.866134	1.1396	0.0013	0.0244278
NM_015492.4	56905	C15orf39	4427	5.89063	3.00237	0.972322	0.0013	0.0244278
NM_004926.2	677	ZFP36L1	3022	70.8804	40.8176	0.796193	0.0013	0.0244278
NM_001085447.1	129881	C2orf77	2169	6.0993	12.3097	-1.01308	0.0013	0.0244278
NM_020949.2	57709	SLC7A14	10133	1.9959	0.999091	0.998351	0.0013	0.0244278
NM_002208.4	3682	ITGAE	3861	3.18089	6.84969	-1.10661	0.0013	0.0244278
TCONS_00165548	XLOC_013709	-	2638	3.47828	1.43576	1.27656	0.0013	0.0244278
NM_001144892.1	2246	FGF1	3660	5.53292	2.17444	1.3474	0.0013	0.0244278
NM_018664.2	55509	BATF3	981	4.14262	1.27248	1.7029	0.00135	0.0250595

NM_078488.1	8875	VNN2	1946	1.7308	8.73203	-2.33488	0.00135	0.0250595
NM_032876.4	84962	JUB	4236	8.74499	4.41432	0.986268	0.00135	0.0250595
NM_006519.2	6993	DYNLT1	759	56.6525	100.243	-0.823287	0.00135	0.0250595
NM_001225.3	837	CASP4	1319	68.1102	112.223	-0.720424	0.00135	0.0250595
NM_005317.2	3004	GZMM	947	1.56867	4.69994	-1.5831	0.00135	0.0250595
NM_152367.2	126868	MAB21L3	3229	14.9894	26.0048	-0.794832	0.00135	0.0250595
NM_005356.3	3932	LCK	2117	7.39862	13.9656	-0.916547	0.00135	0.0250595
NM_001540.3	3315	HSPB1	897	35.363	17.5759	1.00864	0.00135	0.0250595
NM_000958.2	5734	PTGER4	3416	21.573	12.321	0.808103	0.00135	0.0250595
NM_014244.4	9509	ADAMTS2	6754	10.9509	6.20526	0.819479	0.00135	0.0250595
NM_030583.2	4147	MATN2	4053	6.24513	2.37039	1.39761	0.0014	0.0257815
NM_207015.2	254827	NAALADL2	4912	6.55801	11.5681	-0.818828	0.0014	0.0257815
NM_178006.3	90627	STARD13	5926	8.59432	4.14111	1.05336	0.0014	0.0257815
NM_000161.2	2643	GCH1	2926	9.08814	16.5023	-0.86061	0.0014	0.0257815
NM_001079872.1	8450	CUL4B	5184	68.2226	37.7208	0.854891	0.0014	0.0257815
NM_018485.1	27202	GPR77	1287	2.52364	0.742035	1.76595	0.0014	0.0257815
NM_001001555.2	2887	GRB10	5032	5.43273	1.88856	1.52439	0.0014	0.0257815
NM_182493.2	91807	MYLK3	7998	0.309853	0.834925	-1.43006	0.0014	0.0257815
NM_001013627.2	340527	NHSL2	3678	8.51845	4.12557	1.046	0.0014	0.0257815
NM_177937.2	51280	GOLM1	3080	18.891	50.4444	-1.417	0.0014	0.0257815
NM_020830.3	57590	WDFY1	4607	39.908	67.8244	-0.765127	0.0014	0.0257815
TCONS_00095311	XLOC_007699	-	5854	0.553437	1.39678	-1.33562	0.0014	0.0257815
NM_003248.4	7060	THBS4	3223	1.12346	0.367177	1.6134	0.00145	0.0264203
NM_007244.2	11272	PRR4	564	10.6538	2.95333	1.85095	0.00145	0.0264203
NM_014572.2	26524	LATS2	5543	10.8749	6.22344	0.805215	0.00145	0.0264203
ENST00000515343.1	ENSG00000251	RP11-281P23.1	4019	3.2182	6.62469	-1.0416	0.00145	0.0264203
NM_019035.3	54510	PCDH18	5906	51.4343	28.0625	0.874091	0.00145	0.0264203
NM_014737.2	9770	RASSF2	5426	44.2518	25.4755	0.796624	0.00145	0.0264203
NM_182665.2	83593	RASSF5	3514	24.6482	13.4319	0.87582	0.00145	0.0264203
NM_000189.4	3099	HK2	7093	13.488	7.87652	0.776048	0.00145	0.0264203
NM_001109809.2	346171	ZFP57	1870	0.154096	1.60841	-3.38373	0.00145	0.0264203
NM_021814.4	60481	ELOVL5	3000	111.558	62.9874	0.824659	0.00145	0.0264203
NM_001546.2	3400	ID4	2344	39.4605	22.0881	0.837139	0.00145	0.0264203
NM_018421.3	55357	TBC1D2	3317	7.33901	3.74643	0.97007	0.00145	0.0264203
NM_003068.4	6591	SNAI2	2112	38.589	22.3048	0.790832	0.00145	0.0264203
NM_001008.3	6192	RPS4Y1	897	235.471	100.051	1.23481	0.00145	0.0264203
NM_173644.1	284756	C20orf197	2732	1.7911	0.646591	1.46993	0.00145	0.0264203
NM_000584.3	3576	IL8	1705	17.5023	34.9818	-0.999059	0.00145	0.0264203
NM_004212.3	9153	SLC28A2	2463	0.114975	0.778614	-2.75959	0.0015	0.0270821
TCONS_00259832	XLOC_021398	-	724	0.873513	5.1404	-2.55698	0.0015	0.0270821
NM_001010906.1	389643	C8orf80	3887	3.08058	6.11576	-0.989333	0.0015	0.0270821
NM_001031745.1	158787	RIBC1	1416	2.28167	8.10986	-1.82959	0.0015	0.0270821
NM_207437.3	196385	DNAH10	13680	6.57225	11.213	-0.770716	0.0015	0.0270821
NM_003370.3	7408	VASP	2291	31.042	18.0479	0.782393	0.0015	0.0270821
NM_024800.4	79858	NEK11	2953	7.30507	14.9166	-1.02995	0.0015	0.0270821
NM_058170.2	118427	OLFM3	3462	1.00447	0.302689	1.73053	0.0015	0.0270821
NM_022355.2	64174	DPEP2	1709	3.63164	1.26932	1.51656	0.0015	0.0270821
NM_002421.3	4312	MMP1	2055	18.6925	5.08238	1.87888	0.0015	0.0270821
NM_012387.2	23569	PADI4	2265	4.85325	0.0841677	5.84954	0.00155	0.0278206
NM_152773.4	255758	TCTEX1D2	679	9.06833	21.1493	-1.2217	0.00155	0.0278206
NM_017681.2	54830	NUP62CL	1762	1.52703	3.93222	-1.36461	0.00155	0.0278206
NM_000817.2	2571	GAD1	3476	0.959492	2.48666	-1.37387	0.00155	0.0278206
NM_014470.3	27289	RND1	1665	3.59408	1.21584	1.56367	0.00155	0.0278206
NM_001007595.2	388125	C2CD4B	1576	1.86885	0.512985	1.86516	0.00155	0.0278206

NM_003280.2	7134	TNNC1	696	6.91972	2.07537	1.73734	0.00155	0.0278206
NM_016333.3	23524	SRRM2	9353	39.5855	22.7228	0.800833	0.00155	0.0278206
NM_020377.2	57105	CYSLTR2	2548	6.56887	3.10928	1.07907	0.0016	0.0284826
NM_001039706.2	79846	C7orf63	3902	6.30568	12.3613	-0.97111	0.0016	0.0284826
NM_001127443.1	948	CD36	1812	19.7492	2.02754	3.28399	0.0016	0.0284826
NM_024926.2	79989	TTC26	2151	7.88482	14.6594	-0.894677	0.0016	0.0284826
NM_000370.3	7274	TTPA	2633	1.74409	0.598018	1.54421	0.0016	0.0284826
NM_144992.4	200403	VWA3B	4747	5.95631	10.3993	-0.804001	0.0016	0.0284826
NM_001248.2	956	ENTPD3	2793	11.7803	6.1803	0.930633	0.0016	0.0284826
NM_000177.4	2934	GSN	2649	197.177	69.3361	1.50781	0.0016	0.0284826
NM_006725.3	923	CD6	3289	1.75579	4.00962	-1.19135	0.00165	0.0290957
NM_002407.2	4246	SCGB2A1	521	17.7776	38.8369	-1.12737	0.00165	0.0290957
TCONS_00091128	XLOC_007434	-	1938	0.535525	1.65951	-1.63173	0.00165	0.0290957
NM_000640.2	3598	IL13RA2	1373	2.78497	0.558245	2.31869	0.00165	0.0290957
NM_002005.3	2242	FES	2783	10.3519	5.31512	0.961715	0.00165	0.0290957
NM_004126.3	2791	GNG11	954	74.0698	42.3736	0.805721	0.00165	0.0290957
NM_005727.3	10103	TSPAN1	1629	85.1528	147.321	-0.79084	0.00165	0.0290957
NM_001953.3	1890	TYMP	1659	9.80753	26.8081	-1.45071	0.00165	0.0290957
NM_014918.4	22856	CHSY1	4550	32.3763	18.9722	0.771056	0.00165	0.0290957
NM_002569.2	5045	FURIN	4180	12.5931	7.13516	0.819615	0.00165	0.0290957
TCONS_00010095	XLOC_000525	-	2053	0.24841	1.20696	-2.28058	0.0017	0.0297668
NM_019604.2	56253	CRTAM	2422	1.68403	4.05827	-1.26895	0.0017	0.0297668
NM_001030059.1	196051	PPAPDC1A	1521	4.88901	1.88413	1.37564	0.0017	0.0297668
NM_018365.2	55329	MNS1	2023	10.8548	25.7587	-1.24673	0.0017	0.0297668
NM_012452.2	23495	TNFRSF13B	1358	0.75124	2.57065	-1.77479	0.0017	0.0297668
NM_001171.5	368	ABCC6	5117	0.391075	1.11204	-1.50769	0.0017	0.0297668
NM_012304.3	23194	FBXL7	4562	5.502	2.93144	0.908346	0.0017	0.0297668
NM_006994.4	10384	BTN3A3	3002	11.4458	21.9908	-0.942074	0.0017	0.0297668
NM_001768.6	925	CD8A	2912	5.89988	12.1851	-1.04636	0.00175	0.0304286
NM_002221.3	3707	ITPKB	6162	12.2899	7.14154	0.78317	0.00175	0.0304286
NM_020066.4	56776	FMN2	6429	5.73956	3.12918	0.875155	0.00175	0.0304286
NM_001142864.2	9780	FAM38A	7833	7.04767	3.99281	0.819742	0.00175	0.0304286
NM_006009.2	7846	TUBA1A	1666	131.023	238.354	-0.86328	0.00175	0.0304286
NM_178837.3	352909	C19orf51	2168	2.62417	5.90632	-1.1704	0.0018	0.0310732
NM_022153.1	64115	C10orf54	4774	20.5515	11.8568	0.793528	0.0018	0.0310732
TCONS_00230998	XLOC_019497	-	1796	0.189354	1.45178	-2.93866	0.0018	0.0310732
NM_006486.2	2192	FBLN1	2947	99.7612	47.6831	1.065	0.0018	0.0310732
NM_020251.3	408	ARRB1	7498	7.38815	3.67359	1.00802	0.0018	0.0310732
NM_016201.2	51421	AMOTL2	4984	20.2505	11.8955	0.767545	0.0018	0.0310732
NM_001080495.2	84629	TNRC18	10572	6.98445	4.14213	0.753774	0.00185	0.0317167
NM_003733.2	8638	OASL	1820	1.89067	4.776	-1.33691	0.00185	0.0317167
NM_198795.1	56165	TDRD1	4510	1.97611	0.829198	1.25287	0.00185	0.0317167
NM_000801.4	2280	FKBP1A	1633	73.5474	42.6422	0.786392	0.00185	0.0317167
NM_006770.3	8685	MARCO	1835	3.21576	7.27374	-1.17754	0.00185	0.0317167
NM_207310.1	91409	CCDC74B	1471	1.97281	4.91142	-1.31589	0.00185	0.0317167
NM_002160.2	3371	TNC	7599	148.976	72.2417	1.04417	0.0019	0.0323187
NM_001004310.2	343413	FCRL6	1943	1.50774	4.07004	-1.43265	0.0019	0.0323187
NM_002205.2	3678	ITGA5	4250	15.2216	8.64847	0.815608	0.0019	0.0323187
NM_001127462.2	114548	NLRP3	4290	5.51492	0.473628	3.54152	0.0019	0.0323187
NM_005456.3	9479	MAPK8IP1	3205	3.54997	7.0587	-0.991595	0.0019	0.0323187
NM_003270.2	7105	TSPAN6	2069	39.2457	65.2166	-0.732704	0.0019	0.0323187
NM_005709.3	10083	USH1C	2237	0.572827	1.89194	-1.7237	0.0019	0.0323187
NM_001128843.1	79931	TNIP3	2429	0.495592	1.58879	-1.68071	0.0019	0.0323187
NM_002859.2	5829	PXN	3672	14.0707	7.02171	1.0028	0.00195	0.0329693

NM_002970.2	6303	SAT1	1064	534.363	1018.91	-0.931136	0.00195	0.0329693
NM_001101357.1	347475	CCDC160	1299	3.02253	7.59403	-1.32911	0.00195	0.0329693
NM_144962.2	157310	PEBP4	894	2.14869	0.342296	2.65014	0.00195	0.0329693
NM_033292.2	834	CASP1	1357	24.5449	44.9829	-0.873953	0.00195	0.0329693
NM_004431.3	1969	EPHA2	3964	9.10294	5.11571	0.831396	0.00195	0.0329693
NM_001353.5	1645	AKR1C1	1379	19.5827	34.7238	-0.82635	0.00195	0.0329693
NM_000899.4	4254	KITLG	5443	54.8666	31.3504	0.807447	0.00195	0.0329693
NM_001144058.1	50863	NTM	3408	4.63404	1.42491	1.7014	0.00195	0.0329693
NM_000424.3	3852	KRT5	2303	234.244	115.543	1.01958	0.00195	0.0329693
NM_003042.3	6529	SLC6A1	4494	1.4566	0.589159	1.30588	0.002	0.0335868
NM_152550.3	153769	SH3RF2	3043	5.96655	2.96621	1.00828	0.002	0.0335868
NM_003667.2	8549	LGR5	2880	1.45608	0.522087	1.47972	0.002	0.0335868
NM_002192.2	3624	INHBA	2175	8.15161	3.92117	1.0558	0.002	0.0335868
NM_005158.4	27	ABL2	11940	9.50476	4.56226	1.0589	0.002	0.0335868
NM_002003.3	2219	FCN1	1283	17.2839	30.9812	-0.841967	0.00205	0.0341961
NM_001162435.2	100287718	LOC100287718	1058	6.02541	13.4932	-1.1631	0.00205	0.0341961
NM_001783.3	973	CD79A	1258	33.8568	62.0491	-0.873964	0.00205	0.0341961
NM_006263.2	5720	PSME1	995	112.535	187.069	-0.733193	0.00205	0.0341961
NM_000733.3	916	CD3E	1513	8.4412	16.3296	-0.951965	0.00205	0.0341961
TCONS_00218840	XLOC_018567	-	2102	1.41085	4.22651	-1.5829	0.00205	0.0341961
NM_182487.2	169611	OLFML2A	6542	11.5504	6.75313	0.774312	0.00205	0.0341961
NM_006765.3	7991	TUSC3	3884	9.62925	19.6205	-1.02687	0.00205	0.0341961
NM_001129993.1	84542	KIAA1841	4578	9.68426	16.7756	-0.792652	0.00205	0.0341961
NM_018956.3	11092	C9orf9	719	8.76507	19.6947	-1.16797	0.00205	0.0341961
TCONS_00229226	XLOC_018711	-	2071	0.254734	1.04324	-2.034	0.0021	0.0348574
NM_052913.2	114801	TMEM200A	5023	12.771	7.34052	0.79892	0.0021	0.0348574
NM_001101376.2	440585	FAM183A	517	14.872	34.1571	-1.19958	0.0021	0.0348574
NM_173799.3	201633	TIGIT	2968	4.52306	8.63215	-0.932422	0.0021	0.0348574
NM_004093.3	1948	EFNB2	4387	11.0504	6.34603	0.800166	0.00215	0.0355296
TCONS_00105654	XLOC_008400	-	3521	1.33169	0.335198	1.99017	0.00215	0.0355296
NM_080927.3	131566	DCBLD2	6093	37.247	22.1248	0.751464	0.00215	0.0355296
NM_198565.1	375387	LRRC33	2465	4.4759	1.98077	1.17611	0.00215	0.0355296
NM_138803.3	130940	CCDC148	2846	2.53612	5.89906	-1.21786	0.00215	0.0355296
NM_173496.3	143098	MPP7	5207	8.74615	14.9401	-0.772473	0.0022	0.0361165
NM_014265.4	10863	ADAM28	3220	63.022	125.277	-0.99119	0.0022	0.0361165
XM_003118518.1	728763	LOC728763	4773	0.625649	1.4462	-1.20885	0.0022	0.0361165
NM_001004354.2	441478	NRARP	2621	7.36206	3.67157	1.00371	0.0022	0.0361165
NM_001098844.1	653567	TMEM236	5464	1.63113	0.718203	1.18341	0.00225	0.0367046
NM_024989.3	80055	PGAP1	11113	11.8049	7.09943	0.73361	0.00225	0.0367046
NM_014600.2	30845	EHD3	3906	9.27172	5.02788	0.882886	0.00225	0.0367046
NM_007350.3	22822	PHLDA1	5913	26.4457	14.7214	0.845114	0.00225	0.0367046
NM_144577.3	93233	CCDC114	3220	2.15727	4.41656	-1.03371	0.00225	0.0367046
NM_001031737.2	124093	CCDC78	1611	6.69397	15.979	-1.25524	0.00225	0.0367046
NM_002334.3	4038	LRP4	8227	2.85108	1.47768	0.948174	0.00225	0.0367046
TCONS_00233526	XLOC_020559	-	4976	2.17561	6.07835	-1.48226	0.00225	0.0367046
NM_002638.3	5266	PI3	554	16.116	36.0191	-1.16027	0.0023	0.0373663
NM_005737.3	10123	ARL4C	4009	27.1432	15.888	0.772659	0.0023	0.0373663
NM_030761.4	54361	WNT4	3905	3.66618	1.6174	1.1806	0.0023	0.0373663
NM_178026.2	2686	GGT7	2640	9.17513	4.77749	0.941476	0.0023	0.0373663
NM_002842.3	5794	PTPRH	3928	0.708407	1.981	-1.48358	0.0023	0.0373663
NM_001100168.1	1234	CCR5	3426	7.5715	13.9056	-0.87702	0.00235	0.0379132
NM_001042517.1	81624	DIAPH3	4802	1.42367	0.438841	1.69785	0.00235	0.0379132
NM_021961.5	7003	TEAD1	9416	37.4153	22.1059	0.759199	0.00235	0.0379132
NM_147175.3	90161	HS6ST2	4447	1.47508	0.563872	1.38736	0.00235	0.0379132

NM_005906.4	4117	MAK	3883	2.38789	5.42875	-1.18488	0.00235	0.0379132
XM_002342862.3	100288562	LOC100288562	2186	0.161353	1.12857	-2.8062	0.00235	0.0379132
NM_004557.3	4855	NOTCH4	6745	4.15963	1.47085	1.49981	0.00235	0.0379132
NM_000859.2	3156	HMGCR	4582	27.5603	16.3859	0.750138	0.00235	0.0379132
NM_006727.3	1008	CDH10	3436	0.245975	0.808953	-1.71754	0.0024	0.03848
NM_001206602.1	1123	CHN1	2294	18.4304	9.52727	0.95195	0.0024	0.03848
NM_198524.1	374618	TEX9	1414	20.4018	43.6845	-1.09843	0.0024	0.03848
NM_002942.4	6092	ROBO2	8689	6.2952	11.0496	-0.811666	0.0024	0.03848
NM_000906.3	4881	NPR1	4185	1.53132	0.647642	1.24151	0.00245	0.039114
NM_004688.2	9111	NMI	1479	18.6303	32.464	-0.801186	0.00245	0.039114
TCONS_00253855	XLOC_021286	-	1437	0.466937	1.90201	-2.02623	0.00245	0.039114
NM_005064.3	6368	CCL23	625	11.2966	0.43818	4.68822	0.00245	0.039114
TCONS_00293640	XLOC_025525	-	6270	0.942857	1.99941	-1.08446	0.0025	0.0397144
NM_182762.3	346389	MACC1	9159	10.892	18.9415	-0.798287	0.0025	0.0397144
NM_152618.2	166379	BBS12	3244	3.99097	7.46331	-0.903075	0.0025	0.0397144
NM_001007237.1	3321	IGSF3	7193	12.3143	7.04323	0.806029	0.0025	0.0397144
TCONS_00282969	XLOC_023780	-	3213	0.921909	2.33462	-1.34049	0.00255	0.0402995
NM_182628.2	348807	CCDC37	2086	3.32923	6.85021	-1.04096	0.00255	0.0402995
NM_014585.5	30061	SLC40A1	3355	56.4694	94.243	-0.738915	0.00255	0.0402995
NM_002864.2	5858	PZP	4610	2.85142	5.29491	-0.892925	0.00255	0.0402995
NM_014734.3	9766	KIAA0247	5393	20.1116	12.1758	0.724014	0.00255	0.0402995
NM_003948.3	8999	CDKL2	4715	0.666814	1.60176	-1.2643	0.0026	0.040888
NM_006927.3	6483	ST3GAL2	4450	7.49506	4.22032	0.828588	0.0026	0.040888
NM_004996.3	4363	ABCC1	6564	12.9786	5.78362	1.16609	0.0026	0.040888
NM_020840.1	57600	FNIP2	5790	16.1555	9.85943	0.712449	0.0026	0.040888
NM_000953.2	5729	PTGDR	2942	3.84594	7.42563	-0.949176	0.0026	0.040888
NM_001163.3	320	APBA1	6584	2.69559	1.38685	0.958787	0.0026	0.040888
NM_181078.2	50615	IL21R	4849	0.554021	1.66878	-1.59078	0.0026	0.040888
NM_021149.2	23406	COTL1	1827	63.6715	38.2875	0.733776	0.00265	0.0415288
NM_207315.2	129607	CMPK2	2989	4.32456	8.05664	-0.897624	0.00265	0.0415288
NM_015668.3	26166	RGS22	4184	9.38407	16.2235	-0.789802	0.00265	0.0415288
NM_001010923.2	387357	THEMIS	3866	5.29007	10.8968	-1.04255	0.00265	0.0415288
NM_000165.3	2697	GJA1	3130	139.442	73.3788	0.926234	0.00265	0.0415288
NM_001845.4	1282	COL4A1	6532	64.9969	36.4203	0.835628	0.00265	0.0415288
NM_001018071.3	143162	FRMPD2	5024	2.91976	5.3588	-0.876058	0.00265	0.0415288
NM_024848.1	79906	MORN1	1641	3.20908	7.16748	-1.15931	0.00265	0.0415288
TCONS_00162172	XLOC_013545	-	4119	1.45705	3.04982	-1.06567	0.0027	0.0421065
NM_020338.3	57178	ZMIZ1	7555	9.65556	5.78874	0.73811	0.0027	0.0421065
NM_001039360.2	201501	ZBTB7C	4171	9.32096	5.19378	0.843694	0.0027	0.0421065
NM_000499.3	1543	CYP1A1	2608	0.804333	0.195615	2.03978	0.0027	0.0421065
NM_001860.2	1318	SLC31A2	1774	11.1569	20.5591	-0.881846	0.00275	0.0426687
NM_000219.3	3753	KCNE1	3321	3.96127	8.12652	-1.03667	0.00275	0.0426687
NM_001024858.2	6710	SPTB	10064	1.02183	0.472656	1.11229	0.00275	0.0426687
NM_001235.3	871	SERPINH1	2276	33.9003	20.2393	0.744137	0.00275	0.0426687
NM_025257.2	80736	SLC44A4	2634	23.7482	39.8072	-0.74521	0.00275	0.0426687
NM_001005852.2	246126	CYorf15A	865	30.3339	12.2601	1.30696	0.00275	0.0426687
NM_031415.2	56169	GSDMC	2714	6.78051	3.47065	0.966185	0.0028	0.0432452
NM_152362.2	126282	TNFAIP8L1	3817	6.40115	11.0429	-0.786715	0.0028	0.0432452
NM_002185.2	3575	IL7R	1805	23.3234	39.8655	-0.773364	0.0028	0.0432452
NM_002304.2	3955	LFNG	2058	9.81878	2.57536	1.93077	0.00285	0.0437663
NM_006666.1	10856	RUVBL2	1488	23.5467	39.3669	-0.741457	0.00285	0.0437663
NM_005257.3	2627	GATA6	3494	2.16634	0.939303	1.20559	0.00285	0.0437663
NM_003247.2	7058	THBS2	5809	60.2475	28.9329	1.05819	0.00285	0.0437663
NM_021804.2	59272	ACE2	3507	0.801197	1.92579	-1.26522	0.00285	0.0437663

NM_002609.3	5159	PDGFRB	5714	52.8063	29.6801	0.831216	0.00285	0.0437663
NM_017576.1	55582	KIF27	4653	13.1358	21.4201	-0.705461	0.00285	0.0437663
NM_001100396.1	129852	C2orf73	1921	1.18375	3.07481	-1.37714	0.00285	0.0437663
NM_000517.4	3040	HBA2	605	10.664	3.43494	1.6344	0.00285	0.0437663
NM_004341.3	790	CAD	7108	5.55905	3.2114	0.791636	0.0029	0.0443924
NM_014767.2	9806	SPOCK2	5329	6.28743	10.7684	-0.776267	0.0029	0.0443924
NM_001145112.1	197135	PATL2	1951	1.94132	4.33907	-1.16035	0.0029	0.0443924
NM_007038.3	11096	ADAMTS5	9663	1.9889	3.5609	-0.840268	0.00295	0.0449329
NM_005932.3	4285	MIPEP	2429	11.4719	19.5843	-0.771597	0.00295	0.0449329
NM_138770.1	90557	CCDC74A	1457	4.02284	8.61669	-1.09892	0.00295	0.0449329
NM_030820.3	81578	COL21A1	4173	4.14476	7.64131	-0.882531	0.00295	0.0449329
NM_152628.3	169166	SNX31	2457	1.96772	0.637447	1.62615	0.003	0.0454886
NM_152572.2	158067	AK8	2106	2.24374	4.83351	-1.10717	0.003	0.0454886
NM_201526.1	3671	ISLR	2111	22.2926	11.1724	0.996621	0.003	0.0454886
NM_172069.3	130271	PLEKHH2	6954	10.0027	6.13902	0.704304	0.003	0.0454886
NM_178565.4	340419	RSPO2	3149	0.0520553	2.6254	-5.65635	0.00305	0.04606
NM_012293.1	7837	PXDN	6821	12.625	7.4627	0.75851	0.00305	0.04606
NM_138812.2	143241	DYDC1	750	3.545	9.47133	-1.41778	0.00305	0.04606
NM_152588.1	160335	TMTC2	4840	48.6985	29.6731	0.714723	0.00305	0.04606
NM_000100.2	1476	CSTB	658	71.8356	122.62	-0.771421	0.00305	0.04606
NM_018438.5	26270	FBXO6	1551	4.2878	8.73924	-1.02727	0.00305	0.04606
NM_002818.2	5721	PSME2	829	84.6246	139.51	-0.721222	0.0031	0.0465955
NM_020796.3	57556	SEMA6A	6860	6.81346	3.99973	0.768485	0.0031	0.0465955
NM_020877.2	146754	DNAH2	13505	3.60199	5.95256	-0.724718	0.0031	0.0465955
NM_018286.2	55273	TMEM100	1755	11.2743	20.2749	-0.846664	0.00315	0.0471261
NM_016118.3	51667	NUB1	3107	27.8474	45.1508	-0.69721	0.00315	0.0471261
NM_148962.4	165140	OXER1	1763	2.3293	0.854166	1.44731	0.00315	0.0471261
NM_021179.1	57821	C1orf114	1866	5.01895	20.4473	-2.02645	0.00315	0.0471261
NM_001142946.1	29125	C11orf21	2262	4.95623	2.33754	1.08426	0.00315	0.0471261
NM_181780.3	151888	BTLA	3215	1.85134	4.18572	-1.17691	0.00315	0.0471261
TCONS_00314309	XLOC_026611	-	1578	2.22171	0.297276	2.90179	0.00315	0.0471261
NM_002518.3	4862	NPAS2	4004	4.09487	2.13194	0.941654	0.0032	0.0476939
NM_001144960.1	389161	ANKUB1	2476	10.4254	17.9464	-0.783587	0.0032	0.0476939
NM_032821.2	54768	HYDIN	15685	3.15632	5.70957	-0.855141	0.0032	0.0476939
NM_001098843.3	645090	CXorf30	2863	3.5013	6.73563	-0.943923	0.0032	0.0476939
NM_001333.3	1515	CTSL2	4527	3.73219	1.85689	1.00713	0.0032	0.0476939
NM_001629.3	241	ALOX5AP	906	48.3052	27.1364	0.831946	0.00325	0.0483534
TCONS_00103332	XLOC_008749	-	1106	1.70866	0.175463	3.28363	0.00325	0.0483534
NM_153498.2	57118	CAMK1D	2242	15.4831	7.62907	1.02112	0.00325	0.0483534
NM_001130040.1	6464	SHC1	3481	19.821	7.86407	1.33368	0.00325	0.0483534
NM_182906.2	10462	CLEC10A	1771	16.2476	7.19449	1.17526	0.0033	0.0489028
NM_000073.2	917	CD3G	1311	5.76632	11.9662	-1.05324	0.0033	0.0489028
NM_080667.5	112942	CCDC104	1299	27.5693	47.0665	-0.771637	0.0033	0.0489028
NM_001012502.2	286207	C9orf117	1722	5.48466	11.108	-1.01813	0.0033	0.0489028
NM_014358.2	26253	CLEC4E	2158	0.780655	2.06548	-1.40372	0.0033	0.0489028
NM_016523.1	51348	KLRF1	1242	2.10548	5.05444	-1.2634	0.0033	0.0489028
NM_003245.3	7053	TGM3	2677	0.373215	1.09897	-1.55807	0.0033	0.0489028
NM_001079878.1	1261	CNGA3	3794	0.241541	0.738554	-1.61244	0.00335	0.0494369
NM_000328.2	6103	RPGR	3072	8.51408	15.6435	-0.877637	0.00335	0.0494369
TCONS_00019022	XLOC_000467	-	2384	3.05583	1.29656	1.23687	0.00335	0.0494369

Table S6. The common dysregulated protein-coding genes of ECRSwNP *vs* CTRL and non-ECRSwNP *vs* CTRL.

Transcript ID	Gene ID	Gene name	Length	ECRSwNP FPKM	non-ECRSwNP FPKM	Ctrl FPKM	ECRSwNP vs Ctrl log_2(foldchange)	ECRSwNP vs Ctrl P value	ECRSwNP vs Ctrl Q value	non-ECRSwNP vs Ctrl log_2(foldchange)	non-ECRSwNP vs Ctrl P value	non-ECRSwNP vs Ctrl Q value
NM_004131.4	3002	GZMB	927	8.68444	15.4069	3.68794	1.23562	0.00335	0.0494369	2.06269	5.00E-05	0.00170056
NM_001172624.1	4756	NEO1	7055	12.1324	11.2077	20.2755	-0.740875	0.00335	0.0494369	-0.855251	0.00085	0.0176689
NM_003525.2	8346	HIST1H2BI	437	55.8263	60.0578	27.0655	1.04449	0.00335	0.0494369	1.1499	0.0013	0.0244278
NM_018990.3	54440	SASH3	2694	13.8878	15.6595	7.6961	0.851622	0.00335	0.0494369	1.02484	0.00065	0.0142695
NM_001113490.1	154796	AMOT	6945	6.57497	6.06735	12.2304	-0.895413	0.0032	0.0476939	-1.01133	0.00055	0.0124813
NM_001039350.1	1804	DPP6	4705	1.16702	0.414929	3.57046	-1.61328	0.00315	0.0471261	-3.10517	5.00E-05	0.00170056
NM_003013.2	6423	SFRP2	1990	139.326	214.651	83.2395	0.743124	0.0031	0.0465955	1.36665	5.00E-05	0.00170056
NM_004655.3	8313	AXIN2	4234	12.4475	7.79652	20.4522	-0.7164	0.0031	0.0465955	-1.39135	5.00E-05	0.00170056
NM_002610.3	5163	PDK1	4576	34.0586	43.9574	9.17787	1.89179	0.00305	0.04606	2.25988	0.0006	0.013408
NM_020747.2	57507	ZNF608	5645	8.19366	7.90934	13.5527	-0.726	0.00305	0.04606	-0.776951	0.00185	0.0317167
NM_001001872.2	145407	C14orf37	3082	4.74715	4.22888	8.73804	-0.880247	0.00295	0.0449329	-1.04703	0.00075	0.0159498
NM_001199622.1	135112	NCOA7	4052	27.0207	36.6319	3.32381	3.02315	0.0029	0.0443924	3.46219	0.00105	0.0207193
NM_024324.3	79174	CRELD2	1428	19.9681	21.7835	10.57	0.917727	0.00285	0.0437663	1.04327	0.0007	0.015106
NM_001172626.1	270	AMPD1	2395	5.45253	6.33604	1.01457	2.42606	0.00275	0.0426687	2.64271	0.0017	0.0297668
NM_198391.2	23767	FLRT3	4024	4.14387	2.97372	11.3487	-1.45348	0.0027	0.0421065	-1.93219	0.0003	0.00772176
NM_015932.5	51371	POMP	1462	43.8595	51.127	25.9147	0.759119	0.0027	0.0421065	0.980316	0.00015	0.00437065
NM_000057.2	641	BLM	4528	3.41435	3.50817	1.66242	1.03833	0.00265	0.0415288	1.07744	0.00205	0.0341961
NM_001775.2	952	CD38	1491	23.3754	28.9395	12.6906	0.88123	0.00255	0.0402995	1.18928	0.0002	0.00554203
NM_005380.6	4681	NBL1	2026	89.8563	88.0353	166.887	-0.893183	0.00255	0.0402995	-0.92272	0.0015	0.0270821
NM_002526.3	4907	NT5E	4068	16.6419	15.2516	27.6155	-0.730656	0.00255	0.0402995	-0.856522	0.00045	0.0106989
NM_003937.2	8942	KYNU	1672	16.7319	25.4138	6.80954	1.29697	0.00245	0.039114	1.89998	0.0001	0.00312189
NM_145018.3	220042	C11orf82	3565	2.03961	2.00233	0.856252	1.25218	0.0024	0.03848	1.22557	0.00295	0.0449329
NM_001793.4	1001	CDH3	4276	15.7556	16.3987	8.94233	0.817145	0.00235	0.0379132	0.874856	0.00055	0.0124813
NM_006843.2	10993	SDS	1605	1.22725	1.38941	0.322729	1.92704	0.00235	0.0379132	2.10607	0.00105	0.0207193
NM_006178.3	4905	NSF	3983	29.5909	31.1925	18.1373	0.706195	0.0023	0.0373663	0.782241	0.0013	0.0244278
NM_001031709.2	55328	RNLS	2416	5.5012	5.18267	11.2693	-1.03457	0.0022	0.0361165	-1.12063	0.00075	0.0159498
NM_002178.2	3489	IGFBP6	966	45.0021	26.0181	77.1555	-0.777777	0.00215	0.0355296	-1.56825	5.00E-05	0.00170056
NM_002117.4	3107	HLA-C	1525	271.276	386.143	154.551	0.811674	0.0021	0.0348574	1.32105	5.00E-05	0.00170056
NM_004255.3	9377	COX5A	770	44.426	44.4846	23.8837	0.895378	0.0021	0.0348574	0.897277	0.0021	0.0348574
NM_001025266.1	285382	C3orf70	5901	4.42686	4.33434	2.2555	0.972838	0.0021	0.0348574	0.942364	0.0029	0.0443924
NM_014752.2	9789	SPCS2	2708	20.437	22.2905	11.7304	0.800929	0.0019	0.0323187	0.92617	0.00035	0.00869612
NM_052909.3	153478	PLEKHG4B	11515	0.604714	0.513103	1.24942	-1.04693	0.0019	0.0323187	-1.28394	0.00025	0.00665625
NM_207361.4	341640	FREM2	16163	3.21662	3.11999	5.68093	-0.82058	0.00185	0.0317167	-0.864583	0.00065	0.0142695
NM_000063.4	717	C2	2838	15.8909	21.2954	7.39082	1.1044	0.0018	0.0310732	1.52673	5.00E-05	0.00170056
NM_014302.3	23480	SEC61G	466	140.836	180.896	77.2675	0.866086	0.0018	0.0310732	1.22723	5.00E-05	0.00170056
NM_144650.2	137872	ADHFE1	1895	2.5768	2.20059	5.88896	-1.19244	0.0018	0.0310732	-1.42013	0.00025	0.00665625
NM_145287.3	162655	ZNF519	6810	2.33647	1.98114	4.32797	-0.889361	0.0018	0.0310732	-1.12736	0.00035	0.00869612
NM_175862.4	942	CD86	2727	13.1315	12.8441	6.90471	0.92738	0.00175	0.0304286	0.895448	0.0028	0.0432452
NM_033119.4	85407	NKD1	8716	4.78997	3.15174	8.38439	-0.807689	0.00175	0.0304286	-1.41156	5.00E-05	0.00170056
NM_001004067.3	408050	NOMO3	4316	2.47637	2.37045	0.990041	1.32267	0.00175	0.0304286	1.2596	0.0025	0.0397144
NM_001882.3	1393	CRHBP	1838	0.784935	0.779504	2.22215	-1.50131	0.0017	0.0297668	-1.51133	0.003	0.0454886
NM_001242908.1	284654	RSPO1	2910	6.16402	5.62266	12.2863	-0.995101	0.0017	0.0297668	-1.12772	0.00055	0.0124813
NM_000201.2	3383	ICAM1	3246	12.0421	11.6094	6.61486	0.864301	0.00165	0.0290957	0.811511	0.00285	0.0437663
NM_003332.3	7305	TYROBP	591	29.4854	33.9205	11.7458	1.32786	0.00165	0.0290957	1.53002	0.00025	0.00665625
NM_001080444.1	150084	IGSF5	2066	0.115253	0.186116	0.907261	-2.97671	0.00165	0.0290957	-2.28531	0.00185	0.0317167
NM_001025598.1	257106	ARHGAP30	4425	15.2027	15.7537	8.74023	0.798581	0.00165	0.0290957	0.849952	0.00065	0.0142695
NM_001678.3	482	ATP1B2	3332	0.995796	0.762437	2.46752	-1.30914	0.0016	0.0284826	-1.69437	0.0001	0.00312189
NM_021064.4	8969	HIST1H2AG	498	59.9713	66.2763	29.175	1.03954	0.0016	0.0284826	1.18376	0.00055	0.0124813
NM_032777.9	25960	GPR124	6034	14.0118	10.73	24.1094	-0.782951	0.0016	0.0284826	-1.16795	5.00E-05	0.00170056

NM_018102.3	55713	ZNF334	2455	3.58018	3.90401	8.65895	-1.27416	0.0016	0.0284826	-1.14923	0.0025	0.0397144
NM_023073.3	65250	C5orf42	11199	19.4587	18.5595	33.5119	-0.784261	0.0016	0.0284826	-0.852517	0.00095	0.0192772
NM_004836.5	9451	EIF2AK3	4648	32.2178	32.7705	19.1087	0.753634	0.00155	0.0278206	0.778173	0.00105	0.0207193
NM_030627.2	80315	CPEB4	7769	29.6778	32.7505	17.4491	0.766235	0.0015	0.0270821	0.908368	0.0002	0.00554203
NM_002381.4	4148	MATN3	2583	3.62209	1.41571	7.7199	-1.09176	0.00145	0.0264203	-2.44706	5.00E-05	0.00170056
NM_004227.3	9265	CYTH3	4469	14.1338	11.8152	23.9407	-0.760316	0.00145	0.0264203	-1.01882	5.00E-05	0.00170056
NM_006033.2	9388	LIPG	4141	0.922035	0.876609	0.292751	1.65515	0.00145	0.0264203	1.58226	0.002	0.0335868
NM_001112718.1	22998	LIMCH1	6084	12.8545	12.719	24.3684	-0.922737	0.00145	0.0264203	-0.938024	0.0002	0.00554203
NM_013358.2	29943	PADI1	3847	2.10339	2.86203	0.842389	1.32016	0.00145	0.0264203	1.76448	5.00E-05	0.00170056
NM_181644.4	148808	MFSD4	4088	8.48492	8.60961	4.50309	0.913987	0.0014	0.0257815	0.935034	0.0008	0.0168217
NM_019111.4	3122	HLA-DRA	1280	1019.83	1499.11	472.152	1.111	0.00135	0.0250595	1.66678	0.0001	0.00312189
NM_145239.2	112476	PRRT2	2567	1.63494	1.48518	3.78784	-1.21214	0.00135	0.0250595	-1.35074	0.0011	0.0214605
XM_003118524.1	389197	C4orf50	3184	0.540962	0.533624	1.56884	-1.5361	0.00135	0.0250595	-1.5558	0.001	0.0199964
NM_006010.4	7873	MANF	939	54.7061	68.3469	30.5012	0.842833	0.0013	0.0244278	1.16401	5.00E-05	0.00170056
NM_178172.3	338328	GPIHBP1	2281	1.90679	1.95069	4.58965	-1.26724	0.0013	0.0244278	-1.2344	0.00145	0.0264203
NM_178554.4	339855	KY	5704	0.338184	0.341729	0.912367	-1.43181	0.0013	0.0244278	-1.41676	0.0024	0.03848
NM_002030.3	2359	FPR3	2517	24.1341	26.7632	13.4726	0.841044	0.00125	0.02372	0.990223	0.0001	0.00312189
NM_002970.2	6303	SAT1	1064	534.363	1018.91	294.105	0.861489	0.00125	0.02372	1.79263	5.00E-05	0.00170056
NM_005079.2	7163	TPD52	3961	27.0348	32.4916	14.9664	0.853092	0.00125	0.02372	1.11834	0.0001	0.00312189
NM_003329.2	7295	TXN	508	366.501	349.612	206.195	0.829806	0.00125	0.02372	0.76174	0.0011	0.0214605
NM_003793.3	8722	CTSF	2014	19.3251	16.1762	34.152	-0.821496	0.00125	0.02372	-1.0781	5.00E-05	0.00170056
NM_000379.3	7498	XDH	5717	3.00296	6.25099	1.36851	1.13378	0.0012	0.0230244	2.19148	5.00E-05	0.00170056
NM_001135733.1	94241	TP53INP1	5652	49.9421	52.3853	23.9929	1.05765	0.0012	0.0230244	1.12655	0.0007	0.015106
NM_006914.3	6096	RORB	3604	6.01489	4.13765	11.2635	-0.905046	0.00115	0.0222874	-1.44478	5.00E-05	0.00170056
NM_007210.3	11226	GALNT6	4520	6.91363	6.94078	3.5889	0.945901	0.00115	0.0222874	0.951555	0.00135	0.0250595
NM_032679.2	84765	ZNF577	3096	6.02419	5.49141	11.5661	-0.941061	0.00115	0.0222874	-1.07465	0.0005	0.0116249
NM_001198689.1	3953	LEPR	5100	13.3861	16.5626	64.7144	-2.27335	0.0011	0.0214605	-1.96616	0.00185	0.0317167
NM_001085384.1	7710	ZNF154	2747	5.11825	3.6318	10.2001	-0.994856	0.0011	0.0214605	-1.48982	5.00E-05	0.00170056
NM_020203.3	56955	MEPE	2007	0.36773	0.568371	1.66713	-2.18065	0.0011	0.0214605	-1.55246	0.0026	0.040888
NM_000405.4	2760	GM2A	3690	33.4896	33.2595	19.6752	0.767332	0.00105	0.0207193	0.757386	0.0015	0.0270821
NM_000231.2	6445	SGCG	1655	0.160525	0.216968	1.60134	-3.31841	0.00105	0.0207193	-2.88372	0.0005	0.0116249
NM_014584.1	30001	ERO1L	3310	49.0854	62.8114	28.498	0.784432	0.00105	0.0207193	1.14016	5.00E-05	0.00170056
NM_001129908.2	729085	FAM198A	3433	1.63022	1.54762	3.76574	-1.20787	0.00105	0.0207193	-1.28288	0.00055	0.0124813
NM_003511.2	8332	HIST1H2AL	470	24.9788	25.4854	8.03838	1.63573	0.001	0.0199964	1.66469	0.0009	0.0184743
NM_006082.2	10376	TUBA1B	1753	145.728	140.646	84.1687	0.79192	0.001	0.0199964	0.740715	0.002	0.0335868
NM_006808.2	10952	SEC61B	562	79.7751	82.3147	41.3159	0.949242	0.001	0.0199964	0.994454	0.00065	0.0142695
NM_018058.5	55118	CRTAC1	2713	0.154487	0.293499	2.46175	-3.99413	0.001	0.0199964	-3.06826	0.00115	0.0222874
NM_025079.2	80149	ZC3H12A	2716	5.504	6.66306	2.36571	1.21821	0.001	0.0199964	1.49391	0.0001	0.00312189
NM_144616.3	126306	JSRP1	1145	4.94652	6.16069	1.56697	1.65843	0.001	0.0199964	1.97511	5.00E-05	0.00170056
NM_032991.2	836	CASP3	2506	38.8253	41.8237	19.9246	0.962443	0.00095	0.0192772	1.06977	0.0003	0.00772176
NM_002965.3	6280	S100A9	577	31.6324	86.7818	11.6548	1.44047	0.00095	0.0192772	2.89646	5.00E-05	0.00170056
NM_138554.3	7099	TLR4	5653	27.102	29.0756	15.526	0.803715	0.00095	0.0192772	0.905122	0.0002	0.00554203
NM_152547.4	153579	BTNL9	3500	2.02779	1.6415	4.53135	-1.16003	0.00095	0.0192772	-1.46493	0.00015	0.00437065
NM_004967.3	3381	IBSP	1591	0.127051	0.283272	22.5586	-7.47213	0.0009	0.0184743	-6.31535	5.00E-05	0.00170056
NM_000918.3	5034	P4HB	2578	112.846	105.702	63.2556	0.835092	0.00085	0.0176689	0.740736	0.0021	0.0348574
NM_005719.2	10094	ARPC3	884	240.933	237.652	137.249	0.811836	0.00085	0.0176689	0.792055	0.00125	0.02372
NM_020813.2	57573	ZNF471	4967	4.51928	4.45557	8.26025	-0.870093	0.00085	0.0176689	-0.890575	0.001	0.0199964
NM_001290.3	9079	LDB2	2540	25.9535	28.1854	54.161	-1.06133	0.0008	0.0168217	-0.942304	0.0018	0.0310732
NM_152862.1	10109	ARPC2	1448	177.541	178.896	102.67	0.790134	0.0008	0.0168217	0.801107	0.00095	0.0192772
NM_173535.2	165530	CLEC4F	2475	0.916906	0.774704	2.46907	-1.42912	0.0008	0.0168217	-1.67225	0.0008	0.0168217
NM_177455.3	168620	BHLHA15	706	6.34898	6.56593	1.9044	1.73719	0.0008	0.0168217	1.78567	0.0008	0.0168217
NM_019016.2	192666	KRT24	1881	1.6991	1.71115	0.170365	3.31808	0.0008	0.0168217	3.32826	0.0007	0.015106
NM_002298.4	3936	LCP1	3790	105.542	105.497	54.3309	0.957976	0.00075	0.0159498	0.957356	0.0009	0.0184743
NM_005408.2	6357	CCL13	851	168.111	2.65448	0.321955	9.02834	0.00075	0.0159498	3.0435	0.0022	0.0361165

NM_007107.3	6747	SSR3	3716	86.4506	83.7081	48.0381	0.847699	0.00075	0.0159498	0.80119	0.00095	0.0192772
NM_000720.2	776	CACNA1D	7768	3.50119	2.20781	8.05376	-1.20182	0.0007	0.015106	-1.86704	5.00E-05	0.00170056
NM_002090.2	2921	CXCL3	1159	2.09477	4.80877	0.313661	2.73951	0.0007	0.015106	3.93839	5.00E-05	0.00170056
NM_000929.2	5322	PLA2G5	1894	0.164039	0.413318	1.32441	-3.01324	0.0007	0.015106	-1.68003	0.0025	0.0397144
NM_004864.2	9518	GDF15	1200	2.14643	1.76635	0.431044	2.31603	0.0007	0.015106	2.03486	0.0018	0.0310732
NM_021647.6	9848	MFAP3L	6189	2.45327	1.97214	5.02428	-1.03421	0.0007	0.015106	-1.34915	0.0004	0.00971957
NM_005022.2	5216	PFN1	807	183.725	179.468	106.079	0.792411	0.00065	0.0142695	0.758591	0.00135	0.0250595
NM_003026.2	6456	SH3GL2	2728	0.0779284	0.176579	1.60812	-4.36709	0.00065	0.0142695	-3.18699	5.00E-05	0.00170056
NM_006120.3	3108	HLA-DMA	1122	71.571	105.769	38.8935	0.879847	0.0006	0.013408	1.44331	5.00E-05	0.00170056
NM_006982.2	8092	ALX1	1294	1.59448	1.12406	4.80268	-1.59076	0.0006	0.013408	-2.09512	0.0001	0.00312189
NM_018699.2	11107	PRDM5	2480	10.4146	8.92824	20.0417	-0.944402	0.0006	0.013408	-1.16656	5.00E-05	0.00170056
NM_080489.4	27111	SDCBP2	1581	4.48281	4.17642	1.00872	2.15187	0.0006	0.013408	2.04974	0.00065	0.0142695
NM_014618.2	1620	DBC1	3196	4.26732	3.7226	1.64308	1.37693	0.00055	0.0124813	1.17991	0.0027	0.0421065
NM_004961.3	2564	GABRE	3152	2.83253	3.25493	6.26439	-1.14508	0.00055	0.0124813	-0.944549	0.0028	0.0432452
NM_017551.2	2894	GRID1	5849	1.36616	1.07676	3.10595	-1.18491	0.00055	0.0124813	-1.52834	5.00E-05	0.00170056
NM_002118.4	3109	HLA-DMB	1397	58.0641	93.8486	31.0085	0.904982	0.00055	0.0124813	1.59767	5.00E-05	0.00170056
NM_002372.2	4124	MAN2A1	5128	32.7002	30.9774	18.5905	0.814736	0.00055	0.0124813	0.736651	0.00185	0.0317167
NM_002951.3	6185	RPN2	2508	95.2731	94.0982	52.148	0.869457	0.00055	0.0124813	0.851556	0.00055	0.0124813
NM_014256.3	10331	B3GNT3	2720	4.72058	6.00884	1.93042	1.29005	0.00055	0.0124813	1.63818	5.00E-05	0.00170056
NM_015000.3	23012	STK38L	5098	31.3333	28.6834	17.5419	0.836895	0.00055	0.0124813	0.709413	0.00295	0.0449329
NM_031311.3	54504	CPVL	1691	54.4294	70.0336	29.4203	0.887575	0.00055	0.0124813	1.25124	5.00E-05	0.00170056
NM_021181.3	57823	SLAMF7	2657	27.8412	50.9278	14.606	0.930661	0.00055	0.0124813	1.80189	5.00E-05	0.00170056
NM_020980.3	366	AQP9	3021	3.48455	5.48304	1.30761	1.41404	0.0005	0.0116249	2.06805	5.00E-05	0.00170056
NM_000775.2	1573	CYP2J2	1857	12.5142	10.2191	25.523	-1.02823	0.0005	0.0116249	-1.32053	5.00E-05	0.00170056
NM_000474.3	7291	TWIST1	1666	22.5829	19.1523	42.654	-0.91745	0.0005	0.0116249	-1.15516	5.00E-05	0.00170056
NM_016021.2	51465	UBE2J1	4342	57.9104	62.4642	31.4501	0.880758	0.0005	0.0116249	0.989968	0.00015	0.00437065
NM_178568.2	146760	RTN4RL1	3164	0.702349	0.922589	2.17195	-1.62873	0.0005	0.0116249	-1.23523	0.00325	0.0483534
NM_001623.3	199	AIF1	639	68.8854	89.5452	32.3159	1.09195	0.00045	0.0106989	1.47037	5.00E-05	0.00170056
NM_001005738.1	2358	FPR2	1913	2.70072	4.1006	0.818838	1.72169	0.00045	0.0106989	2.32418	5.00E-05	0.00170056
NM_000441.1	5172	SLC26A4	4930	2.74147	23.743	1.16855	1.23023	0.00045	0.0106989	4.34471	5.00E-05	0.00170056
NM_003446.3	7712	ZNF157	1695	1.71162	2.12303	4.81562	-1.49236	0.00045	0.0106989	-1.1816	0.00275	0.0426687
NM_013231.4	23768	FLRT2	7179	13.521	10.6433	25.2854	-0.903105	0.00045	0.0106989	-1.24836	5.00E-05	0.00170056
NM_021237.3	58515	SELK	818	54.368	60.4969	26.596	1.03155	0.00045	0.0106989	1.18565	5.00E-05	0.00170056
NM_022475.2	64399	HHIP	3555	2.48985	3.08747	0.89534	1.47555	0.00045	0.0106989	1.78592	5.00E-05	0.00170056
NM_138780.2	94122	SYTL5	4726	4.16459	4.09938	1.08603	1.93911	0.00045	0.0106989	1.91634	0.00055	0.0124813
NM_153267.4	256691	MAMDC2	3618	5.04728	5.48322	10.0174	-0.988932	0.00045	0.0106989	-0.869417	0.0031	0.0465955
NM_001995.2	2180	ACSL1	3805	28.196	27.8277	15.5685	0.856866	0.0004	0.00971957	0.837898	0.00035	0.00869612
NM_001118890.1	2745	GLRX	1079	30.1375	32.0612	13.2694	1.18346	0.0004	0.00971957	1.27273	0.0001	0.00312189
3507	3507	-	1486	135.167	322.04	61.5317	1.13534	0.0004	0.00971957	2.38784	5.00E-05	0.00170056
NM_002627.4	5214	PFKP	2657	24.4175	24.1835	13.2778	0.878897	0.0004	0.00971957	0.865005	0.0006	0.013408
NM_006516.2	6513	SLC2A1	3670	16.4512	14.7054	8.73311	0.913621	0.0004	0.00971957	0.751778	0.00285	0.0437663
NM_000343.3	6523	SLC5A1	5061	2.30198	4.11193	0.800905	1.52317	0.0004	0.00971957	2.36011	5.00E-05	0.00170056
NM_003816.2	8754	ADAM9	4099	76.7869	67.2931	41.2413	0.896772	0.0004	0.00971957	0.70637	0.0033	0.0489028
NM_130386.2	81035	COLEC12	3134	31.4666	28.7509	57.4232	-0.867811	0.0004	0.00971957	-0.998026	5.00E-05	0.00170056
NM_182658.1	359710	C20orf185	1454	12.1176	14.108	3.4163	1.82659	0.0004	0.00971957	2.04601	5.00E-05	0.00170056
NM_001192.2	608	TNFRSF17	994	18.0928	21.6875	7.18924	1.33151	0.00035	0.00869612	1.59295	5.00E-05	0.00170056
NM_001025195.1	1066	CES1	2013	22.3099	22.0134	56.7756	-1.34758	0.00035	0.00869612	-1.36689	0.00125	0.02372
NM_003383.3	7436	VLDLR	3636	3.39324	1.9108	13.4397	-1.98577	0.00035	0.00869612	-2.81425	5.00E-05	0.00170056
NM_003509.2	8329	HIST1H2AI	469	19.511	19.7312	4.52326	2.10885	0.00035	0.00869612	2.12505	0.00035	0.00869612
NM_003564.1	8407	TAGLN2	1360	136.581	130.903	73.5734	0.892502	0.00035	0.00869612	0.831245	0.00065	0.0142695
NM_004675.2	9077	DIRAS3	1631	7.05424	10.8335	2.58486	1.4484	0.00035	0.00869612	2.06734	5.00E-05	0.00170056
NM_005100.3	9590	AKAP12	8434	19.1537	21.3303	47.4702	-1.3094	0.00035	0.00869612	-1.15412	0.0013	0.0244278
NM_001128227.2	10020	GNE	5298	18.2042	18.5581	7.74155	1.23358	0.00035	0.00869612	1.26136	0.00155	0.0278206
NM_007356.2	22798	LAMB4	5858	1.02813	1.11046	0.274688	1.90415	0.00035	0.00869612	2.01529	0.00015	0.00437065

NM_015364.4	23643	LY96	622	31.0977	42.3746	11.2909	1.46165	0.00035	0.00869612	1.90804	5.00E-05	0.00170056
NM_014331.3	23657	SLC7A11	9648	1.93159	2.45937	0.862837	1.16263	0.00035	0.00869612	1.51113	5.00E-05	0.00170056
NM_017424.2	51816	CECR1	3927	23.899	23.5747	9.39168	1.34749	0.00035	0.00869612	1.32778	0.0005	0.0116249
NM_145739.2	114880	OSBPL6	6811	1.05267	2.65405	7.64767	-2.86096	0.00035	0.00869612	-1.52683	0.00035	0.00869612
NM_152793.2	222166	C7orf41	5814	16.2493	17.0546	29.7241	-0.871254	0.00035	0.00869612	-0.801472	0.001	0.0199964
NM_000478.4	249	ALPL	2596	4.1073	3.94506	9.76129	-1.24888	0.0003	0.00772176	-1.30702	5.00E-05	0.00170056
NM_001175.4	397	ARHGDIB	1216	150.498	145.249	79.4677	0.921303	0.0003	0.00772176	0.870092	0.00035	0.00869612
NM_000059.3	675	BRCA2	11386	5.26711	5.66493	2.76446	0.930012	0.0003	0.00772176	1.03506	5.00E-05	0.00170056
NM_001199149.1	4057	LTF	2508	243.987	485.255	102.844	1.24635	0.0003	0.00772176	2.23829	0.0001	0.00312189
NM_003006.4	6404	SELPLG	2573	15.6738	14.2582	7.42515	1.07787	0.0003	0.00772176	0.941299	0.00095	0.0192772
NM_006367.3	10487	CAP1	2773	98.7364	83.5729	44.2391	1.15826	0.0003	0.00772176	0.917714	0.00185	0.0317167
NM_007191.4	11197	WIF1	2240	0.104994	0.430398	3.76249	-5.16331	0.0003	0.00772176	-3.12794	5.00E-05	0.00170056
NM_015900.3	51365	PLA1A	1780	10.9356	11.4027	3.08154	1.82731	0.0003	0.00772176	1.88765	0.0002	0.00554203
NM_016623.3	51571	FAM49B	2196	41.3185	38.1114	21.7082	0.928544	0.0003	0.00772176	0.811981	0.0015	0.0270821
NM_001160130.1	56479	KCNQ5	6556	0.71944	0.604168	1.97105	-1.45402	0.0003	0.00772176	-1.70594	5.00E-05	0.00170056
XM_003403521.1	100652860	LOC10065286	1520	2.63232	3.23298	7.85582	-1.57743	0.0003	0.00772176	-1.2809	0.00105	0.0207193
NM_032977.3	843	CASP10	5906	8.93825	9.55204	4.33585	1.04368	0.00025	0.00665625	1.13949	5.00E-05	0.00170056
NM_001289.4	1193	CLIC2	2686	17.9547	17.2866	9.31415	0.946865	0.00025	0.00665625	0.89216	0.00065	0.0142695
NM_005508.4	1233	CCR4	1657	8.70532	7.70633	3.11228	1.48393	0.00025	0.00665625	1.30807	0.00095	0.0192772
NM_000847.4	2940	GSTA3	915	0.650144	1.26298	4.12801	-2.66661	0.00025	0.00665625	-1.70862	0.00125	0.02372
NM_001097589.1	6707	SPRR3	902	5.92007	3.49682	0.327627	4.17549	0.00025	0.00665625	3.41592	0.00225	0.0367046
NM_014708.4	9735	KNTC1	6965	13.1954	14.2564	7.32504	0.849129	0.00025	0.00665625	0.960702	5.00E-05	0.00170056
NM_016235.1	51704	GPRC5B	2845	27.1547	24.6947	50.93	-0.907312	0.00025	0.00665625	-1.04432	5.00E-05	0.00170056
NM_017982.3	55061	SUSD4	3027	2.39564	2.82254	6.00956	-1.32685	0.00025	0.00665625	-1.09027	0.00225	0.0367046
NM_020848.2	57608	KIAA1462	9300	11.7107	6.01114	21.0118	-0.843376	0.00025	0.00665625	-1.80549	5.00E-05	0.00170056
NM_024817.2	79875	THSD4	9145	24.1337	17.6737	46.0633	-0.932568	0.00025	0.00665625	-1.38202	5.00E-05	0.00170056
NM_032744.3	84830	C6orf105	1804	5.39781	5.54516	1.50068	1.84676	0.00025	0.00665625	1.88562	0.00025	0.00665625
NM_152864.3	128414	NKAIN4	1430	0.489232	0.66951	2.45336	-2.32617	0.00025	0.00665625	-1.87358	0.00055	0.0124813
NM_153274.2	266675	BEST4	2096	0.959201	1.20193	3.22982	-1.75155	0.00025	0.00665625	-1.4261	0.0008	0.0168217
NM_001204376.1	4883	NPR3	6380	4.74809	5.73845	1.65885	1.51716	0.0002	0.00554203	1.79048	0.0001	0.00312189
NM_002530.2	4916	NTRK3	2818	1.93064	1.87484	8.48226	-2.13537	0.0002	0.00554203	-1.82049	5.00E-05	0.00170056
NM_005065.4	6400	SEL1L	6579	81.8322	100.92	40.679	1.00838	0.0002	0.00554203	1.31086	5.00E-05	0.00170056
NM_003039.2	6518	SLC2A5	2438	2.47123	3.48966	0.667059	1.88935	0.0002	0.00554203	2.3872	5.00E-05	0.00170056
NM_004710.3	9144	SYNGR2	1694	27.0738	28.3693	13.3931	1.01541	0.0002	0.00554203	1.08284	5.00E-05	0.00170056
NM_012081.5	22936	ELL2	6046	47.9627	43.621	24.7787	0.952811	0.0002	0.00554203	0.815919	0.00095	0.0192772
NM_031912.4	83849	SYT15	5508	6.15942	6.28864	11.8717	-0.946654	0.0002	0.00554203	-0.9167	0.00035	0.00869612
NM_032040.3	83987	CCDC8	3334	2.47606	3.14983	6.2283	-1.33079	0.0002	0.00554203	-0.983566	0.00165	0.0290957
NM_198474.3	283298	OLFML1	2905	7.09282	7.86364	13.9819	-0.97913	0.0002	0.00554203	-0.830293	0.00205	0.0341961
NM_173814.4	283659	PRTG	11973	2.97784	2.89744	5.65892	-0.926262	0.0002	0.00554203	-0.96575	5.00E-05	0.00170056
NM_001165.3	330	BIRC3	5229	77.5134	53.0886	27.1518	1.5134	0.00015	0.00437065	0.967354	0.00035	0.00869612
NM_001908.3	1508	CTSB	3783	135.595	118.423	61.0981	1.15011	0.00015	0.00437065	0.95475	0.0012	0.0230244
NM_002009.3	2252	FGF7	3936	71.2442	72.2474	32.1	1.1502	0.00015	0.00437065	1.17037	0.0004	0.00971957
NM_000849.4	2947	GSTM3	4127	8.9259	7.44923	17.5574	-0.97601	0.00015	0.00437065	-1.23691	5.00E-05	0.00170056
3493	3493	-	1061	1474.25	2466.19	410.424	1.8448	0.00015	0.00437065	2.5871	0.0001	0.00312189
NM_002334.3	4038	LRP4	8227	2.85108	1.47768	6.31228	-1.14665	0.00015	0.00437065	-2.09483	5.00E-05	0.00170056
NM_002704.3	5473	PPBP	1307	1.0146	0.9285	5.13818	-2.34035	0.00015	0.00437065	-2.46828	5.00E-05	0.00170056
NM_006948.4	6782	HSPA13	3987	35.16	44.7465	19.5935	0.843558	0.00015	0.00437065	1.1914	5.00E-05	0.00170056
NM_019856.1	8736	MYOM1	5559	0.915933	1.64154	3.51762	-1.94129	0.00015	0.00437065	-1.09955	0.003	0.0454886
NM_014791.2	9833	MELK	2439	3.48268	2.86879	0.952747	1.87003	0.00015	0.00437065	1.59028	0.00055	0.0124813
NM_015065.2	23086	EXPH5	10187	20.5618	22.3952	40.9518	-0.993963	0.00015	0.00437065	-0.870737	0.0008	0.0168217
NM_007364.2	23423	TMED3	1388	39.2003	37.7388	19.8637	0.98073	0.00015	0.00437065	0.925914	0.0004	0.00971957
NM_012413.3	25797	QPCT	1703	10.6364	9.90897	4.26102	1.31974	0.00015	0.00437065	1.21754	0.0005	0.0116249
NM_013340.2	29930	PCDHB1	2534	0.551842	0.775551	2.04343	-1.88866	0.00015	0.00437065	-1.3977	0.00235	0.0379132
NM_020801.2	57561	ARRDC3	4161	154.907	105.071	54.3784	1.5103	0.00015	0.00437065	0.950254	5.00E-05	0.00170056

NM_020844.2	57604	KIAA1456	9590	2.82762	3.90013	8.11618	-1.52121	0.00015	0.00437065	-1.34822	5.00E-05	0.00170056
NM_024893.1	79953	SYNDIG1	2437	1.91458	2.4692	0.447981	2.09552	0.00015	0.00437065	2.46254	5.00E-05	0.00170056
NM_032793.3	84879	MFSD2A	2162	6.96654	6.77281	2.42036	1.52522	0.00015	0.00437065	1.48453	0.0002	0.00554203
NM_080617.4	140689	CBLN4	2508	0.125269	0.302009	4.46969	-5.15707	0.00015	0.00437065	-3.88751	5.00E-05	0.00170056
NM_001136482.1	255809	C19orf38	1089	4.53113	3.26357	0.9874	2.19817	0.00015	0.00437065	1.72474	0.00205	0.0341961
NM_001134363.1	282996	RBM20	7233	1.92979	2.37751	4.45379	-1.20659	0.00015	0.00437065	-0.905582	0.0013	0.0244278
NM_178527.3	284525	SLC9A11	4427	0.874666	1.10057	3.02734	-1.79125	0.00015	0.00437065	-1.45979	5.00E-05	0.00170056
NM_001747.2	822	CAPG	1442	36.599	32.8522	17.5555	1.05988	0.0001	0.00312189	0.904069	0.0006	0.013408
NM_001142699.1	1740	DLG2	8046	0.732339	0.822409	2.50481	-1.77412	0.0001	0.00312189	-1.60678	0.0027	0.0421065
NM_006207.2	5157	PDGFRL	1905	5.0509	5.04637	12.6782	-1.32774	0.0001	0.00312189	-1.32904	5.00E-05	0.00170056
NM_021965.3	5239	PGM5	3338	5.3593	5.92989	11.5312	-1.10542	0.0001	0.00312189	-0.959459	0.00085	0.0176689
NM_173054.2	5649	RELN	11565	0.71523	0.083582	2.0522	-1.52069	0.0001	0.00312189	-4.61784	5.00E-05	0.00170056
NM_005080.3	7494	XBP1	1820	443.071	436.087	168.768	1.39249	0.0001	0.00312189	1.36957	0.00055	0.0124813
NM_006762.2	7805	LAPTM5	2240	98.3309	93.036	45.4328	1.11391	0.0001	0.00312189	1.03405	0.0002	0.00554203
NM_003637.3	8515	ITGA10	5170	1.76991	0.969669	4.08453	-1.20649	0.0001	0.00312189	-2.0746	5.00E-05	0.00170056
NM_032960.2	9261	MAPKAPK2	3052	27.0919	25.1577	13.431	1.0123	0.0001	0.00312189	0.905433	0.0009	0.0184743
NM_004267.3	9435	CHST2	3043	5.30288	4.15804	1.83738	1.52913	0.0001	0.00312189	1.17825	0.00115	0.0222874
NM_005763.3	10157	AASS	5871	21.2372	21.0205	40.6068	-0.935128	0.0001	0.00312189	-0.949921	5.00E-05	0.00170056
NM_021982.1	10802	SEC24A	6387	24.0523	22.5906	12.7041	0.920878	0.0001	0.00312189	0.830426	0.00035	0.00869612
NM_007035.3	11081	KERA	2533	0.236443	0.404639	1.56337	-2.7251	0.0001	0.00312189	-1.94996	0.0003	0.00772176
NM_012128.3	22802	CLCA4	3213	3.14861	9.70005	0.856218	1.87867	0.0001	0.00312189	3.50194	5.00E-05	0.00170056
NM_012450.2	26266	SLC13A4	2896	0.323442	0.459755	1.41974	-2.13405	0.0001	0.00312189	-1.62669	0.001	0.0199964
NM_019609.4	56265	CPXM1	2400	30.893	34.8521	62.6989	-1.02116	0.0001	0.00312189	-0.847192	0.0006	0.013408
NM_173050.3	80274	SCUBE1	3868	0.409544	0.462918	1.63163	-1.99422	0.0001	0.00312189	-1.81749	0.00015	0.00437065
NM_032855.2	84941	HSH2D	2367	9.8204	9.39671	4.01723	1.28958	0.0001	0.00312189	1.22595	0.00015	0.00437065
NM_080596.1	85235	HIST1H2AH	439	40.0337	34.9728	13.0957	1.61213	0.0001	0.00312189	1.41714	0.00075	0.0159498
NM_001130058.1	204962	SLC44A5	3894	0.0925231	0.334828	1.35922	-3.87682	0.0001	0.00312189	-2.02128	0.0007	0.015106
NM_207365.3	344752	AADACL2	1500	2.46449	2.54804	7.16366	-1.53941	0.0001	0.00312189	-1.49131	0.0001	0.00312189
NM_001085.4	12	SERPINA3	1590	469.152	89.048	14.9471	4.97211	5.00E-05	0.00170056	2.57471	5.00E-05	0.00170056
NM_020546.2	108	ADCY2	6553	2.7012	4.16552	9.7531	-1.85226	5.00E-05	0.00170056	-1.22736	5.00E-05	0.00170056
NM_001124.1	133	ADM	1449	21.7287	10.1365	2.81998	2.94584	5.00E-05	0.00170056	1.8458	5.00E-05	0.00170056
NM_000681.3	150	ADRA2A	3876	23.6584	14.3173	8.47532	1.48102	5.00E-05	0.00170056	0.756424	0.00325	0.0483534
NM_000694.2	221	ALDH3B1	2815	12.1305	13.9328	37.5336	-1.62955	5.00E-05	0.00170056	-1.4297	5.00E-05	0.00170056
NM_000698.2	240	ALOX5	2554	21.4268	13.8077	6.86329	1.64244	5.00E-05	0.00170056	1.0085	0.0007	0.015106
NM_001629.3	241	ALOX5AP	906	48.3052	27.1364	13.3778	1.85234	5.00E-05	0.00170056	1.0204	0.00135	0.0250595
NM_001152.4	292	SLC25A5	1351	99.3271	79.9183	45.4862	1.12676	5.00E-05	0.00170056	0.813096	0.0007	0.015106
NM_000044.2	367	AR	4314	0.790178	1.22889	3.04562	-1.94649	5.00E-05	0.00170056	-1.30938	0.002	0.0335868
NM_004319.1	460	ASTN1	7314	1.72593	1.88765	4.67112	-1.43639	5.00E-05	0.00170056	-1.30718	0.00015	0.00437065
NM_000702.3	477	ATP1A2	5464	6.65188	8.24277	38.7615	-2.54279	5.00E-05	0.00170056	-2.23342	5.00E-05	0.00170056
NM_001692.3	525	ATP6V1B1	1939	1.13725	1.94418	6.22053	-2.45149	5.00E-05	0.00170056	-1.67788	0.0002	0.00554203
NM_004049.3	597	BCL2A1	887	63.1543	14.7406	4.45226	3.82627	5.00E-05	0.00170056	1.72719	0.00055	0.0124813
NM_001198.3	639	PRDM1	5165	32.3901	24.887	7.43687	2.12278	5.00E-05	0.00170056	1.74262	5.00E-05	0.00170056
NM_021073.2	653	BMP5	2172	27.5571	28.3511	9.13111	1.59356	5.00E-05	0.00170056	1.63454	5.00E-05	0.00170056
NM_000061.2	695	BTK	2611	22.4299	12.1575	6.12768	1.87201	5.00E-05	0.00170056	0.988437	0.00075	0.0159498
NM_000065.2	729	C6	3606	3.27154	5.57945	21.8203	-2.73763	5.00E-05	0.00170056	-1.96747	5.00E-05	0.00170056
NM_001218.3	771	CA12	3975	21.661	27.1506	3.83575	2.49752	5.00E-05	0.00170056	2.8234	5.00E-05	0.00170056
NM_000616.4	920	CD4	3116	19.5486	14.5046	7.53192	1.37598	5.00E-05	0.00170056	0.945418	0.0019	0.0323187
NM_001781.2	969	CD69	1676	260.814	25.6891	14.1771	4.20138	5.00E-05	0.00170056	0.857589	0.0025	0.0397144
NM_001783.3	973	CD79A	1258	33.8568	62.0491	10.9739	1.62537	5.00E-05	0.00170056	2.49933	5.00E-05	0.00170056
NM_001254.3	990	CDC6	3053	2.98069	2.2121	0.813722	1.87304	5.00E-05	0.00170056	1.44281	0.0008	0.0168217
NM_001792.3	1000	CDH2	4367	1.61473	2.49161	5.61369	-1.79766	5.00E-05	0.00170056	-1.17187	0.0005	0.0116249
NM_004065.2	1038	CDR1	1299	8.47655	3.38912	40.7513	-2.2653	5.00E-05	0.00170056	-3.58786	5.00E-05	0.00170056
NM_004363.2	1048	CEACAM5	3600	41.1753	12.0636	0.671976	5.93723	5.00E-05	0.00170056	4.16611	5.00E-05	0.00170056
NM_005507.2	1072	CFL1	1260	183.278	147.256	85.6049	1.09827	5.00E-05	0.00170056	0.782559	0.00105	0.0207193

NM_001814.4	1075	CTSC	1907	359.574	147.917	63.5718	2.49983	5.00E-05	0.00170056	1.21833	5.00E-05	0.00170056
NM_001854.3	1301	COL11A1	7291	6.26187	12.5191	32.4633	-2.37415	5.00E-05	0.00170056	-1.37468	0.0003	0.00772176
NM_000494.3	1308	COL17A1	5610	6.47481	6.93792	1.50136	2.10857	5.00E-05	0.00170056	2.20823	5.00E-05	0.00170056
NM_001870.2	1359	CPA3	1686	264.984	74.1552	36.5306	2.85873	5.00E-05	0.00170056	1.02144	5.00E-05	0.00170056
NM_000573.3	1378	CR1	8616	13.4739	6.72801	1.75544	2.94027	5.00E-05	0.00170056	1.93835	5.00E-05	0.00170056
NM_001884.3	1404	HAPLN1	4678	1.84705	0.565044	6.04425	-1.71034	5.00E-05	0.00170056	-3.41913	5.00E-05	0.00170056
NM_000395.2	1439	CSF2RB	4848	21.9423	12.4538	3.70911	2.56457	5.00E-05	0.00170056	1.74744	5.00E-05	0.00170056
NM_001321.1	1466	CSRP2	901	68.4496	107.577	290.382	-2.08484	5.00E-05	0.00170056	-1.43258	5.00E-05	0.00170056
NM_005213.3	1475	CSTA	828	43.2697	39.4759	10.0048	2.11267	5.00E-05	0.00170056	1.98029	5.00E-05	0.00170056
NM_001911.2	1511	CTSG	924	46.5036	19.4127	6.01945	2.94964	5.00E-05	0.00170056	1.6893	5.00E-05	0.00170056
NM_000396.3	1513	CTSK	1810	46.1817	58.0294	127.079	-1.46033	5.00E-05	0.00170056	-1.13087	0.0005	0.0116249
NM_000101.2	1535	CYBA	688	162.801	235.814	76.725	1.08534	5.00E-05	0.00170056	1.61988	5.00E-05	0.00170056
NM_000397.3	1536	CYBB	4318	72.5272	96.4217	32.2032	1.17132	5.00E-05	0.00170056	1.58215	5.00E-05	0.00170056
NM_000766.3	1553	CYP2A13	1747	5.61842	4.96824	19.9971	-1.83156	5.00E-05	0.00170056	-2.00899	5.00E-05	0.00170056
NM_001099772.1	1580	CYP4B1	2158	41.2942	45.0329	128.138	-1.63369	5.00E-05	0.00170056	-1.50865	0.0002	0.00554203
NM_000574.3	1604	CD55	2796	68.7664	94.4863	21.4055	1.68372	5.00E-05	0.00170056	2.14212	5.00E-05	0.00170056
NM_001369.2	1767	DNAH5	15573	14.6696	34.1222	86.2259	-2.55529	5.00E-05	0.00170056	-1.33741	0.00015	0.00437065
NM_001372.3	1770	DNAH9	13750	6.92764	11.576	24.7797	-1.83872	5.00E-05	0.00170056	-1.09802	5.00E-05	0.00170056
NM_004944.2	1776	DNASE1L3	1463	5.40095	10.271	19.5908	-1.85889	5.00E-05	0.00170056	-0.931603	0.00145	0.0264203
NM_024422.3	1824	DSC2	5199	12.3131	6.96374	2.49327	2.30409	5.00E-05	0.00170056	1.48182	5.00E-05	0.00170056
NM_001944.2	1830	DSG3	5551	4.54055	3.93888	1.05254	2.10899	5.00E-05	0.00170056	1.9039	5.00E-05	0.00170056
NM_000112.3	1836	SLC26A2	8075	56.1722	71.5924	22.1299	1.34386	5.00E-05	0.00170056	1.69381	5.00E-05	0.00170056
NM_004417.3	1843	DUSP1	2024	356.027	53.0246	27.4142	3.69899	5.00E-05	0.00170056	0.951737	0.0002	0.00554203
NM_004419.3	1847	DUSP5	2528	51.4685	9.9697	3.02362	4.08934	5.00E-05	0.00170056	1.72127	5.00E-05	0.00170056
NM_001974.3	2015	EMR1	3123	19.6724	1.82071	0.317868	5.95161	5.00E-05	0.00170056	2.518	5.00E-05	0.00170056
NM_001428.3	2023	ENO1	2187	172.998	134.316	74.8777	1.20815	5.00E-05	0.00170056	0.843021	0.00105	0.0207193
NM_005233.5	2042	EPHA3	5809	0.591364	1.05007	6.61111	-3.48277	5.00E-05	0.00170056	-2.65441	5.00E-05	0.00170056
NM_000130.4	2153	F5	9179	5.81776	4.60575	2.19784	1.40438	5.00E-05	0.00170056	1.06735	0.00035	0.00869612
NM_000129.3	2162	F13A1	3863	283.732	145.465	50.3977	2.4931	5.00E-05	0.00170056	1.52924	5.00E-05	0.00170056
NM_001442.2	2167	FABP4	838	4.04084	3.9004	34.3176	-3.08622	5.00E-05	0.00170056	-3.13725	5.00E-05	0.00170056
NM_004106.1	2207	FCER1G	591	56.3504	51.6052	20.4269	1.46396	5.00E-05	0.00170056	1.33705	5.00E-05	0.00170056
NM_002003.3	2219	FCN1	1283	17.2839	30.9812	3.82727	2.17504	5.00E-05	0.00170056	3.01701	5.00E-05	0.00170056
NM_002010.2	2254	FGF9	4530	0.735149	1.26164	3.95586	-2.42788	5.00E-05	0.00170056	-1.64869	5.00E-05	0.00170056
NM_005252.3	2353	FOS	2158	486.04	44.4	7.21553	6.07382	5.00E-05	0.00170056	2.62138	5.00E-05	0.00170056
NM_002029.3	2357	FPR1	1317	15.3973	17.3148	2.93228	2.39258	5.00E-05	0.00170056	2.56191	5.00E-05	0.00170056
NM_002046.3	2597	GAPDH	1310	738.082	579.618	290.794	1.34379	5.00E-05	0.00170056	0.995108	0.0007	0.015106
NM_145793.3	2674	GFRA1	9134	8.34266	7.77643	27.8384	-1.7385	5.00E-05	0.00170056	-1.8399	5.00E-05	0.00170056
NM_004004.5	2706	GJB2	2334	14.2501	14.811	5.78519	1.30054	5.00E-05	0.00170056	1.35624	5.00E-05	0.00170056
NM_006142.3	2810	SFN	1315	29.6634	13.6089	6.7159	2.14303	5.00E-05	0.00170056	1.01889	0.00215	0.0355296
NM_005290.1	2838	GPR15	1083	5.75576	2.71639	0.753787	2.93278	5.00E-05	0.00170056	1.84946	0.0027	0.0421065
NM_005306.2	2867	FFAR2	2053	9.19102	1.82213	0.522918	4.13557	5.00E-05	0.00170056	1.80097	0.00125	0.02372
NM_002089.3	2920	CXCL2	1205	67.4621	6.53973	1.4232	5.56687	5.00E-05	0.00170056	2.20009	5.00E-05	0.00170056
NM_000851.3	2949	GSTM5	1570	0.357057	2.35722	7.12814	-4.3193	5.00E-05	0.00170056	-1.59644	0.00015	0.00437065
NM_005322.2	3009	HIST1H1B	790	61.894	55.6771	20.4327	1.59892	5.00E-05	0.00170056	1.4462	5.00E-05	0.00170056
NM_005328.2	3037	HAS2	3275	6.21144	4.18533	1.47874	2.07056	5.00E-05	0.00170056	1.50097	0.00015	0.00437065
NM_001172129.1	3055	HCK	2155	14.9061	9.49841	4.50093	1.72761	5.00E-05	0.00170056	1.07746	0.0022	0.0361165
NM_005337.4	3071	NCKAP1L	3893	32.9666	29.9674	15.6878	1.07136	5.00E-05	0.00170056	0.933746	0.0001	0.00312189
NM_001530.3	3091	HIF1A	4059	109.259	69.53	37.4411	1.54506	5.00E-05	0.00170056	0.893015	0.00025	0.00665625
NM_000189.4	3099	HK2	7093	13.488	7.87652	2.62488	2.36135	5.00E-05	0.00170056	1.58531	5.00E-05	0.00170056
NM_002133.2	3162	HMOX1	1590	19.0023	11.0675	4.10273	2.21152	5.00E-05	0.00170056	1.43167	5.00E-05	0.00170056
NM_002162.3	3385	ICAM3	1780	19.6828	18.9177	7.05418	1.48038	5.00E-05	0.00170056	1.42319	5.00E-05	0.00170056
NM_000598.4	3486	IGFBP3	2613	203.466	365.724	71.5229	1.50831	5.00E-05	0.00170056	2.35428	5.00E-05	0.00170056
NM_001554.4	3491	CYR61	2295	110.679	33.3372	13.7271	3.01128	5.00E-05	0.00170056	1.2801	5.00E-05	0.00170056
3494	3494	-	1019	726.672	766.09	93.4102	2.95965	5.00E-05	0.00170056	3.03586	5.00E-05	0.00170056

3501	3501	-	980	488.109	753.4	85.1202	2.51963	5.00E-05	0.00170056	3.14584	5.00E-05	0.00170056
3503	3503	-	983	3703.86	173.058	21.8126	7.40772	5.00E-05	0.00170056	2.98802	5.00E-05	0.00170056
3505	3505	-	1188	11.741	2.29208	0.520062	4.49673	5.00E-05	0.00170056	2.1399	0.00075	0.0159498
3538	3538	-	320	2118.9	1653.31	391.077	2.43779	5.00E-05	0.00170056	2.07984	5.00E-05	0.00170056
3539	3539	-	318	646.944	996.829	166.569	1.95752	5.00E-05	0.00170056	2.58122	5.00E-05	0.00170056
NM_000576.2	3553	IL1B	1498	3.1396	3.9513	0.462931	2.76171	5.00E-05	0.00170056	3.09346	5.00E-05	0.00170056
NM_000417.2	3559	IL2RA	3216	5.75801	3.33699	1.52618	1.91564	5.00E-05	0.00170056	1.12862	0.00275	0.0426687
NM_000584.3	3576	IL8	1705	17.5023	34.9818	4.72844	1.88811	5.00E-05	0.00170056	2.88717	5.00E-05	0.00170056
NM_001558.3	3587	IL10RA	3656	24.6936	23.2529	8.54456	1.53106	5.00E-05	0.00170056	1.44433	5.00E-05	0.00170056
NM_002460.3	3662	IRF4	5327	10.6825	16.4181	3.36478	1.66667	5.00E-05	0.00170056	2.2867	5.00E-05	0.00170056
NM_000211.3	3689	ITGB2	2958	37.2093	27.7845	12.9395	1.52388	5.00E-05	0.00170056	1.10249	0.0001	0.00312189
NM_002229.2	3726	JUNB	1816	148.001	27.4683	12.9681	3.51257	5.00E-05	0.00170056	1.0828	5.00E-05	0.00170056
NM_000217.2	3736	KCNA1	7983	0.253352	0.525153	2.53179	-3.32095	5.00E-05	0.00170056	-2.26935	5.00E-05	0.00170056
NM_004522.1	3800	KIF5C	6933	4.06583	2.5609	0.995353	2.03027	5.00E-05	0.00170056	1.36337	0.0001	0.00312189
NM_002266.2	3838	KPNA2	1980	20.5454	15.3342	7.89084	1.38057	5.00E-05	0.00170056	0.958505	0.00085	0.0176689
NM_005554.3	3853	KRT6A	2450	21.7183	12.5934	1.69885	3.67628	5.00E-05	0.00170056	2.89004	5.00E-05	0.00170056
NM_153490.2	3860	KRT13	1715	3.097	6.46307	0.675297	2.19728	5.00E-05	0.00170056	3.25863	5.00E-05	0.00170056
NM_000526.4	3861	KRT14	1636	5.29778	9.3015	0.611884	3.11406	5.00E-05	0.00170056	3.92613	5.00E-05	0.00170056
NM_002275.3	3866	KRT15	1840	114.291	84.4943	40.1227	1.51021	5.00E-05	0.00170056	1.07443	5.00E-05	0.00170056
NM_005566.3	3939	LDHA	2208	234.909	195.49	77.9652	1.5912	5.00E-05	0.00170056	1.32619	5.00E-05	0.00170056
NM_001198557.1	4001	LMNB1	2250	17.5965	13.3513	6.30134	1.48156	5.00E-05	0.00170056	1.08324	0.00225	0.0367046
NM_005582.2	4064	CD180	2716	11.7325	9.4064	3.95623	1.56831	5.00E-05	0.00170056	1.24951	0.00015	0.00437065
NM_000239.2	4069	LYZ	1516	905.233	1996.13	199.578	2.18134	5.00E-05	0.00170056	3.32218	0.0024	0.03848
NM_000528.3	4125	MAN2B1	3224	31.3591	26.2749	14.3094	1.13192	5.00E-05	0.00170056	0.876721	0.0009	0.0184743
NM_012328.2	4189	DNAJB9	2538	39.975	59.0113	18.8914	1.08136	5.00E-05	0.00170056	1.64326	5.00E-05	0.00170056
NM_004994.2	4318	MMP9	2336	0.916865	4.45143	14.5385	-3.98702	5.00E-05	0.00170056	-1.70753	5.00E-05	0.00170056
NM_002427.3	4322	MMP13	2719	0.874896	0.879573	159.113	-7.50672	5.00E-05	0.00170056	-7.49903	5.00E-05	0.00170056
NM_002432.1	4332	MNDA	1667	31.3812	39.4322	13.1432	1.25559	5.00E-05	0.00170056	1.58506	5.00E-05	0.00170056
NM_002457.2	4583	MUC2	8623	1.39396	1.72799	0.208277	2.74262	5.00E-05	0.00170056	3.05252	5.00E-05	0.00170056
NM_000615.6	4684	NCAM1	5962	1.0277	2.3043	5.78442	-2.49275	5.00E-05	0.00170056	-1.32784	0.0004	0.00971957
NM_000631.4	4689	NCF4	1398	13.9908	9.52919	3.12951	2.16047	5.00E-05	0.00170056	1.60641	0.0015	0.0270821
NM_002514.3	4856	NOV	2601	5.74545	6.88939	34.4704	-2.58487	5.00E-05	0.00170056	-2.32291	5.00E-05	0.00170056
NM_001007156.1	4916	NTRK3	3982	1.92985	1.84269	6.62195	-1.77877	5.00E-05	0.00170056	-2.20263	0.00025	0.00665625
NM_000275.2	4948	OCA2	3140	0.380531	0.665624	1.91235	-2.32926	5.00E-05	0.00170056	-1.52257	0.00125	0.02372
NM_000602.3	5054	SERPINE1	3181	22.7471	4.26985	0.726664	4.96825	5.00E-05	0.00170056	2.55482	5.00E-05	0.00170056
NM_002578.3	5063	PAK3	2754	1.69967	1.90389	6.02646	-1.82606	5.00E-05	0.00170056	-1.66236	5.00E-05	0.00170056
NM_000922.3	5140	PDE3B	6091	8.94566	9.26319	2.87868	1.63578	5.00E-05	0.00170056	1.6861	5.00E-05	0.00170056
NM_001037339.1	5142	PDE4B	3876	20.0895	13.6349	4.28492	2.2291	5.00E-05	0.00170056	1.66997	5.00E-05	0.00170056
NM_000442.4	5175	PECAM1	4449	36.6826	25.6531	13.0084	1.49566	5.00E-05	0.00170056	0.979693	5.00E-05	0.00170056
NM_001146187.1	5178	PEG3	8375	2.35229	2.27494	5.29089	-1.16944	5.00E-05	0.00170056	-1.21769	0.00045	0.0106989
NM_000291.3	5230	PGK1	2423	107.952	97.5128	57.1127	0.918506	5.00E-05	0.00170056	0.77178	0.0013	0.0244278
NM_002648.3	5292	PIM1	2709	31.7851	11.6142	6.26396	2.34321	5.00E-05	0.00170056	0.890749	0.00205	0.0341961
NM_002649.2	5294	PIK3CG	5379	18.0233	12.7896	7.36006	1.29208	5.00E-05	0.00170056	0.797184	0.00195	0.0329693
NM_000930.3	5327	PLAT	3173	5.84999	13.9735	28.5812	-2.28856	5.00E-05	0.00170056	-1.03237	0.0001	0.00312189
NM_002658.3	5328	PLAU	2378	9.93527	5.91869	2.54705	1.96373	5.00E-05	0.00170056	1.21645	0.00165	0.0290957
NM_002661.2	5336	PLCG2	4241	21.303	16.7802	8.93604	1.25335	5.00E-05	0.00170056	0.909057	0.00025	0.00665625
NM_002664.2	5341	PLEK	2852	35.5301	31.5762	12.9319	1.45811	5.00E-05	0.00170056	1.2879	5.00E-05	0.00170056
NM_199478.1	5354	PLP1	2921	2.1592	4.03874	8.45052	-1.96854	5.00E-05	0.00170056	-1.06513	0.00165	0.0290957
NM_006235.2	5450	POU2AF1	3032	9.76899	9.3752	3.01664	1.69526	5.00E-05	0.00170056	1.6359	5.00E-05	0.00170056
NM_002725.3	5549	PRELP	5820	0.70576	1.29927	4.20122	-2.57356	5.00E-05	0.00170056	-1.69311	5.00E-05	0.00170056
NM_002727.2	5552	SRGN	1254	600.399	222.297	112.672	2.41379	5.00E-05	0.00170056	0.980361	0.0001	0.00312189
NM_000949.5	5618	PRLR	11817	4.6013	3.93311	15.3758	-1.74054	5.00E-05	0.00170056	-1.96692	5.00E-05	0.00170056
NM_000313.3	5627	PROS1	3580	22.5316	20.8197	60.5745	-1.42676	5.00E-05	0.00170056	-1.54076	5.00E-05	0.00170056
NM_000963.2	5743	PTGS2	4493	41.232	15.1943	2.13877	4.26891	5.00E-05	0.00170056	2.82868	5.00E-05	0.00170056

NM_198964.1	5744	PTHLH	1854	12.9291	11.2623	0.810568	3.99554	5.00E-05	0.00170056	3.79642	5.00E-05	0.00170056
NM_001184744.1	5745	PTH1R	1991	1.98806	1.56498	6.44491	-1.6968	5.00E-05	0.00170056	-2.04202	5.00E-05	0.00170056
NM_002825.5	5764	PTN	1549	15.0362	33.1337	60.3928	-2.00594	5.00E-05	0.00170056	-0.866077	0.00055	0.0124813
NM_002864.2	5858	PZP	4610	2.85142	5.29491	10.6603	-1.90249	5.00E-05	0.00170056	-1.00957	0.0003	0.00772176
NM_006743.4	5935	RBM3	4432	83.635	68.2714	32.7683	1.35181	5.00E-05	0.00170056	1.05898	5.00E-05	0.00170056
NM_002922.3	5996	RGS1	1403	201.782	67.9109	19.2747	3.38802	5.00E-05	0.00170056	1.81694	5.00E-05	0.00170056
NM_002923.3	5997	RGS2	1350	282.433	49.8778	16.1875	4.12495	5.00E-05	0.00170056	1.62351	5.00E-05	0.00170056
NM_002934.2	6036	RNASE2	735	13.7152	7.39762	1.89386	2.85638	5.00E-05	0.00170056	1.96573	0.00155	0.0278206
NM_006269.1	6101	RP1	7100	6.13558	8.64943	18.6022	-1.6002	5.00E-05	0.00170056	-1.1048	5.00E-05	0.00170056
NM_001036.3	6263	RYR3	15559	0.794222	0.556124	2.2559	-1.50609	5.00E-05	0.00170056	-2.02023	5.00E-05	0.00170056
NM_005978.3	6273	S100A2	963	129.743	90.9409	23.2854	2.47816	5.00E-05	0.00170056	1.9655	5.00E-05	0.00170056
NM_005980.2	6286	S100P	510	43.0612	53.9113	5.77578	2.8983	5.00E-05	0.00170056	3.2225	5.00E-05	0.00170056
NM_002974.2	6318	SERPINB4	1719	103.029	94.5907	3.23896	4.99138	5.00E-05	0.00170056	4.86809	5.00E-05	0.00170056
NM_004588.4	6327	SCN2B	4922	0.499338	0.610597	1.60203	-1.68181	5.00E-05	0.00170056	-1.39161	0.00105	0.0207193
NM_002976.3	6332	SCN7A	7186	0.456659	1.61744	3.11981	-2.77227	5.00E-05	0.00170056	-0.947749	0.002	0.0335868
NM_002982.3	6347	CCL2	749	116.617	61.4655	27.8862	2.06415	5.00E-05	0.00170056	1.14022	0.00015	0.00437065
NM_005623.2	6355	CCL8	1250	8.91826	6.99974	1.16458	2.93696	5.00E-05	0.00170056	2.5875	5.00E-05	0.00170056
NM_002986.2	6356	CCL11	925	26.4263	10.2261	2.42561	3.44556	5.00E-05	0.00170056	2.07584	5.00E-05	0.00170056
NM_002988.2	6362	CCL18	770	279.754	3.92204	0.653166	8.74249	5.00E-05	0.00170056	2.58608	0.0011	0.0214605
NM_002997.4	6382	SDC1	3201	64.9469	56.1283	26.6973	1.28257	5.00E-05	0.00170056	1.07203	5.00E-05	0.00170056
NM_000450.2	6401	SELE	3875	15.0178	3.73904	1.0889	3.78573	5.00E-05	0.00170056	1.7798	5.00E-05	0.00170056
NM_000655.4	6402	SELL	2442	23.0295	22.236	7.72278	1.57629	5.00E-05	0.00170056	1.5257	5.00E-05	0.00170056
NM_003014.3	6424	SFRP4	2974	7.14851	25.1784	153.406	-4.42357	5.00E-05	0.00170056	-2.60709	5.00E-05	0.00170056
NM_003037.2	6504	SLAMF1	2001	8.36112	6.94837	3.22793	1.37309	5.00E-05	0.00170056	1.10607	0.0013	0.0244278
NM_006931.2	6515	SLC2A3	3938	64.4682	11.5063	6.6203	3.28362	5.00E-05	0.00170056	0.797461	0.00275	0.0426687
NM_006933.4	6526	SLC5A3	11576	95.1134	61.8618	28.0502	1.76164	5.00E-05	0.00170056	1.14104	0.0001	0.00312189
NM_001008539.3	6542	SLC7A2	7571	20.0817	27.3407	81.1648	-2.01497	5.00E-05	0.00170056	-1.5698	5.00E-05	0.00170056
NM_001046.2	6558	SLC12A2	6860	58.2409	55.2115	26.3532	1.14405	5.00E-05	0.00170056	1.06699	0.0002	0.00554203
NM_001040058.1	6696	SPP1	1625	23.5519	13.1633	102.889	-2.12717	5.00E-05	0.00170056	-2.9665	5.00E-05	0.00170056
NM_006280.2	6748	SSR4	725	199.183	253.8	78.1322	1.35011	5.00E-05	0.00170056	1.6997	5.00E-05	0.00170056
NM_003155.2	6781	STC1	3877	19.4096	10.8552	6.10944	1.66766	5.00E-05	0.00170056	0.829278	0.00205	0.0341961
NM_001062.3	6947	TCN1	1567	227.086	59.922	24.9139	3.18821	5.00E-05	0.00170056	1.26613	0.0008	0.0168217
NM_005651.2	6999	TDO2	1693	4.21725	3.39385	0.56747	2.89369	5.00E-05	0.00170056	2.58031	5.00E-05	0.00170056
NM_003226.3	7033	TFF3	1054	104.977	64.5205	17.8191	2.55858	5.00E-05	0.00170056	1.85634	5.00E-05	0.00170056
NM_001128148.1	7037	TFRC	5083	39.0907	43.4388	15.7885	1.30795	5.00E-05	0.00170056	1.46011	5.00E-05	0.00170056
NM_003248.4	7060	THBS4	3223	1.12346	0.367177	10.474	-3.22079	5.00E-05	0.00170056	-4.83419	5.00E-05	0.00170056
NM_175610.2	7082	TJP1	6925	17.6747	13.7364	54.4488	-1.62321	5.00E-05	0.00170056	-1.98689	5.00E-05	0.00170056
NM_006290.2	7128	TNFAIP3	4432	54.1129	26.7074	15.3184	1.82071	5.00E-05	0.00170056	0.801976	0.00135	0.0250595
NM_007115.3	7130	TNFAIP6	1424	10.3747	8.50815	1.43809	2.85083	5.00E-05	0.00170056	2.56469	5.00E-05	0.00170056
NM_001067.3	7153	TOP2A	5753	14.6506	10.5628	4.72787	1.63169	5.00E-05	0.00170056	1.15972	0.00015	0.00437065
NM_003357.4	7356	SCGB1A1	452	48.8071	20.9764	2998.95	-5.94122	5.00E-05	0.00170056	-7.15955	5.00E-05	0.00170056
NM_007153.3	7757	ZNF208	9088	2.41327	1.8439	6.95137	-1.52631	5.00E-05	0.00170056	-1.91454	5.00E-05	0.00170056
NM_013250.2	7762	ZNF215	3658	8.10533	10.744	2.64557	1.61529	5.00E-05	0.00170056	2.02188	5.00E-05	0.00170056
NM_014518.2	7772	ZNF229	4964	0.80532	1.40975	2.9793	-1.88734	5.00E-05	0.00170056	-1.07953	0.0012	0.0230244
NM_003463.3	7803	PTP4A1	5082	42.6519	35.7465	18.4763	1.20693	5.00E-05	0.00170056	0.952128	5.00E-05	0.00170056
NM_006763.2	7832	BTG2	2712	210.617	76.3341	46.769	2.171	5.00E-05	0.00170056	0.706773	0.00335	0.0494369
NM_003469.4	7857	SCG2	2572	88.0674	83.0054	20.8374	2.07944	5.00E-05	0.00170056	1.99403	5.00E-05	0.00170056
NM_003537.3	8358	HIST1H3B	472	50.0093	38.4051	15.5455	1.6857	5.00E-05	0.00170056	1.3048	0.0023	0.0373663
NM_003608.3	8477	GPR65	4522	18.477	9.4031	4.10697	2.16958	5.00E-05	0.00170056	1.19506	5.00E-05	0.00170056
NM_003613.3	8483	CILP	4468	0.386228	0.455992	1.67811	-2.11931	5.00E-05	0.00170056	-1.87976	0.0001	0.00312189
NM_003619.3	8492	PRSS12	4573	11.6658	14.7944	27.5263	-1.23853	5.00E-05	0.00170056	-0.895756	0.00015	0.00437065
NM_003890.2	8857	FCGBP	16390	5.4615	2.30969	0.968251	2.49584	5.00E-05	0.00170056	1.25425	5.00E-05	0.00170056
NM_003897.3	8870	IER3	1240	35.0216	27.5977	10.9513	1.67714	5.00E-05	0.00170056	1.33345	5.00E-05	0.00170056
NM_003955.3	9021	SOCS3	2734	117.977	15.2486	3.55188	5.05378	5.00E-05	0.00170056	2.10202	5.00E-05	0.00170056

NM_001080508.1	9096	TBX18	4070	2.05212	2.66174	5.73194	-1.48191	5.00E-05	0.00170056	-1.10665	0.0006	0.013408
NM_004683.4	9104	RGN	1615	1.45765	1.84038	9.81361	-2.75114	5.00E-05	0.00170056	-2.41478	5.00E-05	0.00170056
NM_021103.3	9168	TMSB10	482	1244.13	1339.84	601.074	1.04952	5.00E-05	0.00170056	1.15645	5.00E-05	0.00170056
NM_005097.2	9211	LGI1	2366	2.39658	4.21462	0.473709	2.3389	5.00E-05	0.00170056	3.15333	5.00E-05	0.00170056
NM_004744.3	9227	LRAT	4888	0.144844	0.406415	7.35898	-5.66694	5.00E-05	0.00170056	-4.17848	5.00E-05	0.00170056
NM_004751.2	9245	GCNT3	2222	8.69937	5.7908	1.53688	2.50091	5.00E-05	0.00170056	1.91376	5.00E-05	0.00170056
NM_004787.1	9353	SLIT2	4950	9.02891	6.46368	55.0618	-2.60843	5.00E-05	0.00170056	-3.09062	5.00E-05	0.00170056
NM_004274.4	9472	AKAP6	10387	5.29712	7.24343	11.8848	-1.16584	5.00E-05	0.00170056	-0.714375	0.0025	0.0397144
NM_001105556.1	9473	C1orf38	2723	16.0843	13.0308	4.60949	1.80297	5.00E-05	0.00170056	1.49925	5.00E-05	0.00170056
NM_006988.3	9510	ADAMTS1	4651	107.144	46.9244	11.7692	3.18646	5.00E-05	0.00170056	1.99532	5.00E-05	0.00170056
NM_004877.2	9535	GMFG	657	54.6316	50.1102	20.698	1.40025	5.00E-05	0.00170056	1.27562	0.0001	0.00312189
NM_004288.4	9595	CYTIP	2210	59.8032	48.1469	26.2065	1.1903	5.00E-05	0.00170056	0.877521	0.00075	0.0159498
NM_006536.5	9635	CLCA2	4025	9.59893	6.52949	2.5704	1.90088	5.00E-05	0.00170056	1.34498	0.00035	0.00869612
NM_014674.2	9695	EDEM1	6153	29.5093	23.8921	12.1409	1.2813	5.00E-05	0.00170056	0.976661	0.0001	0.00312189
NM_014675.3	9696	CROCC	6656	4.96803	5.33402	11.6895	-1.23446	5.00E-05	0.00170056	-1.13191	5.00E-05	0.00170056
NM_014685.2	9709	HERPUD1	2176	111.498	106.739	49.9015	1.15986	5.00E-05	0.00170056	1.09693	5.00E-05	0.00170056
NM_014737.2	9770	RASSF2	5426	44.2518	25.4755	12.0377	1.87818	5.00E-05	0.00170056	1.08155	5.00E-05	0.00170056
NM_012301.3	9863	MAGI2	6880	2.93304	3.06891	6.31978	-1.10748	5.00E-05	0.00170056	-1.04215	5.00E-05	0.00170056
NM_005114.2	9957	HS3ST1	1965	25.9978	23.8248	6.63289	1.97068	5.00E-05	0.00170056	1.84476	5.00E-05	0.00170056
NM_005130.3	9982	FGFBP1	1182	7.63529	4.68987	0.744421	3.35849	5.00E-05	0.00170056	2.65536	5.00E-05	0.00170056
NM_005467.3	10003	NAALAD2	3196	3.64783	4.35563	9.39162	-1.36434	5.00E-05	0.00170056	-1.10849	0.00025	0.00665625
NM_005746.2	10135	NAMPT	4582	109.804	78.8086	33.1088	1.72964	5.00E-05	0.00170056	1.25114	5.00E-05	0.00170056
NM_025195.2	10221	TRIB1	3633	39.9782	19.9818	7.00342	2.51308	5.00E-05	0.00170056	1.51256	5.00E-05	0.00170056
NM_080283.3	10350	ABCA9	6301	3.89484	4.62366	10.4484	-1.42365	5.00E-05	0.00170056	-1.17618	5.00E-05	0.00170056
NM_006332.3	10437	IFI30	1032	108.255	104.067	25.0932	2.10906	5.00E-05	0.00170056	2.05214	5.00E-05	0.00170056
NM_031456.3	10517	FBXW10	3439	0.687069	1.51755	3.57936	-2.38117	5.00E-05	0.00170056	-1.23796	0.00085	0.0176689
NM_006406.1	10549	PRDX4	921	87.0289	86.3149	34.3174	1.34255	5.00E-05	0.00170056	1.33067	5.00E-05	0.00170056
NM_006469.4	10625	IVNS1ABP	4199	125.692	87.5578	49.9464	1.33144	5.00E-05	0.00170056	0.809855	0.00125	0.02372
NM_006636.3	10797	MTHFD2	2188	21.7496	22.7176	8.87822	1.29265	5.00E-05	0.00170056	1.35547	5.00E-05	0.00170056
NM_006639.2	10800	CYSLTR1	1514	20.5954	15.7628	7.66382	1.42618	5.00E-05	0.00170056	1.04039	0.001	0.0199964
NM_032385.3	10826	C5orf4	2997	9.1989	11.8357	20.1378	-1.13037	5.00E-05	0.00170056	-0.766755	0.00315	0.0471261
NM_006875.3	11040	PIM2	2187	61.1768	52.7422	7.95323	2.94337	5.00E-05	0.00170056	2.72935	5.00E-05	0.00170056
NM_007038.3	11096	ADAMTS5	9663	1.9889	3.5609	6.34585	-1.67384	5.00E-05	0.00170056	-0.833573	0.001	0.0199964
NM_007072.2	11148	HHLA2	2660	2.21665	1.35291	7.80557	-1.81612	5.00E-05	0.00170056	-2.52844	5.00E-05	0.00170056
NM_197941.2	11174	ADAMTS6	7268	3.10697	2.94526	1.34298	1.21007	5.00E-05	0.00170056	1.13295	0.00025	0.00665625
NM_007231.3	11254	SLC6A14	4564	106.149	59.3198	29.2591	1.85914	5.00E-05	0.00170056	1.01963	5.00E-05	0.00170056
NM_007361.3	22795	NID2	5046	8.98417	5.57244	21.1646	-1.23619	5.00E-05	0.00170056	-1.92527	5.00E-05	0.00170056
NM_007350.3	22822	PHLDA1	5913	26.4457	14.7214	5.7379	2.20444	5.00E-05	0.00170056	1.35932	5.00E-05	0.00170056
NM_012309.3	22941	SHANK2	10495	2.13441	1.83589	4.74797	-1.15347	5.00E-05	0.00170056	-1.37083	5.00E-05	0.00170056
NM_025153.2	23120	ATP10B	7566	2.20884	10.5547	0.633624	1.80159	5.00E-05	0.00170056	4.05811	5.00E-05	0.00170056
NM_001033602.2	23281	MTUS2	6939	0.319814	0.311843	1.43098	-2.16171	5.00E-05	0.00170056	-2.19812	5.00E-05	0.00170056
NM_021149.2	23406	COTL1	1827	63.6715	38.2875	17.5866	1.85617	5.00E-05	0.00170056	1.1224	5.00E-05	0.00170056
NM_012198.3	25801	GCA	3226	17.687	14.0182	6.82261	1.3743	5.00E-05	0.00170056	1.0389	0.00015	0.00437065
NM_015444.2	25907	TMEM158	1859	3.13113	2.17294	0.548307	2.51363	5.00E-05	0.00170056	1.98659	0.00055	0.0124813
NM_015515.3	25984	KRT23	2147	25.4554	48.4544	10.4588	1.28325	5.00E-05	0.00170056	2.21191	5.00E-05	0.00170056
NM_207303.2	26033	ATRNL1	8732	3.52654	5.13191	8.98249	-1.34886	5.00E-05	0.00170056	-0.807618	0.00105	0.0207193
NM_015668.3	26166	RGS22	4184	9.38407	16.2235	28.5281	-1.6041	5.00E-05	0.00170056	-0.814297	0.00185	0.0317167
NM_012108.2	26228	STAP1	1511	13.8144	14.2708	5.70392	1.27615	5.00E-05	0.00170056	1.32304	5.00E-05	0.00170056
NM_015693.3	27152	INTU	3275	7.3657	11.515	20.2313	-1.4577	5.00E-05	0.00170056	-0.813071	0.00135	0.0250595
NM_198428.2	27241	BBS9	4019	12.957	15.278	27.5062	-1.08603	5.00E-05	0.00170056	-0.8483	0.00065	0.0142695
28526	28526	-	1510	8.43269	7.67714	2.50311	1.75227	5.00E-05	0.00170056	1.61685	5.00E-05	0.00170056
NM_001101312.1	28959	TMEM176B	1298	57.5183	51.8125	19.574	1.55508	5.00E-05	0.00170056	1.40436	5.00E-05	0.00170056
NM_014079.3	28999	KLF15	2525	1.22461	2.07674	4.99131	-2.0271	5.00E-05	0.00170056	-1.2651	0.00045	0.0106989
NM_013381.2	29953	TRHDE	5552	0.335649	0.875037	3.75703	-3.48457	5.00E-05	0.00170056	-2.10218	5.00E-05	0.00170056

NM_015914.5	51061	TXNDC11	3047	32.1322	30.3862	13.9276	1.20607	5.00E-05	0.00170056	1.12547	0.0001	0.00312189
NM_015931.1	51066	C3orf32	1545	0.348944	0.50087	2.79847	-3.00357	5.00E-05	0.00170056	-2.48213	0.0001	0.00312189
NM_016010.2	51101	FAM164A	3352	14.1508	17.1619	28.8579	-1.02809	5.00E-05	0.00170056	-0.749754	0.00185	0.0317167
NM_016459.3	51237	MZB1	827	76.7114	95.5045	25.3635	1.59669	5.00E-05	0.00170056	1.91282	5.00E-05	0.00170056
NM_016644.1	51334	PRR16	1764	2.36126	1.35465	0.422426	2.48279	5.00E-05	0.00170056	1.68115	0.0031	0.0465955
NM_016240.2	51435	SCARA3	3631	8.19438	8.51071	19.6595	-1.26252	5.00E-05	0.00170056	-1.20787	5.00E-05	0.00170056
NM_016529.4	51761	ATP8A2	5006	4.69777	1.86093	0.805169	2.54461	5.00E-05	0.00170056	1.20866	0.0027	0.0421065
NM_017423.2	51809	GALNT7	4307	27.9977	22.7749	11.8354	1.24221	5.00E-05	0.00170056	0.944341	0.00025	0.00665625
NM_018557.2	53353	LRP1B	16531	6.23523	5.0119	31.27	-2.32627	5.00E-05	0.00170056	-2.64135	5.00E-05	0.00170056
NM_144717.3	53833	IL20RB	2047	10.9365	11.4139	3.4085	1.68194	5.00E-05	0.00170056	1.74358	5.00E-05	0.00170056
NM_019050.2	54532	USP53	6595	32.0426	24.8874	91.3625	-1.51161	5.00E-05	0.00170056	-1.87618	5.00E-05	0.00170056
NM_001200049.1	54777	C10orf92	3164	1.01772	1.94165	4.63382	-2.18686	5.00E-05	0.00170056	-1.25492	0.0007	0.015106
NM_017709.3	54855	FAM46C	5720	61.9506	73.8697	27.8846	1.15165	5.00E-05	0.00170056	1.40551	5.00E-05	0.00170056
NM_017839.4	54947	LPCAT2	5388	20.1654	10.5208	6.29546	1.6795	5.00E-05	0.00170056	0.740861	0.00275	0.0426687
NM_018286.2	55273	TMEM100	1755	11.2743	20.2749	55.7012	-2.30467	5.00E-05	0.00170056	-1.45801	5.00E-05	0.00170056
NM_018407.4	55353	LAPTM4B	2238	39.8211	28.0431	15.4569	1.36528	5.00E-05	0.00170056	0.859399	0.0008	0.0168217
NM_001173463.1	55605	KIF21A	6530	23.7067	29.0202	50.1285	-1.08034	5.00E-05	0.00170056	-0.788572	0.0025	0.0397144
NM_018897.2	56171	DNAH7	12394	9.65151	24.3824	44.9611	-2.21985	5.00E-05	0.00170056	-0.882837	0.00135	0.0250595
NM_020130.3	56892	C8orf4	1833	124.593	69.3577	15.0847	3.04607	5.00E-05	0.00170056	2.20097	5.00E-05	0.00170056
NM_020183.3	56938	ARNTL2	1930	12.6242	9.51237	3.62337	1.80079	5.00E-05	0.00170056	1.39247	5.00E-05	0.00170056
NM_020299.4	57016	AKR1B10	1596	6.47911	3.95944	1.1011	2.55685	5.00E-05	0.00170056	1.84635	0.00035	0.00869612
NM_020406.2	57126	CD177	2364	9.13642	13.8532	1.36081	2.74716	5.00E-05	0.00170056	3.34768	5.00E-05	0.00170056
NM_207517.2	57188	ADAMTSL3	7336	1.00455	2.21176	5.16367	-2.36184	5.00E-05	0.00170056	-1.2232	5.00E-05	0.00170056
NM_020672.1	57402	S100A14	1043	36.358	27.7038	13.6965	1.40847	5.00E-05	0.00170056	1.01628	0.0012	0.0230244
NM_020693.2	57453	DSCAML1	6899	0.235658	0.346787	1.03849	-2.13973	5.00E-05	0.00170056	-1.58237	0.00035	0.00869612
NM_020802.2	57562	KIAA1377	7043	6.8155	9.97199	20.8934	-1.61616	5.00E-05	0.00170056	-1.0671	5.00E-05	0.00170056
NM_001099677.1	57604	KIAA1456	9300	3.24096	3.14456	9.92966	-1.61532	5.00E-05	0.00170056	-1.36794	0.00015	0.00437065
NM_020974.2	57758	SCUBE2	4536	1.66733	1.92054	6.48128	-1.95874	5.00E-05	0.00170056	-1.75476	5.00E-05	0.00170056
NM_021572.4	59084	ENPP5	2943	18.8783	22.3109	46.0303	-1.28585	5.00E-05	0.00170056	-1.04483	5.00E-05	0.00170056
NM_001156474.1	60494	CCDC81	2781	3.36532	3.25861	13.5553	-2.01004	5.00E-05	0.00170056	-2.05652	5.00E-05	0.00170056
NM_021928.3	60559	SPCS3	4596	88.1756	91.5712	42.2901	1.06006	5.00E-05	0.00170056	1.11458	5.00E-05	0.00170056
NM_022097.2	63928	CHP2	2366	2.0191	5.60138	12.9581	-2.68206	5.00E-05	0.00170056	-1.21	0.00025	0.00665625
NM_022136.3	64092	SAMSN1	1888	74.1493	34.2492	12.2431	2.59847	5.00E-05	0.00170056	1.48411	5.00E-05	0.00170056
NM_024164.5	64499	TPSB2	1166	15.8194	11.7505	5.04708	1.64818	5.00E-05	0.00170056	1.2192	0.0018	0.0310732
NM_031898.2	64518	TEKT3	1784	0.90014	1.5255	3.70959	-2.04304	5.00E-05	0.00170056	-1.28197	0.00275	0.0426687
NM_194071.2	64764	CREB3L2	7455	33.1735	25.6763	14.7643	1.16792	5.00E-05	0.00170056	0.798327	0.00055	0.0124813
NM_001031716.2	64859	OBFC2A	3849	56.9536	37.2567	22.2308	1.35723	5.00E-05	0.00170056	0.744942	0.00205	0.0341961
NM_023940.2	65997	RASL11B	1957	5.5833	5.9534	19.4123	-1.79778	5.00E-05	0.00170056	-1.70519	5.00E-05	0.00170056
NM_024329.5	79180	EFHD2	2419	29.5684	25.4191	12.1345	1.28494	5.00E-05	0.00170056	1.0668	5.00E-05	0.00170056
NM_024560.2	79611	ACSS3	3033	5.82008	7.52203	19.9558	-1.7777	5.00E-05	0.00170056	-1.40761	5.00E-05	0.00170056
NM_024574.3	79625	NDNF	2871	3.33565	2.32929	12.2793	-1.88018	5.00E-05	0.00170056	-2.39826	5.00E-05	0.00170056
NM_024677.4	79730	NSUN7	3698	6.82306	9.58667	21.2879	-1.64155	5.00E-05	0.00170056	-1.15094	5.00E-05	0.00170056
NM_024721.4	79776	ZFHX4	13958	3.27587	3.57141	9.54205	-1.54242	5.00E-05	0.00170056	-1.4178	5.00E-05	0.00170056
NM_024783.3	79841	AGBL2	3593	4.97866	9.67783	16.3753	-1.71769	5.00E-05	0.00170056	-0.758768	0.0031	0.0465955
NM_024913.4	79974	C7orf58	5320	5.19445	7.07342	14.1535	-1.44612	5.00E-05	0.00170056	-1.00068	0.00015	0.00437065
NM_001142966.1	80000	GREB1L	6052	1.16724	0.909657	5.39672	-2.20898	5.00E-05	0.00170056	-2.56869	5.00E-05	0.00170056
NM_025159.2	80231	CXorf21	1927	9.49256	7.87925	3.16143	1.58622	5.00E-05	0.00170056	1.31748	0.0001	0.00312189
NM_025208.4	80310	PDGFD	3993	17.927	14.3447	50.6722	-1.49906	5.00E-05	0.00170056	-1.82067	5.00E-05	0.00170056
NM_001080427.1	80731	THSD7B	5846	0.413448	0.298006	2.51427	-2.60436	5.00E-05	0.00170056	-3.07673	5.00E-05	0.00170056
NM_030782.3	81037	CLPTM1L	2148	42.3752	35.1958	20.7687	1.02881	5.00E-05	0.00170056	0.760991	0.00185	0.0317167
NM_030810.3	81567	TXNDC5	3231	313.722	328.051	108.516	1.53158	5.00E-05	0.00170056	1.59601	5.00E-05	0.00170056
NM_030926.4	81618	ITM2C	2073	59.6746	57.9318	24.8347	1.26476	5.00E-05	0.00170056	1.222	5.00E-05	0.00170056
NM_030938.3	81671	VMP1	2176	117.521	73.8535	42.9597	1.45186	5.00E-05	0.00170056	0.781681	0.0011	0.0214605
NM_031422.4	83539	CHST9	2246	49.8727	59.9675	118.083	-1.24347	5.00E-05	0.00170056	-0.977543	0.00025	0.00665625

NM_031457.1	83661	MS4A8B	1353	15.5796	37.2902	72.4867	-2.21806	5.00E-05	0.00170056	-0.95892	5.00E-05	0.00170056
NM_031935.2	83872	HMCN1	18212	27.1518	37.6918	11.1751	1.28077	5.00E-05	0.00170056	1.75397	5.00E-05	0.00170056
NM_032048.2	84034	EMILIN2	4009	16.7502	11.5183	5.86863	1.51308	5.00E-05	0.00170056	0.972827	0.0002	0.00554203
NM_032119.3	84059	GPR98	19333	3.9328	4.91004	18.2589	-2.21497	5.00E-05	0.00170056	-1.89479	5.00E-05	0.00170056
NM_032131.4	84071	ARMC2	3413	6.61246	11.2294	18.9244	-1.51698	5.00E-05	0.00170056	-0.75297	0.00275	0.0426687
NM_032291.2	84251	SGIP1	4694	6.60444	2.73182	1.11917	2.56101	5.00E-05	0.00170056	1.28743	0.00075	0.0159498
NM_032532.2	84624	FNDC1	6551	1.57238	1.0843	4.36865	-1.47424	5.00E-05	0.00170056	-2.01042	5.00E-05	0.00170056
NM_032704.3	84790	TUBA1C	1553	38.9999	28.469	14.6569	1.41189	5.00E-05	0.00170056	0.957816	0.00045	0.0106989
NM_001099294.1	85352	KIAA1644	6741	0.722345	0.920432	3.00503	-2.05662	5.00E-05	0.00170056	-1.707	5.00E-05	0.00170056
NM_014903.4	89795	NAV3	9758	2.21698	3.01626	5.85657	-1.40146	5.00E-05	0.00170056	-0.957295	0.00045	0.0106989
NM_033280.2	90701	SEC11C	782	151.468	211.442	70.3127	1.10716	5.00E-05	0.00170056	1.58841	5.00E-05	0.00170056
NM_032160.2	92126	DSEL	9281	2.92292	3.8337	6.57699	-1.17001	5.00E-05	0.00170056	-0.77869	0.00295	0.0449329
NM_001136103.2	92293	TMEM132C	4947	0.257211	0.626457	5.482	-4.41368	5.00E-05	0.00170056	-3.12942	5.00E-05	0.00170056
NM_052863.2	92304	SCGB3A1	466	4.88362	8.45061	80.549	-4.04384	5.00E-05	0.00170056	-3.25274	5.00E-05	0.00170056
NM_001040272.5	92949	ADAMTSL1	7843	1.25487	1.80434	5.358	-2.09416	5.00E-05	0.00170056	-1.57022	5.00E-05	0.00170056
NM_144577.3	93233	CCDC114	3220	2.15727	4.41656	9.73158	-2.17346	5.00E-05	0.00170056	-1.13975	0.00015	0.00437065
NM_022073.3	112399	EGLN3	2722	22.4964	40.2903	6.64	1.76044	5.00E-05	0.00170056	2.60118	5.00E-05	0.00170056
NM_138426.2	113263	GLCCI1	4745	16.8508	17.5286	7.93569	1.08639	5.00E-05	0.00170056	1.14328	5.00E-05	0.00170056
NM_052904.3	114792	KLHL32	3807	1.03989	1.82112	4.80965	-2.20949	5.00E-05	0.00170056	-1.40111	0.00015	0.00437065
NM_052947.3	115701	ALPK2	7303	6.96329	6.02987	0.905442	2.94308	5.00E-05	0.00170056	2.73543	5.00E-05	0.00170056
NM_054114.3	117289	TAGAP	3363	17.2271	17.5662	7.31083	1.23657	5.00E-05	0.00170056	1.2647	5.00E-05	0.00170056
NM_145170.3	118491	TTC18	3704	10.4075	20.0308	40.6025	-1.96394	5.00E-05	0.00170056	-1.01935	5.00E-05	0.00170056
NM_001008781.2	120114	FAT3	19048	0.287291	0.611261	5.86196	-4.3508	5.00E-05	0.00170056	-3.26152	5.00E-05	0.00170056
NM_001098526.1	120425	AMICA1	1957	62.688	28.6569	14.4784	2.11429	5.00E-05	0.00170056	0.984977	0.0022	0.0361165
NM_152860.1	121340	SP7	2974	0.15683	0.382827	2.13551	-3.76731	5.00E-05	0.00170056	-2.47982	5.00E-05	0.00170056
NM_145172.3	126820	WDR63	3007	6.83313	18.9917	31.7857	-2.21776	5.00E-05	0.00170056	-0.743008	0.0028	0.0432452
NM_152376.3	127733	UBXN10	2972	12.5561	28.6263	47.6268	-1.92338	5.00E-05	0.00170056	-0.734432	0.00195	0.0329693
NM_152377.2	127795	C1orf87	2028	5.88121	16.2069	29.0239	-2.30306	5.00E-05	0.00170056	-0.840636	0.00115	0.0222874
NM_080607.2	128434	VSTM2L	1966	2.37048	3.29723	9.42846	-1.99184	5.00E-05	0.00170056	-1.51577	5.00E-05	0.00170056
NM_001085447.1	129881	C2orf77	2169	6.0993	12.3097	21.6062	-1.82473	5.00E-05	0.00170056	-0.811648	0.003	0.0454886
NM_001127391.1	130540	ALS2CR12	1944	3.72552	8.60666	16.0725	-2.10908	5.00E-05	0.00170056	-0.901067	0.00275	0.0426687
NM_182500.2	130813	C2orf50	1056	1.11889	2.72954	6.83274	-2.61039	5.00E-05	0.00170056	-1.3238	0.002	0.0335868
NM_138806.3	131450	CD200R1	2272	18.6071	9.34796	4.05556	2.19788	5.00E-05	0.00170056	1.20475	0.0009	0.0184743
NM_130830.4	131578	LRRC15	5883	0.928249	0.527111	4.55081	-2.29354	5.00E-05	0.00170056	-3.10995	5.00E-05	0.00170056
NM_080826.1	140862	ISM1	2592	0.361083	0.451919	3.53493	-3.29128	5.00E-05	0.00170056	-2.96754	5.00E-05	0.00170056
NM_001018071.3	143162	FRMPD2	5024	2.91976	5.3588	10.6401	-1.86558	5.00E-05	0.00170056	-0.989524	0.00015	0.00437065
NM_001135091.1	143662	MUC15	3392	7.76574	14.0165	27.42	-1.82003	5.00E-05	0.00170056	-0.968098	0.0001	0.00312189
XM_001715090.3	144535	C12orf55	5721	7.68912	16.3407	29.9599	-1.96214	5.00E-05	0.00170056	-0.874561	0.00035	0.00869612
NM_173615.3	146177	VWA3A	4600	3.97837	5.90151	11.1983	-1.49303	5.00E-05	0.00170056	-0.92412	0.00025	0.00665625
NM_020877.2	146754	DNAH2	13505	3.60199	5.95256	10.7199	-1.57343	5.00E-05	0.00170056	-0.848715	0.00045	0.0106989
NM_133459.3	147372	CCBE1	6260	0.377326	0.319426	3.31121	-3.13348	5.00E-05	0.00170056	-3.37381	5.00E-05	0.00170056
NM_153221.2	148113	CILP2	4199	0.0646116	0.229136	2.31852	-5.16527	5.00E-05	0.00170056	-3.33893	5.00E-05	0.00170056
NM_173354.3	150094	SIK1	4706	45.1877	14.1567	4.40303	3.35936	5.00E-05	0.00170056	1.68492	5.00E-05	0.00170056
NM_145912.5	150372	NFAM1	5605	2.98475	2.30641	1.12952	1.4019	5.00E-05	0.00170056	1.02994	0.00295	0.0449329
NM_153228.2	162282	ANKFN1	2426	3.15844	4.73041	10.9039	-1.78755	5.00E-05	0.00170056	-1.2048	0.00025	0.00665625
NM_152598.2	162333	MARCH10	3101	1.5887	3.47258	8.47365	-2.41514	5.00E-05	0.00170056	-1.28697	5.00E-05	0.00170056
NM_199280.2	165186	FAM179A	3548	3.82214	5.7623	10.5229	-1.46108	5.00E-05	0.00170056	-0.868811	0.0021	0.0348574
NM_153362.2	167681	PRSS35	2503	1.18412	1.60902	5.56998	-2.23386	5.00E-05	0.00170056	-1.79149	5.00E-05	0.00170056
NM_144980.3	168090	C6orf118	1839	6.11382	12.7925	25.5535	-2.06337	5.00E-05	0.00170056	-0.998217	0.0003	0.00772176
NM_152632.3	170063	CXorf22	3613	7.50052	16.8435	28.8899	-1.9455	5.00E-05	0.00170056	-0.778376	0.00155	0.0278206
NM_153183.2	170685	NUDT10	2001	22.8247	13.0267	3.37292	2.75853	5.00E-05	0.00170056	1.9494	5.00E-05	0.00170056
NM_207437.3	196385	DNAH10	13680	6.57225	11.213	23.8282	-1.85821	5.00E-05	0.00170056	-1.08749	0.0001	0.00312189
NM_173543.2	199221	DZIP1L	3498	4.02872	5.60001	10.6107	-1.39713	5.00E-05	0.00170056	-0.922023	0.00225	0.0367046
NM_001004303.4	199920	C1orf168	3460	2.06803	4.61639	10.1833	-2.29988	5.00E-05	0.00170056	-1.14137	0.0001	0.00312189

NM_001195811.1	200150	PLD5	3018	0.587491	0.576669	7.00164	-3.57505	5.00E-05	0.00170056	-3.60188	5.00E-05	0.00170056
NM_144992.4	200403	VWA3B	4747	5.95631	10.3993	18.6574	-1.64726	5.00E-05	0.00170056	-0.843257	0.00045	0.0106989
NM_178504.4	201625	DNAH12	9542	12.8389	25.6993	45.6945	-1.83149	5.00E-05	0.00170056	-0.830292	0.003	0.0454886
NM_145000.3	202151	RANBP3L	2543	0.511382	0.806251	2.49278	-2.28528	5.00E-05	0.00170056	-1.62846	0.00025	0.00665625
NM_173549.2	203111	C8orf47	1550	8.76943	12.5778	26.9158	-1.6179	5.00E-05	0.00170056	-1.09757	0.00065	0.0142695
NM_152750.4	222256	CDHR3	6500	11.7392	27.2359	50.6512	-2.10926	5.00E-05	0.00170056	-0.895088	0.0006	0.013408
NM_172239.4	254958	REXO1L1	7032	3.62701	13.632	24.4886	-2.75526	5.00E-05	0.00170056	-0.845111	0.0018	0.0310732
NM_194302.2	255101	CCDC108	5945	1.19487	2.83059	5.20581	-2.12327	5.00E-05	0.00170056	-0.879021	0.0029	0.0443924
NM_025015.2	259217	HSPA12A	5722	1.47978	1.6324	3.58952	-1.27841	5.00E-05	0.00170056	-1.1368	0.0005	0.0116249
NM_152997.2	260436	C4orf7	539	100.371	600.743	19.3682	2.37358	5.00E-05	0.00170056	4.95499	5.00E-05	0.00170056
NM_174975.4	266629	SEC14L3	2084	10.9827	1.07783	54.4952	-2.3109	5.00E-05	0.00170056	-5.65992	5.00E-05	0.00170056
NM_173812.4	283417	DPY19L2	4057	2.20282	1.55026	8.58391	-1.96228	5.00E-05	0.00170056	-2.46912	5.00E-05	0.00170056
NM_173833.5	286133	SCARA5	4151	3.36922	4.76469	11.485	-1.76926	5.00E-05	0.00170056	-1.26929	5.00E-05	0.00170056
NM_001012502.2	286207	C9orf117	1722	5.48466	11.108	28.102	-2.3572	5.00E-05	0.00170056	-1.33907	5.00E-05	0.00170056
NM_173695.2	286464	CXorf59	1956	6.9319	13.6792	24.0057	-1.79205	5.00E-05	0.00170056	-0.811391	0.00215	0.0355296
XM_002343299.3	319089	TTC6	2913	6.0073	6.07558	14.5005	-1.27132	5.00E-05	0.00170056	-1.25501	5.00E-05	0.00170056
NM_199289.1	341676	NEK5	2912	5.07284	13.2112	22.3474	-2.13924	5.00E-05	0.00170056	-0.758343	0.0025	0.0397144
NM_198505.2	344905	ATP13A5	3657	7.9423	1.33878	0.202547	5.29323	5.00E-05	0.00170056	2.72459	5.00E-05	0.00170056
NM_198285.2	349136	WDR86	2041	8.14617	6.01338	25.095	-1.6232	5.00E-05	0.00170056	-2.06115	5.00E-05	0.00170056
XM_003118942.2	374467	C12orf63	2401	6.2977	12.83	24.4605	-1.95756	5.00E-05	0.00170056	-0.930935	0.0004	0.00971957
NM_213606.3	387700	SLC16A12	4622	3.39611	3.4552	19.0353	-2.48672	5.00E-05	0.00170056	-2.46183	5.00E-05	0.00170056
NM_001003845.2	389058	SP5	1947	0.499975	0.622839	2.53688	-2.34313	5.00E-05	0.00170056	-2.02613	0.0001	0.00312189
NM_205855.3	389558	FAM180A	1801	0.482871	0.856059	3.18214	-2.72029	5.00E-05	0.00170056	-1.89421	0.0006	0.013408
NM_001001557.2	392255	GDF6	3701	0.151771	0.214651	1.32606	-3.12718	5.00E-05	0.00170056	-2.62708	5.00E-05	0.00170056
NM_001170820.3	402778	IFITM10	3709	0.698434	1.00254	2.86259	-2.03513	5.00E-05	0.00170056	-1.51367	0.0003	0.00772176
NM_001080534.1	440279	UNC13C	8140	1.18901	1.09559	4.96343	-2.06158	5.00E-05	0.00170056	-2.17963	5.00E-05	0.00170056
NM_001010922.2	440603	BCL2L15	4973	4.73389	6.0172	1.73169	1.45085	5.00E-05	0.00170056	1.79691	5.00E-05	0.00170056
XM_001716834.2	642131	LOC642131	570	49.3887	81.0706	11.6645	2.08206	5.00E-05	0.00170056	2.79705	5.00E-05	0.00170056
NM_001098843.3	645090	CXorf30	2863	3.5013	6.73563	15.3101	-2.12852	5.00E-05	0.00170056	-1.1846	0.00015	0.00437065
NM_001013647.1	646851	LOC646851	10322	1.77391	2.98194	5.49157	-1.63028	5.00E-05	0.00170056	-0.880965	0.00075	0.0159498
XM_003118626.2	100506209	LOC10050620	3519	1.21469	2.3406	4.65572	-1.93842	5.00E-05	0.00170056	-0.992128	0.00305	0.04606
XM_003118966.1	100506711	LOC10050671	1898	4.06174	11.1922	20.2473	-2.31756	5.00E-05	0.00170056	-0.855238	0.0022	0.0361165
XM_003118889.1	100506859	LOC10050685	703	5.49813	9.52558	22.1987	-2.01346	5.00E-05	0.00170056	-1.2206	0.0015	0.0270821
XM_003403451.1	100508736	LOC10050873	1567	5.48834	10.1482	19.0839	-1.79792	5.00E-05	0.00170056	-0.91113	0.0024	0.03848

Table S7. Summary of patient characteristics.

	Control	ECRSwNP	non-ECRSwNP
Total no. of subjects	34	35	31
Sex, male, n (%)	14(41.2)	23(65.7)	15(48.3)
Age (y), mean ± SD	46.0±14.1	43.0±11.7	45.2±10.4
Positive Phadiatop result, n (%)	2(5.9)	10(28.6)	5(16.1)
Asthma in history, n (%)	0(0)	7(20.0)	0(0)
Peripheral blood eosinophil count (10^9/L), mean ± SD	0.12±0.07	0.40±0.14	0.15±0.08
Peripheral blood eosinophil percentage (%), mean ± SD	1.84±1.05	5.62±2.26	2.28±1.04
Mean tissue eosinophil count*, mean ± SD	1.5±1.2	134.9±72.3	4.2±2.3
Total serum IgE (KU/L), mean ± SD	60.8±104.3	140.2±121.0	81.8±100.4
Methodology used			
RNA sequencing	n=3(1 male)	n=3(3 male)	n=3(1 male)
Age (y), mean ± SD	48.7±7.6	46.3±3.5	47.3±6.1
qRT-PCR	n=31	n=32	n=28
Age (y), mean ± SD	45.7±14.7	42.7±12.1	45.0±10.8
in situ hybridization	n=5	n=5	n=5
Age (y), mean ± SD	46.8±16.8	33.2±9.8	34.6±8.8

*Mean of the eosinophil count of 5 separate HPFs.

Table S8. Primer pairs used for qRT-PCR experiments.

lncRNA Name	Forward Primer (5'-3')	Reverse Primer (5'-3')
TCONS_00032728	TCCGTTTGATTCCGTTTGATGT	CATCGAATGAATTGAATGCAATCA
TCONS_00070847	AGGAGTAGCTTAGCAGCAGGAATG	CCTGCGTCACATTCCATCAAG
TCONS_00208641	GCAGAAGACAGAGATGAAGAAAAGAA	GCGTGGTTTCCTGTGCTCAT
TCONS_00223831	AAACAAGTCTGGGTGGTGCTTG	CTCCCTGAAGTTGTAAATCCCAGAAC
TCONS_00254761	CCCATCTGAAAACAGCAACACAG	CCTGTGGCAGATGCAGTTTTAC
XLOC_010280	ACTGGGAAGCCACAAGAAAAGATT	ACAGTGGTCAGTCCTTTCTTGGCT
CCL18	CGTCTATACCTCCTGGCAGATTCC	TTAGGAGGATGACACCTGGCTTG
